# Redox Reorganization: Aluminium Promoted 1,5‐Hydride Shifts Allow the Controlled Synthesis of Multisubstituted Cyclohexenes

**DOI:** 10.1002/anie.202307424

**Published:** 2023-07-21

**Authors:** Lewis B. Smith, Roly J. Armstrong, Jingyan Hou, Edward Smith, Ming Sze, Alistair J. Sterling, Alex Smith, Fernanda Duarte, Timothy J. Donohoe

**Affiliations:** ^1^ Chemistry Research Laboratory University of Oxford OX1 3TA Oxford UK; ^2^ School of Natural and Environmental Sciences Newcastle University NE1 7RU Newcastle Upon Tyne UK; ^3^ Syngenta, Jealott's Hill International Research Centre RG42 6EY Bracknell Berkshire UK

**Keywords:** 1,5-Hydride Shift, Aluminium, Cyclohexene, Ring Formation, Selective

## Abstract

An efficient synthesis of cyclohexenes has been achieved from easily accessible tetrahydropyrans via a tandem 1,5‐hydride shift–aldol condensation. We discovered that readily available aluminium reagents, e.g. Al_2_O_3_ or Al(O^*t*^Bu)_3_ are essential for this process, promoting the 1,5‐hydride shift with complete regio‐ and enantiospecificity (in stark contrast to results obtained under basic conditions). The mild conditions, coupled with multiple methods available to access the tetrahydropyran starting materials makes this a versatile method with exceptional functional group tolerance. A wide range of cyclohexenes (>40 examples) have been prepared, many in enantiopure form, showing our ability to selectively install a substituent at each position around the newly forged cyclohexene ring. Experimental and computational studies revealed that aluminium serves a dual role in facilitating the hydride shift, activating both the alkoxide nucleophile and the electrophilic carbonyl group.

## Graphical abstract

An efficient synthesis of multisubstituted cyclohexenes has been achieved from tetrahydropyrans (easily accessible via many synthetic approaches). The process operates via a tandem 1,5‐hydride shift–aldol condensation mediated by simple aluminium reagents (e.g. Al_2_O_3_), enabling the synthesis of cyclohexenes (>40 examples) bearing substituents at each position around the cyclohexene ring with high levels of chemo‐, stereo‐ and regioselectivity.

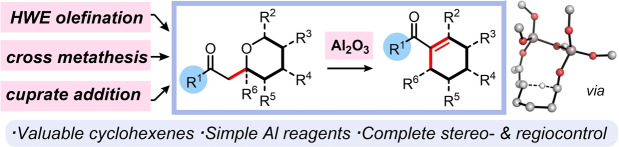

## Introduction

**Scheme 1:**
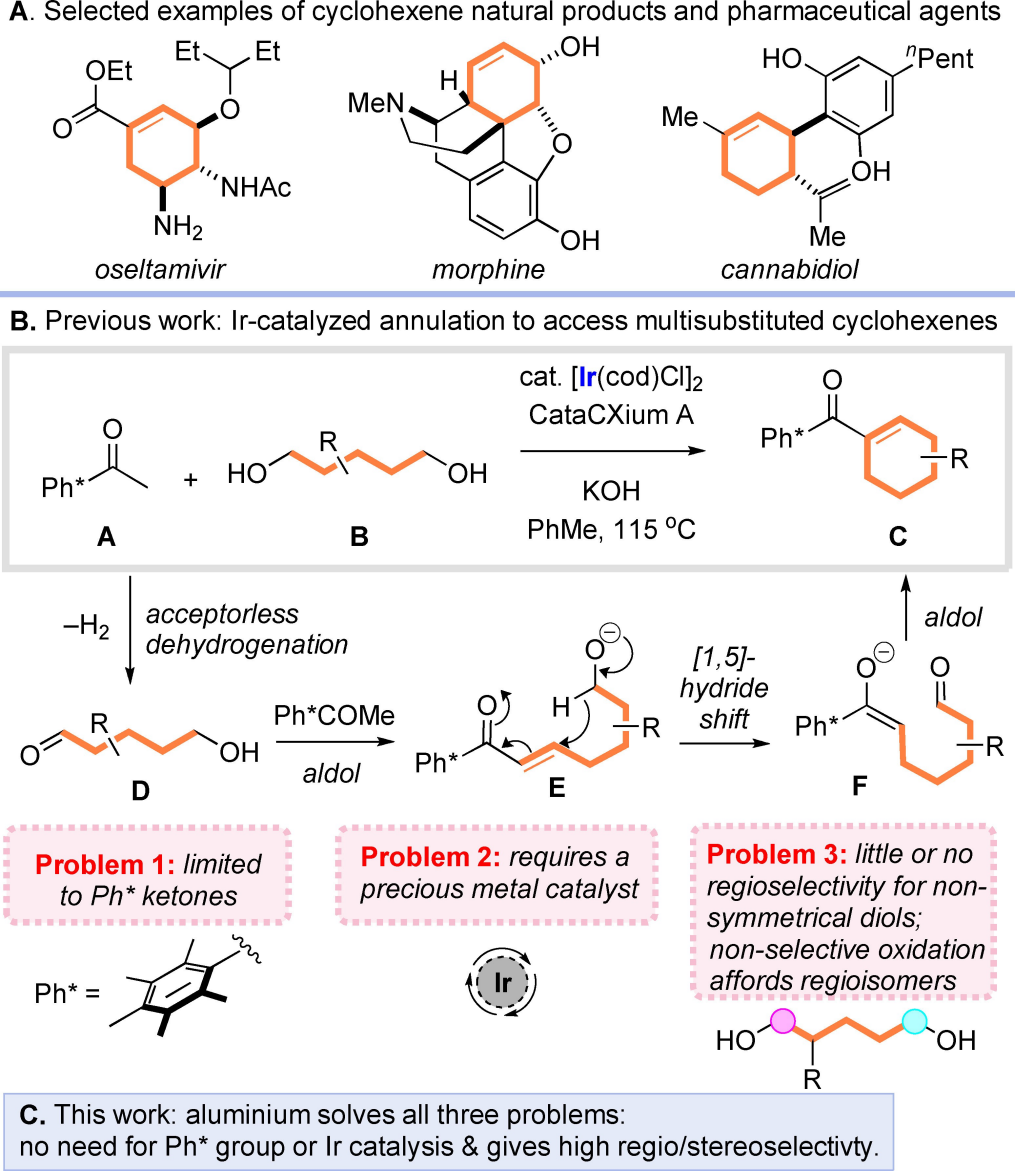
Previous work: opportunities and challenges for the synthesis of cyclohexenes from 1,5‐diols.

Multisubstituted cyclohexenes are ubiquitous building blocks for organic synthesis, and are prevalent amongst pharmaceutical agents, natural products and functional materials (Scheme 1A).^[1]^

Many impressive methods have been reported for the synthesis of such materials, including Diels–Alder cycloaddition,^[2]^ partial reduction of aromatic precursors,^[3]^ and ring closing metathesis.^[4]^ However, all of these reactions rely on the availability of appropriately substituted precursors, and often require the use of harsh conditions or expensive catalysts. As such the development of new and powerful approaches for the synthesis of cyclohexene containing materials is highly desirable.

Building upon work by our group on annulative hydrogen borrowing reactions,^[5]^ we recently reported a new method for the synthesis of cyclohexenes via a reaction between pentamethylacetophenone (**A**) and substituted 1,5‐diols (**B**) (Scheme 1B).[^6^, ^7^] This process was mediated by a commercially available iridium(I) catalyst, modified by the bulky trialkyl phosphine ligand CataCXium A, and enabled direct access to multisubstituted cyclohexenes (**C**). Importantly, the pentamethylphenyl (Ph*) group proved essential for this chemistry, and other less sterically hindered ketones proved completely untenable in the desired annulation reaction. A detailed mechanistic interrogation revealed that the process takes place via initial acceptorless dehydrogenation of the diol to form hydroxyaldehyde **D**, which could condense with the aryl ketone to form an enone (**E**) that is poised to undergo the key step: an intramolecular 1,5‐hydride shift.^[8]^ Final intramolecular aldol condensation of the resulting enolate (**F**) delivered the corresponding cyclohexene products.

This reaction represents a conceptually new approach for the synthesis of cyclohexenes, but suffers from several critical drawbacks. Firstly, as mentioned above, the process displays a stringent requirement for the use of Ph* ketones, and fails completely when applied to other ketones (the olefin group in the products also precludes cleavage of the Ph* group with Br_2_ and necessitates the use of strong acid).^[9]^ Notably, related hydrogen borrowing methods targeting acyl cyclohexanes developed within our group and by others are also limited to the preparation of *ortho*‐substituted ketones.^[5]^ Secondly, the reaction relies upon an expensive precious metal catalyst. Thirdly, and perhaps most importantly, when non‐symmetrical 1,5‐diols are employed, regioisomeric mixtures of the corresponding hydroxyaldehyde are produced leading to inseparable mixtures of cyclohexene products, and thereby limiting the methodology to the synthesis of symmetrical or sterically biased cyclohexenes.

Our aim at the outset of this work was to develop a new transition metal free method for cyclohexene synthesis, capable of overcoming all three of these challenges. In this article, we describe how our initial attempts to this end were thwarted by an interesting and unexpected isomerization pathway. Ultimately, we were able to overcome this issue, using readily available aluminium reagents to develop an extremely general and mild synthesis of acyl cyclohexenes. The aluminium mediated process proceeds with complete regio‐ and stereocontrol, and tolerates a remarkably wide range of aliphatic and aromatic ketones.

## Results and Discussion

### First Generation Approach

**Scheme 2:**
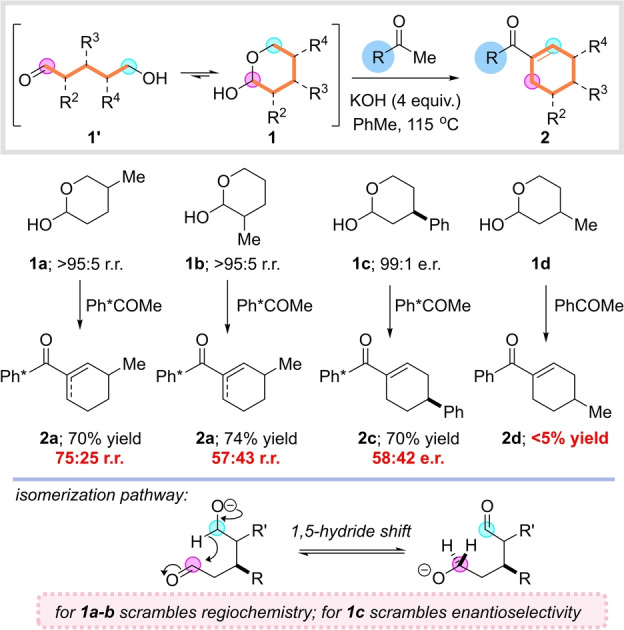
Preliminary results: first‐generation transition‐metal free annulation.

The mechanistic analysis presented in Scheme 1 suggested that if we were to replace the diol starting material (**B**) with a preformed hydroxyaldehyde (**D**), the resulting process would have no requirement for a transition‐metal catalyst (it being solely involved in the initial oxidation). Moreover, non‐symmetrical hydroxyaldehydes would be expected to react to afford regioisomerically pure cyclohexenes, thus solving another of the drawbacks of our initial method. With this in mind we set out to synthesize several model hydroxyaldehydes **1′** by reduction of the corresponding lactones with DIBAL‐H (as shown in Scheme 2, these were observed to exist exclusively as the lactols **1**, see Supporting Information for full details). We began by investigating the reaction between pentamethylacetophenone and a δ‐substituted lactol **1 a** (Scheme 2). Promisingly, this transition metal free reaction gave a good yield of the cyclohexene product **2 a** (70 % yield), but we were dismayed to discover extensive erosion of the regiochemistry had occurred to deliver a mixture of C3‐ and C5‐ substituted products (75 : 25 r.r.). With a β‐substituted lactol **1 b**, the problem was even more pronounced, and the undesired C3‐substituted cyclohexene **2 a** was still observed as the major product. We speculated that an unusual isomerization pathway was operative in which the open‐chain alkoxyaldehyde intermediate underwent a base mediated 1,5‐hydride shift, causing the defined regiochemistry of the lactol to be lost (Scheme 2, bottom). This hypothesis was supported by the observation that an enantioenriched γ‐substituted lactol **1 c** reacted to afford the corresponding cyclohexene **2 c** with extensive racemization (58 : 42 e.r.). This isomerization pathway, which causes a loss of both stereochemical and regiochemical integrity, threatened to completely derail our proposed strategy for selective cyclohexene synthesis. A final serious issue was that this first‐generation approach was still limited to pentamethylacetophenone—with acetophenone as the aldol coupling partner, a complex mixture of products was obtained, suggesting problems with chemoselectivity in the aldol reaction. It was clear that in order to develop a practical and general synthesis of cyclohexenes, we would need to solve these three significant challenges of stereo‐, regio‐ and chemoselectivity.

### Second Generation Approach

With this in mind, we decided to investigate alternative and less basic methods for C=C bond formation, hoping to prevent the undesired hydroxyaldehyde isomerization and also escape the requirement for a Ph* group (our previous work suggested that strongly basic conditions can cause undesired Meerwein‐Ponndorf‐Verley reduction of non‐Ph* ketones). After some experimentation, we were delighted to find that lactol **1 d** underwent smooth Horner–Wadsworth–Emmons (HWE) olefination with phenyl‐substituted phosphonoacetate **3**.^[10]^ Notably, the hydroxyenone **5 d** initially produced in the HWE reaction underwent spontaneous cyclization to form tetrahydropyran **4 d**, which was isolated as the sole product in 92 % yield. Similar cyclizations were observed for all other hydroxyaldehydes involved in this study, and appear to be inconsequential, with the tetrahydropyran simply serving as a masked form of the hydroxyenone **5** in situ. We then set out to identify conditions for the transformation of this tetrahydropyran intermediate to cyclohexene **2 d**. Treatment of **4 d** with KOH afforded a trace amount of the desired product along with a complex mixture of side products (Table 1, entry 1). Weaker bases returned only unreacted tetrahydropyran (entry 2–3), and stronger bases led to decomposition of the starting material (entries 4–5). We wondered whether Lewis acids might also be able to affect the desired 1,5‐hydride shift. We initially tested magnesium, titanium and boron reagents, which did not promote the desired hydride shift (entries 6–8). Promisingly, when we evaluated Al(O^*i*^Pr)_3_, **2 d** was formed in 25 % yield, but competing Meerwein‐Ponndorf‐Verley reduction of **5 d** was observed (entry 9). We therefore investigated Al(O^*t*^Bu)_3_, which lacks a nucleophilic β‐hydride, and were delighted to isolate **2 d** in 97 % yield (entry 10). Sub‐stoichiometric loadings of Al(O^*t*^Bu)_3_ led to significantly reduced yield (entry 11) and a lower yield with Mg(O^*t*^Bu)_2_ confirmed Al as the optimal metal (entry 12). Remarkably, we found that various types of alumina could also promote the desired reaction, affording **2 d** in 96 % isolated yield when 5 eq. of basic alumina were employed (entries 13–17).

**Table 1:** A second‐generation cyclohexene synthesis.^[a]^

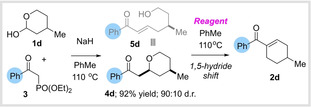			
Entry	*Reagent* (eq.)	Yield **4 d**/%^[b]^	Yield **2 d**/%^[b]^
1	KOH (4)	<5	8
2	Cs_2_CO_3_ (3)	100	<5
3	^*i*^Pr_2_NEt (3)	99	<5
4	KHMDS (3)	<5	<5
5	NaH (3)	<5	<5
6	MgCl_2_ (1)	78	<5
7	Ti(O^*i*^Pr)_4_ (1)	<5	<5
8	BF_3_⋅OEt_2_ (1)	<5	<5
9^[c]^	Al(O^*i*^Pr)_3_ (1)	15	25
**10**	**Al(O** ^***t***^ **Bu)_3_ (1)**	**<5**	**(97)**
11	Al(O^*t*^Bu)_3_ (0.5)	20	(57)
12	Mg(O^*t*^Bu)_2_ (1)	<5	(67)
13	Al_2_O_3_ (neutral) (1)	52	36
14	Al_2_O_3_ (nanoparticles) (1)	36	42
15	Al_2_O_3_ (acidic) (1)	36	48
16	Al_2_O_3_ (basic) (1)	(43)	(48)
**17**	**Al_2_O_3_ (basic) (5)**	**<5**	**(96)**

[a] Conditions: (i) HWE reagent (1.4 eq.), NaH (1.2 eq.), PhMe, r.t., 20 min *then* lactol (1 eq.), 110 °C, 16 h; (ii) tetrahydropyran (1 eq.), reagent (1–5 eq.), PhMe, 110 °C, 16 h. [b] Determined by ^1^H NMR analysis *vs* CHCl_2_CHCl_2_ as an internal standard; values in parentheses indicate the yield of isolated product. [c] Reduced product was isolated in 26 % yield (see Supporting Information).

**Scheme 3:**
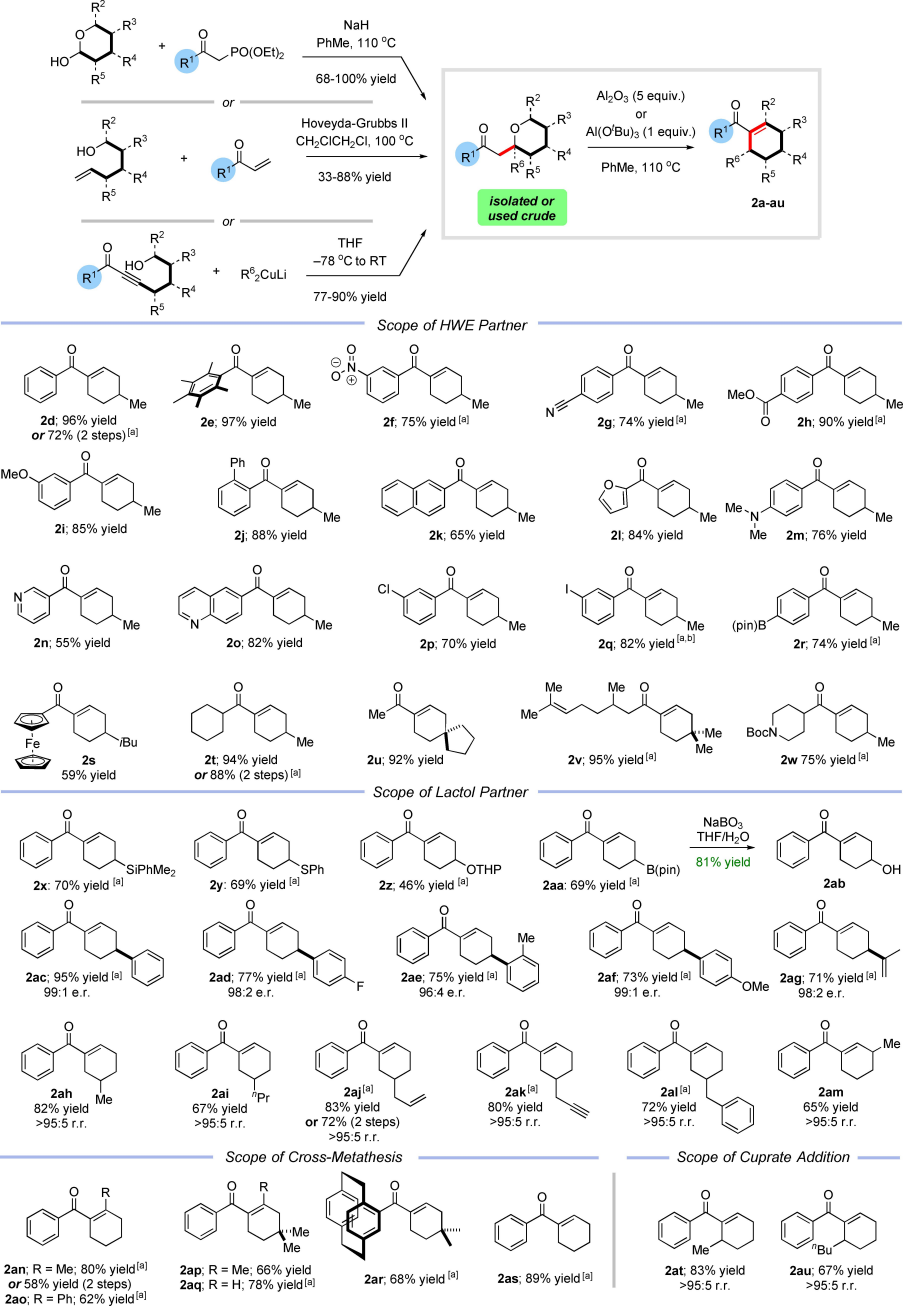
Chemo‐, regio‐ and enantiocontrolled synthesis of cyclohexenes; r.r=regiosiomeric ratio. For experimental details of HWE, cross‐metathesis, and cuprate addition reactions and characterization of tetrahydropyrans, see Supporting Information. Conditions for 1,5‐hydride shift: tetrahydropyran (1 eq.), basic Al_2_O_3_ (5 eq.), 110 °C, PhMe, 16 h. Yields are quoted for the 1,5‐hydride shift and refer to isolated material after column chromatography. [a] with Al(O^*t*^Bu)_3_ (1 eq.) instead of Al_2_O_3_. [b] HWE reaction with LiN^*i*^Pr_2_ instead of NaH.

With a second‐generation approach established, we evaluated its generality, initially focusing on the HWE partner (Scheme 3).

We were pleased to find that Ph* ketones were still tolerated in the process and **2 e** was isolated in 97 % yield. The reaction also tolerated aryl ketones substituted with both electron withdrawing and donating groups (**2 f**–**2 i**, **2 m**). Sterically demanding acyl cyclohexenes were obtained in high yields (**2 j** and **2 k**) and heterocyclic groups were tolerated, including furans (**2 l**), pyridines (**2 n**) and quinolines (**2 o**). The functional group compatibility of this method is particularly noteworthy; cyclohexenes substituted with halogens (**2 p** and **2 q**), boronic esters (**2 r**) and metallocenes (**2 s**) were all isolated in high yields, with no evidence of competing side reactions. The chemistry was not restricted to aromatic ketones and a series of aliphatic and saturated heterocyclic examples were prepared in high yields (**2 t**–**2 w**).

We next moved to investigate the scope of the lactol partner, focusing initially on examples bearing heavily functionalizable atoms such as Si, B, S and even protected oxygen, all of which would have been poorly tolerated under the strongly basic reaction conditions of our first‐generation approach. We were delighted to find that the corresponding cyclohexene products **2 x**–**2 aa** were obtained in uniformly good to yields with no evidence of competing side reactions. The only failure lay with the direct introduction of a hydroxyl group at C‐4 in which case a complex reaction mixture was observed. However, we were pleased to find that hydroxylated cyclohexene **2 ab** could be efficiently accessed by chemoselective oxidation of the boron atom in **2 aa** (or via deprotection of the THP protected analogue **2 z**, see Supporting Information). The full range of potential heteroatom derivatization reactions is very large and so this aspect of the methodology greatly increases the scope of accessible compounds (for example **2 y** could be easily oxidized to the corresponding sulfoxide or sulfone—see Supporting Information, not shown). We next turned our attention to enantiopure C3‐substituted examples that had racemized under our first‐generation conditions. We were delighted to find that with our new method, a series of enantioenriched lactols reacted to generate cyclohexenes **2 ac**–**2 ag** in high yields with essentially complete stereochemical fidelity. In contrast to our previous results, regioisomerically pure *α*‐substituted lactols (straightforwardly obtained by α‐alkylation of δ‐valerolactone) also participated in the desired reaction, affording C5‐substituted products **2 ah**–**2 al** with complete regiocontrol. Again, the functional group compatibility here is notable, with alkenes and alkynes well tolerated in the process. In a similar manner, a γ‐substituted lactol was smoothly converted to C3‐substituted cyclohexene **2 am** without any isomerization.

These results demonstrated that by employing a combination of HWE olefination followed by aluminium mediated isomerization, it was possible to selectively functionalize three of the five positions around the acyl‐cyclohexene core (C3, C4 and C5). However, it became apparent that few methods are available in the literature for the synthesis of the δ‐substituted lactols, that would be required to prepare C2 substituted cyclohexenes. This problem serves to illustrate a major advantage of our method—which is that *many* other methods to synthesize the tetrahydropyran (or hydroxyenone) intermediates are available. For example, we envisaged that the tetrahydropyrans required to produce C2‐substituted cyclohexenes could instead be rapidly assembled by a cross‐metathesis reaction between hydroxyalkenes and vinyl ketones (followed by cyclization).^[11]^ Treatment with Al_2_O_3_ would then affect ring opening and transformation to the desired cyclohexenes (Scheme 3). Pleasingly, this approach also proved successful and a variety of C2‐ substituted cyclohexenes (**2 an**–**2 ap**) were prepared in high yields including examples bearing aliphatic and aromatic substituents. The metathesis method was not limited to C2‐substitution and other cyclohexenes such as **2 aq**, **2 ar** and **2 as** could also be readily accessed using this approach. Finally, we set out to access the last remaining substitution pattern of acyl cyclohexenes bearing a C6‐substituent. Here, we found that the requisite tetrahydropyrans could be straightforwardly prepared by addition of a cuprate to hydroxyalkynes (easily made in one step, see Supporting Information).^[12]^ Gratifyingly, upon exposure to Al(O^*t*^Bu)_3_, these tetrahydropyrans smoothly underwent rearrangement and condensation to afford the corresponding C6‐substituted cyclohexenes **2 at** and **2 au** in excellent yields, and with complete regiocontrol. These results highlight the remarkable generality of this method, which can be employed to install a substituent at any desired position around the acyl‐cyclohexene ring with complete regiocontrol.

We also wanted to investigate whether it was possible to streamline the process, to enable the pyran formation and aluminium mediated rearrangement to be carried out in a single operation. Interestingly, we discovered that addition of Al_2_O_3_ or Al(O^*t*^Bu)_3_ after the HWE reaction gave no conversion to the desired cyclohexene. This observation was rationalized on the basis that the aluminium mediated 1,5‐hydride shift was inhibited by sodium diethylphosphate, the byproduct of the HWE reaction, which was confirmed by control experiments with externally added NaO_2_P(OEt)_2_. Pleasingly, we found that following the HWE reaction, this byproduct could be removed by aqueous workup followed by a filtration through a short SiO_2_ plug. The crude tetrahydropyrans thus obtained could be directly treated with Al(O^*t*^Bu)_3_ and transformed to the corresponding cyclohexenes in good to excellent yields and with no erosion in selectivity (see yield for two steps for **2 d**, **2 t** and **2 aj** in Scheme 3). For tetrahydropyrans synthesised via other methods, a one‐pot approach could be deployed more straightforwardly. For example, following cross metathesis, direct addition of Al(O^*t*^Bu)_3_ to the reaction enabled the synthesis of cyclohexene **2 an** in 58 % yield (Scheme 3). Pleasingly, we also found that tetrahydropyrans could be prepared via a Mukaiyama‐type addition of silyl enol ethers to lactol acetates, and transformed to the corresponding cyclohexenes without purification (see Supporting Information).^[13]^

### Mechanistic Studies

**Scheme 4:**
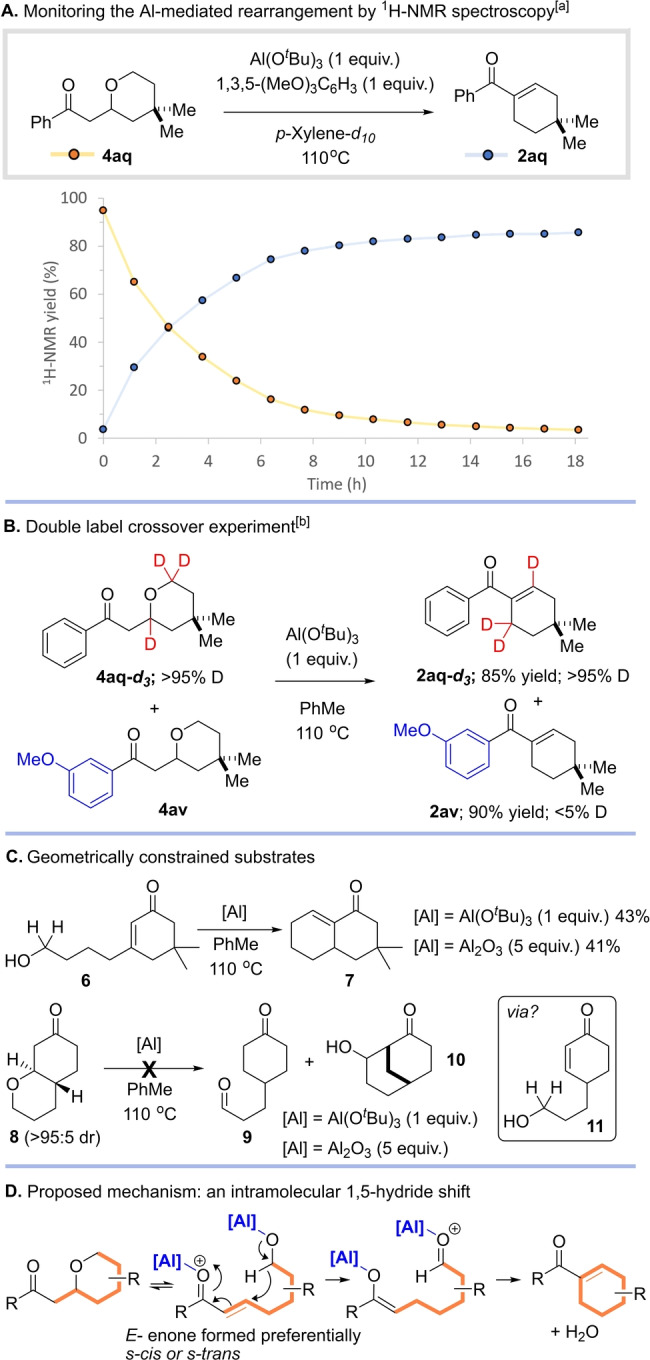
Experiments to probe the mechanism of the aluminium mediated rearrangement. [a] The reaction was carried out in an NMR tube and analyzed by variable temperature ^1^H NMR spectroscopy (500 MHz, 110 °C) with tetrahydropyran **4 aq** (0.1 mmol, 1 eq.), Al(O^*t*^Bu)_3_ (1 eq.), 1,3,5‐trimethoxybenzene (1 eq.), *p*‐Xylene‐d_10_, 16 h. [b] Reaction conditions: **4 aq‐*d***_***3***_ (0.5 eq.), **4 av** (0.5 eq.), Al(O^*t*^Bu)_3_ (1 eq.), PhMe, 110 °C, 16 h. The extent of deuterium labelling was determined by separating enone products **2 aq‐*d***_***3***_ and **2 av** by column chromatography followed by analysis employing a combination of ^1^H, ^2^H, and ^13^C NMR spectroscopy (See Supporting Information for details). For all compounds, the percentage of D incorporation indicated refers to the amount of D present at each site.

Having developed an efficient strategy for cyclohexene synthesis, we set out to understand the unique ability of aluminium‐derived Lewis acids to promote this chemistry. We began by monitoring the course of the Al(O^*t*^Bu)_3_ mediated rearrangement of tetrahydropyran **4 aq** by ^1^H NMR spectroscopy (Scheme 4A). The reaction was performed in an NMR tube in *p*‐xylene‐*d_10_* along with 1 eq. of 1,3,5‐trimethoxybenzene as an internal standard, and heated to 110 °C, with ^1^H NMR spectra recorded over the course of 18 h. We observed a steady consumption of tetrahydropyran starting material **4 aq**, accompanied by formation of cyclohexene **2 aq**, with the reaction reaching completion after approximately 14 hours. No intermediates were observed at any time, which implies that the final intramolecular aldol process is rapid, preventing accumulation of the intermediate ketoaldehyde. To shed further light on the nature of the hydride transfer step itself, we synthesized deuterated tetrahydropyran **4 aq‐*d***_***3***_ and subjected it to a double label crossover experiment with 3‐methoxyphenyl‐substituted tetrahydropyran **4 av** under our optimized conditions (Scheme 4B). Following this reaction cyclohexenes **2 aq‐*d***_***3***_ and **2 av** were isolated in yields of 85 % and 90 % respectively. ^1^H NMR analysis of **2 aq‐*d***_***3***_ and **2 av** revealed that no crossover of the deuterium label had taken place (>95 % D and <5 % D respectively).^[14]^ This result strongly supports our proposal that the hydride shift occurs via an intramolecular process rather than an alternative intermolecular pathway.

We recognized that the hydride shift reaction could conceivably proceed via either an *E*‐ or Z*‐*configured enone (alkene geometry is interconvertible via the ring closed THP compound, see **4 d** and **5 d** in Table 1). Moreover, each enone isomer may react in the *s‐cis* or *s‐trans* conformation. Therefore, we prepared two constrained model systems that were designed to probe the viability of the alkene isomers in the hydride shift reaction. Compound **6** was prepared (see Supporting Information) and subjected to cyclization conditions involving both Al(O^*t*^Bu)_3_ and Al_2_O_3_ (Scheme 4C); in both cases the desired product **7** was isolated in reasonable yield. Compound **6** possesses a geometry whereby the hydroxy alkyl side‐chain and carbonyl group are fixed in a *trans* arrangement and are also held as the *s‐trans* conformer and this result shows the viability of reaction via this arrangement. Another model compound **8** was also prepared (see Scheme 4C and Supporting Information) and subjected to the same two reaction conditions. However, in both cases none of the products **9**/**10** that might arise from a hydride shift process were observed and the reactions returned starting material, together with the formation of complex mixtures. Note that in this case the initial product of a hydride shift and an aldol reaction (**10**) cannot undergo dehydration because the product would be too strained‐ and so we predicted to find this intermediate or the aldehyde precursor **9** as the products. In order to react, compound **8** is required to first ring open to intermediate **11**. Once opened, compound **11** has the hydroxy alkyl side‐chain and carbonyl *cis* disposed and the enone fixed in the *s‐trans* conformer. We interpret the failure of compound **8** to undergo hydride shift as anecdotal evidence for the unfeasibility of reaction via a *Z‐s‐trans* configured enone. Of course, it is also possible that compound **8** fails to react because of its inability to undergo tetrahydropyran ring opening; note that experiments to trap putative intermediate **11** in a Diels Alder cycloaddition failed.

Taken together, these experiments implicate a pathway involving ring opening of the tetrahydropyran followed by a 1,5‐hydride shift mediated by Al (Scheme 4D). Final unimolecular aldol condensation and loss of water would deliver the cyclohexene.[^15^, ^16^] However, some questions still remained, namely: (i) what is the role of aluminium in the key rearrangement step—is it associated with the alkoxide, the carbonyl, or both; (ii) if a double‐activation pathway is operative, as is commonly observed in the Meerwein‐Pondorff‐Verley reaction, what is the aggregation state of aluminium during the reaction and can a single aluminium atom coordinate both the alcohol (hydride donor) and carbonyl (hydride acceptor) simultaneously; and (iii) which isomer of the key enone intermediate undergoes the key 1,5‐hydride shift (our experimental results appear to discount rearrangement via a *Z‐s‐trans* configured enone, but cannot distinguish between pathways involving *E*‐*s*‐*cis* and *E*‐*s*‐*trans* and *Z‐s‐cis* intermediates.

**Scheme 5:**
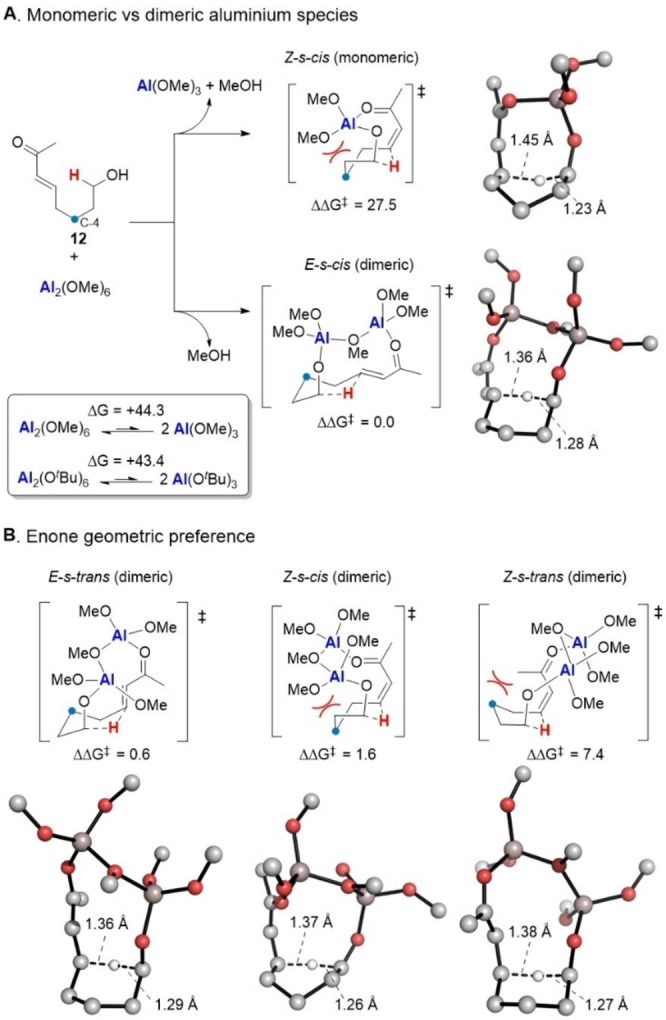
Computational modeling of the key hydride transfer transition state, comparing (A) the likelihood of Al(OMe)_3_ and Al_2_(OMe)_6_ as the active Al species, and (B) the conformational and geometric preferences for the dimeric TS. Calculations were carried out at the CPCM(PhMe)‐ωB97X‐D3/ma‐def2‐TZVPP//PBE0‐D3BJ/def2‐SVP level,^[17]^ with free energy corrections applied for a 1 M standard state and 383 K using a qRRHO approximation.^[18]^ All free energies are reported in kcal mol^−1^, and distances in Å. Non‐reactive hydrogen atoms are hidden for clarity.

To explore these factors further and probe the role of the key aluminium species on the hydride migration, we turned to computational modelling to uncover the coordination mode and aggregation state likely to be responsible for the observed reaction (see Supporting Information for computational details). For a model system comprising Al_2_(OMe)_6_ and (*E*)‐8‐hydroxyoct‐3‐en‐2‐one (**12**), the lowest energy reaction pathways for both monomeric and dimeric Al species were calculated (Scheme 5A). Our calculations reveal a stark preference for the dimeric binding mode, where the lowest energy *E*‐*s*‐*cis* transition state (TS) for hydride transfer is 27.5 kcal mol^−1^ lower than any monomeric TS (*Z*‐*s*‐*cis*). This preference stems primarily from the enthalpically favourable Al−O interactions in the dimeric Al_2_(OMe)_6_ complex, which dismantling to monomers is endergonic (ΔG=44.3 kcal mol^−1^, Scheme 5A inlay). While the monomeric species is entropically favourable over the dimeric form, this effect (−TΔS=−15.8 kcal mol^−1^ at 383 K) is unable to offset the enthalpic contribution discussed above. When methoxide is replaced by *tert*‐butoxide ligands in the Al complex, similar energetics are obtained (ΔG=43.4 kcal mol^−1^, see Scheme 5A inlay and Supporting Information), suggesting that methoxide is a good model for the system tested experimentally.

Interestingly, the monomeric TS is less destabilized than would be expected considering only Al_2_(OMe)_6_ decomposition. This predicts that the monomeric Al(OMe)_3_ would facilitate hydride transfer more efficiently than the dimeric complex (see Supporting Information); however, this preference is overridden by unfavourable monomer formation. We also note that the *Z*‐*s*‐*cis* monomeric TS is slightly destabilized by the requirement to form a boat‐like 6‐membered TS, which results from the minimization of steric clashes between one alkoxy ligand and the substrate C‐4 position (with respect to the starting pyran, shown with a blue dot in Scheme 5A)—an effect that will be exacerbated with *tert*‐butoxide ligands. In this case, the corresponding monomeric *E*‐*s*‐*cis* TS, which is 2.3 kcal mol^−1^ higher in free energy than *Z*‐*s*‐*trans* (see Supporting Information), may then become the lowest energy geometry; however, the monomeric pathway will still be disfavoured relative to the dimeric one.

For the dimeric pathway, the TS featuring an *E*‐*s‐cis* enone is predicted to be only slightly lower in energy than with an *E*‐*s‐trans* enone (ΔΔG^≠^=0.6 kcal mol^−1^, Scheme 5B). This is likely due to the flexibility of the 10‐membered ring in the [7.3.1] bicyclic TS; therefore both pathways will be traversed in the reaction. The *Z*‐enone TSs are predicted to be higher in energy than those corresponding to the *E*‐enones (ΔΔG^≠^=1.6 and 7.4 kcal mol^−1^, for *s*‐*cis* and *s*‐*trans* conformers relative to the *E*‐*s‐cis* TS, respectively). A closer examination of the differences between the *E* and *Z* TS geometries reveals that, as observed in the monomeric *Z*‐*s*‐*cis* TS, the dimeric *Z*‐enone TSs suffer from a transannular steric penalty through the placement of either an alkoxy ligand (*Z*‐*s*‐*cis*) or ketone alkyl substituent (*Z*‐*s*‐*trans*) inside the concave face of the bicyclic TS. These substituents will therefore be proximal to the substrate C‐4 position, in the *Z*‐*s*‐*cis* case forcing a boat‐like 6‐membered ring in the [1,5]‐hydride transfer step. As noted above, this steric effect will likely be magnified when *tert*‐butoxide ligands are considered. This result is in line with the successful reaction of compound **6** (via *E*‐*s*‐*trans*) and the lack of product observed from the reaction of substrate **8**. The failure of **8** to react can be predicted by the high calculated energy of the dimeric *Z‐s‐trans* TS and may provide additional evidence that the monomeric Al species is also not active in this reaction since, in this case, the lowest energy monomeric *Z*‐*s*‐*cis* TS is inaccessible to **8** (via **11**) because of geometric constraints.

In summary, these computational models predict a dimeric Al complex to be the active species in the reaction, in which TSs containing both *E*‐*s*‐*cis* and *E*‐*s*‐*trans* enones are readily accessible to the substrate.

## Conclusion

Substituted cyclohexanes are of paramount importance in naturally occurring and medicinally relevant molecules, but existing methods for their synthesis have been restricted by poor regiocontrol, poor functional group tolerance and a reliance upon expensive transition metal catalysts. We have developed a new method for cyclohexene synthesis, which employs simple and inexpensive aluminium reagents to promote a 1,5‐hydride shift‐aldol cascade to directly afford cyclohexenes. We have shown that the hydroxyenone/tetrahydropyran precursors can be straightforwardly assembled by a wide variety of chemistry (HWE olefination, cross‐metathesis, cuprate addition to alkynes and Mukaiyama additions were all explored, but *many* other routes are possible) and transformed to the corresponding cyclohexenes. The generality and functional group tolerance of the method is particularly noteworthy—cyclohexenes substituted at every possible position can be accessed with complete regiocontrol, including aromatic and aliphatic products substituted with esters, nitriles, nitro groups, amines, pyridines, halogens, boronic esters and carbamates. The mechanism of the process was interrogated employing both experimental and computational studies, which revealed that the key step of the process occurs via a 1,5‐hydride shift promoted by an aluminium dimer, which served a key role in activating both the alkoxide nucleophile and the electrophilic carbonyl group. We anticipate that this chemistry will find wide‐spread application in the synthesis of valuable acyl‐cyclohexenes, as well as spurring the development of other new redox transfer processes using these aluminium reagents.

## Conflict of interest

The authors declare no conflict of interest.

1

## Supporting information

As a service to our authors and readers, this journal provides supporting information supplied by the authors. Such materials are peer reviewed and may be re‐organized for online delivery, but are not copy‐edited or typeset. Technical support issues arising from supporting information (other than missing files) should be addressed to the authors.

Supporting Information

## Data Availability

The data that support the findings of this study are available in the supplementary material of this article.
