# Supplementary material for: Redox Reorganization: Aluminium Promoted 1,5‐Hydride Shifts Allow the Controlled Synthesis of Multisubstituted Cyclohexenes
Source: Angew Chem Int Ed Engl. 2023 Jul 21;62(36):e202307424. doi: 10.1002/anie.202307424 (PMC10953022; doi:10.1002/anie.202307424)
Supplement: Supplementary file 1 — Supporting Information [file ANIE-62-0-s001.pdf]

Angewandte Chemie
Eine Zeitschrift der Gesellschaft Deutscher Chemiker

Supporting Information

Redox Reorganization: Aluminium Promoted 1,5-Hydride Shifts Allow the Controlled Synthesis of Multisubstituted Cyclohexenes

L. B. Smith, R. J. Armstrong, J. Hou, E. Smith, M. Sze, A. J. Sterling, A. Smith, F. Duarte, T. J. Donohoe**

Supporting Information for

Redox Reorganization: Aluminium Promoted 1,5-Hydride Shifts Allow the Controlled Synthesis of Multisubstituted Cyclohexenes

Lewis B. Smith, Roly J. Armstrong, Jingyan Hou, Ming Sze, Alistair J. Sterling, Alex Smith, Fernanda Duarte and Timothy J. Donohoe

Contents

**1.**	**General Information**	**S1**
**2.**	**General Procedures**	**S3**
**3.**	**Experimental Procedures**	**S5**
3.1.	*Synthesis of Starting Materials*	**S5**
	3.1.1 Synthesis of Lactols	**S5**
	3.1.2 Synthesis of HWE Reagents	**S25**
	3.1.3 Synthesis of Cross Metathesis Precursors	**S38**
	3.1.4 Synthesis of Cuprate Addition Precursors	**S39**
3.2.	*Synthesis of Cyclohexenes*	**S40**
	3.2.1 Synthesis of Tetrahydropyrans	**S40**
	3.1.2 Synthesis of Cyclohexenes	**S80**
**4.**	**Mechanistic Experiments**	**S116**
4.1.	*Monitoring the Reaction by ^{1}H-NMR*	**S116**
4.2.	*Double Label Crossover Experiment*	**S118**
**5.**	**Computational Studies**	**S122**
**6.**	**References**	**S127**
**7.**	**NMR Spectra**	**S130**

1. General Information

Procedures using oxygen and/or moisture-sensitive materials were performed with anhydrous solvents (*vide infra*) under an atmosphere of anhydrous argon/nitrogen in flame-dried flasks, using standard Schlenk techniques. Analytical thin-layer chromatography was performed on Merck Kieselgel 60 F254 0.25 mm pre-coated aluminium plates and visualised using a combination of UV light (254 nm) and aqueous ceric ammonium molybdate (CAM), aqueous basic potassium permanganate stains or vanillin solution. The removal of solvents *in vacuo* was achieved using a Büchi rotary evaporator with an oil pump (0.1 mmHg) or diaphragm pump (15 mmHg) at bath temperatures up to 50˚C. Flash column chromatography (FCC) was carried out using Apollo Scientific silica gel 60 (0.040 –0.063 nm) and Merck 60 Å silica gel (0.015-0.040). Pressure was applied at the column head via a flow of nitrogen with the solvent system used in parentheses. Reactions at 0 °C were performed using an ice-water bath, which was covered with cotton and foil if overnight stirring was required. Other temperatures were obtained using a Julabo FT902 immersion cooler or the heating plate of the stirrer with an oil bath. Unless stated otherwise, solution NMR spectra were recorded at room temperature; ^{1}H and ^{13}C NMR experiments were carried out using Bruker NMR spectrometers (400, 500 or 700 MHz) in the deuterated solvent stated, using the residual non-deuterated solvent signal as an internal reference. Chemical shifts (δ) are given in ppm and coupling constants (*J*) are quoted to the nearest 0.5 hertz (Hz). ^{2}H NMR experiments were carried out using Bruker AVIII HD 500 (77 or 92 MHz) in protonated solvent with a single drop of the corresponding deuterated solvent as an internal reference. Resonances are described as s (singlet), d (doublet), t (triplet), q (quartet), p (pentet), h (hextet), hept (heptet) and m (multiplet). ^{1}H and ^{13}C NMR peaks for diastereomers were assigned major or minor. If major or minor diastereomers cannot be distinguished, the peaks were assigned with a '′' (e.g H_1 and $H_{1'}$, C_1 anc $C_{1'}$). Peaks for diastereotopic environments were assigned with a subscript 'a' and 'b' (e.g H_a and H_b), or subscript 'ax.' (axial) and 'eq.' (equatorial) where applicable. Assignments were made with the assistance of gCOSY, gHSQC, gHMBC or NOESY NMR spectra. All chiral compounds are racemic unless marked with an absolute stereochemistry descriptor.

Chiral HPLC separations were achieved using an Agilent 1260 Infinity series normal phase HPLC unit and HP Chemstation software. Chiralpak® columns (250 × 4.6 mm), fitted with

matching Chiralpak® Guard Cartridges (10 × 4 mm), were used as specified in the text. Solvents used were of HPLC grade (Fisher Scientific, Sigma Aldrich or Rathburn); all eluent systems were isocratic. Reverse phase HPLC separations were achieved using an Dionex Ultimate 3000 HPLC unit and Chromeleon software. An agilent Poroshell 120 EC-C18 4μm column (150 × 4.6 mm), fitted with matching Agilent Guard Cartridges (10 × 4 mm), was used as specified in the text. Solvents used were of HPLC grade (Fisher Scientific, Sigma Aldrich or Rathburn); all eluent systems were isocratic. Low-resolution mass spectra were recorded using a Walters LCT premier XE. High-resolution mass spectra (EI and ESI) were recorded using a Bruker MicroTOF spectrometer by the internal service at the University of Oxford. Infrared measurements (neat, thin film) were carried out using a Bruker Tensor 27 FT-IR with internal calibration in the range 600-4000 cm^{-1}. Optical rotations were recorded on a Perkin-Elmer 241 polarimeter at 25°C in a 10 cm cell in the stated solvent; $[\alpha]_D$ values are given in $10^{-1}deg.cm^2g^{-1}$ (concentration c given as g/100mL). Melting points were obtained using a Leica Galen III heated-stage microscope and are uncorrected.

2. General Procedures

General Procedure 1: Synthesis of Lactols by DIBAL-Reduction

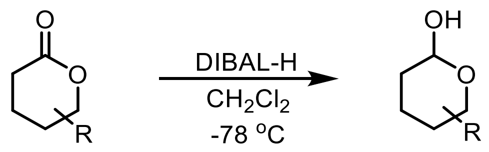

A flame dried RBF under N_2 was charged with lactone (1 equiv.), dry CH_2Cl_2 (0.5 M), and the resulting stirred solution was cooled to –78 °C. DIBAL-H in hexanes (1 M, 1.4 equiv.) was added dropwise and the resulting solution was stirred at –78 °C for 2 hours. Aq. Rochelle salt (2 mL/mmol lactone) was added dropwise, and the resulting mixture was warmed to RT and stirred vigorously. The aqueous phase was then extracted three times with CH_2Cl_2. The combined organic phases were then dried over $MgSO_4$, filtered and concentrated *in vacuo*. For details of purification, see experimental procedures.

General Procedure 2: HWE reagent synthesis by Claisen Condensation

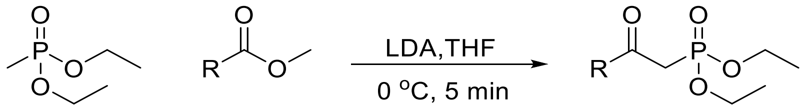

A flame dried RBF under an argon atmosphere was charged with diisopropylamine (2.25 equiv.) and dry THF (0.2 M) and the resulting stirred solution was cooled to 0 °C. *n*-BuLi in hexanes (2.5 M, 2.2 equiv.) was added dropwise, and the resulting solution was stirred for 30 minutes at 0 °C. A separate flame dried RBF under an argon atmosphere was charged with methyl ester (1 equiv.), diethyl methylphosphonate (1.1 equiv.) and dry THF (0.2 M) and then cooled to 0 °C. The LDA solution was then added dropwise to this flask *via* canula and the resulting mixture was stirred for a further 5 minutes at 0 °C. After this time, the reaction mixture was diluted with 3 M aq. HCl, and then warmed to RT. The aqueous phase was extracted three times with EtOAc. The combined organic phases were dried over $MgSO_4$, filtered and concentrated *in vacuo*. For details of purification, see experimental procedures.

General Procedure 3: Tetrahydropyran Synthesis by HWE Reaction

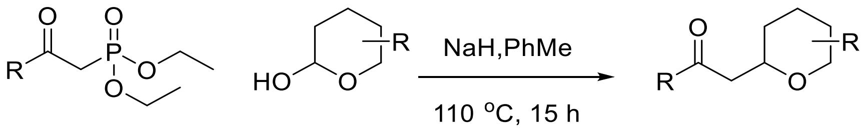

A 10 mL flame dried RBF fitted with a reflux condenser was charged with HWE reagent (1.4 equiv., 1.4 mmol) and the flask was evacuated and backfilled with N_2 three times. Dry toluene (5 mL) was added, and 60% wt. NaH (48 mg, 1.2 eq., 1.2 mmol) was added in one portion [care: vigorous release of H_2]. The resulting suspension was stirred at RT for 30 min, by which time a homogeneous solution had formed. Lactol (1.0 equiv., 1 mmol) was added and the reaction was heated at 110 °C for 15 h. The reaction was then cooled to RT, diluted with Et_2O (50 mL), and filtered through a plug of silica gel washing with Et_2O. For details of purification, see experimental procedures.

General Procedure 4: Cyclohexene Synthesis by Aluminium Mediated [1,5]-Hydride Shift

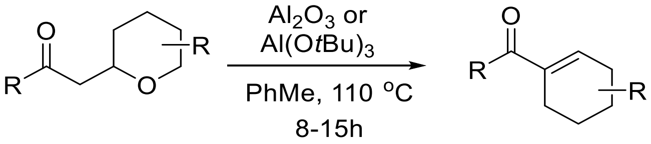

A 2–5 mL flame dried Biotage® microwave vial was charged with tetrahydropyran (0.3 mmol, 1 equiv.), and basic alumina (Brockmann I, 5 equiv. 150 mg) or aluminium *tert*-butoxide (74 mg, 1 equiv.) and dry toluene (1.5 mL, 0.2 M). The headspace was briefly purged with N_2 and the reaction vessel was sealed with a microwave vial cap (containing a Reseal™ septum) and heated at 110 °C for 16 h. The reaction was then cooled to RT, diluted with Et_2O (50 mL), filtered through a plug of silica gel, washing with Et_2O and concentrated *in vacuo*. For details of purification, see experimental procedures.

3. Experimental Procedures

3.1 Synthesis of Starting Materials

3.1.1. Synthesis of Lactols

5-Methyltetrahydro-*2H*-pyran-2-ol, 1a

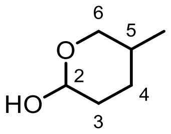

According to a modified literature procedure,[1] a stirred solution of 2-methylhex-5-en-1-ol[2] (5.00 g, 39.4 mmol) in CH_2Cl_2 (500 mL) was cooled to –78 °C and a stream of ozone was bubbled through the solution. As soon a pale blue colour developed, ozone bubbling was halted and a stream of N_2 was bubbled through the solution until the blue colour disappeared (~10 min). After this time, dimethylsulfide (29 mL, 390 mmol) was added slowly and the resulting mixture was stirred at –78 °C and left in the CO_2/acetone bath overnight to slowly come to RT. The resulting solution was concentrated *in vacuo*. The title compound was afforded after purification by FCC (50% EtOAc/pentane) as a colourless oil (2.48 g, 21.3 mmol, 54% yield, 54:46 mixture of anomers by ^{1}H NMR).

^{1}H NMR ($CDCl_3$, 400 MHz) δ 5.16 (q, *J* = 3.0 Hz, 1H, minor H_2), 4.67 (ddd, *J* = 8.5, 6.0, 2.0 Hz, 1H, major H_2), 3.89 (ddd, *J* = 11.5, 4.5, 2.5 Hz, 1H, major H_{6a}), 3.84 (1H, d, *J* = 6.0 Hz, major OH), 3.59 (t, *J* = 10.5 Hz, 1H, minor H_{6a}), 3.49 (ddd, *J* = 11.0, 4.5, 1.5 Hz, 1H, minor H_{6b}), 3.33 (dd, *J* = 3.5, 1.5 Hz, 1H, minor OH), 3.11 (dd, *J* = 11.5, 10.5 Hz, 1H, major H_{6b}), 1.92 – 1.35 (m, 9H, major H_3 x 2, minor H_3 x 2, major H_{4a}, minor H_4 x 2, major H_5, minor H_5), 1.26 – 1.11 (m, 1H, major H_{4b}), 0.84 (d, *J* = 6.5 Hz, 3H, minor CH_3), 0.80 (d, *J* = 6.5 Hz, 3H, major CH_3).

^{13}C NMR (101 MHz, $CDCl_3$) δ 96.3, 91.6, 72.2, 66.5, 32.5, 30.6, 30.3, 30.0, 26.1, 17.5, 16.6. *N.B.* the peak at 30.0 corresponds to two overlapping signals.

HRMS (ESI+): Found $[M+Na]^+$ = 139.0731; $C_6H_{12}O_2Na$ requires 139.0730, Δ 1.01 ppm

IR (film) ν_{max}/cm^{-1} 3376, 2952, 2931, 2873, 1458, 1059, 1033, 867

3-Methyltetrahydro-*2H*-pyran-2-ol, 1b

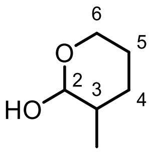

DIBAL-H in hexanes (1M, 2.4 mL, 2.4 mmol, 1.4 eq.), 3-methyltetrahydro-*2H*-pyran-2-one[3] (194 mg, 1.67 mmol, 1 eq.) and CH_2Cl_2 (3 mL) were subjected to **General Procedure 1**. The title compound was afforded after purification by FCC (35 → 50% Et_2O/pentane) as a colourless oil (138 mg, 1.19 mmol, 70% yield, 2:1 mixture of anomers by ^{1}H NMR).

^{1}H NMR (400 MHz, $CDCl_3$) δ 4.98 (t, *J* = 3.5 Hz, 1H, minor H_2), 4.32 (dd, *J* = 7.5, 6.0 Hz, 1H, major H_2), 4.04 – 3.89 (m, 2H, major H_{6a}, minor H_{6a}), 3.59 – 3.45 (m, 2H, major H_{6b}, minor H_{6b}), 3.30 – 3.14 (m, 1H, major OH), 2.87 – 2.70 (m, 1H, minor OH), 1.88 – 1.41 (m, 9H, major H_3, minor H_3, minor H_4 x 2, major H_{4a}, major H_5 x 2, minor H_5 x 2), 1.27 – 1.14 (m, 1H, major H_{4b}), 0.97 (d, *J* = 6.5 Hz, 3H, major CH_3), 0.92 (d, *J* = 7.0 Hz, 3H, minor CH_3).

^{13}C NMR (101 MHz, $CDCl_3$) δ 101.0, 95.2, 66.1, 60.3, 37.3, 34.7, 30.5, 25.9, 25.3, 25.1, 17.0, 16.3.

HRMS (ESI+): Found $[M+Na]^+$ = 139.0730; $C_6H_{12}O_2Na$ requires 139.0730, Δ 0.37 ppm

IR (film) ν_{max}/cm^{-1} 3378, 2932, 2852, 1462, 1154, 1067, 1029, 987, 959

***(*R*)*-4-Phenyltetrahydro-2*H*-pyran-2-one, S1**

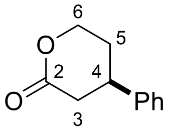

Enantioenriched: According to a modified literature procedure,[4] a 5 mL microwave vial was charged with $Rh(acac)(C_2H_4)_2$ (3.1 mg, 12 μmol, 3 mol%), *(R)*-BINAP (9.0 mg, 14 μmol, 4 mol%), phenyl boronic acid (244 mg, 2.00 mmol, 5 eq.), and dry 1,4-dioxane (2 mL, 0.2 M). The vial was then flushed with argon, followed by the addition of water (0.2 mL, 0.02 M) and 5,6-dihydro-2*H*-pyran-2-one (39 mg, 0.40 mmol, 1 eq.). The resulting mixture was heated at 100 ^{o}C for 3 h. After removal of the solvent, the residue was diluted with sat. aq. sodium bicarbonate (10 mL), extracted with EtOAc (3 x 10 mL), dried over $MgSO_4$ and concentrated *in vacuo*. The title compound was afforded after purification by FCC (20% EtOAc/pentane) as a colourless oil (69 mg, 0.39 mmol, 98% yield, 99:1 e.r.). The spectral data matched that previously reported in the literature.[5]

Racemic: The racemic compound was prepared following the same procedure using racemic BINAP. The title compound was isolated as a colourless oil (42 mg, 0.24 mmol, 60% yield).

^{1}H NMR (400 MHz, $CDCl_3$) δ 7.42 – 7.35 (m, 2H, ArCH x 2), 7.33 – 7.27 (m, 1H, ArCH), 7.27 – 7.21 (m, 2H, ArCH x 2), 4.53 (ddd, *J* = 11.5, 5.0, 4.0 Hz, 1H, H_{6a}), 4.42 (ddd, *J* = 11.5, 10.5, 4.0 Hz, 1H, H_{6b}), 3.27 (tdd, *J* = 10.5, 6.0, 4.5 Hz, 1H, H_4), 2.95 (ddd, *J* = 17.5, 6.0, 1.5 Hz, 1H, H_{3a}), 2.66 (dd, *J* = 17.5, 10.5 Hz, 1H, H_{3b}), 2.21 (dqd, *J* = 14.0, 4.0, 1.5 Hz, 1H, H_{5a}), 2.07 (dtd, *J* = 14.0, 10.5, 5.0 Hz, 1H, H_{5b}).

^{13}C NMR (101 MHz, $CDCl_3$) δ 170.8, 142.9, 129.1, 127.4, 126.6, 68.8, 37.6, 37.6, 30.5.

$\alpha_{25}{}^{D}$= –5.0 (c = 1.0, $CHCl_3$). Lit. $\alpha_{20}{}^{D}$ = –4.1 (c = 1.0, $CHCl_3$).[5]

HPLC: Enantiomeric excess was determined by HPLC with a Chiralpak® IG column (90:10 hexane:IPA, 1.0 mL min-1, 210 nm, room temperature); t_r (major) = 27.6 min, t_r (minor) = 29.2 min.

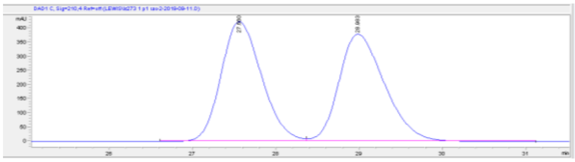

#	Time	Type	Area	Height	Width	Area%	Symmetry
1	27.56	BV	14329.4	423.6	0.5246	49.888	0.792
2	28.983	VB	14394	379.2	0.5868	50.112	0.678

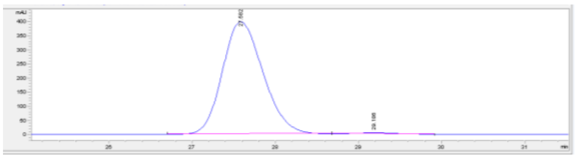

#	Time	Type	Area	Height	Width	Area%	Symmetry
1	27.582	BB	13560.5	398.2	0.5293	99.006	0.791
2	29.186	BB	136.1	4.2	0.4868	0.994	0.856

(4*R*)-4-Phenyltetrahydro-2*H*-pyran-2-ol, 1c

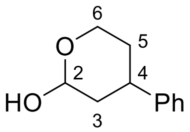

DIBAL-H in hexanes (1M, 5.6 mL, 5.6 mmol, 1.4 eq.), lactone **S1** (700 mg, 3.97 mmol, 1 eq.), and CH_2Cl_2 (8 mL) were subjected to **General Procedure 1**. The title compound was afforded after purification by FCC (40% Et_2O/pentane) as a white solid (706 mg, 3.96 mmol, 99% yield, 66:34 mixture of anomers by ^{1}H NMR). Analysis by chiral HPLC was not possible, owing to extensive peak broadening, presumably due to interconversion of anomers on the HPLC timescale.

^{1}H NMR (400 MHz, $CDCl_3$) δ 7.48 – 7.15 (m, 10H minor ArCH x 5 and major ArCH x 5), 5.54 – 5.39 (m, 1H, major H_2), 4.86 (ddd, *J* = 9.5, 6.5, 2.0 Hz, 1H, minor H_2), 4.30 – 4.10 (m, 2H, major H_{6ax} and minor H_{6eq}), 3.79 (ddd, *J* = 11.5, 4.5, 2.0 Hz, 1H, major H_{6eq}), 3.74 – 3.62 (m, 1H, minor H_{6ax}), 3.41 (d, *J* = 6.5 Hz, 1H, minor OH), 3.25 (tt, *J* = 12.0, 4.0 Hz, 1H, major H_4), 2.95 – 2.78 (m, 2H, minor H_4, major OH), 2.24 – 2.12 (m, 1H, minor H_{3eq}), 2.02 (ddt, *J* = 13.5, 3.5, 1.5 Hz,

1H, major H_{3eq}), 1.96 – 1.73 (m, 5H, major H_{3ax}, major H_{5eq}, major H_{5ax}, minor H_{5eq}, minor H_{5ax}), 1.62 (td, *J* = 12.5, 9.5 Hz, 1H, minor H_{3ax}).

^{13}C NMR (101 MHz, $CDCl_3$) δ 145.7, 144.6, 128.8, 128.7, 126.9, 126.9, 126.7, 126.5, 96.5, 91.9, 66.0, 66.0, 40.7, 40.5, 37.7, 34.7, 33.2, 32.9.

HRMS (ESI+): Found $[M+Na]^+$ = 201.0888 ; $C_{11}H_{14}O_2Na$ requires 201.0886, Δ 1.15 ppm

IR (film) ν_{max}/cm^{-1} 3338, 2954, 2877, 1386, 1252, 1140, 874, 760, 701

m.p.: 128-129 ^{o}C

α_{25}^{D}= −42.4 (c = 1.0, $CHCl_3$).

4-Methyltetrahydro-*2H*-pyran-2-ol, 1d

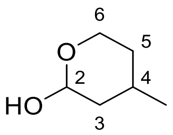

DIBAL-H in hexanes (1M, 17.2 mL, 17.2 mmol, 1.4 eq.), 4-methyltetrahydro-2*H*-pyran-2-one[6] (1.40 g, 13.1 mmol, 1 eq.), and CH_2Cl_2 (25 mL) were subjected to **General Procedure 1**. The title compound was afforded after purification by FCC (Et_2O) as a colourless oil (1.46 g, 12.8 mmol, 97% yield, 55:45 mixture of anomers by ^{1}H NMR). The spectral data matched that reported in the literature.[7]

^{1}H NMR (400 MHz, $CDCl_3$) δ 5.29-5.25 (m, 1H, minor H_2), 4.65 (ddd, *J* = 9.5, 6.0, 2.0 Hz, 1H, major H_2), 4.08 – 3.91 (m, 2H, major H_{6a} and minor H_{6a}), 3.62 (ddd, *J* = 11.0, 5.0, 2.0 Hz, 1H, minor H_{6b}), 3.55 – 3.29 (m, 2H, major H_{6b} and OH), 2.96 – 2.72 (m, 1H, OH), 2.07 – 1.93 (m, 1H, minor H_4), 1.89 (ddt, *J* = 13.0, 4.0, 2.0 Hz, 1H, major H_{3a}), 1.84 – 1.61 (m, 2H, major H_4 and minor H_{3a}), 1.61 – 1.53 (m, 1H, minor H_{5a}), 1.52 – 1.44 (m, 1H, major H_{5a}), 1.37 – 1.10 (m, 3H, minor H_{3b}, major H_{5b} and minor H_{5b}), 1.07 – 0.99 (m, 1H, major H_{3b}), 0.97 (d, *J* = 6.5 Hz, 3H major CH_3), 0.91 (d, *J* = 6.5 Hz, 3H, minor CH_3).

^{13}C NMR (101 MHz, $CDCl_3$) δ 96.3, 91.9, 65.8, 59.9, 41.8, 38.8, 34.2, 33.7, 29.5, 23.7, 22.2, 21.9.

4-Isobutyltetrahydro-*2H*-pyran-2-ol, S2

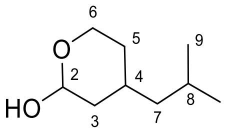

DIBAL-H in hexanes (1M, 9.0 mL, 9.0 mmol, 1.4 eq.), 4-isobutyltetrahydro-2*H*-pyran-2-one[8] (1.00 g, 6.41 mmol, 1 eq.) and CH_2Cl_2 (13 mL) were subjected to **General Procedure 1**. The title compound was afforded after purification by FCC (Et_2O) as a colourless oil (1.01 g, 16.4 mmol, 100% yield, 52:48 mixture of anomers by ^{1}H NMR).

^{1}H NMR (400 MHz, $CDCl_3$) δ 5.34 – 5.20 (m, 1H, major H_2), 4.66 (ddd, *J* = 9.5, 6.5, 2.0 Hz, 1H, minor H_2), 4.06 – 3.92 (m, 2H, major H_{6a} and minor H_{6a}), 3.68 – 3.57 (m, 2H, major $H_{6b,}$ and minor OH), 3.48 (td, *J* = 12.0, 2.5 Hz, 1H, minor H_{6b}), 3.04 (dd, *J* = 3.0, 2.0 Hz, 1H, major OH), 2.04 – 1.93 (m, 1H, major H_4), 1.90 (ddt, *J* = 12.5, 4.0, 2.0 Hz, 1H, minor H_{3a}), 1.79 – 1.55 (m, 5H, major $H_{3a,}$ major H_{5a} and major H_8, minor H_4 and, minor H_8), 1.50 (ddq, *J* = 13.5, 4.0, 2.0 Hz, 1H, minor H_{5a}), 1.31 – 0.93 (m, 8H, major $H_{3b,}$ major H_{5b} and major H_7 x 2, minor $H_{3b,}$ minor H_{5b} and minor H_7 x 2), 0.90 – 0.83 (m, 12H major H_9 x 6 and minor H_9 x 6).

^{13}C NMR (101 MHz, $CDCl_3$) δ 96.4, 91.9, 65.8, 59.9, 46.4, 46.1, 40.2, 37.3, 32.5, 32.1, 32.1, 26.1, 24.5, 24.4, 23.1, 22.9, 22.8, 22.8.

HRMS (ESI+): Found $[M+Na]^+$ = 181.1201 ; $C_9H_{18}O_2Na$ requires 181.1199, Δ 1.10 ppm.

IR (film) ν_{max}/cm^{-1} 3380, 2953, 2870, 1467, 1370, 1070, 898.

8-Oxaspiro[4.5]decan-7-ol, S3

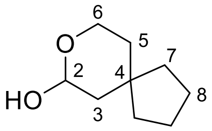

DIBAL-H in hexanes (1M, 8.6 mL, 8.6 mmol, 1.33 eq.), 8-oxaspiro[4.5]decan-7-one[9] (1.00 g, 6.49 mmol, 1 eq.) and CH_2Cl_2 (13 mL) were subjected to **General Procedure 1**. The title compound was afforded after purification by FCC (Et_2O) as a white solid (973 mg, 6.23 mmol, 96% yield). The spectral data matched that previously reported in the literature.[10]

^{1}H NMR (400 MHz, $CDCl_3$) δ 4.83 (ddd, *J* = 8.0, 5.5, 2.5 Hz, 1H, H_2), 3.93 (dt, *J* = 12.0, 4.0 Hz, 1H, H_{6a}), 3.78 (d, *J* = 5.5 Hz, 1H, OH), 3.58 (ddd, *J* = 11.5, 10.5, 2.5 Hz, 1H, H_{6b}), 1.71 – 1.27 (m, 12H, H_3 x 2, H_5 x 2, H_7 x 4 and H_8 x 4).

^{13}C NMR (101 MHz, $CDCl_3$) δ 94.3, 62.6, 44.2, 41.3, 41.1, 36.7, 36.4, 24.5, 23.8.

4-(Dimethyl(phenyl)silyl)tetrahydro-2*H*-pyran-2-one S4

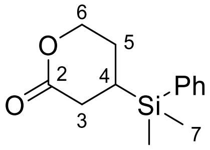

A 5 mL microwave vial was charged with 1,3-bis(diphenylphosphaneyl)propane (8.2 mg, 0.020 mmol, 10 mol%) and bis(1,5-cyclooctadiene)rhodium(I) trifluoromethanesulfonate (4.7 mg, 0.010 mmol, 5.0 mol%) under argon. De-oxegenated dioxane/H_2O (10:1) (0.5 mL, 0.4 M) was added, followed by 5,6-dihydro-2H-pyran-2-one (19.6 mg, 0.200 mmol, 1.00 equiv.), (dimethylphenylsilyl)boronic acid pinacol ester (131 mg, 0.500 mmol, 2.50 equiv.) and triethylamine (20.2 mg, 0.200 mmol, 1.00 equiv.). The resulting mixture was stirred at 50 °C for 16 h. Upon cooling, the solvent was removed *in vacuo*. Purification by FCC (20% EtOAc/Pentane) afforded the product as a colourless oil (33.1 mg, 0.141 mmol, 71% yield). The spectroscopic data matched that previously reported in the literature.[11]

^{1}H NMR (400 MHz, $CDCl_3$) δ 7.54 – 7.37 (m, 2H, ArCH x 2), 7.41 – 7.31 (m, 3H, ArCH x 3), 4.38 – 4.29 (m, 1H, H_{6a}), 4.29 – 4.19 (m, 1H, H_{6b}), 2.57 (ddd, *J* = 17.5, 5.5, 1.5 Hz, 1H, H_{3a}), 2.28 (dd, *J* = 17.5, 12.5 Hz, 1H, H_{3b}), 1.90 – 1.79 (m, 1H, H_{5a}), 1.72 – 1.58 (m, 1H, H_{5b}), 1.47 – 1.31 (m, 1H, H_4), 0.33 (s, 6H, H_7 x 6).

^{13}C NMR (101 MHz, $CDCl_3$) δ 171.7, 135.6, 134.0, 129.1, 128.2, 70.4, 31.0, 23.8, 18.5, -5.5, -5.6.

4-(Dimethyl(phenyl)silyl)tetrahydro-2*H*-pyran-2-ol S5

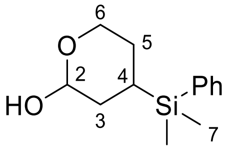

Lactone **S4** (185 mg, 0.791 mmol, 1.00 eq.), DIBAL-H (1M in hexanes, 1.2 mL, 1.2 mmol, 1.5 eq.) and CH_2Cl_2 (1.9 mL) were subjected to **General Procedure 1**. Purification by FCC (20% EtOAc/pentane) afforded the product as a colourless oil (123 mg, 0.520 mmol, 66% yield, 61:39 mixture of anomers by ^{1}H NMR).

^{1}H NMR (400 MHz, $CDCl_3$) δ 7.53 – 7.42 (m, 4H, major ArCH x 2, minor ArCH x 2), 7.42 – 7.30 (m, 6H, major ArCH x 3, minor ArCH x 3), 5.21 (t, *J* = 2.0 Hz, 1H, minor H_2), 4.67 – 4.58 (m, 1H, major H_2), 4.06 – 3.92 (m, 2H, major H_{6a}, minor H_{6a}), 3.59 – 3.42 (m, 2H, major H_{6b}, minor H_{6b}), 2.94 (s, 1H, major OH), 2.50 (s, 1H, minor OH), 1.85 – 1.76 (m, 1H, major H_{3a}), 1.71 – 1.31 (m,

7H, minor H_{3a}, minor H_{3b}, minor H_5 x 2, minor H_4, major H_5 x 2), 1.30 – 1.03 (m, 2H, major H_{3b}, major H_4), 0.34 – 0.21 (m, 12H, major H_7 x 6, H6 minor H_7 x 6).

^{13}C NMR (101 MHz, $CDCl_3$) δ 137.3, 137.1, 134.1, 134.0, 129.3, 129.2, 128.0, 127.9, 97.2, 90.9, 68.2, 60.4, 33.8, 30.8, 26.3, 25.8, 22.1, 15.2, -5.3, -5.4, -5.4, -5.6.

HRMS (ESI+): Found $[M+Na]^+$ = 259.1124; $C_{13}H_{20}O_2SiNa$ requires 259.1125, Δ -0.31 ppm

IR (film) ν_{max}/cm^{-1} : 3374 (broad), 2952, 1252, 1068, 834, 700

4-(Phenylthio)tetrahydro-2*H*-pyran-2-one S6

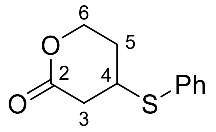

A 100 mL RBF was charged with 5,6-dihydro-2*H*-pyran-2-one (1.47 g, 15.0 mmol, 1.00 eq.) in toluene (30 mL). Thiophenol (1.7 mL, 17 mmol, 1.1 eq.) was added followed by triethylamine (2.1 mL, 15 mmol, 1.0 eq.) and the resulting mixture was stirred at RT for 1.5 h. The reaction mixture was then diluted with Et_2O (50 mL) and washed with 3M aq. HCl (3 x 15 mL), water (2 x 15 mL), 3M aq. NaOH (3 x 15 mL), and water (2 x 15 mL). The organic layer was dried over Na_2SO_4, filtered and concentrated *in vacuo*. Purification by FCC (30% EtOAc/pentane) afforded the product as a colourless oil (2.31 g, 11.1 mmol, 74% yield).

^{1}H NMR (400 MHz, $CDCl_3$) δ 7.48 – 7.41 (m, 2H, ArCH x 2), 7.40 – 7.28 (m, 3H, ArCH x 3), 4.52 (ddd, *J* = 11.5, 6.0, 4.5 Hz, 1H, H_{6a}), 4.27 (ddd, *J* = 11.5, 8.5, 4.0 Hz, 1H, H_{6b}), 3.63 – 3.51 (m, 1H, H_4), 2.97 – 2.87 (m, 1H, H_{3a}), 2.55 (dd, *J* = 17.5, 9.0 Hz, 1H, H_{3b}), 2.27 – 2.15 (m, 1H, H_{5a}), 1.95 – 1.82 (m, 1H, H_{5b}).

^{13}C NMR (101 MHz, $CDCl_3$) δ 169.3, 133.6, 132.1, 129.5, 128.5, 67.3, 39.5, 36.8, 29.2.

HRMS (ESI+): Found $[M+H]^+$ = 209.0632; $C_{11}H_{13}O_2S$ requires 209.0631, Δ 0.60 ppm

IR (film) ν_{max}/cm^{-1} : 2973, 1740, 1255, 1071, 749, 693

4-(Phenylthio)tetrahydro-2*H*-pyran-2-ol S7

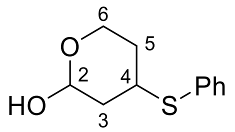

Lactone **S6** (498 mg, 2.39 mmol, 1.00 eq.), DIBAL-H (1M in hexanes, 3.3 mL, 3.3 mmol, 1.4 eq.) and CH_2Cl_2 (5 mL) were subjected to **General Procedure 1**. Purification by FCC (30%

EtOAc/pentane) afforded the product as a colourless oil (445 mg, 2.12 mmol, 89% yield, 66:34 mixture of anomers by ^{1}H NMR).

^{1}H NMR (400 MHz, $CDCl_3$) δ 7.49 – 7.38 (m, 4H, major ArCH x 2, minor ArCH x 2), 7.36 – 7.20 (m, 6H, major ArCH x 3, minor ArCH x 3), 5.33 – 5.26 (m, 1H, major H_2), 4.76 – 4.66 (m, 1H, minor H_2), 4.17 – 3.95 (m, 2H, minor H_{6a}, major H_{6a}), 3.75 – 3.57 (m, 2H, major H_{6b}, major H_4), 3.54 – 3.43 (m, 1H, minor H_{6b}), 3.39 – 3.11 (m, 2H, minor H_4, minor OH), 2.55 (s, 1H, major OH), 2.26 – 2.14 (m, 1H, minor H_{3a}), 2.11 – 2.01 (m, 1H, major H_{3a}), 1.96 – 1.80 (m, 2H, minor H_{5a}, minor H_{5b}), 1.77 – 1.53 (m, 3H, major H_{5a}, major H_{5b}, major H_{3b}), 1.52 – 1.39 (m, 1H, minor H_{3b}).

^{13}C NMR (101 MHz, $CDCl_3$) δ 133.6, 133.4, 133.1, 132.7, 129.1, 129.1, 127.9, 127.4, 95.5, 91.9, 64.2, 59.9, 42.3, 39.5, 38.7, 37.2, 32.6, 32.1.

HRMS (ESI+): Found $[M+Na]^+$ = 233.0608; $C_{11}H_{14}O_2SNa$ requires 233.0607, Δ 0.38 ppm

IR (film) ν_{max}/cm^{-1} : 3376 (broad), 2950, 1118, 1067, 744, 692

(*R*)-4-(4-Fluorophenyl)tetrahydro-2*H*-pyran-2-one, S8

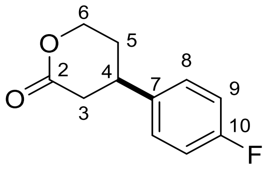

Enantioenriched: A Schlenk tube was charged with $Rh(acac)(C_2H_4)_2$ (79 mg, 0.31 mmol, 3 mol%), *(R)*-BINAP (222 mg, 0.357 mmol, 3.5 mol%), 4-fluorophenylboronic acid (7.13 g, 51.0 mmol, 5 eq.), and the Schlenk tube was evacuated and backfilled with N_2 three times. Dry Ar-sparged 1,4-dioxane (51 mL, 0.2 M) and water (5.1 mL) were added sequentially. The reaction mixture was degassed by evacuating and backfilling with N_2 twice and then 5,6-dihydro-2*H*-pyran-2-one (1.00 g, 10.2 mmol, 1 eq.) was added *via* syringe. The resulting mixture was heated at 100 ^oC for 3 h. After removal of the solvent, the residue was diluted with sat. aq. sodium bicarbonate (50 mL), extracted with EtOAc (3 x 30 mL), dried over $MgSO_4$ and concentrated *in vacuo*. The title compound was afforded after purification by FCC (30% EtOAc/pentane) as a white solid (1.56 g, 8.03 mmol, 79% yield, 98:2 e.r.). The spectral data matched that reported in the literature.[12] The absolute configuration was confirmed to be (*R*)- by comparing the order of peak elution from chiral HLPC to that reported for (*S*)-**S8** in the literature (see below).[13]

Racemic: The racemic compound was prepared following the same procedure using racemic BINAP. The title compound was isolated as a white solid (130 mg, 0.67 mmol, 66% yield).

^{1}H NMR (400 MHz, $CDCl_3$) δ 7.20 – 7.14 (m, 2H, H_8 x 2), 7.07 – 7.01 (m, 2H, H_9 x 2), 4.50 (ddd, *J* = 11.5, 5.0, 4.0 Hz, 1H, H_{6a}), 4.38 (ddd, *J* = 11.5, 10.5, 3.5 Hz, 1H, H_{6b}), 3.23 (tdd, *J* = 10.5, 6.0, 4.5 Hz, 1H, H_4), 2.90 (ddd, *J* = 17.5, 6.0, 1.5 Hz, 1H, H_{3a}), 2.58 (dd, *J* = 17.5, 10.5 Hz, 1H, H_{3b}), 2.16 (ddtd, *J* = 14.0, 4.5, 3.5, 1.5 Hz, 1H, H_{5a}), 2.00 (dtd, *J* = 14.0, 10.5, 5.0 Hz, 1H, H_{5b}).

^{13}C NMR (101 MHz, $CDCl_3$) δ 170.4, 161.9 (d, *J* = 246.0 Hz), 138.5 (d, *J* = 3.5 Hz), 128.0 (d, *J* = 8.0 Hz), 115.8 (d, *J* = 21.5 Hz), 68.5, 37.7, 36.8, 30.4.

^{19}F NMR (377 MHz, $CDCl_3$) δ -115.3.

$\alpha_{25}{}^{D}$= –3.9 (c = 1.0, $CHCl_3$).

HPLC: Enantiomeric excess was determined by HPLC with a Chiralpak® IC column (70:30 hexane:IPA, 0.8 mL min^{-1}, 210 nm, room temperature); t_r (major) = 29.0 min, t_r (minor) = 33.0 min, 98:2 e.r. Lit. data for (*S*)-**S8** the same conditions; t_r (minor) = 28.5 min, t_r (major) = 33.0 min.[13]

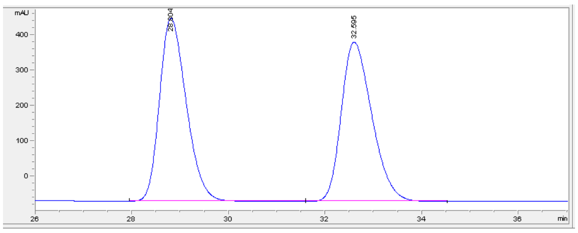

#	Time	Type	Area	Height	Width	Area%	Symmetry
1	28.804	BB	19911.4	518.2	0.5943	50.048	0.708
2	32.595	BB	19873.5	450.9	0.6822	49.952	0.688

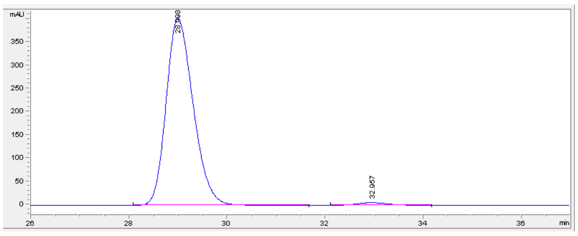

#	Time	Type	Area	Height	Width	Area%	Symmetry
1	28.998	BB	15442.7	402.4	0.5937	98.481	0.739
2	32.957	BB	238.2	5.6	0.5861	1.519	0.882

(4*R*)-4-(4-Fluorophenyl)tetrahydro-2*H*-pyran-2-ol, S9

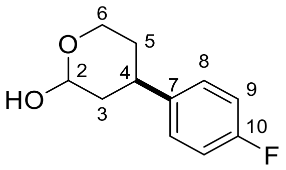

DIBAL-H in hexanes (1M, 9.0 mL, 9.0 mmol, 1.3 eq.), lactone **S8** (1.32 g, 6.79 mmol, 1 eq., 98:2 e.r.), and CH_2Cl_2 (14 mL) were subjected to **General Procedure 1**. The title compound was afforded after purification by FCC (50% Et_2O/pentane) as a white solid (1.27 g, 6.47 mmol, 95% yield, 52:48 mixture of anomers by ^{1}H NMR). Analysis by chiral HPLC was not possible, owing to extensive peak broadening, presumably due to interconversion of anomers on the HPLC timescale.

^{1}H NMR (400 MHz, $CDCl_3$) δ 7.23 – 7.10 (m, 4H, minor ArCH x 2, major ArCH x 2), 7.06 – 6.92 (m, 4H, minor ArCH x 2, minor ArCH x 2), 5.42 (s, 1H, major H_2), 4.82 (ddd, *J* = 9.5, 6.0, 2.0 Hz, 1H, minor H_2), 4.25 – 4.08 (m, 2H, minor H_{6a} and major H_{6a}), 3.75 (ddd, *J* = 11.5, 4.0, 2.5 Hz, 1H, major H_{6b}), 3.70 – 3.60 (m, 1H, minor H_{6b}), 3.56 (d, *J* = 6.0 Hz, 1H, minor OH), 3.27 – 3.16 (m, 1H, major H_4), 2.95 – 2.91 (m, 1H, major OH), 2.87 – 2.76 (m, 1H, minor H_4), 2.18 – 2.06 (m, 1H, minor H_{3a}), 1.96 (ddt, *J* = 13.5, 3.5, 1.5 Hz, 1H, major H_{3a}), 1.87 – 1.62 (m, 5H, major H_{3b}, major H_5 x 2, minor H_5 x 2), 1.55 (td, *J* = 12.5, 9.5 Hz, 1H, minor H_{3b}).

^{13}C NMR (101 MHz, $CDCl_3$) δ 161.7 (d, *J* = 244.5 Hz), 161.6 (d, *J* = 244.0 Hz), 141.3 (d, *J* = 3.2 Hz), 140.3 (d, *J* = 3.0 Hz), 128.3 (d, *J* = 8.0 Hz), 128.2 (d, *J* = 8.0 Hz), 115.5 (d, *J* = 21.0 Hz), 115.4 (d, *J* = 21.0 Hz), 96.4, 91.8, 65.9, 59.9, 40.6, 40.0, 37.9, 34.0, 33.4, 33.1.

^{19}F NMR (376 MHz, $CDCl_3$) δ -116.6 (minor), -117.0 (major).

HRMS: Acceptable ionisation could not be obtained by ESI, CI or EI methods

IR (film) ν_{max}/cm^{-1} 3336 (br), 2947, 1601, 1510, 1443, 1385, 1220, 1118, 1064, 982, 895, 808.

m.p.: 115-116 °C

α_{25}^{D}= −66.4 (c = 1.0, CH_2Cl_2).

(*R*)-4-(*o*-Tolyl)tetrahydro-2*H*-pyran-2-one, S10

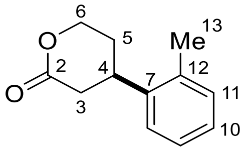

Enantioenriched: A Schlenk tube was charged with Rh(acac)(C_2H_4)$_2$ (79 mg, 0.31 mmol, 3 mol%), *(R)*-BINAP (222 mg, 0.357 mmol, 3.5 mol%), *o*-tolylboronic acid (6.93 g, 51.0 mmol, 5 eq.), and the Schlenk tube was evacuated and backfilled with N_2 three times. Dry Ar-sparged 1,4-dioxane (51 mL, 0.2 M) and water (5.1 mL) were added sequentially. The reaction mixture was degassed by evacuating and backfilling with N_2 twice and then 5,6-dihydro-2*H*-pyran-2-one (1.00 g, 10.2 mmol, 1 eq.) was added *via* syringe. The resulting mixture was heated at 100 °C for 3 h. After removal of the solvent, the residue was diluted with sat. aq. sodium bicarbonate (50 mL), extracted with EtOAc (3 x 30 mL), dried over $MgSO_4$ and concentrated *in vacuo*. The title compound was afforded after purification by FCC (30% EtOAc/pentane) as a pale yellow gum (1.61 g, 8.46 mmol, 83% yield, 97:3 e.r.). The absolute configuration was assigned as (*R*)- by analogy to **S1**, **S8** and **S12**.

Racemic: The racemic compound was prepared following the same procedure using racemic BINAP. The title compound was isolated as a white solid (138 mg, 0.73 mmol, 71% yield).

^{1}H NMR (400 MHz, $CDCl_3$) δ 7.35 – 7.21 (m, 4H, ArCH x 4), 4.59 (ddd, *J* = 11.5, 5.0, 4.5 Hz, 1H, H_{6a}), 4.48 (ddd, *J* = 11.5, 10.0, 4.0 Hz, 1H, H_{6b}), 3.54 (tdd, *J* = 10.5, 6.0, 4.5 Hz, 1H, H_4), 2.95 (ddd, *J* = 17.5, 6.0, 1.5 Hz, 1H, H_{3a}), 2.68 (dd, *J* = 17.5, 10.5 Hz, 1H, H_{3b}), 2.43 (s, 3H, H_{13} x 3), 2.25 – 2.17 (m, 1H, H_{5a}), 2.16 – 2.06 (m, 1H, H_{5b}).

^{13}C NMR (101 MHz, $CDCl_3$) δ 171.1, 141.0, 135.3, 130.9, 127.1, 126.9, 125.0, 68.7, 37.0, 33.2, 29.5, 19.4.

HRMS (ESI+): Found $[M+H]^+$ = 191.1066 ; $C_{12}H_{15}O_2$ requires 191.1067, Δ –0.07 ppm.

IR (film) ν_{max}/cm^{-1} 2962, 1732, 1402, 1254, 1215, 1072, 755, 726.

α_{25}^{D}= +8.3 (c = 1.0, $CHCl_3$).

HPLC: Enantiomeric excess was determined by HPLC with a Chiralpak® IC column (70:30 hexane:IPA, 1.0 mL min^{-1}, 210 nm, room temperature); t_r (major) = 20.9 min, t_r (minor) = 23.7 min, 97:3 e.r.

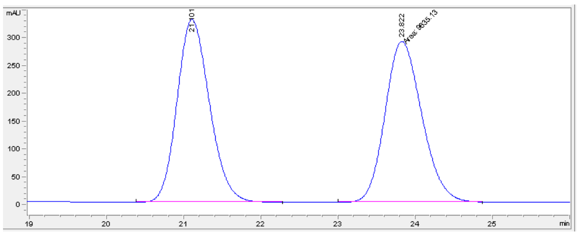

#	Time	Type	Area	Height	Width	Area%	Symmetry
1	21.101	BB	9623.3	329.2	0.4531	49.969	0.814
2	23.822	MF	9635.1	289.9	0.554	50.031	0.817

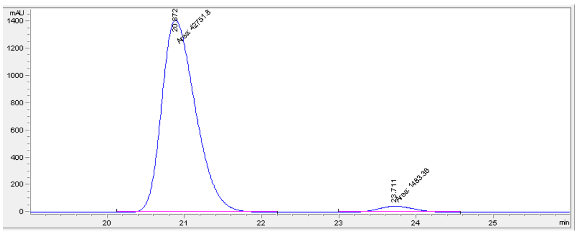

#	Time	Type	Area	Height	Width	Area%	Symmetry
1	20.872	MF	42751.8	1407.1	0.5064	96.647	0.669
2	23.711	MF	1483.4	44.9	0.5503	3.353	0.874

(4*R*)-4-(*o*-Tolyl)tetrahydro-2*H*-pyran-2-ol, S11

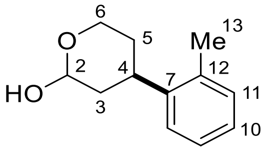

DIBAL-H in hexanes (1M, 10.1 mL, 10.1 mmol, 1.33 eq.), lactone **S10** (1.44 g, 7.57 mmol, 1 eq., 97:3 e.r.), and CH_2Cl_2 (15 mL) were subjected to **General Procedure 1**. The title compound was afforded after purification by FCC (50% Et_2O/pentane) as a white solid (1.42 g, 7.39 mmol, 98% yield, 55:45 mixture of anomers by ^{1}H NMR). Analysis by chiral HPLC was not possible, owing to extensive peak broadening, presumably due to interconversion of anomers on the HPLC timescale.

^{1}H NMR (400 MHz, $CDCl_3$) δ 7.24 – 7.10 (m, 8H, minor ArCH x 4, major ArCH x 4), 5.45 (s, 1H, major H_2), 4.87 (ddd, *J* = 9.5, 6.5, 2.0 Hz, 1H, minor H_2), 4.22 (ddd, *J* = 13.0, 11.5, 2.5 Hz, 1H, major H_{6a}), 4.16 (ddd, *J* = 11.5, 4.5, 1.5 Hz, 1H, minor H_{6a}), 3.78 (ddd, *J* = 11.5, 4.5, 2.0 Hz, 1H,

major H_{6b}), 3.74 – 3.64 (m, 2H, minor H_{6b} + minor OH), 3.49 (tt, *J* = 12.5, 3.5 Hz, 1H, major H_4), 3.06 (tt, *J* = 12.0, 4.0 Hz, 1H, minor H_4), 3.02 – 2.99 (m, 1H, major OH), 2.40 (s, 3H, major H_{13} x 3), 2.36 (s, 3H, minor H_{13} x 3), 2.06 (ddt, *J* = 13.0, 3.5, 2.0 Hz, 1H, minor H_{3a}), 1.95 – 1.56 (m, 7H, major H_3 x 2, minor H_{3b}, major H_5 x 2, minor H_5 x 2).

^{13}C NMR (101 MHz, $CDCl_3$) δ 143.3, 142.3, 135.6, 135.1, 130.5, 130.5, 126.5, 126.3, 126.3, 126.1, 125.5, 125.2, 96.6, 91.9, 66.1, 60.2, 39.7, 37.0, 36.3, 32.4, 32.0, 30.2, 19.3, 19.3.

HRMS: Acceptable ionisation could not be obtained by ESI, CI or EI methods

IR (film) ν_{max}/cm^{-1} 3374 (br), 2948, 1492, 1461, 1442, 1250, 1116, 1067, 1033, 958, 753.

m.p.: 87-90 °C

α_{25}^{D}= −38.0 (c = 1.0, CH_2Cl_2).

(*R*)-4-(4-Methoxyphenyl)tetrahydro-2*H*-pyran-2-one, S12

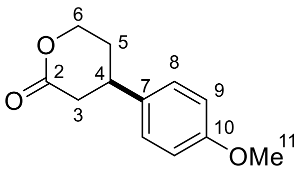

Enantioenriched: A Schlenk tube was charged with Rh(acac)(C_2H_4)$_2$ (79 mg, 0.31 mmol, 3 mol%), *(R)*-BINAP (222 mg, 0.357 mmol, 3.5 mol%), 4-methoxyphenylboronic acid (7.75 g, 51.0 mmol, 5 eq.), and the Schlenk tube was evacuated and backfilled with N_2 three times. Dry Ar-sparged 1,4-dioxane (51 mL, 0.2 M) and water (5.1 mL) were added sequentially. The reaction mixture was degassed by evacuating and backfilling with N_2 twice and then 5,6-dihydro-2*H*-pyran-2-one (1.00 g, 10.2 mmol, 1 eq.) was added *via* syringe. The resulting mixture was heated at 100 °C for 3 h. After removal of the solvent, the residue was diluted with sat. aq. sodium bicarbonate (50 mL), extracted with EtOAc (3 x 30 mL), dried over $MgSO_4$ and concentrated *in vacuo*. The title compound was afforded after purification by FCC (30% EtOAc/pentane) as a pale yellow gum (1.89 g, 9.17 mmol, 90% yield, 98:2 e.r.). The spectral data matched that reported in the literature.[14] The absolute configuration was confirmed to be (*R*)- by correlation of the specific rotation with the reported in the literature (see below).[15]

Racemic: The racemic compound was prepared following the same procedure using racemic BINAP. The title compound was isolated as a white solid (194 mg, 0.94 mmol, 92% yield).

^1H NMR (400 MHz, $CDCl_3$) δ 7.12 (d, *J* = 8.5 Hz, 2H, ArCH x 2), 6.89 (d, *J* = 8.5 Hz, 2H, ArCH x 2), 4.49 (ddd, *J* = 11.5, 5.0, 4.0 Hz, 1H, H_{6a}), 4.37 (ddd, *J* = 11.5, 10.5, 4.0 Hz, 1H, H_{6b}), 3.80 (s, 3H,

H_{11} x 3), 3.18 (tdd, *J* = 10.5, 6.0, 4.5 Hz, 1H, H_4), 2.89 (ddd, *J* = 17.5, 6.0, 1.5 Hz, 1H, H_{3a}), 2.59 (dd, *J* = 17.5, 10.5 Hz, 1H, H_{3b}), 2.20 – 2.10 (m, 1H, H_{5a}), 1.99 (dtd, *J* = 14.0, 10.5, 5.0 Hz, 1H, H_{5b}).

^{13}C NMR (101 MHz, $CDCl_3$) δ 170.8, 158.7, 134.9, 127.4, 114.3, 68.7, 55.3, 37.8, 36.7, 30.5.

$\alpha_{25}{}^{D}$= –7.7 (c = 1.0, $CHCl_3$). Lit. $\alpha_{20}{}^{D}$= –6.2 (c = 1.0, $CHCl_3$).[15]

HPLC: Enantiomeric excess was determined by HPLC with a Chiralpak® IC column (70:30 hexane:IPA, 1.0 mL min^{-1}, 210 nm, room temperature); t_r (major) = 39.2 min, t_r (minor) = 47.7 min, 98:2 e.r.

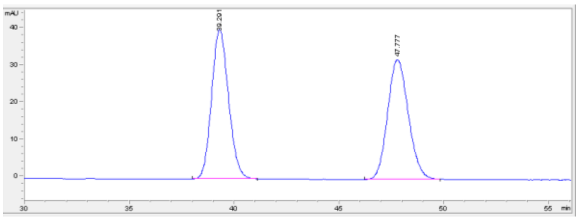

#	Time	Type	Area	Height	Width	Area%	Symmetry
1	39.291	BB	2251.7	40	0.8583	50.428	0.858
2	47.777	BB	2213.4	32.2	1.0131	49.572	0.859

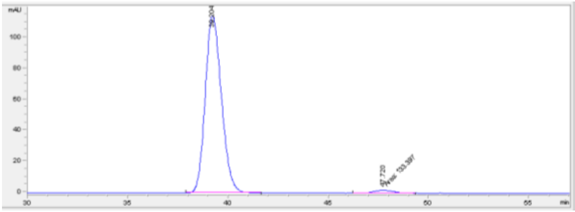

#	Time	Type	Area	Height	Width	Area%	Symmetry
1	39.204	BB	6422.2	114.5	0.8705	97.965	0.794
2	47.72	MM	133.4	1.9	1.1605	2.035	0.965

(4*R*)-4-(4-Methoxyphenyl)tetrahydro-2*H*-pyran-2-ol, S13

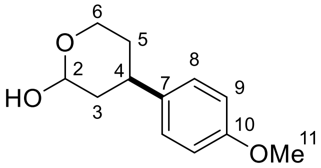

DIBAL-H in hexanes (1M, 9.8 mL, 9.8 mmol, 1.3 eq.), lactone **S12** (1.52 g, 7.37 mmol, 1 eq., 98:2 e.r.), and CH_2Cl_2 (15 mL) were subjected to **General Procedure 1**. The title compound was afforded after purification by FCC (40% Et_2O/pentane) followed by recrystallization from boiling hexane/EtOAc as a white solid (1.24 g, 5.95 mmol, 81% yield, 50:50 mixture of anomers by ^{1}H NMR). Analysis by chiral HPLC was not possible, owing to extensive peak broadening, presumably due to interconversion of anomers on the HPLC timescale, so a small sample of **S13** was oxidised to the lactone, which indicated the enantiomeric purity to be 99.5:0.5 e.r. (i.e. a small increase, as a result of crystallization). A procedure for the oxidation and HPLC data for the lactone are provided below.

^{1}H NMR (400 MHz, $CDCl_3$) δ 7.17 – 7.11 (m, 4H, ArCH x 4), 6.89 – 6.84 (m, 4H, ArCH x 4), 5.42 (s, 1H, H_2), 4.82 (ddd, *J* = 9.5, 6.5, 2.0 Hz, 1H, H_2’), 4.22 – 4.10 (m, 2H, H_{6a} and H_{6a}’), 3.82 – 3.71 (m, 8H, H_{6b}, H_{11} x 3, H_{11}’ x 3 and OH), 3.69 – 3.60 (m, 1H, H_{6b}’), 3.17 (tt, *J* = 12.0, 4.0 Hz, 1H,

H_4), 3.09-3.07 (m, 1H, OH’), 2.85 – 2.73 (m, 1H, H_4’), 2.15 – 2.08 (m, 1H, H_{3a}), 1.99 – 1.93 (m, 1H, H_{3a}’), 1.87 – 1.65 (m, 5H, H_{3b}’, H_5 x 2 and H_5’ x 2), 1.56 (td, *J* = 12.5, 9.5 Hz, 1H, H_{3b}).

^{13}C NMR (101 MHz, $CDCl_3$) δ 158.3, 158.2, 137.9, 136.9, 127.8, 127.7, 114.1, 114.1, 96.5, 91.9, 65.9, 60.0, 55.4, 40.7, 39.9, 37.9, 33.8, 33.4, 33.2. [N.B. The peak at 55.4 ppm corresponds to two overlapping signals].

HRMS (ESI+): Found $[M+Na]^+$ = 231.0992 ; $C_{12}H_{16}O_3Na$ requires 231.0992, Δ 0.30 ppm.

IR (film) ν_{max}/cm^{-1} 2924, 1692, 1422, 1270, 1243, 1176, 1148.

m.p.: 86-88 °C

α_{25}^{D}= −30.9 (c = 1.0, CH_2Cl_2).

(*R*)-4-(4-Methoxyphenyl)tetrahydro-2*H*-pyran-2-one, S12

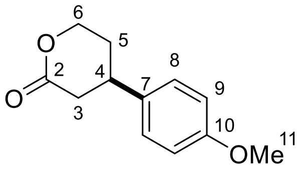

This experiment was performed to determine the e.r. of lactol **S13**. To a stirred solution of lactol **S13** (20 mg, 0.096 mmol, 1 equiv.) in anhydrous CH_2Cl_2 (1 mL) at RT, was added pyridinium chlorochromate (41 mg, 0.19 mmol, 2 equiv). The resulting solution was stirred at RT for 16 h, and then concentrated *in vacuo*. The title compound was afforded after purification by FCC (50% Et_2O/pentane) as a white solid (17 mg, 0.082 mmol, 86% yield, 99.5:0.5 e.r.). The spectral data was identical to that described for **S12** above.

HPLC: Enantiomeric excess was determined by HPLC with a Chiralpak® IC column (70:30 hexane:IPA, 1.0 mL min^{-1}, 210 nm, room temperature); t_r (major) = 38.6 min, t_r (minor) = 46.9 min, 99.5:0.5 e.r.

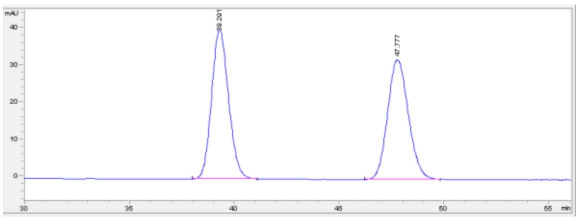

#	Time	Type	Area	Height	Width	Area%	Symmetry
1	39.291	BB	2251.7	40	0.8583	50.428	0.858
2	47.777	BB	2213.4	32.2	1.0131	49.572	0.859

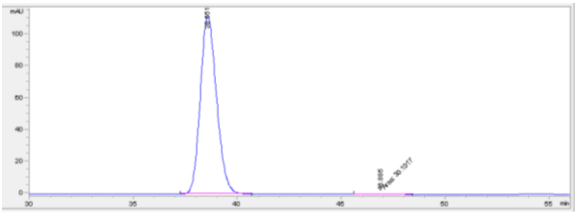

#	Time	Type	Area	Height	Width	Area%	Symmetry
1	38.551	BB	6059.4	111.2	0.8457	99.506	0.8
2	46.885	MM	30.1	3.9E-1	1.2882	0.494	0.713

(4*S*)-4-(Prop-1-en-2-yl)tetrahydro-2*H*-pyran-2-ol, S14

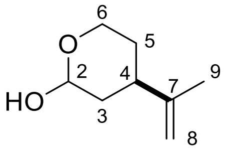

DIBAL-H in hexanes (1M, 2.5 mL, 2.5 mmol, 1.3 eq.), (*R*)-4-(prop-1-en-2-yl)tetrahydro-2*H*-pyran-2-one[16] (262 mg, 1.87 mmol, 1 eq.), and CH_2Cl_2 (3.7 mL) were subjected to **General Procedure 1**. The title compound was afforded after purification by FCC (50% Et_2O/pentane) as a colourless oil (248 mg, 1.74 mmol, 93% yield, 52:48 mixture of anomers by ^{1}H NMR). The spectral data matched that reported in the literature.[16]

^{1}H NMR (400 MHz, $CDCl_3$) δ 5.35 (s, 1H, major H_2), 4.76 – 4.68 (m, 5H, minor H_2, major H_8 x 2 and minor H_8 x 2), 4.09 – 4.02 (m, 2H, major H_{6a} and minor H_{6a}), 3.89 (d, *J* = 6.5 Hz, 1H, major OH), 3.68 (ddd, *J* = 11.0, 4.5, 2.0 Hz, 1H, minor H_{6b}), 3.53 (td, *J* = 12.0, 2.5 Hz, 1H, major H_{6b}), 3.27 – 3.25 (m, 1H, minor OH), 2.54 (tt, *J* = 12.5, 3.5 Hz, 1H, minor H_4), 2.18 (tt, *J* = 12.0, 3.5 Hz, 1H, major H_4), 1.97 (ddt, *J* = 12.5, 4.0, 2.0 Hz, 1H, minor H_{3a}), 1.89 – 1.80 (m, 1H, major H_{3a}), 1.72 (s, 6H, major H_9 x 3 and minor H_9 x 3), 1.69 – 1.39 (m, 5H, major H_5 x 2, minor H_5 x 2 and major H_{3b}), 1.30 (td, *J* = 12.5, 9.5 Hz, 1H, minor H_{3b}).

^{13}C NMR (101 MHz, $CDCl_3$) δ 148.9, 147.9, 109.6, 109.1, 96.5, 91.8, 65.8, 59.9, 41.6, 38.2, 35.4, 35.3, 30.9, 30.4, 20.8, 20.7.

α_{25}^{D}= −37.7 (c = 1.0, CH_2Cl_2).

3-Propyltetrahydro-2*H*-pyran-2-ol, S15

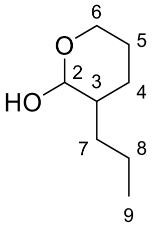

DIBAL-H in hexanes (1.23 M, 3.2 mL, 3.92 mmol, 1.3 eq.), 3-propyltetrahydro-2*H*-pyran-2-one[17] (419 mg, 2.95 mmol, 1 eq.) and CH_2Cl_2 (6 mL) were subjected to **General Procedure 1**. The title compound was afforded after purification by FCC (40% Et_2O/pentane) as a colourless oil (254 mg, 1.76 mmol, 60% yield, 53:47 mixture of anomers).

^{1}H NMR (400 MHz, $CDCl_3$) δ 5.06 (t, *J* = 3.5 Hz, 1H, minor H_2), 4.42 (dd, *J* = 7.0, 6.0 Hz, 1H, major H_2), 4.01 – 3.91 (m, 2H, major H_{6a} and minor H_{6a}), 3.58 – 3.53 (m, 1H, minor H_{6b}), 3.51 – 3.44 (m, 1H, major H_{6b}), 3.34 – 3.31 (m, 1H, major OH), 2.94 – 2.88 (m, 1H, minor OH), 1.92

(dqd, J = 13.0, 4.5, 2.0 Hz, 1H, major H_{5a}), 1.69 – 1.07 (m, 17H, major H_{5b}, major H_3, minor H_3, major H_4 x 2, minor H_4 x 2, minor H_5 x 2, major H_7 x 2, minor H_7 x 2, major H_8 x 2, minor H_8 x 2), 0.89 (t, J = 7.5 Hz, 6H, major H_9 x 3, minor H_9 x 3).

^{13}C NMR (101 MHz, $CDCl_3$) δ 99.7, 94.2, 65.4, 60.0, 41.6, 39.6, 33.9, 33.4, 27.2, 25.6, 24.9, 23.8, 19.9, 19.9, 14.4, 14.4.

HRMS (ESI+): Found $[M+Na]^+$ = 167.1045 $C_8H_{16}O_2Na$ requires 167.1043, Δ 1.72 ppm

IR (film) ν_{max}/cm^{-1} 3388 (br), 2933, 2872, 1457, 1151, 1076, 1040, 1019.

3-Allyltetrahydro-2*H*-pyran-2-ol, S16

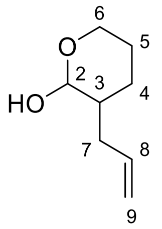

DIBAL-H in hexanes (1M, 7.3 mL, 7.3 mmol, 1.4 eq.), 3-allyltetrahydro-2*H*-pyran-2-one[18] (730 mg, 5.21 mmol, 1 eq.) and CH_2Cl_2 (20 mL) were subjected to **General Procedure 1**. The title compound was afforded after purification by FCC (20% Et_2O/pentane) as a colourless oil (682 mg, 4.79 mmol, 92% yield, 57:43 mixture of anomers).

^{1}H NMR (400 MHz, $CDCl_3$) δ 5.89 – 5.67 (m, 2H, major H_8 and minor H_8), 5.16 – 4.91 (m, 5H, minor H_2, major H_9 x 2, minor H_9 x 2), 4.46 (d, J = 7.0 Hz, 1H, major H_2), 4.10 – 3.86 (m, 2H, major H_{6a}, minor H_{6a}), 3.60 – 3.44 (m, 2H, major H_{6b}, minor H_{6b}), 3.34 (s, 1H, major OH), 2.91 (s, 1H, minor OH), 2.41 (dddt, J = 14.0, 6.5, 4.5, 1.5 Hz, 1H, major H_{7a}), 2.17 (dtt, J = 14.0, 7.0, 1.5 Hz, 1H, minor H_{7a}), 2.03 – 1.85 (m, 3H, major H_{4a}, major $H_{7b,}$ minor H_{7b}), 1.73 (pd, J = 7.5, 3.0 Hz, 1H, minor H_3), 1.67 – 1.40 (m, 7H, major H_3, minor H_4 x 2, major H_5 x 2, minor H_5 x 2), 1.29 – 1.11 (m, 1H, major H_{4b}).

^{13}C NMR (101 MHz, $CDCl_3$) δ 136.7, 136.3, 116.5, 116.3, 99.1, 93.9, 65.4, 60.1, 41.6, 39.8, 36.1, 35.7, 26.9, 25.4, 24.8, 23.6.

HRMS (ESI+): Found $[M+Na]^+$ = 165.0888 $C_8H_{14}O_2Na$ requires 165.0886, Δ 1.21 ppm

IR (film) ν_{max}/cm^{-1} 3373, 2940, 2851, 1641, 1439, 1276, 1062, 983, 910

3-(Prop-2-yn-1-yl)tetrahydro-2*H*-pyran-2-ol, S17

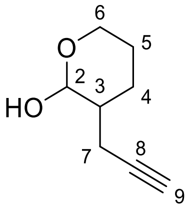

DIBAL-H in hexanes (1M, 4.0 mL, 4.0 mmol, 1.4 eq.), 3-(prop-2-yn-1-yl)tetrahydro-2*H*-pyran-2-one[19] (400 mg, 2.89 mmol, 1 eq.) and CH_2Cl_2 (10 mL) were subjected to **General Procedure 1**. The title compound was afforded after purification by FCC (20% Et_2O/pentane) as a colourless oil (381 mg, 2.72 mmol, 94% yield, 58:42 mixture of anomers).

^{1}H NMR (400 MHz, $CDCl_3$) δ 5.18 (d, *J* = 3.0 Hz, 1H, minor H_2), 4.56 (d, *J* = 7.5 Hz, 1H, major H_2), 4.08 – 3.87 (m, 2H, major H_{6a}, minor H_{6a}), 3.65 – 3.43 (m, 2H, major H_{6b}, minor H_{6b}), 3.39 (s, 1H, minor OH), 2.88 (s, 1H, major OH), 2.43 (ddd, *J* = 17.0, 4.5, 2.5 Hz, 1H, major H_{7a}), 2.34 – 2.23 (m, 2H, major $H_{7b,}$ minor H_{7a}), 2.13 (ddd, *J* = 17.0, 7.5, 2.5 Hz, 1H, minor H_{7b}), 2.06 – 1.95 (m, 3H, major H_{4a}, major H_9, minor H_9), 1.94 – 1.86 (m, 1H, minor H_3), 1.74 – 1.54 (m, 7H, major H_3, minor H_4 x 2, minor H_5 x 2, major H_5 x 2), 1.54 – 1.37 (m, 1H, major H_{4b}).

^{13}C NMR (101 MHz, $CDCl_3$) δ 98.3, 93.1, 82.8, 82.0, 70.0, 69.3, 65.7, 60.1, 40.6, 39.3, 26.8, 25.0, 24.8, 23.5, 20.8, 20.6.

HRMS (ESI+): Found $[M+H]^+$ = 141.0910; $C_8H_{13}O_2$ requires 141.0910, Δ -0.22 ppm

IR (film) ν_{max}/cm^{-1} 3373, 3292, 2942, 1432, 1277, 1075, 988, 895, 622

3-Benzyltetrahydro-2*H*-pyran-2-ol, S18

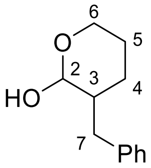

DIBAL-H in hexanes (1M, 7.3 mL, 7.3 mmol, 1.4 eq.), 3-benzyltetrahydro-2H-pyran-2-one[20] (1.00 g, 5.26 mmol, 1 eq.) and CH_2Cl_2 (20 mL) were subjected to **General Procedure 1**. The title compound was afforded after purification by FCC (20% Et_2O/pentane) as a colourless oil (1.00 g, 5.20 mmol, 99% yield, 52:48 mixture of anomers).

^{1}H NMR (400 MHz, CDCl3) δ 7.36 – 7.25 (m, 4H, ArCH x 4), 7.26 – 7.15 (m, 6H, ArCH x 6), 4.99 (d, *J* = 3.0 Hz, 1H, minor H_2), 4.55 (d, *J* = 7.0 Hz, 1H, major H_2), 4.13 – 3.88 (m, 2H, major H_{6a} and minor H_{6a}), 3.67 – 3.43 (m, 3H, major H_{6b}, minor H_{6b} and OH), 3.19 – 3.01 (m, 2H, major H_{7a} and OH), 2.74 (dd, *J* = 13.5, 7.5 Hz, 1H, minor H_{7a}), 2.54 (dd, *J* = 13.5, 8.0 Hz, 1H,

minor H_{7b}), 2.43 (dd, *J* = 13.5, 9.5 Hz, 1H, major H_{7b}), 2.05 – 1.91 (m, 1H, minor H_3), 1.86 – 1.40 (m, 8H, major H_3, major H_{4a}, major H_5 x 2, minor H_4 x 2, minor H_5 x 2), 1.33 – 1.13 (m, 1H, major H_{4b}).

^{13}C NMR (101 MHz, $CDCl_3$) δ 140.3, 139.9, 129.5, 129.2, 128.4, 126.1, 126.0, 99.0, 93.6, 65.4, 59.9, 43.5, 41.9, 38.1, 37.5, 26.6, 25.5, 24.7, 23.5. N.B. the signal at 128.4 ppm corresponds to two overlapping signals.

Acceptable ionisation could not be obtained by ESI, CI or EI methods

IR (film) ν_{max}/cm^{-1} 3381, 2941, 1496, 1454, 1290, 1128, 1073, 985, 748, 700

m.p.: 73-74 °C

4,4-Dimethyltetrahydro-2*H*-pyran-2-ol, S19

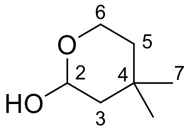

DIBAL-H in hexanes (1M, 13.4 mL, 13.4 mmol, 1.33 eq.), 4,4-dimethyltetrahydro-2*H*-pyran-2-one[9] (1.30 g, 9.83 mmol, 1 eq.) and CH_2Cl_2 (13 mL) were subjected to **General Procedure 1**. The title compound was afforded after purification by FCC (40% Et_2O/pentane) as a colourless oil (1.16 g, 8.90 mmol, 91% yield). The spectral data matched that previously reported in the literature.[21]

^{1}H NMR (400 MHz, $CDCl_3$) δ 4.91 (ddd, *J* = 8.5, 5.5, 2.5 Hz, 1H, H_2), 3.91 (ddd, *J* = 12.0, 4.5, 3.5 Hz, 1H, H_{6a}), 3.77 (d, *J* = 5.5 Hz, 1H, OH), 3.63 (ddd, *J* = 12.0, 11.0, 3.0 Hz, 1H, H_{6b}), 1.58 (ddd, *J* = 13.0, 2.5, 1.5 Hz, 1H, H_{3a}), 1.44 (ddd, *J* = 13.5, 11.0, 4.5 Hz, 1H, H_{5a}), 1.31 – 1.21 (m, 2H, H_{3b} and H_{5b}), 1.01 (s, 3H, H_{7a} x 3), 0.98 (s, 3H, H_{7b} x 3).

^{13}C NMR (101 MHz, $CDCl_3$) δ 93.4, 61.5, 45.2, 38.0, 31.8, 29.8, 26.4.

3,3-Dimethylpentane-1,1,5,5-d$_4$-1,5-diol, S20-*d$_3$*

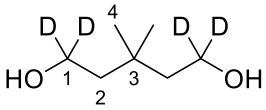

Iodine (7.52 g, 29.6 mmol, 1.2 eq) in dry THF (125 mL) was added dropwise *via* dropping funnel to a 1 litre three necked flask at 0 °C under argon atmosphere charged with 3,3-dimethylglutaric acid (3.95 g, 24.7 mmol, 1 eq.), $NaBD_4$ (3.10 g, 74.1 mmol, 3 eq.) and dry THF (125 mL). The reaction mixture was then warmed to RT and stirred for 48 hours. The

reaction was quenched with MeOH (25 mL) at 0 °C, and then concentrated *in vacuo*. The resulting residue was diluted with water (100 mL), extracted with EtOAc (3x 200 mL), dried over $MgSO_4$, filtered and concentrated *in vacuo*. The title compound was afforded after purification by FCC (EtOAc) as a yellow oil (666 mg, 4.89 mmol, 20% yield, 95% D incorporation by ^{1}H and ^{13}C NMR).

^{1}H NMR (400 MHz, $CDCl_3$) δ 3.72 (t, *J* = 7.0 Hz, 0.2H, H_1), 2.06 (s, 2H, OH x 2), 1.56 (s, 4H, H_2 x 4), 0.94 (s, 6H, H_4 x 6). [N.B. in the absence of any deuteration, the peak for H_1 (δ = 3.72 ppm) would be 4.00, the measured integral by quantitative ^{1}H NMR was 0.20 thus indicating that overall 3.80 D was incorporated across the four symmetrical positions, i.e. 95% D at each position].

^{13}C NMR (101 MHz, $CDCl_3$) δ 59.1 (1:2:3:2:1 quintet, *J* = 21.0 Hz), 44.1, 31.8, 28.2.

^{2}H NMR (92 MHz, $CHCl_3$ + 1 drop $CDCl_3$) δ 3.70 (s).

Apart from deuterium incorporation at C_1, the spectral data of **S20-*d₃*** was consistent with that of **S20** that we have prepared and reported previously.[22]

4,4-Dimethyltetrahydro-2*H*-pyran-2,6,6-d₃-2-ol, S19-*d₃*

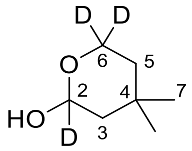

A 50 mL RBF under argon atmosphere was charged with IBX (30% wt. with stabilizer, 4.11 g, 4.41 mmol, 1.2 eq.), dry DMSO (4.3 mL) and acetone (15.2 mL), and stirred for 40 min at RT. 3,3-dimethylpentane-1,1,5,5-d_4-1,5-diol, **S20-*d₃*** (500 mg, 3.67 mmol, 1 eq.) was added, and the resulting mixture was stirred for 5 hours at RT. The reaction was then diluted with water (15 mL) and filtered. The resulting aqueous solution was extracted with Et_2O (4 x 50 mL). The organic phase was then washed with sat. aq. Na_2CO_3 (20 mL), brine (20 mL), dried over $MgSO_4$ and concentrated *in vacuo*. The title compound was afforded after purification by FCC (50% Et_2O/pentane) as a colourless oil (187 mg, 1.40 mmol, 38% yield, >95% D incorporation by ^{1}H and ^{13}C NMR).

^{1}H NMR (400 MHz, $CDCl_3$) δ 3.69 (s, 1H, OH), 1.57 (dd, *J* = 13.0, 1.5 Hz, 1H, H_{3a}), 1.42 (d, *J* = 13.0 Hz, 1H, H_{5a}), 1.35 – 1.18 (m, 2H, H_{3b} and H_{5b}), 1.01 (s, 3H, H_{7a} x 3), 0.98 (s, 3H, H_{7b} x 3). [N.B. in the absence of any deuteration, the peaks for H_2, H_{6a} and H_{6b} (see spectra) should each integrate to 1.00. Integration of the quantitative ^{1}H NMR spectrum showed an integral

for each of these peaks of 0.02-0.03 thus indicating 0.97-0.98 D incorporation at each position, i.e. >95% D at each position].

^{13}C NMR (101 MHz, $CDCl_3$) δ 92.9 (1:1:1 t, *J* = 24.5 Hz), 60.8 (1:2:3:2:1 quintet, *J* = 21.5 Hz), 45.1, 37.9, 31.7, 29.8, 26.5.

^{2}H NMR (92 MHz, $CHCl_3$ + 1 drop $CDCl_3$) δ 4.91 (s, 1H, H_2), 3.90 (s, 1H, H_{6a}), 3.62 (s, 1H, H_{6b}).

The spectral data of **S19-*d₃*** was consistent with that of **S19** (see above) with >95% incorporation of deuterium at C2 and C6 (see spectra).

3.1.2 Synthesis of HWE Reagents

Diethyl (2-oxo-2-phenylethyl)phosphonate, S21

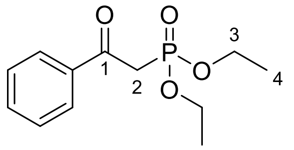

A 25 mL RBF was charged with 2-bromo acetophenone (4.0 g, 20 mmol, 1 eq.), triethyl phosphite (3.6 g, 22 mmol, 1.1 eq.) and then fitted with a reflux condenser and heated to 160 °C for 24h. The reaction mixture was then cooled to RT and purified by FCC (50-100% Et_2O/pentane) to afford the title compound as a colourless oil (4.90 g, 19.1 mmol, 96% yield). The spectroscopic data matched that previously reported in the literature.[23]

^{1}H NMR (400 MHz, $CDCl_3$) δ 8.07 – 8.00 (m, 2H, ArCH x 2), 7.65 – 7.56 (m, 1H, ArCH), 7.55 – 7.45 (m, 2H, ArCH x 2), 4.22 – 4.07 (m, 4H, H_3 x 4), 3.65 (d, *J* = 23.0 Hz, 2H, H_2 x 2), 1.30 (td, *J* = 7.0, 0.5 Hz, 6H, H_4 x 6).

^{31}P NMR (162 MHz, $CDCl_3$) δ 19.9.

^{13}C NMR (101 MHz, $CDCl_3$) δ 192.1 (d, *J* = 7.0 Hz), 136.7 (d, *J* = 1.5 Hz), 133.8, 129.2, 128.7, 62.8 (d, *J* = 6.5 Hz), 38.6 (d, *J* = 130.0 Hz), 16.4 (d, *J* = 6.5 Hz).

2-Bromo-1-(2,3,4,5,6-pentamethylphenyl)ethan-1-one, S22

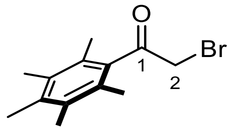

$AlCl_3$ (12.0 g, 87.8 mmol, 1.3 eq.) was added portionwise to a stirred solution of pentamethylbenzene (10.0 g, 67.5 mmol, 1 eq.), bromoacetyl bromide (6.44 mL, 74.3 mmol, 1.1 eq.) in dry CH_2Cl_2 (350 mL, 0.2 M) at 0 °C. The reaction was warmed to RT and stirred for 3 hours. The reaction mixture was then poured onto crushed ice and once melted, extracted with CH_2Cl_2 (3 x 200 mL), dried over $MgSO_4$ and concentrated *in* vacuo. The title compound was afforded after purification by FCC (7% Et_2O/pentane) as a white solid (15.0 g, 55.7 mmol, 83% yield.

^{1}H NMR (400 MHz, $CDCl_3$) δ 4.28 (s, 2H, H_2 x 2), 2.25 (s, 3H, $ArCH_3$), 2.19 (s, 6H, $ArCH_3$ x 2), 2.12 (s, 6H, $ArCH_3$ x 2).

^{13}C NMR (101 MHz, $CDCl_3$) δ 201.2, 137.1, 136.9, 133.5, 128.3, 38.2, 17.7, 16.9, 16.1.

HRMS (ESI+): Found $[M+Na]^+$ = 291.0356; $C_{13}H_{37}{}^{79}BrONa$ requires 291.0355, Δ 0.34 ppm

m.p.: 109-110 °C

IR (film) ν_{max}/cm^{-1} 2997, 2935, 1717, 1446, 1396, 1306, 1270, 1234, 1101

Diethyl (2-oxo-2-(2,3,4,5,6-pentamethylphenyl)ethyl)phosphonate, S23

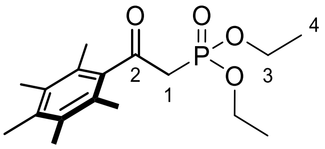

A 50 mL RBF was charged with bromide **S22** (15.8 g, 58.7 mmol, 1 eq.) and triethyl phosphite (11.3 mL, 1.1 eq., 65.9 mmol). The flask was fitted with a reflux condenser and heated at 140 °C for 24 h. The title compound was afforded by purification by FCC (40-60% EtOAc/pentane) as a white solid (11.4 g, 34.9 mmol, 59% yield).

^{1}H NMR (400 MHz, $CDCl_3$) δ 4.29 – 4.08 (m, 4H, H_3 x 4), 3.41 (d, *J* = 20.5 Hz, 2H, H_1 x 2), 2.25 (s, 3H, $ArCH_3$), 2.20 (s, 6H, $ArCH_3$ x 2), 2.19 (s, 6H, $ArCH_3$ x 2), 1.32 (td, *J* = 7.0 Hz, 0.5 Hz, 6H, H_4 x 6).

^{13}C NMR (101 MHz, $CDCl_3$) δ 202.6 (d, *J* = 7.0 Hz), 134.0, 136.1, 133.2, 127.8, 62.4 (d, *J* = 6.5 Hz), 44.2 (d, *J* = 133.0 Hz), 17.5, 16.8, 16.3 (d, *J* = 6.5 Hz), 16.0.

^{31}P NMR (162 MHz, $CDCl_3$) δ 19.8.

m.p.: 89-90 °C

HRMS (ESI+): Found $[M+H]^+$ = 327.1721; $C_{17}H_{28}O_4P$ requires 327.1720, Δ 0.26 ppm

IR (film) ν_{max}/cm^{-1} 2982, 2908, 1698, 1256, 1022, 965

Diethyl (2-(3-nitrophenyl)-2-oxoethyl)phosphonate, S24

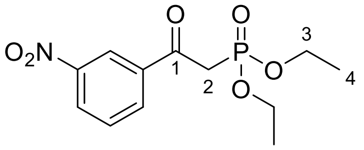

Methyl diethyl phosphonate (1.2 g, 7.9 mmol, 1.1 eq.), methyl 3-nitrobenzoate (1.3 g, 7.2 mmol, 1 eq.), diisopropylamine (2.28 mL, 16.2 mmol, 2.25 eq.), *n*-BuLi in hexanes (6.3 mL, 2.5 M, 16 mmol, 2.2 eq.) and dry THF (36 mL, 0.2 M) were subjected to **General Procedure 2**. The title compound was afforded after purification by FCC (50-100% EtOAc/pentane) as a white solid (1.20 g, 3.99 mmol, 55% yield).

^{1}H NMR (400 MHz, $CDCl_3$) δ 8.86 (t, *J* = 2.0 Hz, 1H, ArCH), 8.44 (ddd, *J* = 8.0, 2.5, 1.0 Hz, 1H, ArCH), 8.36 (ddd, *J* = 8.0, 1.5, 1.0 Hz, 1H, ArCH), 7.70 (t, *J* = 8.0 Hz, 1H, ArCH), 4.25 – 4.02 (m, 4H, H_3 x 4), 3.68 (d, *J* = 23.0 Hz, 2H, H_2 x 2), 1.29 (td, *J* = 7.0, 0.5 Hz, 6H, H_4 x 6).

^{31}P NMR (162 MHz, $CDCl_3$) δ 18.4.

^{13}C NMR (101 MHz, $CDCl_3$) δ 190.1 (d, *J* = 6.5 Hz), 148.6, 137.8, 134.8, 130.0, 128.0, 124.1, 63.1 (d, *J* = 6.5 Hz), 39.2 (d, *J* = 128.5 Hz), 16.4 (d, *J* = 6.5 Hz).

HRMS (ESI+): Found $[M+Na]^+$ = 324.0608; $C_{12}H_{16}O_6NNaP$ requires 324.0607, Δ 0.05 ppm

IR (film) ν_{max}/cm^{-1} 2980, 1689, 1534, 1351, 1265, 1021, 973, 807.

m.p.: 67-68 °C

Diethyl (2-(4-cyanophenyl)-2-oxoethyl)phosphonate, S25

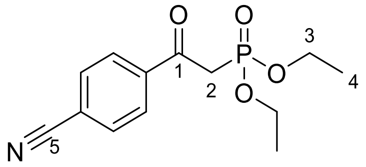

Methyl diethyl phosphonate (1.2 g, 7.9 mmol, 1.1 eq.), methyl 4-cyanobenzoate (1.16 g, 7.20 mmol, 1 eq.), diisopropylamine (2.28 mL, 16.2 mmol, 2.25 eq.), *n*-BuLi in hexanes (6.3 mL, 2.5 M, 16 mmol, 2.2 eq.) and dry THF (36 mL, 0.2 M) were subjected to **General Procedure 2**. The title compound was afforded after purification by FCC (50-100% EtOAc/pentane) as a white solid (820 mg, 2.92 mmol, 41% yield).

^{1}H NMR (400 MHz, $CDCl_3$) δ 8.14 – 8.06 (m, 2H, ArCH x 2), 7.79 – 7.73 (m, 2H, ArCH x 2), 4.19 – 4.05 (m, 4H, H_3 x 4), 3.62 (d, *J* = 23.0 Hz, 2H, H_2 x 2), 1.27 (td, *J* = 7.0, 0.5 Hz, 6H, H_4 x 6).

^{31}P NMR (162 MHz, $CDCl_3$) δ 18.5.

^{13}C NMR (101 MHz, $CDCl_3$) δ 190.9 (d, *J* = 6.5 Hz), 139.5 (d, *J* = 1.5 Hz), 132.5, 129.6, 117.9, 116.9, 63.0 (d, *J* = 6.5 Hz), 39.1 (d, *J* = 128.5 Hz), 16.3 (d, *J* = 6.5 Hz).

HRMS (ESI+): Found $[M+H]^+$ = 282.0890; $C_{13}H_{17}O_4NP$ requires 282.0890, Δ -0.04 ppm

IR (film) ν_{max}/cm^{-1} 2951, 2873, 2840, 1685, 1509, 1360, 1140, 1090, 858.

m.p.: 55-56 °C

Methyl 4-(2-(diethoxyphosphoryl)acetyl)benzoate, S26

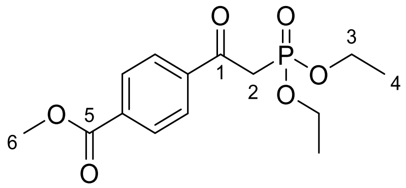

Methyl diethyl phosphonate (1.2 g, 7.9 mmol, 1.1 eq.), dimethyl terephthalate (1.4 g, 7.2 mmol, 1 eq.), diisopropylamine (2.28 mL, 16.2 mmol, 2.25 eq.), *n*-BuLi in hexanes (6.3 mL, 2.5 M, 15.8 mmol, 2.2 eq.) and dry THF (36 mL, 0.2 M) were subjected to **General Procedure 2**. The title compound was afforded after purification by FCC (50-100% Et_2O/pentane) as a colourless oil (960 mg, 3.05 mmol, 42% yield). The spectroscopic data matched that previously reported in the literature.[24]

^{1}H NMR (400 MHz, $CDCl_3$) δ 8.16 – 8.01 (m, 4H, ArCH x 4), 4.19 – 4.06 (m, 4H, H_3 x 4), 3.94 (s, 3H, H_6 x 3), 3.64 (d, *J* = 23.0 Hz, 2H, H_2 x 2), 1.26 (t, *J* = 7.0 Hz, 6H, H_4 x 6).

^{31}P NMR (162 MHz, $CDCl_3$) δ 19.2.

^{13}C NMR (101 MHz, $CDCl_3$) δ 191.7 (d, *J* = 6.5 Hz), 166.2, 139.8 (d, *J* = 2.0 Hz), 134.4, 129.9, 129.1, 62.9 (d, *J* = 6.5 Hz), 52.6, 39.0 (d, *J* = 129.5 Hz), 16.4 (d, *J* = 6.5 Hz).

Diethyl (2-(3-methoxyphenyl)-2-oxoethyl)phosphonate, S27

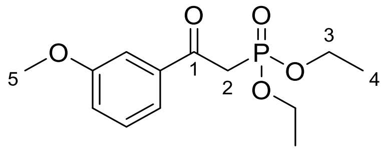

Methyl diethyl phosphonate (1.2 g, 7.9 mmol, 1.1 eq.), methyl 3-methoxybenzoate (1.2 g, 7.2 mmol, 1 eq.), diisopropylamine (2.28 mL, 16.2 mmol, 2.25 eq.), *n*-BuLi in hexanes (6.3 mL, 2.5 M, 16 mmol, 2.2 eq.) and dry THF (36 mL, 0.2 M) were subjected to **General Procedure 2**. The title compound was afforded after purification by FCC (50-100% EtOAc/pentane$_2$) as a colourless oil (1.90 g, 6.64 mmol, 92% yield). The spectroscopic data matched that previously reported in the literature.[25]

^{1}H NMR (400 MHz, $CDCl_3$) δ 7.58 (ddd, *J* = 7.5, 1.5, 1.0 Hz, 1H, ArCH), 7.52 (dd, *J* = 2.5, 1.5 Hz, 1H, ArCH), 7.37 (t, *J* = 8.0 Hz, 1H, ArCH), 7.12 (ddd, *J* = 8.5, 2.5, 1.0 Hz, 1H, ArCH), 4.27 – 4.03 (m, 4H, H_3 x 4), 3.84 (s, 3H, H_5 x 3), 3.61 (d, *J* = 23.0 Hz, 2H, H_2 x 2), 1.27 (td, *J* = 7.0, 0.5 Hz, 6H, H_4 x 6).

^{31}P NMR (162 MHz, $CDCl_3$) δ 19.9.

^{13}C NMR (101 MHz, $CDCl_3$) δ 191.9 (d, *J* = 6.5 Hz), 159.9, 138.0 (d, *J* = 2.0 Hz), 129.7, 122.0, 120.5, 112.9, 62.8 (d, *J* = 6.5 Hz), 55.6, 38.7 (d, *J* = 130.0 Hz), 16.4 (d, *J* = 6.5 Hz).

Diethyl (2-([1,1'-biphenyl]-2-yl)-2-oxoethyl)phosphonate, S28

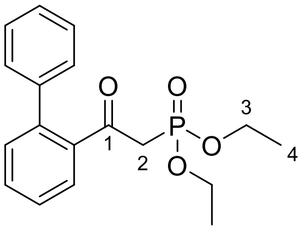

Methyl diethyl phosphonate (1.2 g, 7.9 mmol, 1.1 eq.), methyl [1,1'-biphenyl]-2-carboxylate (1.53 g, 7.21 mmol, 1 eq.), diisopropylamine (2.28 mL, 16.2 mmol, 2.25 eq.), *n*-BuLi in hexanes (6.3 mL, 2.5 M, 16 mmol, 2.2 eq.) and dry THF (36 mL, 0.2 M) were subjected to **General Procedure 2**. The title compound was afforded after purification by FCC (50-100% EtOAc/pentane) as a colourless oil (1.70 g, 5.11 mmol, 71% yield).

^{1}H NMR (400 MHz, $CDCl_3$) δ 7.61 – 7.48 (m, 2H, ArCH x 2), 7.46 – 7.30 (m, 7H, ArCH x 7), 4.04 – 3.85 (m, 4H, H_3 x 4), 2.91 (d, *J* = 22.3 Hz, 2H, H_2 x 2), 1.16 (td, *J* = 7.0, 0.5 Hz, 6H, H_4 x 6).

^{13}C NMR (101 MHz, $CDCl_3$) δ 198.6 (d, *J* = 6.5 Hz), 140.5, 140.3, 140.2, 131.2, 130.2, 129.1, 129.0, 129.0, 128.3, 127.6, 62.4 (d, *J* = 6.5 Hz), 42.2 (d, *J* = 126.5 Hz), 16.2 (d, *J* = 6.5 Hz).

^{31}P NMR (162 MHz, $CDCl_3$) δ 19.70.

HRMS (ESI+): Found $[M+H]^+$ = 333.1249; $C_{18}H_{22}O_4P$ requires 333.1250, Δ -0.43 ppm

IR (film) ν_{max}/cm^{-1} 3058, 1688, 1476, 1255, 1022, 959, 776, 703

m.p.: 68-69 ^{o}C

Diethyl (2-(naphthalen-2-yl)-2-oxoethyl)phosphonate, S29

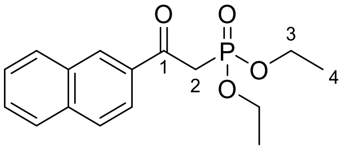

Methyl diethyl phosphonate (900 mg, 5.90 mmol, 1.1 eq.), methyl 2-naphthoate (1.00 g, 5.36 mmol, 1 eq.), diisopropylamine (1.70 mL, 12.1 mmol, 2.25 eq.), *n*-BuLi in hexanes (4.7 mL, 2.5 M, 12 mmol, 2.2 eq.) and dry THF (30 mL, 0.2 M) were subjected to **General Procedure 2**. The title compound was afforded after purification by FCC (50-100%

EtOAc/pentane) as a white solid (1.26 g, 4.11 mmol, 77% yield). The spectroscopic data matched that previously reported in the literature.[26]

^{1}H NMR (400 MHz, $CDCl_3$) δ 8.61 – 8.49 (m, 1H, ArCH), 8.05 (dd, *J* = 8.5, 2.0 Hz, 1H, ArCH), 7.98 (ddt, *J* = 8.0, 1.5, 0.5 Hz, 1H, ArCH), 7.93 – 7.84 (m, 2H, ArCH x 2), 7.69 – 7.45 (m, 2H, ArCH x 2), 4.24 – 4.04 (m, 4H, H_3 x 4), 3.76 (d, *J* = 23.0 Hz, 2H, H_2 x 2), 1.27 (td, *J* = 7.0, 0.5 Hz, 6H, H_4 x 6).

^{31}P NMR (162 MHz, $CDCl_3$) δ 20.0.

^{13}C NMR (101 MHz, $CDCl_3$) δ 191.9 (d, *J* = 6.5 Hz), 135.9, 134.0 (d, *J* = 2.0 Hz), 132.5, 131.6, 129.9, 129.0, 128.6, 127.9, 127.0, 124.3, 62.8 (d, *J* = 6.5 Hz), 38.7 (d, *J* = 130.0 Hz), 16.4 (d, *J* = 6.5 Hz).

Diethyl (2-(furan-2-yl)-2-oxoethyl)phosphonate, S30

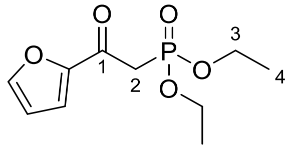

Methyl diethyl phosphonate (1.2 g, 7.9 mmol, 1.1 eq.), methyl furan-2-carboxylate (907 mg, 7.19 mmol, 1 eq.), diisopropylamine (2.28 mL, 16.2 mmol, 2.25 eq.), *n*-BuLi in hexanes (6.3 mL, 2.5 M, 16 mmol, 2.2 eq.) and dry THF (36 mL, 0.2 M) were subjected to **General Procedure 2**. The title compound was afforded after purification by FCC (100% EtOAc/pentane) as a colourless oil (1.45 g, 5.89 mmol, 82% yield. The spectroscopic data matched that previously reported in the literature.[27]

^{1}H NMR (400 MHz, $CDCl_3$) δ 7.64 (dd, *J* = 1.5, 1.0 Hz, 1H, ArCH), 7.32 (dd, *J* = 3.5, 1.0 Hz, 1H, ArCH), 6.58 (dd, *J* = 3.5, 1.5 Hz, 1H, ArCH), 4.31 – 3.97 (m, 4H, H_3 x 4), 3.51 (d, *J* = 22.5 Hz, 2H, H_2 x 2), 1.30 (td, *J* = 7.0, 0.5 Hz, 6H, H_4 x 6).

^{31}P NMR (162 MHz, $CDCl_3$) δ 19.5.

^{13}C NMR (101 MHz, $CDCl_3$) δ 180.1 (d, *J* = 7.0 Hz), 152.4 (d, *J* = 2.5 Hz), 147.2, 119.0, 112.9, 62.8 (d, *J* = 6.5 Hz), 38.4 (d, *J* = 130.0 Hz), 16.4 (d, *J* = 6.5 Hz).

Diethyl (2-(4-(dimethylamino)phenyl)-2-oxoethyl)phosphonate, S31

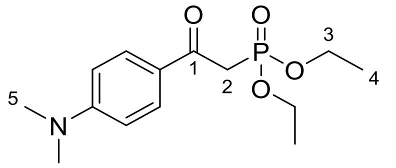

Methyl diethyl phosphonate (1.2 g, 7.9 mmol, 1.1 eq.), methyl 4-(dimethylamino)benzoate (1.29 g, 7.20 mmol, 1 eq.), diisopropylamine (2.28 mL, 16.2 mmol, 2.25 eq.), *n*-BuLi in hexanes (6.3 mL, 2.5 M, 16 mmol, 2.2 eq.) and dry THF (36 mL, 0.2 M) were subjected to **General Procedure 2** . The title compound was afforded after purification by FCC (50-100% EtOAc/pentane) as a colourless oil (1.00 g, 3.34 mmol, 46% yield).

^{1}H NMR (400 MHz, $CDCl_3$) δ 7.95 – 7.85 (m, 2H, ArCH x 2), 6.67 – 6.57 (m, 2H, ArCH x 2), 4.20 – 4.02 (m, 4H, H_3 x 4), 3.51 (d, *J* = 22.5 Hz, 2H, H_2 x 2), 3.04 (s, 6H, H_5 x 6), 1.26 (td, *J* = 7.0, 0.5 Hz, 6H, H_4 x 6).

^{31}P NMR (162 MHz, $CDCl_3$) δ 21.4.

^{13}C NMR (101 MHz, $CDCl_3$) δ 189.4 (d, *J* = 6.5 Hz), 153.8, 131.5, 124.6 (d, *J* = 2.0 Hz), 110.7, 62.5 (d, *J* = 6.5 Hz), 40.1, 37.9 (d, *J* = 129.5 Hz), 16.4 (d, *J* = 6.5 Hz).

HRMS (ESI+): Found $[M+H]^+$ = 300.1355; $C_{14}H_{23}O_4NP$ requires 300.1359, Δ -1.41 ppm

IR (film) ν_{max}/cm^{-1} 2986, 1657, 1593, 1369, 1521, 1025, 941, 791.

Diethyl (2-oxo-2-(pyridin-3-yl)ethyl)phosphonate, S32

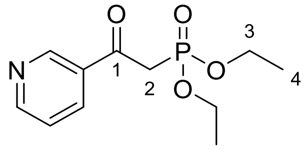

Methyl diethyl phosphonate (1.2 g, 7.9 mmol, 1.1 eq.), methyl nicotinate (0.99 g, 7.2 mmol, 1 eq.), diisopropylamine (2.28 mL, 16.2 mmol, 2.25 eq.), *n*-BuLi in hexanes (6.3 mL, 2.5 M, 16 mmol, 2.2 eq.) and dry THF (36 mL, 0.2 M) were subjected to **General Procedure 8**. The title compound was afforded after purification by FCC (2% MeOH, CH_2Cl_2) as a colourless oil (620 mg, 2.41 mmol, 33% yield). The spectroscopic data matched that previously reported in the literature.[28]

^{1}H NMR (400 MHz, $CDCl_3$) δ 9.20 (d, *J* = 2.0 Hz, 1H, ArCH), 8.79 (dd, *J* = 5.0, 1.5 Hz, 1H, ArCH), 8.30 (dt, *J* = 8.0, 2.0 Hz, 1H, ArCH), 7.43 (ddd, *J* = 8.0, 5.0, 1.0 Hz, 1H, ArCH), 4.21 – 4.05 (m, 4H, H_3 x 4), 3.63 (d, *J* = 23.0 Hz, 2H, H_2 x 2), 1.28 (t, *J* = 7.0 Hz, 6H, H_4 x 6).

^{31}P NMR (162 MHz, $CDCl_3$) δ 18.8.

^{13}C NMR (101 MHz, $CDCl_3$) δ 191.0 (d, *J* = 7.0 Hz), 153.9, 150.5, 136.5, 132.0, 123.7, 63.0 (d, *J* = 6.5 Hz), 39.1 (d, *J* = 129.0 Hz), 16.4 (d, *J* = 6.5 Hz).

Diethyl (2-oxo-2-(quinolin-6-yl)ethyl)phosphonate, S33

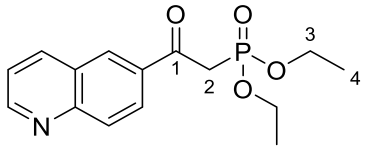

Methyl diethyl phosphonate (1.2 g, 7.9 mmol, 1.1 eq.), methyl quinoline-6-carboxylate (1.35 g, 7.21 mmol, 1 eq.), diisopropylamine (2.28 mL, 16.2 mmol, 2.25 eq.), *n*-BuLi in hexanes (6.3 mL, 2.5 M, 16 mmol, 2.2 eq.) and dry THF (36 mL, 0.2 M) were subjected to **General Procedure 2**. The title compound was afforded after purification by FCC (30-100% EtOAc/pentane) as a colourless oil (2.10 g, 6.83 mmol, 95% yield).

^{1}H NMR (400 MHz, $CDCl_3$) δ 9.00 (dd, *J* = 4.5, 1.5 Hz, 1H, ArCH), 8.55 (d, *J* = 2.0 Hz, 1H, ArCH), 8.34 – 8.23 (m, 2H, ArCH x 2), 8.14 (dt, *J* = 9.0, 0.5 Hz, 1H, ArCH), 7.47 (dd, *J* = 8.5, 4.5 Hz, 1H, ArCH), 4.24 – 4.02 (m, 4H, H_3 x 4), 3.74 (d, *J* = 23.0 Hz, 2H, H_2 x 2), 1.26 (td, *J* = 7.0, 0.5 Hz, 6H, H_4 x 6).

^{31}P NMR (162 MHz, $CDCl_3$) δ 19.49.

^{13}C NMR (101 MHz, $CDCl_3$) δ191.3 (d, *J* = 6.5 Hz), 152.9, 150.3, 137.8, 134.30 (d, *J* = 2.0 Hz), 131.3, 130.1, 128.0, 127.4, 122.08, 62.8 (d, *J* = 6.5 Hz), 38.9 (d, *J* = 129.0 Hz), 16.3 (d, *J* = 6.5 Hz).

HRMS (ESI+): Found $[M+H]^+$ = 308.1042; $C_{15}H_{19}O_4NP$ requires 308.1046, Δ -1.41 ppm

IR (film) ν_{max}/cm^{-1} 2982, 2908, 1678, 1621, 1249, 1055, 1022, 827

m.p.: 47-48 ^{o}C

Diethyl (2-(3-chlorophenyl)-2-oxoethyl)phosphonate, S34

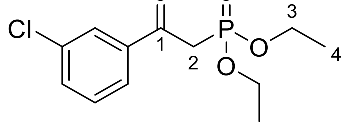

Methyl diethyl phosphonate (1.2 g, 7.9 mmol, 1.1 eq.), methyl 3-chlorobenzoate (1.23 g, 7.21 mmol, 1 eq.), diisopropylamine (2.28 mL, 16.2 mmol, 2.25 eq.), *n*-BuLi in hexanes (6.3 mL, 2.5 M, 16 mmol, 2.2 eq.) and dry THF (36 mL, 0.2 M) were subjected to **General**

Procedure 2. The title compound was afforded after purification by FCC (50% EtOAc/pentane) as a colourless oil (1.55 g, 5.33 mmol, 74% yield). The spectroscopic data matched that previously reported in the literature.[29]

^{1}H NMR (400 MHz, $CDCl_3$) δ 7.97 (t, *J* = 2.0 Hz, 1H, ArCH), 7.89 (ddd, *J* = 8.0, 2.0, 1.0 Hz, 1H, ArCH), 7.55 (ddd, *J* = 8.0, 2.0, 1.0 Hz, 1H, ArCH), 7.42 (t, *J* = 8.0 Hz, 1H, ArCH), 4.22 – 4.03 (m, 4H, H_3 x 4), 3.59 (d, *J* = 22.5 Hz, 2H, H_2 x 2), 1.28 (td, *J* = 7.0, 0.5 Hz, 6H, H_4 x 6).

^{31}P NMR (162 MHz, $CDCl_3$) δ 19.2.

^{13}C NMR (101 MHz, $CDCl_3$) δ 190.9 (d, *J* = 6.5 Hz), 138.1 (d, *J* = 2.0 Hz), 135.1, 133.7, 130.1, 129.2, 127.4, 62.9 (d, *J* = 6.5 Hz), 38.9 (d, *J* = 129.5 Hz), 16.4 (d, *J* = 6.5 Hz).

Diethyl (2-(3-iodophenyl)-2-oxoethyl)phosphonate, S35

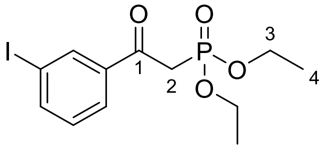

Methyl diethyl phosphonate (1.2 g, 7.9 mmol, 1.1 eq.), methyl 3-Iodobenzoate (1.88 g, 7.17 mmol, 1 eq.), diisopropylamine (2.28 mL, 16.2 mmol, 2.25 eq.), *n*-BuLi in hexanes (6.3 mL, 2.5 M, 16 mmol, 2.2 eq.) and dry THF (36 mL, 0.2 M) were subjected to **General Procedure 2** . The title compound was afforded after purification by FCC (50% EtOAc/pentane) as a colourless oil (1.9 g, 4.97 mmol, 69% yield). The spectroscopic data matched that previously reported in the literature.[29]

^{1}H NMR (400 MHz, $CDCl_3$) δ 8.32 (t, *J* = 1.5 Hz, 1H, ArCH), 7.97 (ddd, *J* = 8.0, 1.5, 1.0 Hz, 1H, ArCH), 7.90 (ddd, *J* = 8.0, 1.5, 1.0 Hz, 1H, ArCH), 7.22 (t, *J* = 8.0 Hz, 1H, ArCH), 4.24 – 4.01 (m, 4H, H_3 x 4), 3.58 (d, *J* = 23.0 Hz, 2H, H_2 x 2), 1.28 (td, *J* = 7.0, 0.5 Hz, 6H, H_4 x 6).

^{31}P NMR (162 MHz, $CDCl_3$) δ 19.2.

^{13}C NMR (101 MHz, $CDCl_3$) 190.7 (d, *J* = 7.0 Hz), 142.5, 138.3 (d, *J* = 2.0 Hz), 138.0, 130.4, 128.4, 94.4, 62.9 (d, *J* = 6.5 Hz) 38.7 (d, *J* = 129.5 Hz), 16.4 (d, *J* = 6.5 Hz).

Diethyl(2-oxo-2-(4-(4,4,5,5-tetramethyl-1,3,2-dioxaborolan-2-yl)phenyl)ethyl)phosphonate, S36

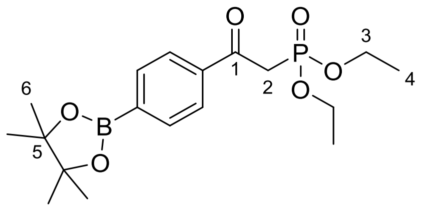

Methyl diethyl phosphonate (1.2 g, 7.9 mmol, 1.1 eq.), methyl 4-(4,4,5,5-tetramethyl-1,3,2-dioxaborolan-2-yl)benzoate[30] (1.89 g, 7.21 mmol, 1 eq.), diisopropylamine (2.28 mL, 16.2 mmol, 2.25 eq.), *n*-BuLi in hexanes (6.3 mL, 2.5 M, 16 mmol, 2.2 eq.) and dry THF (36 mL, 0.2 M) were subjected to **General Procedure 2**. The title compound was afforded after purification by FCC (50-100% EtOAc/pentane) as a colourless oil (1.65 g, 4.32 mmol, 60% yield).

^{1}H NMR (400 MHz, $CDCl_3$) δ 8.03 – 7.83 (m, 4H, ArCH x 4), 4.19 – 4.05 (m, 4H, H_3 x 4), 3.63 (d, *J* = 23.0 Hz, 2H, H_2 x 2), 1.35 (s, 12H, H_6 x 12), 1.27 (t, *J* = 7.0 Hz, 6H, H_4 x 6).

^{13}C NMR (101 MHz, $CDCl_3$) δ 192.4 (d, *J* = 6.5 Hz), 138.5 (d, *J* = 2.0 Hz), 135.0, 128.1, 84.4, 62.8 (d, *J* = 6.5 Hz), 38.7 (d, *J* = 130.0 Hz), 25.0, 16.4 (d, *J* = 6.5 Hz). n.b. ArC attached to boron was not observed due to quadrapolar relaxtion.

^{31}P NMR (162 MHz, $CDCl_3$) δ 19.8.

HRMS (ESI+): Found $[M+H]^+$ = 383.1787; $C_{18}H_{29}O_6BP$ requires 383.1790, Δ -0.26 ppm

IR (film) ν_{max}/cm^{-1} 2988, 1681, 1509, 1360, 1275, 1088, 1024, 962, 822.

Methyl ferrocene carboxylate, S37

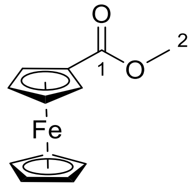

Ferrocenecarboxylic acid (3.0 g, 13 mmol), concentrated sulphuric acid (2 mL) and MeOH (30 mL, 0.4 M) were subjected to **General Procedure 7**. The title compound was afforded after purification by FCC (5-10% Et_2O/pentane) as a red oil (2.0 g, 8.2 mmol, 62% yield). The spectroscopic data matched that previously reported in the literature.[31]

^{1}H NMR (400 MHz, $CDCl_3$) δ 4.80 (s, 2H, ArCH x 2), 4.39 (s, 2H, ArCH x 2), 4.20 (s, 5H, ArCH x 5), 3.80 (s, 3H, H_2 x 3).

^{13}C NMR (101 MHz, $CDCl_3$) δ 172.4, 71.4, 71.2, 70.3, 69.9, 51.7.

Diethyl (2-oxo-2-ferrocene ethyl)phosphonate, S38

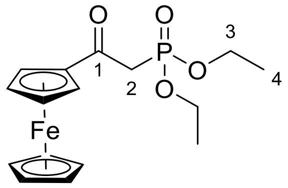

Methyl diethyl phosphonate (1.2 g, 7.9 mmol, 1.1 eq.), methyl ester **S37** (1.76 g, 7.21 mmol, 1 eq.), diisopropylamine (2.28 mL, 16.2 mmol, 2.25 eq.), *n*-BuLi in hexanes (6.3 mL, 2.5 M, 16 mmol, 2.2 eq.) and dry THF (36 mL, 0.2 M) were subjected to **General Procedure 2**. The title compound was afforded after purification by FCC (50-100% EtOAc/pentane) as a red oil (2.6 g, 7.1 mmol, 99% yield).

^{1}H NMR (400 MHz, $CDCl_3$) δ 4.83 (t, J = 2.0 Hz, 2H, ArCH x 2), 4.55 (t, J = 2.0 Hz, 2H, ArCH x 2), 4.24 (s, 5H, ArCH x 5), 4.22 – 4.12 (m, 4H, H_3 x 4), 3.39 (d, J = 22.0 Hz, 2H, H_2 x 2), 1.32 (t, J = 7.0 Hz, 6H, H_4 x 4).

^{31}P NMR (162 MHz, $CDCl_3$) δ 20.7.

^{13}C NMR (101 MHz, $CDCl_3$) δ 195.4, 79.1, 73.1, 70.2, 70.2, 62.6 (d, J = 6.0 Hz), 39.7 (d, J = 132.0 Hz), 16.5 (d, J = 6.0 Hz).

HRMS (ESI+): Found $[M+H]^+$ = 365.0608; $C_{16}H_{21}FeO_4P$ requires 365.0600, Δ 2.94 ppm

IR (film) ν_{max}/cm^{-1} 2981, 2901, 1659, 1452, 1250, 1019, 967, 823, 782.

Diethyl (2-cyclohexyl-2-oxoethyl)phosphonate, S39

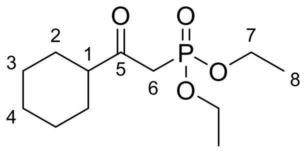

Methyl diethyl phosphonate (1.18 g, 7.76 mmol, 1.1 eq.), methyl cyclohexanecarboxylate (1.0 g, 7.0 mmol, 1 eq.), LDA in THF (2M, 7.7 mL, 15 mmol) and dry THF (15 mL, 0.47 M) were subjected to **General Procedure 2**. The title compound was afforded after purification by FCC (60% Et_2O/pentane) as a colourless oil (1.4 g, 5.3 mmol, 76% yield). The spectroscopic data matched that previously reported in the literature.[32]

^{1}H NMR (400 MHz, $CDCl_3$) δ 4.23 – 4.06 (m, 4H, H_7 x 4), 3.11 (d, *J* = 22.5 Hz, 2H, H_6 x 2), 2.59 (tt, *J* = 11.0, 3.5 Hz, 1H, H_1), 1.95 – 1.59 (m, 5H, H_{2a} x 2, H_{3a} x 2, H_{4a}) 1.36 – 1.13 (m, 11H, H_{2b} x 2, H_{3b} x 2, H_{4b}, H_8 x 6).

^{31}P NMR (162 MHz, $CDCl_3$) δ 20.4.

^{13}C NMR (101 MHz, $CDCl_3$) δ 205.6 (d, *J* = 6.0 Hz), 62.6 (d, *J* = 6.5 Hz), 51.6, 40.4 (d, *J* = 128.5 Hz), 28.4, 25.9, 25.7, 16.5 (d, *J* = 6.5 Hz).

Methyl 3,7-dimethyloct-6-enoate, S40

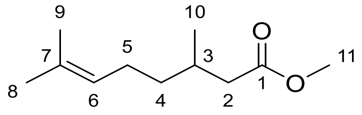

Dimethyl sulfate (1.3 mL, 1.2 equiv., 14 mmol) was added to a 50 mL RBF charged with citronellic acid (2.0 g, 1 equiv., 12.0 mmol), acetone (22 mL, 0.5 M) and potassium carbonate (2.0 g, 1.2 equiv., 14 mmol). The reaction vessel was then fitted with a reflux condenser and refluxed for 2 hours. The reaction mixture was then cooled to RT, diluted with water (50 mL), extracted with diethyl ether (3 x 50 mL), dried over sodium sulfate, brine and then concentrated *in vacuo*. The title compound was afforded after purification by FCC (5% Et_2O/pentane) as a colourless oil (1.95 g, 10.5 mmol, 90% yield). The spectroscopic data matched that previously reported in the literature.[33]

^{1}H NMR (400 MHz, $CDCl_3$) δ 5.13 – 5.03 (m, 1H, H_6), 3.66 (s, 3H, H_{11} x 3), 2.32 (dd, *J* = 14.5, 6.0 Hz, 2H, H_{2a}), 2.11 (dd, *J* = 14.0, 8.0 Hz, 1H, H_{2b}), 2.05 – 1.88 (m, 3H, H_3, H_5 x 2), 1.67 (s, 3H, H_8 x 3 or H_9 x 3), 1.60 (s, 3H, H_8 x 3 or H_9 x 3), 1.41 – 1.28 (m, 1H, H_{4a}), 1.27 – 1.15 (m, 1H, H_{4b}), 0.94 (d, *J* = 6.5 Hz, 3H, H_{10} x 3).

^{13}C NMR (101 MHz, $CDCl_3$) δ 173.9, 131.7, 124.4, 51.5, 41.7, 36.9, 30.2, 25.8, 25.6, 19.8, 17.8.

Diethyl (4,8-dimethyl-2-oxonon-7-en-1-yl)phosphonate, S41

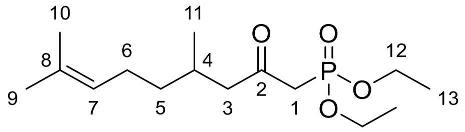

Methyl diethyl phosphonate (1.2 g, 8 mmol, 1.1 eq.), methyl ester **S40** (1.33 mg, 7.2 mmol, 1 eq.), diisopropylamine (2.28 mL, 16.2 mmol, 2.25 eq.), *n*-BuLi in hexanes (6.3 mL, 2.5 M, 15.8 mmol, 2.2 eq.) and dry THF (36 mL, 0.2 M) were subjected to **General Procedure 2**. The

title compound was afforded after purification by FCC (50% EtOAc/pentane) as a colourless oil (1.95 g, 6.41 mmol, 89% yield

^{1}H NMR (400 MHz, $CDCl_3$) δ 5.06 (t hept, *J* = 7.0, 1.5 Hz, 1H, H_7), 4.20 – 4.06 (m, 4H, H_{12} x 4), 3.15 – 2.94 (m, 2H, H_1 x 2), 2.59 (dd, *J* = 17.0, 5.5 Hz, 1H, H_{3a}), 2.44 (dd, *J* = 17.0, 8.0 Hz, 1H, H_{3b}), 2.14 – 1.84 (m, 3H, H_4, H_6 x 2), 1.66 (s, 3H, H_{10} x 3 or H_9 x 3), 1.58 (s, 3H, H_{10} x 3 or H_9 x 3), 1.38 – 1.12 (m, 8H, H_5 x 2, H_{13} x 6), 0.90 (d, *J* = 6.5 Hz, 3H, H_{11} x 3).

^{31}P NMR (162 MHz, $CDCl_3$) δ 20.0.

^{13}C NMR (101 MHz, $CDCl_3$) δ 202.0 (d, *J* = 6.0 Hz), 131.7, 124.4, 62.6 (d, *J* = 6.5 Hz), 51.5, 42.8 (d, *J* = 127.0 Hz), 36.9, 28.7, 25.8, 25.6, 19.7, 17.8, 16.4 (d, *J* = 6.5 Hz).

HRMS (ESI+): Found $[M+H]^+$ = 305.1876; $C_{15}H_{30}O_4P$ requires 305.1874, Δ -0.74 ppm

IR (film) ν_{max}/cm^{-1} 2973, 2937, 1713, 1465, 1365, 1254, 1018, 966

tert-Butyl 4-(2-(diethoxyphosphoryl)acetyl)piperidine-1-carboxylate, S42

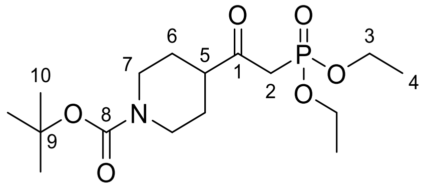

Methyl diethyl phosphonate (1.2 g, 7.9 mmol, 1.1 eq.), 1-(tert-butyl) 4-methyl piperidine-1,4-dicarboxylate (1.75 g, 7.19 mmol, 1 eq.), diisopropylamine (2.28 mL, 16.2 mmol, 2.25 eq.), *n*-BuLi in hexanes (6.3 mL, 2.5 M, 16 mmol, 2.2 eq.) and dry THF (36 mL, 0.2 M) were subjected to **General Procedure 2** . The title compound was afforded after purification by FCC (50-100% EtOAc/pentane) as a colourless oil (2.1 g, 5.8 mmol, 80% yield).

^{1}H NMR (400 MHz, $CDCl_3$) δ 4.20 – 3.98 (m, 6H, H_3 x 4, H_{7a} x 2), 3.10 (d, *J* = 23.0 Hz, 2H, H_2), 2.82 – 2.68 (m, 3H, H_5, H_{7b} x 2), 1.90 – 1.77 (m, 2H, H_{6a} x 2), 1.57 – 1.39 (m, 11H, H_{6b} x 2, H_{10} x 9), 1.36 – 1.26 (m, 6H, H_4 x 6).

^{31}P NMR (162 MHz, $CDCl_3$) δ 19.7.

^{13}C NMR (101 MHz, $CDCl_3$) δ 203.8 (d, *J* = 6.0 Hz), 154.7, 79.7, 62.7 (d, *J* = 6.5 Hz), 49.3, 43.2 (br s), 40.6 (d, *J* = 128.0 Hz), 28.5, 27.4, 16.4 (d, *J* = 6.5 Hz).

HRMS (ESI+): Found $[M+H]^+$ = 364.1884; $C_{16}H_{31}O_6NP$ requires 364.1884, Δ 0.10 ppm

IR (film) ν_{max}/cm^{-1} 2931, 1689, 1422, 1238, 1160, 1012, 963, 770.

3.1.3 Synthesis of Cross Metathesis Precursors

1-(1,4(1,4)-dibenzenacyclohexaphane-1^2-yl)prop-2-en-1-one, S43

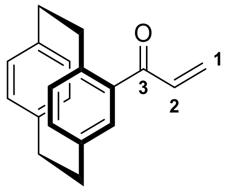

To a mixture of 4-acetyl[2.2]paracyclophane (1.00 g, 3.99 mmol, 1 equiv.) and paraformaldehyde (239 mg, 7.99 mmol, 2 equiv.) in dry THF (4 mL, 1 M), was added *i*-Pr_2NH:TFA (860 mg, 3.99 mmol, 1 equiv.) and trifluoroacetic acid (0.03 mL, 0.4 mmol, 0.1 equiv.). The reaction mixture was stirred under reflux for 2 h. Once the reaction mixture became clear, it was cooled to room temperature and a second portion of paraformaldehyde (239 mg, 7.99 mmol, 2 equiv.) was added. The reaction mixture was then stirred under reflux for an additional 6 h. After removal of the solvent, the residue was diluted with Et_2O, washed with 1M NaOH, 1M HCl and brine. The solution mixture was dried over $MgSO_4$ and concentrated *in vacuo*. The title compound was afforded after purification by FCC (5% EtOAc/pentane) as a white solid (339 mg, 0.935 mmol, 23% yield).

^{1}H NMR (400 MHz, $CDCl_3$) δ 6.83 (d, *J* = 2.0 Hz, 1H, ArH), 6.76 (dd, *J* = 17.5, 10.5 Hz, 1H, H_2), 6.66 (dd, *J* = 8.0, 2.0 Hz, 1H, ArH), 6.62 – 6.49 (m, 4H, ArH x 4), 6.35 (dd, *J* = 8.5, 1.5 Hz, 1H, ArH), 6.19 (dd, *J* = 17.5, 1.5 Hz, 1H, H_{1a}), 5.84 (dd, *J* = 10.5, 1.5 Hz, 1H, H_{1b}), 3.68 (ddd, *J* = 12.5, 10.0, 2.0 Hz, 1H, Paracyclophane-CH_2), 3.29 – 2.95 (m, 6H, Paracyclophane-CH_2 x 6), 2.89 (ddd, *J* = 12.5, 10.0, 6.0 Hz, 1H, Paracyclophane-CH_2).

^{13}C NMR (101 MHz, $CDCl_3$) δ 193.5, 141.5, 134.0, 139.7, 139.3, 137.2, 136.3, 136.1, 136.0, 133.6, 132.8, 132.6, 132.5, 131.2, 129.4, 35.5, 35.4, 35.2, 35.1.

HRMS (ESI+): Found $[M+H]^+$ = 263.1429; $C_{19}H_{19}O$ requires 263.1430, Δ -0.37 ppm

IR (film) ν_{max}/cm^{-1}: 2980, 2888, 1664, 1602, 1473, 1462.

m.p.: 103-104 °C

3.1.4 Synthesis of Cuprate Addition Precursors

7-Hydroxy-1-phenylhept-2-yn-1-one, S44

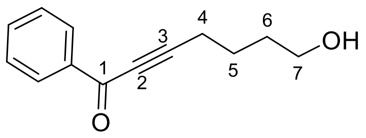

A solution of *n*-BuLi in hexanes (23 mL, 2.5M, 58 mmol, 2.4 equiv.) was added dropwise to a solution of 5-hexyn-1-ol (2.83 g, 1.2 equiv, 28.8 mmol) in dry THF (100 mL, 0.25M) cooled to –78 °C under a nitrogen atmosphere. The resulting solution was stirred for 1 hour, and then a solution of *N*-methoxy-*N*-methylbenzamide (3.39 g, 1 equiv., 24.0 mmol) in THF (10 mL) was added dropwise by syringe. The reaction mixture was then warmed to RT and stirred for 1 hour. The reaction was then quenched with sat. aq. ammonium chloride (50 mL), and extracted with EtOAc (3 x 100 mL). The combined organic layers were then dried over $MgSO_4$, and concentrated *in vacuo.* The title compound was afforded after purification by FCC (combiflash rf, 10 -> 50% EtOAc/cyclohexane) as a yellow oil (4.15 g, 20.5 mmol, 86% yield).

^{1}H NMR (400 MHz, $CDCl_3$) δ 8.17 – 8.10 (m, 2H, ArCH x 2), 7.65 – 7.53 (m, 1H, ArCH), 7.53 – 7.41 (m, 2H, ArCH x 2), 3.72 (t, *J* = 6.0, Hz, 2H, H_7 x 2), 2.56 (t, *J* = 6.5 Hz, 1H, H_4 x 2), 1.92 – 1.69 (m, 4H, H_5 x 2, H_6 x 2), 1.60 (s, 1H, OH).

^{13}C NMR (101 MHz, $CDCl_3$) δ 178.4, 137.0, 134.1, 129.7, 128.7, 96.4, 80.0, 62.3, 31.9, 24.4, 19.2.

HRMS (ESI+): Found $[M+H]^+$ = 203.1067; $C_{13}H_{15}O_2$ requires 203. 1067 Δ 0.20 ppm

IR (film) ν_{max}/cm^{-1} 3389, 2938, 2869, 2236, 2200, 1639, 1449, 1265, 701.

3.2 Synthesis of Cyclohexenes

3.2.1 Synthesis of Tetrahydropyrans

2-(4-Methyltetrahydro-2*H*-pyran-2-yl)-1-phenylethan-1-one, 4d

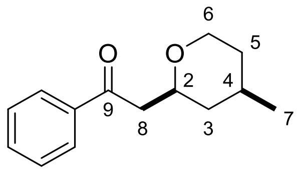

major *(cis)*

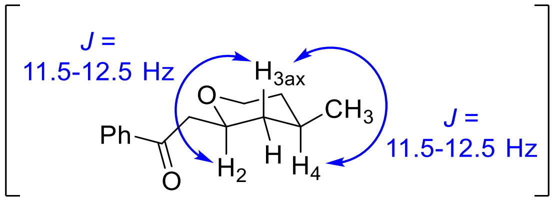

Phosphonate **S21** (356 mg, 1.4 equiv., 1.40 mmol), dry toluene (5 mL) 60% wt. NaH (48 mg, 1.2 eq., 1.2 mmol) and lactol **1d** (116 mg, 1 equiv., 1.00 mmol) were subjected to **General Procedure 3**. The title compound was afforded after purification by FCC (10% Et_2O/pentane) as a colourless oil (201 mg, 0.920 mmol, 92% yield, 94:6 *cis*/*trans*).

^{1}H NMR (400 MHz, $CDCl_3$) δ 8.01 – 7.92 (m, 2H, ArCH x 2), 7.61 – 7.51 (m, 1H, ArCH), 7.50 – 7.41 (m, 2H, ArCH x 2), 4.01 – 3.89 (m, 2H, H_2, H_{6eq}), 3.46 (ddd, *J* = 12.5, 11.5, 2.0 Hz, 1H, H_{6ax}), 3.30 (dd, *J* = 16.0, 6.5 Hz, 1H, H_{8a}), 2.93 (dd, *J* = 16.0, 6.0 Hz, 1H, H_{8b}), 1.81 – 1.60 (m, 2H, H_{3eq}, H_4), 1.54 (ddq, *J* = 13.5, 4.0, 2.0 Hz, 1H, H_{5eq}), 1.32 – 1.15 (m, 1H, H_{5ax}), 1.00 (dt, *J* = 12.5, 11.5 Hz, 1H, H_{3ax}), 0.94 (d, *J* = 6.5 Hz, 3H, H_7 x 3). The minor diastereoisomer displays diagnostic signals at δ = 4.30 (dtd, *J* = 9.5, 6.5, 3.0 Hz, 1H, H_2), 3.77 – 3.68 (m, 2H, H_6 x 2), 2.13 – 2.00 (m, 1H, H_4), 1.10 (d, *J* = 7.0 Hz, 3H, H_7 x 3).

^{13}C NMR (101 MHz, $CDCl_3$) δ 198.5, 137.4, 133.2, 128.7, 128.4, 74.2, 68.4, 45.4, 40.8, 34.6, 30.3, 22.4. The minor diastereoisomer displays diagnostic signals at δ = 63.0, 44.3, 37.7, 32.2, 25.1, 18.7.

HRMS (ESI+): Found $[M+H]^+$ = 219.1381; $C_{14}H_{19}O_2$ requires 219.1380, Δ 0.69 ppm

IR (film) ν_{max}/cm^{-1} 2978, 2842, 1683, 1597, 1489, 1382, 1079, 750, 689.

2-(-4-Methyltetrahydro-*2H*-pyran-2-yl)-1-(2,3,4,5,6-pentamethylphenyl)ethan-1-one, 4e

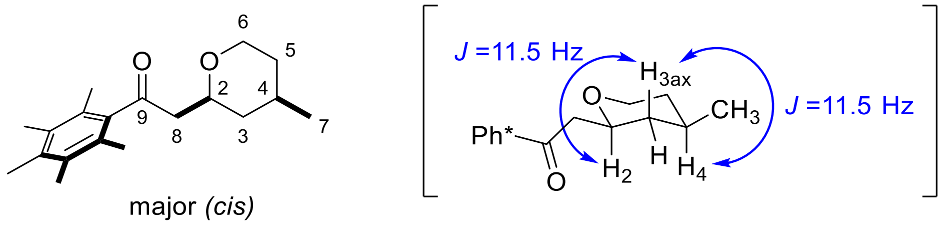

Phosphonate **S23** (456 mg, 1.4 equiv., 1.40 mmol), dry toluene (5 mL) 60% wt. NaH (48 mg, 1.2 eq., 1.2 mmol) and lactol **1d** (116 mg, 1 equiv., 1.00 mmol) were subjected to **General Procedure 3**. The title compound was afforded after purification by FCC (5 → 10% Et_2O/pentane) as a white solid (277 mg, 0.960 mmol, 96% yield, >95:5 *cis*/*trans*).

^1H NMR (400 MHz, $CDCl_3$) δ 4.04 – 3.93 (m, 2H, H_2 and H_{6eq}), 3.52 (ddd, *J* = 12.5, 11.5, 2.0 Hz, 1H, H_{6ax}), 3.01 (dd, *J* = 18.0, 7.0 Hz, 1H, H_{8a}), 2.73 (dd, *J* = 18.0, 5.5 Hz, 1H, H_{8b}), 2.26 (s, 3H, $ArCH_3$), 2.21 (s, 6H, $ArCH_3$ x 2), 2.16 (s, 6H, $ArCH_3$ x 2), 1.82 (ddt, *J* = 13.0, 4.0, 2.0 Hz, 1H, H_{3eq}), 1.79 – 1.66 (m, 1H, H_4), 1.58 (ddq, *J* = 13.5, 4.0, 2.0 Hz, 1H, H_{5eq}), 1.25 (qd, *J* = 12.5, 4.5 Hz, 1H, H_{5ax}), 0.99 (q, *J* = 11.5 Hz, 1H, H_{3ax}), 0.98 (d, *J* = 6.5 Hz, 3H, H_7 x 3).

^{13}C NMR (101 MHz, $CDCl_3$) δ 209.7, 140.5, 135.5, 133.2, 127.6, 72.9, 68.2, 52.3, 40.6, 34.5, 30.3, 22.4, 17.1, 16.8, 16.0.

HRMS (ESI+): Found $[M+H]^+$ = 289.2163; $C_{19}H_{29}O_2$ requires 289.2162, Δ 0.35 ppm

IR (film) ν_{max}/cm^{-1} 2924, 1690, 1655, 1455, 1382, 1306, 1195, 1093, 915, 732

m.p.: 246-248 °C

2-(4-Methyltetrahydro-2*H*-pyran-2-yl)-1-(3-nitrophenyl)ethan-1-one, 4f

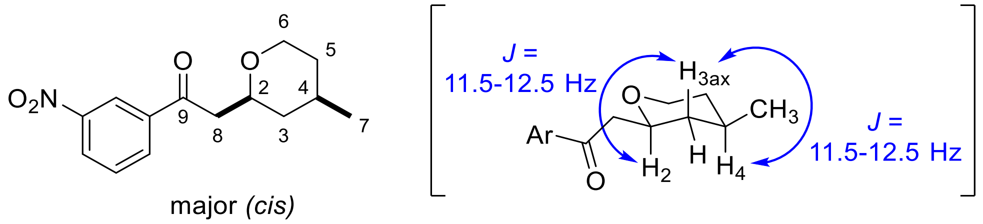

Phosphonate **S24** (420 mg, 1.4 equiv., 1.40 mmol), dry toluene (5 mL) 60% wt. NaH (48 mg, 1.2 eq., 1.2 mmol) and lactol **1d** (116 mg, 1 equiv., 1.00 mmol) were subjected to **General Procedure 3**. The title compound was afforded after purification by FCC (20% Et_2O/pentane) as a colourless oil (180 mg, 0.683 mmol, 68% yield, 92:8 *cis*/*trans*).

^1H NMR (400 MHz, $CDCl_3$) δ 8.79 (t, *J* = 2.0 Hz, 1H, ArCH), 8.40 (ddd, *J* = 8.0, 2.5, 1.0 Hz, 1H, ArCH), 8.29 (ddd, *J* = 7.5, 1.5, 1.0 Hz, 1H, ArCH), 7.66 (t, *J* = 8.0 Hz, 1H, ArCH), 3.97 – 3.88 (m,

2H, H_2, H_{6eq}), 3.43 (ddd, J = 12.5, 11.5, 2.5 Hz, 1H, H_{6ax}), 3.32 (dd, J = 16.0, 7.5 Hz, 1H, H_{8a}), 2.92 (dd, J = 16.0, 5.0 Hz, 1H, H_{8b}), 1.81 – 1.60 (m, 2H, H_{3eq}, H_4), 1.54 (ddq, J = 13.5, 4.0, 2.0 Hz, 1H, H_{5eq}), 1.28 – 1.15 (m, 1H, H_{5ax}), 1.04 (dt, J = 12.5, 11.5 Hz, 1H, H_{3ax}), 0.95 (d, J = 6.5 Hz, 3H, H_7 x 3). The minor diastereoisomer displays diagnostic signals at δ = 4.28 (dddd, J = 10.0, 7.5, 5.0, 3.0 Hz, 1H, H_2), 3.73 – 3.68 (m, 2H, H_6 x 2), 2.15 – 2.01 (m, 1H, H_4), 1.11 (d, J = 7.0 Hz, 3H, H_7 x 3).

^{13}C NMR (101 MHz, $CDCl_3$) δ 196.7, 148.6, 138.8, 134.0, 129.9, 127.4, 123.5, 74.1, 68.4, 45.6, 40.7, 34.4, 30.3, 22.4. The minor diastereoisomer displays diagnostic signals at δ = 69.1, 63.0, 44.7, 37.7, 31.9, 25.1, 18.5.

HRMS (ESI+): Found $[M+H]^+$ = 264.1231; $C_{14}H_{18}O_4N$ requires 264.1230, Δ 0.28 ppm

IR (film) ν_{max}/cm^{-1} 2981, 1696, 1533, 1382, 1350, 1256, 1090.

4-(2-(4-Methyltetrahydro-2*H*-pyran-2-yl)acetyl)benzonitrile, 4g

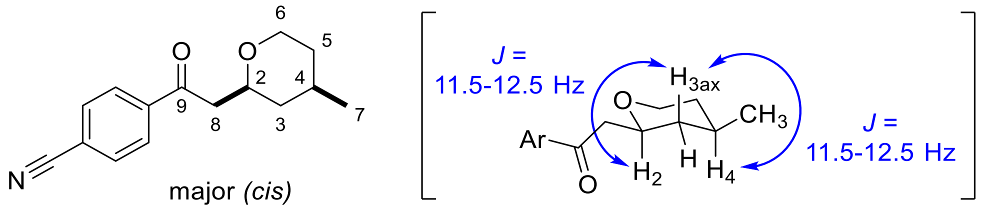

Phosphonate **S25** (393 mg, 1.4 equiv., 1.40 mmol), dry toluene (5 mL) 60% wt. NaH (48 mg, 1.2 eq., 1.2 mmol) and lactol **1d** (116 mg, 1 equiv., 1.00 mmol) were subjected to **General Procedure 3**. The title compound was afforded after purification by FCC (20% Et_2O/pentane) as a white solid (243 mg, 1.00 mmol, 100% yield, 91:9 *cis*/*trans*).

^{1}H NMR (400 MHz, $CDCl_3$) δ 8.08 – 7.99 (m, 2H, ArCH x 2), 7.80 – 7.72 (m, 2H, ArCH x 2), 3.97 – 3.85 (m, 2H, H_2, H_{6eq}), 3.46 – 3.38 (m, 1H, H_{6ax}), 3.28 (dd, J = 16.0, 7.5 Hz, 1H, H_{8a}), 2.88 (dd, J = 16.0, 5.0 Hz, 1H, H_{8b}), 1.80 – 1.49 (m, 3H, H_{3eq}, H_4, H_{5eq}), 1.27 – 1.14 (m, 1H, H_{5ax}), 1.01 (dt, J = 12.5, 11.5 Hz, 1H, H_{3ax}), 0.95 (d, J = 6.5 Hz, 3H, H_7 x 3). The minor diastereoisomer displays diagnostic signals at δ = 4.25 (dddd, J = 10.0, 7.5, 5.0, 3.0 Hz, 1H, H_2), 3.73 – 3.66 (m, 2H, H_6 x 2), 2.13 – 2.02 (m, 1H, H_4), 1.11 (d, J = 7.0 Hz, 3H, H_7 x 3).

^{13}C NMR (101 MHz, $CDCl_3$) δ 197.6, 140.5, 132.6, 128.9, 118.1, 116.4, 74.1, 68.4, 45.7, 40.7, 34.5, 30.3, 22.4. The minor diastereoisomer displays diagnostic signals at δ = 69.1, 63.0, 44.8, 37.7, 31.9, 25.1, 18.5.

HRMS (ESI+): Found $[M+H]^+$ = 244.1334; $C_{15}H_{18}O_2N$ requires 244.1332, Δ 0.64 ppm

IR (film) ν_{max}/cm^{-1} 2981, 2952, 1691, 1382, 1258, 1174, 1089, 941, 827.

m.p.: 48-49 ^{o}C

Methyl 4-(2-(4-methyltetrahydro-2*H*-pyran-2-yl)acetyl)benzoate, 4h

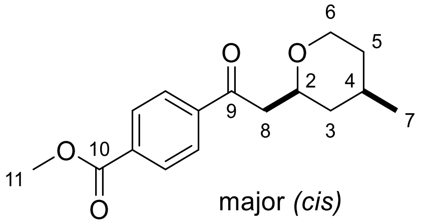

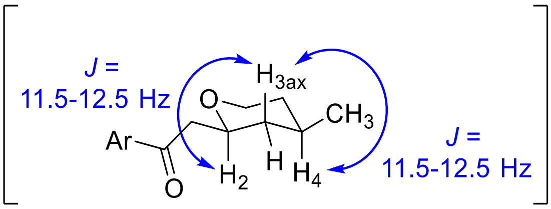

Phosphonate **S26** (440 mg, 1.4 equiv., 1.40 mmol), dry toluene (5 mL) 60% wt. NaH (48 mg, 1.2 eq., 1.2 mmol) and lactol **1d** (116 mg, 1 equiv., 1.00 mmol) were subjected to **General Procedure 3**. The title compound was afforded after purification by FCC (20% Et_2O/pentane) as a white solid (220 mg, 0.796 mmol, 80% yield, 92:8 *cis*/*trans*).

^{1}H NMR (400 MHz, $CDCl_3$) δ 8.14 – 8.09 (m, 2H, ArCH x 2), 8.03 – 7.97 (m, 2H, ArCH x 2), 4.01 – 3.87 (m, 5H, H_2, H_{6eq}, H_{11} x 3), 3.44 (ddd, *J* = 12.5, 11.5, 2.5 Hz, 1H, H_{6ax}), 3.30 (dd, *J* = 16.0, 7.0 Hz, 1H, H_{8a}), 2.92 (dd, *J* = 16.0, 5.5 Hz, 1H, H_{8b}), 1.80 – 1.46 (m, 3H, H_{3eq}, H_4, H_{5eq}), 1.28 – 1.18 (m, 1H, H_{5ax}), 1.01 (dt, *J* = 12.5, 11.5 Hz, 1H, H_{3ax}), 0.94 (d, *J* = 6.5 Hz, 3H, H_7 x 3). The minor diastereoisomer displays diagnostic signals at δ = 4.33 – 4.23 (m, 1H, H_2), 3.74 – 3.67 (m, 2H, H_6 x 2), 2.13 – 2.01 (m, 1H, H_4), 1.10 (d, *J* = 7.0 Hz, 3H, H_7 x 3).

^{13}C NMR (101 MHz, $CDCl_3$) δ 198.2, 166.4, 140.7, 134.0, 129.9, 128.3, 74.1, 68.4, 52.6, 45.8, 40.7, 34.5, 30.3, 22.4. The minor diastereoisomer displays diagnostic signals at δ = 63.0, 37.7, 18.6.

HRMS (ESI+): Found $[M+Na]^+$ = 299.1254; $C_{16}H_{20}O_4Na$ requires 299.1254, Δ 0.16 ppm

IR (film) ν_{max}/cm^{-1} 2981, 2844, 1726, 1689, 1437, 1378, 1279, 1108, 975, 763.

m.p.: 67-68 ^{o}C

1-(3-Methoxyphenyl)-2-(4-methyltetrahydro-2*H*-pyran-2-yl)ethan-1-one, 4i

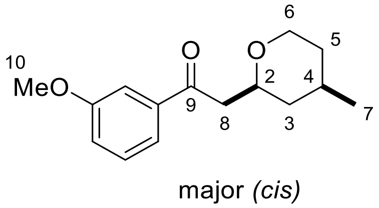

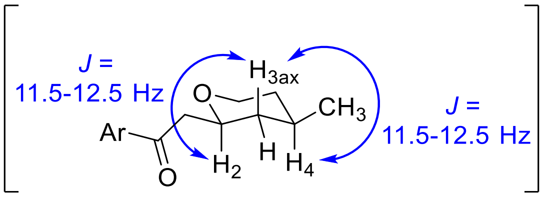

Phosphonate **S27** (400 mg, 1.4 equiv., 1.40 mmol), dry toluene (5 mL) 60% wt. NaH (48 mg, 1.2 eq., 1.2 mmol) and lactol **1d** (116 mg, 1 equiv., 1.00 mmol) were subjected to **General**

Procedure 9. The title compound was afforded after purification by FCC (10% Et_2O/pentane) as a colourless oil (235 mg, 0.946 mmol, 95% yield, 85:15 *cis*/*trans*).

^{1}H NMR (400 MHz, $CDCl_3$) δ 7.57 – 7.46 (m, 4H, major ArCH x 2, minor ArCH x 2), 7.36 (t, *J* = 8.0 Hz, 2H, major ArCH, minor ArCH), 7.10 (ddd, *J* = 8.0, 2.5, 1.0 Hz, 2H, major ArCH, minor ArCH), 4.29 (dtd, *J* = 9.5, 6.5, 3.0 Hz, 1H, minor H_2), 4.01 – 3.89 (m, 2H, major H_2, major $H6_{eq}$), 3.87 – 3.82 (m, 6H, major H_{10} x 3, minor H_{10} x 3), 3.76 – 3.69 (m, 2H, minor H_6 x 2), 3.45 (ddd, *J* = 12.5, 11.5, 2.0 Hz, 1H, major H_{6ax}), 3.34 – 3.22 (m, 2H, major H_{8a}, minor H_{8a}), 2.99 – 2.85 (m, 2H, major H_{8b}, minor H_{8b}), 2.06 (qp, *J* = 7.0, 4.5 Hz, 1H, minor H_4), 1.88 – 1.58 (m, 4H, major H_{3eq}, major H_4, minor H_{3eq}, minor H_{5eq}), 1.58 – 1.46 (m, 2H, major H_{5eq}, minor H_{3ax}), 1.31 – 1.15 (m, 2H, major H_{5ax}, minor H_{5ax}), 1.10 (d, *J* = 7.0 Hz, 3H, minor H_7 x 3), 0.99, (dt, *J* = 12.5, 11.5 Hz, 1H, major H_{3ax}), 0.94 (d, *J* = 6.5 Hz, 3H, major H_7 x 3).

δ ^{13}C NMR (101 MHz, $CDCl_3$) δ 198.3, 159.9, 138.8, 129.7, 121.1, 119.8, 112.5, 74.1, 68.4, 55.6, 45.6, 40.7, 34.6, 30.3, 22.4. The minor diastereoisomer displays diagnostic signals at δ = 198.4, 121.1, 69.1, 62.9, 44.4, 37.7, 32.2, 25.1, 18.7.

HRMS (ESI+): Found $[M+H]^+$ = 249.1486; $C_{15}H_{21}O_3$ requires 249.1485, Δ 0.40 ppm

IR (film) v_{max}/cm^{-1} 3003, 2837, 1683, 1597, 1430, 1262, 1090, 826, 686.

1-([1,1'-Biphenyl]-2-yl)-2-(4-methyltetrahydro-2*H*-pyran-2-yl)ethan-1-one, 4j

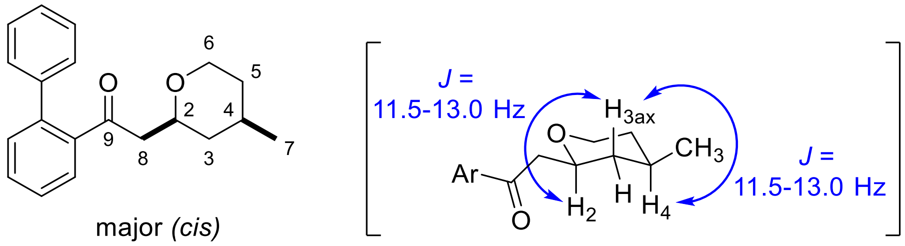

Phosphonate **S28** (465 mg, 1.4 equiv., 1.40 mmol), dry toluene (5 mL) 60% wt. NaH (48 mg, 1.2 eq., 1.2 mmol) and lactol **1d** (116 mg, 1 equiv., 1.00 mmol) were subjected to **General Procedure 3**. The title compound was afforded after purification by FCC (5-10% Et_2O/pentane) as a colourless oil (303 mg, 1.03 mmol, quant, 74:26 *cis*/*trans*).

^{1}H NMR (400 MHz, $CDCl_3$) δ 7.56 – 7.45 (m, 4H, major ArCH x 2, minor ArCH x 2), 7.45 – 7.29 (m, 14H, minor ArCH x 7, minor ArCH x 7), 3.99 (tdd, *J* = 8.0, 5.5, 3.5 Hz, 1H, minor H_2), 3.85 (ddd, *J* = 11.5, 4.5, 1.5 Hz, 1H, major H_{6eq}), 3.60 (dddd, *J* = 11.0, 7.5, 5.5, 2.0 Hz, 1H, major H_2), 3.55 – 3.44 (m, 2H, minor H_6 x 2), 3.29 (ddd, *J* = 12.5, 11.5, 2.0 Hz, 1H, major H_{6ax}), 2.70 –

2.49 (m, 2H, major H_{8a}, minor H_{8a}), 2.34 – 2.22 (m, 2H, major H_{8b}, minor H_{8b}), 1.83 – 1.71 (m, 1H, minor H_4), 1.69 – 1.39 (m, 4H, major H_{3eq}, major H_4, major H_{5eq}, minor H_{5eq}), 1.33 – 1.01 (m, 4H, major H_{5ax}, minor H_3 x 2, minor H_{5ax}), 0.96 (d, J = 7.0 Hz, 3H, minor H_7 x 3), 0.85 (d, J = 6.5 Hz, 3H, major H_7 x 3), 0.66 (dt, J = 13.0, 11.5 Hz, 1H, major H_{3ax}).

^{13}C NMR (101 MHz, $CDCl_3$) δ 205.8, 141.3, 140.7, 140.2, 130.6, 130.3, 129.1, 128.8, 127.9, 127.9, 127.6, 74.4, 68.1, 49.7, 40.3, 34.4, 30.2, 22.3. The minor diastereoisomer displays diagnostic signals at δ = 130.2, 128.0, 127.6, 69.4, 62.5, 48.1, 37.3, 32.3, 24.9, 19.1.

HRMS (ESI+): Found $[M+H]^+$ = 295.1695; $C_{20}H_{23}O_2$ requires 295.1693, Δ 0.91 ppm

IR (film) ν_{max}/cm^{-1} 2979, 1687, 1381, 1257, 1177, 1089, 744, 701

4-Methyltetrahydro-2*H*-pyran-2-yl)-1-(naphthalen-2-yl)ethan-1-one, 4k

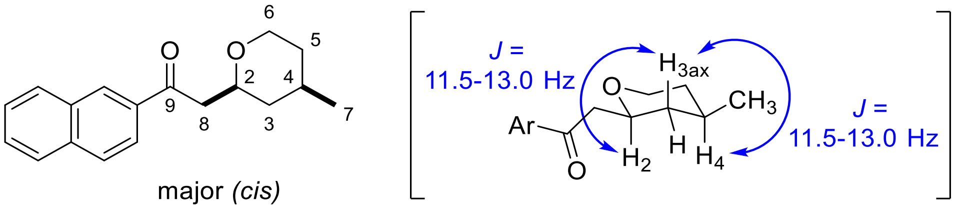

Phosphonate **S29** (428 mg, 1.4 equiv., 1.40 mmol), dry toluene (5 mL) 60% wt. NaH (48 mg, 1.2 eq., 1.2 mmol) and lactol **1d** (116 mg, 1 equiv., 1.00 mmol) were subjected to **General Procedure 3**. The title compound was afforded after purification by FCC (5-10% Et_2O/pentane) as a colourless oil (268 mg, 0.999 mmol, 100% yield, 83:17 *cis*/*trans*).

^{1}H NMR (400 MHz, $CDCl_3$) δ 8.48 (d, J = 2.0 Hz, 2H, major ArCH, minor ArCH), 8.04 (dd, J = 8.5, 2.0 Hz, 2H, major ArCH, minor ArCH), 8.00 – 7.93 (m, 2H, major ArCH, minor ArCH), 7.93 – 7.84 (m, 4H, major ArCH x 2, minor ArCH x 2), 7.66 – 7.47 (m, 4H, major ArCH x 2, minor ArCH x 2), 4.37 (dtd, J = 9.5, 6.5, 3.0 Hz, 1H, minor H_2), 4.09 – 3.92 (m, 2H, major H_2, major H_{6eq}), 3.81 – 3.71 (m, 2H, minor H_6 x 2), 3.54 – 3.39 (m, 3H, major H_{6ax}, major H_{8a}, minor H_{8a}), 3.15 – 2.97 (m,2H, major H_{8b}, minor H_{8b}), 4.44 – 4.30 (m, 1H, minor H_4), 1.88 – 1.77 (m, 2H, major H_{3eq}, minor H_{5eq}), 1.76 – 1.61 (m, 2H, major H_4, minor H_{3eq}), 1.60 – 1.50 (m, 2H, major H_{5eq}, minor H_{3ax}), 1.35 – 1.16 (m, 2H, major H_{5ax}, minor H_{5ax}), 1.12 (d, J = 7.0 Hz, 1H, minor H_7 x 3), 1.05 (dt, J = 13.0, 11.5 Hz, 1H, major H_{3ax}), 0.95 (d, J = 6.5 Hz, 3H, major H_7 x 3).

^{13}C NMR (101 MHz, $CDCl_3$) δ 198.6, 198.5, 135.7, 134.8, 132.7, 130.2, 130.2, 129.8, 129.8, 128.6, 128.5, 128.5, 127.9, 126.9, 124.1, 74.3, 69.2, 68.4, 63.0, 45.5, 44.4, 40.8, 37.8, 34.6,

32.2, 30.3, 25.1, 22.4, 18.7. N.B. The peaks at δ = 135.7, 134.8, 132.7, 128.6, 127.9, 126.9, 124.1 correspond to overlapping signals.

HRMS (ESI+): Found [M+H]+ = 269.1536; $C_{18}H_{21}O_2$ requires 269.1536, Δ -0.16 ppm

IR (film) ν_{max}/cm^{-1} 2998, 2838, 1678, 1469, 1383, 1172, 1088, 817, 744

1-(Furan-2-yl)-2-(4-methyltetrahydro-2*H*-pyran-2-yl)ethan-1-one, 4l

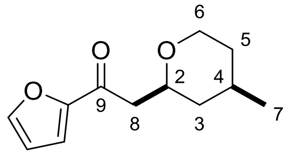

major *(cis)*

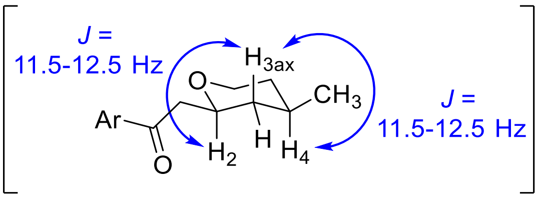

Phosphonate **S30** (344 mg, 1.4 equiv., 1.40 mmol), dry toluene (5 mL) 60% wt. NaH (48 mg, 1.2 eq., 1.2 mmol) and lactol **1d** (116 mg, 1 equiv., 1.00 mmol) were subjected to **General Procedure 3**. The title compound was afforded after purification by FCC (20% Et_2O/pentane) as a white solid (201 mg, 0.965 mmol, 97% yield, 72:28 *cis*/*trans*).

^{1}H NMR (400 MHz, $CDCl_3$) δ 7.58 (dt, *J* = 1.5, 0.5 Hz, 2H, major ArCH, minior ArCH), 7.20 (dt, *J* = 3.5, 0.5 Hz, 2H, major ArCH, minior ArCH), 6.52 (ddd, *J* = 3.5, 2.0, 1.0 Hz, 2H, major ArCH, minior ArCH), 4.27 (tdd, *J* = 8.5, 5.5, 3.0 Hz, 1H, minor H_2), 3.98 – 3.85 (m, 2H, major H_2, major H_{6eq}), 3.77 – 3.63 (m, 2H, minor H_6 x 2), 3.43 (ddd, *J* = 12.5, 11.5, 2.0 Hz, 1H, major H_{6ax}), 3.24 – 3.05 (m, 2H, major H_{8a}, minor H_{8a}), 2.84 – 2.70 (m, 2H, major H_{8b}, minor H_{8b}), 2.05 (qp, *J* = 7.0, 4.5 Hz, 1H, minor H_4), 1.85 – 1.55 (m, 4H, major H_{3eq}, major H_4, minor H_{3eq}, minor H_{5eq}), 1.56 – 1.39 (m, 2H, major H_{5eq}, minor H_{3ax}), 1.32 – 1.13 (m, 2H, major H_{5ax}, minor H_{5ax}), 1.08 (d, *J* = 7.0 Hz, 3H, minor H_7 x 3), 1.00 (dt, *J* = 12.5, 11.5 Hz, 1H, major H_{3ax}), 0.93 (d, *J* = 6.5 Hz, 3H, major H_7 x 3).

^{13}C NMR (101 MHz, $CDCl_3$) δ 187.6, 187.4, 153.2, 146.7, 146.6, 117.8, 117.7, 112.4, 112.4, 74.1, 69.2, 68.3, 62.8, 45.4, 44.2, 40.6, 37.7, 34.5, 32.1, 30.3, 25.0, 22.4, 18.8. *N.B.* The peak at δ = 153.2 ppm corresponds to two overlapping signals.

HRMS (ESI+): Found [M+Na]+ = 231.0992; $C_{12}H_{16}O_3Na$ requires 231.0992, Δ 0.14 ppm

m.p: 70-71 ^{o}C

IR (film) ν_{max}/cm^{-1} 2997, 2886, 1673, 1381, 1265, 1165, 1090, 954

1-(4-(Dimethylamino)phenyl)-2-(4-methyltetrahydro-2H-pyran-2-yl)ethan-1-one, 4m

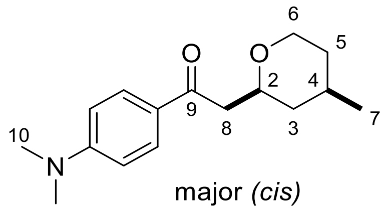

Phosphonate **S31** (418 mg, 1.4 equiv., 1.40 mmol), dry toluene (5 mL) 60% wt. NaH (48 mg, 1.2 eq., 1.2 mmol) and lactol **1d** (116 mg, 1 equiv., 1.00 mmol) were subjected to **General Procedure 3**. The title compound was afforded after purification by FCC (25% Et_2O/pentane) as a white solid (261 mg, 0.999 mmol, 100% yield, 91:9 *cis*/*trans*).

^{1}H NMR (400 MHz, $CDCl_3$) δ 7.96 – 7.83 (m, 2H, ArCH x 2), 6.74 – 6.58 (m, 2H, ArCH x 2), 4.03 – 3.85 (m, 2H, H_2, H_{6eq}), 3.46 (ddd, *J* = 12.5, 11.5, 2.5 Hz, 1H, H_{6ax}), 3.21 (dd, *J* = 15.5, 6.0 Hz, 1H, H_{8a}), 3.05 (s, 6H, H_{10} x 6), 2.85 (dd, *J* = 15.5, 6.5 Hz, 1H, H_{8b}), 1.76 (ddt, *J* = 13.0, 4.0, 2.0 Hz, 1H, H_{3eq}), 1.73 – 1.57 (m, 1H, H_4), 1.52 (ddq, *J* = 13.0, 4.0, 2.0 Hz, 1H, H_{5eq}), 1.27 – 1.14 (m, 1H, H_{5ax}), 1.03 – 0.95 (m, 1H, H_{3ax}), 0.92 (d, *J* = 6.5 Hz, 3H, H_7 x 3). The minor diastereoisomer displays diagnostic signals at δ = 4.28 (dtd, *J* = 9.5, 6.5, 3.0 Hz, 1H, H_2), 3.76 – 3.70 (m, 2H, H_6 x 2), 2.12 – 1.98 (m, 1H, H_4), 1.08 (d, *J* = 7.0 Hz, 3H, H_7 x 3).

^{13}C NMR (101 MHz, $CDCl_3$) δ 196.4, 153.5, 130.7, 125.6, 110.8, 74.7, 68.4, 44.7, 40.8, 40.2, 34.7, 30.3, 22.4. The minor diastereoisomer displays diagnostic signals at δ = 69.5, 62.9, 43.6, 37.8, 32.3, 25.1, 18.8.

HRMS (ESI+): Found $[M+H]^+$ = 262.1803; $C_{16}H_{24}O_2N$ requires 262.1802, Δ 0.52 ppm

IR (film) ν_{max}/cm^{-1} 2949, 2921, 2839, 1659, 1598, 1370, 1189, 1167, 1089, 818.

m.p.: 59-60 °C

4-Methyltetrahydro-2*H*-pyran-2-yl)-1-(pyridin-3-yl)ethan-1-one, 4n

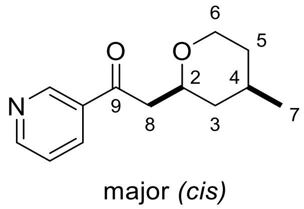

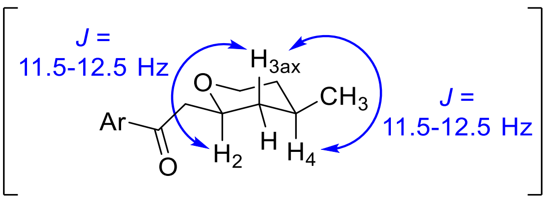

Phosphonate **S32** (360 mg, 1.4 equiv., 1.40 mmol), dry toluene (5 mL) 60% wt. NaH (48 mg, 1.2 eq., 1.2 mmol) and lactol **1d** (116 mg, 1 equiv., 1.00 mmol) were subjected to **General Procedure 3**. The title compound was afforded after purification by FCC (70% Et_2O/pentane) as a colourless oil (215 mg, 0.980 mmol, 98% yield, 74:26 *cis*/*trans*).

^1H NMR (400 MHz, $CDCl_3$) δ 9.27 – 9.12 (m, 2H, major ArCH, minor ArCH), 8.85 – 8.66 (m, 2H, major ArCH, minor ArCH), 8.37 – 8.18 (m, 2H, major ArCH, minor ArCH), 7.52 – 7.33 (m, 2H, major ArCH, minor ArCH), 4.28 (dddd, *J* = 9.9, 7.5, 5.0, 3.0 Hz, 1H, minor H_2), 4.01 – 3.85 (m, 2H, major H_2, major H_{6eq}), 3.75 – 3.64 (m, 2H, minor H_6 x 2), 3.50 – 3.37 (m, 1H, major H_{6ax}), 3.35 – 3.23 (m, 2H, major H_{8a}, minor H_{8a}), 2.95 – 2.84 (m, 2H, major H_{8b}, minor H_{8b}), 2.16 – 1.92 (m, 1H, minor H_4), 1.87 – 1.58 (m, 4H, major H_{3eq}, major H_4, minor H_{3eq}, minor H_{5eq}), 1.58 – 1.45 (m, 2H, major H_{5eq}, minor H_{3ax}), 1.31 – 1.14 (m, 2H, major H_{5ax}, minor H_{5ax}), 1.10 (d, *J* = 7.0 Hz, 3H, minor H_7 x 3), 1.02 (dt, *J* = 12.5, 11.5 Hz, 1H, major H_{3ax}), 0.94 (d, *J* = 6.5 Hz, 3H, major H_7 x 3).

^{13}C NMR (101 MHz, $CDCl_3$) δ 197.7, 197.6, 153.5, 150.0, 149.9, 135.8, 132.7, 123.7, 74.0, 69.0, 68.3, 63.0, 45.7, 44.8, 40.7, 37.7, 34.5, 31.9, 30.3, 25.1, 22.4, 18.5. *N.B.* The peaks at δ = 153.5, 135.8, 132.7 and 123.7 correspond to overlapping signals.

HRMS (ESI+): Found $[M+H]^+$ = 220.1332; $C_{13}H_{18}O_2N$ requires 220.1332, Δ -0.19 ppm

IR (film) ν_{max}/cm^{-1} 2999, 2886, 1689, 1585, 1380, 1259, 1089, 971, 703.

4-Methyltetrahydro-2*H*-pyran-2-yl)-1-(quinolin-6-yl)ethan-1-one, 4o

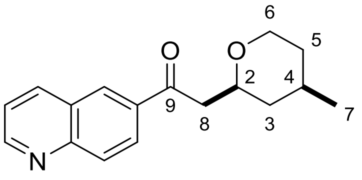

major *(cis)*

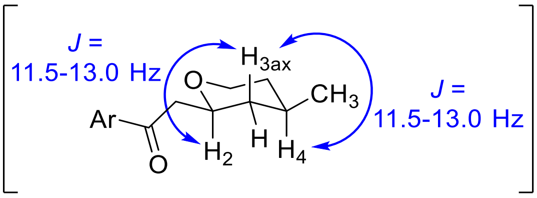

Phosphonate **S33** (430 mg, 1.4 equiv., 1.40 mmol), dry toluene (5 mL) 60% wt. NaH (48 mg, 1.2 eq., 1.2 mmol) and lactol **1d** (116 mg, 1.00 equiv., 1 mmol) were subjected to **General Procedure 3**. The title compound was afforded after purification by FCC (80% Et_2O/pentane) as a colourless oil (241 mg, 0.895 mmol, 90% yield, 64:36 *cis*/*trans*).

^1H NMR (400 MHz, $CDCl_3$) δ 9.00 (dd, *J* = 4.5, 1.5 Hz, 2H, major ArCH, minor ArCH), 8.46 (d, *J* = 2.0 Hz, 2H, major ArCH, minor ArCH), 8.34 – 8.23 (m, 4H, major ArCH x 2, minor ArCH x 2), 8.16 (dt, *J* = 9.0, 1.0 Hz, 2H, major ArCH, minor ArCH), 7.48 (dd, *J* = 8.5, 4.0 Hz, 2H, major ArCH, minor ArCH), 4.34 (dddd, *J* = 9.0, 7.0, 5.5, 3.0 Hz, 1H, minor H_2), 4.04 – 3.91 (m, 2H, major H_2 major H_{6eq}), 3.78 – 3.68 (m, 2H, minor H_6 x 2), 3.54 – 3.37 (m, 3H, major H_{6ax}, major H_{8a}, minor H_{8a}), 3.13 – 2.94 (m, 2H, major H_{8b}, minor H_{8b}), 2.17 – 2.02 (m, 1H, minor H_4), 1.90 – 1.75 (m, 2H, major H_{3eq}, minor H_{5ax}), 1.75 – 1.61 (m, 2H, major H_4, minor H_{3eq}), 1.60 – 1.49 (m, 2H,

major H_{5eq}, minor H_{3ax}), 1.34 – 1.15 (m, 2H, major H_{5ax}, minor H_{5ax}), 1.12 (d, *J* = 7.2 Hz, 3H, minor H_7 x 3), 1.05 (dt, *J* = 13.0, 11.5 Hz, 1H, major H_{3ax}), 0.95 (d, *J* = 6.5 Hz, 3H, major H_7 x 3).

^{13}C NMR (101 MHz, $CDCl_3$) δ 198.1, 198.0, 152.5, 150.0, 138.0, 135.4, 130.0, 130.0, 129.9, 129.9, 128.1, 128.1, 127.7, 122.0, 74.3, 69.2, 68.4, 63.0, 45.6, 44.6, 40.8, 37.8, 34.5, 32.1, 30.3, 25.1, 22.4, 18.6. N.B. The peaks at δ = 152.5, 150.0, 138.0, 135.4, 127.7 and 122.0 ppm correspond to overlapping signals.

HRMS (ESI+): Found $[M+H]^+$ = 270.1488; $C_{17}H_{20}O_2N$ requires 270.1489, Δ -0.08 ppm

IR (film) ν_{max}/cm^{-1} 2952, 2925, 1673, 1459, 1168, 1089, 839

1-(3-Chlorophenyl)-2-(4-methyltetrahydro-2*H*-pyran-2-yl)ethan-1-one, 4p

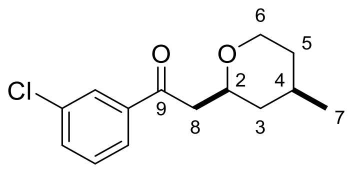

Phosphonate **S34** (407 mg, 1.4 equiv., 1.40 mmol), dry toluene (5 mL) 60% wt. NaH (48 mg, 1.2 eq., 1.2 mmol) and lactol **1d** (116 mg, 1 equiv., 1.00 mmol) were subjected to **General Procedure 3**. The title compound was afforded after purification by FCC (4-10% Et_2O/pentane) as a colourless oil (215 mg, 0.851 mmol, 85% yield, 94:6 *cis*/*trans*).

^{1}H NMR (400 MHz, $CDCl_3$) δ 7.93 (t, *J* = 2.0 Hz, 1H, ArCH), 7.83 (ddd, *J* = 8.0, 1.5, 1.0 Hz, 1H, ArCH), 7.52 (ddd, *J* = 8.0, 2.0, 1.0 Hz, 1H, ArCH), 7.40 (t, *J* = 8.0 Hz, 1H, ArCH), 3.99 – 3.87 (m, 2H, H_2, H_{6eq}), 3.44 (ddd, *J* = 12.5, 11.5, 2.0 Hz, 1H, H_{6ax}), 3.25 (dd, *J* = 16.0, 7.0 Hz, 1H, H_{8a}), 2.87 (dd, *J* = 16.0, 5.5 Hz, 1H, H_{8b}), 1.80 – 1.50 (m, 3H, H_{3eq}, H_4, H_{5eq}), 1.27 – 1.14 (m, 1H, H_{5ax}), 1.05 – 0.95 (m, 1H, H_{3ax}), 0.94 (d, *J* = 6.5 Hz, 3H, H_7 x 3). The minor diastereoisomer displays diagnostic signals at δ = 4.31 – 4.24 (m, 1H, H_2), 3.74 – 3.68 (m, 2H, H_6 x 2), 2.11 – 2.02 (m, 1H, H_4), 1.10 (d, *J* = 7.1 Hz, 3H, H_7 x 3).

^{13}C NMR (101 MHz, $CDCl_3$) δ 197.4, 139.2, 135.1, 133.1, 130.0, 128.5, 126.5, 74.1, 68.4, 45.5, 40.7, 34.5, 30.3, 22.4.

HRMS (ESI+): Found $[M+H]^+$ = 253.0990; $C_{14}H_{18}O_2Cl$ requires 253.0990, Δ 0.12 ppm.

IR (film) ν_{max}/cm^{-1} 2951, 2841, 1689, 1572, 1423, 1206, 1090, 785, 681.

1-(3-Iodophenyl)-2-(4-methyltetrahydro-2*H*-pyran-2-yl)ethan-1-one, 4q

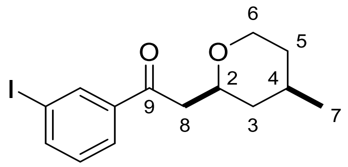

major *(cis)*

Phosphonate **S35** (535 mg, 1.4 equiv., 1.40 mmol), dry toluene (5 mL), 1M LDA in THF (1.2 mL, 1.2 eq., 1.2 mmol) and lactol **1d** (116 mg, 1 equiv., 1.00 mmol) were subjected to **General Procedure 3**. The title compound was afforded after purification by FCC (4-10% Et_2O/pentane) as a white solid (282 mg, 0.819 mmol, 82% yield, 91:9 *cis*/*trans*).

^{1}H NMR (400 MHz, $CDCl_3$) δ 8.28 (t, *J* = 1.5 Hz, 1H, ArCH), 7.95 – 7.83 (m, 2H, ArCH x 2), 7.19 (t, *J* = 8.0 Hz, 1H, ArCH), 4.01 – 3.87 (m, 2H, H_2, H_{6eq}), 3.44 (ddd, *J* = 12.5, 11.5, 2.5 Hz, 1H, H_{6ax}), 3.24 (dd, *J* = 16.0, 7.0 Hz, 1H, H_{8a}), 2.86 (dd, *J* = 16.0, 5.5 Hz, 1H, H_{8b}), 1.79 – 1.47 (m, 3H, H_{3eq}, H_4, H_{5eq}), 1.27 – 1.13 (m, 1H, H_{5ax}), 1.06 – 0.96 (m, 1H, H_{3ax}), 0.94 (d, *J* = 6.5 Hz, 3H, H_7 x 3). The minor diastereoisomer displays diagnostic signals at δ = 4.26 (dddd, *J* = 9.0, 7.0, 5.5, 3.0 Hz, 1H, H_2), 3.74 – 3.67 (m, 2H, H_6 x 2), 2.12 – 1.99 (m, 1H, H_4), 1.10 (d, *J* = 7.0 Hz, 3H, H_7 x 3).

^{13}C NMR (101 MHz, $CDCl_3$) δ 197.2, 141.9, 139.2, 137.4, 130.4, 127.6, 94.6, 74.0, 68.3, 45.4, 40.7, 34.5, 30.3, 22.4. The minor diastereoisomer displays diagnostic signals at δ = 69.0, 62.9, 44.3, 37.7, 32.1, 25.1, 18.7

HRMS (ESI+): Found $[M+H]^+$ = 345.0345; $C_{14}H_{18}O_2I$ requires 345.0346, Δ -0.42 ppm

IR (film) ν_{max}/cm^{-1} 2997, 2886, 1394, 1252, 1151, 1071, 954.

m.p.: 44-45 °C

2-(4-Methyltetrahydro-2*H*-pyran-2-yl)-1-(4-(4,4,5,5-tetramethyl-1,3,2-dioxaborolan-2-yl)phenyl)ethan-1-one, 4r

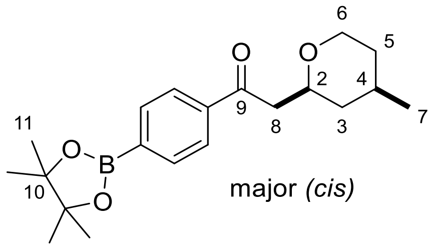

major *(cis)*

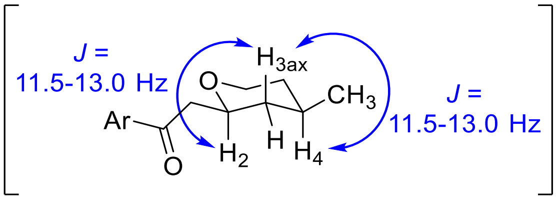

Phosphonate **S36** (535 mg, 1.4 equiv., 1.40 mmol), dry toluene (5 mL) 60% wt. NaH (48 mg, 1.2 eq., 1.2 mmol) and lactol **1d** (116 mg, 1 equiv., 1.00 mmol) were subjected to **General**

Procedure 9. The title compound was afforded after purification by FCC (15% Et_2O/pentane) as a white solid (325 mg, 0.944 mmol, 94% yield, 92:8 *cis*/*trans*).

^{1}H NMR (400 MHz, $CDCl_3$) δ 8.00 – 7.83 (m, 4H, ArCH x 4), 4.01 – 3.87 (m, 2H, H_2, H_{6eq}), 3.53 – 3.41 (m, 1H, H_{6ax}), 3.29 (dd, *J* = 16.0, 6.5 Hz, 1H, H_{8a}), 2.94 (dd, *J* = 16.0, 6.0 Hz, 1H, H_{8b}), 1.81 – 1.59 (m, 2H, H_{3eq}, H_4), 1.53 (ddq, *J* = 13.5, 4.0, 2.0 Hz, 1H, H_{5eq}), 1.35 (s, 12H, H_{11} x 12), 1.27 – 1.18 (m, 1H, H_{5ax}), 0.99 (dt, *J* = 13.0, 11.5 Hz, 1H, H_{3ax}), 0.93 (d, *J* = 6.5 Hz, 3H, H_7 x 3). The minor diastereoisomer displays diagnostic signals at δ = 4.29 (dtd, *J* = 9.5, 6.5, 3.0 Hz, 1H, H_2), 3.75 – 3.69 (m, 2H, H_6 x 2), 2.12 – 2.00 (m, 1H, H_4), 1.09 (d, *J* = 7.0 Hz, 3H, H_7 x 3).

^{13}C NMR (101 MHz, $CDCl_3$) δ 198.8, 139.3 , 135.0, 127.4, 84.3, 74.1, 68.3, 45.6, 40.7, 34.6, 30.3, 25.0, 22.4. n.b. ArC attached to boron was not observed due to quadrupolar relaxation.

HRMS (ESI+): Found $[M+H]^+$ = 345.2230; $C_{20}H_{30}O_4B$ requires 345.2232, Δ -0.51 ppm

IR (film) ν_{max}/cm^{-1} 2951, 2873, 2840, 1685, 1509, 1360, 1140, 1090, 858.

m.p.: 73-74 °C

2-(4-Isobutyltetrahydro-2*H*-pyran-2-yl)-1-ferrocene ethan-1-one, 4s

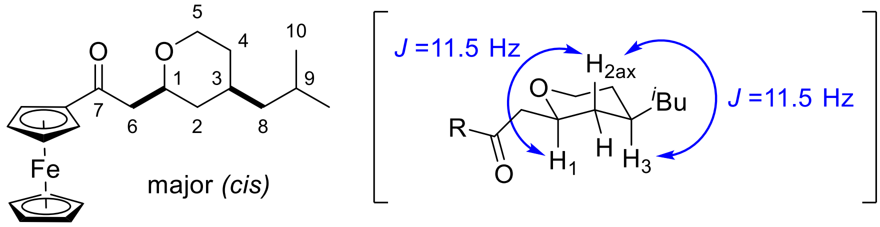

Phosphonate **S38** (509 mg, 1.4 equiv., 1.40 mmol), dry toluene (5 mL) 60% wt. NaH (48 mg, 1.2 eq., 1.2 mmol) and lactol **S2** (116 mg, 1 equiv., 1.00 mmol) were subjected to **General Procedure 3**. The title compound was afforded after purification by FCC (25% Et_2O/pentane) as a white solid (387 mg, 1.05 mmol, quant, > 95:5 *cis*/*trans*).

^{1}H NMR (400 MHz, $CDCl_3$) δ 4.83 – 4.78 (m, 1H, ArCH), 4.78 – 4.74 (m, 1H, ArCH), 4.53 – 4.46 (m, 2H, ArCH x 2), 4.22 (s, 5H, ArCH x 5), 4.05 – 3.87 (m, 2H, H_1, H_{5eq}), 3.54 – 3.42 (m, 1H, H_{5ax}), 3.02 (dd, *J* = 16.0, 6.5 Hz, 1H, H_{6a}), 2.67 (dd, *J* = 16.0, 6.0 Hz, 1H, H_{6b}), 1.85 – 1.49 (m, 4H, H_{2eq}, H_3, H_{4eq}, H_9), 1.30 – 1.14 (m, 1H, H_{4ax}), 1.14 – 1.05 (m, 2H, H_8 x 2), 0.94 (q, *J* = 11.5 Hz, 1H, H_{2ax}), 0.89 – 0.85 (m, 6H, H_{10} x 6)

^{13}C NMR (101 MHz, $CDCl_3$) δ 202.3, 79.6, 74.0, 72.5, 72.4, 70.0, 69.6, 69.5, 68.3, 46.7, 39.3, 33.1, 32.8, 24.4, 23.0, 22.9. N.B. The peak at δ = 46.7 ppm corresponds to two overlapping signals.

HRMS (ESI+): Found $[M+H]^+$ = 369.1506; $C_{21}H_{29}O_2Fe$ requires 369.1511, Δ -0.50 ppm

IR (film) $ν_{max}/cm^{-1}$ 2924, 2838, 1670, 1454, 1094, 1026, 820.

1-Cyclohexyl-2-(4-methyltetrahydro-2*H*-pyran-2-yl)ethan-1-one, 4t

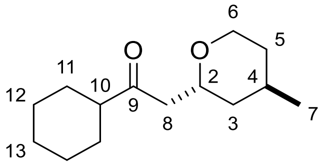

major *(trans)*

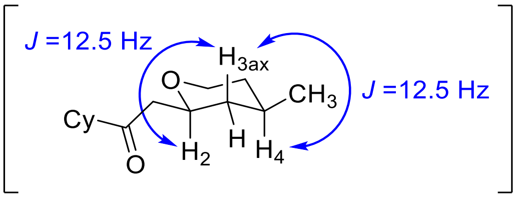

Phosphonate **S39** (550 mg, 1.4 equiv., 2.10 mmol), dry toluene (7.5 mL) 60% wt. NaH (72 mg, 1.2 eq., 1.8 mmol) and lactol **1d** (174 mg, 1 equiv., 1.50 mmol) were subjected to **General Procedure 3**. The title compound was afforded after purification by FCC (5-10% Et_2O/pentane) as a colourless oil (284 mg, 1.23 mmol, 85% yield, 29:71 *cis*/*trans*).

^{1}H NMR (400 MHz, $CDCl_3$) δ 4.18 – 4.05 (m, 1H, major H_2), 3.92 (ddd, *J* = 11.5, 4.5, 1.5 Hz, 1H, minor H_{6eq}), 3.75 (dddd, *J* = 11.0, 7.0, 5.5, 2.0 Hz, 1H, minor H_2), 3.71 – 3.65 (m, 2H, major H_6 x 2), 3.41 (ddd, *J* = 12.5, 11.5, 2.5 Hz, 1H, minor H_{6ax}), 2.73 (d, *J* = 16.0, 7.5 Hz, 1H, major H_{8a}), 2.71 (d, *J* = 16.0, 7.0 Hz, 1H, minor H_{8a}), 2.47 – 2.29 (m, 4H, major H_{8b}, major H_{10}, minor H_{8b}, minor H_{10}), 2.01 (qp, *J* = 7.0, 4.5 Hz, 1H, major H_4), 1.91 – 1.71 (m, 9H, major H_{5eq}, major H_{11a} x 2 major H_{12a} x 2, minor H_{11a} x 2, minor H_{12a} x 2), 1.69 – 1.59 (m, 4H, major H_{13a}, minor H_{3eq}, minor H_4, minor H_{13a}), 1.55 – 1.46 (m, 2H, major H_{3eq}, minor H_{5eq}), 1.44 – 1.35 (m, 1H, major H_{3ax}), 1.35 – 1.11 (m, 12H, major H_{5ax}, major H_{11b} x 2, major H_{12b} x 2, major H_{13b}, minor H_{5ax}, minor H_{11b} x 2, minor H_{12b} x 2, minor H_{13b}), 1.07 (d, *J* = 7.0 Hz, 3H, major H_7 x 3), 0.91 (d, *J* = 6.5 Hz, 3H, minor H_7 x 3) 0.88, (q, *J* = 12.5 Hz, 1H minor H_{3ax}).

^{13}C NMR (101 MHz, $CDCl_3$) δ 212.5, 68.8, 62.8, 51.5, 46.4, 37.6, 32.1, 28.4, 28.2, 26.0, 25.8, 25.7, 25.1, 18.7. The minor diastereoisomer displays diagnostic signals at δ = 212.4, 73.9, 68.3, 51.6, 47.5, 40.6, 34.6, 30.3, 28.2, 22.4.

HRMS (ESI+): Found $[M+H]^+$ = 225.1850; $C_{14}H_{25}O_2$ requires 225.1849, Δ 0.34 ppm

IR (film) $ν_{max}/cm^{-1}$ 2927, 2853, 1708, 1449, 1315, 1080, 1003

1-(8-Oxaspiro[4.5]decan-7-yl)propan-2-one, 4u

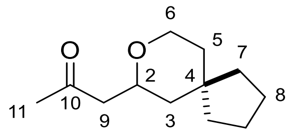

Diethyl (2-oxopropyl)phosphonate (272 mg, 1.4 equiv., 1.40 mmol), dry toluene (5 mL) 60% wt. NaH (48 mg, 1.2 eq., 1.2 mmol) and lactol **S3** (156 mg, 1 equiv., 1.00 mmol) were subjected to **General Procedure 3**. The title compound was afforded after purification by FCC (20% Et_2O/pentane) as a colourless oil (196 mg, 0.999 mmol, 100% yield).

^{1}H NMR (400 MHz, $CDCl_3$) δ 3.89 – 3.77 (m, 2H, H_2, H_{6eq}), 3.52 (ddd, *J* = 12.5, 11.5, 2.0 Hz, 1H, H_{6ax}), 2.64 (dd, *J* = 15.5, 8.0 Hz, 1H, H_{9a}), 2.36 (dd, *J* = 15.5, 4.5 Hz, 1H, H_{9b}), 2.17 (s, 3H, H_{11} x 3), 1.71 – 1.47 (m, 7H, H_{5eq}, H_8 x 4, H_{7a} x 2), 1.44 – 1.20 (m, 5H, H_3 x 2, H_{5ax}, H_{7b} x 2).

13C NMR (101 MHz, CDCl3) δ 207.7, 71.5, 65.8, 50.5, 43.9, 42.6, 41.1, 37.6, 34.5, 31.2, 24.9, 23.7.

HRMS (ESI+): Found $[M+H]^+$ = 197.1539; $C_{12}H_{20}O_2$ requires 197.1536, Δ 1.57 ppm

IR (film) ν_{max}/cm^{-1} 2998, 2867, 1715, 1381, 1357, 1253, 1167, 1102, 954.

1-(4,4-Dimethyltetrahydro-2*H*-pyran-2-yl)-4,8-dimethylnon-7-en-2-one, 4v

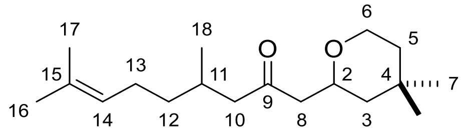

Phosphonate **S41** (426 mg, 1.4 equiv., 1.40 mmol), dry toluene (5 mL) 60% wt. NaH (48 mg, 1.2 eq., 1.2 mmol) and lactol **S19** (130 mg, 1 equiv., 1.00 mmol) were subjected to **General Procedure 3**. The title compound was afforded after purification by FCC (5-10% Et_2O/pentane) as a colourless oil (280 mg, 0.998 mmol, 100% yield, 1:1 d.r.).

^{1}H NMR (400 MHz, $CDCl_3$) δ 5.08 (dd hept, *J* = 8.5, 5.5, 1.5 Hz, 1H, H_{14}), 3.95 – 3.88 (m, 1H, H_2), 3.77 (ddd, *J* = 12.0, 5.0, 1.5 Hz, 1H, H_{6eq}), 3.58 (ddd, *J* = 13.0, 12.0, 2.5 Hz, 1H, H_{6ax}), 2.61 (dd, *J* = 15.5, 7.5 Hz, 1H, H_{8a}), 2.46 – 2.38 (m, 1H, H_{10a}), 2.34 – 2.18 (m, 2H, H_{8b}, H_{10b}), 2.07 – 1.87 (m, 3H, H_{11}, H_{13} x 2), 1.67 (s, 3H, H_{16} x 3, or H_{17} x 3), 1.59 (s, 3H, H_{16} x 3, or H_{17} x 3), 1.51 – 1.38 (m, 1H, H_{5eq}), 1.35 – 1.08 (m, 5H, H_{5ax}, H_3 x 2, H_{12} x 2), 1.02 (s, 3H, H_{7a} x 3), 0.93 (s, 3H, H_{7b} x 3), 0.88 (d, *J* = 6.5 Hz, 3H, H_{18}).

^{13}C NMR (101 MHz, $CDCl_3$) δ 209.5, 209.4, 131.6, 124.6, 70.1, 70.0, 64.5, 51.5, 50.1, 50.1, 45.0, 38.7, 37.1 , 37.1, 33.3, 28.8, 28.8, 25.8, 25.6, 25.6, 24.1, 19.9, 19.9, 17.8. Several peaks for the two diastereoisomers overlapped.

HRMS (ESI+): Found $[M+H]^+$ = 281.2475; $C_{13}H_{17}O_2$ requires 281.2474, Δ 0.81 ppm

IR (film) ν_{max}/cm^{-1} 2972, 2900, 1714, 1469, 1364, 1190, 1084.

***tert*-Butyl 4-(2-(4-methyltetrahydro-2*H*-pyran-2-yl)acetyl)piperidine-1-carboxylate, 4w**

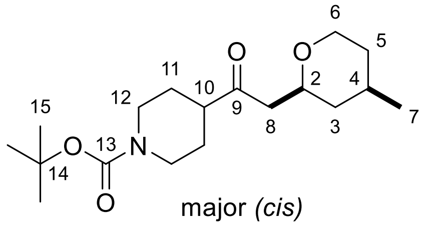

Phosphonate **S42** (509 mg, 1.4 equiv., 1.40 mmol), dry toluene (5 mL) 60% wt. NaH (48 mg, 1.2 eq., 1.2 mmol) and lactol **1d** (116 mg, 1 equiv., 1.00 mmol) were subjected to **General Procedure 3**. The title compound was afforded after purification by FCC (50% Et_2O/pentane) as a white solid (325 mg, 0.999 mmol, 100% yield, 88:12 *cis*/*trans*).

^1H NMR (400 MHz, $CDCl_3$) δ 4.17 – 4.01 (m, 2H, H_{12a} x 2), 3.91 (ddd, *J* = 11.5, 4.5, 1.5 Hz, 1H, H_{6eq}), 3.81 – 3.70 (m, 1H, H_2), 3.40 (ddd, *J* = 12.5, 11.5, 2.0 Hz, 1H, H_{6ax}), 2.86 – 2.62 (m, 3H, H_{12b} x 2, H_{8a}), 2.48 (tt, *J* = 11.5, 3.5 Hz, 1H, H_{10}), 2.40 (dd, *J* = 16.0, 5.0 Hz, 1H, H_{8b}), 1.86 – 1.76 (m, 2H, H_{11a} x 2), 1.70 – 1.40 (m, 14H, H_{3eq}, H_4, H_{5eq}, H_{11b} x 2, H_{15} x 9), 1.25 – 1.11 (m, 1H, H_{5ax}), 0.96 – 0.83 (m, 4H, H_7 x 3, H_{3ax}). The minor diastereoisomer displays diagnostic signals at δ = 3.69 – 3.63 (m, 2H, H_6 x 2), 2.08 – 1.96 (m, 1H, H_4), 1.08 (d, *J* = 7.0 Hz, 3H, H_7 x 3)

^{13}C NMR (101 MHz, $CDCl_3$) δ 210.7, 154.8, 79.7, 74.0, 68.3, 49.4, 47.4, 43.4 (br, s), 40.6, 34.5, 30.3, 28.6, 27.4, 27.2, 22.4. *N.B.* broad singlet observed at 43.4 ppm corresponds to both C_{11a} and C_{11b}. The minor diastereoisomer displays diagnostic signals at δ = 62.9, 46.5, 37.6, 32.0, 25.1, 18.5 ppm.

HRMS (ESI+): Found $[M+Na]^+$ = 348.2146; $C_{18}H_{31}O_4NNa$ requires 348.2145, Δ 0.26 ppm

IR (film) ν_{max}/cm^{-1} 2951, 2859, 1693, 1422, 1276, 1165, 1091, 1016.

m.p.: 40-41 ^oC

2-((2*R*,4*R*)-4-(dimethyl(phenyl)silyl)tetrahydro-2*H*-pyran-2-yl)-1-phenylethan-1-one 4x

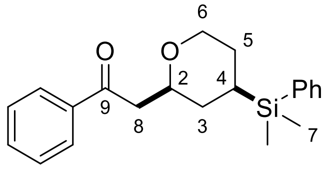

major *(cis)*

Phosphonate **S21** (152 mg, 0.590 mmol, 1.20 eq.), 60% wt. NaH (20 mg, 0.50 mmol, 1.0 eq.), lactol **S5** (116 mg, 0.492 mmol, 1.00 eq.) and dry toluene (2.5 mL) were subjected to **General Procedure 3**. Purification by FCC (5% Et_2O/pentane) afforded the product as a colourless oil (140 mg, 0.413 mmol, 84% yield, >95:5 d.r.).

^{1}H NMR (400 MHz, $CDCl_3$) δ 7.98 – 7.91 (m, 2H, ArCH x 2), 7.59 – 7.52 (m, 1H, ArCH), 7.51 – 7.40 (m, 4H, ArCH x 4), 7.40 – 7.30 (m, 3H, ArCH x 3), 4.01 – 3.86 (m, 2H, H_2, H_{6a}), 3.53 – 3.38 (m, 1H, H_{6b}), 3.25 (dd, *J* = 16.0, 7.0 Hz, 1H, H_{8a}), 2.87 (dd, *J* = 16.0, 5.5 Hz, 1H, H_{8b}), 1.69 (ddd, *J* = 12.0, 2.5 Hz, 2.5 Hz, 1H, H_{3eq}), 1.50 – 1.40 (m, 2H, H_5 x 2), 1.30 – 1.09 (m, 2H, H_{3ax}, H_4), 0.27 (s, 6H, H_7 x 6).

^{13}C NMR (101 MHz, $CDCl_3$) δ 198.5, 137.5, 137.4, 134.1, 133.2, 129.2, 128.7, 128.4, 127.9, 75.3, 69.8, 45.6, 32.8, 26.6, 23.0, -5.3, -5.4.

HRMS (ESI+): Found $[M+H]^+$ = 339.1778; $C_{21}H_{27}O_2Si$ requires 339.1775, Δ 0.92 ppm

IR (film) ν_{max}/cm^{-1} : 2921, 1684, 1255, 1096, 811, 700

1-Phenyl-2-((2*R*,4*R*)-4-(phenylthio)tetrahydro-2*H*-pyran-2-yl)ethan-1-one 4y

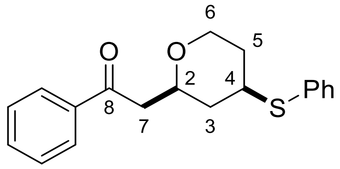

major *(cis)*

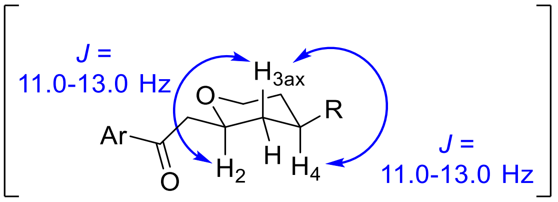

Phosphonate **S21** (2.56 g, 10.0 mmol, 1.40 eq.), 60% wt. NaH (342 mg, 8.56 mmol, 1.20 eq.), lactol **S7** (1.49 g, 7.13 mmol, 1.00 eq.) and dry toluene (35 mL) were subjected to **General Procedure 3**. Purification by FCC (20% Et_2O/pentane) afforded the product as an off-white solid (1.77 g, 5.68 mmol, 80% yield, 53:47 d.r., diastereomers were separable by chromatography).

Data for major **4y**:

^{1}H NMR (400 MHz, $CDCl_3$) δ 7.98 – 7.89 (m, 2H, ArCH x 2), 7.60 – 7.51 (m, 1H, ArCH), 7.50 – 7.37 (m, 4H, ArCH x 4), 7.35 – 7.20 (m, 3H, ArCH x 3), 4.05 – 3.94 (m, 2H, H_2, H_{6a}), 3.53 – 3.42

(m, 1H, H_{6b}), 3.36 – 3.22 (m, 2H, H_4, H_{7a}), 2.90 (dd, *J* = 16.5, 5.5 Hz, 1H, H_{7b}), 2.15 – 2.05 (m, 1H, H_{3eq}), 1.93 – 1.82 (m, 1H, H_{5a}), 1.70 – 1.55 (m, 1H, H_{5b}), 1.42 (ddd, *J* = 13.0, 12.0, 11.0 Hz, 1H, H_{3ax}).

^{13}C NMR (101 MHz, $CDCl_3$) δ 197.9, 137.2, 133.5, 133.3, 133.0, 129.1, 128.7, 128.4, 127.5, 74.1, 68.0, 44.9, 43.6, 39.0, 33.1.

m.p. = 54-56 °C

HRMS (ESI+): Found $[M+H]^+$ 313.1256; $C_{19}H_{21}O_2S$ requires 313.1257, Δ -0.31 ppm

IR (film) $_{vmax/}$cm^{-1}: 1684, 1448, 1212, 1070, 749, 691

<u>Data for minor **4y**:</u>

^{1}H NMR (400 MHz, $CDCl_3$) δ 7.99 – 7.90 (m, 2H, ArCH x 2), 7.61 – 7.51 (m, 1H, ArCH), 7.51 – 7.37 (m, 4H, ArCH x 4), 7.33 – 7.18 (m, 3H, ArCH x 3), 4.57 – 4.46 (m, 1H, H_2), 4.05 – 3.94 (m, 1H, H_{6a}), 3.83 – 3.72 (m, 2H, H_{6b}, H_4), 3.28 (dd, *J* = 16.0, 7.0 Hz, 1H, H_{7a}), 2.89 (dd, *J* = 16.0, 5.5 Hz, 1H, H_{7b}), 2.11 – 1.97 (m, 1H, H_{5a}), 1.96 – 1.78 (m, 2H, H_{3eq}, H_{3ax}), 1.74 – 1.62 (m, 1H, H_{5b}).

^{13}C NMR (101 MHz, $CDCl_3$) δ 197.9, 137.3, 135.0, 133.2, 132.1, 129.2, 128.7, 128.4, 127.2, 69.3, 63.5, 44.9, 42.4, 36.1, 30.3.

m.p. = 54-56 °C

HRMS (ESI+): Found $[M+H]^+$ 313.1256; $C_{19}H_{21}O_2S$ requires 313.1257, Δ -0.31 ppm

IR (film) ν_{max}/cm^{-1}: 1684, 1448, 1212, 1070, 749, 691

1-Phenyl-2-(4-((tetrahydro-2*H*-pyran-2-yl)oxy)tetrahydro-2*H*-pyran-2-yl)ethan-1-one 4z

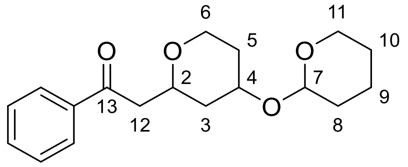

A 5 mL microwave vial was charged with tetrahydropyran **4ab** (51.9 mg, 0.236 mmol, 1.00 eq.), pyridinium *para*-toluenesulfonate (6.0 mg, 0.024 mmol, 0.10 eq.) and dry CH_2Cl_2 (0.3 mL) under nitrogen. 3,4-dihydro-2*H*-pyran (30.0 mg, 0.354 mmol, 1.50 eq.) was then added and the resulting mixture was stirred at RT for 2 h. The mixture was diluted with Et_2O (5 mL), washed with water (3 x 5 mL) and brine (5 mL). The organic layer was dried over Na_2SO_4, filtered and concentrated *in vacuo*. Purification by FCC (10-15% EtOAc/pentane) afforded the product as a colourless oil (53.4 mg, 0.175 mmol, 74% yield, 33:33:17:17 d.r.).

^{1}H NMR (400 MHz, $CDCl_3$) δ 8.00 – 7.92 (m, 8H, major ArCH x 2, major ArCH′ x 2, minor ArCH x 2, minor ArCH′ x 2), 7.60 – 7.50 (m, 4H, major ArCH, major ArCH′, minor ArCH, minor ArCH′), 7.49 – 7.41 (m, 8H, major ArCH x 2, major ArCH′ x 2, minor ArCH x 2, minor ArCH′ x 2), 4.80 – 4.67 (m, 4H, major H_7, major $H_{7'}$, minor H_7, minor $H_{7'}$), 4.50 – 4.30 (m, 2H, major H_2, major $H_{2'}$), 4.15 – 4.07 (m, 2H, major H_4, major $H_{4'}$), 4.06 – 3.68 (m, 14H, major H_{11a}, major $H_{11a'}$, minor H_{11a}, minor $H_{11a'}$, major H_{6a}, major $H_{6a'}$, minor H_{6a}, minor $H_{6a'}$, major H_{6b}, major $H_{6b'}$, minor H_4, minor $H_{4'}$, minor H_2, minor $H_{2'}$), 3.55 – 3.21 (m, 10H, major H_{11b}, major $H_{11b'}$, minor H_{11b}, minor $H_{11b'}$, minor H_{6b}, minor $H_{6b'}$, major H_{12a}, major $H_{12a'}$, minor H_{12a}, minor $H_{12a'}$), 3.02 – 2.82 (m, 4H, major H_{12b}, major $H_{12b'}$, minor H_{12b}, minor $H_{12b'}$), 2.21 – 1.18 (m, 40H, major H_3 x 2, major $H_{3'}$ x 2, minor H_3 x 2, minor $H_{3'}$ x 2, major H_5 x 2, major $H_{5'}$ x 2, minor H_5 x 2, minor $H_{5'}$ x 2, major H_9 x 2, major $H_{9'}$ x 2, minor H_9 x 2, minor $H_{9'}$ x 2, major H_{10} x 2, major $H_{10'}$ x 2, minor H_{10} x 2, minor $H_{10'}$ x 2, major H_8 x 2, major $H_{8'}$ x 2, minor H_8 x 2, minor $H_{8'}$ x 2).
^{13}C NMR (101 MHz, $CDCl_3$) δ 198.4, 198.0, 137.5, 137.4, 137.3, 137.2, 133.3, 133.3, 133.2, 133.1, 128.7, 128.7, 128.7, 128.6, 128.4, 128.4, 128.4, 97.0, 96.8, 96.6, 73.0, 72.8, 72.2, 71.8, 69.3, 69.0, 68.0, 68.0, 66.4, 66.2, 63.6, 63.1, 63.1, 62.9, 62.9, 62.6, 45.2, 45.1, 45.1, 45.0, 39.8, 38.3, 37.9, 35.5, 34.0, 32.2, 31.9, 31.3, 31.3, 31.2, 29.5, 25.7, 25.7, 25.6, 20.1, 19.9, 19.9, 19.7.
N.B. The peak at 198.4, 198.0, 128.4, 97.0, 31.3, 25.6 ppm corresponds to two overlapping signals.
HRMS (ESI+): Found $[M+Na]^+$ = 327.1565; $C_{18}H_{24}O_4Na$ requires 327.1567, Δ -0.48 ppm
IR (film) ν_{max}/cm^{-1}: 2944, 2867, 1686, 1075, 1024, 692

1-phenyl-2-((2*S*,4*R*)-4-(4,4,5,5-tetramethyl-1,3,2-dioxaborolan-2-yl)tetrahydro-2*H*-pyran-2-yl)ethan-1-one 4aa

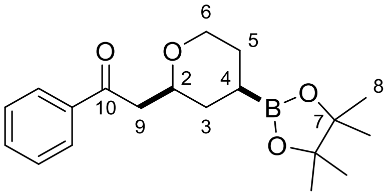

major (*cis*)

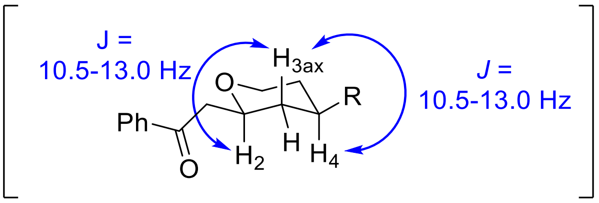

A 100 mL RBF was charged with CuI (86 mg, 0.45 mmol, 3 mol%), NaO^{t}Bu (43 mg, 0.45 mmol, 3 mol%) and BINAP (298 mg, 0.450 mmol, 3 mol%) in dry THF (10 mL) under nitrogen, and the resulting mixture was stirred at RT for 30 min. Bis(pinacolato)diboron (4.19 g, 16.5 mmol, 1.10 eq.) in dry THF (10 mL) was added. After stirring for 10 min, 5,6-dihydro-2*H*-pyran-2-one (1.49

g, 15.0 mmol, 1.00 eq.) was added, followed by dry MeOH (0.60 mL, 15 mmol, 1.0 eq.). Dry THF (10 mL) was added to bring the total concentration to 0.5 M, and the resulting mixture was stirred for 24 h, filtered through a plug of celite, and the solvents removed in vacuo to afford 4-(4,4,5,5-tetramethyl-1,3,2-dioxaborolan-2-yl)tetrahydro-2*H*-pyran-2-one, which was used without further purification.

4-(4,4,5,5-Tetramethyl-1,3,2-dioxaborolan-2-yl)tetrahydro-2*H*-pyran-2-one (3.42 g, 15.0 mmol, 1.00 eq.), DIBAL-H (1M in hexanes, 20 mL, 20 mmol, 1.3 eq.) and CH_2Cl_2 (30 mL) were subjected to **General Procedure 1**, affording 4-(4,4,5,5-tetramethyl-1,3,2-dioxaborolan-2-yl)tetrahydro-2*H*-pyran-2-ol which was used without further purification.

Phosphonate **S21** (3.08 g, 12.0 mmol, 1.20 eq.), 60% wt. NaH (480 mg, 12.0 mmol, 1.20 eq.), 4-(4,4,5,5-tetramethyl-1,3,2-dioxaborolan-2-yl)tetrahydro-2*H*-pyran-2-ol (3.30 g, 10.0 mmol, 1.00 eq.) and dry toluene (50 mL) were subjected to **General Procedure 3**. Purification by FCC (10-30% Et_2O/pentane) afforded the product as a white solid (630 mg, 1.91 mmol, 13% yield over 3 steps, 73:27 d.r.).

^{1}H NMR (600 MHz, $CDCl_3$) δ 7.97 – 7.91 (m, 4H, major ArCH x 2, minor ArCH x 2), 7.56 – 7.49 (m, 2H, major ArCH, minor ArCH), 7.46 – 7.40 (m, 4H, major ArCH x 2, minor ArCH x 2), 4.02 – 3.89 (m, 3H, minor H_2, major H_{6a}, major H_2), 3.87 – 3.81 (m, 1H, minor H_{6a}), 3.53 – 3.41 (m, 2H, minor H_{6b}, major H_{6b}), 3.28 – 3.20 (m, 2H, major H_{9a}, minor H_{9a}), 2.94 – 2.86 (m, 2H, major H_{9b}, minor H_{9b}), 1.89 – 1.84 (m, 1H, minor H_{3eq}), 1.83 – 1.77 (m, 1H, major H_{3eq}), 1.74 – 1.63 (m, 2H, minor H_{5a}, major H_{5a}), 1.59 – 1.45 (m, 4H, minor H_{5b}, major H_{5b}, minor H_{3ax} minor H_4), 1.32 (td, *J* = 13.0, 10.5 Hz, 1H, major H_{3ax}), 1.27 – 1.19 (m, 25H, major H_4, major H_8 x 12, minor H_8 x 12).

^{13}C NMR (151 MHz, $CDCl_3$) δ 198.4, 198.4, 137.5, 137.4, 133.1, 133.0, 128.6, 128.6, 128.4, 128.3, 83.5, 83.2, 74.8, 73.3, 69.4, 67.6, 45.6, 45.5, 33.1, 33.1, 27.1, 27.0, 25.0, 24.9, 24.9, 24.8, 19.5 (broad), 17.4 (broad).

m.p. = 82-83 °C

HRMS (ESI+): Found $[M+H]^+$ = 331.2076; $C_{19}H_{28}BO_4$ requires 331.2076, Δ 0 ppm

IR (film) $ν_{max}/cm^{-1}$: 2977, 1685, 1372, 1318, 1144, 691

2-((2*R*,4*R*)-4-hydroxytetrahydro-2*H*-pyran-2-yl)-1-phenylethan-1-one 4ab

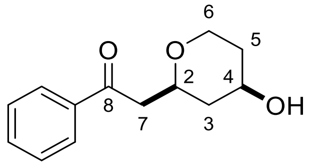

major (*cis*)

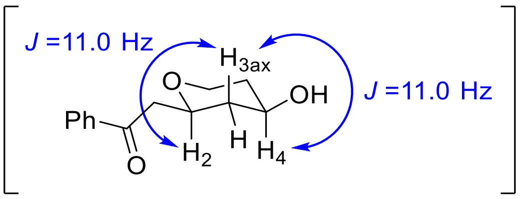

A 25 mL RBF was charged with tetrahydropyran **4aa** (118 mg, 0.356 mmol, 1.00 eq.) in THF (3 mL) and water (3 mL). Sodium perborate (tetrahydrate, 274 mg, 1.78 mmol, 5.00 eq.) was added and the reaction was stirred at RT for 30 min. Water (5 mL) was added, and then the mixture was extracted with EtOAc (3 x 15 mL). The organic layers were washed with brine, dried with $MgSO_4$ and concentrated *in vacuo*. Purification by FCC (50% Et_2O/pentane) afforded the product as a colourless oil (68.2 mg, 0.310 mmol, 86% yield, 87:13 d.r.).

^{1}H NMR (400 MHz, $CDCl_3$) δ 8.00 – 7.91 (m, 4H, major ArCH x 2, minor ArCH x 2), 7.60 – 7.52 (m, 2H, major ArCH, minor ArCH), 7.51 – 7.41 (m, 4H, major ArCH x 2, minor ArCH x 2), 4.47 – 4.36 (m, 1H, minor H_2), 4.30 – 4.21 (m, 1H, minor H_4), 4.03 – 3.72 (m, 5H, major H_2, minor H_{6a}, major H_{6a}, major H_4, minor H_{6b}), 3.51 – 3.40 (m, 1H, major H_{6b}), 3.40 – 3.24 (m, 2H, major H_{7a}, minor H_{7a}), 3.01 – 2.87 (m, 2H, major H_{7b}, minor H_{7b}), 2.15 – 2.07 (m, 1H, major H_{3eq}), 1.94 – 1.80 (m, 3H, major H_{5a}, minor H_{5a}, minor H_{3eq}), 1.72 (s, 1H, major OH), 1.67 (s, 1H, minor OH), 1.65 – 1.59 (m, 2H, minor H_{5b}, minor H_{3ax}), 1.59 – 1.46 (m, 1H, major H_{5b}), 1.31 (q, J = 11.0 Hz, 1H, major H_{3ax}).

^{13}C NMR (101 MHz, $CDCl_3$) δ 198.5, 198.0, 137.3, 137.2, 133.4, 133.3, 128.7, 128.7, 128.4, 128.4, 72.7, 68.6, 68.0, 66.1, 63.9, 62.8, 45.1, 44.9, 41.6, 39.1, 35.5, 32.8.

HRMS (ESI+): Found $[M+H]^+$ = 221.1172; $C_{13}H_{17}O_3$ requires 221.1172, Δ 0.09 ppm

IR (film) ν_{max}/cm^{-1}: 3431 (broad), 2922, 1682, 1066, 752, 691

1-Phenyl-2-((2*R*,4*R*)-4-phenyltetrahydro-2*H*-pyran-2-yl)ethan-1-one, 4ac

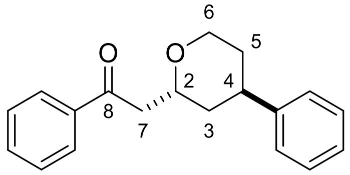

major (*trans*)

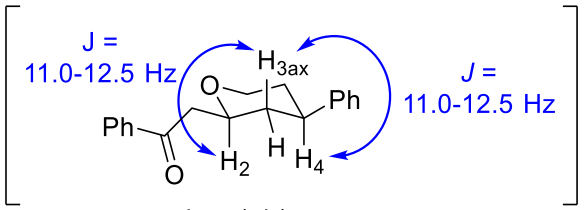

minor (*cis*)

Phosphonate **S21** (358 mg, 1.4 equiv., 1.40 mmol), dry toluene (5 mL) 60% wt. NaH (48 mg, 1.2 eq., 1.2 mmol) and lactol **1c** (178 mg, 1 equiv., 1.00 mmol) were subjected to **General**

Procedure 3. The title compound was afforded after purification by FCC (5-10% Et_2O/pentane) as a colourless oil (233 mg, 0.831 mmol, 83% yield, 43:57 *cis*/*trans*, 99:1 e.r).

^{1}H NMR (400 MHz, $CDCl_3$) δ 8.01 (ddd, *J* = 7.5, 6.5, 1.5 Hz, 4H, major ArCH x 2, minor ArCH x 2), 7.65 – 7.55 (m, 2H, major ArCH, minor ArCH), 7.51 (dd, *J* = 8.0, 6.5 Hz, 4H, major ArCH x 2, minor ArCH x 2), 7.41 – 7.30 (m, 6H, major ArCH x 3, minor ArCH x 3), 7.30 – 7.18 (m, 4H, major ArCH x 2, minor ArCH x 2), 4.57 (td, *J* = 6.5, 4.5 Hz, 1H, major H_2), 4.21 – 4.09 (m, 2H, minor H_2, minor H_{6a}), 3.92 – 3.73 (m, 2H, major H_6 x 2), 3.73 – 3.61 (m, 1H, minor H_{6b}), 3.50 (dd, *J* = 16.0, 6.5 Hz, 1H, major H_{7a}), 3.40 (dd, *J* = 16.0, 6.5 Hz, 1H, minor H_{7a}), 3.27 – 3.12 (m, 2H, major H_{7b}, major H_4), 3.02 (dd, *J* = 16.0, 5.5 Hz, 1H, minor H_{7b}), 2.89 (ddt, *J* = 12.0, 9.0, 4.0 Hz, 1H, minor H_4), 2.23 (ddd, *J* = 12.5, 8.0, 4.0 Hz, 1H, major H_{3eq}), 2.08 – 1.76 (m, 6H, major H_{3ax}, minor H_{3eq}, major H_5 x 2, minor H_5 x 2), 1.57 (td, *J* = 12.5, 11.0 Hz, 1H, minor H_{3ax}).

^{13}C NMR (101 MHz, $CDCl_3$) δ 198.4, 198.3, 145.6, 144.5, 137.4, 137.3, 133.3, 133.3, 128.8, 128.7, 128.7, 128.7, 128.4, 128.3, 127.4, 126.9, 126.5, 126.3, 74.4, 69.8, 68.5, 62.9, 45.3, 42.4, 41.7, 39.7, 35.9, 35.3, 33.4, 32.3.

HRMS (ESI+): Found $[M+H]^+$ = 281.1537; $C_{19}H_{21}O_2$ requires 281.1536, Δ 0.45 ppm

IR (film) ν_{max}/cm^{-1}: 2955, 2921, 2870, 2842, 1683, 1586

α_{25}^{D}= −8.5 (c = 1.0, $CHCl_3$).

HPLC: Enantiomeric excess was determined by HPLC with a Chiralpak® IC column (97:3 hexane:IPA, 1.0 mL min^{-1}, 210 nm, room temperature); minor diastereomer t_r (minor) = 11.3 min, major diastereomer t_r (minor) = 19.0 min, minor diastereomer t_r (major) = 22.2 min, major diastereomer t_r (major) = 22.8 min.

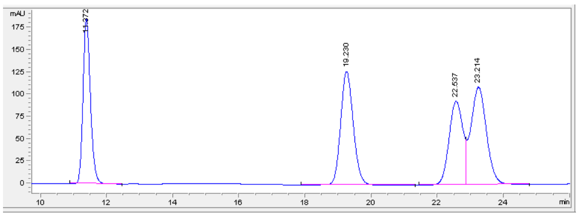

#	Time	Type	Area	Height	Width	Area%	Symmetry
1	11.372	BB	2894.3	185.8	0.2397	22.799	0.785
2	19.23	BB	3426.1	128.1	0.4141	26.988	0.854
3	22.537	BV	2803.6	94.3	0.4547	22.085	1.008
4	23.214	VB	3570.7	110	0.4907	28.128	0.868

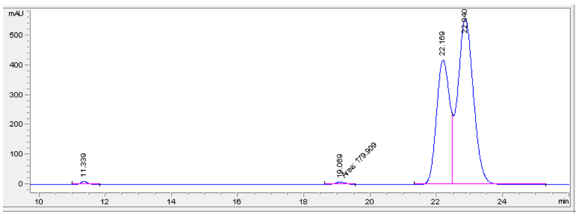

#	Time	Type	Area	Height	Width	Area%	Symmetry
1	11.339	BB	151.3	10	0.2331	0.497	0.805
2	19.069	MM	179.9	7.6	0.3945	0.591	1.046
3	22.169	BV	11894.9	421	0.4356	39.081	0.952
4	22.84	VB	18210.2	560.2	0.4932	59.830	0.842

2-((2*S*,4*R*)-4-(4-Fluorophenyl)tetrahydro-2*H*-pyran-2-yl)-1-phenylethan-1-one, 4ad

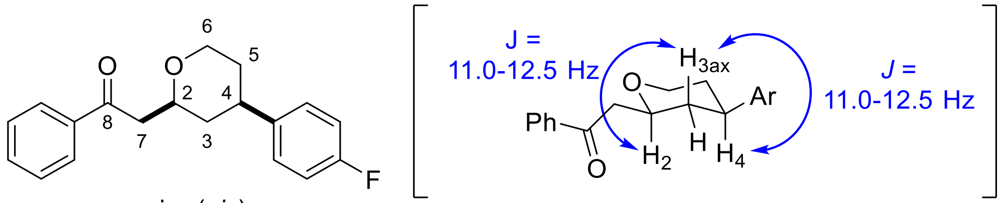

major (*cis*)

Phosphonate **S21** (358 mg, 1.4 equiv., 1.40 mmol), dry toluene (5 mL) 60% wt. NaH (48 mg, 1.2 eq., 1.2 mmol) and lactol **S9** (196 mg, 1 equiv., 1.00 mmol) were subjected to **General Procedure 3**. The title compound was afforded after purification by FCC (10% Et_2O/pentane) as a white solid (290 mg, 0.972 mmol, 97% yield, 94:6 *cis*/*trans*, 98:2 e.r).

^{1}H NMR (400 MHz, $CDCl_3$) δ 8.00 – 7.94 (m, 2H, ArCH x 2), 7.60 – 7.54 (m, 1H, ArCH), 7.50 – 7.43 (m, 2H, ArCH x 2), 7.21 – 7.14 (m, 2H, ArCH x 2), 7.03 – 6.96 (m, 2H, ArCH x 2), 4.16 – 4.07 (m, 2H, H_2 and H_{6a}), 3.66 – 3.58 (m, 1H, H_{6b}), 3.37 (dd, *J* = 16.0, 6.5 Hz, 1H, H_{7a}), 2.99 (dd, *J* = 16.0, 6.0 Hz, 1H, H_{7b}), 2.91 – 2.80 (m, 1H, H_4), 1.99 (ddt, *J* = 12.5, 3.5, 1.5 Hz, 1H, H_{3eq}), 1.78 – 1.71 (m, 2H, H_5 x 2), 1.49 (td, *J* = 12.5, 11.0 Hz, 1H, H_{3ax}). The minor diastereoisomer displays diagnostic signals at δ = 4.52 (qd, *J* = 6.0, 4.0 Hz, 1H), 3.83 (ddd, *J* = 11.5, 7.5, 4.0 Hz, 1H), 3.74 (ddd, *J* = 12.0, 6.5, 4.0 Hz, 1H), 3.46 (dd, *J* = 16.0, 6.0 Hz, 1H), 3.23 – 3.09 (m, 2H), 2.21 – 2.13 (m, 1H).

^{13}C NMR (101 MHz, $CDCl_3$) δ 198.2, 161.6 (d, *J* = 244.0 Hz), 141.3 (d, *J* = 3.0 Hz), 137.3, 133.3, 128.7, 128.4, 128.2 (d, *J* = 83.0 Hz), 115.4 (d, *J* = 21.0 Hz), 74.3, 68.4, 45.2, 41.0, 39.8, 33.5.

^{19}F NMR (377 MHz, $CDCl_3$) δ –116.9. The minor diastereoisomer displays diagnostic signal at δ = –117.2.

HRMS (ESI+): Found $[M+H]^+$ = 299.1441; $C_{19}H_{20}O_2F$ requires 299.1442, Δ –0.27 ppm

IR (film) ν_{max}/cm^{-1}: 2935, 2847, 1684, 1599, 1510, 1221, 1083, 833, 753, 691.

m.p.: 62-65 °C

α_{25}^{D}= −9.0 (c = 1.0, $CHCl_3$).

HPLC: Enantiomeric excess was determined by HPLC with a Chiralpak® IB-N column (98:2 hexane:IPA, 1.0 mL min^{-1}, 254 nm, room temperature); minor diastereomer t_r (minor) = 11.8 min, major diastereomer t_r (major) = 14.0 min, minor diastereomer t_r (major) = 14.9 min, major diastereomer t_r (minor) = 18.2 min.

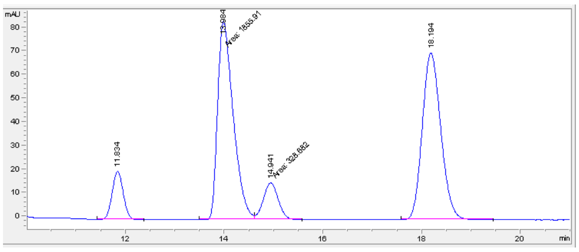

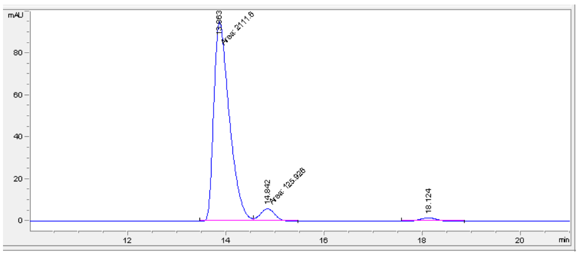

#	Time	Type	Area	Height	Width	Area%	Symmetry
1	11.834	BB	320.7	20.5	0.2448	7.369	0.948
2	13.984	MF	1855.9	84.3	0.3671	42.640	0.586
3	14.941	FM	328.7	15.6	0.3509	7.552	0.946
4	18.194	BB	1847.2	70.8	0.4065	42.439	0.825

#	Time	Type	Area	Height	Width	Area%	Symmetry
1	13.863	MF	2111.6	95.3	0.3693	92.664	0.568
2	14.842	FM	125.9	6	0.3508	5.526	0.96
3	18.124	BB	41.2	1.7	0.3782	1.809	0.956

1-Phenyl-2-((2*S*,4*R*)-4-(*o*-tolyl)tetrahydro-2*H*-pyran-2-yl)ethan-1-one, 4ae

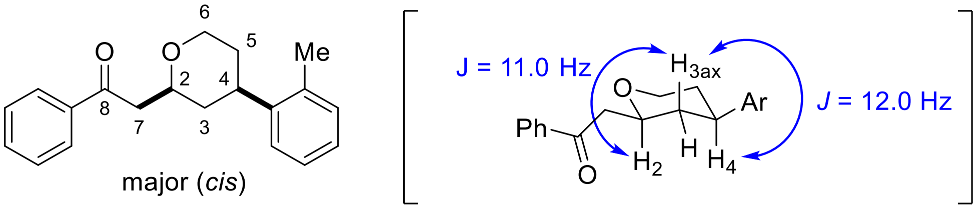

Phosphonate **S21** (358 mg, 1.4 equiv., 1.40 mmol), dry toluene (5 mL) 60% wt. NaH (48 mg, 1.2 eq., 1.2 mmol) and lactol **S11** (192 mg, 1 equiv., 1.00 mmol) were subjected to **General Procedure 3**. The title compound was afforded after purification by FCC (20% Et_2O/pentane) as a white solid (284 mg, 0.965 mmol, 96% yield, >95:5 *cis*/*trans*, 95:5 e.r).

^{1}H NMR (400 MHz, $CDCl_3$) δ 8.01 – 7.95 (m, 2H, ArCH x 2), 7.60 – 7.54 (m, 1H, ArCH), 7.50 – 7.43 (m, 2H, ArCH x 2), 7.24 – 7.08 (m, 4H, ArCH x 4), 4.21 – 4.09 (m, 2H, H_2 and H_{6eq}), 3.67 (td, *J* = 12.0, 2.5 Hz, 1H, H_{6ax}), 3.39 (dd, *J* = 16.0, 7.0 Hz, 1H, H_{7a}), 3.10 (tt, *J* = 12.0, 3.5 Hz, 1H, H_4), 2.98 (dd, *J* = 16.0, 5.5 Hz, 1H, H_{7b}), 2.38 (s, 3H, $ArCH_3$ x 3), 1.92 (ddt, *J* = 13.0, 4.0, 2.0 Hz, 1H, H_{3eq}), 1.80 (dtd, *J* = 13.5, 12.0, 4.5 Hz, 1H, H_{5ax}), 1.70 (ddq, *J* = 13.5, 4.0, 2.0 Hz, 1H, H_{5eq}), 1.56 (ddd, *J* = 13.0, 12.0, 11.0 Hz, 1H, H_{3ax}). The minor diastereoisomer displays diagnostic signals at δ = 4.72 – 4.66 (m, 1H), 3.92 – 3.84 (m, 2H).

^{13}C NMR (101 MHz, $CDCl_3$) δ 198.3, 143.5, 137.4, 135.3, 133.3, 130.6, 128.7, 128.4, 126.4, 126.2, 125.5, 74.6, 68.7, 45.3, 38.8, 37.4, 32.7, 19.5.

HRMS (ESI+): Found $[M+H]^+$ = 295.1695; $C_{20}H_{23}O_2$ requires 295.1693, Δ 0.70 ppm

IR (film) ν_{max}/cm^{-1}:2937, 2845, 1684, 1448, 1378, 1084, 975, 752, 690.

m.p.: 90-93 ^{o}C

$\alpha_{25}{}^{D}$= +2.3 (c = 1.0, $CHCl_3$).

HPLC: Enantiomeric excess was determined by HPLC with a Chiralpak® IA column (99:1 hexane:IPA, 1.0 mL min^{-1}, 230 nm, room temperature); major diastereomer t_r (major) =

15.4 min, minor diastereomer t_r (minor)= 18.1 min, major diastereomer t_r (minor) = 21.0 min, minor diastereomer t_r (major) = 25.4 min.

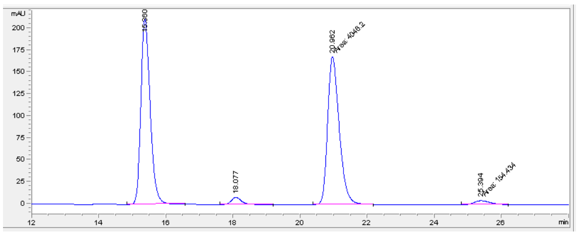

#	Time	Type	Area	Height	Width	Area%	Symmetry
1	15.36	BB	4063.4	210.4	0.2993	48.200	0.714
2	18.077	BB	166.3	8.1	0.3184	1.973	0.79
3	20.962	MF	4046.2	167.7	0.4021	47.995	0.691
4	25.394	MF	154.4	4.5	0.569	1.832	0.695

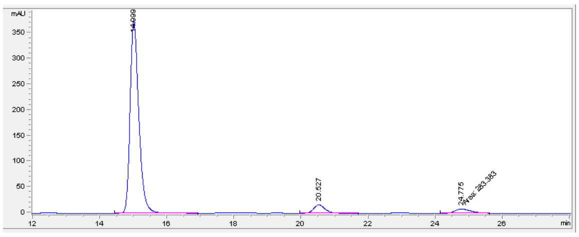

#	Time	Type	Area	Height	Width	Area%	Symmetry
1	14.999	BB	6804.8	376.5	0.2769	91.046	0.68
2	20.527	BB	385.8	16.5	0.3594	5.162	0.804
3	24.775	MF	283.4	8.3	0.5668	3.792	0.636

2-((2*S*,4*R*)-4-(4-Methoxyphenyl)tetrahydro-2*H*-pyran-2-yl)-1-phenylethan-1-one, 4af

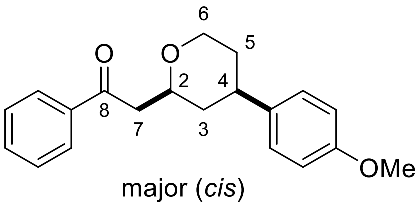

major (*cis*)

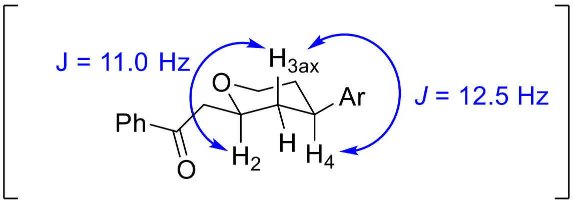

Phosphonate **S21** (358 mg, 1.4 equiv., 1.40 mmol), dry toluene (5 mL) 60% wt. NaH (48 mg, 1.2 eq., 1.2 mmol) and lactol **S13** (192 mg, 1 equiv., 1.00 mmol) were subjected to **General Procedure 3**. The title compound was afforded after purification by FCC (20% Et_2O/pentane) as a white solid (295 mg, 0.950 mmol, 95% yield, 95:5 *cis*/*trans*, 99:1 e.r).

^{1}H NMR (400 MHz, $CDCl_3$) δ 8.00 – 7.95 (m, 2H, ArCH x 2), 7.59 – 7.54 (m, 1H, ArCH), 7.51 – 7.43 (m, 2H, ArCH x 2), 7.18 – 7.12 (m, 2H, ArCH x 2), 6.89 – 6.84 (m, 2H, ArCH x 2), 4.16 – 4.06 (m, 2H, H_2 and H_{6a}), 3.79 (s, 3H, OCH_3 x 3), 3.66 – 3.58 (m, 1H, H_{6b}), 3.37 (dd, *J* = 16.0, 6.5 Hz, 1H, H_{7a}), 2.99 (dd, *J* = 16.0, 5.5 Hz, 1H, H_{7b}), 2.82 (dtd, *J* = 12.5, 9.5, 3.5 Hz, 1H, H_4), 1.98 (br d, *J* = 12.5 Hz, 1H, H_{3eq}), 1.78 – 1.71 (m, 2H, H_5 x 2), 1.50 (td, *J* = 12.5, 11.0 Hz, 1H, H_{3ax}). The minor diastereoisomer displays diagnostic signals at δ = 4.53 (qd, *J* = 6.5, 4.0 Hz, 1H, H_2), 3.46 (dd, *J* = 16.0, 6.5 Hz, 1H, H_{7a}), 3.18 (dd, *J* = 16.0, 7.0 Hz, 1H, H_{7b}), 3.13 – 3.06 (m, 1H, H_4), 2.17 (ddd, *J* = 13.5, 8.0, 4.0 Hz, 1H, H_{3a}).

^{13}C NMR (101 MHz, $CDCl_3$) δ 198.3, 158.2, 137.9, 137.3, 133.3, 128.7, 128.4, 127.7, 114.0, 74.4, 68.5, 55.4, 45.3, 40.8, 39.9, 33.6.

HRMS (ESI+): Found $[M+H]^+$ = 311.1643; $C_{20}H_{23}O_3$ requires 311.1642, Δ 0.32 ppm

IR (film) ν_{max}/cm^{-1}:2934, 2838, 1684, 1513, 1248, 1179, 1082, 1034, 829, 753, 691.

m.p.: 99-101 °C

α_{25}^{D}= –7.6 (c = 1.0, $CHCl_3$).

HPLC: Enantiomeric excess was determined by HPLC with a Chiralpak® IB-N column (98:2 hexane:IPA, 1.0 mL min^{-1}, 230 nm, room temperature); minor diastereomer t_r (minor) = 16.1 min, minor diastereomer t_r (major) = 18.5 min, major diastereomer t_r (major) = 19.4 min, major diastereomer t_r (minor)= 24.0 min.

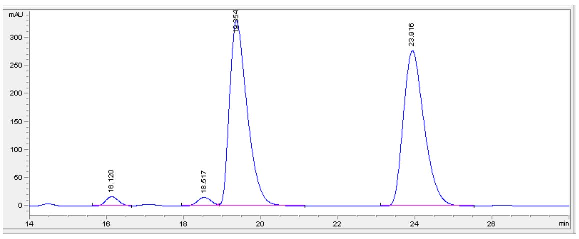

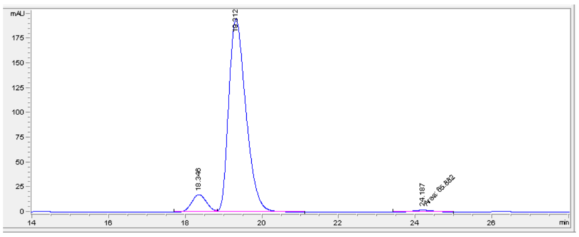

#	Time	Type	Area	Height	Width	Area%	Symmetry
1	16.12	BB	386.8	16.8	0.3599	1.780	0.894
2	18.517	BV E	393.8	15.6	0.4008	1.813	0.986
3	19.354	VB R	10522.7	330.9	0.487	48.429	0.732
4	23.916	BB	10424.7	277.7	0.58	47.978	0.729

#	Time	Type	Area	Height	Width	Area%	Symmetry
1	18.346	BV E	472.1	17.6	0.4214	7.157	0.939
2	19.312	VB R	6058.3	194.1	0.4841	91.844	0.997
3	24.187	MM	65.9	1.8	0.5945	0.999	0.948

1-Phenyl-2-((2*S*,4*R*)-4-(prop-1-en-2-yl)tetrahydro-2*H*-pyran-2-yl)ethan-1-one, 4ag

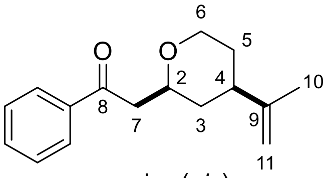

major (*cis*)

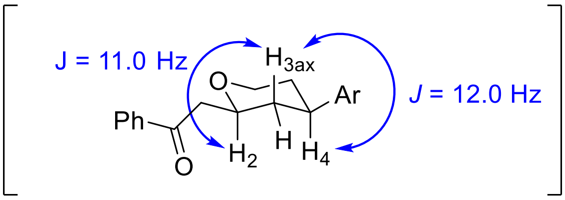

Phosphonate **S21** (358 mg, 1.4 equiv., 1.40 mmol), dry toluene (5 mL) 60% wt. NaH (48 mg, 1.2 eq., 1.2 mmol) and lactol **S14** (142 mg, 1 equiv., 1.00 mmol) were subjected to **General Procedure 3**. The title compound was afforded after purification by FCC (10% Et_2O/pentane) as a pale yellow oil (228 mg, 0.933 mmol, 93% yield, 88:12 *cis*/*trans*, 98:2 e.r).

^{1}H NMR (400 MHz, $CDCl_3$) δ 7.99 – 7.94 (m, 2H, ArCH x 2), 7.58 – 7.53 (m, 1H, ArCH), 7.49 – 7.43 (m, 2H, ArCH x 2), 4.74 – 4.72 (m, 1H, H_{11a}), 4.71 – 4.69 (m, 1H, H_{11b}), 4.05 – 3.97 (m, 2H, H_2 and H_{6eq}), 3.51 (ddd, *J* = 12.5, 11.5, 2.5 Hz, 1H, H_{6ax}), 3.33 (dd, *J* = 16.0, 6.5 Hz, 1H, H_{7a}), 2.96 (dd, *J* = 16.0, 5.5 Hz, 1H, H_{7b}), 2.21 (tt, *J* = 12.0, 3.5 Hz, 1H, H_4), 1.86 (ddt, *J* = 13.0, 4.0, 2.0 Hz, 1H, H_{3eq}), 1.73 (s, 3H, H_{10} x 3), 1.68 – 1.60 (m, 1H, H_{5eq}), 1.56 – 1.47 (m, 1H, H_{5ax}), 1.27 (ddd, *J* = 13.0, 12.0, 11.0 Hz, 1H, H_{3ax}). The minor diastereoisomer displays diagnostic signals at δ = 4.92 – 4.90 (m, 1H, H_{11a}), 4.86 – 4.84 (m, 1H, H_{11b}), 4.39 (qd, *J* = 7.0, 3.5 Hz, 1H, H_2), 3.78 – 3.68 (m, 2H, H_6 x 2), 3.36 (dd, *J* = 16.0, 6.0 Hz, 1H, H_{7a}), 3.07 (dd, *J* = 16.0, 7.0 Hz, 1H, H_{7b}), 2.42 (p, *J* = 5.5 Hz, 1H, H_4), 1.97 (dddd, *J* = 13.5, 6.5, 3.5, 1.0 Hz, 1H, H_{3a}).

^{13}C NMR (101 MHz, $CDCl_3$) δ 198.3, 148.9, 137.4, 133.2, 128.7, 128.4, 109.1, 74.2, 68.3, 45.4, 42.5, 37.2, 31.2, 20.8. The minor diastereoisomer displays signals at δ = 198.4, 146.8, 137.3, 133.2, 128.7, 128.3, 111.0, 69.7, 63.3, 43.2, 36.7, 34.1, 29.4, 22.1.

HRMS (ESI+): Found $[M+H]^+$ = 245.1538; $C_{16}H_{21}O_2$ requires 245.1536, Δ 0.89 ppm

IR (film) ν_{max}/cm^{-1}: 2936, 2846, 1684, 1448, 1129, 1086, 975, 889, 752, 690.

α_{25}^{D}= –18.3 (c = 1.0, $CHCl_3$).

HPLC: Enantiomeric excess was determined by HPLC with a Chiralpak® IA column (99:1 hexane:IPA, 1.0 mL min^{-1}, 230 nm, room temperature); major diastereomer t_r (major) = 10.8 min, minor diastereomer t_r (minor) = 13.9 min, major diastereomer t_r (minor)= 15.2 min, minor diastereomer t_r (major) = 20.0 min.

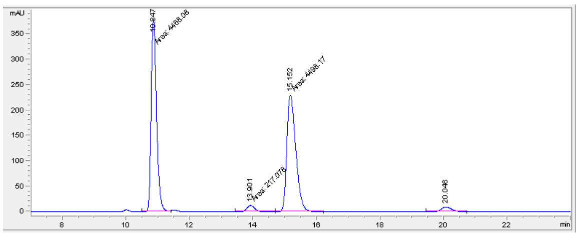

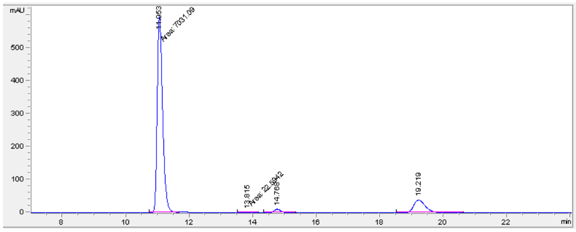

#	Time	Type	Area	Height	Width	Area%	Symmetry
1	10.847	MF	4468.1	379	0.1965	47.542	0
2	13.901	MF	217.1	12.6	0.2865	2.310	0
3	15.152	MF	4498.2	229.1	0.3273	47.862	0
4	20.046	BB	214.9	9.2	0.3624	2.286	0.814

#	Time	Type	Area	Height	Width	Area%	Symmetry
1	11.053	MF	7031.1	596	0.1966	85.722	0
2	13.815	MM	22.6	1.6	0.2382	0.275	0.816
3	14.768	BB	158.3	10.6	0.2306	1.930	0.847
4	19.219	BB	990.2	38.7	0.3906	12.073	0.653

rac-2-((2*R*,3*S*)-3-Methyltetrahydro-2*H*-pyran-2-yl)-1-phenylethan-1-one, 4ah

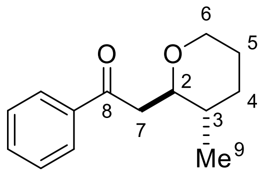

major (*trans*)

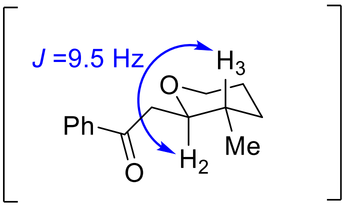

Phosphonate **S21** (358 mg, 1.4 equiv., 1.40 mmol), dry toluene (5 mL) 60% wt. NaH (48 mg, 1.2 eq., 1.2 mmol) and lactol **1b** (116 mg, 1 equiv., 1.00 mmol) were subjected to **General Procedure 3**. The title compound was afforded after purification by FCC (10% Et_2O/pentane) as a yellow oil (199 mg, 0.912 mmol, 91% yield, 20:80 *cis*/*trans*, >95:5 r.r.).

^{1}H NMR (400 MHz, $CDCl_3$) δ 7.99 – 7.94 (m, 2H, ArCH x 2), 7.58 – 7.51 (m, 1H, ArCH), 7.48 – 7.42 (m, 2H, ArCH x 2), 3.89 (ddt, *J* = 11.5, 4.5, 2.0 Hz, 1H, H_{6eq}), 3.63 (ddd, *J* = 9.5, 8.5, 3.5 Hz, 1H, H_2), 3.37 (ddd, *J* = 12.5, 11.5, 2.5 Hz, 1H, H_{6ax}), 3.17 (dd, *J* = 15.5, 8.5 Hz, 1H, H_{7a}), 3.05 (dd, *J* = 15.5, 3.5 Hz, 1H, H_{7b}), 1.87 – 1.77 (m, 1H, H_{4eq}), 1.72 – 1.58 (m, 1H, H_{5a}), 1.57 – 1.46 (m, 2H, H_{5b} and H_3), 1.23 (tdd, *J* = 13.0, 11.5, 4.0 Hz, 1H, H_{4ax}), 0.89 (d, *J* = 6.5 Hz, 3H, H_9 x 3). The

minor diastereoisomer displays diagnostic signals at δ = 4.12 (ddd, *J* = 7.5, 5.0, 2.5 Hz, 1H, H_2), 3.52 – 3.44 (m, 1H, H_{6b}), 3.26 (dd, *J* = 16.0, 8.0 Hz, 1H, H_{7a}), 2.82 (dd, *J* = 16.0, 5.0 Hz, 1H, H_{7b}), 1.04 (d, *J* = 7.0 Hz, 3H, H_9 x 3).

^{13}C NMR (101 MHz, $CDCl_3$) δ 199.4, 137.7, 133.0, 128.6, 128.5, 80.5, 68.5, 43.1, 36.1, 32.8, 26.6, 18.3. The minor diastereoisomer displays signals at δ = 198.8, 137.5, 133.1, 128.7, 128.4, 76.5, 69.0, 42.2, 31.2, 30.8, 21.0, 12.3.

HRMS (ESI+): Found $[M+H]^+$ = 219.1380; $C_{14}H_{19}O_2$ requires 219.1380, Δ –0.01 ppm

IR (film) ν_{max}/cm^{-1}: 2928, 2848, 1685, 1449, 1274, 1210, 1096, 751, 691.

rac-1-Phenyl-2-((2_R_,3_S_)-3-propyltetrahydro-2_H_-pyran-2-yl)ethan-1-one, 4ai

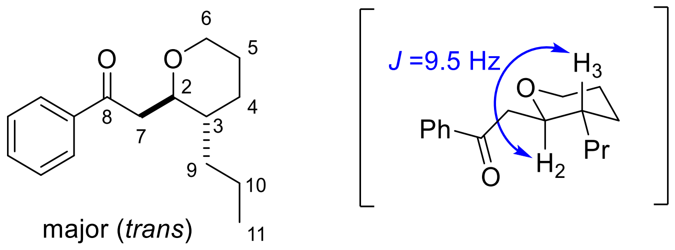

Phosphonate **S21** (358 mg, 1.4 equiv., 1.40 mmol), dry toluene (5 mL) 60% wt. NaH (48 mg, 1.2 eq., 1.2 mmol) and lactol **S15** (144 mg, 1 equiv., 1.00 mmol) were subjected to **General Procedure 3**. The title compound was afforded after purification by FCC (10% Et_2O/pentane) as a pale yellow oil (229 mg, 0.929 mmol, 93% yield, 20:80 *cis*/*trans*, >95:5 r.r.).

^{1}H NMR (400 MHz, $CDCl_3$) δ 7.99 – 7.95 (m, 2H, ArCH x 2), 7.57 – 7.52 (m, 1H, ArCH), 7.48 – 7.42 (m, 2H, ArCH x 2), 3.90 – 3.83 (m, 1H, H_{6eq}), 3.71 (ddd, *J* = 9.5, 8.0, 3.5 Hz, 1H, H_2), 3.35 (td, *J* = 11.5, 3.0 Hz, 1H, H_{6ax}), 3.17 (dd, *J* = 15.5, 8.0 Hz, 1H, H_{7a}), 3.08 (dd, *J* = 15.5, 3.5 Hz, 1H, H_{7b}), 1.99 – 1.92 (m, 1H, H_{4a}), 1.84 – 1.04 (m, 8H, H_{4b}, H_5 x 2, H_3, H_9 x 2 and H_{10} x 2), 0.89 (t, *J* = 7.0 Hz, 3H, H_{11} x 3). The minor diastereoisomer displays diagnostic signals at δ = 4.17 (ddd, *J* = 8.0, 4.5, 2.5 Hz, 1H), 3.54 – 3.46 (m, 1H), 3.31 (dd, *J* = 16.0, 8.0 Hz, 1H), 2.85 (dd, *J* = 16.0, 4.5 Hz, 1H).

^{13}C NMR (101 MHz, $CDCl_3$) δ 199.5, 137.8, 133.0, 128.6, 128.5, 79.3, 68.3, 43.1, 40.6, 34.4, 29.3, 26.5, 19.5, 14.5. The minor diastereoisomer displays diagnostic peaks at δ = 198.9, 137.5, 133.1, 128.7, 128.4, 41.4, 36.5, 28.2, 26.7, 21.5, 20.7.

HRMS (ESI+): Found $[M+H]^+$ = 247.1693; $C_{16}H_{23}O_2$ requires 247.1693, Δ 0.04 ppm

IR (film) ν_{max}/cm^{-1}: 2931, 2869, 1685, 1449, 1097, 751, 691.

2-(3-Allyltetrahydro-2*H*-pyran-2-yl)-1-phenylethan-1-one, 4aj

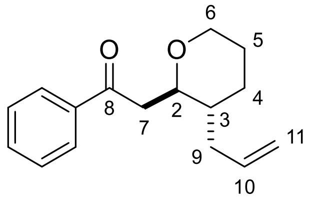

major *(trans)*

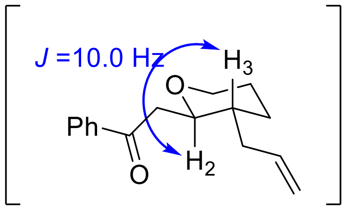

Phosphonate **S21** (358 mg, 1.4 equiv., 1.40 mmol), dry toluene (5 mL) 60% wt. NaH (48 mg, 1.2 eq., 1.2 mmol) and lactol **S16** (142 mg, 1 equiv., 1.00 mmol) were subjected to **General Procedure 3**. The title compound was afforded after purification by FCC (10% Et_2O/pentane) as a colourless oil (240 mg, 0.982 mmol, 98% yield, 25:75 *cis*/*trans*, >95:5 r.r.).

^{1}H NMR (400 MHz, $CDCl_3$) δ 8.02 – 7.92 (m, 4H, major ArCH x 2, minor ArCH x 2), 7.61 – 7.51 (m, 2H, major ArCH, minor ArCH), 7.49 – 7.40 (m, 4H, major ArCH x 2, minor ArCH x 2), 5.87 – 5.65 (m, 2H, major H_{10}, minor H_{10}), 5.16 – 4.96 (m, 4H, major H_{11} x 2, minor H_{11} x 2), 4.20 (ddd, *J* = 7.5, 5.0, 2.5 Hz, 1H, minor H_2), 3.99 – 3.83 (m, 2H, major H_{6eq}, minor H_{6eq}), 3.75 (ddd, *J* = 10.0, 7.5, 4.0 Hz, 1H, major H_2), 3.51 (td, *J* = 11.0, 2.5 Hz, 1H, minor H_{6ax}), 3.40 – 3.32 (m, 1H, major H_{6ax}) 3.31 (dd, *J* = 16.0, 7.5 Hz, 1H, minor H_{7a}), 3.16 (dd, *J* = 15.5, 7.5 Hz, 1H, major H_{7a}), 3.13 (dd, *J* = 15.5, 4.0 Hz, 1H, major H_{7b}), 2.89 (dd, *J* = 16.0, 5.0 Hz, 1H, minor H_{7b}), 2.32 – 2.24 (m, 2H, minor H_9 x 2), 2.24 – 2.13 (m, 1H, major H_{9a}), 1.97 – 1.46 (m, 9H, major H_3, major H_{4eq}, major H_5 x 2, major H_{9b}, minor H_3, minor H_4 x 2, minor H_{5eq}), 1.37 – 1.14 (m, 2H, major H_{4ax}, minor H_{5ax}).

^{13}C NMR (101 MHz, $CDCl_3$) δ 199.2, 198.6, 137.7, 137.7, 137.4, 136.0, 133.2, 133.0, 128.7, 128.6, 128.5, 128.4, 116.8, 116.1, 78.9, 76.5, 68.6, 68.3, 43.1, 41.7, 40.5, 37.0, 36.4, 30.9, 29.5, 26.9, 26.4, 21.1.

HRMS (ESI+): Found $[M+H]^+$ = 245.1537; $C_{16}H_{21}O_2$ requires 245.1536, Δ 0.45 ppm

IR (film) ν_{max}/cm^{-1} 3006, 2848, 1686, 1448, 1269, 1094, 996, 911, 750, 690.

1-Phenyl-2-(3-(prop-2-yn-1-yl)tetrahydro-2*H*-pyran-2-yl)ethan-1-one, 4ak

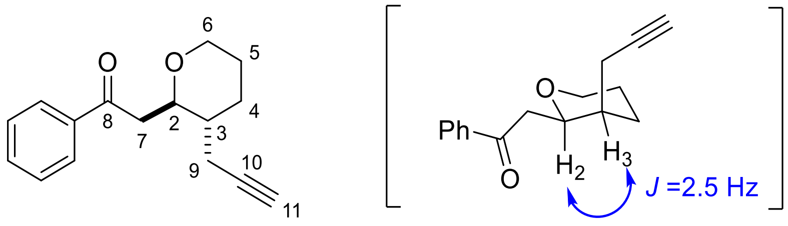

major *(trans)* minor (*cis*)

Phosphonate **S21** (358 mg, 1.4 equiv., 1.40 mmol), dry toluene (5 mL) 60% wt. NaH (48 mg, 1.2 eq., 1.2 mmol) and lactol **S17** (140 mg, 1 equiv., 1.00 mmol) were subjected to **General Procedure 3**. The title compound was afforded after purification by FCC (5-10% Et_2O/pentane) as a colourless oil (242 mg, 0.999 mmol, 100% yield, 37:63 *cis*/*trans*, >95:5 r.r.).

^1H NMR (400 MHz, $CDCl_3$) δ 8.03 – 7.90 (m, 4H, major ArCH x 2, minor ArCH x 2), 7.59 – 7.51 (m, 2H, major ArCH, minor ArCH), 7.50 – 7.40 (m, 4H, major ArCH x 2, minor ArCH x 2), 4.22 (ddd, *J* = 7.5, 5.0, 2.5 Hz, 1H, minor H_2), 3.98 – 3.79 (m, 3H, major H_2, major H_{6eq}, minor H_{6eq}), 3.51 (td, *J* = 11.0, 2.5 Hz, 1H, minor H_{6ax}), 3.36 (td, *J* = 11.5, 3.0 Hz, 1H, major H_{6ax}), 3.28 (dd, *J* = 16.5, 7.5 Hz, 1H, minor H_{7a}), 3.24 – 3.11 (m, 2H, major H_7 x 2), 2.97 (dd, *J* = 16.5, 5.0 Hz, 1H, minor H_{7b}), 2.51 – 2.36 (m, 2H, minor H_9 x 2), 2.31 – 2.15 (m, 2H, major H_9 x 2), 2.08 (dtd, *J* = 11.5, 3.5, 2.0 Hz, 1H, minor H_{4eq}), 2.04 – 1.96 (m, 3H, major H_{4eq}, major H_{11}, minor H_3), 1.93 (t, *J* = 2.5 Hz, 1H, minor H_{11}), 1.83 – 1.55 (m, 5H, major H_3, major H_5 x 2, minor H_{4ax}, minor H_{5eq}), 1.53 – 1.40 (m, 1H, major H_{4ax}), 1.39 – 1.31 (m, 1H, minor H_{5ax}).

^{13}C NMR (101 MHz, $CDCl_3$) δ 198.8, 198.1, 137.5, 137.3, 133.2, 133.1, 128.7, 128.6, 128.5, 128.3, 83.7, 81.8, 78.2, 75.7, 70.4, 69.7, 68.9, 68.3, 42.9, 41.8, 39.6, 36.2, 29.6, 27.8, 26.3, 22.0, 20.9, 16.4.

HRMS (ESI+): Found $[M+H]^+$ = 243.1380; $C_{16}H_{19}O_2$ requires 243.1380, Δ 0.24 ppm

IR (film) ν_{max}/cm^{-1} 3650, 3292, 2981, 2851, 1685, 1449, 1379, 1090, 968, 751, 691, 634.

2-(3-Benzyltetrahydro-2*H*-pyran-2-yl)-1-phenylethan-1-one, 4al

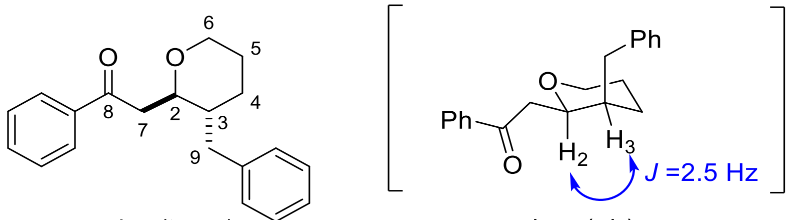

major *(trans)* minor (*cis*)

Phosphonate **S21** (358 mg, 1.4 equiv., 1.40 mmol), dry toluene (5 mL) 60% wt. NaH (48 mg, 1.2 eq., 1.2 mmol) and lactol **S18** (192 mg, 1 equiv., 1.00 mmol) were subjected to **General Procedure 3**. The title compound was afforded after purification by FCC (10-15% Et_2O/pentane) as a colourless oil (294 mg, 0.999 mmol, 100% yield, 44:56 *cis*/*trans*, >95:5 r.r.).
^{1}H NMR (400 MHz, $CDCl_3$) δ 8.09 – 7.91 (m, 4H, major ArCH x 2, minor ArCH x 2), 7.62 – 7.54 (m, 2H, major ArCH, minor ArCH), 7.52 – 7.44 (m, 4H, major ArCH x 2, minor ArCH x 2), 7.36 – 7.27 (m, 4H, major ArCH x 2, minor ArCH x 2), 7.26 – 7.14 (m, 6H, major ArCH x 3, minor ArCH x 3), 4.29 (ddd, *J* = 8.0, 5.0, 2.5 Hz, 1H, minor H_2), 4.02 (ddt, *J* = 11.5, 4.0, 2.0 Hz, 1H, minor H_{6eq}), 3.95 – 3.82 (m, 2H, major H_2, major H_{6eq}), 3.58 (td, *J* = 11.5, 3.0 Hz, 1H, minor H_{6ax}), 3.45 – 3.35 (m, 2H, major H_{6ax}, minor H_{7a}), 3.33 – 3.19 (m, 2H, major H_7 x 2), 2.98 (dd, *J* = 16.0, 5.0 Hz, 1H, minor H_{7b}), 2.93 – 2.76 (m, 3H, major H_{9a}, minor H_9 x 2), 2.30 (dd, *J* = 13.5, 10.0 Hz, 1H, major H_{9b}), 2.11 (ddq, *J* = 12.0, 6.0, 2.5 Hz, 1H, minor H_3), 1.97 – 1.67 (m, 3H, major H_3, major H_{4eq}, minor H_{5eq}), 1.68 – 1.49 (m, 4H, major H_5 x 2, minor H_4 x 2), 1.41 – 1.31 (m, 1H, minor H_{5ax}), 1.31 – 1.11 (m, 1H, major H_{4ax}). N.B. within the multiplet at δ = 3.45-3.35 ppm, a clear signal could be observed at δ = 3.39 (dd, *J* = 16.0, 8.0 Hz, minor H_{7a}).
^{13}C NMR (101 MHz, $CDCl_3$) δ 199.2, 198.6, 141.0, 139.9, 137.7, 137.3, 133.2, 133.1, 129.3, 129.1, 128.7, 128.6, 128.5, 128.5, 128.5, 128.3, 126.2, 126.0, 79.3, 76.6, 68.7, 68.3, 43.2, 42.5, 41.8, 38.9, 38.2, 32.4, 29.5, 26.4, 26.3, 21.3.
HRMS (ESI+): Found $[M+H]^+$ = 295.1693; $C_{20}H_{23}O_2$ requires 295.1693, Δ 0.29 ppm
IR (film) ν_{max}/cm^{-1} 3061, 2939, 2850, 1685, 1450, 1092, 751, 701.

***rac*-2-((2*S*,5*R*)-5-Methyltetrahydro-2*H*-pyran-2-yl)-1-phenylethan-1-one, 4am**

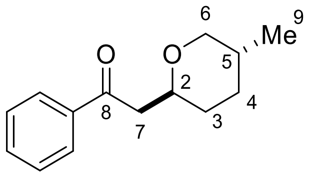

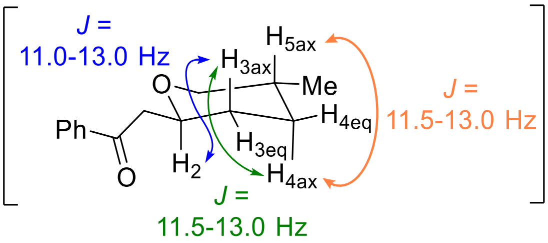

Phosphonate **S21** (358 mg, 1.4 equiv., 1.40 mmol), dry toluene (5 mL) 60% wt. NaH (48 mg, 1.2 eq., 1.2 mmol) and lactol **1a** (116 mg, 1 equiv., 1.00 mmol) were subjected to **General Procedure 3**. The title compound was afforded after purification by FCC (10% Et_2O/pentane) as an orange oil (197 mg, 0.902 mmol, 90% yield, 30:70 *cis*/*trans*, >95:5 r.r.).

^{1}H NMR (400 MHz, $CDCl_3$) δ 7.98 – 7.94 (m, 4H, major ArCH x 2 and minor ArCH x 2), 7.58 – 7.52 (m, 2H, major ArCH and minor ArCH), 7.48 – 7.42 (m, 4H, major ArCH x 2 and minor ArCH x 2), 4.03 – 3.95 (m, 1H, minor H_2), 3.91 – 3.80 (m, 2H, major H_2 and major H_{6eq}), 3.68 – 3.58 (m, 2H, minor H_6 x 2), 3.31 (dd, J = 16.0, 6.5 Hz, 1H, minor H_{7a}), 3.29 (dd, J = 16.0, 6.5 Hz, 1H, major H_{7a}), 3.04 (t, J = 11.0 Hz, 1H, major H_{6ax}), 2.98 (dd, J = 16.0, 6.0 Hz, 1H, minor H_{7b}), 2.93 (dd, J = 16.0, 6.0 Hz, 1H, major H_{7b}), 1.87 – 1.54 (m, 8H, major H_5, major H_{3eq}, major H_{4eq}, minor H_3 x 2, minor H_4 x 2 and minor H_5), 1.38 (tdd, J = 13.0, 11.0, 3.5 Hz, 1H, major H_{3ax}), 1.18 (tdd, J = 13.0, 11.5, 4.0 Hz, 1H, major H_{4ax}), 1.08 (d, J = 7.0 Hz, 3H, minor H_9 x 3), 0.78 (d, J = 6.5 Hz, 3H, major H_9 x 3).

^{13}C NMR (101 MHz, $CDCl_3$) δ 198.5, 198.5, 137.4, 137.4, 133.2, 128.7, 128.4, 128.4, 75.0, 74.2, 74.1, 72.9, 45.3, 44.9, 32.4, 32.1, 30.9, 28.9, 28.2, 27.1, 17.3, 16.8. N.B. the peaks at δ = 133.2 and 128.7 correspond to overlapping signals.

HRMS (ESI+): Found $[M+H]^+$ = 219.1380; $C_{14}H_{19}O_2$ requires 219.1380, Δ 0.41 ppm

IR (film) ν_{max}/cm^{-1} 2927, 2851, 1684, 1449, 1206, 1087, 751, 690.

rac-2-((2_S_,6_S_)-6-Methyltetrahydro-2_H_-pyran-2-yl)-1-phenylethan-1-one, 4an

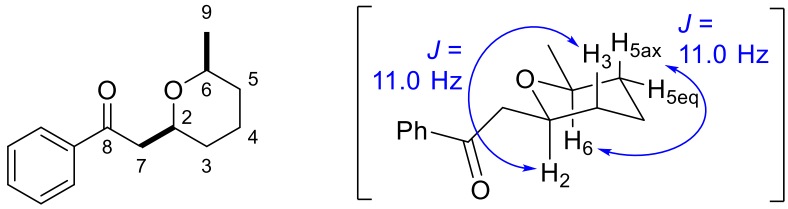

A 10–20 mL Biotage® microwave vial was charged with hept-6-en-2-ol[34] (103 mg, 0.902 mmol, 1 equiv.), 1-phenylprop-2-en-1-one[35] (238 mg, 1.80 mmol, 2 equiv.), 1,2-dichloroethane (9 mL, 0.1 M), and Hoveyda-Grubbs Catalyst® M720 (56 mg, 0.089 mmol, 10 mol%). The vial was flushed with nitrogen and then sealed with a microwave vial cap (containing a Reseal™ septum) and heated at 100 °C for 15 h. The reaction was then cooled to RT and concentrated *in vacuo*. The title compound was afforded after purification by FCC (10% Et_2O/pentane) as a yellow oil (172 mg, 0.788 mmol, 88% yield, >95:5 d.r.).

^{1}H NMR (400 MHz, $CDCl_3$) δ 8.03 – 7.92 (m, 2H, ArCH x 2), 7.59 – 7.51 (m, 1H, ArCH), 7.49 – 7.41 (m, 2H, ArCH x 2), 3.98 (dddd, J = 11.0, 6.5, 6.0, 2.0 Hz, 1H, H_2), 3.49 (dqd, J = 11.0, 6.0, 2.0 Hz, 1H, H_6), 3.31 (dd, J = 16.0, 6.0 Hz, 1H, H_{7a}), 2.97 (dd, J = 16.0, 6.5 Hz, 1H, H_{7b}), 1.87 – 1.68 (m, 2H, H_{4a}, H_{3a}), 1.61 – 1.54 (m, 2H, H_{4b}, H_{5a}), 1.30 – 1.16 (m, 2H, H_{3b}, H_{5b}), 1.14 (d, J = 6.0 Hz, 3H, H_9 x 3).

^{13}C NMR (101 MHz, $CDCl_3$) δ 198.6, 137.5, 133.1, 128.6, 128.4, 74.3, 74.2, 45.8, 33.3, 31.6, 23.7, 22.3.

HRMS (ESI+): Found $[M+H]^+$ = 219.1381 ; $C_{14}H_{19}O_2$ requires 219.1380, Δ 0.66 ppm

IR (film) ν_{max}/cm^{-1}: 2931, 2860, 1686, 1597, 1580, 1449.

rac-1-Phenyl-2-((2S,4R)-4-phenyltetrahydro-2H-pyran-2-yl)ethan-1-one, 4ao

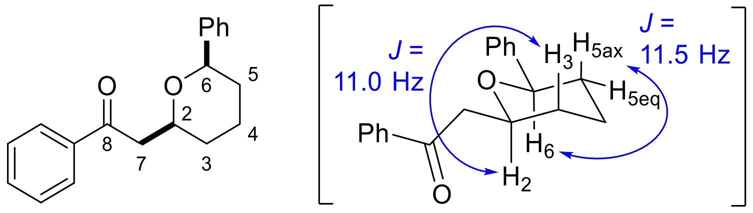

A 10–20 mL Biotage® microwave vial was charged with 1-phenylhex-5-en-1-ol[36] (159 mg, 0.902 mmol, 1 equiv.), 1-phenylprop-2-en-1-one[35] (238 mg, 1.80 mmol, 2 equiv.), 1,2-dichloroethane (9 mL, 0.1 M), and Hoveyda-Grubbs Catalyst® M720 (56 mg, 0.089 mmol, 10 mol%). The vial was flushed with nitrogen and then sealed with a microwave vial cap (containing a Reseal™ septum) and heated at 100 °C for 15 h. The reaction was then cooled to RT and concentrated *in vacuo*. The title compound was afforded after purification by FCC (10% Et_2O/pentane) as a yellow oil (202 mg, 0.720 mmol, 80% yield, >95:5 d.r.).

^1H NMR (400 MHz, $CDCl_3$) δ 7.95 – 7.85 (m, 2H, ArCH x 2), 7.50 – 7.41 (m, 1H, ArCH), 7.41 – 7.30 (m, 2H, ArCH x 2), 7.25 – 7.08 (m, 5H, ArCH x 5), 4.34 (dd, *J* = 11.5, 2.0 Hz, 1H, H_6), 4.09 (dtd, *J* = 11.0, 6.0, 2.0 Hz, 1H, H_2), 3.33 (dd, *J* = 16.0, 6.0 Hz, 1H, H_{7a}), 2.97 (dd, *J* = 16.0, 6.5 Hz, 1H, H_{7b}), 1.92 – 1.55 (m, 4H, H_4 x 2, H_{5a}, H_{3a}), 1.50 – 1.23 (m, 2H, H_{5b}, H_{3b}).

^{13}C NMR (101 MHz, $CDCl_3$) δ 198.6, 143.2, 137.5, 133.0, 128.5, 128.4, 128.2, 127.2, 125.8, 79.8, 75.0, 45.6, 33.3, 31.4, 23.9.

HRMS (ESI+): Found $[M+H]^+$ = 281.1536 ; $C_{19}H_{21}O_2$ requires 281.1536, Δ 0.07 ppm

IR (film) ν_{max}/cm^{-1}: 2935, 1683, 1597, 1449, 1341, 1284.

rac-1-Phenyl-2-((2S,6S)-4,4,6-trimethyltetrahydro-2H-pyran-2-yl)ethan-1-one, 4ap

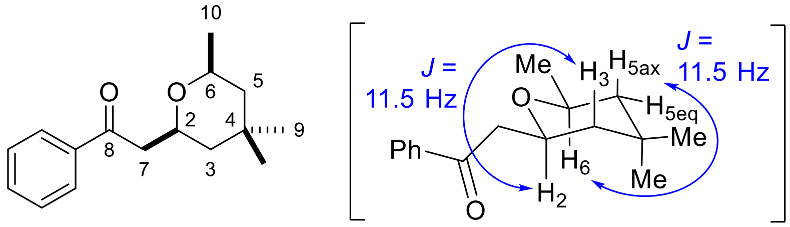

A 10–20 mL Biotage® microwave vial was charged with 4,4-dimethylhept-6-en-2-ol[37] (128 mg, 0.900 mmol, 1 equiv.), 1-phenylprop-2-en-1-one[35] (238 mg, 1.80 mmol, 2 equiv.), 1,2-dichloroethane (9 mL, 0.1 M), and Hoveyda-Grubbs Catalyst® M720 (56 mg, 0.089 mmol, 10 mol%). The vial was flushed with nitrogen and then sealed with a microwave vial cap (containing a Reseal™ septum) and heated at 100 °C for 15 h. The reaction was then cooled to RT and concentrated *in vacuo*. The title compound was afforded after purification by FCC (10% Et_2O/pentane) as a yellow oil (98 mg, 0.398 mmol, 44% yield, >95:5 d.r.).

^{1}H NMR (400 MHz, $CDCl_3$) δ 8.01 – 7.92 (m, 2H, ArCH x 2), 7.59 – 7.51 (m, 1H, ArCH), 7.50 – 7.40 (m, 2H, ArCH x 2), 4.16 (dtd, *J* = 11.5, 6.0, 2.0 Hz, 1H, H_2), 3.66 (dqd, *J* = 11.5, 6.0, 2.0 Hz, 1H, H_6), 3.31 (dd, *J* = 16.0, 6.0 Hz, 1H, H_{7a}), 2.91 (dd, *J* = 16.0, 6.5 Hz, 1H, H_{7b}), 1.46 (dt, *J* = 13.0, 2.0 Hz, 1H, H_{3a}), 1.30 (dt, *J* = 13.0, 2.0 Hz, 1H, H_{5a}), 1.17 – 1.08 (m, 5H, H_{3b}, H_{10} x 3, H_{5b}), 1.05 (s, 3H, H_{9a} x 3), 0.93 (s, 3H, H_{9b} x 3).

^{13}C NMR (101 MHz, $CDCl_3$) δ 198.7, 137.6, 133.1, 128.6, 128.4, 70.2, 69.7, 46.6, 45.7, 44.7, 33.2, 29.8, 25.0, 22.2.

HRMS (ESI+): Found $[M+H]^+$ = 247.1693; $C_{16}H_{23}O_2$ requires 247.1695, Δ 0.90 ppm

IR (film) ν_{max}/cm^{-1}: 2953, 2903, 1687, 1597, 1449, 1374.

2-(4,4-Dimethyltetrahydro-2*H*-pyran-2-yl)-1-phenylethan-1-one, 4aq

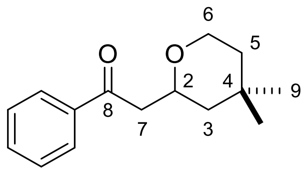

Method A: A 10–20 mL Biotage® microwave vial was charged with 3,3-dimethylhex-5-en-1-ol[38] (115 mg, 0.897 mmol, 1 equiv.), 1-phenylprop-2-en-1-one[35] (238 mg, 1.80 mmol, 2 equiv.), 1,2-dichloroethane (9 mL, 0.1 M), and Hoveyda-Grubbs Catalyst® M720 (56 mg, 0.089 mmol, 10 mol%). The vial was flushed with nitrogen and then sealed with a microwave vial cap (containing a Reseal™ septum) and heated at 100 °C for 15 h. The reaction was then cooled to RT and concentrated *in vacuo*. The title compound was afforded after purification by FCC (10% Et_2O/pentane) as a yellow oil (184 mg, 0.792 mmol, 88% yield).

Method B: Phosphonate **S21** (1.04 g, 1.4 equiv., 4.06 mmol), dry toluene (15 mL) 60% wt. NaH (140 mg, 1.2 eq., 3.48 mmol) and 4,4-dimethyltetrahydro-2*H*-pyran-2-ol (378 mg, 1 equiv., 2.90 mmol) were subjected to **General Procedure 3**. The title compound was afforded after purification by FCC (10% Et_2O/pentane) as a yellow oil (626 mg, 2.69 mmol, 93% yield).

^1H NMR (400 MHz, $CDCl_3$) δ 7.98 – 7.94 (m, 2H, ArCH x 2), 7.59 – 7.53 (m, 1H, ArCH), 7.49 – 7.43 (m, 2H, ArCH x 2), 4.18 – 4.07 (m, 1H, H_2), 3.80 (ddd, *J* = 12.0, 5.0, 1.5 Hz, 1H, H_{6eq}), 3.63 (ddd, *J* = 13.0, 12.0, 2.5 Hz, 1H, H_{6ax}), 3.28 (dd, *J* = 16.0, 7.0 Hz, 1H, H_{7a}), 2.86 (dd, *J* = 16.0, 5.5 Hz, 1H, H_{7b}), 1.54 – 1.43 (m, 2H, H_{3a} and H_{5a}), 1.28 – 1.19 (m, 2H, H_{3b} and H_{5b}), 1.06 (s, 3H, H_{9a} x 3), 0.96 (s, 3H, H_{9b} x 3).
^1H NMR (500 MHz, *p*-Xylene-d_{10}, 298 K) δ 7.98 – 7.92 (m, 2H, ArCH x 2), 7.32 – 7.25 (m, 1H, ArCH), 7.22 (m, 2H, ArCH x 2), 4.21 (dtd, *J* = 12.0, 6.0, 2.0 Hz, 1H, H_2), 3.76 (ddd, *J* = 11.5, 5.0, 1.5 Hz, 1H, H_{6eq}), 3.55 (ddd, *J* = 13.0, 11.5, 2.0 Hz, 1H, H_{6ax}), 3.20 (dd, *J* = 16.0, 6.5 Hz, 1H, H_{7a}), 2.62 (dd, *J* = 16.0, 6.0 Hz, 1H, H_{7b}), 1.52 – 1.41 (m, 2H, H_{3a} and H_{5eq}), 1.17 (dd, *J* = 13.0, 11.5 Hz, 1H, H_{5ax}), 1.07 – 1.01 (m, 2H, H_{3b}, H_{9a} x 3), 0.97 (s, 3H, H_{9b} x 3).
^{13}C NMR (101 MHz, $CDCl_3$) δ 198.5, 137.5, 133.2, 128.7, 128.4, 70.2, 64.6, 45.5, 45.1, 38.8, 33.3, 29.1, 24.1.
HRMS (ESI+): Found $[M+H]^+$ = 233.1537; $C_{15}H_{21}O_2$ requires 233.1536, Δ 0.48 ppm
IR (film) ν_{max}/cm^{-1} 2952, 1685, 1449, 1382, 1277, 1210, 1104, 1092, 753, 612.

1-(1,4(1,4)-dibenzenacyclohexaphane-1^2-yl)-2-(4,4-dimethyltetrahydro-2H-pyran-2-yl)ethan-1-one, 4ar

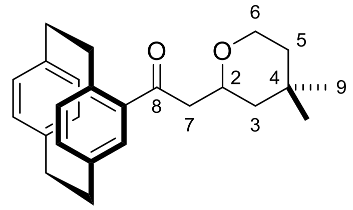

A 10–20 mL Biotage® microwave vial was charged with 3,3-dimethylhex-5-en-1-ol[38] (115 mg, 0.898 mmol, 1 equiv.), enone **S43** (472 mg, 1.80 mmol, 2 equiv.), 1,2-dichloroethane (9 mL, 0.1 M), and Hoveyda-Grubbs Catalyst® M720 (56 mg, 0.089 mmol, 10 mol%). The vial was flushed with nitrogen and then sealed with a microwave vial cap (containing a Reseal™ septum) and heated at 100 °C for 15 h. The reaction was then cooled to RT and concentrated *in vacuo*. The title compound was afforded after purification by FCC (10% Et_2O/pentane) as a yellow oil (107 mg, 0.295 mmol, 33% yield, 1:1 d.r.).
^1H NMR (400 MHz, $CDCl_3$) δ 7.01 (d, *J* = 1.9 Hz, 1H, ArCH), 6.89 (d, *J* = 1.9 Hz, 1H, ArCH), 6.63 (dd, *J* = 7.7, 1.8 Hz, 2H, ArCH x 2), 6.58 – 6.44 (m, 8H, ArCH x 8), 6.34 (ddd, *J* = 8.5, 7.0, 2.0 Hz, 2H, ArCH x 2), 4.20 – 4.01 (m, 2H, H_2 and $H_{2'}$), 3.97 – 3.85 (m, 3H, Cyclophane-CH_2 x 2 and H_{6a}), 3.79 (ddd, *J* = 12.0, 5.0, 1.5 Hz, 1H, $H_{6a'}$), 3.74 – 3.59 (m, 2H, H_{6b} and $H_{6b'}$), 3.30 –

2.94 (m, 14H, Cyclophane-CH_2 x 12, H_{7a} and $H_{7a'}$), 2.89 – 2.80 (m, 2H, Cyclophane-CH_2 x 2), 2.76 (dd, J = 16.0, 5.5 Hz, 1H, H_{7b}), 2.48 (dd, J = 15.5, 5.0 Hz, 1H, $H_{7b'}$), 1.61 – 1.32 (m, 4H, H_5 x 2 and $H_{5'}$ x 2), 1.31 – 1.14 (m, 4H, H_3 x 2 and $H_{3'}$ x 2), 1.08 (s, 3H, $H_{9a'}$ x 3), 1.07 (s, 3H, H_{9a} x 3), 0.98 (s, 3H, H_{9b} x 3), 0.96 (s, 3H, $H_{9b'}$ x 3).

^{13}C NMR (101 MHz, $CDCl_3$) δ 201.1, 200.6, 141.7, 141.5, 140.4, 139.9, 139.8, 139.3, 139.2, 138.3, 138.0, 136.5, 136.4, 136.3, 134.1, 133.6, 133.0, 133.0, 132.8, 132.8, 132.6, 132.2, 131.6, 131.3, 70.9, 70.2, 64.6, 64.5, 47.6, 47.5, 45.1, 45.0, 38.8, 38.8, 36.1, 36.1, 35.3, 35.3, 35.2, 35.2, 35.1, 33.4, 33.3, 29.2, 29.1, 24.2, 24.1. *N.B.* The peaks at δ = 140.4, 136.4, 35.2 ppm correspond to overlapping signals.

HRMS (ESI+): Found $[M-H]^+$ = 363.2320; $C_{25}H_{31}O_2$ requires 363.2319, Δ 0.30 ppm

IR (film) ν_{max}/cm^{-1}: 2951, 2925, 2854, 1674, 1593, 1552.

1-Phenyl-2-(tetrahydro-2*H*-pyran-2-yl)ethan-1-one, 4as

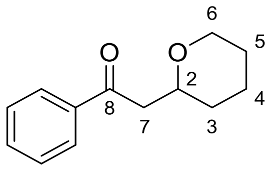

A 10–20 mL Biotage® microwave vial was charged with hex-5-en-1-ol (90 mg, 0.90 mmol, 1 equiv.), 1-phenylprop-2-en-1-one[35] (238 mg, 1.80 mmol, 2 equiv.), 1,2-dichloroethane (9 mL, 0.1 M), and Hoveyda-Grubbs Catalyst® M720 (56 mg, 0.09 mmol, 10 mol%). The vial was flushed with nitrogen and then sealed with a microwave vial cap (containing a Reseal™ septum) and heated at 100 °C for 15 h. The reaction was then cooled to RT and concentrated *in vacuo*. The title compound was afforded after purification by FCC (10% Et_2O/pentane) as a yellow oil (123 mg, 0.602 mmol, 67% yield). The spectral data matched that previously reported in the literature.[39]

^1H NMR (400 MHz, $CDCl_3$) δ 8.00 – 7.91 (m, 2H, ArCH x 2), 7.59 – 7.49 (m, 1H, ArCH), 7.49 – 7.39 (m, 2H, ArCH x 2), 4.00 – 3.89 (m, 2H, H_2, H_{6a}), 3.54 – 3.39 (m, 1H, H_{6b}), 3.28 (dd, J = 16.0, 6.5 Hz, 1H, H_{7a}), 2.91 (dd, J = 16.0, 6.0 Hz, 1H, H_{7b}), 1.82 (dddd, J = 11.0, 8.0, 4.5, 2.0 Hz, 1H, H_{4a}), 1.77 – 1.69 (m, 1H, H_{3a}), 1.64 – 1.44 (m, 3H, H_{4b}, H_5 x 2), 1.41 – 1.29 (m, 1H, H_{3b}).

^{13}C NMR (101 MHz, $CDCl_3$) δ 198.4, 137.4, 133.1, 128.6, 128.3, 74.4, 68.7, 45.5, 32.1, 26.0, 23.5.

7-Hydroxy-3-methyl-1-phenylhept-2-en-1-one, 4at

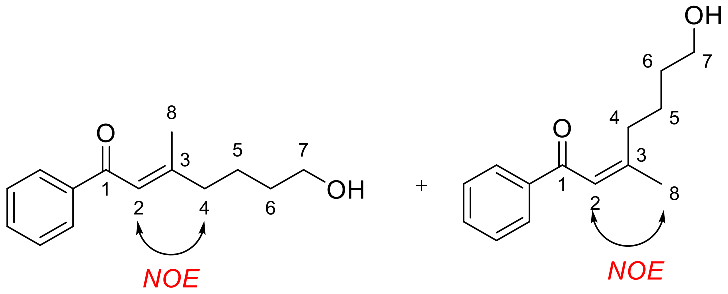

Major (E) *Minor (Z)*

A solution of MeLi (11.3 mL, 18 mmol, 1.6M, 6 equiv.) was added slowly to a solution of CuI (1.43 g, 7.5 mmol, 2.5 equiv.) in dry THF (30 mL, 0.1 M) cooled to –78 ^{o}C under nitrogen. The reaction mixture was warmed to 0 ^{o}C and stirred for 1 hour, which resulted in the formation of a colourless solution. The solution was cooled to –78 ^{o}C and a solution of hydroxyalkyne **S44** (606 mg, 3.00 mmol, 1 equiv.) in dry THF (5 mL) was added dropwise, and stirred for thirty minutes. The reaction was then quenched by the addition of sat. aq. ammonium chloride (30 mL), and then warmed to room temperature. The reaction mixture was then treated with 20% aq. ammonium hydroxide, which resulted in the formation of clear blue aqueous phase. The reaction mixture was then extracted with EtOAc (3 x 50 mL), and the organic phases were combined and dried over $MgSO_4$. The title compound was afforded after purification by FCC (combiflash rf, 10 -> 30% EtOAc/cyclohexane) as an inconsequential mixture of E/Z stereoisomers as a yellow oil (588 mg, 2.69 mmol, 90% yield, 67:33 *E*/*Z*).

^{1}H NMR (400 MHz, $CDCl_3$) δ 7.98 – 7.88 (m, 4H, major ArCH x 2, minor ArCH x 2), 7.56 – 7.49 (m, 2H, major ArCH, minor ArCH), 7.49 – 7.39 (m, 4H, major ArCH x 2, minor ArCH x 2), 6.79 – 6.69 (m, 2H, major H_2, minor H_2), 3.83 – 3.60 (m, 4H, major H_7 x 2, minor H_7 x 2), 2.62 (t, *J* = 8.0 Hz, 2H, minor H_4 x 2), 2.29 (t, *J* = 7.0 Hz, 2H, major H_4 x 2), 2.20 (s, 3H, major H_8 x 3), 2.03 (s, 3H, minor H_8 x 3), 1.78 – 1.55 (m, 8H, major H_5 x 2, major H_6 x 2, minor H_5 x 2, minor H_6 x 2). *N.B.* the enone isomers were characterised by 1D NOE analysis. OH signal was not observed.

^{13}C NMR (101 MHz, $CDCl_3$) δ 191.9, 191.4, 161.6, 159.9, 139.5, 139.3, 132.5, 132.4, 128.6, 128.4, 128.3, 121.1, 120.9, 62.7, 62.1, 41.3, 33.7, 32.4, 32.3, 26.1, 24.4, 24.0, 19.9. *N.B*. The peak at δ = 128.6 corresponds to two overlapping signals.

HRMS (ESI+): Found $[M+Na]^+$ = 241.1199; $C_{14}H_{18}O_2Na$ requires 241.1199, Δ 0.20 ppm

IR (film) ν_{max}/cm^{-1} 2934, 2864, 1674, 1448, 1083, 1047, 751, 690.

3-Butyl-7-hydroxy-1-phenylhept-2-en-1-one (4au) and 2-(2-butyltetrahydro-2*H*-pyran-2-yl)-1-phenylethan-1-one (4au')

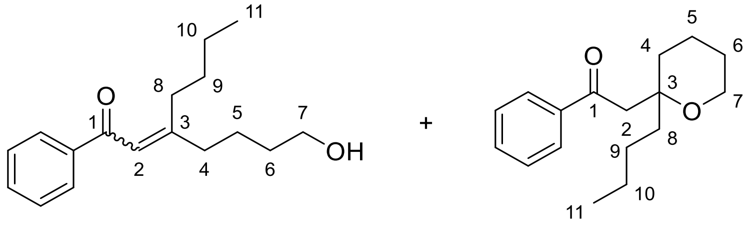

4au **4au'**

A solution of *n*-BuLi (1.2 mL, 3.0 mmol, 2.5M, 6 equiv.) was added slowly to a solution of CuI (286 mg, 1.50 mmol, 3 equiv.) in dry THF (5 mL, 0.1 M) cooled to –78 °C under nitrogen. The reaction mixture was warmed to 0 °C and stirred for 1 hour, which resulted in the formation of a grey precipitate. The solution was cooled to –78 °C and a solution of hydroxyalkyne **S44** (101 mg, 0.500 mmol, 1 equiv.) in dry THF (2 mL) was added dropwise, and stirred for thirty minutes. The reaction was then quenched by the addition of sat. aq. ammonium chloride (10 mL), and then warmed to room temperature. The reaction mixture was then treated with 20% aq. ammonium hydroxide, which resulted in the formation of clear blue aqueous phase. The reaction mixture was then extracted with EtOAc (3 x 30 mL), and the organic phases were combined and dried over $MgSO_4$. Purification by FCC (combiflash rf, 0 -> 30% EtOAc/cyclohexane) afforded **4au** (46 mg, 0.18 mmol, 35%, 66:34 ratio of alkene isomers) and **4au'** (55 mg, 0.21 mmol, 42%). *N.B.* we were unable to assign the identity (*E*/*Z*) of the geometrical isomers of **4au** due to overlapping ^{1}H NMR signals. **4au** and **4au'** were combined and this material was used in the aluminium mediated rearrangement.

Data for **4au** (66:34 d.r.):

^{1}H NMR (400 MHz, $CDCl_3$) δ 8.03 – 7.85 (m, 4H, major ArCH x 2, minor ArCH x 2), 7.57 – 7.41 (m, 6H, Major ArCH x 3, minor ArCH x 3), 6.81 – 6.62 (m, 2H, major H_2, minor H_2), 3.83 – 3.61 (m, 4H, major H_7 x 2, minor H_7 x 2), 2.72 – 2.51 (m, 4H, major H_8 x 2, minor H_8 x 2), 2.41 – 2.21 (m, 4H, major H_4 x 2, minor H_4 x 2), 1.72 – 1.59 (m, 8H, major H_5 x 2, major H_6 x 2, minor H_5 x 2, minor H_6 x 2), 1.60 – 1.33 (m, 8H, major H_{10} x 2, major H_9 x 2, minor H_{10} x 2 minor H_9 x 2), 1.05 – 0.82 (m, 6H, major H_{11} x 3, minor H_{11} x 3). *N.B.* OH signal was not observed.

^{13}C NMR (101 MHz, $CDCl_3$) δ 191.6, 191.6, 165.9, 164.4, 139.5, 139.5, 132.5, 132.4, 128.6, 128.6, 128.4, 128.3, 120.6, 120.1, 62.8, 61.9, 39.2, 38.7, 32.8, 32.6, 32.3, 31.0, 30.1, 24.7, 24.2, 23.2, 22.7, 14.1. N.B. The peaks at δ = 32.6 and 14.1 ppm correspond to overlapping signals.

HRMS (ESI+): Found $[M+H]^+$ = 261.1847; $C_{17}H_{25}O_2$ requires 261.1849, Δ -0.77 ppm

IR (film) ν_{max}/cm^{-1} 2933, 2861, 1677, 1448, 1349, 1084, 752, 690.

<u>Data for **4au'**:</u>

^{1}H NMR (400 MHz, $CDCl_3$) δ 8.00 – 7.93 (m, 2H, ArCH x 2), 7.58 – 7.50 (m, 1H, ArCH), 7.49 – 7.40 (m, 2H, ArCH x 2), 3.68 – 3.64 (m, 2H, H_7 x 2), 3.24 (d, *J* = 15.5 Hz, 1H, H_{2a}), 3.14 (d, *J* = 15.5 Hz, 1H, H_{2b}), 1.91 – 1.56 (m, 6H, H_4 x 2, H_5 x 2, H_8 x 2), 1.55 – 1.45 (m, 2H, H_6 x 2), 1.41 – 1.13 (m, 4H, H_9 x 2, H_{10} x 2), 0.86 (t, *J* = 7.0 Hz, 3H, H_{11} x 3).

^{13}C NMR (101 MHz, $CDCl_3$) δ 199.3, 138.6, 132.9, 128.6, 128.4, 75.4, 61.5, 44.8, 34.3, 33.7, 25.9, 25.4, 23.3, 19.2, 14.2.

HRMS (ESI+): Found $[M+H]^+$ = 261.1848; $C_{17}H_{25}O_2$ requires 261.1849, Δ -0.38 ppm

IR (film) ν_{max}/cm^{-1} 2933, 2860, 1677, 1448, 1350, 1085, 1000, 752, 690.

2-(4,4-Dimethyltetrahydro-2*H*-pyran-2-yl-2,6,6-*d₃*)-1-phenylethan-1-one, 4aq-*d₃*

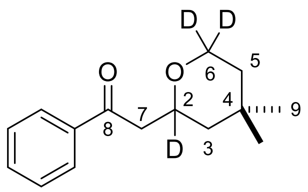

Phosphonate **S21** (269 mg, 1.4 equiv., 1.05 mmol), dry toluene (3.8 mL) 60% wt. NaH (36 mg, 1.2 eq., 0.90 mmol) and lactol **S19-*d₃*** (100 mg, 1 equiv., 0.425 mmol) were subjected to **General Procedure 3**. The title compound was afforded after purification by FCC (10% Et_2O/pentane) as a yellow oil (148 mg, 0.629 mmol, 84% yield, >95% D incorporation by ^{1}H and ^{13}C NMR).

^{1}H NMR (400 MHz, $CDCl_3$) δ 7.98 – 7.94 (m, 2H, ArCH x 2), 7.58 – 7.52 (m, 1H, ArCH), 7.47 – 7.42 (m, 2H, ArCH x 2), 3.27 (d, *J* = 16.0 Hz, 1H, H_{7a}), 2.84 (d, *J* = 16.0 Hz, 1H, H_{7b}), 1.51 – 1.42 (m, 2H, H_{3a} and H_{5a}), 1.27 – 1.17 (m, 2H, H_{3b} and H_{5b}), 1.05 (s, 3H, H_{9a} x 3), 0.95 (s, 3H, H_{9b} x 3). [N.B. in the absence of any deuteration, the peaks for H_2, H_{6a} and H_{6b} (see spectra) should each integrate to 1.00. Integration of the quantitative ^{1}H NMR spectrum showed an integral for each of these peaks of 0.02-0.03 thus indicating 0.97-0.98 D incorporation at each position, i.e. >95% D at each position].

^{13}C NMR (101 MHz, $CDCl_3$) δ 198.4, 137.3, 133.0, 128.5, 128.2, 69.6 (1:1:1 t, *J* = 21.4 Hz), 63.7 (1:2:3:2:1 quintet, *J* = 21.5 Hz), 38.4, 33.2, 29.0, 24.0.

^{2}H NMR (92 MHz, $CHCl_3$ + 1 drop $CDCl_3$) δ 4.12 (s, 1H, H_2), 3.76 (s, 1H, H_{6a}), 3.61 (s, 1H, H_{6b}).

The spectral data of **4aq-*d*₃** was consistent with that of **4aq** (see above) with >95% incorporation of deuterium at C2 and C6 (see spectra).

2-(4,4-Dimethyltetrahydro-2*H*-pyran-2-yl)-1-(3-methoxyphenyl)ethan-1-one, 4av

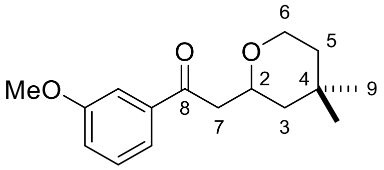

Phosphonate **S27** (362 mg, 1.4 equiv., 1.26 mmol), dry toluene (4.5 mL) 60% wt. NaH (43 mg, 1.2 eq., 1.1 mmol) and lactol **S19** (117 mg, 1 equiv., 0.900 mmol) were subjected to **General Procedure 3**. The title compound was afforded after purification by FCC (10% Et_2O/pentane) as a white solid (235 mg, 0.896 mmol, 100% yield).

^{1}H NMR (400 MHz, $CDCl_3$) δ 7.54 (ddd, *J* = 7.5, 1.5, 1.0 Hz, 1H, ArCH), 7.49 (dd, *J* = 2.5, 1.5 Hz, 1H, ArCH), 7.36 (t, *J* = 8.0 Hz, 1H, ArCH), 7.10 (ddd, *J* = 8.0, 2.5, 1.0 Hz, 1H, ArCH), 4.12 (dddd, *J* = 11.5, 7.0, 5.5, 2.0 Hz, 1H, H_2), 3.85 (s, 3H, OCH_3 x 3), 3.80 (ddd, *J* = 12.0, 5.0, 1.5 Hz, 1H, H_{6eq}), 3.63 (ddd, *J* = 13.0, 12.0, 2.5 Hz, 1H, H_{6ax}), 3.25 (dd, *J* = 16.0, 7.0 Hz, 1H, H_{7a}), 2.85 (dd, *J* = 16.0, 5.5 Hz, 1H, H_{7b}), 1.53 – 1.42 (m, 2H, H_{3a} and H_{5a}), 1.29 – 1.18 (m, 2H, H_{3b} and H_{5b}), 1.06 (s, 3H, H_{9a} x 3), 0.95 (s, 3H, H_{9b} x 3).

^{13}C NMR (101 MHz, $CDCl_3$) δ 198.4, 159.9, 138.8, 129.7, 121.1, 119.8, 112.5, 70.3, 64.6, 55.6, 45.6, 45.1, 38.8, 33.3, 29.1, 24.1.

HRMS (ESI+): Found $[M+H]^+$ = 263.1642; $C_{16}H_{23}O_3$ requires 263.1642, Δ 0.05 ppm

IR (film) ν_{max}/cm^{-1}: 2953, 1684, 1597, 1583, 1456, 1430, 1280, 1259, 1249, 1092.

m.p.: 36-38 °C

3-(4-Hydroxybutyl)-5,5-dimethylcyclohex-2-en-1-one 6

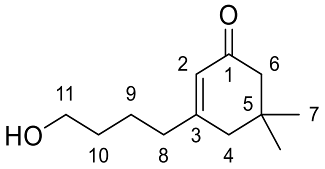

A solution of MeMgBr in THF (5.0 mL, 2M, 10 mmol, 2.0 eq.) was added to a solution of 4-chloro-1-butanol (814 mg, 7.5 mmol, 1.5 eq.) in THF (10 ml) at -78 °C under an argon atmosphere. The resulting solution was warmed to RT, and then magnesium turnings (243 mg, 10.0 mmol, 2.00 eq.) were added and then mixture was heated under reflux for 3 h. The

reaction mixture was then cooled to 0 °C and 3-ethoxy-5,5-dimethylcyclohex-2-en-1-one[40] (841 mg, 5.00 mmol, 1.00 eq.) was added dropwise, and stirred for 1.5 hours. The reaction mixture was then quenched with sat. aq. ammonium chloride (10 mL), diluted with 3M HCl (30 mL) and extracted with Et_2O (3 x 30 mL). The organic layers were washed with brine (50 mL), dried over $MgSO_4$, and concentrated *in vacuo.* Purification by FCC (30-60% EtOAc/pentane) afforded the product as a colourless oil (621 mg, 2.19 mmol, 44% yield).

^{1}H NMR (400 MHz, $CDCl_3$) δ 5.91 – 5.84 (m, 1H, H_2), 3.70 – 3.63 (m, 2H, H_{11} x 2), 2.26 – 2.15 (m, 6H, H_8 x 2, H_4 x 2, H_6 x 2), 1.67 – 1.52 (m, 4H, H_{10} x 2, H_9 x 2), 1.41 (s, 1H, OH), 1.03 (s, 6H, H_7 x 6).

^{13}C NMR (101 MHz, $CDCl_3$) δ 200.3, 164.0, 124.8, 62.4, 51.1, 44.0, 37.9, 33.7, 32.3, 28.4, 23.2.

HRMS (ESI+): Found $[M+H]^+$ = 197.1537; $C_{12}H_{21}O_2$ requires 197.1536, Δ 0.45 ppm

IR (film) ν_{max}/cm^{-1}: 3430 (broad), 2954, 1663, 1460, 1412, 1369

(4a*S*,8a*S*)-Octahydro-7*H*-chromen-7-one 8

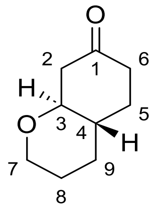

A 50 mL RBF was charged with 4-(2-ethoxytetrahydro-2H-pyran-3-yl)butan-2-one[41] (1.09 g, 5.50 mmol, 1.00 eq.), HCl (3M, 14 mL, 41 mmol, 7.5 eq.) and CH_2Cl_2 (28 mL). The resulting mixture was then heated under reflux for 3 h, extracted with CH_2Cl_2 (3 x 20 mL), dried over $MgSO_4$ and concentrated *in vacuo*. Purification by FCC (10-20% Et_2O/pentane) afforded the product as a white solid (270 mg, 1.75 mmol, 32% yield). The spectroscopic data matched that previously reported in the literature[41].

^{1}H NMR (400 MHz, $CDCl_3$) δ 4.03 – 3.95 (m, 1H, H_{7a}), 3.42 (td, *J* = 12.0, 2.5 Hz, 1H, H_{7b}), 3.12 (ddd, *J* = 12.0, 9.5, 5.0 Hz, 1H, H_3), 2.67 (ddt, *J* = 14.0, 5.0, 1.5 Hz, 1H, H_{2a}), 2.44 – 2.22 (m, 3H, H_{2b}, H_6 x 2), 1.97 – 1.48 (m, 5H, H_{8a}, H_{5a}, H_9 x 2, H_4), 1.34 – 1.12 (m, 2H, H_{5b}, H_{8b}).

^{13}C NMR (101 MHz, $CDCl_3$) δ 208.9, 79.6, 68.2, 48.3, 41.2, 40.7, 29.8, 28.2, 26.2.

3.2.2 Synthesis of Cyclohexenes

(3-Methylcyclohex-1-en-1-yl) (2,3,4,5,6-pentamethylphenyl)methanone, 2a, and (5-Methylcyclohex-1-en-1-yl)(2,3,4,5,6-pentamethylphenyl)methanone, 2a'

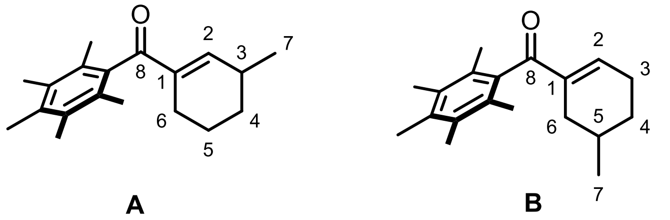

Method 1: A 5 mL microwave vial was charged with lactol **1a** (143 mg, 1.23 mmol, 2 eq.), 1-(2,3,4,5,6-pentamethylphenyl)ethan-1-one[9] (114 mg, 0.600 mmol, 1 eq.), KOH (134 mg, 2.40 mmol, 4 eq.) and toluene (2.4 mL, 0.25 M). The vial was then sealed and heated at 115 ^{o}C for 24 hours. After cooling to RT, the reaction mixture was diluted with 3M aq. HCl (6 mL) and extracted with CH_2Cl_2 (4 x 10 mL). The title compound was afforded after purification by FCC (5% Et_2O/pentane) as a white solid (114 mg, 0.420 mmol, 70% yield, inseparable regioisomeric mixture 75:25 of **A**:**B**).

Method 2: A 5mL microwave vial was charged with lactol **1b** (143 mg, 1.23 mmol, 2 eq.), 1-(2,3,4,5,6-pentamethylphenyl)ethan-1-one[9] (114 mg, 0.600 mmol, 1 eq.), KOH (134 mg, 2.40 mmol, 4 eq.) and toluene (2.4 mL, 0.25 M). The vial was then sealed and heated at 115 ^{o}C for 24 hours. After cooling to RT, the reaction mixture was diluted with 3M aq. HCl (6 mL) and extracted with CH_2Cl_2 (4 x 10 mL). The title compound was afforded after purification by FCC (5% Et_2O/pentane) as a white solid (120 mg, 0.444 mmol, 74% yield, inseparable regioisomeric mixture 57:43 of **A**:**B**).

NMR data for major regioisomer (**A**): ^{1}H NMR (400 MHz, $CDCl_3$) δ 6.37 – 6.29 (m, 1H, H_2), 2.53 – 2.40 (m, 1H, H_{6a}), 2.38 – 2.15 (m, 11H, H_3, H_{6b}, $ArCH_3$ x 6), 2.01 (s, 6H, $ArCH_3$ x 2), 1.92 – 1.77 (m, 2H, H_{4a}, H_{5a}), 1.67 – 1.49 (m, 1H, H_{4b}), 1.32 – 1.15 (m, 1H, H_{5b}), 0.97 (d, *J* = 7.0 Hz, 3H, H_7 x 3). The minor regioisomer displays diagnostic signals at δ = 6.50 – 6.45 (m, 1H, H_2), 2.74 – 2.62 (m, 1H, H_{6a}), 1.77 – 1.66 (m, 1H, H_{5a} or H_{4a}), 1.06 (d, *J* = 6.4 Hz, 3H, H_7 x 3).

^{13}C NMR (101 MHz, $CDCl_3$) δ 204.1, 150.7, 139.8, 138.3, 135.0, 132.7, 129.3, 129.2, 31.3, 30.5, 22.6, 20.9, 20.6, 17.6, 17.6, 16.8, 16.1. N.B. additional peaks for C_6Me_5 were observed due to restricted rotation about the C_1-C(O) axis. The minor regioisomer displays diagnostic signals at δ = 145.2, 140.3, 21.6.

HRMS (ESI+): Found $[M+H]^+$ = 271.2054; $C_{19}H_{27}O$ requires 271.2056, Δ –0.95 ppm

IR (film) ν_{max}/cm^{-1} 2926, 2856, 1654, 1634, 1455, 1308, 1268, 1192

m.p.: 96-98 °C

(2,3,4,5,6-Pentamethylphenyl)(1,2,3,6-tetrahydro-[1,1'-biphenyl]-4-yl)methanone, 2c

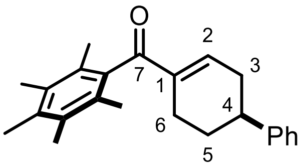

A 5mL microwave vial was charged with lactol **1c** (214 mg, 1.20 mmol, 2 eq.), 1-(2,3,4,5,6-pentamethylphenyl)ethan-1-one[9] (114 mg, 0.600 mmol, 1 eq.), KOH (134 mg, 2.40 mmol, 4 eq.) and toluene (2.4 mL, 0.25 M). The vial was then sealed and heated at 115 °C for 24 hours. After cooling to RT, the reaction mixture was diluted with 3M aq. HCl (6 mL) and extracted with CH_2Cl_2 (4 x 10 mL). The title compound was afforded after purification by FCC (5% Et_2O/pentane) as a white solid (140 mg, 0.421 mmol, 70% yield, 58:42 e.r).

^{1}H NMR (400 MHz, $CDCl_3$) δ 7.35 – 7.28 (m, 2H, ArCH x 2), 7.25 – 7.18 (m, 3H, ArCH x 3), 6.62 – 6.54 (m, 1H, H_2), 2.91 – 2.80 (m, 1H, H_4), 2.80 – 2.69 (m, 1H, H_{6a}), 2.54 – 2.36 (m, 2H, H_{6b}, H_{3a}), 2.37 – 2.26 (m, 1H, H_{3b}), 2.25 (s, 3H, $ArCH_3$), 2.20 (s, 6H, $ArCH_3$ x 2), 2.12 (dddt, *J* = 11.5, 5.5, 2.5, 1.5 Hz, 1H, H_{5a}), 2.08-2.03 (m, 6H, $ArCH_3$ x 2), 1.86 – 1.72 (m, 1H, H_{5b}).

^{13}C NMR (101 MHz, $CDCl_3$) δ 203.3, 146.1, 144.6, 140.6, 138.3, 135.2, 132.8, 129.3, 129.2, 128.7, 127.0, 126.5, 39.6, 34.4, 29.5, 23.2, 17.6, 16.8, 16.1. N.B. additional peaks for C_6Me_5 were observed due to restricted rotation about the C_1-C(O) axis

HRMS (ESI+): Found $[M+H]^+$ = 333.2214; $C_{24}H_{29}O$ requires 333.2213, Δ 0.30 ppm

IR (film) ν_{max}/cm^{-1} 2931, 1650, 1635, 1451, 1428, 1379, 1311, 1265, 1181, 852, 756, 699

m.p.: 160-162 °C

HPLC: Enantiomeric excess was determined by HPLC with a Chiralpak® IA column (99:1 hexane:IPA, 1.0 mL min^{-1}, 254 nm, room temperature); t_r (minor) = 11.2 min, t_r (major) = 12.6 min.

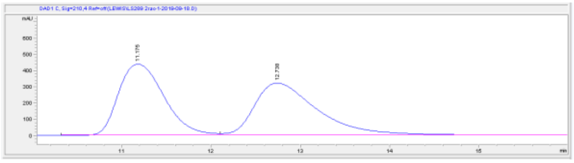

#	Time	Type	Area	Height	Width	Area%	Symmetry
1	11.175	BV	15517.3	437.3	0.5679	49.095	0.693
2	12.738	VB	16089.4	321.1	0.7506	50.905	0.504

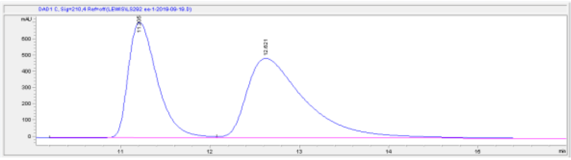

#	Time	Type	Area	Height	Width	Area%	Symmetry
1	11.205	BV	16088	711	0.35	41.528	0.586
2	12.621	VB	22651.9	490.3	0.685	58.472	0.432

(4-Methylcyclohex-1-en-1-yl)(phenyl)methanone, 2d

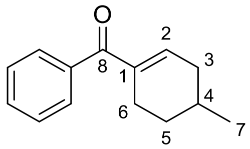

Method 1: Tetrahydropyran **4d** (65 mg, 0.30 mmol, 1 equiv.), basic alumina (150 mg, 5 equiv. 1.50 mmol), dry PhMe (1.5 mL, 0.2 M) were subjected to **General Procedure 4**. The title compound was afforded after purification by FCC (5% Et_2O/pentane) as a colourless oil (58 mg, 0.29 mmol, 96% yield).

Method 2: A 10 mL flame dried RBF fitted with a reflux condenser was charged with phosphonate **S21** (180 mg, 0.700 mmol, 1.4 equiv.) and the flask was evacuated and backfilled with N_2 three times. Dry toluene (2.5 mL) was added, and 60% wt. NaH (24 mg, 0.60 mmol, 1.2 equiv.) was added in one portion [care: vigorous release of H_2]. The resulting suspension was stirred at RT for 20 min, by which time a homogeneous solution had formed. Lactol **2d** (58 mg, 0.50 mmol, 1.0 equiv.) was added and the reaction was heated at 110 °C for 15 h. The reaction was then cooled to RT, and water (3 mL) was added. The resulting mixture was extracted with a 1:1 mixture of pentane/Et_2O (5 x 3 mL) and the combined organic extracts were filtered through a short pad of silica gel, washing with three further 3 mL portions of 1:1 pentane/Et_2O. The filtrate was concentrated under reduced pressure and the residue was dissolved in dry PhMe (2.5 mL) and transferred to a flame dried 2–5 mL Biotage® microwave vial and aluminium *tert*-butoxide (185 mg, 0.750 mmol 1.5 equiv.) was added. The headspace was briefly purged with N_2 and the reaction vessel was sealed with a microwave vial cap (containing a Reseal™ septum) and heated at 110 °C for 16 h. The reaction was then cooled to RT, diluted with Et_2O (50 mL), filtered through a plug of silica gel and concentrated *in vacuo*. The title compound was afforded after purification by FCC (5% Et_2O/pentane) as a colourless oil (72 mg, 0.36 mmol, 72% yield over 2 steps).

Method 3: A solution of trimethylsilyl trifluoromethanesulfonate (7 mg, 0.03 mmol, 0.1 equiv.) in CH_2Cl_2 (1 mL) was added dropwise to a Schlenk tube charged with 4-methyltetrahydro-2*H*-pyran-2-yl acetate[42] (52 mg, 0.33 mmol, 1.1 equiv.), 1-phenyl-1-trimethylsiloxyethylene (58 mg, 0.3 mmol, 1 equiv.) and CH_2Cl_2 (0.5 mL) cooled to –78 °C under argon. The reaction mixture was stirred for 3 hours, and then warmed to RT. Xylene (1.5 mL, 0.2 M), and alumina (600 mg, 6 mmol, 20 equiv.) were added, and the then the vessel

was heated to 110 ^{o}C for 15 hours. The reaction mixture was then cooled to RT, filtered through silica, washing with Et_2O and concentrated *in vacuo*. The title compound was afforded after purification by FCC (5% Et_2O/pentane) as a colourless oil (40 mg, 0.20 mmol, 67% yield).

^{1}H NMR (400 MHz, $CDCl_3$) δ 7.66 – 7.59 (m, 2H, ArCH x 2), 7.53 – 7.44 (m, 1H, ArCH), 7.45 – 7.36 (m, 2H, ArCH x 2), 6.59 – 6.51 (m, 1H, H_2), 2.67 – 2.53 (m, 1H, H_{6a}), 2.42 – 2.22 (m, 2H, H_{3a}, H_{6b}), 1.94 – 1.80 (m, 2H, H_{3b}, H_{5a}), 1.82 – 1.66 (m, 1H, H_4), 1.30 (dtd, *J* = 12.0, 10.0, 5.0 Hz, 1H, H_{5b}), 1.02 (d, *J* = 6.5 Hz, 3H, H_7 x 3).

^{13}C NMR (101 MHz, $CDCl_3$) δ 198.3, 143.7, 138.9, 138.6, 131.4, 129.3, 128.1, 34.7, 30.4, 27.9, 24.2, 21.7.

HRMS (ESI+): Found $[M+H]^+$ = 201.1275; $C_{14}H_{17}O$ requires 201.1274, Δ 0.71 ppm

IR (film) ν_{max}/cm^{-1} 3000, 2886, 1725, 1685, 1380, 1252, 1154, 1088, 954, 752.

(4-Methylcyclohex-1-en-1-yl)(2,3,4,5,6-pentamethylphenyl)methanone, 2e

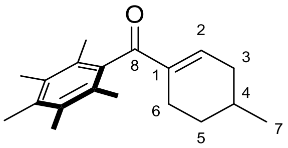

Tetrahydropyran **4e** (87 mg, 0.30 mmol, 1 equiv.), Al(O^{t}Bu)$_3$ (74 mg, 1 equiv. 0.30 mmol), dry PhMe (1.5 mL, 0.2 M) were subjected to **General Procedure 4**, and heated for 48 hours. The title compound was afforded after purification by FCC (5% Et_2O/pentane) as a colourless oil (79 mg, 0.29 mmol, 97% yield).

^{1}H NMR (400 MHz, $CDCl_3$) δ 6.49 – 6.43 (m, 1H, H_2), 2.70 – 2.58 (m, 1H, H_{6a}), 2.35 – 2.11 (m, 11H, H_{3a}, H_{6b} and $ArCH_3$ x 9), 2.01 (s, 6H, $ArCH_3$ x 6), 1.90 – 1.63 (m, 3H, H_{3b}, H_4 and H_{5a}), 1.27 (dtd, *J* = 13.0, 10.5, 5.5 Hz, 1H, H_{5b}), 0.99 (d, *J* = 6.5 Hz, 3H, H_7).

^{13}C NMR (101 MHz, $CDCl_3$) δ 203.5, 145.2, 140.5, 138.4, 135.0, 132.7, 129.2, 34.8, 30.4, 28.0, 22.5, 21.5, 17.6, 16.8, 16.1.

HRMS (ESI+): Found $[M+H]^+$ = 271.2055; $C_{19}H_{27}O$ requires 271.2056, Δ –0.39 ppm.

IR (film) ν_{max}/cm^{-1} 2931, 1652, 1633, 1192, 1168, 700.

m.p.: 143-144 ^{o}C.

(4-Methylcyclohex-1-en-1-yl)(3-nitrophenyl)methanone, 2f

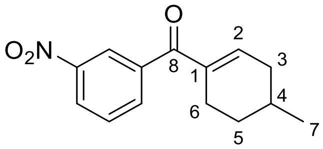

Tetrahydropyran **4f** (79 mg, 0.30 mmol, 1 equiv.), $Al(O^tBu)_3$ (74 mg, 1 equiv. 0.30 mmol), dry PhMe (1.5 mL, 0.2 M) were subjected to **General Procedure 4**. The title compound was afforded after purification by FCC (10% Et_2O/pentane) as a colourless oil (55 mg, 0.22 mmol, 75% yield).

^1H NMR (400 MHz, $CDCl_3$) δ 8.43 (t, *J* = 2.0 Hz, 1H, ArCH), 8.35 (ddd, *J* = 8.0, 2.5, 1.0 Hz, 1H, ArCH), 7.94 (dt, *J* = 7.5, 1.5 Hz, 1H, ArCH), 7.62 (t, *J* = 7.9 Hz, 1H, ArCH), 6.62 – 6.50 (m, 1H, H_2), 2.68 – 2.55 (m, 1H, H_{6a}), 2.45 – 2.28 (m, 2H, H_{3a}, H_{6b}), 1.98 – 1.83 (m, 2H, H_{3b}, H_{5a}), 1.82 – 1.69 (m, 1H, H_4), 1.39 – 1.23 (m, 1H, H_{5b}), 1.03 (d, *J* = 6.5 Hz, 3H, H_7 x 3).

^{13}C NMR (101 MHz, $CDCl_3$) δ 195.4, 148.0, 145.6, 140.4, 138.4, 134.9, 129.5, 125.8, 124.0, 34.8, 30.2, 27.9, 24.0, 21.6.

HRMS (ESI+): Found $[M+H]^+$ = 246.1127; $C_{14}H_{16}O_3N$ requires 246.1125, Δ 0.75 ppm

IR (film) ν_{max}/cm^{-1} 2981, 2886, 1650, 1531, 1381, 1253, 1152, 1073, 954.

4-(4-Methylcyclohex-1-ene-1-carbonyl)benzonitrile, 2g

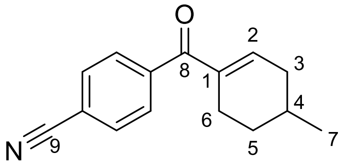

Tetrahydropyran **4g** (73 mg, 0.30 mmol, 1 equiv.), $Al(O^tBu)_3$ (74 mg, 1 equiv. 0.30 mmol), dry PhMe (1.5 mL, 0.2 M) were subjected to **General Procedure 4**.The title compound was afforded after purification by FCC (10% Et_2O/pentane) as a white solid (50 mg, 0.222 mmol, 74% yield).

^1H NMR (400 MHz, $CDCl_3$) δ 7.77 – 7.63 (m, 4H, ArCH x 4), 6.57 – 6.48 (m, 1H, H_2), 2.66 – 2.53 (m, 1H, H_{3a}), 2.48 – 2.24 (m, 2H, H_{3b}, H_{6a}), 1.95 – 1.82 (m, 2H, H_{5a}, H_{6b}), 1.81 – 1.67 (m, 1H, H_4), 1.37 – 1.23 (m, 1H, H_{5b}), 1.02 (d, *J* = 6.5 Hz, 3H, H_7 x 3).

^{13}C NMR (101 MHz, $CDCl_3$) δ 196.3, 145.8, 142.9, 138.5, 132.1, 129.5, 118.3, 114.7, 34.8, 30.2, 27.8, 23.8, 21.6.

HRMS (ESI+): Found $[M+H]^+$ = 226.1228; $C_{15}H_{16}ON$ requires 226.1226, Δ 0.72 ppm

IR (film) v_{max}/cm^{-1} 2998, 2981, 2886, 2230, 1648, 1381, 1263, 1154, 1082, 942, 878.

m.p.: 94-95 ^oC

Methyl 4-(4-methylcyclohex-1-ene-1-carbonyl)benzoate, 2h

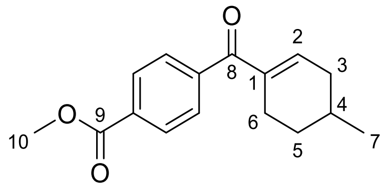

Tetrahydropyran **4h** (83 mg, 0.30 mmol, 1 equiv.), Al(O*t*Bu)$_3$ (74 mg, 1 equiv. 0.30 mmol), dry PhMe (1.5 mL, 0.2 M) were subjected to **General Procedure 4**. The title compound was afforded after purification by FCC (5-10% Et_2O/pentane) as a white solid (70 mg, 0.27 mmol, 90% yield).

^1H NMR (400 MHz, $CDCl_3$) δ 8.12 – 8.03 (m, 2H, ArCH x 2), 7.69 – 7.60 (m, 2H, ArCH x 2), 6.58 – 6.52 (m, 1H, H_2), 3.94 (s, 3H, H_{10} x 3), 2.66 – 2.54 (m, 1H, H_{6a}), 2.42 – 2.25 (m, 2H, H_{3a}, H_{6b}), 1.94 – 1.82 (m, 2H, H_{3b}, H_{5a}), 1.82 – 1.66 (m, 1H, H_4), 1.37 – 1.22 (m, 1H, H_{5b}), 1.02 (d, *J* = 6.5 Hz, 3H, H_7 x 3).

^{13}C NMR (101 MHz, $CDCl_3$) δ 197.4, 166.6, 145.2, 143.0, 138.7, 132.4, 129.4, 129.0, 52.5, 34.8, 30.3, 27.9, 23.9, 21.6.

HRMS (ESI+): Found $[M+H]^+$ = 259.1330; $C_{16}H_{19}O_3$ requires 259.1329, Δ 0.48 ppm

IR (film) v_{max}/cm^{-1} 2948, 2923, 1725, 1632, 1279, 1107, 747, 719.

m.p.: 94-95 ^oC

(3-Methoxyphenyl)(4-methylcyclohex-1-en-1-yl)methanone, 2i

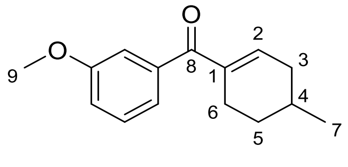

Tetrahydropyran **4i** (75 mg, 0.30 mmol, 1 equiv.), basic alumina (150 mg, 5 equiv., 1.50 mmol), dry PhMe (1.5 mL, 0.2 M) were subjected to **General Procedure 4**. The title compound was afforded after purification by FCC (5% Et_2O/pentane) as a colourless oil (59 mg, 0.26 mmol, 85% yield).

^1H NMR (400 MHz, $CDCl_3$) δ 7.35 – 7.27 (m, 1H, ArCH), 7.21 – 7.13 (m, 2H, ArCH x 2), 7.03 (ddd, *J* = 8.0, 2.5, 1.0 Hz, 1H, ArCH), 6.61 – 6.53 (m, 1H, H_2), 3.84 (s, 3H, H_9 x 3), 2.65 – 2.52 (m, 1H,

H_{6a}), 2.41 – 2.24 (m, 2H, H_{3a}, H_{6b}), 1.94 – 1.82 (m, 2H, H_{3b}, H_{5a}), 1.80 – 1.67 (m, 1H, H_4), 1.29 (dtd, *J* = 13.0, 11.0, 5.5 Hz, 1H, H_{5b}), 1.01 (d, *J* = 6.5 Hz, 3H, H_7 x 3).

^{13}C NMR (101 MHz, $CDCl_3$) δ 198.0, 159.5, 143.8, 140.2, 138.5, 129.1, 121.8, 117.5, 114.0, 55.5, 34.7, 30.4, 27.9, 24.2, 21.7.

HRMS (ESI+): Found $[M+H]^+$ = 231.1380; $C_{15}H_{19}O_2$ requires 231.1380, Δ 0.26 ppm

IR (film) ν_{max}/cm^{-1} 2952, 2870, 1647, 1579, 1430, 1268, 1233, 1048, 803.

[1,1'-Biphenyl]-2-yl(4-methylcyclohex-1-en-1-yl)methanone, 2j

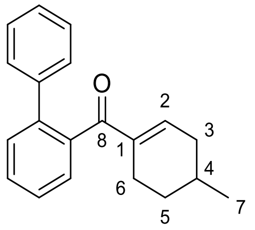

Tetrahydropyran **4j** (88 mg, 0.30 mmol, 1 equiv.), basic alumina (150 mg, 5 equiv., 1.50 mmol), dry PhMe (1.5 mL, 0.2 M) were subjected to **General Procedure 4**. The title compound was afforded after purification by FCC (2% Et_2O/pentane) as a white solid (73 mg, 0.26 mmol, 88% yield).

^{1}H NMR (400 MHz, $CDCl_3$) δ 7.49 – 7.09 (m, 9H, ArCH x 9), 6.12 – 5.99 (m, 1H, H_2), 2.23 (ddtd, *J* = 17.5, 5.5, 2.5, 1.0 Hz, 1H, H_{6a}), 2.00 – 1.85 (m, 2H, H_{3a}, H_{6b}), 1.57 – 1.34 (m, 2H, H_{3b}, H_{5a}), 1.34 – 1.22 (m, 1H, H_4), 0.83 (dtd, *J* = 13.0, 10.5, 5.5 Hz, 1H, H_{5b}), 0.71 (d, *J* = 6.5 Hz, 3H, H_7 x 3).

^{13}C NMR (101 MHz, $CDCl_3$) δ 200.7, 144.7, 141.4, 140.9, 140.0, 139.7, 129.9, 129.5, 128.8, 128.5, 128.5, 127.3, 127.3, 34.4, 30.0, 27.5, 23.1, 21.3.

HRMS (ESI+): Found $[M+H]^+$ = 277.1859; $C_{20}H_{21}O$ requires 277.1857, Δ 0.77 ppm

IR (film) ν_{max}/cm^{-1} 2951, 1651, 1636, 1474, 1275, 1078, 747, 699.

m.p.: 90-91 ^oC

(4-Methylcyclohex-1-en-1-yl)(naphthalen-2-yl)methanone, 2k

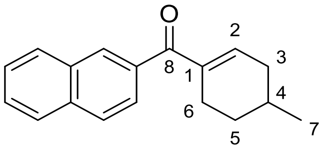

Tetrahydropyran **4k** (81 mg, 0.30 mmol, 1 equiv.), basic alumina (150 mg, 5 equiv., 1.50 mmol), dry PhMe (1.5 mL, 0.2 M) were subjected to **General Procedure 4**. The title compound was afforded after purification by FCC (4% Et_2O/pentane) as a colourless oil (49 mg, 0.20 mmol, 65% yield).

^{1}H NMR (400 MHz, $CDCl_3$) δ 8.12 (dt, *J* = 1.5, 0.5 Hz, 1H, ArCH), 7.95 – 7.83 (m, 3H, ArCH x 3), 7.75 (dd, *J* = 8.5, 1.5 Hz, 1H, ArCH), 7.60 – 7.50 (m, 2H, ArCH x 2), 6.61 (qd, *J* = 3.1, 1.2 Hz, 1H, H_2), 2.72 – 2.60 (m, 1H, H_{6a}), 2.49 – 2.31 (m, 2H, H_{3a}, H_{6b}), 1.98 – 1.85 (m, 2H, H_{3b}, H_{5a}), 1.85 – 1.71 (m, 1H, H_4), 1.41 – 1.28 (m, 1H, H_{5b}), 1.04 (d, *J* = 6.5 Hz, 3H, H_7 x 3).

^{13}C NMR (101 MHz, $CDCl_3$) δ 198.3, 143.6, 138.8, 136.1, 134.9, 132.4, 130.2, 129.2, 128.1, 127.9, 127.8, 126.7, 125.9, 34.7, 30.5, 28.0, 24.3, 21.7.

HRMS (ESI+): Found $[M+H]^+$ = 251.1431; $C_{18}H_{19}O_2$ requires 251.1430, Δ 0.28 ppm

IR (film) ν_{max}/cm^{-1} 3057, 2950, 2870, 1647, 1453, 1381, 1277, 1135, 817, 755.

Furan-2-yl(4-methylcyclohex-1-en-1-yl)methanone, 2l

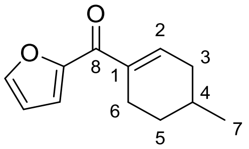

Tetrahydropyran **4l** (81 mg, 0.30 mmol, 1 equiv.), basic alumina (150 mg, 5 equiv., 1.50 mmol), dry PhMe (1.5 mL, 0.2 M) were subjected to **General Procedure 4**, heating for 8 hours. The title compound was afforded after purification by FCC (5-10% Et_2O/pentane) as a colourless oil (48 mg, 0.25 mmol, 84% yield).

^{1}H NMR (400 MHz, $CDCl_3$) δ 7.59 (dd, *J* = 1.5, 1.0 Hz, 1H, ArCH), 7.06 (dd, *J* = 3.5, 1.0 Hz, 1H, ArCH), 6.92 (ddt, *J* = 5.0, 3.0, 1.0 Hz, 1H, H_2), 6.49 (dd, *J* = 3.5, 1.5 Hz, 1H, ArCH), 2.60 – 2.49 (m, 1H, H_{6a}), 2.44 – 2.22 (m, 2H, H_{3a}, H_{6b}), 1.95 – 1.65 (m, 3H, H_{3b}, H_4, H_{5a}), 1.27 (dtd, *J* = 13.0, 11.0, 5.5 Hz, 1H, H_{5b}), 1.00 (d, *J* = 6.5 Hz, 3H, H_7 x 3).

^{13}C NMR (101 MHz, $CDCl_3$) δ 183.9, 152.4, 146.3, 140.5, 138.1, 118.7, 111.7, 34.6, 30.3, 27.9, 24.2, 21.7.

HRMS (ESI+): Found $[M+H]^+$ = 191.1067 $C_{12}H_{15}O_2$ requires 191.1067, Δ 0.41 ppm

IR (film) ν_{max}/cm^{-1} 2999, 2886, 1634, 1380, 1252, 1152, 1072, 953, 831.

(4-(Dimethylamino)phenyl)(4-methylcyclohex-1-en-1-yl)methanone, 2m

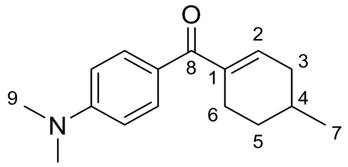

Tetrahydropyran **4m** (78 mg, 0.30 mmol, 1 equiv.), Al(O^{t}Bu)$_3$ (74 mg, 1 equiv. 0.30 mmol), dry PhMe (1.5 mL, 0.2 M) were subjected to **General Procedure 4**. The title compound was afforded after purification by FCC (15% Et_2O/pentane) as a colourless oil (56 mg, 0.23 mmol, 76% yield).

^{1}H NMR (400 MHz, $CDCl_3$) δ 7.75 – 7.66 (m, 2H, ArCH x 2), 6.69 – 6.61 (m, 2H, ArCH x 2), 6.36 (tdd, *J* = 3.5, 2.5, 1.5 Hz, 1H, H_2), 3.04 (s, 6H, H_9 x 6), 2.61 – 2.46 (m, 1H, H_{6a}), 2.43 – 2.26 (m, 2H, H_{3a}, H_{6b}), 1.93 – 1.69 (m, 3H, H_{3b}, H_4, H_{5a}), 1.31 (dtd, *J* = 13.0, 10.5, 5.5 Hz, 1H, H_{5b}), 1.02 (d, *J* = 6.5 Hz, 3H, H_7 x 3).

^{13}C NMR (101 MHz, $CDCl_3$) δ 197.1, 153.0, 138.6, 138.3, 132.0, 125.8, 110.7, 40.3, 34.3, 30.7, 28.1, 25.0, 21.8.

HRMS (ESI+): Found $[M+H]^+$ = 244.1698; $C_{16}H_{22}ON$ requires 244.1696, Δ 0.92 ppm

IR (film) ν_{max}/cm^{-1} 2995, 2921, 2877, 1631, 1593, 1526, 1366, 1271, 1189, 1122, 821.

(4-Methylcyclohex-1-en-1-yl)(pyridin-3-yl)methanone, 2n

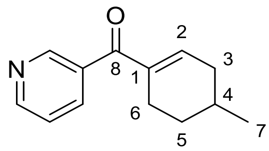

Tetrahydropyran **4n** (66 mg, 0.30 mmol, 1 equiv.), basic alumina (150 mg, 5 equiv., 1.50 mmol), dry PhMe (1.5 mL, 0.2 M) were subjected to **General Procedure 4**. The title compound was afforded after purification by FCC (60-70% Et_2O/pentane) as a colourless oil (33 mg, 0.17 mmol, 55% yield).

^{1}H NMR (400 MHz, $CDCl_3$) δ 8.81 (dd, *J* = 2.0, 1.0 Hz, 1H, ArCH), 8.70 (dd, *J* = 5.0, 2.0 Hz, 1H, ArCH), 7.92 (dt, *J* = 8.0, 2.0 Hz, 1H, ArCH), 7.42 – 7.27 (m, 1H, ArCH), 6.61 – 6.54 (m, 1H, H_2), 2.65 – 2.53 (m, 1H, H_{6a}), 2.42 – 2.25 (m, 2H, H_{3a}, H_{6b}), 1.96 – 1.80 (m, 2H, H_{3b}, H_{5a}), 1.80 – 1.67 (m, 1H, H_4), 1.29 (dtd, *J* = 13.0, 11.0, 5.5 Hz, 1H, H_{5b}), 1.01 (d, *J* = 6.5 Hz, 3H, H_7 x 3).

^{13}C NMR (101 MHz, $CDCl_3$) δ 196.0, 152.0, 150.0, 145.3, 138.8, 136.6, 134.4, 123.3, 34.8, 30.2, 27.8, 23.9, 21.6.

HRMS (ESI+): Found $[M+H]^+$ = 202.1226; $C_{13}H_{16}ON$ requires 202.1226, Δ -0.10 ppm

IR (film) ν_{max}/cm^{-1} 2999, 2886, 1646, 1583, 1380, 1268, 1153, 1071, 954, 719.

(4-Methylcyclohex-1-en-1-yl)(quinolin-6-yl)methanone, 2o

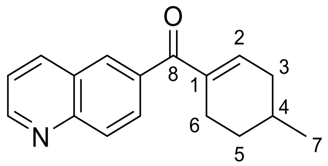

Tetrahydropyran **4o** (81 mg, 0.30 mmol, 1 equiv.), basic alumina (150 mg, 5 equiv., 1.50 mmol), dry PhMe (1.5 mL, 0.2 M) were subjected to **General Procedure 4**. The title compound was afforded after purification by FCC (50% Et_2O/pentane) as a white solid (62 mg, 0.25 mmol, 82% yield).

^{1}H NMR (400 MHz, $CDCl_3$) δ 8.98 (dd, *J* = 4.0, 2.0 Hz, 1H, ArCH), 8.24 (ddd, *J* = 8.0, 2.0, 1.0 Hz, 1H, ArCH), 8.19 – 8.07 (m, 2H, ArCH x 2), 7.96 (dd, *J* = 8.5, 2.0 Hz, 1H, ArCH), 7.47 (dd, *J* = 8.5, 4.5 Hz, 1H, ArCH), 6.66 – 6.56 (m, 1H, H_2), 2.71 – 2.59 (m, 1H, H_{6a}), 2.46 – 2.31 (m, 2H, H_{3a}, H_{6b}), 1.97 – 1.84 (m, 2H, H_{3b}, H_{5a}), 1.85 – 1.70 (m, 1H, H_4), 1.34 (dtd, *J* = 13.0, 11.0, 5.5 Hz, 1H, H_{5b}), 1.04 (d, *J* = 6.5 Hz, 3H, H_7 x 3).

^{13}C NMR (101 MHz, $CDCl_3$) δ 197.4, 151.9, 149.3, 144.5, 138.8, 137.4, 136.9, 129.7, 129.6, 129.5, 127.4, 121.9, 34.8, 30.4, 27.9, 24.2, 21.7.

HRMS (ESI+): Found $[M+H]^+$ = 252.1383; $C_{17}H_{18}ON$ requires 252.1383, Δ -0.12 ppm

IR (film) ν_{max}/cm^{-1} 2999, 2886, 1642, 1380, 1251, 1152, 1089, 954.

m.p.: 80-81 ^{o}C

(3-Chlorophenyl)(4-methylcyclohex-1-en-1-yl)methanone, 2p

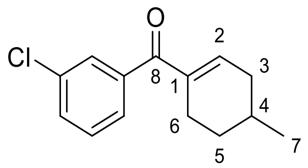

Tetrahydropyran **4p** (76 mg, 0.30 mmol, 1 equiv.), basic alumina (150 mg, 5 equiv., 1.50 mmol), dry PhMe (1.5 mL, 0.2 M) were subjected to **General Procedure 4**. The title

compound was afforded after purification by FCC (2% Et_2O/pentane) as a colourless oil (49 mg, 0.21 mmol, 70% yield).

^1H NMR (400 MHz, $CDCl_3$) δ 7.58 (t, *J* = 2.0 Hz, 1H, ArCH), 7.50 – 7.43 (m, 2H, ArCH x 2), 7.34 (t, *J* = 8.0 Hz, 1H, ArCH), 6.55 (ddd, *J* = 5.0, 2.5, 1.5 Hz, 1H, H_2), 2.64 – 2.51 (m, 1H, H_{6a}), 2.43 – 2.23 (m, 2H, H_{3a}, H_{6b}), 1.96 – 1.82 (m, 2H, H_{3b}, H_{5a}), 1.81 – 1.67 (m, 1H, H_4), 1.36 – 1.22 (m, 1H, H_{5b}), 1.02 (d, *J* = 6.5 Hz, 3H, H_7 x 3).

^{13}C NMR (101 MHz, $CDCl_3$) δ 196.6, 144.6, 140.6, 138.4, 134.3, 131.3, 129.5, 129.2, 127.3, 34.7, 30.3, 27.9, 24.0, 21.6.

HRMS (ESI+): Found $[M+H]^+$ = 235.0885; $C_{14}H_{16}OCl$ requires 235.0884, Δ 0.28 ppm

IR (film) ν_{max}/cm^{-1} 2951, 2926, 2871, 1648, 1566, 1418, 1255, 1078, 736, 709.

(3-Iodophenyl)(4-methylcyclohex-1-en-1-yl)methanone, 2q

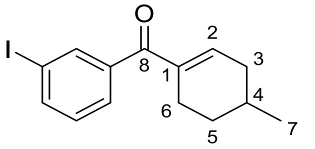

Tetrahydropyran **4q** (103 mg, 0.300 mmol, 1 equiv.), Al(O*t*Bu)$_3$ (74 mg, 1 equiv. 0.30 mmol), dry PhMe (1.5 mL, 0.2 M) were subjected to **General Procedure 4**. The title compound was afforded after purification by FCC (5-10% Et_2O/pentane) as a white solid (80 mg, 0.25 mmol, 82% yield).

^1H NMR (400 MHz, $CDCl_3$) δ 7.92 (t, *J* = 1.5 Hz, 1H, ArCH), 7.81 (ddd, *J* = 8.0, 2.0, 1.0 Hz, 1H, ArCH), 7.55 (ddd, *J* = 7.5, 1.5, 1.0 Hz, 1H, ArCH), 7.15 (t, *J* = 8.0 Hz, 1H, ArCH), 6.58 – 6.51 (m, 1H, H_2), 2.64 – 2.50 (m, 1H, H_{6a}), 2.43 – 2.19 (m, 2H, H_{3a}, H_{6b}), 1.95 – 1.82 (m, 2H, H_{3b}, H_{5a}), 1.81 – 1.65 (m, 1H, H_4), 1.28 (dtd, *J* = 13.0, 10.5, 5.5 Hz, 1H, H_{5b}), 1.02 (d, *J* = 6.5 Hz, 3H, H_7 x 3).

^{13}C NMR (101 MHz, $CDCl_3$) δ 196.4, 144.7, 140.9, 140.1, 138.4, 137.8, 129.9, 128.3, 93.9, 34.7, 30.3, 27.9, 24.0, 21.6.

HRMS (ESI+): Found $[M+H]^+$ = 327.0239; $C_{14}H_{16}OI$ requires 327.0240, Δ -0.33 ppm

IR (film) ν_{max}/cm^{-1} 2950, 2925, 2869, 1647, 1558, 1378, 1256, 1062, 728.

m.p.: 63-64 ^oC

(4-Methylcyclohex-1-en-1-yl)(4-(4,4,5,5-tetramethyl-1,3,2-dioxaborolan-2-yl)phenyl)methanone, 2r

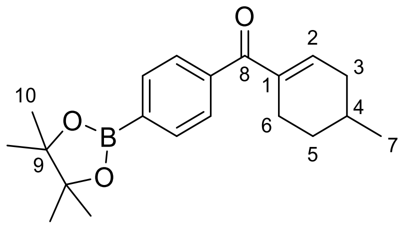

Tetrahydropyran **4r** (103 mg, 0.300 mmol, 1 equiv.), Al(O*t*Bu)$_3$ (74 mg, 1 equiv. 0.30 mmol), dry PhMe (1.5 mL, 0.2 M) were subjected to **General Procedure 4**. The title compound was afforded after purification by FCC (10% Et_2O/pentane) as a white solid (72 mg, 0.22 mmol, 74% yield).

^{1}H NMR (400 MHz, $CDCl_3$) δ 7.87 – 7.81 (m, 2H, ArCH x 2), 7.62 – 7.54 (m, 2H, ArCH x 2), 6.58 – 6.46 (m, 1H, H_2), 2.67 – 2.51 (m, 1H, H_{6a}), 2.42 – 2.23 (m, 2H, H_{3a}, H_{6b}), 1.93 – 1.79 (m, 2H, H_{3b}, H_{5a}), 1.79 – 1.68 (m, 1H, H_4), 1.35 (s, 13H, H_{5b}, H_{10} x 12), 1.01 (d, *J* = 6.5 Hz, 3H, H_7 x 3).

^{13}C NMR (101 MHz, $CDCl_3$) δ 198.4, 144.3, 141.3, 138.7, 134.5, 128.3, 84.2, 34.7, 30.4, 28.0, 25.0, 24.1, 21.7. *N.B. ArC attached to boron was not observed due to quadrupolar relaxation.*

HRMS (ESI+): Found $[M+H]^+$ = 327.2129; $C_{20}H_{28}O_3B$ requires 327.2126, Δ 0.98 ppm

IR (film) ν_{max}/cm^{-1} 2980, 2886, 1647, 1397, 1356, 1260, 1144, 1088, 964, 878

m.p.: 88-89 ^{o}C

(4-Isobutyllcyclohex-1-en-1-yl)(ferrocene)methanone, 2s

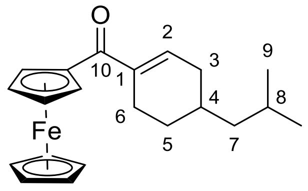

Method 1: Tetrahydropyran **4s** (110 mg, 0.300 mmol, 1 equiv.), basic alumina (150 mg, 5 equiv., 1.50 mmol), dry PhMe (1.5 mL, 0.2 M) were subjected to **General Procedure 4**. The title compound was afforded after purification by FCC (5-10% Et_2O/pentane) as a colourless oil (53 mg, 0.15 mmol, 50% yield).

Method 2: Tetrahydropyran **4s** (110 mg, 0.300 mmol, 1 equiv.), aluminium tert-butoxide (75 mg, 1 equiv., 0.3 mmol), dry PhMe (1.5 mL, 0.2 M) were subjected to **General Procedure 4**. The title compound was afforded after purification by FCC (5-10% Et_2O/pentane) as a colourless oil (62 mg, 0.18 mmol, 59% yield).

^{1}H NMR (400 MHz, $CDCl_3$) δ 6.72 (p, *J* = 2.5 Hz, 1H, H_2), 4.80 (t, *J* = 2.0 Hz, 2H, ArCH x 2), 4.47 (t, *J* = 2.0 Hz, 2H, ArCH x 2), 4.18 (s, 5H, ArCH x 5), 2.60 – 2.47 (m, 1H, H_{6a}), 2.40 – 2.26 (m, 2H, H_{3a}, H_{6b}), 1.95 – 1.54 (m, 4H, H_{3b}, H_4, H_{5a}, H_8), 1.34 – 1.12 (m, 3H, H_{5b}, H_7 x 2), 0.94 – 0.88 (m, 6H, H_9 x 6).

^{13}C NMR (101 MHz, $CDCl_3$) δ 200.0, 139.8, 136.0, 78.9, 71.8, 71.2, 71.2, 70.1, 46.0, 32.8, 30.6, 28.9, 25.1, 24.9, 23.1, 22.9. *N.B.* The peak at δ = 71.8 ppm corresponds to two overlapping signals.

HRMS (ESI+): Found $[M+H]^+$ = 351.1406; $C_{21}H_{27}OFe$ requires 351.1406, Δ 0.81 ppm

IR (film) ν_{max}/cm^{-1} 2953, 2868, 1625, 1443 1276, 821.

Cyclohexyl(4-methylcyclohex-1-en-1-yl)methanone, 2t

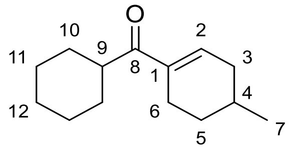

Method 1: Tetrahydropyran **4t** (67 mg, 0.30 mmol, 1 equiv.), basic alumina (150 mg, 5 equiv., 1.50 mmol), dry PhMe (1.5 mL, 0.2 M) were subjected to **General Procedure 4**. The title compound was afforded after purification by FCC (2% Et_2O/pentane) as a colourless oil (58 mg, 0.28 mmol, 94% yield).

Method 2: A 10 mL flame dried RBF fitted with a reflux condenser was charged with Phosphonate **S39** (184 mg, 0.700 mmol, 1.4 equiv.) and the flask was evacuated and backfilled with N_2 three times. Dry toluene (2.5 mL) was added, and 60% wt. NaH (24 mg, 0.60 mmol, 1.2 equiv.) was added in one portion [care: vigorous release of H_2]. The resulting suspension was stirred at RT for 20 min, by which time a homogeneous solution had formed. Lactol **2d** (58 mg, 0.50 mmol, 1.0 equiv.) was added and the reaction was heated at 110 °C for 15 h. The reaction was then cooled to RT, and water (3 mL) was added. The resulting mixture was extracted with a 1:1 mixture of pentane/Et_2O (5 x 3 mL) and the combined organic extracts were filtered through a short pad of silica gel, washing with 3 further 3 mL portions of 1:1 pentane/Et_2O. The filtrate was concentrated under reduced pressure and the residue was dissolved in dry PhMe (2.5 mL) and transferred to a flame dried 2–5 mL Biotage® microwave vial and aluminium *tert*-butoxide (185 mg, 0.750 mmol 1.5 equiv.) was added. The headspace was briefly purged with N_2 and the reaction vessel was sealed with a microwave vial cap (containing a Reseal™ septum) and heated at 110 °C for 16 h. The reaction was then cooled

to RT, diluted with Et_2O (50 mL), filtered through a plug of silica gel and concentrated *in vacuo*. The title compound was afforded after purification by FCC (3% Et_2O/pentane) as a colourless oil (91 mg, 0.44 mmol, 88% yield over two steps).

^{1}H NMR (400 MHz, $CDCl_3$) δ 6.85 (dt, *J* = 5.0, 2.0 Hz, 1H, H_2), 2.96 (tt, *J* = 11.5, 3.5 Hz, 1H, H_9), 2.49 – 2.26 (m, 2H, H_{3a}, H_{6a}), 2.19 – 1.99 (m, 1H, H_{6b}), 1.94 – 1.55 (m, 8H, H_{3b}, H_4, H_{5a}, H_{10a} x 2, H_{11a} x 2, H_{12a}), 1.48 – 1.08 (m, 6H, $H_{5b,}$ H_{10b} x 2, H_{11b} x 2, H_{12b}), 0.98 (d, *J* = 6.5 Hz, 3H, H_7 x 3).

^{13}C NMR (101 MHz, $CDCl_3$) δ 205.1, 138.6, 138.0, 44.4, 34.7, 30.5, 29.9, 27.9, 26.1, 26.1, 23.6, 21.6.

HRMS (ESI+): Found $[M+H]^+$ = 207.1744; $C_{14}H_{23}O$ requires 207.1743, Δ 0.25 ppm

IR (film) ν_{max}/cm^{-1} 2998, 2929, 1709, 1663, 1450, 1382, 1248, 1151, 954.

1-(Spiro[4.5]dec-7-en-8-yl)ethan-1-one, 2u

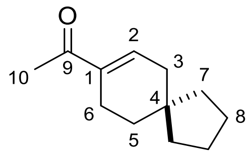

Tetrahydropyran **4u** (58 mg, 0.30 mmol, 1 equiv.), basic alumina (150 mg, 5 equiv. 1.50 mmol), dry PhMe (1.5 mL, 0.2 M) were subjected to **General Procedure 4**. The title compound was afforded after purification by FCC (10% Et_2O/pentane) as a colourless oil (49 mg, 0.28 mmol, 92% yield).

^{1}H NMR (400 MHz, $CDCl_3$) δ 6.84 (tt, *J* = 4.0, 1.5 Hz, 1H, H_2), 2.32 – 2.23 (m, 5H, H_6 x 2, H_{10} x 3), 2.13 (dt, *J* = 4.0, 2.5 Hz, 2H, H_3 x 2), 1.70 – 1.59 (m, 4H, H_7 x 4), 1.50 (t, *J* = 6.5 Hz, 2H, H_5 x 2), 1.46 – 1.31 (m, 4H, H_8 x 4).

^{13}C NMR (101 MHz, $CDCl_3$) δ 199.4, 140.7, 139.4, 40.5, 39.0, 38.3, 33.3, 25.4, 24.5, 21.7.

HRMS (ESI+): Found $[M+H]^+$ = 179.1432; $C_{12}H_{19}O$ requires 179.1430, Δ 0.90 ppm

IR (film) ν_{max}/cm^{-1} 2951, 2856, 1665, 1639, 1385, 1248, 1074, 953, 807.

3,7-Dimethyl-1-(4-methylcyclohex-1-en-1-yl)oct-6-en-1-one, 2v

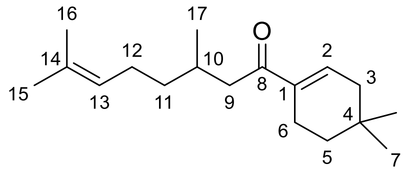

Tetrahydropyran **4v** (84 mg, 0.30 mmol, 1 equiv.), basic alumina (150 mg, 5 equiv., 1.50 mmol), dry PhMe (1.5 mL, 0.2 M) were subjected to **General Procedure 4**. The title compound was afforded after purification by FCC (2-5% Et_2O/pentane) as a colourless oil (75 mg, 0.29 mmol, 95% yield).

^{1}H NMR (400 MHz, $CDCl_3$) δ 6.79 (tt, *J* = 4.0, 1.5 Hz, 1H, H_2), 5.08 (t hept, *J* = 7.0, 1.7 Hz, 1H, H_{13}), 2.61 (dd, *J* = 15.0, 5.5 Hz, 1H, H_{9a}), 2.42 (dd, *J* = 15.0, 8.0 Hz, 1H, H_{9b}), 2.31 – 2.21 (m, 2H, H_6 x 2), 2.10 – 1.90 (m, 5H, H_3 x 2, $H_{10,}$ H_{12} x 2), 1.68 (s, 3H, H_{15} x 3 or H_{16} x 3), 1.59 (s, 3H, H_{15} x 3 or H_{16} x 3), 1.44 – 1.13 (m, 4H, H_5 x 2, H_{11} x 2), 0.91 (s, 6H, H_7 x 6), 0.89 (d, *J* = 6.5 Hz, 3H, H_{17} x 3).

^{13}C NMR (101 MHz, $CDCl_3$) δ 201.7, 138.9, 138.7, 131.5, 124.7, 44.6, 40.2, 37.4, 35.1, 30.1, 28.6, 28.3, 28.2, 25.9, 25.7, 21.2, 20.1, 17.8.

HRMS (ESI+): Found $[M+H]^+$ = 263.2370; $C_{18}H_{31}O$ requires 263.2369, Δ 0.33 ppm

IR (film) ν_{max}/cm^{-1} 2980, 2867, 1667, 1639, 1460, 1381, 1363, 1170, 1085, 733.

***Tert*-butyl 4-(4-methylcyclohex-1-ene-1-carbonyl)piperidine-1-carboxylate, 2w**

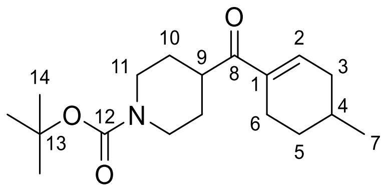

Tetrahydropyran **4w** (98 mg, 0.30 mmol, 1 equiv.), $Al(O^tBu)_3$ (74 mg, 1 equiv. 0.30 mmol), dry PhMe (1.5 mL, 0.2 M) were subjected to **General Procedure 4**, and heated for 48 hours. The title compound was afforded after purification by FCC (10-20% Et_2O/pentane) as a colourless oil (69 mg, 0.22 mmol, 75% yield).

^{1}H NMR (400 MHz, $CDCl_3$) δ 6.95 – 6.81 (m, 1H, H_2), 4.22 – 4.00 (m, 2H, H_{11a} x 2), 3.11 (tt, *J* = 10.5, 4.5 Hz, 1H, H_9), 2.98 – 2.60 (m, 2H, H_{11b} x 2), 2.49 – 2.26 (m, 2H, H_{3a}, H_{6a}), 2.21 – 2.03 (m, 1H, H_{6b}), 1.93 – 1.73 (m, 2H, H_{3b}, H_{5a}), 1.72 – 1.52 (m, 5H, H_4, H_{10} x 4), 1.45 (s, 9H, H_{14} x 9), 1.26 – 1.11 (m, 1H, H_{5b}), 0.98 (d, *J* = 6.5 Hz, 3H, H_7 x 3).

^{13}C NMR (101 MHz, $CDCl_3$) δ 203.0, 154.7, 139.1, 137.8, 79.5, 43.4, 42.1, 34.5, 30.2, 28.8, 28.5, 27.7, 23.4, 21.5.

HRMS (ESI+): Found $[M+Na]^+$ = 330.2039; $C_{18}H_{29}O_3NNa$ requires 330.2040, Δ -0.23 ppm

IR (film) ν_{max}/cm^{-1} 2951, 2927, 1682, 1659, 1635, 1423, 1160, 906.

(4-(Dimethyl(phenyl)silyl)cyclohex-1-en-1-yl)(phenyl)methanone 2x

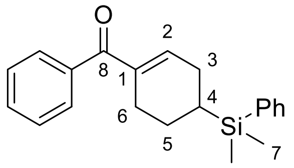

tert-Butanol (496 mg, 6.60 mmol, 2.96 eq.), trimethylaluminium (2M in toluene, 1.1 mL, 2.2 mmol, 1.0 eq.), tetrahydropyran **4x** (755 mg, 2.23 mmol, 1.00 eq.) and dry toluene (10 mL) were subjected to **General Procedure 4.** Purification by FCC (0-2% Et_2O/pentane) afforded the product as a white solid (502 mg, 1.57 mmol, 70% yield).

^1H NMR (400 MHz, $CDCl_3$) δ 7.63 – 7.56 (m, 2H, ArCH x 2), 7.55 – 7.44 (m, 3H, ArCH x 3), 7.43 – 7.31 (m, 5H, ArCH x 5), 6.62 – 6.55 (m, 1H, H_2), 2.66 – 2.55 (m, 1H, H_{6a}), 2.33 – 1.94 (m, 4H, H_{6b}, H_{3a}, H_{3b}, H_{5a}), 1.46 – 1.30 (m, 1H, H_{5b}), 1.08 (dddd, *J* = 12.5, 11.5, 5.0, 2.5 Hz, 1H, H_4), 0.32 (s, 3H, H_{7a} x 3), 0.31 (s, 3H, H_{7b} x 3).

^{13}C NMR (101 MHz, $CDCl_3$) δ 198.3, 144.8, 138.9, 138.8, 137.7, 134.1, 131.4, 129.3, 129.2, 128.1, 127.9, 27.4, 25.0, 23.2, 20.4, -5.0, -5.1.

m.p. = 42-43 °C

HRMS (ESI+): Found $[M+H]^+$ = 321.1670; $C_{21}H_{25}OSi$ requires 321.1669, Δ 0.23 ppm

IR (film) ν_{max}/cm^{-1}: 2918, 1645, 1262, 1113, 809, 701

Phenyl(4-(phenylthio)cyclohex-1-en-1-yl)methanone 2y

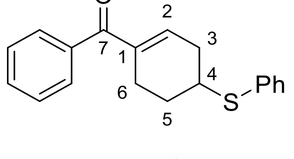

tert-Butanol (186 mg, 2.51 mmol, 3.00 eq.), trimethylaluminium (2M in toluene, 0.4 mL, 0.8 mmol, 1.0 eq.), tetrahydropyran **4y** (261 mg, 0.835 mmol, 1.00 eq.) and dry toluene (4 mL) were subjected to **General Procedure 4**. Purification by FCC (5% Et_2O/pentane) afforded the product as a yellow solid (171 mg, 0.580 mmol, 69% yield).

^{1}H NMR (400 MHz, $CDCl_3$) δ 7.62 – 7.53 (m, 2H, ArCH x 2), 7.49 – 7.31 (m, 5H, ArCH x 5), 7.29 – 7.16 (m, 3H, ArCH x 3), 6.48 – 6.37 (m, 1H, H_2), 3.38 (dddd, J = 10.0, 8.0, 5.5, 3.0 Hz, 1H, H_4), 2.72 – 2.53 (m, 2H, H_{6a}, H_{3a}), 2.46 – 2.20 (m, 2H, H_{6b}, H_{3b}), 2.16 – 2.04 (m, 1H, H_{5a}), 1.76 – 1.62 (m, 1H, H_{5b}).

^{13}C NMR (101 MHz, $CDCl_3$) δ 197.5, 140.9, 138.3, 138.2, 134.2, 132.4, 131.6, 129.2, 129.0, 128.2, 127.3, 41.9, 32.7, 28.3, 23.5.

m.p. = 68-69 °C

HRMS (ESI+): Found $[M+H]^+$ 295.1151; $C_{19}H_{19}OS$ requires 295.1151, Δ -0.21 ppm

IR (film) ν_{max}/cm^{-1}: 1643, 1263, 956, 745, 702, 657

Phenyl(4-(phenylsulfinyl)cyclohex-1-en-1-yl)methanone S45

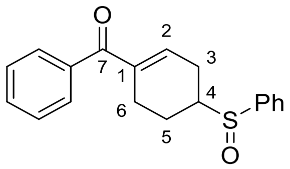

A 5 mL RBF was charged with cyclohexene **2y** (59.9 mg, 0.203 mmol, 1.00 equiv.) in $CHCl_3$ (1.2 mL, 0.2M) at 0 °C. *m*-CPBA (70% wt., 49 mg, 0.20 mmol, 1.0 equiv.) was added in one portion and the resulting mixture was stirred at 0 °C for 20 min, after which the mixture was filtered. The filtrate was washed with 3M NaOH (10 mL) and brine (10 mL), dried over Na_2SO_4, and concentrated *in vacuo*. Purification by FCC (20-30% EtOAc/pentane) afforded the product as a colourless oil (45.0 mg, 0.145 mmol, 71% yield, 62:38 d.r., diastereomers were separable by column chromatography).

Data for major **S45**:

^{1}H NMR (600 MHz, $CDCl_3$) δ 7.64 – 7.56 (m, 4H, ArCH x 4), 7.56 – 7.47 (m, 4H, ArCH x 4), 7.42 – 7.36 (m, 2H, ArCH x 2), 6.54 – 6.48 (m, 1H, H_2), 2.89 – 2.81 (m, 1H, H_4), 2.81 – 2.68 (m, 2H, H_{5a}, H_{3a}), 2.49 – 2.40 (m, 1H, H_{5b}), 2.29 – 2.21 (m, 1H, H_{6a}), 2.13 – 2.04 (m, 1H, H_{3b}), 1.88 – 1.78 (m, 1H, H_{6b}).

^{13}C NMR (151 MHz, $CDCl_3$) δ 197.1, 141.8, 140.6, 138.1, 131.8, 131.3, 129.3, 129.2, 128.3, 124.7, 58.6, 23.8, 23.2, 22.5. The peaks at δ = 138.1 ppm correspond to overlapping signals.

Data for minor **S45**:

^1H NMR (400 MHz, $CDCl_3$) δ 7.70 – 7.45 (m, 8H, ArCH x 8), 7.44 – 7.35 (m, 2H, ArCH x 2), 6.48 – 6.44 (m, 1H, H_2), 2.97 – 2.83 (m, 1H, H_4), 2.86 – 2.72 (m, 1H, H_{6a}), 2.49 – 2.28 (m, 3H, H_{6b}, H_{3a}, H_{3b}), 2.26 – 2.09 (m, 1H, H_{5a}), 1.88 – 1.71 (m, 1H, H_{5b}).

^{13}C NMR (101 MHz, $CDCl_3$) δ 197.1, 141.5, 139.4, 138.8, 138.0, 131.9, 131.6, 129.4, 129.3, 128.3, 125.2, 59.0, 25.9, 23.6, 20.6.

HRMS (ESI+): Found $[M+H]^+$ = 311.1098; $C_{19}H_{19}O_2S$ requires 311.1100, Δ -0.62 ppm.

IR (film) ν_{max}/cm^{-1}: 1639, 1444, 1266, 1043, 750, 703.

Phenyl(4-(phenylsulfonyl)cyclohex-1-en-1-yl)methanone S46

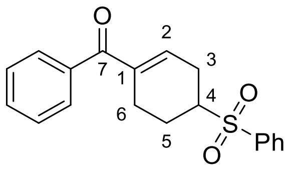

A 10 mL RBF was charged with cyclohexene **2y** (57.6 mg, 0.196 mmol, 1.00 equiv.) in a 4:1 mixture of EtOH/THF (1.25 mL, 0.2M) at 0 °C. Ammonium molybdate (24.7 mg, 0.0200 mmol, 0.100 equiv.) dissolved in H_2O_2 (30% wt. 0.25 mL, 2.0 mmol, 10 equiv.) was added dropwise, after which the mixture was stirred at RT for 16 h. The reaction was quenched with saturated aqueous $NaHCO_3$ (1 mL), and then extracted with EtOAc (3 x 10 mL). The combined organic layers were washed with brine (10 mL), dried over Na_2SO_4, and concentrated *in vacuo*. Purification by FCC (20% EtOAc/Pentane) afforded the product as a colourless oil (55.2 mg, 0.169 mmol, 86% yield).

^1H NMR (400 MHz, $CDCl_3$) δ 8.00 – 7.82 (m, 2H, ArCH x 2), 7.75 – 7.64 (m, 1H, ArCH), 7.64 – 7.54 (m, 4H, ArCH x 4), 7.54 – 7.46 (m, 1H, ArCH), 7.46 – 7.33 (m, 2H, ArCH x 2), 6.56 – 6.41 (m, 1H, H_2), 3.33 – 3.16 (m, 1H, H_4), 2.88 – 2.70 (m, 1H, H_{6a}), 2.65 – 2.52 (m, 2H, H_{3a}, H_{3b}), 2.46 – 2.24 (m, 2H, H_{6b}, H_{5a}), 1.81 – 1.66 (m, 1H, H_{5b}).

^{13}C NMR (101 MHz, $CDCl_3$) δ 196.8, 138.7, 138.2, 137.8, 137.0, 134.1, 131.9, 129.4, 129.2, 129.0, 128.3, 59.2, 25.4, 23.5, 21.7.

HRMS (ESI+): Found $[M+H]^+$ = 327.1049; $C_{19}H_{19}O_3S$ requires 327.1049, Δ -0.16 ppm.

IR (film) ν_{max}/cm^{-1}: 1642, 1305, 1264, 1147, 1086, 705.

Phenyl(4-((tetrahydro-2*H*-pyran-2-yl)oxy)cyclohex-1-en-1-yl)methanone 2z

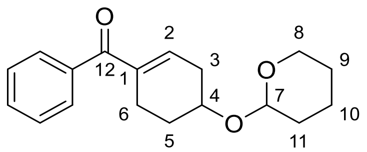

tert-Butanol (28 mg, 0.38 mmol, 3.0 eq.), trimethylaluminium (2M in toluene, 0.06 mL, 0.1 mmol, 1 eq.), tetrahydropyran **4z** (38.6 mg, 0.127 mmol, 1.00 eq.) and dry toluene (0.5 mL) were subjected to **General Procedure 4**. Purification by FCC (5% EtOAc/pentane) afforded the product as a yellow oil (16.8 mg, 0.0587 mmol, 46% yield, 55:45 d.r. mixture of anomers by ^{1}H NMR).

^{1}H NMR (400 MHz, $CDCl_3$) δ 7.65 – 7.59 (m, 4H, major ArCH x 2, minor ArCH x 2), 7.54 – 7.45 (m, 2H, major ArCH, minor ArCH), 7.45 – 7.37 (m, 4H, major ArCH x 2, minor ArCH x 2), 6.52 – 6.41 (m, 2H, major H_2, minor H_2), 4.80 – 4.73 (m, 2H, major H_7, minor H_7), 4.07 – 3.86 (m, 4H, major H_4, minor H_4, major H_{8a}, minor H_{8a}), 3.58 – 3.46 (m, 2H, major H_{8b}, minor H_{8b}), 2.76 – 2.52 (m, 4H, major H_{6a}, minor H_{6a}, major H_{3a}, minor H_{3a}), 2.51 – 2.32 (m, 3H, major H_{6b}, minor H_{6b}, minor H_{3b}), 2.31 – 2.20 (m, 1H, major H_{3b}), 2.13 – 1.91 (m, 2H, major H_{5a}, minor H_{5a}), 1.90 – 1.68 (m, 6H, major H_{10a}, minor H_{10a}, major H_{11a}, minor H_{11a}, major H_{5b}, minor H_{5b}), 1.63 – 1.48 (m, 8H, major H_{9a}, minor H_{9a}, major H_{9b}, minor H_{9b}, major H_{10b}, minor H_{10b}, major H_{11b}, minor H_{11b}).

^{13}C NMR (101 MHz, $CDCl_3$) δ 197.7, 141.5, 140.5, 138.7, 138.6, 138.6, 138.2, 131.6, 131.5, 129.3, 128.2, 97.3, 97.3, 70.4, 70.3, 63.1, 62.9, 33.7, 32.0, 31.3, 31.3, 29.0, 26.9, 25.6, 25.6, 22.9, 22.4, 20.1, 19.9. *N.B.* The peaks at δ = 197.7, 129.3, 128.2 ppm correspond to overlapping signals.

HRMS (ESI+): Found $[M+Na]^+$ = 309.1461; $C_{18}H_{22}O_3Na$ requires 309.1461, Δ -0.09 ppm

IR (film) ν_{max}/cm^{-1}: 2941, 1646, 1264, 1076, 1032, 705

Phenyl(4-(4,4,5,5-tetramethyl-1,3,2-dioxaborolan-2-yl)cyclohex-1-en-1-yl)methanone 2aa

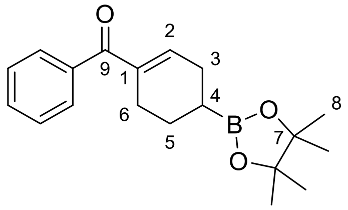

tert-Butanol (382 mg, 5.16 mmol, 3.00 eq.), trimethylaluminium (2M in toluene, 0.86 mL, 1.7 mmol, 1.0 eq.), tetrahydropyran **4aa** (567 mg, 1.72 mmol, 1.00 eq.) and dry toluene (5 mL) were subjected to **General Procedure 4**. Purification by FCC (3-5% Et_2O/pentane) afforded the product as a white solid (373 mg, 1.19 mmol, 69% yield).

^1H NMR (600 MHz, $CDCl_3$) δ 7.62 – 7.58 (m, 2H, ArCH x 2), 7.49 – 7.43 (m, 1H, ArCH), 7.41 – 7.35 (m, 2H, ArCH x 2), 6.61 – 6.56 (m, 1H, H_2), 2.54 – 2.46 (m, 1H, H_{6a}), 2.37 – 2.26 (m, 3H, H_{6b}, H_{3a}, H_{3b}), 1.97 – 1.89 (m, 1H, H_{5a}), 1.62 (dddd, J = 13.5, 10.5, 9.0, 5.5 Hz, 1H, H_{5b}), 1.24 – 1.21 (m, 12H, H_4, H_8 x 12).

^{13}C NMR (151 MHz, $CDCl_3$) δ 198.3, 144.6, 138.9, 138.5, 131.3, 129.2, 128.0, 83.3, 27.5, 24.9, 24.8, 24.2, 23.6, 16.9 (broad).

m.p. = 78-80 °C

HRMS (ESI+): Found $[M+H]^+$ = 313.1972; $C_{19}H_{26}BO_3$ requires 313.1970, Δ 0.91 ppm

IR (film) ν_{max}/cm^{-1}: 2978, 1646, 1379, 1321, 1143, 704

(4-Hydroxycyclohex-1-en-1-yl)(phenyl)methanone 2ab

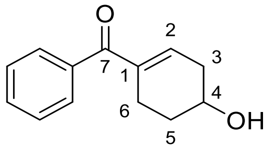

Method 1: A 25 mL RBF was charged with cyclohexene **2aa** (88.4 mg, 0.283 mmol, 1.00 eq.) in THF (2.5 mL) and water (2.5 mL). Sodium perborate tetrahydrate (218 mg, 1.42 mmol, 5.00 eq.) was added and the reaction was stirred at RT for 30 min. Water was added, and then the mixture was extracted with EtOAc (3 x 15 mL). The organic phases were washed with brine, dried with $MgSO_4$ and the solvent was removed *in vacuo*. Purification by FCC (50% Et2O/pentane) afforded the product as a colourless oil (46.4 mg, 0.229 mmol, 81% yield).

Method 2: A 5 mL microwave vial was charged with cyclohexene **2x** (64 mg, 0.20 mmol, 1.0 eq.) in dry CH_2Cl_2 (1 mL) under argon. BF_3·2AcOH (75.2 mg, 0.400 mmol, 2.00 eq.) was added and the resulting solution was stirred at RT for 7 h. The reaction was quenched by the addition of saturated aqueous $NaHCO_3$ (3 mL), and extracted with CH_2Cl_2 (3 x 10 mL). The organic layers were washed with brine, dried over Na_2SO4, and concentrated *in vacuo* to give (4-(fluorodimethylsilyl)cyclohex-1-en-1-yl)(phenyl)methanone, which was used without further purification.

A 5 mL microwave vial was charged with crude (4-(fluorodimethylsilyl)cyclohex-1- en-1-yl)(phenyl)methanone (52.5 mg, 0.200 mmol, 1.00 eq.) in Et_2O (0.6 mL) at 0 °C. *m*-CPBA (70% wt., 200 mg, 0.800 mmol, 4.00 eq.) was added, followed by Et_3N (24.2 mg, 0.240 mmol, 1.20 eq.), and the mixture was stirred at RT for 2 h. The reaction was quenched with aqueous $Na_2S_2O_3$ (2 mL), diluted with Et_2O (5 mL). The organic layers were washed with $Na_2S_2O_3$ (5 mL) and 3M HCl (5 mL), then concentrated *in vacuo*. The crude product was then redissolved in MeOH (5 mL), to which was added a drop of concentrated HCl, and left for 15 min, before being concentrated *in vacuo*. The product was again redissolved in Et_2O (5 mL), and washed with saturated aqueous $NaHCO_3$ (5 mL), and concentrated *in vacuo*. Purification by FCC (50% Et_2O/pentane) afforded the product as a colourless oil (14.0 mg, 0.0692 mmol, 35% yield over 2 steps).

Method 3: A 5 mL RBF was charged with cyclohexene **2z** (17.0 mg, 0.059 mmol, 1.00 eq.) in MeOH (0.12 mL) at RT. $TsOH.H_2O$ (1.1 mg, 0.0058 mmol, 0.10 eq.) was added dropwise, and the mixture was stirred for 1 h. The reaction was quenched by addition of water (1 mL), and the mixture was extracted with Et_2O (3 x 10 mL). The combined organic phases were washed with brine (10 mL), dried over Na_2SO_4 and concentrated *in vacuo*. Purification by FCC (25% EtOAc/pentane) afforded the product as a colourless oil (7.8 mg, 0.039 mmol, 65% yield).

^1H NMR (400 MHz, $CDCl_3$) δ 7.67 – 7.56 (m, 2H, ArCH x 2), 7.54 – 7.45 (m, 1H, ArCH), 7.44 – 7.36 (m, 2H, ArCH x 2), 6.49 – 6.41 (m, 1H, H_2), 4.15 – 4.00 (m, 1H, H_4), 2.70 – 2.53 (m, 2H, H_{6a}, H_{3a}), 2.52 – 2.40 (m, 1H, H_{6b}), 2.33 – 2.20 (m, 1H, H_{3b}), 2.19 – 2.06 (m, 1H, OH), 2.02 – 1.91 (m, 1H, H_{5a}), 1.83 – 1.69 (m, 1H, H_{5b}).

^{13}C NMR (101 MHz, $CDCl_3$) δ 197.7, 140.7, 138.4, 138.2, 131.6, 129.2, 128.2, 65.9, 34.9, 30.5, 22.2.

HRMS (ESI+): Found $[M+H]^+$ = 203.1066; $C_{13}H_{15}O_2$ requires 203.1067, Δ -0.29 ppm.

IR (film) ν_{max}/cm^{-1}: 3421 (broad), 2926, 1633, 1264, 1072, 703.

(*S*)-Phenyl(1,2,3,6-tetrahydro-[1,1'-biphenyl]-4-yl)methanone, 2ac

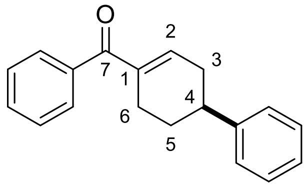

Tetrahydropyran **4ac** (84 mg, 0.30 mmol, 1 equiv.), aluminium tert-butoxide (75 mg, 1 equiv., 0.30 mmol), dry PhMe (1.5 mL, 0.2 M) were subjected to **General Procedure 4**. The title

compound was afforded after purification by FCC (5-10% Et_2O/pentane) as a colourless oil (75 mg, 0.29 mmol, 95% yield, 99:1 e.r.).

^{1}H NMR (400 MHz, $CDCl_3$) δ 7.63 – 7.56 (m, 2H, ArCH x 2), 7.43 (t, *J* = 7.5 Hz, 1H, ArCH), 7.35 (t, *J* = 7.5 Hz, 2H, ArCH x 2), 7.25 (t, *J* = 7.5 Hz, 2H, ArCH x 2), 7.20 – 7.12 (m, 3H, ArCH x 3), 6.58 (dt, *J* = 5.0, 2.5 Hz, 1H, H_2), 2.88 – 2.70 (m, 1H, H_4), 2.70 – 2.56 (m, 1H, H_{6a}), 2.56 – 2.23 (m, 3H, H_3 x 2, H_{6b}), 2.05 (ddq, *J* = 13.0, 5.0, 2.5 Hz, 1H, H_{5a}), 1.76 (qd, *J* = 12.0, 5.0 Hz, 1H, H_{5b}).

^{13}C NMR (101 MHz, $CDCl_3$) δ 198.0, 146.1, 143.1, 138.7, 138.7, 131.5, 129.3, 128.7, 128.2, 126.9, 126.5, 39.5, 34.3, 29.5, 24.9.

HRMS (ESI+): Found $[M+H]^+$ = 263.3597; $C_{19}H_{19}O$ requires 263.3595, Δ -0.56 ppm

IR (film) ν_{max}/cm^{-1} : 2927, 2868, 1672, 1640, 1579, 1479

α_{25}^{D}= −70.8 (c = 1.0, $CHCl_3$).

HPLC: Enantiomeric excess was determined by HPLC with a Chiralpak® IH column (99:1 hexane:IPA, 1.0 mL min^{-1}, 254 nm, room temperature); t_r (minor) = 8.1 min, t_r (major) = 9.1 min.

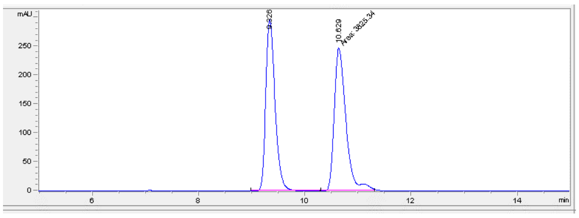

#	Time	Type	Area	Height	Width	Area%	Symmetry
1	9.326	BB	3605	297.6	0.1873	48.517	0.69
2	10.629	MF	3825.3	248.5	0.2565	51.483	0.623

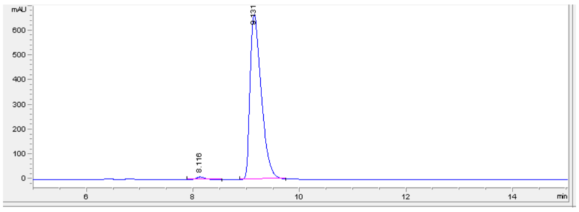

#	Time	Type	Area	Height	Width	Area%	Symmetry
1	8.116	BB	113.9	10.3	0.1693	1.160	0.734
2	9.131	BB	9704.6	667.1	0.2217	98.840	0.532

(*S*)-(4'-Fluoro-1,2,3,6-tetrahydro-[1,1'-biphenyl]-4-yl)(phenyl)methanone, 2ad

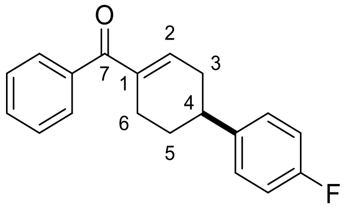

Tetrahydropyran **4ad** (90 mg, 0.30 mmol, 1 equiv.), aluminium tert-butoxide (75 mg, 1 equiv., 0.30 mmol), dry PhMe (1.5 mL, 0.2 M) were subjected to **General Procedure 4**. The title compound was afforded after purification by FCC (5% Et_2O/pentane) as a colourless oil (65 mg, 0.23 mmol, 77% yield, 98:2 e.r.).

^{1}H NMR (400 MHz, $CDCl_3$) δ 7.70 – 7.65 (m, 2H, ArCH x 2), 7.55 – 7.49 (m, 1H, ArCH), 7.47 – 7.41 (m, 2H, ArCH x 2), 7.24 – 7.17 (m, 2H, ArCH x 2), 7.05 – 6.98 (m, 2H, ArCH x 2), 6.66 –

6.63 (m, 1H, H_2), 2.92 – 2.83 (m, 1H, H_4), 2.77 – 2.69 (m, 1H, H_{6a}), 2.62 – 2.43 (m, 2H, H_{3a} and H_{6b}), 2.41 – 2.30 (m, 1H, H_{3b}), 2.16 – 2.05 (m, 1H, H_{5a}), 1.80 (dddd, *J* = 13.0, 12.0, 11.0, 5.5 Hz, 1H, H_{5b}).

^{13}C NMR (101 MHz, $CDCl_3$) δ 197.9, 161.6 (d, *J* = 244.0 Hz), 142.8, 141.7 (d, *J* = 3.0 Hz), 138.7, 138.7, 131.6, 129.3, 128.3 (d, *J* = 7.5 Hz), 128.2, 115.4 (d, *J* = 21.0 Hz), 38.8, 34.4, 29.6, 24.8.

^{19}F NMR (377 MHz, $CDCl_3$) δ -116.9.

HRMS (ESI+): Found $[M+H]^+$ = 281.1335; $C_{19}H_{18}OF$ requires 281.1336, Δ -0.48 ppm

IR (film) ν_{max}/cm^{-1} : 2927, 1642, 1509, 1261, 1221, 831.

α_{25}^{D}= −79.4 (c = 1.0, $CHCl_3$).

HPLC: Enantiomeric excess was determined by HPLC with a Chiralpak® IH column (99:1 hexane:IPA, 1.0 mL min^{-1}, 254 nm, room temperature), t_r (minor) = 14.4 min, t_r (major) = 16.2 min.

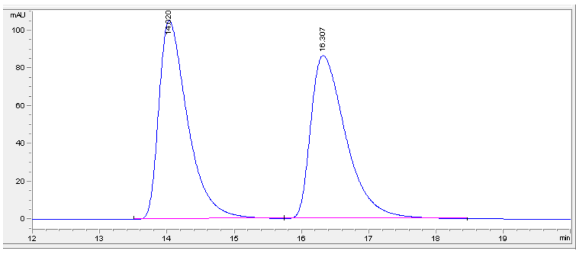

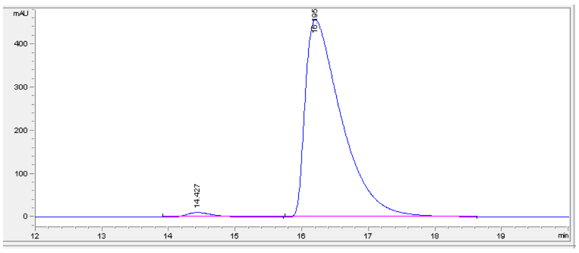

#	Time	Type	Area	Height	Width	Area%	Symmetry
1	14.02	BB	3128.2	104.8	0.4501	50.040	0.519
2	16.307	BB	3123.1	86.2	0.5589	49.960	0.501

#	Time	Type	Area	Height	Width	Area%	Symmetry
1	14.427	BB	309	10.9	0.4265	1.756	0.625
2	16.195	BB	17286.5	457.1	0.5652	98.244	0.358

(*S*)-(2'-Methyl-1,2,3,6-tetrahydro-[1,1'-biphenyl]-4-yl)(phenyl)methanone, 2ae

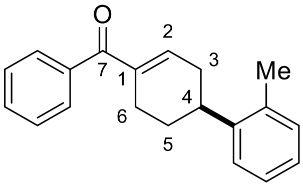

Tetrahydropyran **4ae** (88 mg, 0.30 mmol, 1 equiv.), aluminium tert-butoxide (75 mg, 1 equiv., 0.30 mmol), dry PhMe (1.5 mL, 0.2 M) were subjected to **General Procedure 4**. The title compound was afforded after purification by FCC (5% Et_2O/pentane) as a pale yellow solid (62 mg, 0.22 mmol, 75% yield, 96:4 e.r.).

^{1}H NMR (400 MHz, $CDCl_3$) δ 7.73 – 7.67 (m, 2H, ArCH x 2), 7.56 – 7.50 (m, 1H, ArCH), 7.48 – 7.41 (m, 2H, ArCH x 2), 7.25 – 7.11 (m, 4H, ArCH x 4), 6.72 – 6.67 (m, 1H, H_2), 3.16 – 3.06 (m, 1H, H_4), 2.81 – 2.72 (m, 1H, H_{6a}), 2.60 – 2.46 (m, 2H, H_{6b} and H_{3a}), 2.38 (s, 4H, H_{3b} and $ArCH_3$ x 3), 2.10 – 2.02 (m, 1H, H_{5a}), 1.86 (dtd, *J* = 13.0, 11.5, 5.0 Hz, 1H, H_{5b}).

^{13}C NMR (101 MHz, $CDCl_3$) δ 198.0, 144.1, 143.5, 138.7, 138.7, 135.5, 131.5, 130.6, 129.3, 128.2, 126.5, 126.2, 125.3, 35.1, 33.4, 28.9, 25.2, 19.6.

HRMS (ESI+): Found $[M+H]^+$ = 277.1585; $C_{20}H_{21}O$ requires 277.1587, Δ –0.66 ppm

IR (film) ν_{max}/cm^{-1} : 2928, 1643, 1262, 956, 756, 727, 703, 666.

m.p.: 83-85 °C

$\alpha_{25}{}^{D}$= −84.0 (c = 1.0, $CHCl_3$).

HPLC: Enantiomeric excess was determined by HPLC with a Chiralpak® IH column (99:1 hexane:IPA, 1.0 mL min^{-1}, 254 nm, room temperature), t_r (minor) = 9.6 min, t_r (major) = 10.5 min.

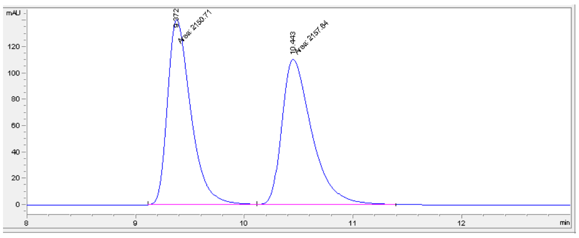

#	Time	Type	Area	Height	Width	Area%	Symmetry
1	9.372	FM	2150.7	141.6	0.2532	49.920	0.629
2	10.443	MF	2157.6	111.2	0.3234	50.080	0.574

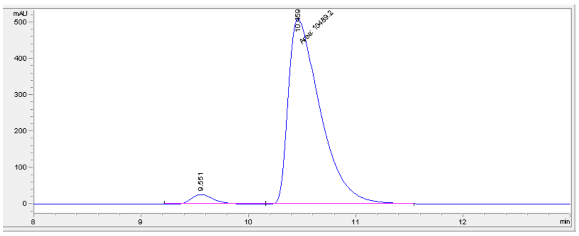

#	Time	Type	Area	Height	Width	Area%	Symmetry
1	9.551	BV	411.7	27.1	0.2315	3.777	0.681
2	10.459	MF	10489.2	508.8	0.3436	96.223	0.438

(*S*)-(4'-Methoxy-1,2,3,6-tetrahydro-[1,1'-biphenyl]-4-yl)(phenyl)methanone, 2af

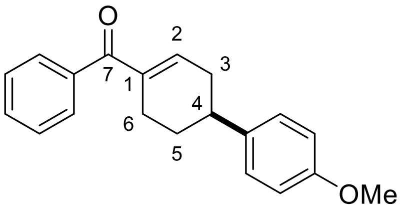

Tetrahydropyran **4af** (93 mg, 0.30 mmol, 1 equiv.), aluminium tert-butoxide (75 mg, 1 equiv., 0.30 mmol), dry PhMe (1.5 mL, 0.2 M) were subjected to **General Procedure 4**. The title compound was afforded after purification by FCC (8% Et_2O/pentane) as a white solid (64 mg, 0.22 mmol, 73% yield, 99:1 e.r.).

^1H NMR (400 MHz, $CDCl_3$) δ 7.69 – 7.65 (m, 2H, ArCH x 2), 7.55 – 7.49 (m, 1H, ArCH), 7.46 – 7.41 (m, 2H, ArCH x 2), 7.17 (d, *J* = 8.5 Hz, 2H, ArCH x 2), 6.88 (d, *J* = 8.5 Hz, 2H, ArCH x 2), 6.68 – 6.63 (m, 1H, H_2), 3.81 (s, 3H, OCH_3 x 3), 2.88 – 2.80 (m, 1H, H_4), 2.77 – 2.68 (m, 1H, H_{6a}), 2.62 – 2.42 (m, 2H, H_{3a} and H_{6b}), 2.42 – 2.31 (m, 1H, H_{3b}), 2.10 (ddtd, *J* = 13.0, 5.5, 2.5, 2.0 Hz, 1H, H_{5a}), 1.80 (dddd, *J* = 13.0, 12.0, 11.0, 5.5 Hz, 1H, H_{5b}).

^{13}C NMR (101 MHz, $CDCl_3$) δ 198.0, 158.2, 143.3, 138.8, 138.7, 138.2, 131.5, 129.3, 128.2, 127.8, 114.1, 55.4, 38.7, 34.5, 29.7, 24.9.

HRMS (ESI+): Found $[M+H]^+$ = 293.1536; $C_{20}H_{21}O_2$ requires 293.1536, Δ –0.14 ppm.

IR (film) ν_{max}/cm^{-1} : 2931, 1642, 1512, 1246, 1178, 1035, 827, 703, 662.

m.p.: 105-107 ^oC

$\alpha_{25}{}^D$= −85.1 (c = 1.0, $CHCl_3$).

HPLC: Enantiomeric excess was determined by HPLC with a Chiralpak® IG column (97:3 hexane:IPA, 1.0 mL min^{-1}, 254 nm, room temperature), t_r (minor) = 43.4 min, t_r (major) = 45.0 min.

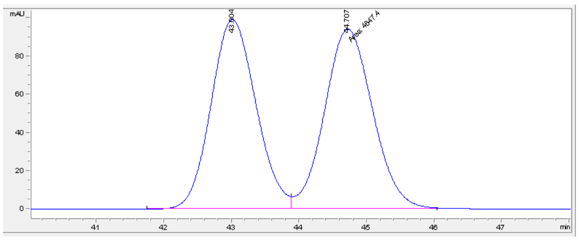

#	Time	Type	Area	Height	Width	Area%	Symmetry
1	43.004	BV	4578.4	99	0.7198	49.626	0.875
2	44.707	MF	4647.4	94	0.8243	50.374	0.896

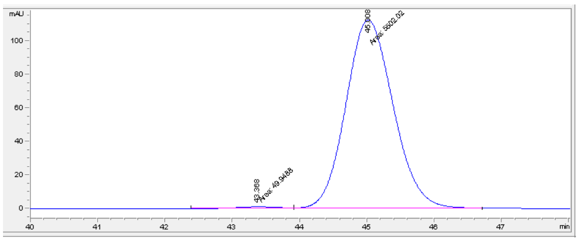

#	Time	Type	Area	Height	Width	Area%	Symmetry
1	43.368	MF	49.9	1.1	0.7588	0.900	0
2	45.008	MF	5502	112.5	0.8153	99.100	0.867

(*S*)-Phenyl(4-(prop-1-en-2-yl)cyclohex-1-en-1-yl)methanone, 2ag

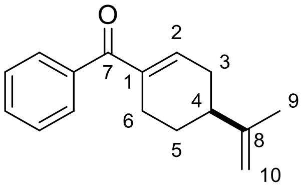

Tetrahydropyran **4ag** (73 mg, 0.30 mmol, 1 equiv.), aluminium tert-butoxide (75 mg, 1 equiv., 0.30 mmol), dry PhMe (1.5 mL, 0.2 M) were subjected to **General Procedure 4**. The title compound was afforded after purification by FCC (5% Et_2O/pentane) as a colourless oil (48 mg, 0.21 mmol, 71% yield, 98:2 e.r.).

^{1}H NMR (400 MHz, $CDCl_3$) δ 7.66 – 7.61 (m, 2H, ArCH x 2), 7.53 – 7.47 (m, 1H, ArCH), 7.45 – 7.38 (m, 2H, ArCH x 2), 6.62 – 6.57 (m, 1H, H_2), 4.79 (p, *J* = 1.5 Hz, 1H, H_{10a}), 4.77 – 4.75 (m, 1H, H_{10b}), 2.70 – 2.61 (m, 1H, H_{6a}), 2.45 – 2.32 (m, 2H, H_{6b} and H_{3a}), 2.30 – 2.14 (m, 2H, H_{3b} and H_4), 2.02 – 1.94 (m, 1H, H_{5a}), 1.78 (t, *J* = 1.0 Hz, 3H, H_9 x 3), 1.63 – 1.49 (m, 1H, H_{5b}).

^{13}C NMR (101 MHz, $CDCl_3$) δ 198.0, 148.9, 143.4, 138.8, 138.6, 131.5, 129.3, 128.2, 109.5, 40.4, 31.6, 27.1, 24.5, 20.9.

HRMS (ESI+): Found $[M+H]^+$ = 227.1431; $C_{16}H_{19}O$ requires 227.1430, Δ 0.38 ppm.

IR (film) ν_{max}/cm^{-1} : 2920, 1644, 1446, 1377, 1312, 1262, 890, 868, 703, 664.

$\alpha_{25}{}^D$= −110.1 (c = 1.0, $CHCl_3$).

HPLC: Enantiomeric excess was determined by HPLC with a Chiralpak® IH column (99:1 hexane:IPA, 1.0 mL min^{-1}, 254 nm, room temperature), t_r (minor) = 8.1 min, t_r (major) = 9.2 min.

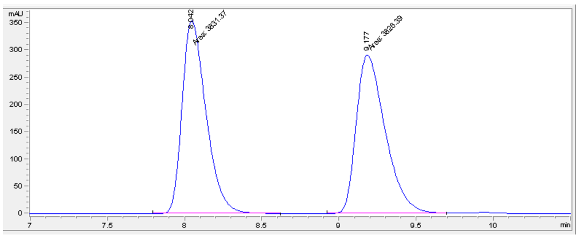

#	Time	Type	Area	Height	Width	Area%	Symmetry
1	8.042	MF	3831.4	355.6	0.1796	50.033	0.657
2	9.177	MF	3826.4	292.2	0.2182	49.967	0.603

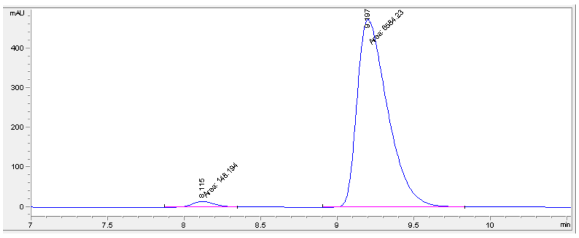

#	Time	Type	Area	Height	Width	Area%	Symmetry
1	8.115	MF	148.2	14.3	0.1724	2.201	0.772
2	9.197	MF	6584.2	475	0.231	97.799	0.541

(5-Methylcyclohex-1-en-1-yl)(phenyl)methanone, 2ah

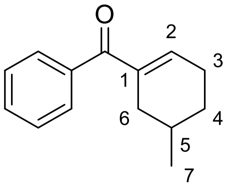

Tetrahydropyran **4ah** (65 mg, 0.30 mmol, 1 equiv.), basic alumina (153 mg, 5 equiv., 1.50 mmol), dry PhMe (1.5 mL, 0.2 M) were subjected to **General Procedure 4**. The title compound was afforded after purification by FCC (5% Et_2O/pentane) as a colourless oil (49 mg, 0.24 mmol, 82% yield, >95:5 r.r.).

^{1}H NMR (400 MHz, $CDCl_3$) δ 7.64 – 7.59 (m, 2H, ArCH x 2), 7.51 – 7.46 (m, 1H, ArCH), 7.44 – 7.37 (m, 2H, ArCH x 2), 6.58 – 6.53 (m, 1H, H_2), 2.68 – 2.58 (m, 1H, H_{6a}), 2.39 – 2.22 (m, 2H, H_3 x 2), 1.98 – 1.87 (m, 1H, H_{6b}), 1.81 – 1.68 (m, 2H, H_{4a} and H_5), 1.31 – 1.19 (m, 1H, H_{4b}), 1.06 (d, J = 6.5 Hz, 3H, H_7).

^{13}C NMR (101 MHz, $CDCl_3$) δ 198.3, 143.8, 139.0, 138.5, 131.4, 129.2, 128.1, 32.4, 30.0, 28.3, 26.4, 21.8.

HRMS (ESI+): Found $[M+H]^+$ = 201.1274; $C_{14}H_{17}O$ requires 201.1274, Δ 0.10 ppm.

IR (film) ν_{max}/cm^{-1}: 2924, 1642, 1432, 1269, 1245, 703, 663.

Phenyl(5-propylcyclohex-1-en-1-yl)methanone, 2ai

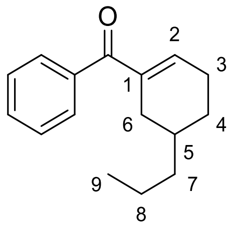

Tetrahydropyran **4ai** (74 mg, 0.30 mmol, 1 equiv.), aluminium *tert*-butoxide (73 mg, 1 equiv., 0.30 mmol), dry PhMe (1.5 mL, 0.2 M) were subjected to **General Procedure 4**. The title compound was afforded after purification by FCC (5% Et_2O/pentane) as a colourless oil (46 mg, 0.20 mmol, 67% yield, >95:5 r.r.).

^1H NMR (400 MHz, $CDCl_3$) δ 7.65 – 7.59 (m, 2H, ArCH x 2), 7.51 – 7.46 (m, 1H, ArCH), 7.43 – 7.37 (m, 2H, ArCH x 2), 6.58 – 6.53 (m, 1H, H_2), 2.70 – 2.61 (m, 1H, H_{6a}), 2.38 – 2.20 (m, 2H, H_3 x 2), 1.98 – 1.87 (m, 1H, H_{6b}), 1.85 – 1.77 (m, 1H, H_{4a}), 1.69 – 1.56 (m, 1H, H_5), 1.48 – 1.18 (m, 5H, H_{4b}, H_7 x 2 and H_8 x 2), 0.93 (t, *J* = 7.0 Hz, 3H, H_9 x 3).

^{13}C NMR (101 MHz, $CDCl_3$) δ 198.3, 144.0, 139.0, 138.6, 131.3, 129.2, 128.1, 38.8, 33.0, 30.6, 28.1, 26.4, 20.1, 14.4.

HRMS (ESI+): Found $[M+H]^+$ = 229.1589; $C_{16}H_{21}O$ requires 229.1587, Δ 0.86 ppm

IR (film) ν_{max}/cm^{-1} 2917, 1643, 1447, 1274, 1252, 703, 663.

(5-Allylcyclohex-1-en-1-yl)(phenyl)methanone, 2aj

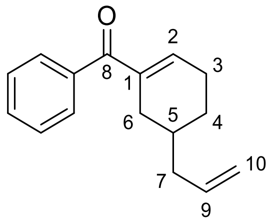

Method 1: Tetrahydropyran **4aj** (73 mg, 0.30 mmol, 1 equiv.), $Al(OtBu)_3$ (74 mg, 1 equiv. 0.30 mmol), dry PhMe (1.5 mL, 0.2 M) were subjected to **General Procedure 4**. The title compound was afforded after purification by FCC (4% Et_2O/pentane) as a colourless oil (56 mg, 0.25 mmol, 83% yield, >95:5 r.r.).

Method 2: A 10 mL flame dried RBF fitted with a reflux condenser was charged with Phosphonate **S21** (180 mg, 0.700 mmol, 1.4 equiv.) and the flask was evacuated and backfilled with N_2 three times. Dry toluene (2.5 mL) was added, and 60% wt. NaH (24 mg, 0.60 mmol, 1.2 equiv.) was added in one portion [care: vigorous release of H_2]. The resulting suspension

was stirred at RT for 20 min, by which time a homogeneous solution had formed. Lactol **S16** (71 mg, 0.50 mmol, 1.0 equiv.) was added and the reaction was heated at 110 °C for 15 h. The reaction was then cooled to RT, and water (3 mL) was added. The resulting mixture was extracted with a 1:1 mixture of pentane/Et_2O (5 x 3 mL) and the combined organic extracts were filtered through a short pad of silica gel, washing with 3 further 3 mL portions of 1:1 pentane/Et_2O. The filtrate was concentrated under reduced pressure and the residue was dissolved in dry PhMe (2.5 mL) and transferred to a flame dried 2–5 mL Biotage® microwave vial and aluminium *tert*-butoxide (185 mg, 0.750 mmol 1.5 equiv.) was added. The headspace was briefly purged with N_2 and the reaction vessel was sealed with a microwave vial cap (containing a Reseal™ septum) and heated at 110 °C for 16 h. The reaction was then cooled to RT, diluted with Et_2O (50 mL), filtered through a plug of silica gel and concentrated *in vacuo*. The title compound was afforded after purification by FCC (5% Et_2O/pentane) as a colourless oil (82 mg, 0.36 mmol, 72% yield over 2 steps, >95:5 r.r.).

^{1}H NMR (400 MHz, $CDCl_3$) δ 7.67 – 7.59 (m, 2H, ArCH x 2), 7.55 – 7.45 (m, 1H, ArCH), 7.45 – 7.36 (m, 2H, ArCH x 2), 6.62 – 6.51 (m, 1H, H_2), 5.86 (ddt, *J* = 17.0, 10.0, 7.0 Hz, 1H, H_9), 5.17 – 4.94 (m, 2H, H_{10} x 2), 2.66 (dddt, *J* = 17.5, 5.0, 2.5, 1.5 Hz, 1H, H_{6a}), 2.42 – 2.05 (m, 4H, H_3 x 2, H_7 x 2), 1.99 (dddt, *J* = 17.5, 10.0, 3.5, 2.5 Hz, 1H, H_{6b}), 1.84 (ddt, *J* = 12.0, 3.5, 2.0 Hz, 1H, H_{4a}), 1.78 – 1.64 (m, 1H, H_5), 1.27 (dddd, *J* = 13.0, 11.0, 10.0, 6.0 Hz, 1H, H_{4b}).

^{13}C NMR (101 MHz, $CDCl_3$) δ 198.2, 143.8, 138.9, 138.4, 136.8, 131.4, 129.3, 128.2, 116.3, 40.7, 33.4, 30.4, 27.5, 26.3.

HRMS (ESI+): Found $[M+H]^+$ = 227.1429; $C_{16}H_{19}O$ requires 227.1430, Δ -0.50 ppm

IR (film) ν_{max}/cm^{-1} 2917, 2859, 1641, 1636, 1446, 1277, 911, 701.

Phenyl(5-(prop-2-yn-1-yl)cyclohex-1-en-1-yl)methanone, 2ak

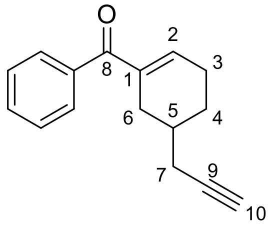

Tetrahydropyran **4ak** (73 mg, 0.30 mmol, 1 equiv.), Al(O*t*Bu)$_3$ (74 mg, 1 equiv. 0.30 mmol), dry PhMe (1.5 mL, 0.2 M) were subjected to **General Procedure 4**. The title compound was

afforded after purification by FCC (4% Et_2O/pentane) as a colourless oil (54 mg, 0.24 mmol, 80% yield, >95:5 r.r.).

^{1}H NMR (400 MHz, $CDCl_3$) δ 7.66 – 7.59 (m, 2H, ArCH x 2), 7.54 – 7.45 (m, 1H, ArCH), 7.46 – 7.36 (m, 2H, ArCH x 2), 6.61 – 6.53 (m, 1H, H_2), 2.73 (dddd, *J* = 17.5, 5.0, 2.0, 1.0 Hz, 1H, H_{6a}), 2.46 – 2.19 (m, 4H, H_3 x 2 H_7 x 2), 2.10 (dddt, *J* = 17.5, 10.0, 3.5, 2.5 Hz, 1H, H_{6b}), 2.01 (t, *J* = 2.5 Hz, 1H, H_{10}), 1.99 – 1.81 (m, 2H, H_{4a}, H_5), 1.42 (dddd, *J* = 13.0, 11.0, 10.0, 6.5 Hz, 1H, H_{4b}).

^{13}C NMR (101 MHz, $CDCl_3$) δ 197.9, 143.6, 138.8, 138.0, 131.5, 129.3, 128.2, 82.7, 69.7, 32.9, 29.9, 27.1, 26.1, 25.2.

HRMS (ESI+): Found $[M+H]^+$ = 225.1275; $C_{16}H_{17}O$ requires 225.1274, Δ 0.49 ppm

IR (film) ν_{max}/cm^{-1} 2941, 2918, 1666, 1642, 1279, 1260, 748, 707.

(5-Benzylcyclohex-1-en-1-yl)(phenyl)methanone, 2al

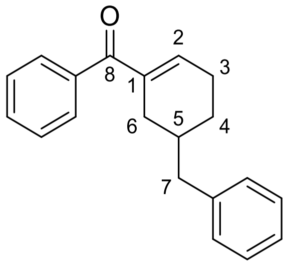

Tetrahydropyran **4al** (88 mg, 0.30 mmol, 1 equiv.), aluminium tert-butoxide (75 mg, 1 equiv., 0.30 mmol), dry PhMe (1.5 mL, 0.2 M) were subjected to **General Procedure 4**. The title compound was afforded after purification by FCC (4% Et_2O/pentane) as a colourless oil (60 mg, 0.22 mmol, 72% yield, >95:5 r.r by Q^{1}H NMR).

^{1}H NMR (400 MHz, $CDCl_3$) δ 7.66 – 7.57 (m, 2H, ArCH x 2), 7.53 – 7.46 (m, 1H, ArCH), 7.44 – 7.37 (m, 2H, ArCH x 2), 7.34 – 7.27 (m, 2H, ArCH x 2), 7.25 – 7.17 (m, 3H, ArCH x 3), 6.61 – 6.52 (m, 1H, H_2), 2.76 (dd, *J* = 13.5, 6.5 Hz, 1H, H_{7a}), 2.69 – 2.57 (m, 2H, H_{6a}, H_{7b}), 2.43 – 2.16 (m, 2H, H_3 x 2), 2.13 – 2.00 (m, 1H, H_{6b}), 2.00 – 1.86 (m, 1H, H_5), 1.87 – 1.75 (m, 1H, H_{4a}), 1.36 – 1.23 (m, 1H, H_{4b}).

^{13}C NMR (101 MHz, $CDCl_3$) δ 198.1, 143.8, 140.6, 138.9, 138.3, 131.4, 129.3, 129.2, 128.4, 128.1, 126.1, 42.9, 35.5, 30.6, 27.5, 26.3.

HRMS (ESI+): Found $[M+H]^+$ = 277.1586; $C_{20}H_{21}O$ requires 277.1587, Δ -0.22 ppm

IR (film) ν_{max}/cm^{-1} 2916, 1642, 1494, 1279, 745, 663.

(3-Methylcyclohex-1-en-1-yl)(phenyl)methanone, 2am

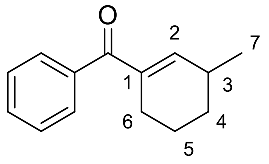

2am

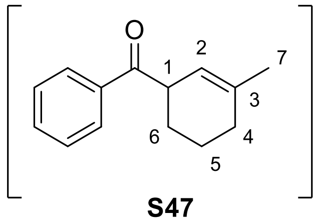

S47

Tetrahydropyran **4am** (65 mg, 0.30 mmol, 1 equiv.), Al(O*t*Bu)$_3$ (74 mg, 1 equiv. 0.30 mmol), dry PhMe (1.5 mL, 0.2 M) were subjected to **General Procedure 4**. Purification by FCC (5% Et_2O/pentane) afforded the title compound **2am** as a colourless oil (39 mg, 0.19 mmol, 65% yield) along with skipped enone **S47** as a colourless oil (5 mg, 0.02 mmol, 8% yield).

<u>Data for **2am**:</u>

^{1}H NMR (400 MHz, $CDCl_3$) δ 7.67 – 7.61 (m, 2H, ArCH x 2), 7.52 – 7.47 (m, 1H, ArCH), 7.44 – 7.39 (m, 2H, ArCH x 2), 6.40 – 6.37 (m, 1H, H_2), 2.50 – 2.27 (m, 3H, H_3 and H_6 x 2), 1.92 – 1.82 (m, 2H, H_{5a} and H_{4a}), 1.67 – 1.56 (m, 1H, H_{5a}), 1.32 – 1.19 (m, 1H, H_{4a}), 1.06 (d, *J* = 7.0 Hz, 3H, H_7 x 3).

^{13}C NMR (101 MHz, $CDCl_3$) δ 198.6, 149.2, 138.8, 137.9, 131.5, 129.4, 128.2, 31.4, 30.5, 24.3, 21.1, 21.0.

HRMS (ESI+): Found $[M+H]^+$ = 201.1274; $C_{14}H_{17}O$ requires 201.1274, Δ -0.13 ppm

IR (film) ν_{max}/cm^{-1} 2929, 1645, 1446, 1271, 1246, 709.

<u>Data for **S47**:</u>

^{1}H NMR (400 MHz, $CDCl_3$) δ 7.99 – 7.94 (m, 2H, ArCH x 2), 7.58 – 7.53 (m, 1H, ArCH), 7.50 – 7.44 (m, 2H, ArCH x 2), 5.44 (dq, *J* = 3.0, 1.5 Hz, 1H, H_2), 4.11 – 4.00 (m, 1H, H_1), 2.07 – 1.62 (m, 9H, H_4 x 2, H_5 x 2, H_6 x 2 and H_7 x 3).

^{13}C NMR (101 MHz, $CDCl_3$) δ 202.3, 137.6, 136.6, 132.9, 128.7, 128.7, 118.9, 44.4, 29.9, 25.6, 24.2, 21.5.

HRMS (ESI+): Found $[M+H]^+$ = 201.1275; $C_{14}H_{17}O$ requires 201.1274, Δ 0.63 ppm

IR (film) ν_{max}/cm^{-1} 2932, 1683, 1448, 1211, 701.

(2-Methylcyclohex-1-en-1-yl)(phenyl)methanone, 2an

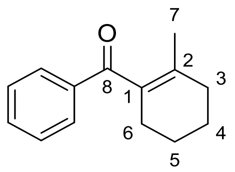

Method 1: Tetrahydropyran **4an** (65 mg, 0.30 mmol, 1 equiv.), Al(O*t*Bu)$_3$ (74 mg, 1 equiv. 0.30 mmol), dry PhMe (1.5 mL, 0.2 M) were subjected to **General Procedure 4**. The title compound was afforded after purification by FCC (3% Et_2O/pentane) as a colourless oil (48 mg, 0.27 mmol, 80% yield).

Method 2: A 2-5 mL Biotage® microwave vial was charged with hept-6-en-2-ol[34] (34 mg, 0.30 mmol, 1 equiv.), 1-phenylprop-2-en-1-one[35] (79 mg, 0.60 mmol, 2 equiv.), 1,2-dichloroethane (3 mL, 0.1 M), and Hoveyda-Grubbs Catalyst® M720 (19 mg, 0.030 mmol, 10 mol%). The vial was flushed with nitrogen and then sealed with a microwave vial cap (containing a Reseal™ septum) and heated at 100 ^oC for 15 h. The reaction was then cooled to RT and the vial was opened and aluminium *tert*-butoxide (222 mg, 0.901 mmol, 3 equiv.) was added. The headspace was briefly purged with N_2 and the reaction vessel was resealed with a fresh microwave vial cap (containing a Reseal™ septum) and heated at 110 ^oC for 16 h. The reaction was then cooled to RT, diluted with Et_2O (20 mL), filtered through a plug of silica gel and concentrated *in vacuo*. The title compound was afforded after purification by FCC (5% Et_2O/pentane) as a colourless oil (35 mg, 0.17 mmol, 58% yield).

^{1}H NMR (400 MHz, $CDCl_3$) δ 7.93 – 7.84 (m, 2H, ArCH x 2), 7.59 – 7.50 (m, 1H, ArCH), 7.50 – 7.40 (m, 2H, ArCH x 2), 2.25 – 2.18 (m, 2H, H_6 x 2), 2.12 – 2.08 (m, 2H, H_3 x 2), 1.79 – 1.65 (m, 4H, H_5 x 2, H_4 x 2), 1.54 (tt, *J* = 2.0, 1.0 Hz, 3H, H_7 x 3).

^{13}C NMR (101 MHz, $CDCl_3$) δ 201.7, 137.1, 134.9, 133.1, 132.5, 129.4, 128.8, 31.3, 27.6, 22.7, 22.4, 21.3.

HRMS (ESI+): Found $[M+H]^+$ = 201.1276; $C_{14}H_{17}O$ requires 201.1274, Δ 0.85 ppm

IR (film) ν_{max}/cm^{-1}: 2981, 2931, 1659, 1596, 1579, 1448, 1380.

Phenyl(3,4,5,6-tetrahydro-[1,1'-biphenyl]-2-yl)methanone, 2ao

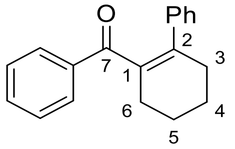

Tetrahydropyran **4ao** (84 mg, 0.30 mmol, 1 equiv.), Al(O*t*Bu)$_3$ (74 mg, 1 equiv. 0.30 mmol), dry PhMe (1.5 mL, 0.2 M) were subjected to **General Procedure 4**. The title compound was afforded after purification by FCC (3% Et_2O/pentane) as a white solid (49 mg, 0.19 mmol, 62% yield).

^{1}H NMR (400 MHz, $CDCl_3$) δ 7.71 – 7.65 (m, 2H, ArCH x 2), 7.36 – 7.29 (m, 1H, ArCH), 7.24 – 7.18 (m, 2H, ArCH x 2), 7.12 – 6.96 (m, 5H, ArCH x 5), 2.54 – 2.44 (m, 4H, H_6 x 2, H_3 x 2), 1.95 – 1.78 (m, 4H, H_5 x 2, H_4 x 2).

^{13}C NMR (101 MHz, $CDCl_3$) δ 201.5, 142.0, 140.0, 137.0, 135.5, 132.5, 129.3, 128.1, 128.0, 127.3, 31.0, 28.2, 23.0, 22.2. *N.B.* the peak at 128.0 ppm corresponds to two overlapping signals.

HRMS (ESI+): Found $[M+H]^+$ = 263.1431; $C_{19}H_{19}O$ requires 263.1430, Δ 0.21 ppm

IR (film) ν_{max}/cm^{-1}: 2980, 1650, 1597, 1579, 1447, 1383.

m.p.: 75-77 °C

Phenyl(2,4,4-trimethylcyclohex-1-en-1-yl)methanone, 2ap

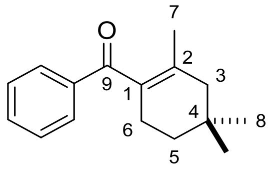

Tetrahydropyran **4ap** (74 mg, 0.30 mmol, 1 equiv.), basic alumina (150 mg, 5 equiv., 1.50 mmol), dry PhMe (1.5 mL, 0.2 M) were subjected to **General Procedure 4**. The title compound was afforded after purification by FCC (3% Et_2O/pentane) as a colourless oil (45 mg, 0.20 mmol, 66% yield).

^{1}H NMR (400 MHz, $CDCl_3$) δ 7.90 – 7.83 (m, 2H, ArCH x 2), 7.59 – 7.50 (m, 1H, ArCH), 7.50 – 7.40 (m, 2H, ArCH x 2), 2.27 (th, *J* = 6.0, 2.0 Hz, 2H, H_6 x 2), 1.89 (br s, H_3 x 2), 1.52 (tt, *J* = 2.0, 1.0 Hz, 3H, H_7 x 3), 1.46 (t, *J* = 6.5 Hz, 2H, H_5 x 2), 1.01 (s, 6H, H_8 x 6).

^{13}C NMR (101 MHz, $CDCl_3$) δ 201.7, 137.4, 134.1, 133.1, 131.1, 129.3, 128.8, 45.4, 35.1, 29.3, 28.5, 25.5, 21.5.

HRMS (ESI+): Found $[M+H]^+$ = 229.1587; $C_{16}H_{21}O$ requires 229.1587, Δ 0.15 ppm

IR (film) ν_{max}/cm^{-1}: 2951, 2915, 2866, 1661, 1596, 1580.

Phenyl(2,4,4-trimethylcyclohex-1-en-1-yl)methanone, 2aq

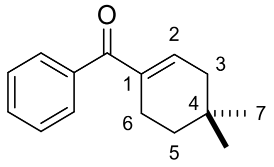

Tetrahydropyran **4aq** (70 mg, 0.30 mmol, 1 equiv.), Al(O*t*Bu)$_3$ (74 mg, 1 equiv., 0.30 mmol), dry PhMe (1.5 mL, 0.2 M) were subjected to **General Procedure 4**. The title compound was afforded after purification by FCC (3% Et_2O/pentane) as a colourless oil (50 mg, 0.23 mmol, 78% yield).

^{1}H NMR (400 MHz, $CDCl_3$) δ 7.66 – 7.59 (m, 2H, ArCH x 2), 7.53 – 7.45 (m, 1H, ArCH), 7.45 – 7.36 (m, 2H, ArCH x 2), 6.51 (tt, *J* = 4.0, 1.5 Hz, 1H, H_2), 2.46 (ttd, *J* = 6.5, 2.5, 1.5 Hz, 2H, H_6 x 2), 2.05 (dt, *J* = 4.5, 2.5 Hz, 2H, H_3 x 2), 1.50 (t, *J* = 6.5 Hz, 2H, H_5 x 2), 0.97 (s, 6H, H_7 x 6).

^{1}H NMR (500 MHz, *p*-Xylene-*d$_{10}$*, 298 K) δ 7.75 – 7.68 (m, 2H, ArCH x 2), 7.33 – 7.26 (m, 1H, ArCH), 7.23 (m, 2H, ArCH x 2), 6.36 (tt, *J* = 4.0, 1.5 Hz, 1H, H_2), 2.60 (tq, *J* = 6.5, 2.5 Hz, 2H, H_6 x 2), 1.80 (dt, *J* = 4.5, 2.5 Hz, 2H, H_3 x 2), 1.38 (t, *J* = 6.5 Hz, 2H, H_5 x 2), 0.94 (s, 6H, H_7 x 6).

^{13}C NMR (101 MHz, $CDCl_3$) δ 198.2, 143.2, 138.9, 137.6, 131.4, 129.2, 128.1, 40.2, 35.0, 28.7, 28.3, 21.9.

HRMS (ESI+): Found $[M-H]^+$ = 215.1431; $C_{15}H_{19}O$ requires 215.1430, Δ 0.40 ppm.

IR (film) ν_{max}/cm^{-1}: 2981, 1644, 1598, 1578, 1446, 1381.

1,4(1,4)-Dibenzenacyclohexaphane-1^2-yl(4,4-dimethylcyclohex-1-en-1-yl)methanone, 2ar

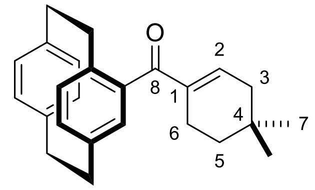

Tetrahydropyran **4ar** (109 mg, 0.300 mmol, 1 equiv.), Al(O*t*Bu)$_3$ (74 mg, 1 equiv. 0.30 mmol), dry PhMe (1.5 mL, 0.2 M) were subjected to **General Procedure 4**. The title compound was afforded after purification by FCC (3% Et_2O/pentane) as a yellow solid (70 mg, 0.20 mmol, 68% yield).

^{1}H NMR (400 MHz, $CDCl_3$) δ 6.77 (dd, *J* = 8.0, 1.5 Hz, 1H, ArCH), 6.62 – 6.51 (m, 4H, ArCH x 4), 6.45 (d, *J* = 8.0 Hz, 1H, ArCH), 6.35 (dd, *J* = 8.0, 1.5 Hz, 1H, ArCH), 6.16 (tt, *J* = 4.0, 1.5 Hz, 1H, H_2), 3.32 – 2.79 (m, 8H, Paracyclophane-CH_2 x 8), 2.57 – 2.35 (m, 2H, H_6 x 2), 1.90 (tt, *J* = 4.0, 2.5 Hz, 2H, H_3 x 2), 1.48 (t, *J* = 6.5 Hz, 2H, H_5 x 2), 0.94 (s, 3H, H_{7a} x 3), 0.92 (s, 3H, H_{7b} x 3).

^{13}C NMR (101 MHz, $CDCl_3$) δ 198.2, 143.4, 140.3, 139.8, 139.3, 139.2, 139.2, 136.8, 135.3, 135.2, 133.1, 132.9, 132.6, 132.2, 131.6, 40.2, 35.7, 35.3, 35.2, 35.1, 34.9, 28.7, 28.5, 28.1, 21.6.

HRMS (ESI+): Found $[M+H]^+$ = 345.2214; $C_{25}H_{29}O$ requires 345.2213, Δ 0.42 ppm

IR (film) ν_{max}/cm^{-1}: 2980, 1638, 1462, 1383, 1262, 1152.

m.p.: 100-101 °C

Cyclohex-1-en-1-yl(phenyl)methanone, 2as

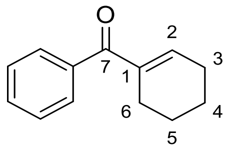

Tetrahydropyran **4as** (61 mg, 0.30 mmol, 1 equiv.), Al(O*t*Bu)$_3$ (74 mg, 1 equiv. 0.30 mmol), dry PhMe (1.5 mL, 0.2 M) were subjected to **General Procedure 4**. The title compound was afforded after purification by FCC (3% Et_2O/pentane) as a yellow solid (50 mg, 0.27 mmol, 89% yield). The spectral data matched that previously reported in the literature.[43]

^{1}H NMR (400 MHz, CDCl$_3$) δ 7.66 – 7.57 (m, 2H, ArCH x 2), 7.53 – 7.45 (m, 1H, ArCH), 7.44 – 7.36 (m, 2H, ArCH x 2), 6.58 (tt, *J* = 4.0, 1.5 Hz, 1H, H$_2$), 2.47 – 2.38 (m, 2H, H$_6$ x 2), 2.31 – 2.22 (m, 2H, H$_3$ x 2), 1.79 – 1.62 (m, 4H, H$_5$ x 2, H$_4$ x 2).

^{13}C NMR (101 MHz, CDCl$_3$) δ 198.4, 144.2, 138.9, 131.4, 129.3, 128.1, 26.3, 24.1, 22.2, 21.8. N.B. The peak at δ = 138.9 ppm corresponds to two overlapping signals.

(6-Methylcyclohex-1-en-1-yl)(phenyl)methanone, 2at

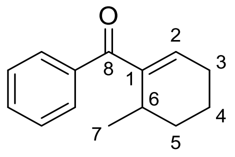

Tetrahydropyran **4at** (65 mg, 0.30 mmol, 1 equiv.), Al(O*t*Bu)$_3$ (75 mg, 0.30 mmol, 1 equiv.), dry PhMe (1.5 mL, 0.2M) were subjected to **General Procedure 4**. The title compound was afforded after purification by FCC (0 -> 10% TMBE/cyclohexane) as a colourless oil (50 mg, 0.25 mmol, 83% yield).

^{1}H NMR (400 MHz, CDCl$_3$) δ 7.73 – 7.62 (m, 2H, ArCH x 2), 7.55 – 7.47 (m, 1H, ArCH), 7.45 – 7.37 (m, 2H, ArCH x 2), 6.40 (td, *J* = 4.0, 1.5 Hz, 1H, H$_2$), 3.09 – 2.88 (m, 1H, H$_6$), 2.37 – 2.05 (m, 2H, H$_3$ x 2), 1.86– 1.50 (m, 4H, H$_4$ x 2, H$_5$ x 2), 1.07 (d, *J* = 7.0 Hz, 3H, H$_7$ x 3).

^{13}C NMR (101 MHz, CDCl$_3$) δ 198.6, 144.1, 141.8, 139.2, 131.7, 129.5, 128.2, 30.2, 28.1, 26.3, 19.9, 18.6.

HRMS (ESI+): Found $[M+H]^+$ = 201.1273; $C_{14}H_{17}O$ requires 201.1274, Δ -0.45ppm

IR (film) ν_{max}/cm^{-1} 2929, 2869, 1646, 1447, 1267, 721, 699.

(6-butylcyclohex-1-en-1-yl)(phenyl)methanone 2au

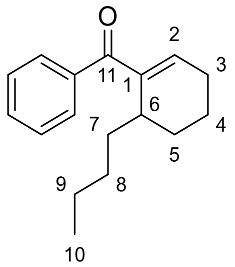

Tetrahydropyran **4au** (78 mg, 0.30 mmol, 1 equiv.), aluminium tert-butoxide (75 mg, 0.30 mmol, 1 equiv.), dry toluene (1.5 mL, 0.2M) were subjected to **General Procedure 4**. The title compound was afforded after purification by FCC (0 -> 10% TMBE/cyclohexane) as a colourless oil (49 mg, 0.20 mmol, 67% yield).

^{1}H NMR (400 MHz, $CDCl_3$) δ 7.73 – 7.64 (m, 2H, ArCH x 2), 7.54 – 7.46 (m, 1H, ArCH), 7.46 – 7.36 (m, 2H, ArCH x 2), 6.38 (td, *J* = 4.0, 1.0 Hz, 1H, H_2), 3.08 – 2.75 (m, 1H, H_6), 2.39 – 2.02 (m, 2H, H_3 x 2), 1.80 – 1.54 (m, 4H, H_4 x 2, H_5 x 2), 1.53 – 1.39 (m, 1H, H_{7a}), 1.42 – 1.13 (m, 5H, H_{7b}, H_8 x 2, H_9 x 2), 0.85 (t, *J* = 7.0 Hz, 3H, H_{10} x 3).

^{13}C NMR (101 MHz, $CDCl_3$) δ 198.6, 143.7, 141.8, 139.0, 131.8, 129.6, 128.2, 33.5, 32.8, 29.8, 26.4, 26.3, 22.9, 18.4, 14.2.

HRMS (ESI+): Found $[M+H]^+$ = 243.1741; $C_{17}H_{23}O$ requires 243.1743, Δ -0.82ppm

IR (film) ν_{max}/cm^{-1} 2929, 2858, 1648, 1447, 1267, 902, 719.

(4,4-Dimethylcyclohex-1-en-1-yl)(3-methoxyphenyl)methanone, 2av

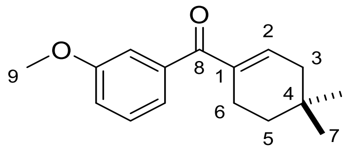

Tetrahydropyran **4av** (79 mg, 0.30 mmol, 1 equiv.), aluminium *tert*-butoxide (73 mg, 1 equiv., 0.30 mmol), dry PhMe (1.5 mL, 0.2 M) were subjected to **General Procedure 4**. The title compound was afforded after purification by FCC (5% Et_2O/pentane) as a colourless oil (63 mg, 0.26 mmol, 86% yield).

^{1}H NMR (400 MHz, $CDCl_3$) δ 7.31 (t, *J* = 7.8 Hz, 1H, ArCH), 7.21 – 7.15 (m, 2H, ArCH x 2), 7.03 (ddd, *J* = 8.0, 2.5, 1.0 Hz, 1H, ArCH), 6.53 (tt, *J* = 4.0, 1.5 Hz, 1H, H_2), 3.84 (s, 3H, OCH_3 x 3), 2.45 (tq, *J* = 6.5, 2.5 Hz, 2H, H_6 x 2), 2.04 (dt, *J* = 4.5, 2.5 Hz, 2H, H_3 x 2), 1.49 (t, *J* = 6.5 Hz, 2H, H_5 x 2), 0.97 (s, 6H, H_7 x 6).

^{13}C NMR (101 MHz, $CDCl_3$) δ 197.9, 159.5, 143.3, 140.2, 137.6, 129.1, 121.8, 117.5, 114.0, 55.5, 40.2, 35.0, 28.7, 28.3, 22.0.

HRMS (ESI+): Found $[M+H]^+$ = 245.1537; $C_{16}H_{21}O_2$ requires 245.1536, Δ 0.33 ppm.

IR (film) ν_{max}/cm^{-1}: 2953, 1645, 1579, 1429, 1271, 1044, 793, 769, 722.

3,3-Dimethyl-3,4,4a,5,6,7-hexahydronaphthalen-1(2*H*)-one 7

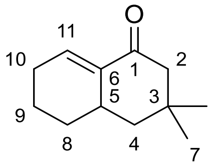

Method 1: 3-(4-Hydroxybutyl)-5,5-dimethylcyclohex-2-en-1-one **6** (59 mg, 0.30 mmol, 1.0 eq.), aluminium *tert*-butoxide (74 mg, 1.0 equiv., 0.30 mmol), dry PhMe (1.5 mL) were subjected to **General Procedure 4**. The title compound was afforded after purification by FCC (5% Et_2O/pentane) as a colourless oil (23 mg, 0.13 mmol, 43% yield).

Method 2: 3-(4-Hydroxybutyl)-5,5-dimethylcyclohex-2-en-1-one **6** (79 mg, 0.30 mmol, 1 equiv.), alumina (153 mg, 5.0 equiv., 1.5 mmol), dry PhMe (1.5 mL) were subjected to **General Procedure 4**. The title compound was afforded after purification by FCC (5% Et_2O/pentane) as a colourless oil (22 mg, 0.12 mmol, 41% yield).

^1H NMR (400 MHz, $CDCl_3$) δ 6.68 – 6.61 (m, 1H, H_{11}), 2.57 – 2.41 (m, 1H, H_5), 2.34 – 2.09 (m, 4H, H_{2a}, H_{10} x 2, H_{2b}), 1.91 – 1.76 (m, 2H, H_{8a}, H_{9a}), 1.71 – 1.46 (m, 2H, H_{4a}, H_{9b}), 1.35 – 1.13 (m, 2H, H_{4b}, H_{8b}), 1.02 (s, 3H, H_{7a} x 3), 0.98 (s, 3H, H_{7b} x 3).

^{13}C NMR (101 MHz, $CDCl_3$) δ 202.0, 139.2, 135.5, 54.0, 44.9, 33.6, 32.0, 32.0, 30.5, 26.3, 26.3, 21.8.

HRMS (ESI+): Found $[M+H]^+$ = 179.1432; $C_{12}H_{19}O$ requires 179.1430, Δ 0.86 ppm.

IR (film) ν_{max}/cm^{-1}: 2955, 1689, 1621, 1423, 1368, 1316

4. Mechanistic Experiments

4.1. Monitoring the Reaction by ^{1}H-NMR

An oven dried 5 mL volumetric flask was charged with tetrahydropyran **4aq** (232 mg, 1.00 mmol) and 1,3,5-trimethoxybenzene (168 mg, 1.00 mmol) and the volume was made up to 5 mL with *p*-xylene-d_{10}. An oven dried S400 NMR tube was charged with aluminium *tert*-butoxide (25 mg, 0.10 mmol, 1 equiv.) and 0.50 mL of the above stock solution was added via syringe (1 equiv. of **4aq** and 1 equiv. 1,3,5-trimethoxybenzene). The NMR tube was purged with argon, capped and then placed into a 500 MHz NMR spectrometer (preheated to 110 ^oC). Once the sample was locked and shimmed (<5 min), it was analysed by running ^{1}H-NMR experiments every 70 minutes for 18 h (8 scans, relaxation delay = 25 s).

The following diagnostic signals were integrated to produce the timecourse plot:

1,3,5-trimethoxybenzene: δ = 6.08-6.32 ppm (ArCH)

Tetrahydropyran **4aq**: δ = 3.12-3.34 ppm (H_{7a})

Cyclohexene **2aq**: δ = 6.32-6.45 ppm (H_2)

To analyse the data, the integral for 1,3,5-trimethoxybenzene (δ = 6.08-6.32 ppm) was set at 300. The peak for cyclohexene **2aq** at δ = 6.32-6.45 ppm was found to overlap with the downfield ^{13}C-satelite peak for the 1,3,5-trimethoxybenzene peak at δ = 6.38 ppm. To correct for this, we separately integrated the upfield satellite peak for 1,3,5-trimethoxybenzne (δ = 6.02-6.08 ppm) and subtracted this from the integral for the cyclohexene peak.

The data obtained from this experiment were as follows:

Time/h	Integral standard (6.08-6.32 ppm)	Integral Tetrahydropyran **4aq** (3.12-3.34 ppm)	Integral Cyclohexene **2aq** (6.32-6.45 ppm)	Integral standard ^{13}C satellite (6.02-6.08 ppm)	Corrected Integral for Cyclohexene **2aq**
0.00	300.00	94.80	6.45	2.69	3.76
1.17	300.00	65.05	32.03	2.41	29.62
2.48	300.00	46.41	48.70	2.66	46.04
3.78	300.00	33.99	60.42	2.93	57.49
5.09	300.00	23.96	69.94	3.17	66.76
6.39	300.00	16.17	77.81	3.41	74.40
7.69	300.00	11.82	81.53	3.54	77.98
9.00	300.00	9.47	83.74	3.51	80.23
10.30	300.00	7.94	85.42	3.56	81.85
11.60	300.00	6.67	86.61	3.54	83.07
12.91	300.00	5.53	87.31	3.74	83.58
14.21	300.00	4.91	88.20	3.62	84.58
15.52	300.00	4.29	88.69	3.70	84.99
16.82	300.00	3.94	88.77	3.72	85.06
18.12	300.00	3.62	89.36	3.68	85.67

4.2. Double Label Crossover Experiment

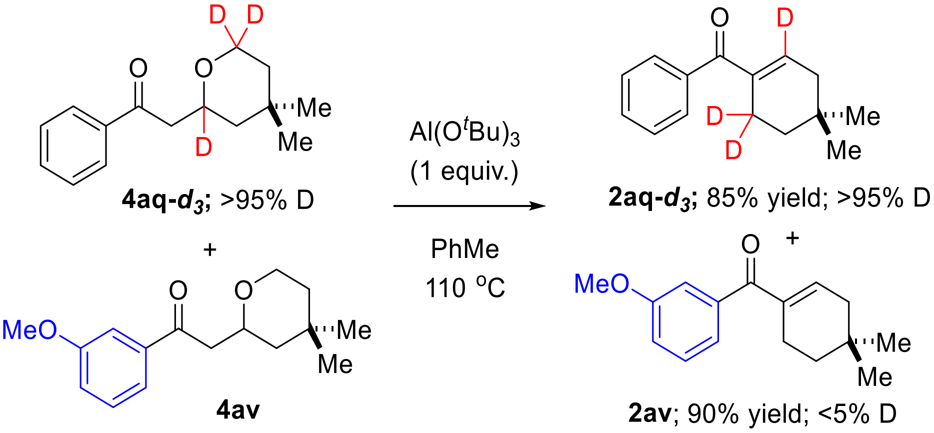

A 2–5 mL flame dried Biotage® microwave vial was charged with tetrahydropyran **4aq-*d*₃** (47 mg, 0.20 mmol, 1 equiv.), tetrahydropyran **4av** (52 mg, 0.20 mmol, 1 equiv.), aluminium *tert*-butoxide (99 mg, 0.40 mmol, 2 equiv.) and dry toluene (2.0 mL). The headspace was briefly purged with N_2 and the reaction vessel was sealed with a microwave vial cap (containing a Reseal™ septum) and heated at 110 °C for 16 h. The reaction was then cooled to RT, diluted with Et_2O (50 mL), filtered through a plug of silica gel and concentrated *in vacuo*. Purification via FCC (3-6% Et_2O/pentane) afforded **2aq-*d*₃** as a colourless oil (37 mg, 0.17 mmol, 85% yield) and **2av** as a colourless oil (44 mg, 0.18 mmol, 90% yield).

*Data for **2aq-d₃** from crossover experiment:*

^{1}H NMR (400 MHz, $CDCl_3$) δ 7.66 – 7.59 (m, 2H, ArCH x 2), 7.51 – 7.46 (m, 1H, ArCH), 7.44 – 7.38 (m, 2H, ArCH x 2), 2.04 (s, 2H, H_3 x 2), 1.48 (s, 2H, H_5 x 2), 0.97 (s, 6H, H_7 x 6). The peaks at δ = 6.51 ppm (H_2) and 2.43 ppm (H_6) integrate to 3% and 6% respectively, implying 97% D incorporation at each position (i.e. for H_6, 97% D for each hydrogen of the methylene group).

^{13}C NMR (101 MHz, $CDCl_3$) δ 198.2, 143.0 (1:1:1 triplet, J = 23.5 Hz, C_2) 138.9, 137.4, 131.4, 129.2, 128.1, 40.1, 34.9, 28.6, 28.3, 21.9 (m, C_6). The ^{13}C peaks corresponding to C_6 and C_2 displayed reduced intensity due to $^2J_{CD}$ coupling.

^{2}H NMR (92 MHz, $CHCl_3$ + 1 drop $CDCl_3$) δ 6.55 (s, 1H, H_2), 2.43 (s, 2H, H_6).

Stack plot showing reference ^{1}H NMR spectrum of **2aq** along with quantitative ^{1}H NMR and ^{2}H NMR spectra of **2aq-*d₃*** from crossover experiment:

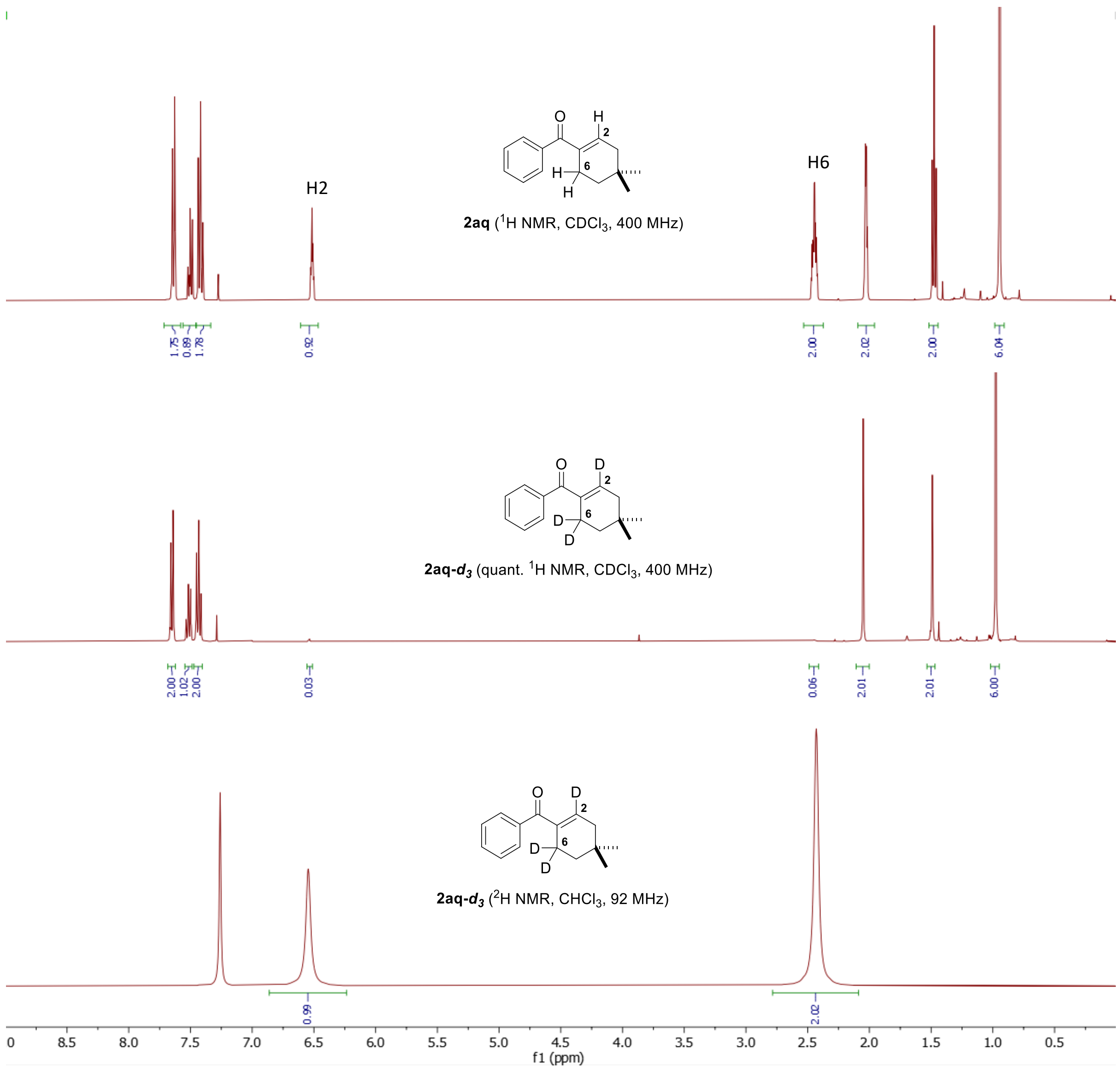

Stack plot showing reference ^{13}C NMR spectrum of **3al** along with ^{13}C NMR spectrum of **3al-*d₃*** from crossover experiment. Expansions show key regions for C_2 and C_6.

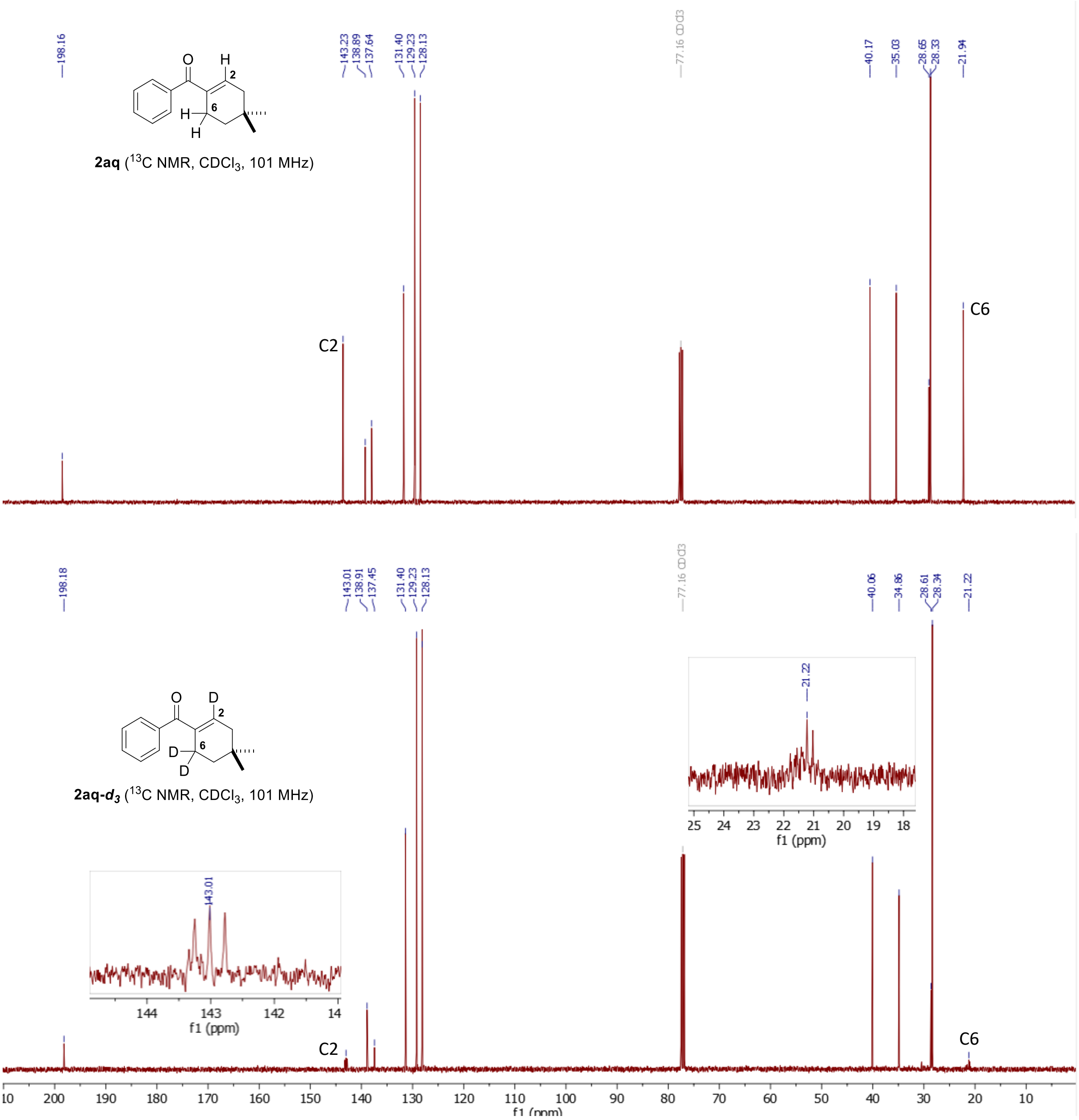

*Data for **2av** from crossover experiment:*

<5% D incorporation at the 2- and 6-positions by quantitative ^1H NMR and ^{13}C NMR.

The ^1H NMR data was identical to that described above for non-deuterated **2av** and the peaks corresponding to H_2 and H_6 integrated to >95% implying <5% D incorporation at these positions:

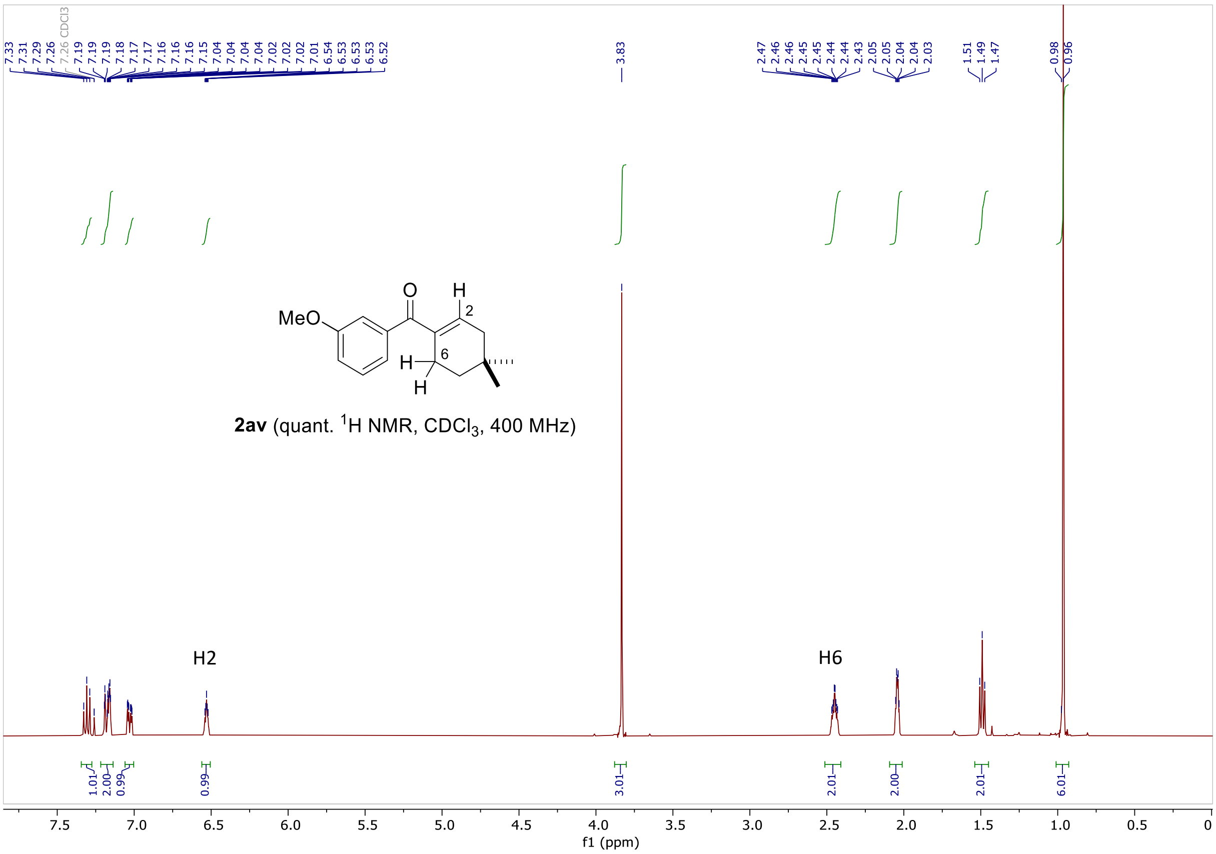

The ^{13}C NMR data was identical to that described above and showed no CD coupling:

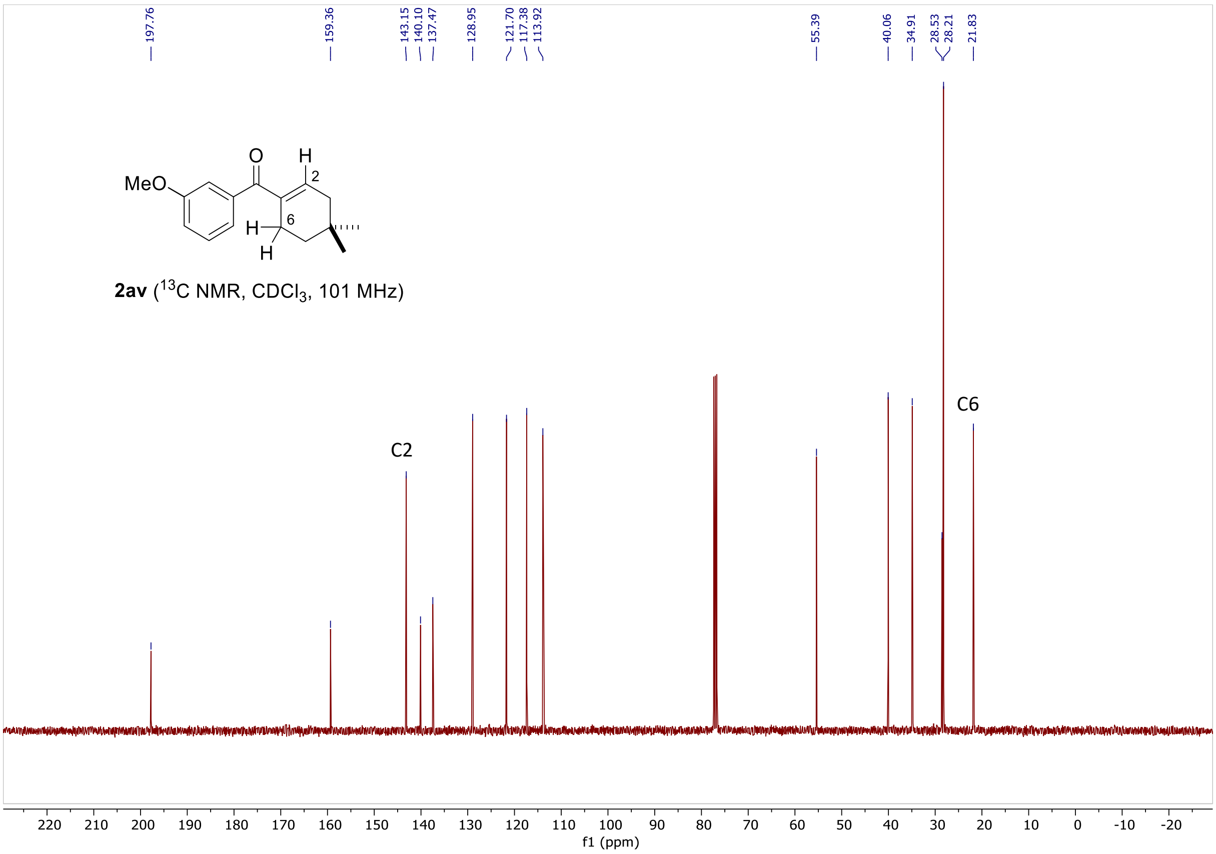

5. Computational Studies

Quantum mechanical calculations were carried out using the ORCA package (v4.2.1)[44]. For a small model system (Scheme 1), benchmark calculations were carried out at the CPCM(PhMe)-DLPNO-CCSD(T)/ma-def2-TZVPP//CPCM(PhMe)-PBE0-D3BJ/def2-SVP level of theory[45]. brief benchmarking study revealed that the CPCM(PhMe)-ωB97X-D3/ma-def2-TZVPP//CPCM(PhMe)-PBE0-D3BJ/def2-SVP level was sufficient to accurately describe the reaction pathways under study here (see Scheme S1, Tables S1–2 and S5–6). Coulomb and Hartree-Fock exchange integrals were obtained using the RIJCOSX approximation[46], with the appropriate auxiliary basis sets and default integration grids, and the toluene solvent environment was modelled using the CPCM implicit solvation model with Gaussian point charges scheme to avoid discontinuities on the potential energy surface[47]. Hirshfeld population analysis was used to calculate partial atomic charges[48]. Generation of minima and transition state (TS) conformers was carried out using CREST, in which metadynamics sampling was employed using GFN-FF[49]. In situations where CREST was unsuccessful at generating reasonable conformers, a systematic rotor search was carried out using UFF implemented in Openbabel[50]. The Python tool Otherm[51], was interfaced with ORCA to obtain scaled free energy corrections, where Grimme's quasi rigid rotor harmonic oscillator (qRRHO) approximation was used to estimate free energies at the experimental temperature of 383 K, and a 1M standard state, using numerical frequencies[52].

Structures were verified to be local minima or transition states through the presence of either zero or one imaginary frequency upon evaluation of the Hessian. A small number of stationary point structures displayed one additional small imaginary frequency ($|\boldsymbol{\nu}_{imag}|$ < 50 cm^{-1}) despite numerous attempts to refine the structures. Numerical tests reveal these additional imaginary frequencies to introduce only small additional errors into our free energy estimates, on the order of 1 kcal mol^{-1}. This error should not impact the qualitative conclusions drawn from this study, as noted elsewhere[53]. This issue has been mentioned in similar studies and was attributed to the implementation of the CPCM solvation module in ORCA.

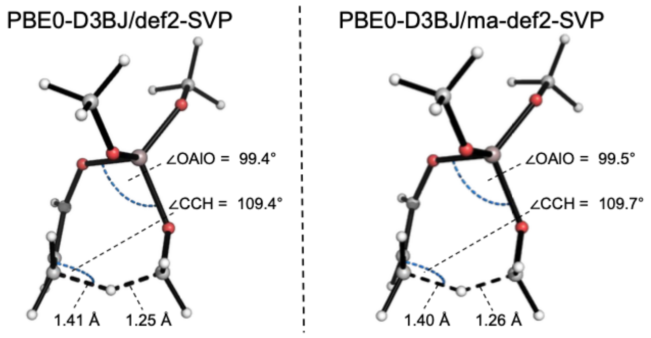

Scheme S1. Validation of the use of the def2-SVP basis set for geometry optimisations, with the PBE0 functional and D3BJ empirical dispersion.

Table S1. Comparison of TS and reaction free energy changes for the hydride transfer shown in Scheme S1, for a selection of DFT functionals relative to CPCM(PhMe)-DLPNO-CCSD(T)/ma-def2-TZVPP, with geometries from the CPCM(PhMe)-PBE0-D3BJ/def2-SVP level.

	Error (ma-def2-TZVPP basis) vs DLPNO-CCSD(T)/ma-def2-TZVPP			
	PBE0-D3BJ	M06-2X	**ωB97X-D3**	B3LYP-D3BJ
$\Delta E^{\ddagger}$	11.9	4.2	**2.6**	6.9
ΔE	1.6	0.6	**0.2**	1.4

Table S2. Comparison of TS and reaction free energy changes for a selection of basis sets employed for the CPCM(PhMe)-ωB97X-D3/*basis* level, relative to CPCM(PhMe)-DLPNO-CCSD(T)/ma-def2-TZVPP.

	Error (ωB97X-D3 functional) vs DLPNO-CCSD(T)/ma-def2-TZVPP				
	def2-SVP	def2-TZVP	def2-TZVPP	**ma-def2-TZVPP**	ma-def2-QZVPP
$\Delta E^{\ddagger}$	5.7	2.8	2.8	**2.6**	2.5
ΔE	2.4	0.4	0.4	**0.2**	0.2

Table S3. Energetics of decomposition of $Al_2(OR)_3$ (R = Me, ^{t}Bu) dimer into monomers. Free energies calculated at 383 K and 1 M. All energies in kcal mol^{-1}.

	**CPCM(PhMe)-PBE0-D3BJ/def2-SVP**				**CPCM(PhMe)-ωB97X-D3/ma-def2-TZVPP**		
**Process**	**ΔE**	**ΔH**	**TΔS**	**ΔG**	**ΔE**	**ΔH**	**ΔG**
$Al_2(OMe)_6$ → 2 $Al(OMe)_3$	75.5	73.5	15.8	57.8	62.1	60.1	44.3
$Al_2(OtBu)_6$ → 2 $Al(O^tBu)_3$	78.8	76.1	21.5	54.6	67.5	64.8	43.4

Table S4. Energetics for the benchmark hydride transfer reaction described in Scheme 1. Free energies calculated at 383 K and 1 M. All energies in kcal mol^{-1}.

	**CPCM(PhMe)-PBE0-D3BJ/def2-SVP**				**CPCM(PhMe)-ωB97X-D3/ma-def2-TZVPP**		
**Process**	**ΔE**	**ΔH**	**TΔS**	**ΔG**	**ΔE**	**ΔH**	**ΔG**
Pre-TS complex → TS	12.2	9.4	-4.3	13.7	21.5	18.7	22.9
Pre-TS complex → post-TS complex	1.8	1.6	-1.1	2.7	3.2	2.9	4.1

Table S5. Benchmark of DFT (ma-def2-TZVPP basis) against DLPNO-CCSD(T)/ma-def2-TZVPP energies for the small benchmark system shown in Scheme 1, using the geometry obtained from the CPCM(PhMe)-PBE0-D3BJ/def2-SVP level. Energies in kcal mol^{-1}.

Method (ma-def2-TZVPP basis)	$\Delta E^{‡}$	ΔE_r	Error in $\Delta E^{‡}$	Error in ΔE_r
Reference: DLPNO-CCSD(T)/ma-def2-TZVPP	24.1	3.4		
PBE0-D3BJ	12.2	1.8	-11.9	-1.6
M06-2X	19.9	2.7	-4.2	-0.6
wB97X-D3	21.5	3.2	-2.6	-0.2
B3LYP-D3BJ	17.3	4.8	-6.9	1.4

Table S6. Benchmark of basis sets (ωB97X-D3 functional) against DLPNO-CCSD(T)/ma-def2-TZVPP energies for the small benchmark system shown in Scheme 1, using the geometry obtained from the CPCM(PhMe)-PBE0-D3BJ/def2-SVP level. Energies in kcal mol^{-1}.

Basis set (with ωB97X-D3 functional)	ΔE‡	ΔEr	Error in ΔE‡	Error in ΔEr
Reference: DLPNO-CCSD(T)/ma-def2-TZVPP	24.1	3.4		
def2-SVP	18.4	1.0	-5.7	-2.4
def2-TZVP	21.3	2.9	-2.8	-0.4
def2-TZVPP	21.3	3.0	-2.8	-0.4
ma-def2-TZVPP	21.5	3.2	-2.6	-0.2
ma-def2-QZVPP	21.6	3.2	-2.5	-0.2

Table S7. Energetics for the hydride transfer of enone **9** facilitated by monomeric ($Al(OMe)_3$) or dimeric ($Al_2(OMe)_3$) complexes. Free energies calculated at 383 K and 1 M. All energies in kcal mol^{-1}.

		CPCM(PhMe)-PBE0-D3BJ/def2-SVP				CPCM(PhMe)-wB97X-D3/ma-def2-TZVPP		
**System**	**Process**	**ΔE**	**ΔH**	**TΔS**	**ΔG**	**ΔE**	**ΔH**	**ΔG**
$Al(OMe)_3$ monomer	*E*-enone + $Al_2(OMe)_6$ → *E-s-cis* pre-TS complex + $Al(OMe)_3$ + MeOH	48.5	46.0	11.1	35.0	42.7	40.3	29.2
	E-enone + $Al_2(OMe)_6$ → *E-s-cis* TS + $Al(OMe)_3$ + MeOH	64.5	59.2	8.8	50.5	65.3	60.1	51.3
	E-enone + $Al_2(OMe)_6$ → *E-s-trans* TS + $Al(OMe)_3$ + MeOH	68.4	62.9	8.4	54.5	70.6	65.1	56.7
	E-enone + $Al_2(OMe)_6$ → *Z-s-cis* TS + $Al(OMe)_3$ + MeOH	62.0	56.9	9.0	47.8	63.1	58.0	49.0
	E-enone + $Al_2(OMe)_6$ → *Z-s-trans* TS + $Al(OMe)_3$ + MeOH	71.0	65.5	8.4	57.0	73.0	67.6	59.1
	E-enone + $Al_2(OMe)_6$ → *E* post-TS + $Al(OMe)_3$ + MeOH	49.8	47.2	10.9	36.3	43.9	41.3	30.4
	E-enone + $Al_2(OMe)_6$ → *Z* post-TS + $Al(OMe)_3$ + MeOH	48.2	45.9	10.8	35.2	41.2	38.9	28.2
$Al_2(OMe)_6$ dimer	*E*-enone + $Al_2(OMe)_6$ → *E-s-trans* pre-TS complex + MeOH	-16.6	-17.0	-8.3	-8.7	-8.1	-8.5	-0.2
	E-enone + $Al_2(OMe)_6$ → *E-s-cis* TS + MeOH	5.9	2.3	-7.6	10.0	17.4	13.9	21.5
	E-enone + $Al_2(OMe)_6$ → *E-s-trans* TS + MeOH	6.3	2.7	-8.2	11.0	17.6	14.0	22.2
	E-enone + $Al_2(OMe)_6$ → *Z-s-cis* TS + MeOH	7.4	3.9	-8.0	11.9	18.6	15.1	23.1
	E-enone + $Al_2(OMe)_6$ → *Z-s-trans* TS + MeOH	12.4	9.1	-8.8	17.9	23.4	20.1	28.9
	E-enone + $Al_2(OMe)_6$ → *E* post-TS complex + MeOH	-13.0	-13.9	-8.4	-5.5	-4.9	-5.8	2.6
	E-enone + $Al_2(OMe)_6$ → *Z* post-TS complex + MeOH	-13.7	-14.3	-7.0	-7.3	-5.8	-6.4	0.6

6. References

(1) K. Takeda, M. Toyota, *Tetrahedron Lett.* **2011**, *52*, 5872–5875.

(2) Y. Chen, M. Leonardi, P. Dingwall, R. Labes, P. Pasau, D. C. Blakemore, S. V. Ley, *J. Org. Chem.* **2018**, *83*, 15558–15568.

(3) X.-H. Li, S. -L. Wan, D. Chen, Q. R. Liu, C. -H. Ding,P. Fang, X. -L. Hou, *Synthesis* **2016**, *48*, 1568–1572.

(4) Y. Takaya, T. Senda, H. Kurushima, M. Ogasawara, T. Hayashi, *Tetrahedron: Asymm.* **1999**, *10*, 4047–4056.

(5) H. Grugel, F. Albrecht, T. Minuth, M. M. K. Boysen, *Org. Lett.* **2012**, *14*, 3780–3783.

(6) W. M. Akhtar, R. J. Armstrong, J. R. Frost, N. G. Stevenson, T. J. Donohoe, *J. Am. Chem. Soc.* **2018**, *140*, 11916–11920.

(7) N. T. Reynolds, T. Rovis, *Tetrahedron* **2005**, *61*, 6368–6378.

(8) L. B. Smith, R. J. Armstrong, D. Matheau-Raven, T. J. Donohoe, *J. Am. Chem. Soc.* **2020**, *142*, 2514–2523.

(9) R. J. Armstrong, W. M. Akhtar, T. A. Young, F. Duarte, T. J. Donohoe, *Angew. Chem. Int. Ed.* **2019**, *58*, 12558–12562.

(10) I. R. Hazelden, R. C. Carmona, T. Langer, P. G. Pringle, J. F. Bower, *Angew. Chem. Int. Ed.* **2018**, *57*, 5124–5128.

(11) C. Walter, R. Fröhlich, M. Oestreich, *Tetrahedron*, **2009**, *65*, 5513-5520.

(12) Y. Wang, P. Li, X. Liang, J. Ye, *Adv. Synth. Catal.* **2008**, *350*, 1383–1389.

(13) A. Díaz-Rodríguez, J. Iglesias-Fernández, C. Rovira, V. Gotor-Fernández, *ChemCatChem* **2014**, *6*, 977–980.

(14) Q. Wen, L. Zhang, J. Xiong, Q. Zeng, *Eur. J. Org. Chem.* **2016**, 32, 5360–5364.

(15) T. Hayashi, M. Kawatsura, Y. Uozumi, *Chem. Commun.* **1997**, *6*, 561–562.

(16) M. Cases, F. G. -L. de Turiso, M. S. Hadjisoteriou, G. Pattenden, *Org. Biomol. Chem.* **2005**, *3*, 2786–2804.

(17) O. Temme, S. -A. Taj, P. G. Andersson, *J. Org. Chem.* **1998**, *63*, 6007–6015.

(18) S. Rashid, B. A. Bhat, G. Mehta, *Org. Lett.* **2015**, *17*, 3604–3607.

(19) G. Kummerlöwe, B. Crone, M. Kretschmer, S. F. Kirsch, B. Luy, *Angew. Chem. Int. Ed.* **2011**, *50*, 2643–2645.

(20) E. E. Kwan, J. R. Scheerer, D. A. Evans, *J. Org. Chem.* **2013**, *78*, 175–203.

(21) R. Kim, S. Lee, J. Lee, H. -Y. Lee, *Eur. J. Org. Chem.* **2020**, *31*, 4931–4936.

(22) R. J. Armstrong, W. M. Akhtar, J. R. Frost, K. E. Christensen, N. G. Stevenson, T. J. Donohoe, *Tetrahedron* **2019**, *75*, 130680.

(23) S. Touqeer, L. Ielo, M. Miele, E. Urban, W. Holzer, V. Pace, *Org. Biomol. Chem.* **2021**, *19*, 2425–2429.

(24) P. Tang, C. Zhang, E. Chen, B. Chen, W. Chen, Y. Yu, *Tetrahedron Lett.* **2017**, *58*, 2157–2161.

(25) X. Chen, X. Li, X. -L Chen, L. -B. Qu, K. -Y. Chen, K. Sun, Z. -D. Liu, W. -Z. Bi, Y. -Y. Xia, H. -T. Wu, Y. -F. Zhao, *Chem. Commun.* **2015**, *51*, 3846–3849.

(26) Y. Zhou, C. Rao, S. Mai, Q. Song, *J. Org. Chem.* **2016**, *81*, 2027–2034.

(27) M. Zhou, Y. Zhou, Q. Song, *Chem. Eur. J.* **2015**, *21*, 10654–10659.

(28) W. Liang, Z. Zhang, D. Yi, Q. Fu, S. Chen, L. Yang, F. Du,J. Ji, W. Wei, *Chinese J. Chem.* **2017**, *35*, 1378–1382.

(29) B. S. Lauber, L. A. Hardegger, A. K. Asraful, B. A. Lund, O. Dumele, M. Harder, B. Kuhn, R. A. Engh, F. Diederich, *Chem. Eur. J.* **2016**, *22*, 211–221.

(30) P. W. Tan, M. Haughey, D. J. Dixon, *Chem. Commun.* **2015**, *51*, 4406–4409.

(31) I. Sivák, J. Václav, D. Berkeš, A. Kolarovič, *Tetrahedron* **2015**, *71*, 8876–8884.

(32) L. Xie, R. Yuan, R. Wang, Z. Peng, J. Xiang, W. He, *Eur. J. Org. Chem.* **2014**, 2668–2671.

(33) A. M. Whittaker, V. M. Dong, *Angew. Chem. Int. Ed.* **2015**, *54*, 1312–1315.

(34) A. Sharma, S. Gamre, S. Chattopadhyay, *Tetrahedron: Asymm.* **2009**, *20*, 1164–1167.

(35) A. Bugarin, K. D. Jones, B. T. Connell, *Chem. Commun.* **2010**, *46*, 1715–1717.

(36) C. D. Aretz, H. Escobedo, B. J. Cowen, *Eur. J. Org. Chem.* **2018**, 1880–1884.

(37) Y. -S. Hon, F. -J. Chang, L. Lu, W. -C. Lin, *Tetrahedron* **1998**, *54*, 5233–5246.

(38) R. E. Ruscoe, N. J. Fazakerley, H. Huang, S. Flitsch, D. J. Procter, *Chem. Eur. J.* **2016**, *22*, 116–119.

(38) P. -Y. Ji, Y. -F. Liu, J. -W. Xu, W. -P. Luo, Q. Liu, C. -C Guo, *J. Org. Chem.* **2017**, *82*, 2965–2971.

(40) S. Poplata, T. Bach, *J. Am. Chem. Soc.* **2018**, *140*, 3228–3231.

(41) P. Duhamel, A. Deyine, G. Dujardin, G. Plé, J. -M. Poirier, *J. Chem. Soc., Perkin Trans. 1* **1995**, *17*, 2103

(42) J. A. C. Romero, S. A. Tabacco, K. A. Woerpel, *J. Am. Chem. Soc.* **2000**, *122*, 168–169.

(43) S. Tanaka, T. Kunisawa, Y. Yoshii, T. Hattori, *Org. Lett.* **2019**, *21*, 8509–8513.

(44) F. Neese, *Wiley Interdiscip. Rev. Comput. Mol. Sci.* **2011**, *2*, 73-78.

(45) (a) D. G. Liakos, M. Sparta, M. K. Kesharwani, J. M. L. Martin, F. Neese, *J. Chem. Theory Comput.* **2015**, *11*, 1525–1539. (b) V. Barone, M. Cossi, *J. Phys. Chem. A* **1998**, *102*, 1995-2001. (c) F. Weigend, R. Ahlrichs, *Phys. Chem. Chem. Phys.* ***2005**, 7*, 3297-3305. (d) C. Adamo, V. Barone, *J. Chem. Phys.* ***1999**, 110*, 6158-6170. (e) S. Grimme, J. Antony, S. Ehrlich, H. Krieg, *J. Chem. Phys.* ***2010**, 132*, 154104. (f) S. Grimme, S. Ehrlich, L. Goerigk, *J. Comput. Chem.* ***2011**, 32*, 1456-1465.

(46) F. Neese, F. Wennmohs, A. Hansen, U. Becker, *Chem. Phys.* **2009**, *356*, 98-109.

(47) D. M. York, M. Karplus, *Chem. Phys.* **1999**, *103*, 11060-11079.

(48) F. L. Hirshfeld, *Theor. Chim. Acta* **1977**, *44*, 129-138.

(49) S. Grimme, *J. Chem. Theory Comput.* **2019**, *15*, 2847–2862.

(50) (a) A. K. Rappe, C. J. Casewit, K. S. Colwell, W. A. Goddard III, W. M. Skiff, *J. Am. Chem. Soc.* **1992**, *114*, 10024–10035. (b) N. M. O'Boyle, M. Banck, C. A. James, C. Morley, T. Vandermeersch, G. R. Hutchison, *J. Cheminform.* **2011**, *3* (1), 33.

(51) T. Young, duartegroup/otherm: Major symmetry improvements. *Zenodo* **2020**.

(52) S. Grimme, *Chem. Eur. J.* ***2012**, 18*, 9955-9964.

(53) (a) S. Xu, E. A. Carter, *Proc. Natl. Acad. Sci. USA* ***2019**, 116*, 22953-22958. (b) D. Conic, K. Pierloot, T. N. Parac-Vogt, J. N. Harvey, *Phys. Chem. Chem. Phys*. **2020**, *22*, 25136-25145.

7. NMR Spectra

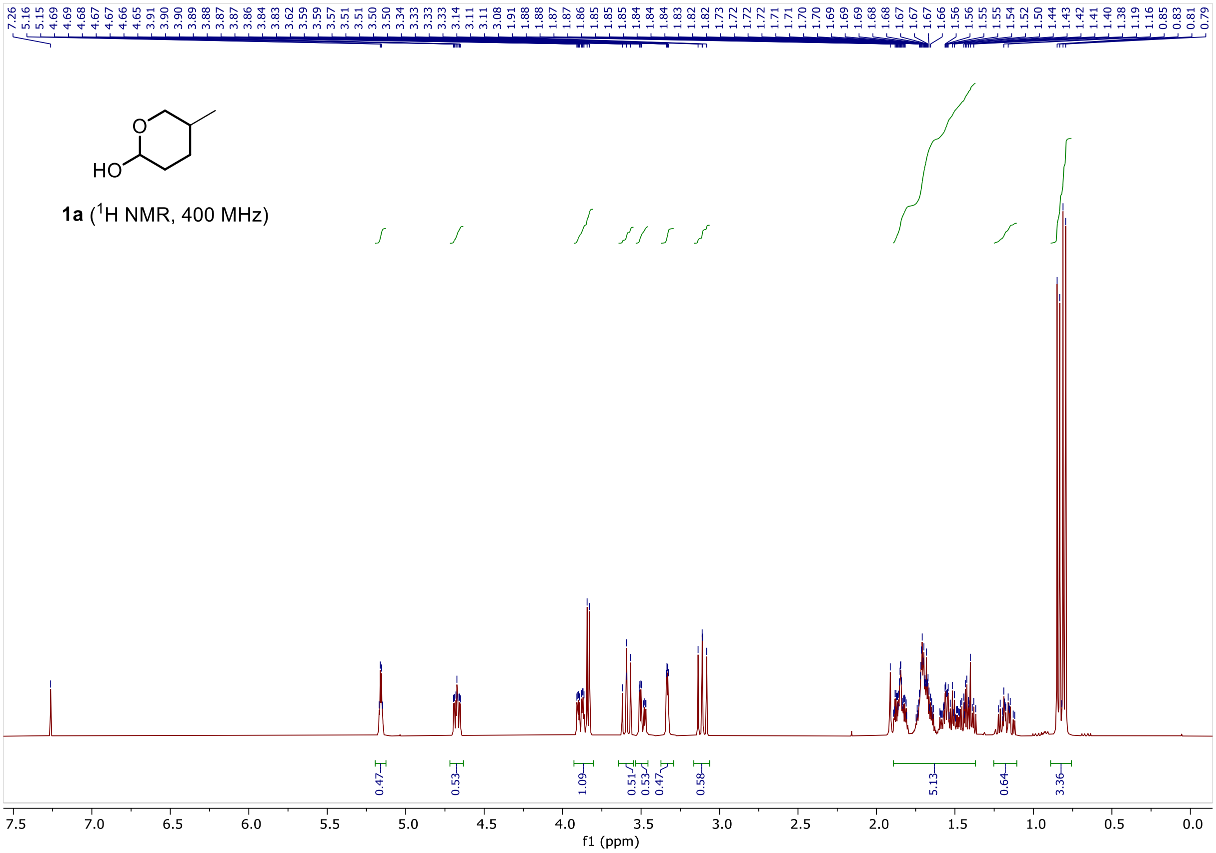

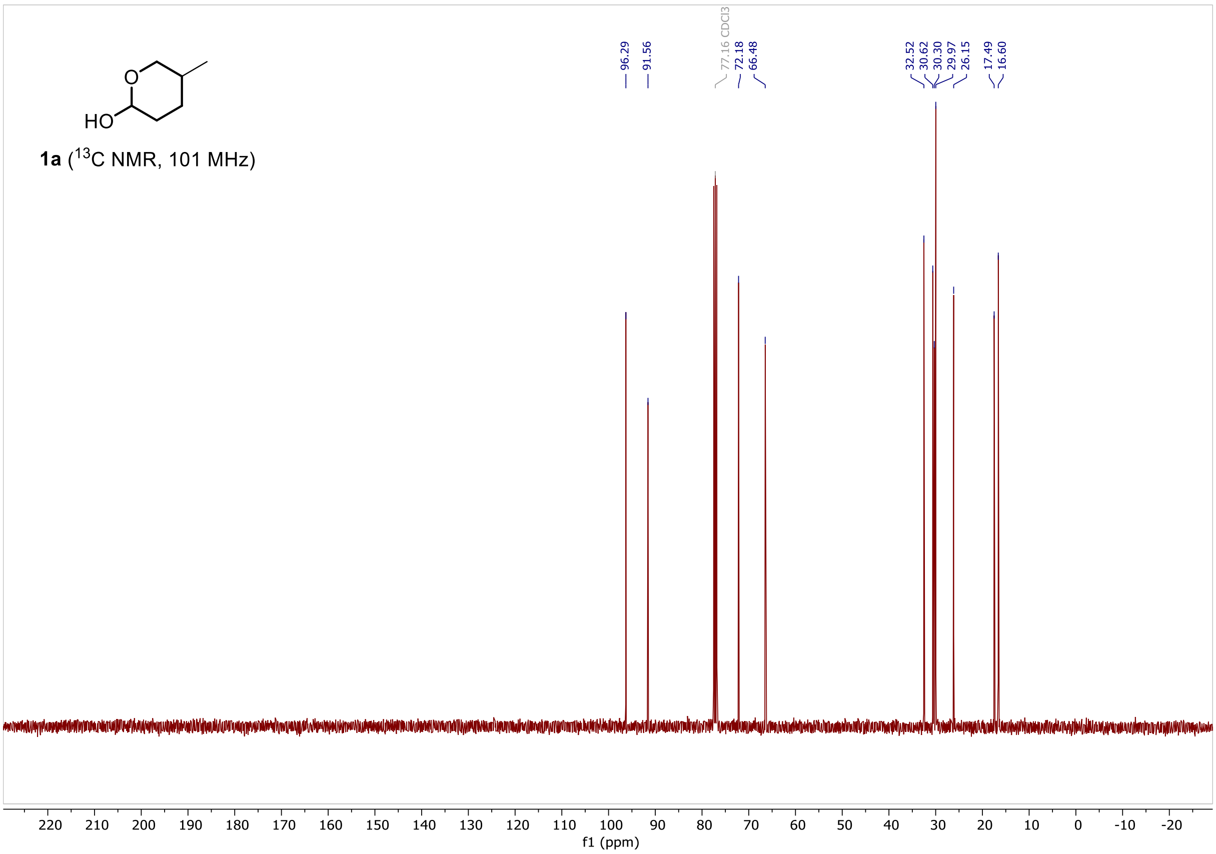

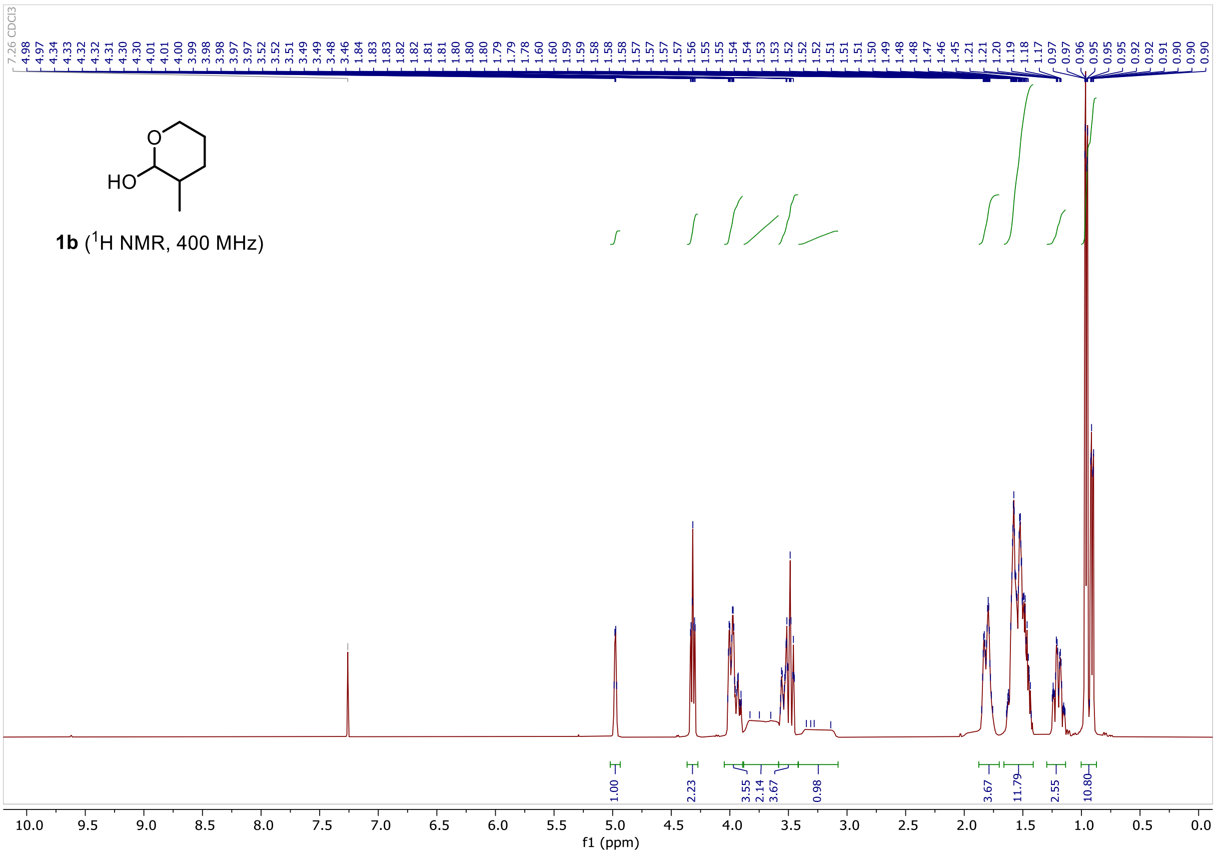

1b (^{1}H NMR, 400 MHz)

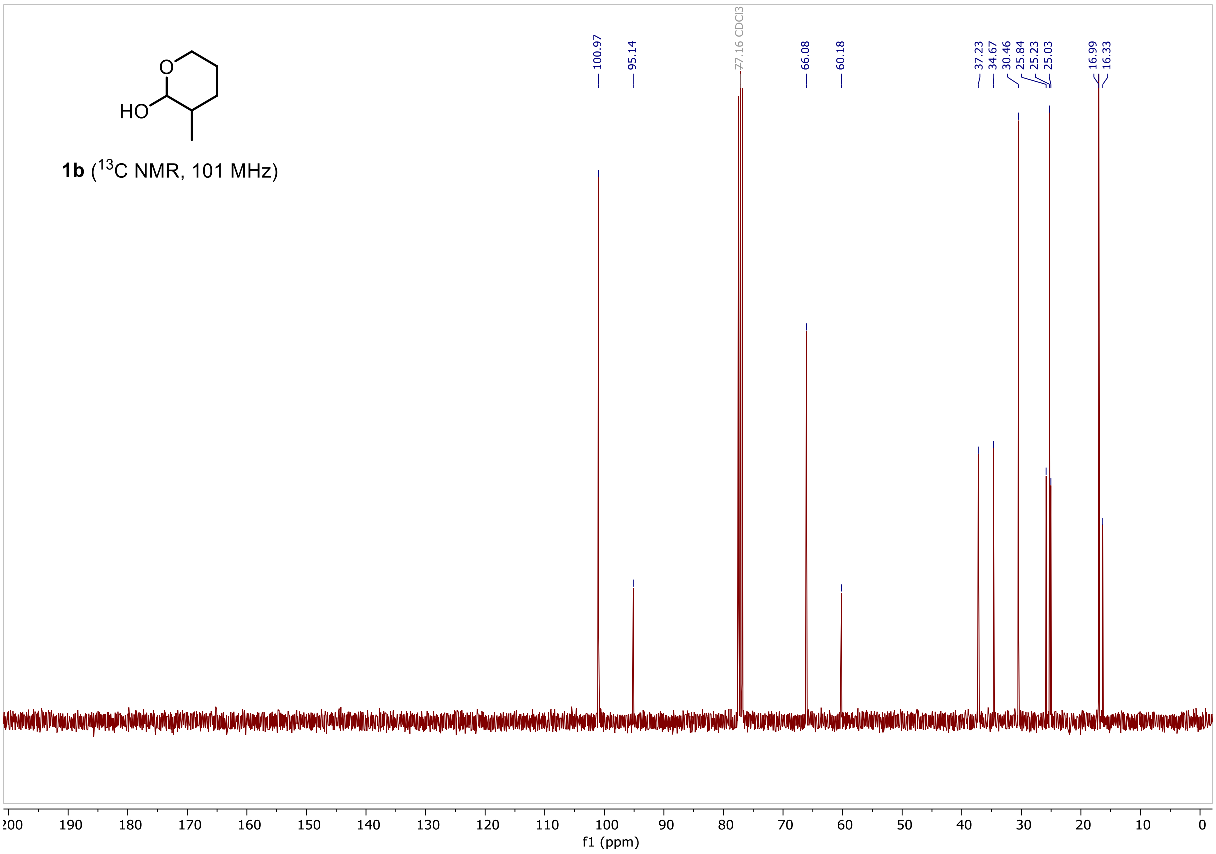

1b (^{13}C NMR, 101 MHz)

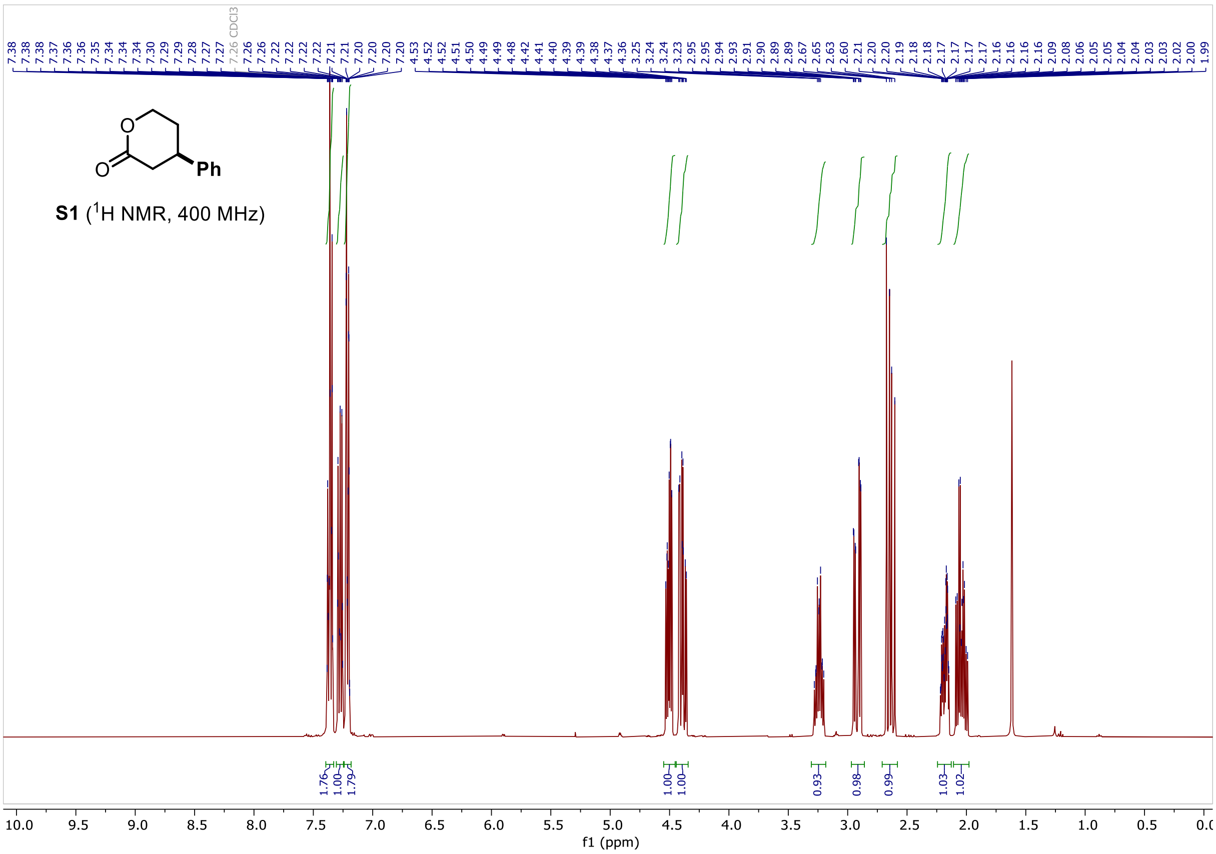

S1 (¹H NMR, 400 MHz)
f1 (ppm)

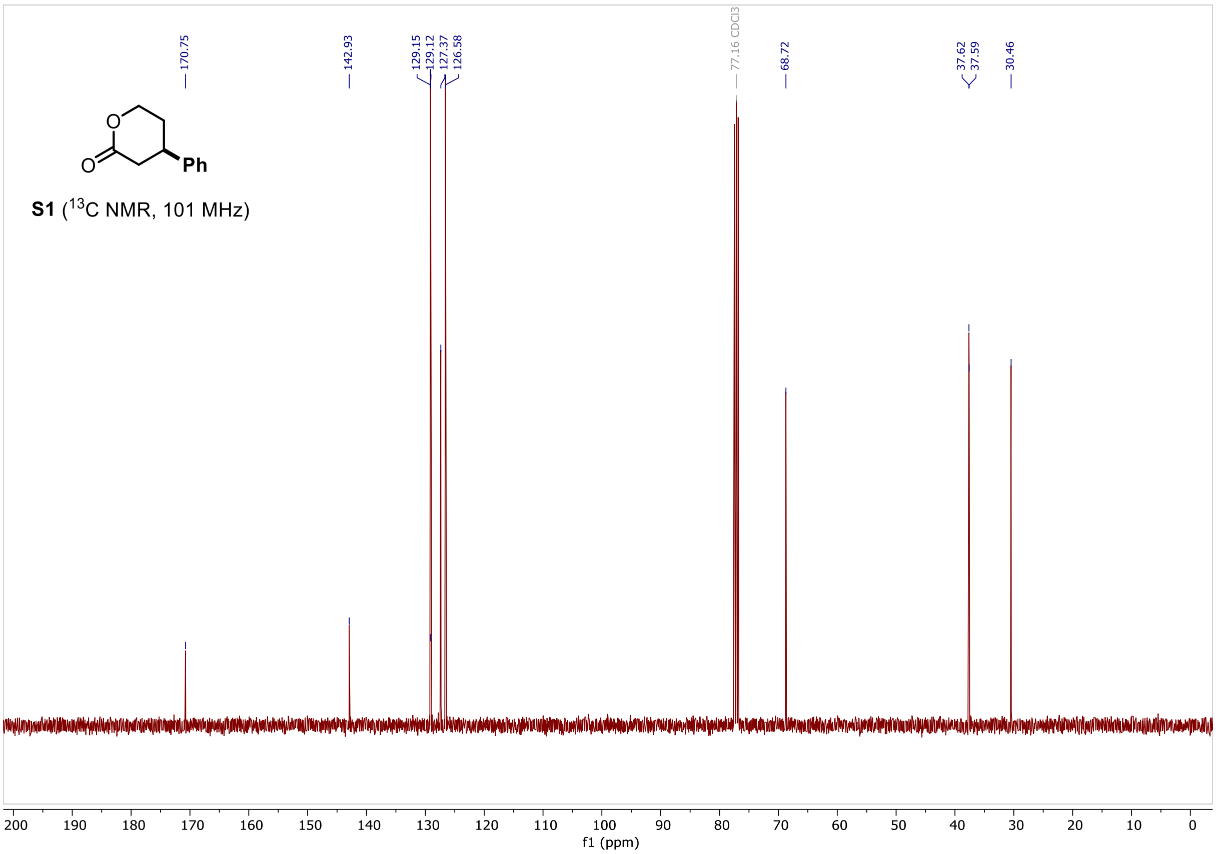

S1 (¹³C NMR, 101 MHz)
170.75
142.93
129.15
129.12
127.37
126.58
77.16 CDCl3
68.72
37.62
37.59
30.46
f1 (ppm)

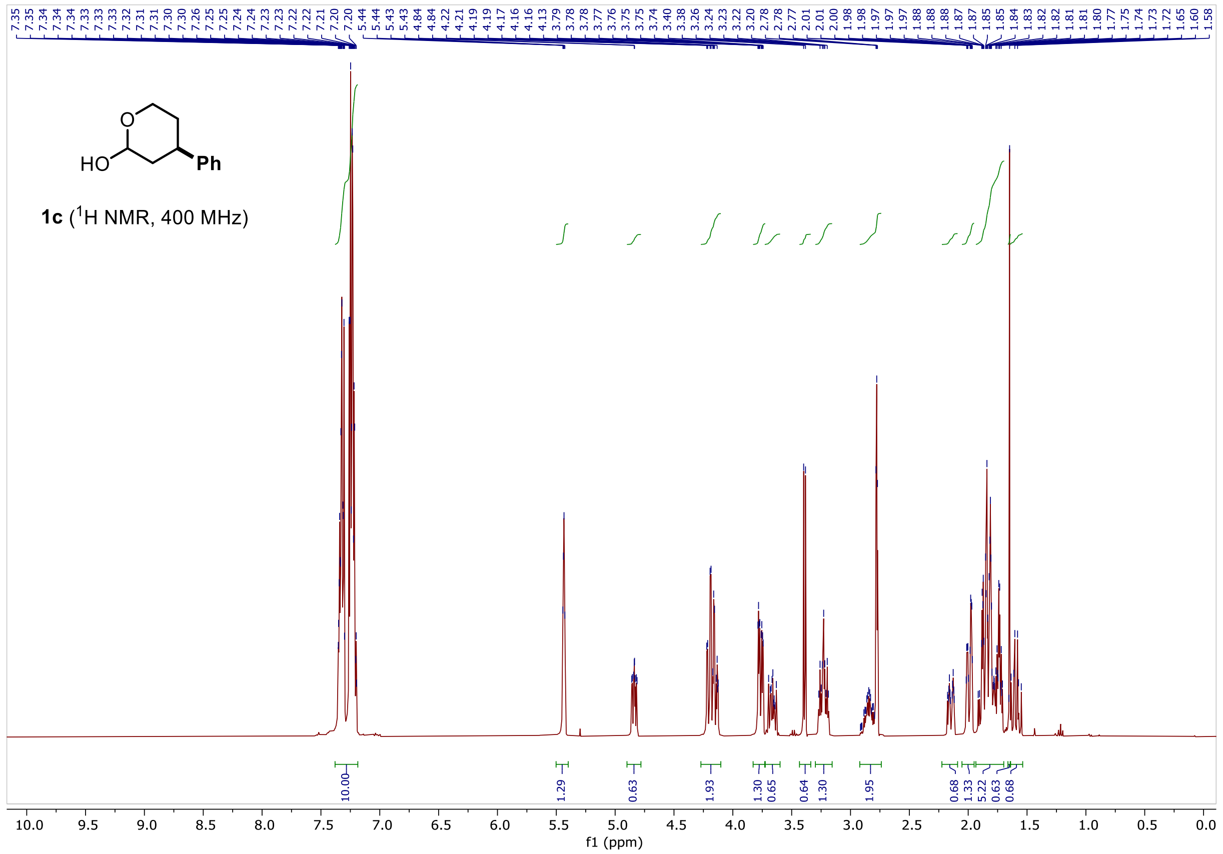
HO
O
Ph
1c (¹H NMR, 400 MHz)
f1 (ppm)

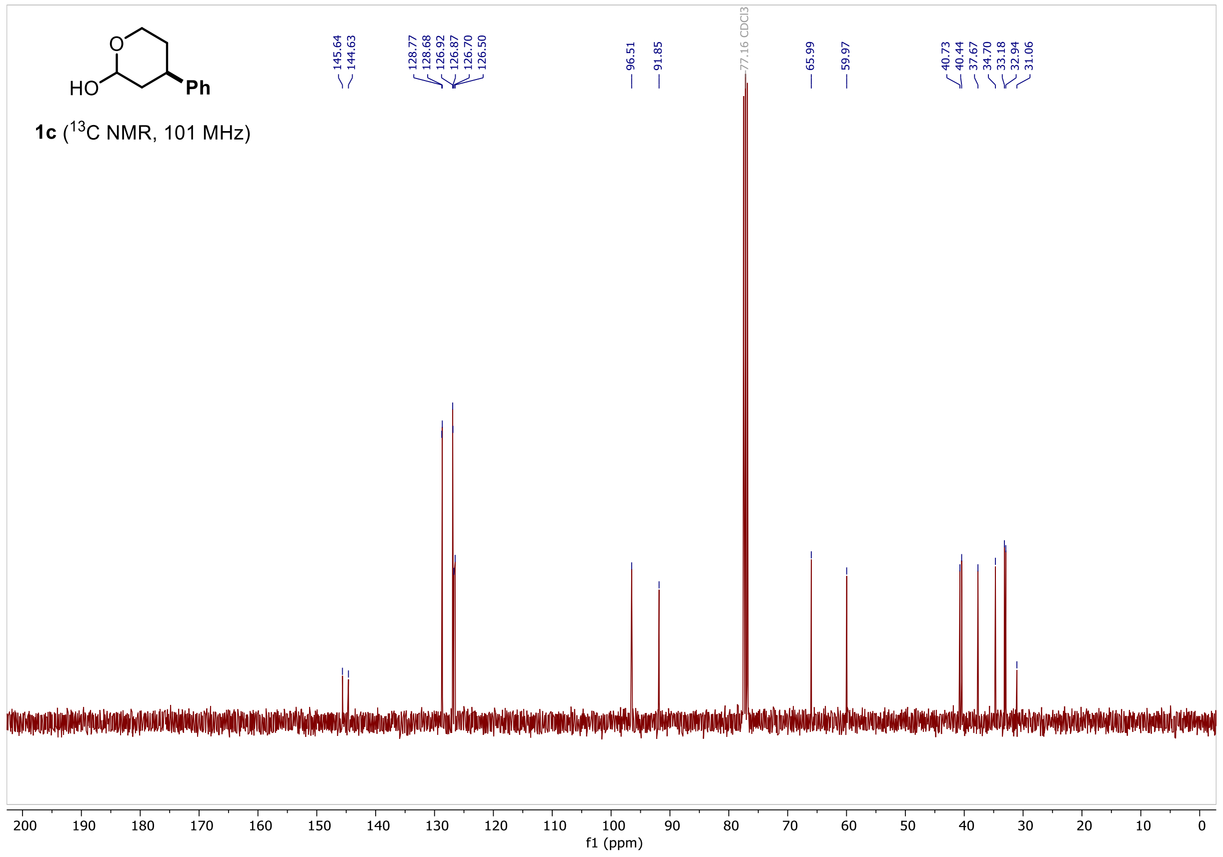
HO
O
Ph
1c (¹³C NMR, 101 MHz)
77.16 CDCl3
f1 (ppm)

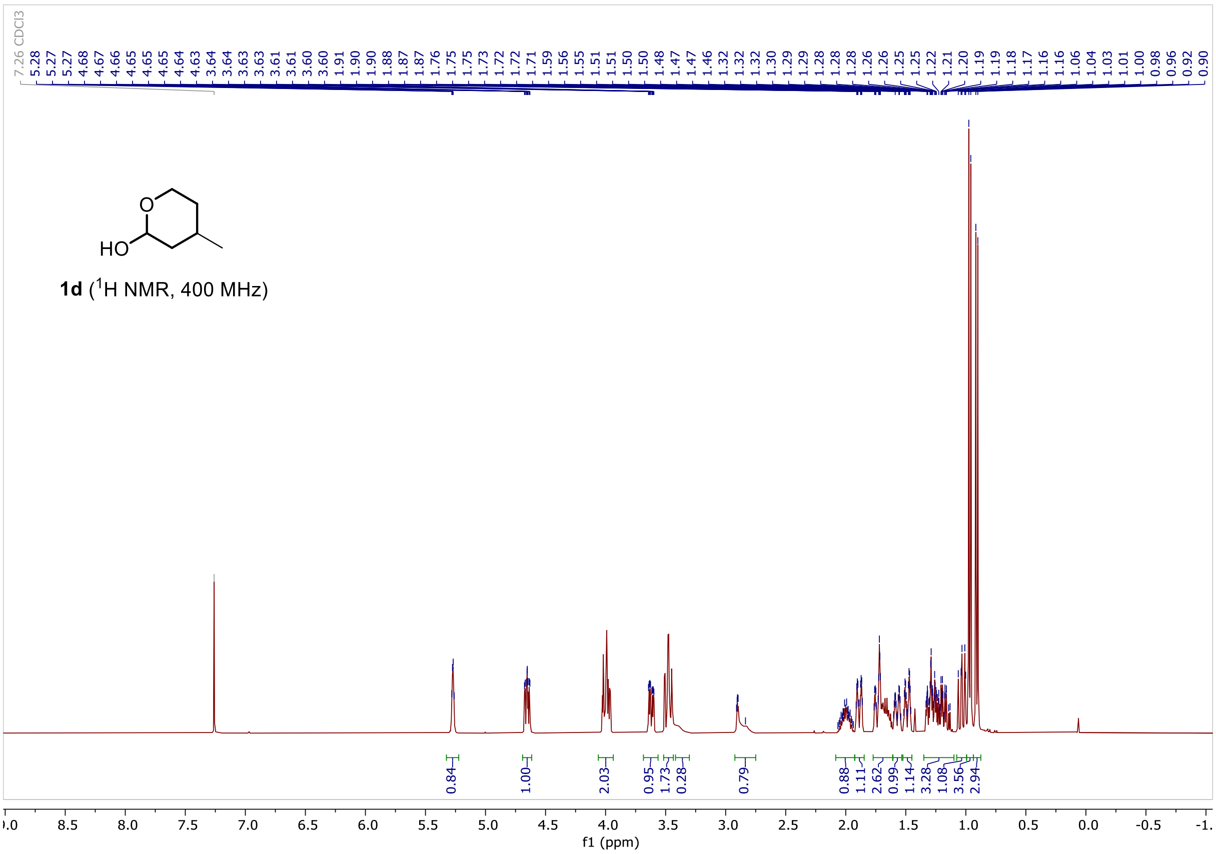

7.26 CDCl3
5.28
5.27
5.27
4.68
4.67
4.66
4.65
4.65
4.65
4.64
4.63
3.64
3.64
3.63
3.63
3.61
3.61
3.60
3.60
1.91
1.90
1.90
1.88
1.87
1.87
1.76
1.75
1.75
1.73
1.72
1.72
1.71
1.59
1.56
1.55
1.51
1.51
1.50
1.50
1.48
1.47
1.47
1.46
1.32
1.32
1.32
1.30
1.29
1.29
1.28
1.28
1.28
1.26
1.25
1.25
1.22
1.21
1.20
1.19
1.19
1.18
1.17
1.16
1.16
1.06
1.04
1.03
1.01
1.00
0.98
0.96
0.92
0.90
HO
O
1d (1H NMR, 400 MHz)
0.84
1.00
2.03
0.95
1.73
0.28
0.79
0.88
1.11
2.62
0.99
1.14
3.28
1.08
3.56
2.94
f1 (ppm)

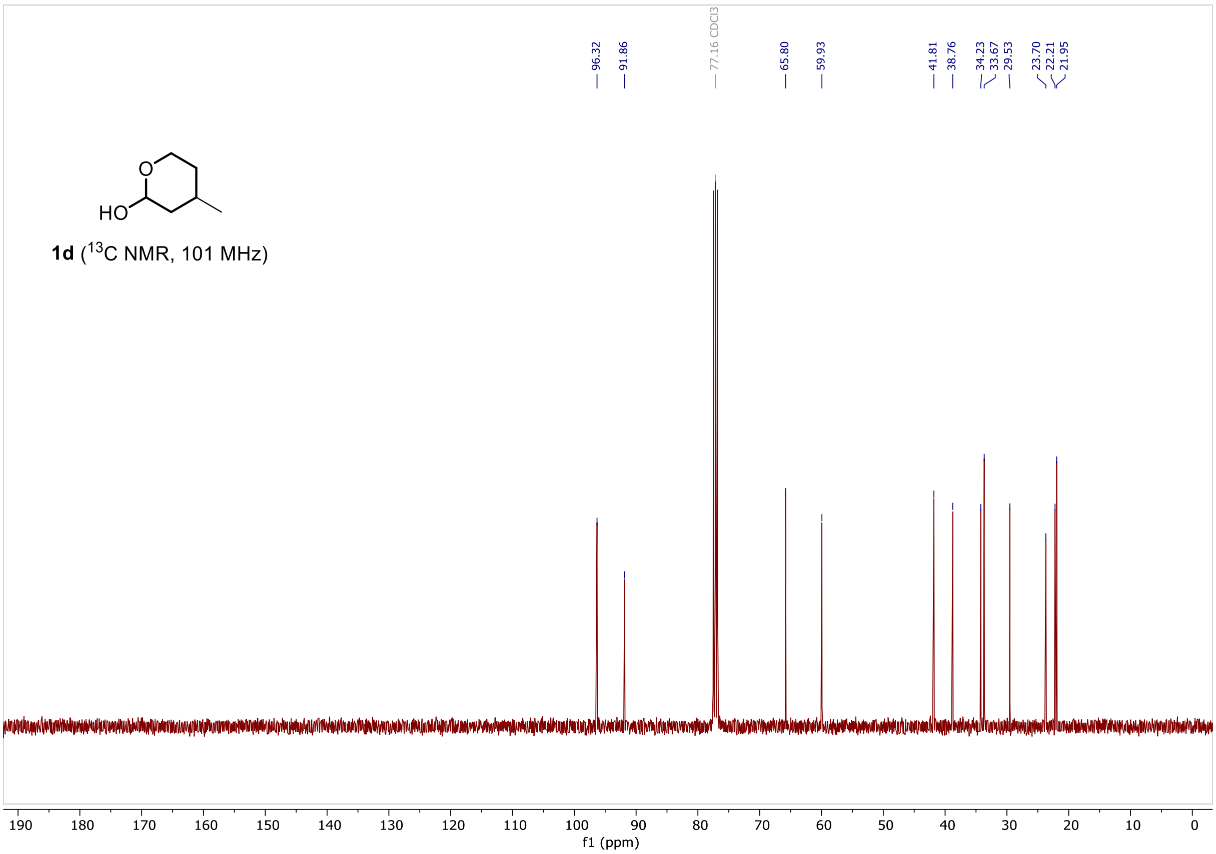

96.32
91.86
77.16 CDCl3
65.80
59.93
41.81
38.76
34.23
33.67
29.53
23.70
22.21
21.95
HO
O
1d (13C NMR, 101 MHz)
f1 (ppm)

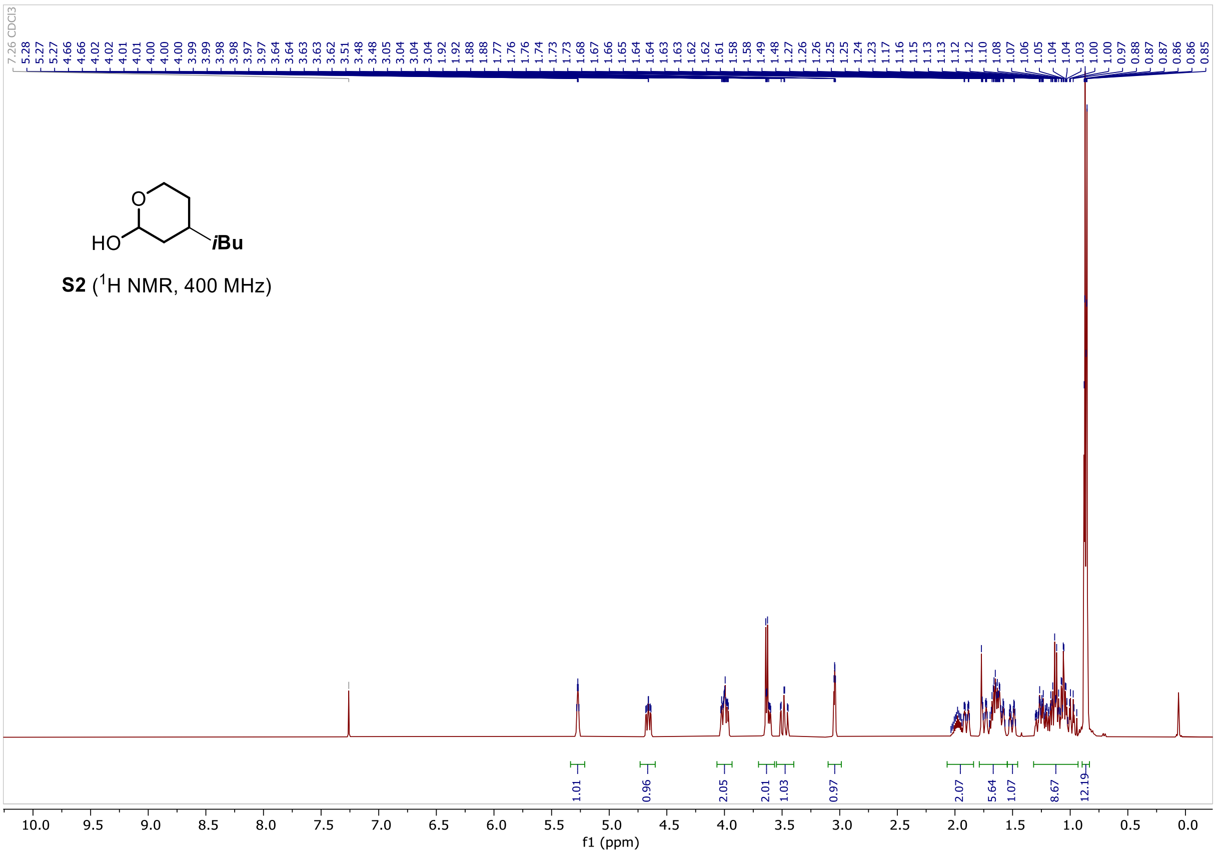
HO
O
iBu
S2 (^{1}H NMR, 400 MHz)
f1 (ppm)

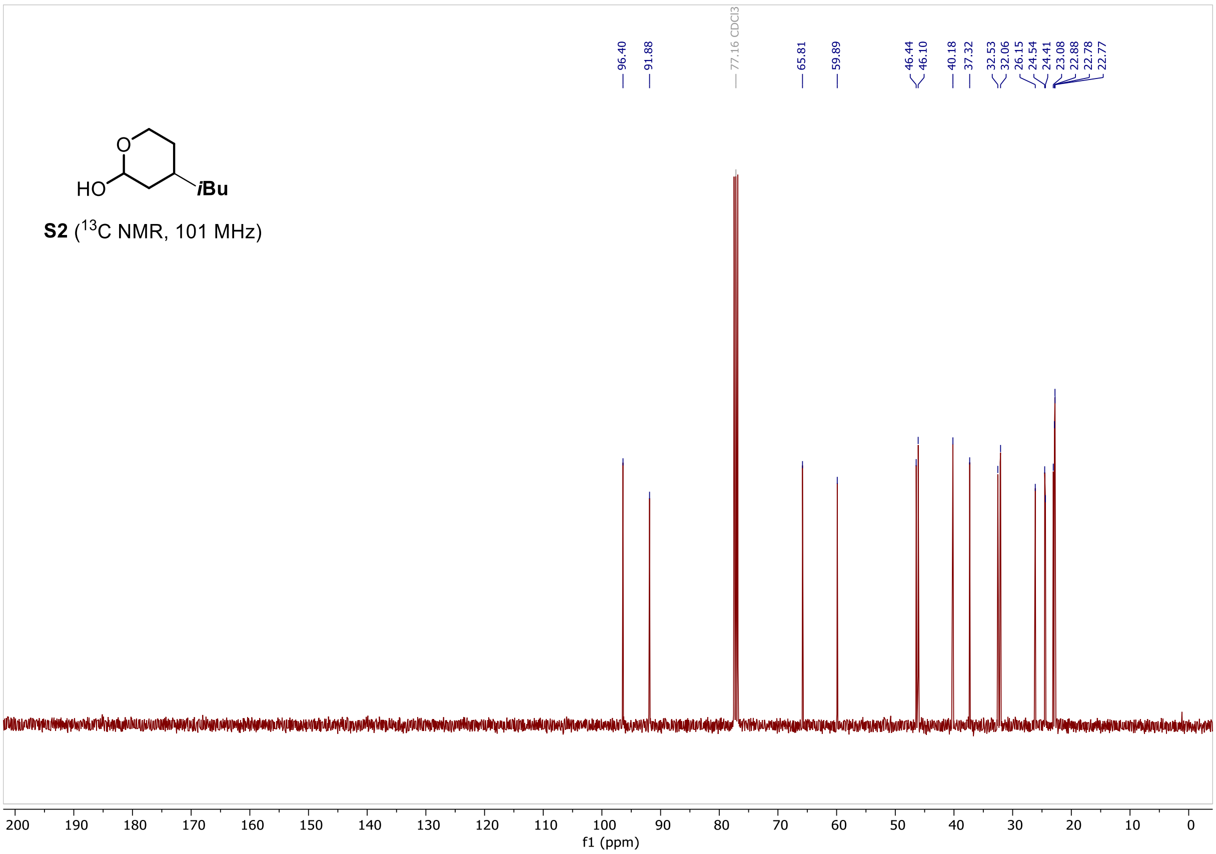
HO
O
iBu
S2 (^{13}C NMR, 101 MHz)
96.40
91.88
77.16 CDCl3
65.81
59.89
46.44
46.10
40.18
37.32
32.53
32.06
26.15
24.54
24.41
23.08
22.88
22.78
22.77
f1 (ppm)

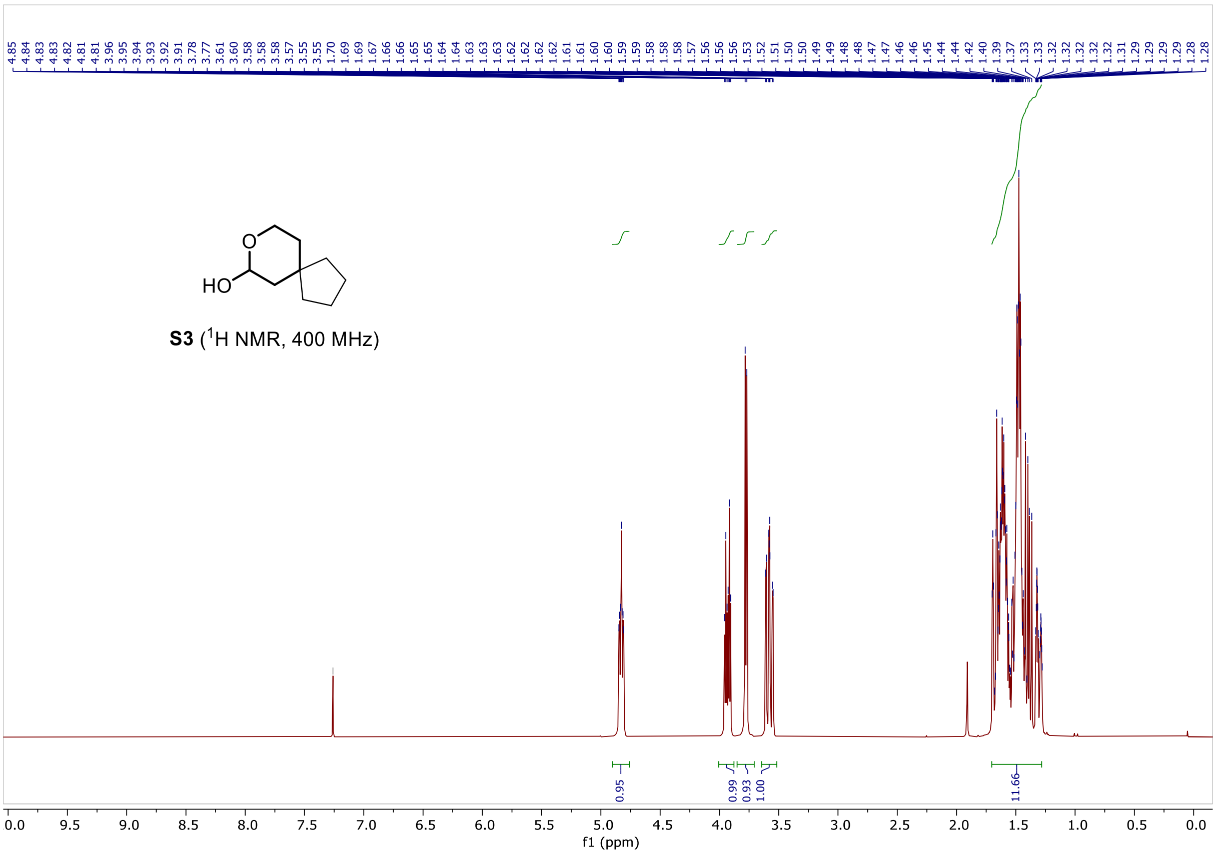
S3 (1H NMR, 400 MHz)
HO
O
0.95
0.99
0.93
1.00
11.66
f1 (ppm)

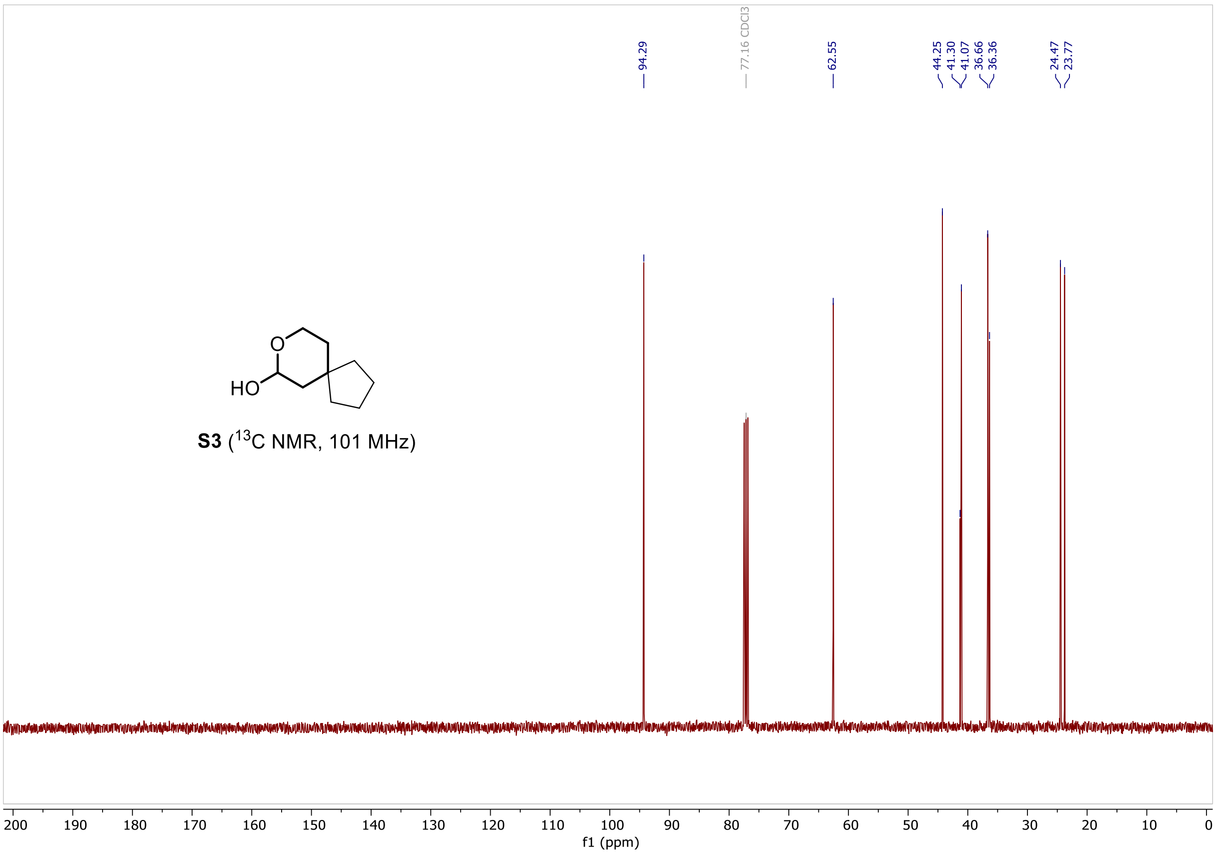
S3 (13C NMR, 101 MHz)
HO
O
94.29
77.16 CDCl3
62.55
44.25
41.30
41.07
36.66
36.36
24.47
23.77
f1 (ppm)

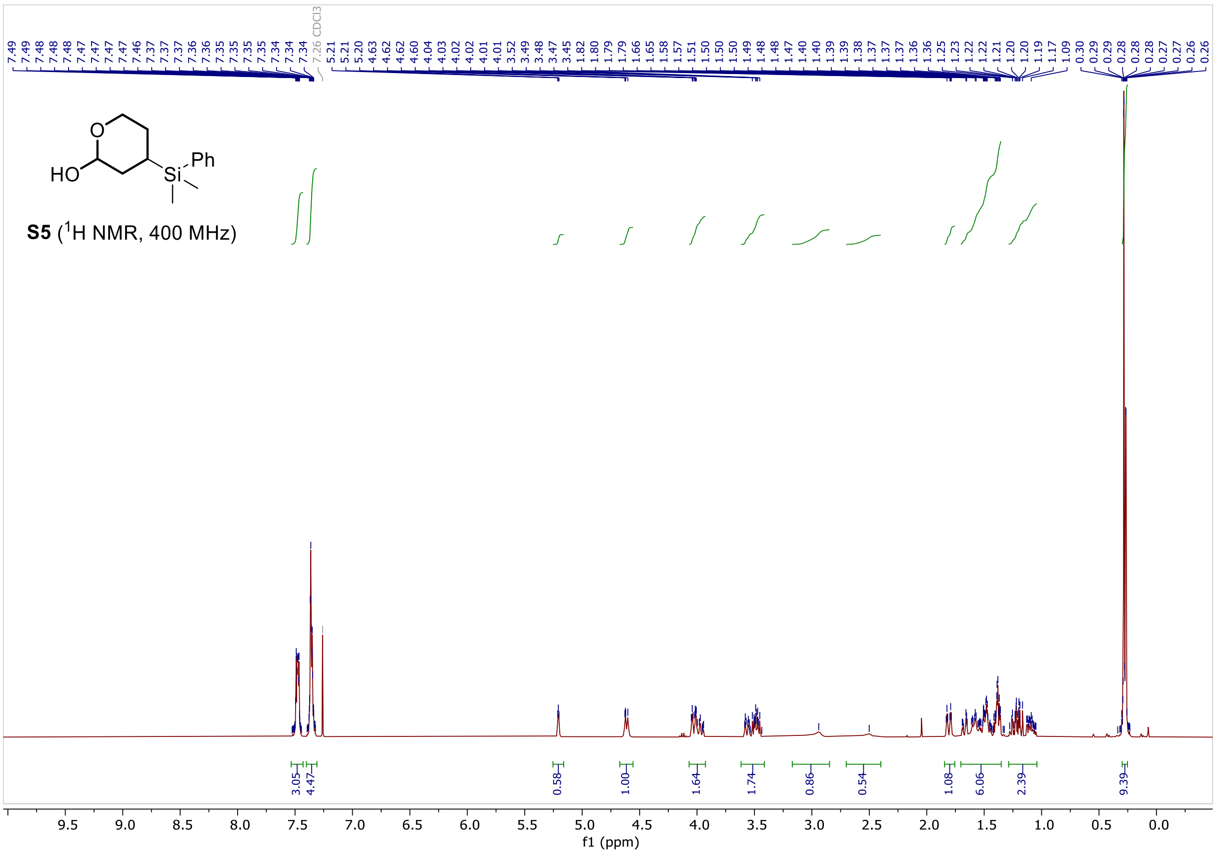
7.49 7.49 7.48 7.48 7.47 7.47 7.47 7.46 7.37 7.37 7.37 7.36 7.36 7.35 7.35 7.35 7.34 7.34 7.26 CDCl3 5.21 5.21 5.20 4.63 4.62 4.62 4.60 4.04 4.03 4.02 4.02 4.01 4.01 3.52 3.49 3.48 3.47 3.45 1.82 1.80 1.79 1.79 1.66 1.65 1.58 1.57 1.51 1.50 1.50 1.50 1.49 1.48 1.47 1.40 1.39 1.39 1.38 1.37 1.37 1.37 1.36 1.25 1.23 1.22 1.22 1.21 1.20 1.20 1.19 1.17 1.09 0.30 0.29 0.29 0.28 0.28 0.28 0.27 0.27 0.26 0.26
HO
O
Si
Ph
S5 (1H NMR, 400 MHz)
3.05 4.47 0.58 1.00 1.64 1.74 0.86 0.54 1.08 6.06 2.39 9.39
9.5 9.0 8.5 8.0 7.5 7.0 6.5 6.0 5.5 5.0 4.5 4.0 3.5 3.0 2.5 2.0 1.5 1.0 0.5 0.0
f1 (ppm)

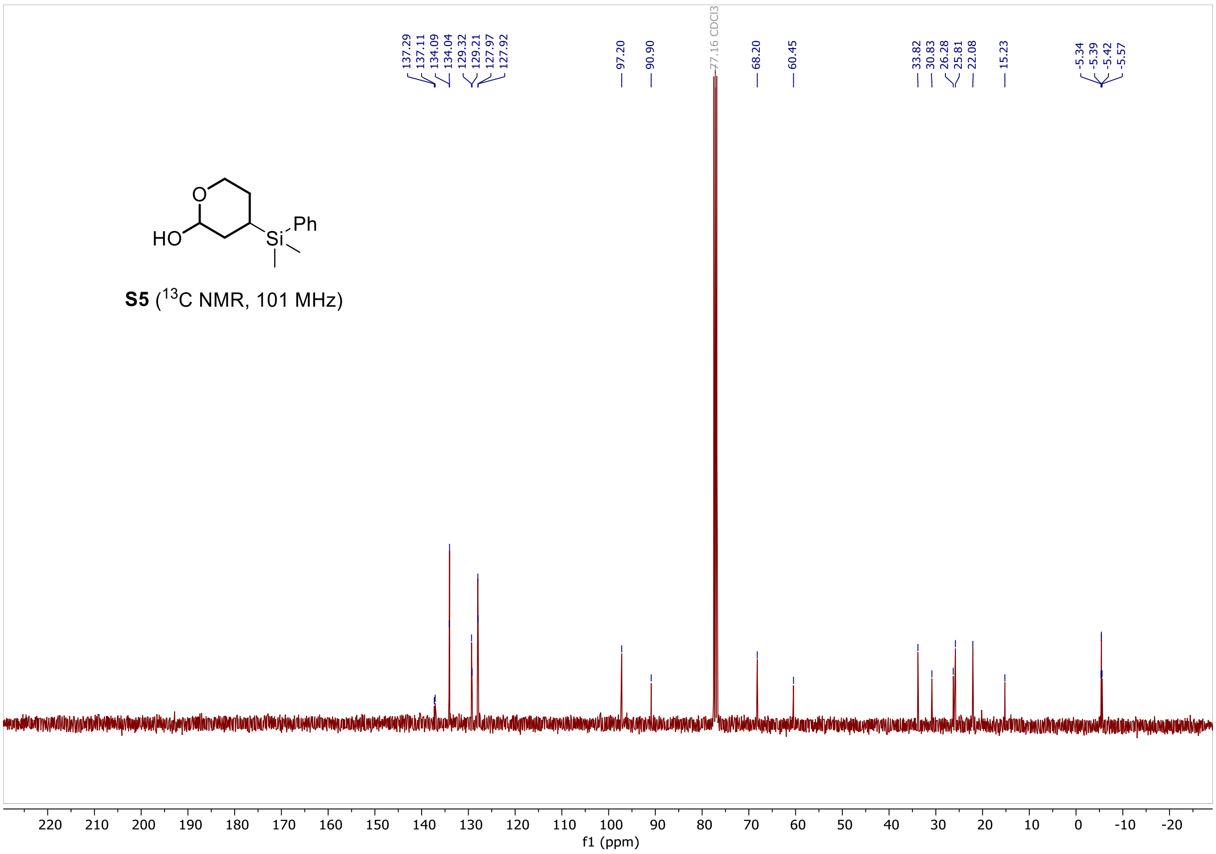
137.29 137.11 134.09 134.04 129.32 129.21 127.97 127.92
97.20
90.90
77.16 CDCl3
68.20
60.45
33.82 30.83 26.28 25.81 22.08
15.23
-5.34 -5.39 -5.42 -5.57
HO
O
Si
Ph
S5 (13C NMR, 101 MHz)
220 210 200 190 180 170 160 150 140 130 120 110 100 90 80 70 60 50 40 30 20 10 0 -10 -20
f1 (ppm)

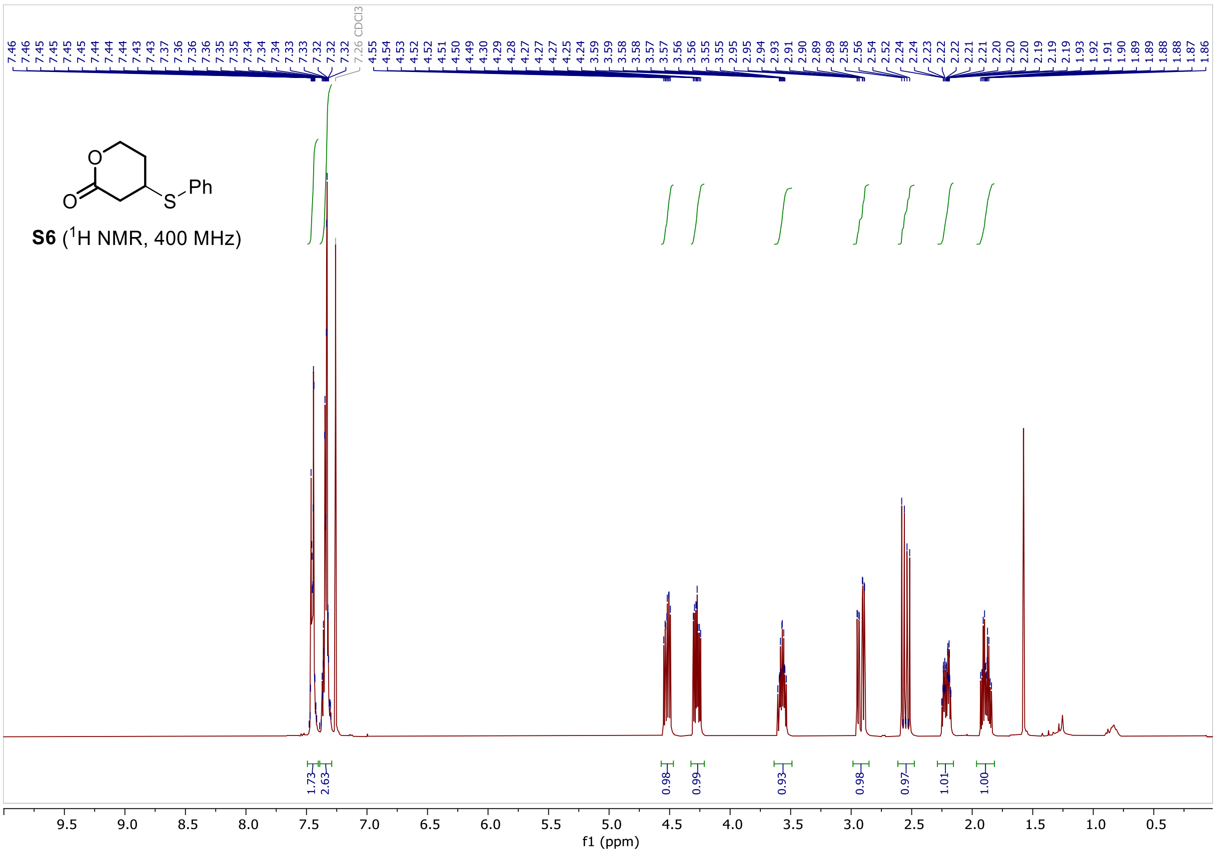
S6 (1H NMR, 400 MHz)
Ph
O
O
S
f1 (ppm)

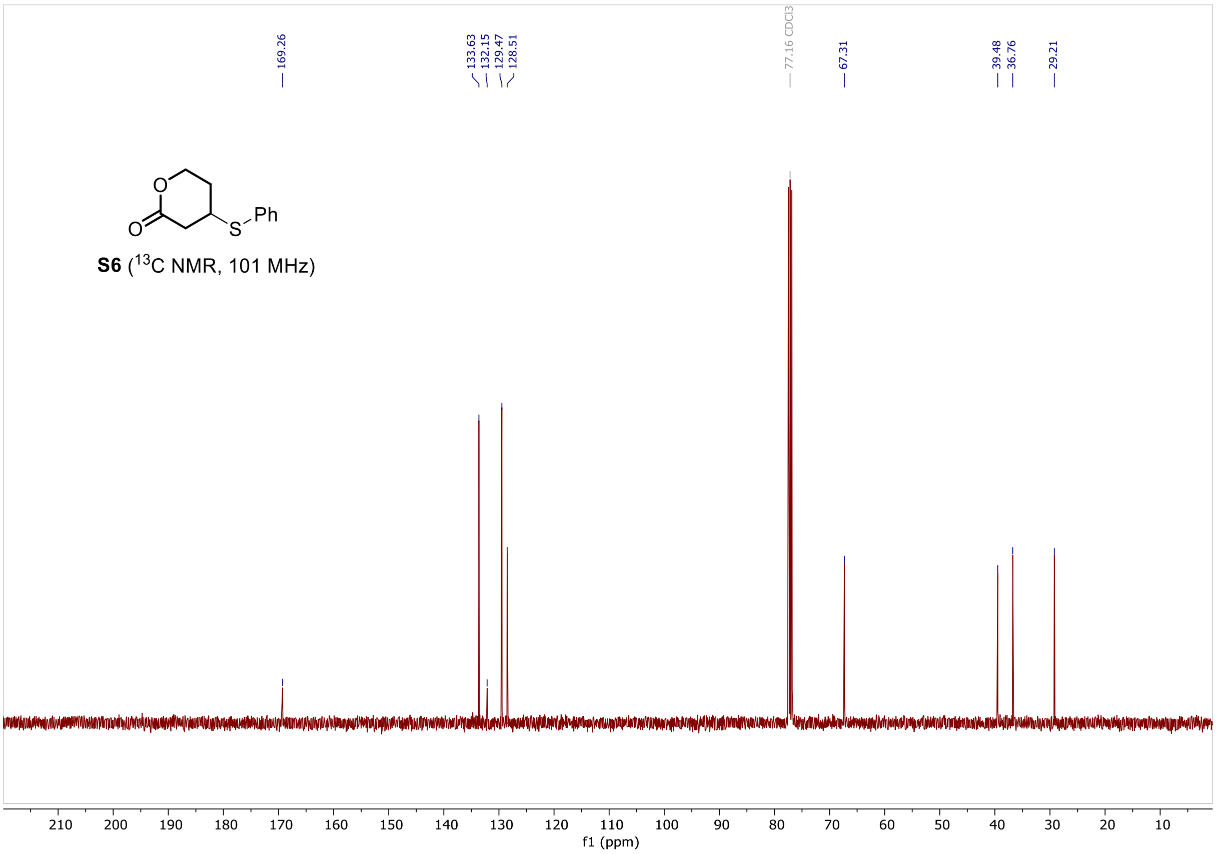
S6 (13C NMR, 101 MHz)
169.26
133.63
132.15
129.47
128.51
77.16 CDCl3
67.31
39.48
36.76
29.21
f1 (ppm)

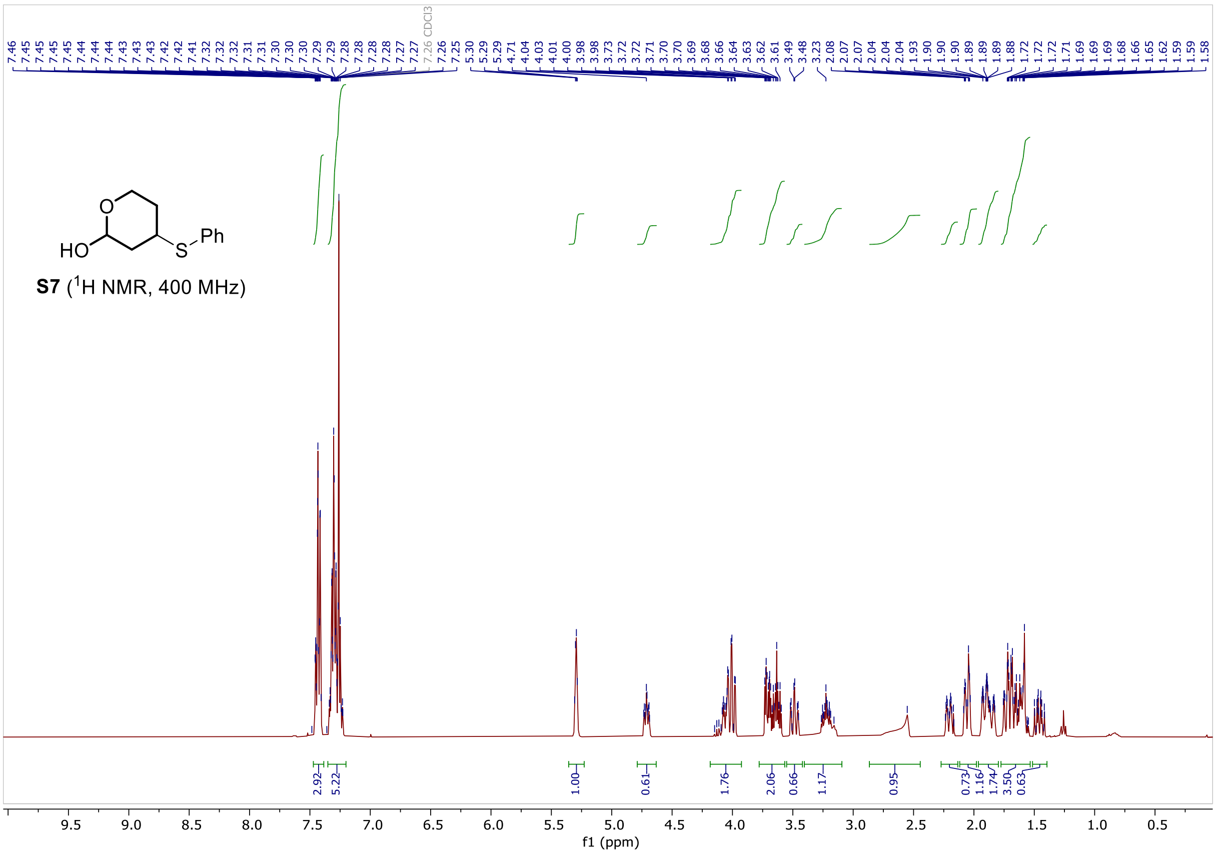

HO
O
S
Ph
S7 (^{1}H NMR, 400 MHz)
7.46 7.45 7.45 7.45 7.45 7.44 7.44 7.43 7.43 7.43 7.42 7.42 7.41 7.32 7.32 7.32 7.31 7.31 7.30 7.30 7.29 7.29 7.28 7.28 7.28 7.28 7.27 7.26 CDCl3 7.26 7.25 5.30 5.29 5.29 4.71 4.04 4.03 4.01 4.00 3.98 3.98 3.73 3.72 3.72 3.71 3.70 3.69 3.68 3.66 3.64 3.63 3.62 3.61 3.49 3.48 3.23 2.08 2.07 2.04 2.04 2.04 1.93 1.90 1.90 1.90 1.89 1.89 1.89 1.88 1.72 1.72 1.72 1.71 1.69 1.69 1.69 1.68 1.66 1.65 1.62 1.59 1.59 1.58
2.92 5.22 1.00 0.61 1.76 2.06 0.66 1.17 0.95 0.73 1.16 1.74 3.50 0.63
9.5 9.0 8.5 8.0 7.5 7.0 6.5 6.0 5.5 5.0 4.5 4.0 3.5 3.0 2.5 2.0 1.5 1.0 0.5
f1 (ppm)

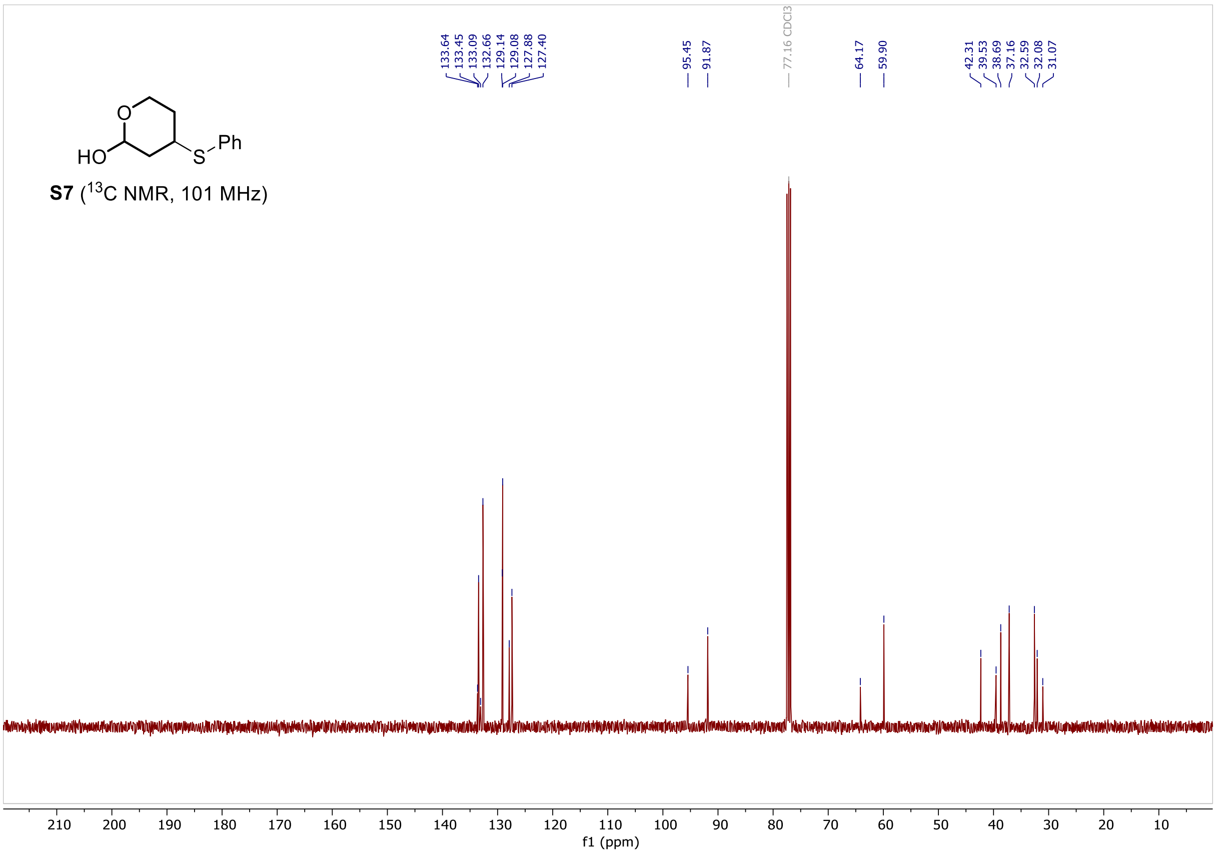

HO
O
S
Ph
S7 (^{13}C NMR, 101 MHz)
133.64 133.45 133.09 132.66 129.14 129.08 127.88 127.40
95.45 91.87
77.16 CDCl3
64.17 59.90
42.31 39.53 38.69 37.16 32.59 32.08 31.07
210 200 190 180 170 160 150 140 130 120 110 100 90 80 70 60 50 40 30 20 10
f1 (ppm)

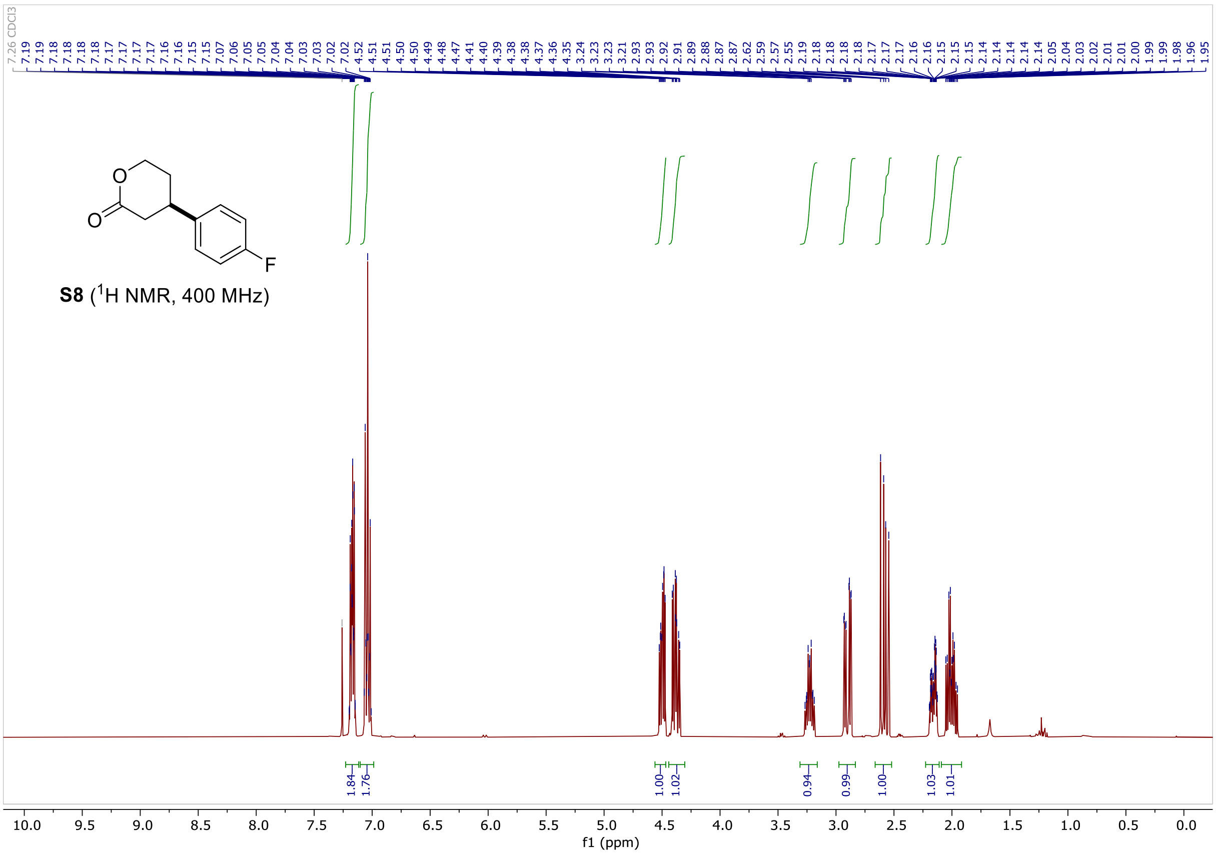

S8 (^{1}H NMR, 400 MHz)

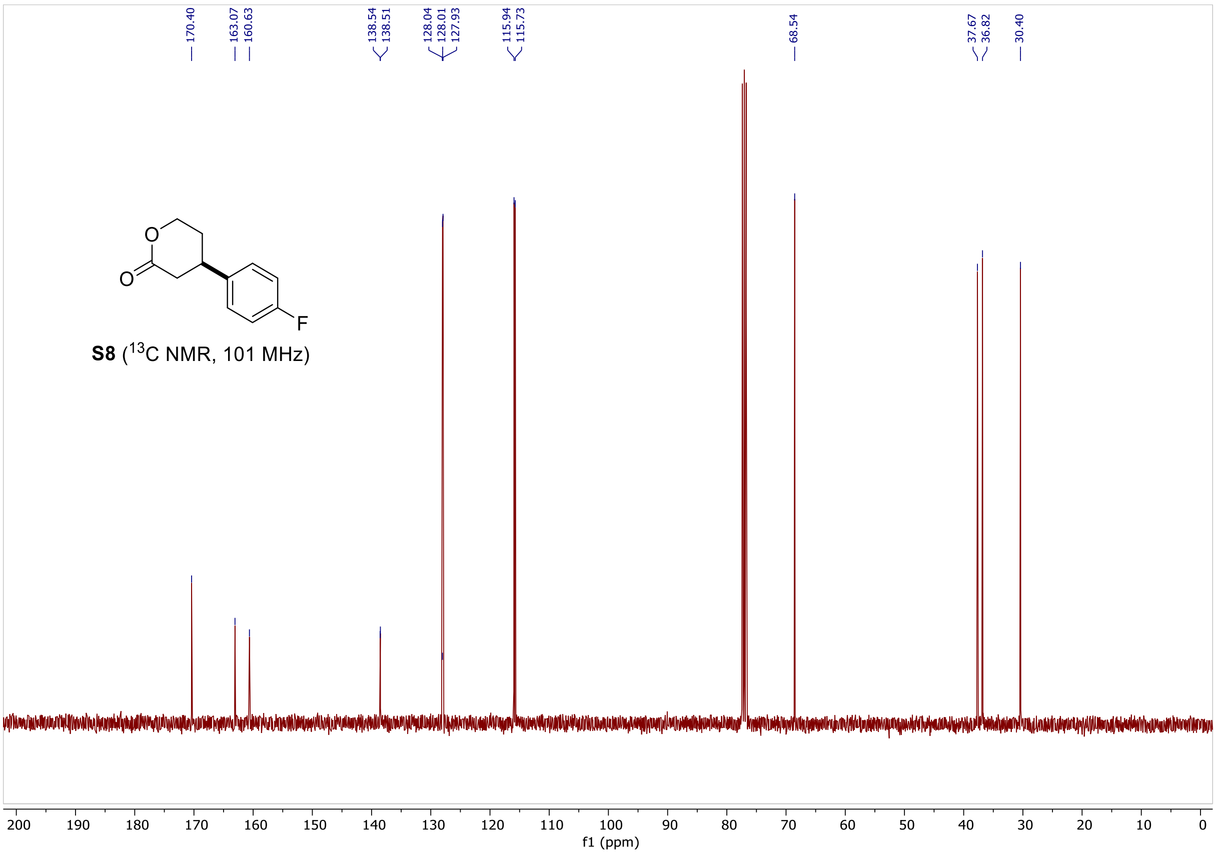

S8 (^{13}C NMR, 101 MHz)

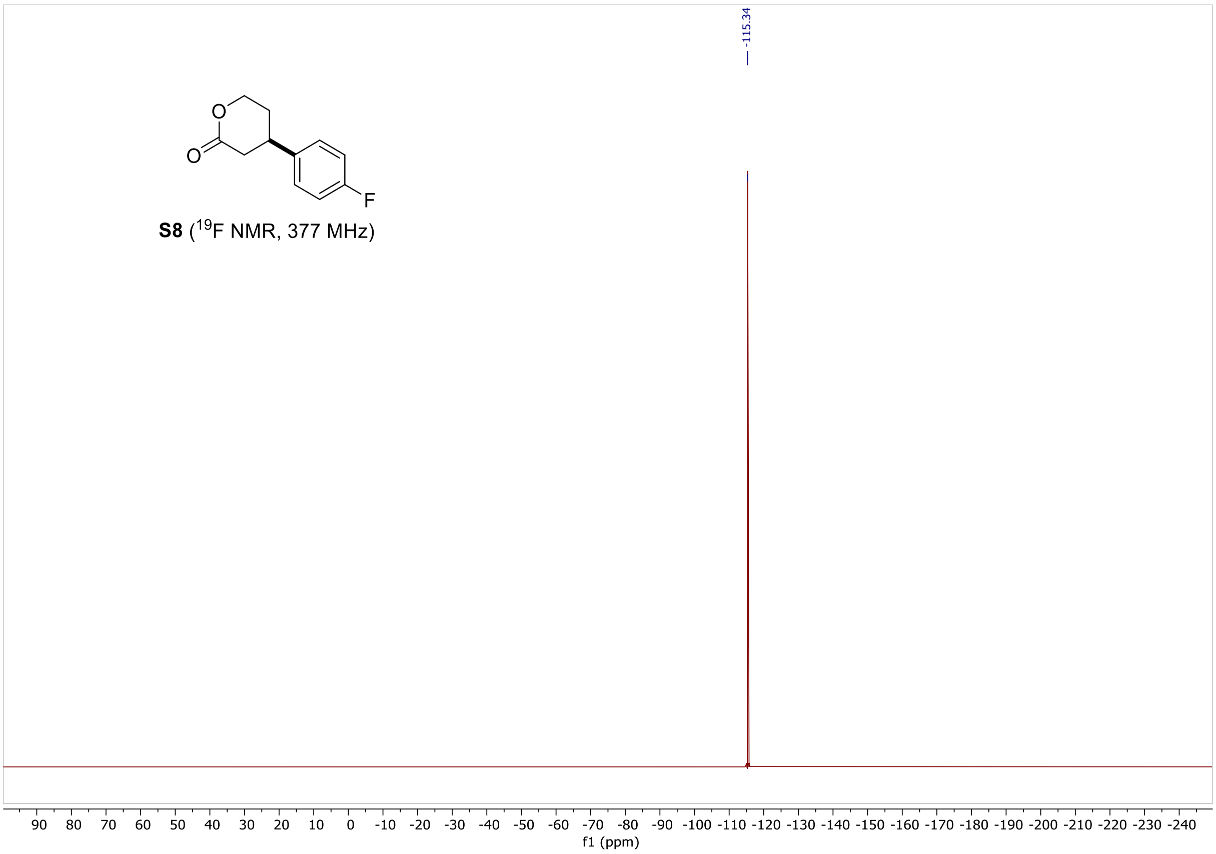
-115.34
S8 (19F NMR, 377 MHz)
90 80 70 60 50 40 30 20 10 0 -10 -20 -30 -40 -50 -60 -70 -80 -90 -100 -110 -120 -130 -140 -150 -160 -170 -180 -190 -200 -210 -220 -230 -240
f1 (ppm)

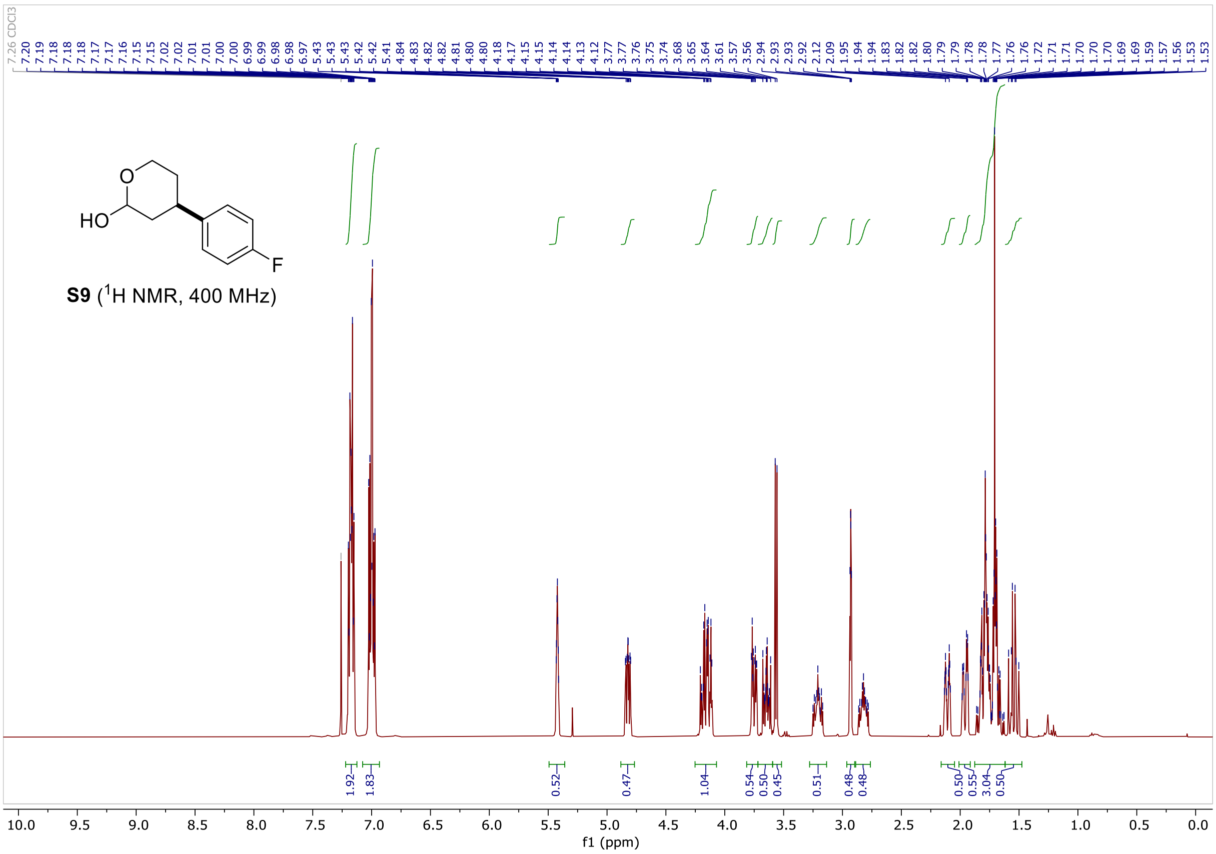

S9 (^{1}H NMR, 400 MHz)

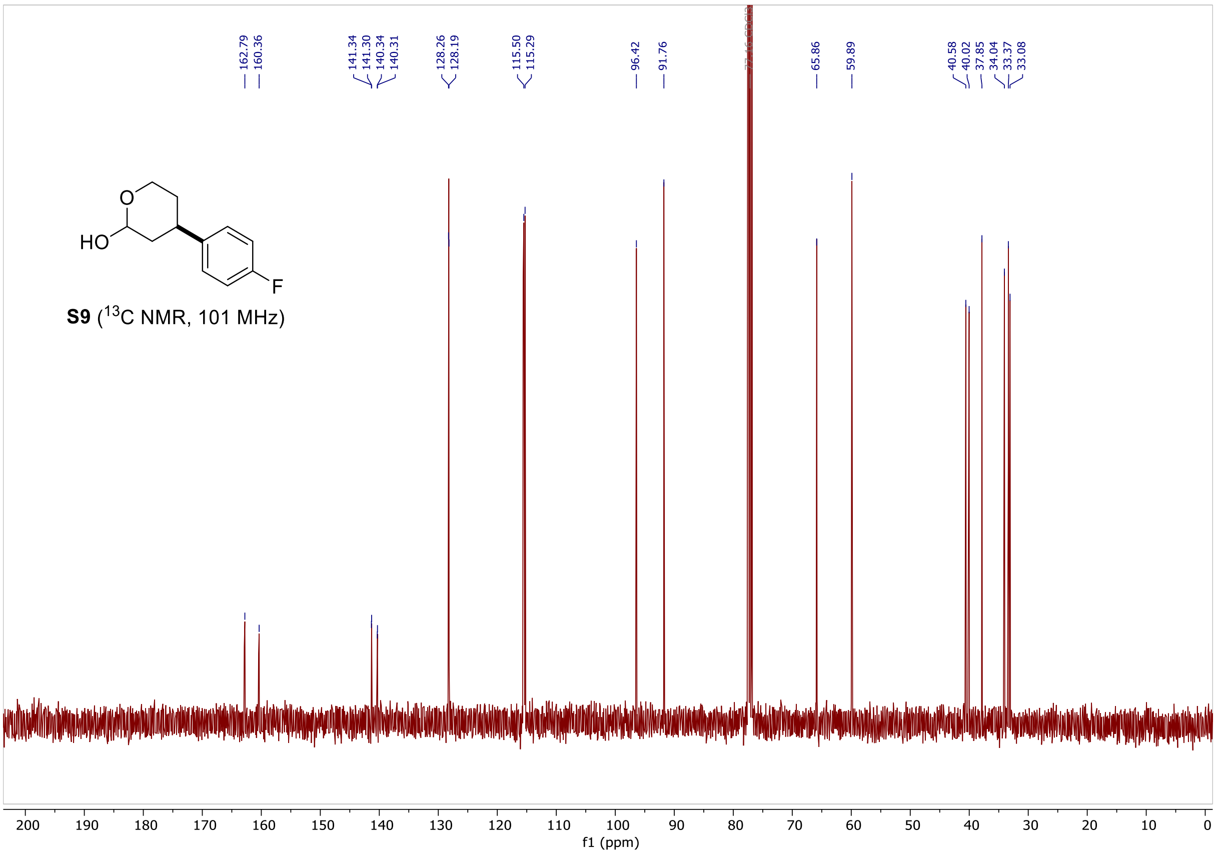

S9 (^{13}C NMR, 101 MHz)

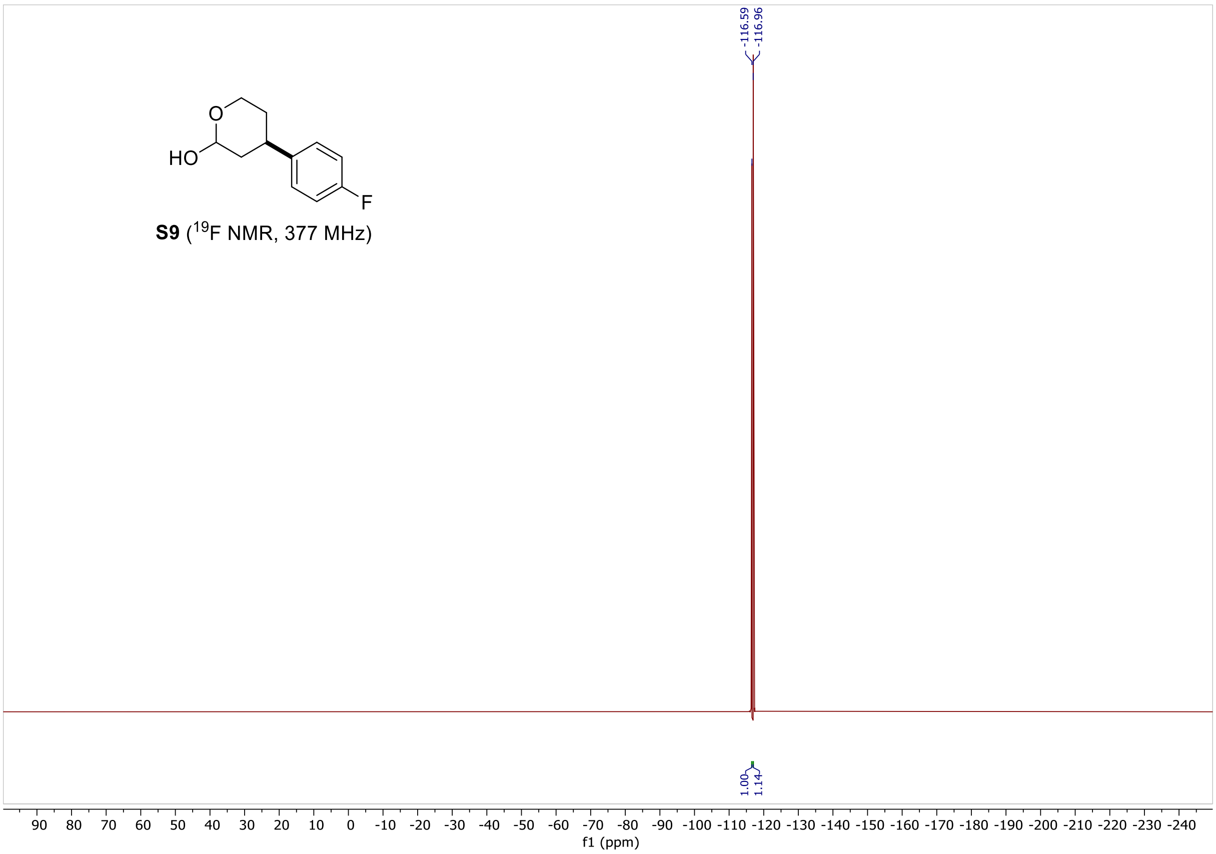

-116.59
-116.96
HO
O
F
S9 (19F NMR, 377 MHz)
1.00
1.14
90 80 70 60 50 40 30 20 10 0 -10 -20 -30 -40 -50 -60 -70 -80 -90 -100 -110 -120 -130 -140 -150 -160 -170 -180 -190 -200 -210 -220 -230 -240
f1 (ppm)

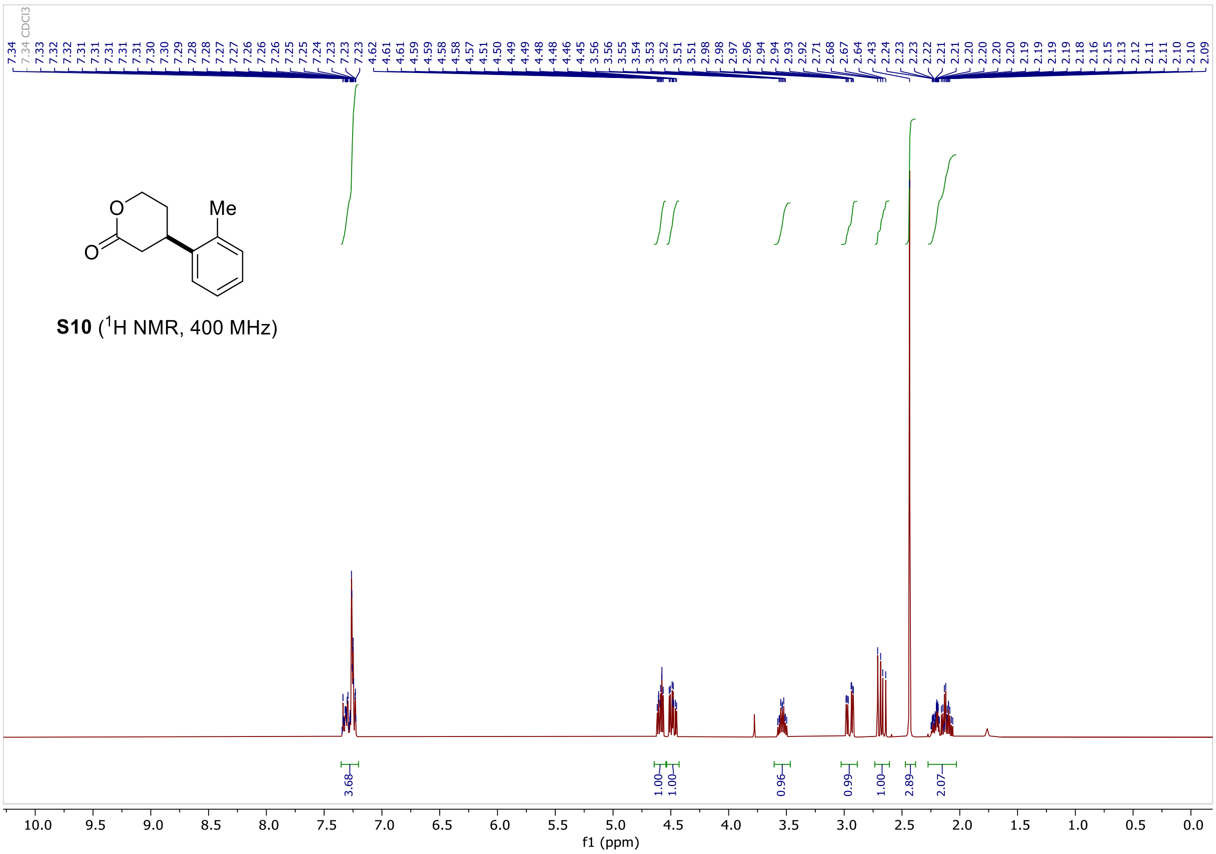
7.34
7.34 CDCl3
7.33
7.32
7.32
7.31
7.31
7.31
7.31
7.30
7.29
7.28
7.28
7.27
7.27
7.26
7.26
7.25
7.25
7.24
7.23
7.23
7.23
4.62
4.61
4.61
4.59
4.59
4.58
4.58
4.57
4.51
4.50
4.49
4.49
4.48
4.48
4.46
4.45
3.56
3.56
3.55
3.54
3.53
3.52
3.51
3.51
2.98
2.98
2.97
2.96
2.94
2.94
2.93
2.92
2.71
2.68
2.67
2.64
2.43
2.24
2.24
2.23
2.23
2.22
2.21
2.21
2.20
2.20
2.20
2.19
2.19
2.19
2.19
2.18
2.16
2.15
2.13
2.12
2.11
2.11
2.10
2.09
O
O
Me
S10 (1H NMR, 400 MHz)
3.68
1.00
1.00
0.96
0.99
1.00
2.89
2.07
10.0 9.5 9.0 8.5 8.0 7.5 7.0 6.5 6.0 5.5 5.0 4.5 4.0 3.5 3.0 2.5 2.0 1.5 1.0 0.5 0.0
f1 (ppm)

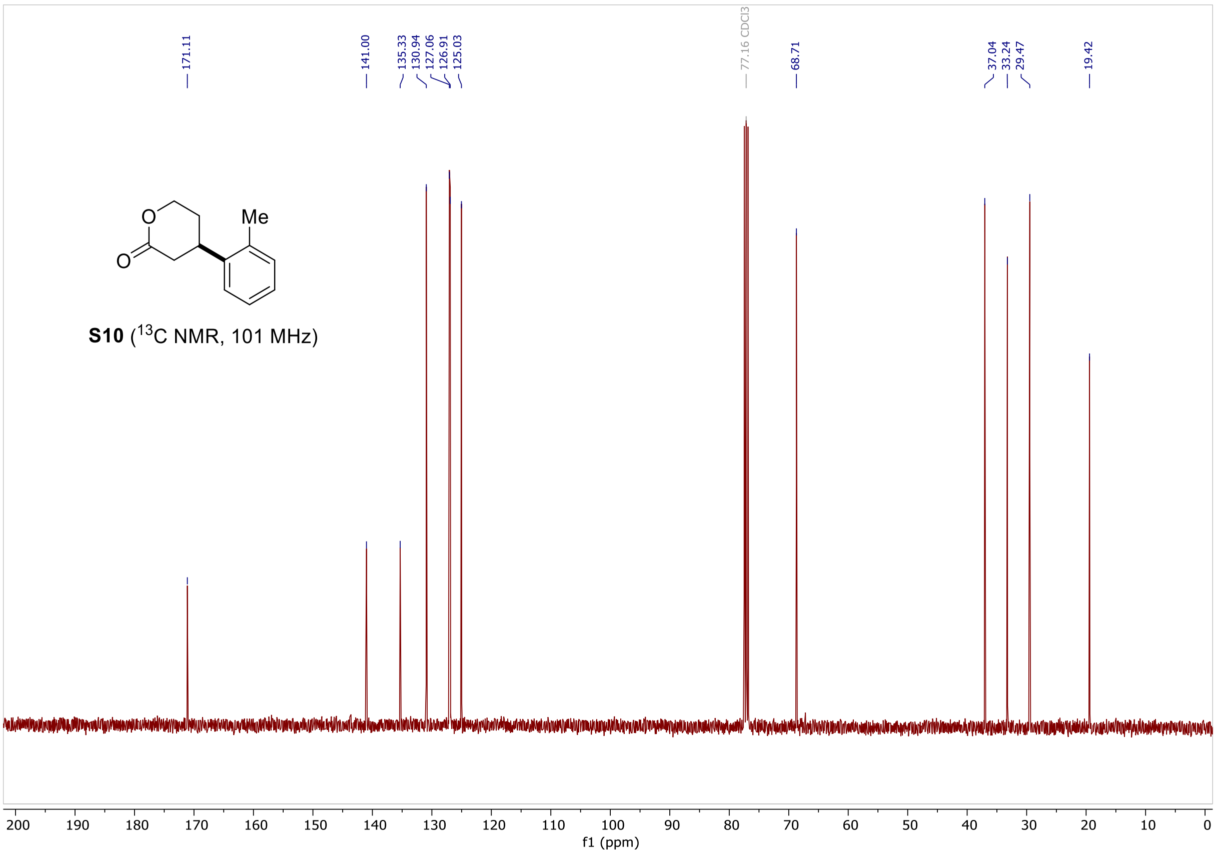
171.11
141.00
135.33
130.94
127.06
126.91
125.03
77.16 CDCl3
68.71
37.04
33.24
29.47
19.42
O
O
Me
S10 (13C NMR, 101 MHz)
200 190 180 170 160 150 140 130 120 110 100 90 80 70 60 50 40 30 20 10 0
f1 (ppm)

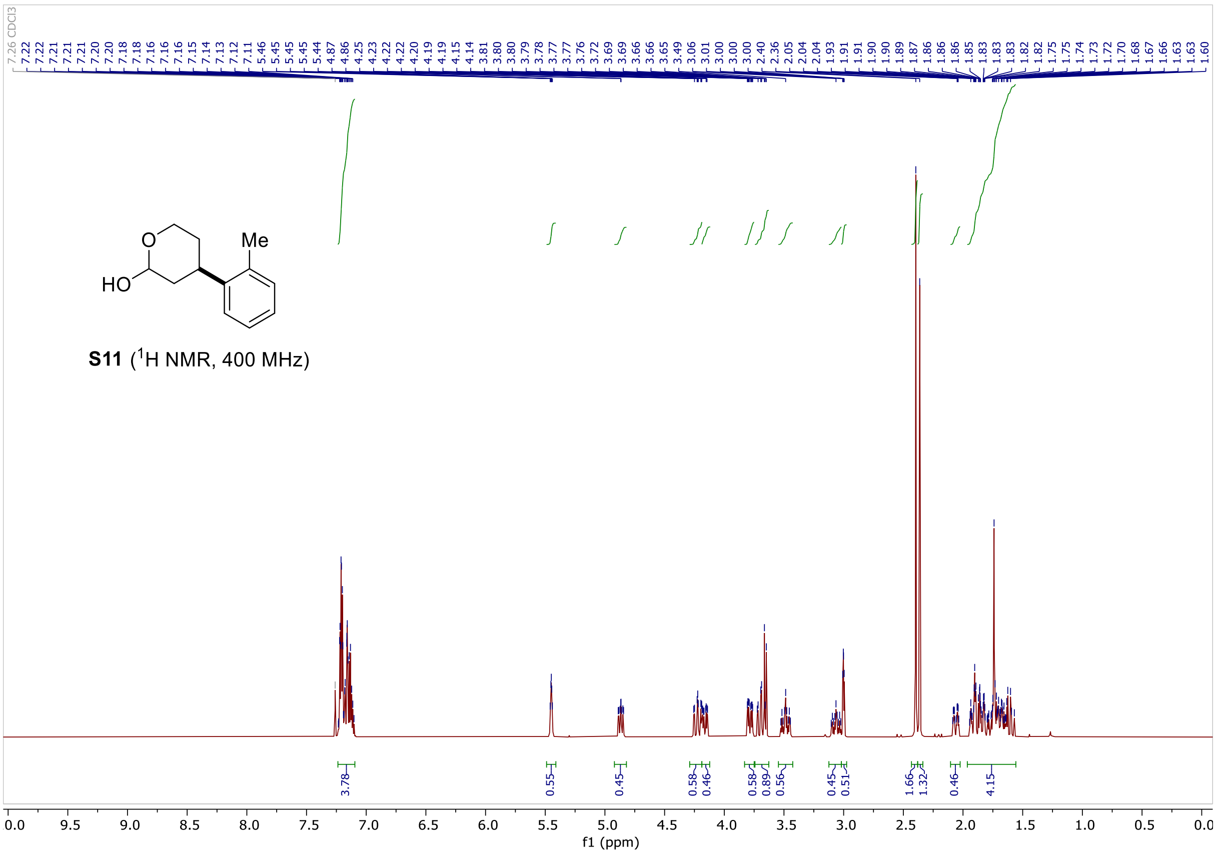
Me
HO
O
S11 (1H NMR, 400 MHz)
f1 (ppm)

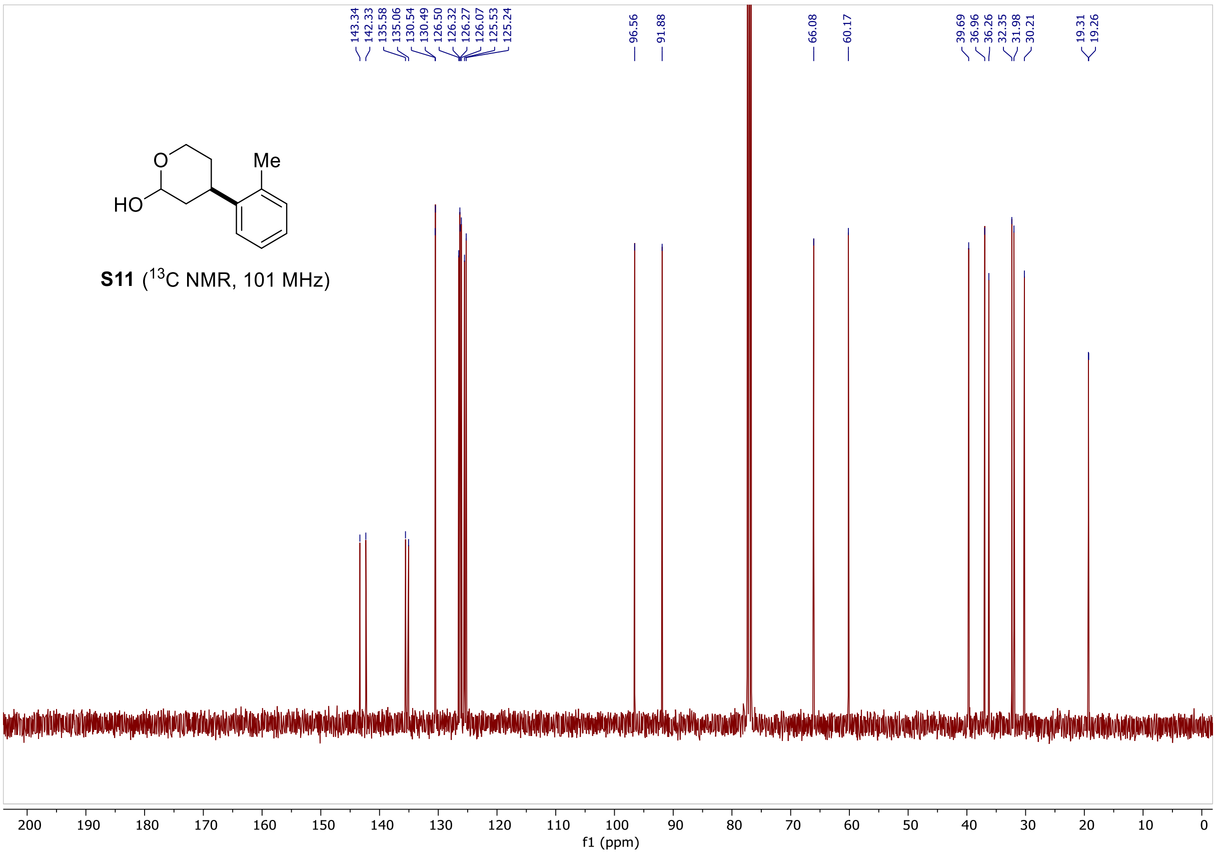
143.34
142.33
135.58
135.06
130.54
130.49
126.50
126.32
126.27
126.07
125.53
125.24
96.56
91.88
66.08
60.17
39.69
36.96
36.26
32.35
31.98
30.21
19.31
19.26
Me
HO
O
S11 (13C NMR, 101 MHz)
f1 (ppm)

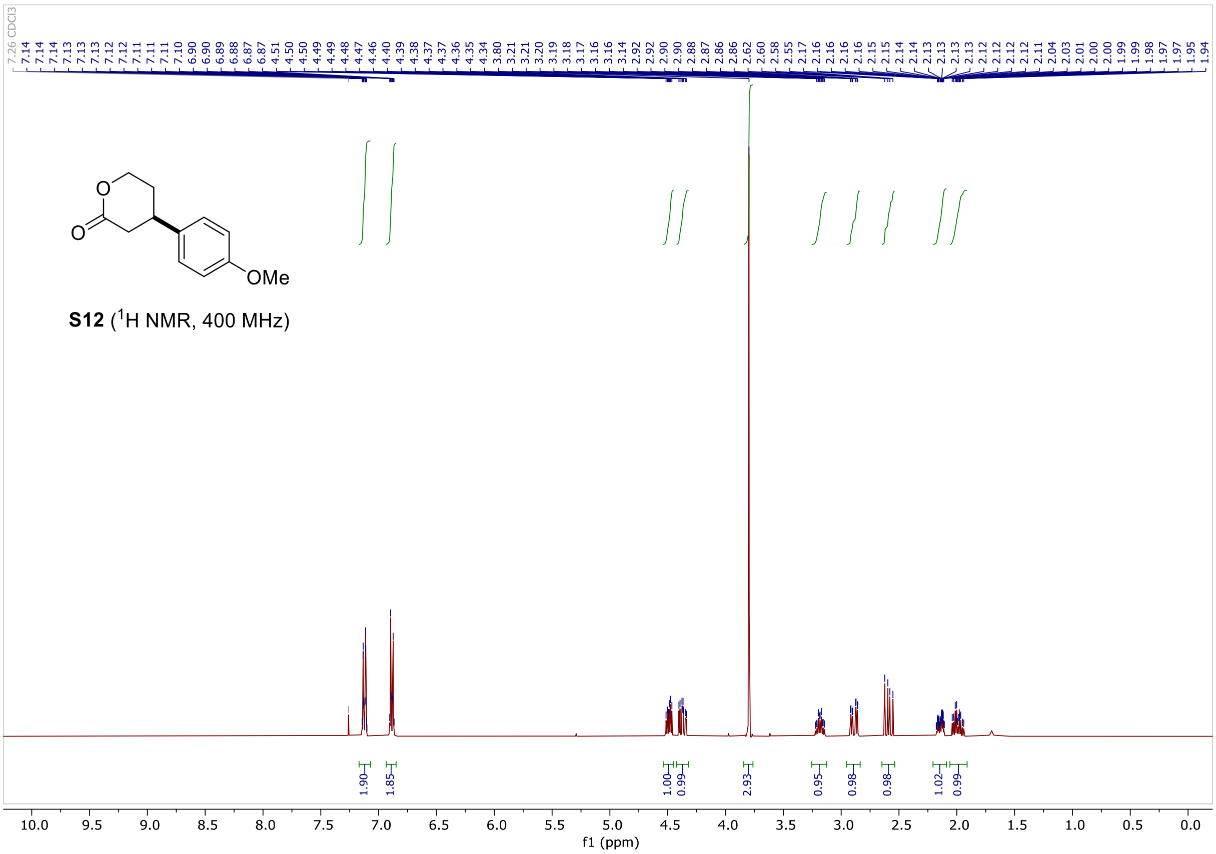

OMe
S12 (1H NMR, 400 MHz)
1.90
1.85
1.00
0.99
2.93
0.95
0.98
0.98
1.02
0.99
10.0 9.5 9.0 8.5 8.0 7.5 7.0 6.5 6.0 5.5 5.0 4.5 4.0 3.5 3.0 2.5 2.0 1.5 1.0 0.5 0.0
f1 (ppm)

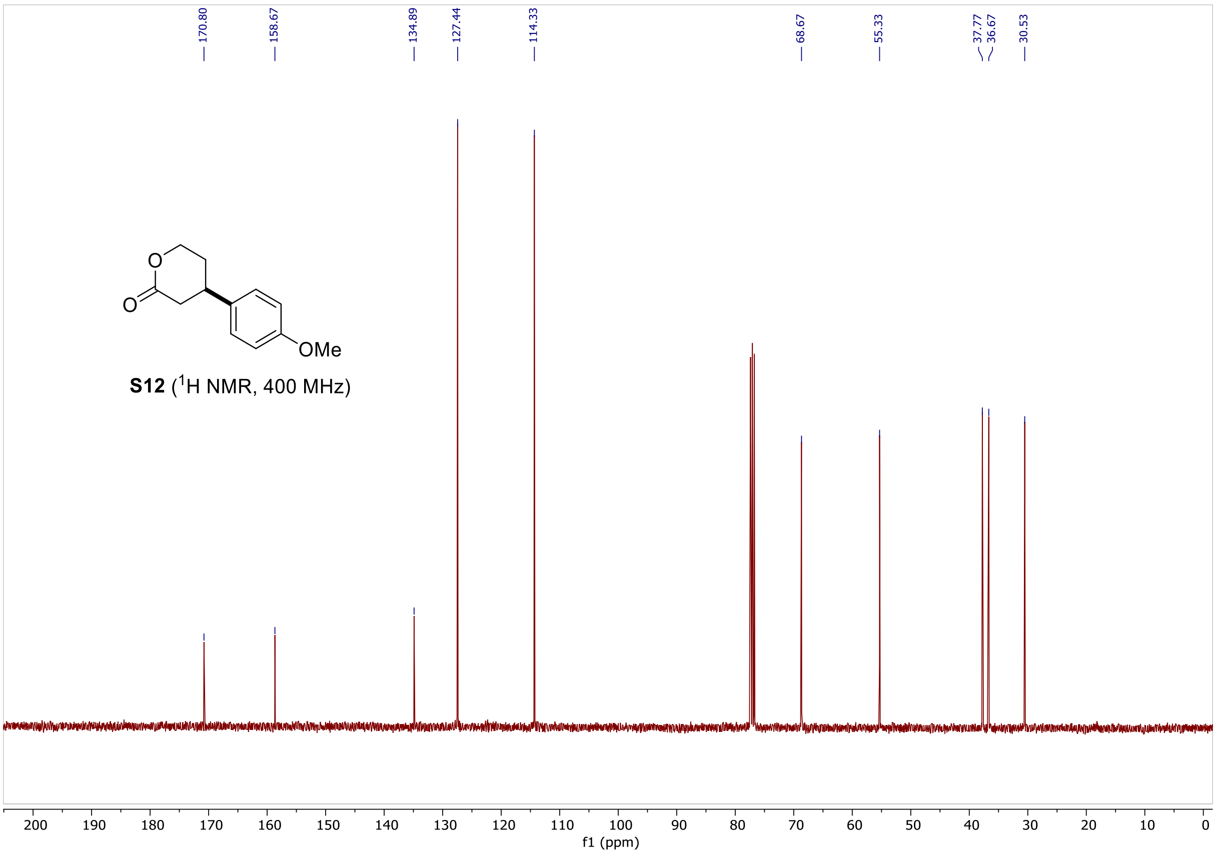

170.80
158.67
134.89
127.44
114.33
68.67
55.33
37.77
36.67
30.53
OMe
S12 (1H NMR, 400 MHz)
200 190 180 170 160 150 140 130 120 110 100 90 80 70 60 50 40 30 20 10 0
f1 (ppm)

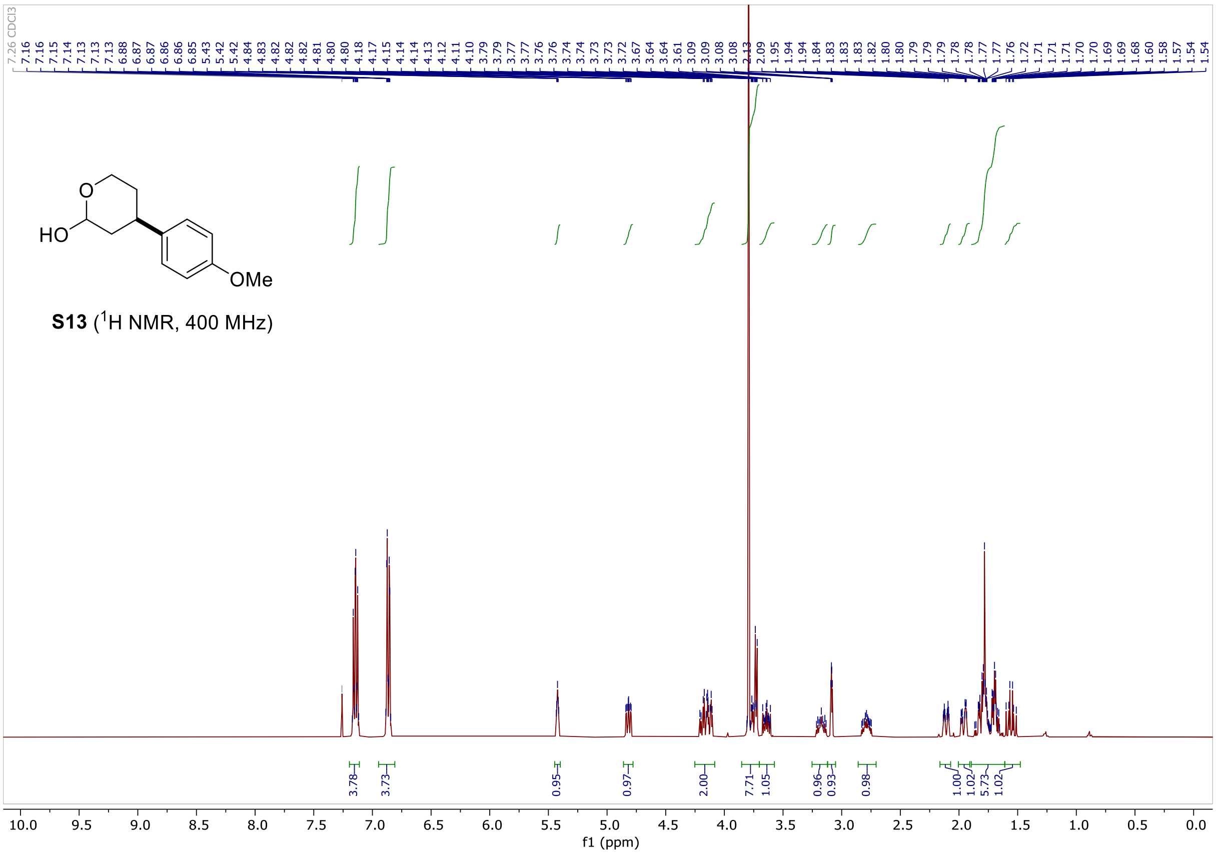
HO
O
OMe
S13 (1H NMR, 400 MHz)
f1 (ppm)

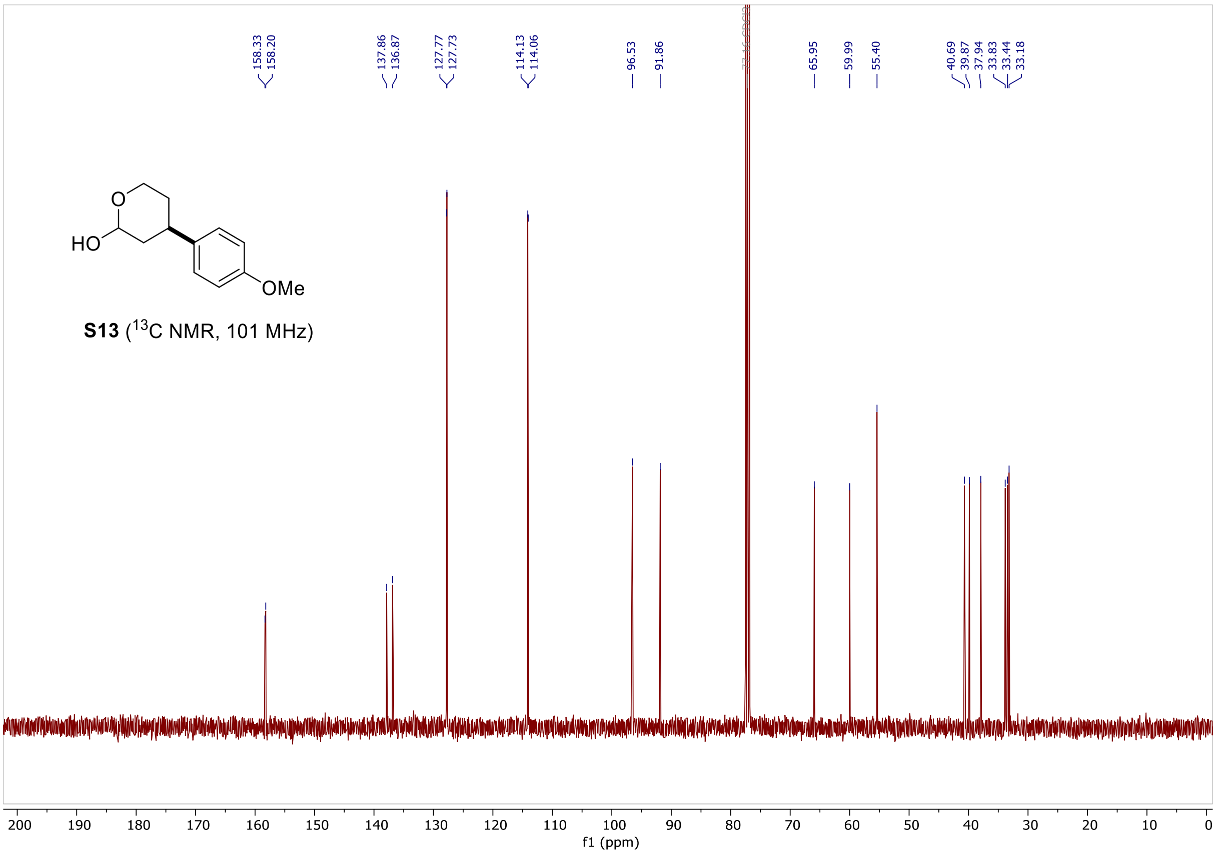
HO
O
OMe
S13 (13C NMR, 101 MHz)
f1 (ppm)

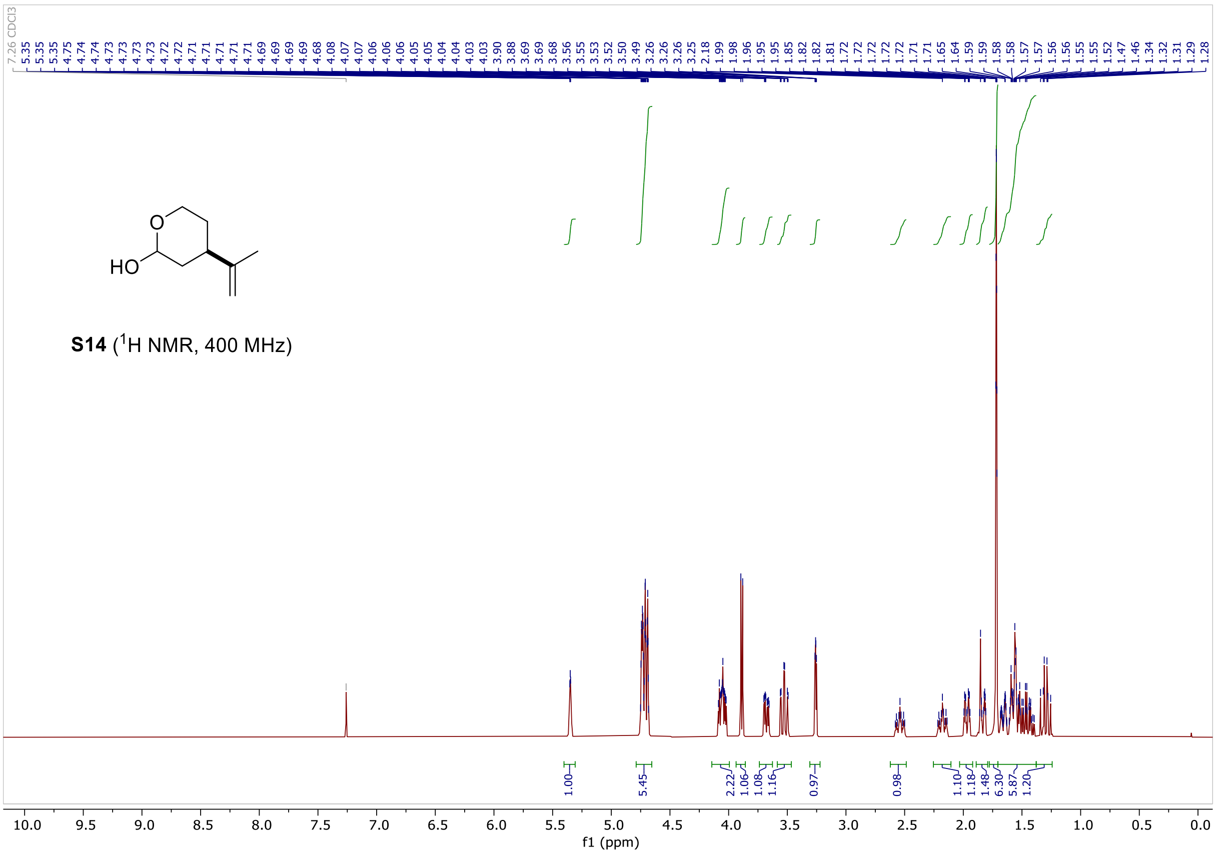

S14 (^{1}H NMR, 400 MHz)

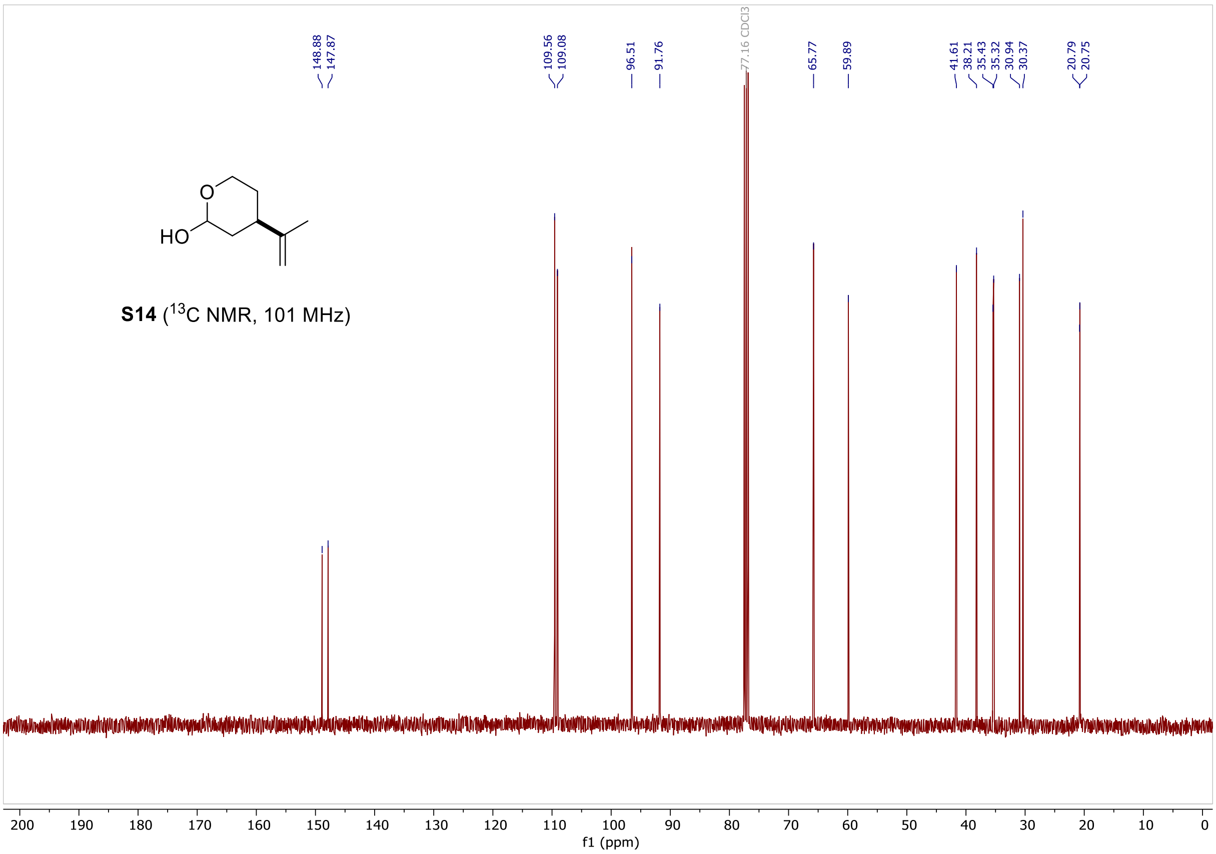

S14 (^{13}C NMR, 101 MHz)

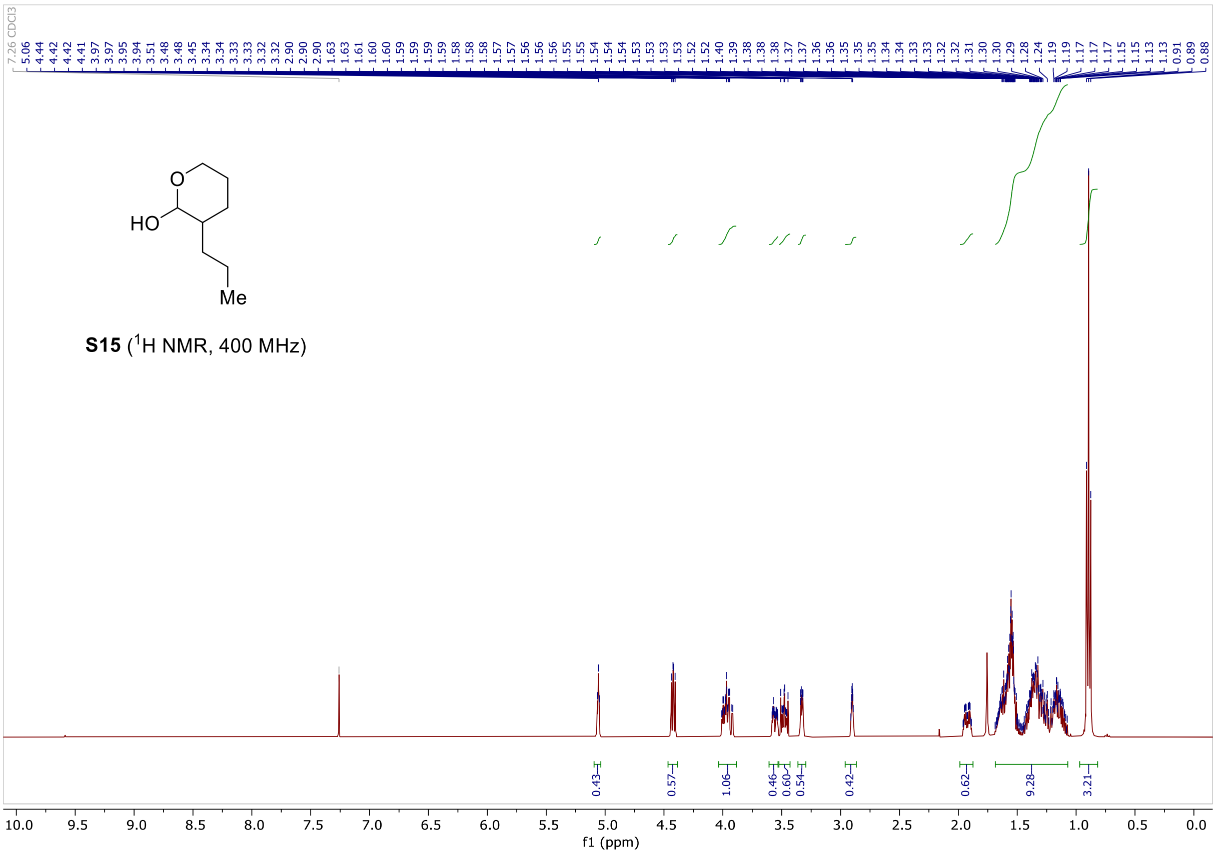
HO
O
Me
S15 (1H NMR, 400 MHz)
f1 (ppm)

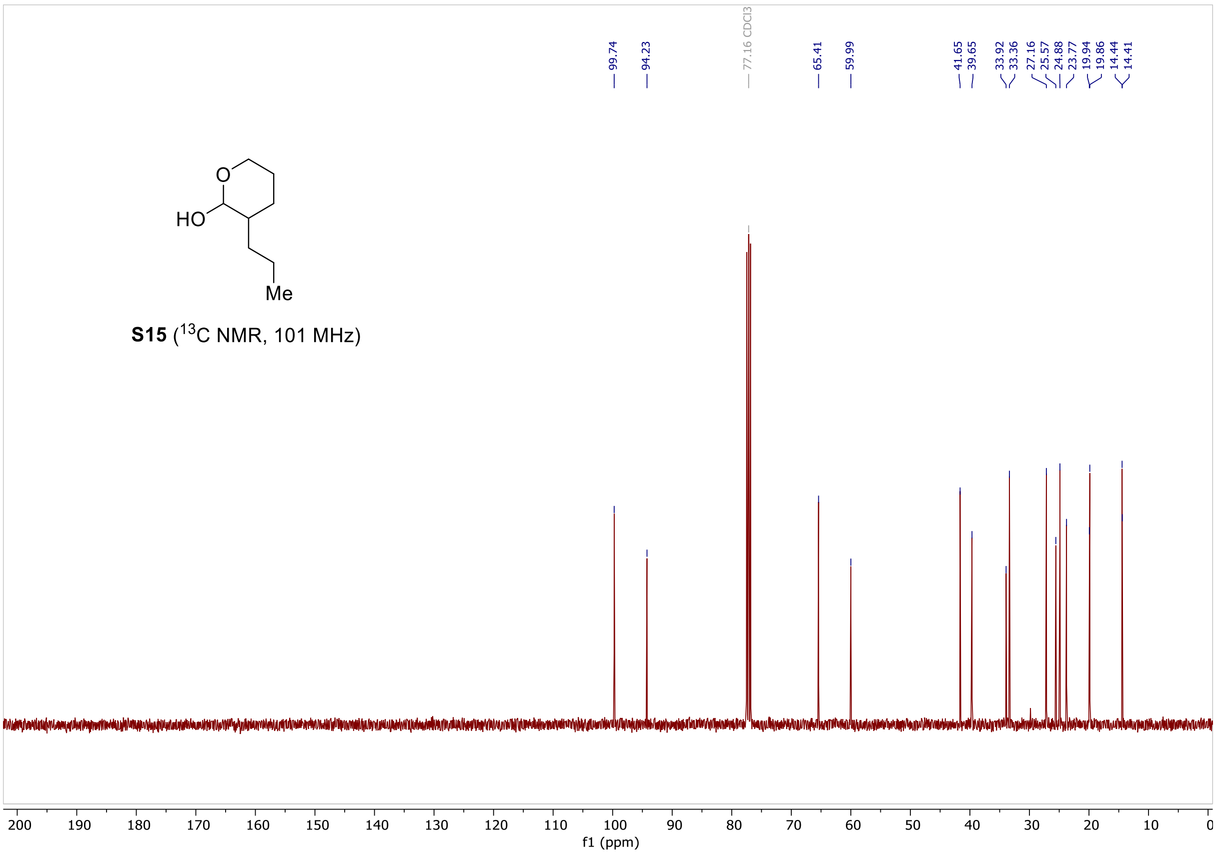
HO
O
Me
S15 (13C NMR, 101 MHz)
f1 (ppm)

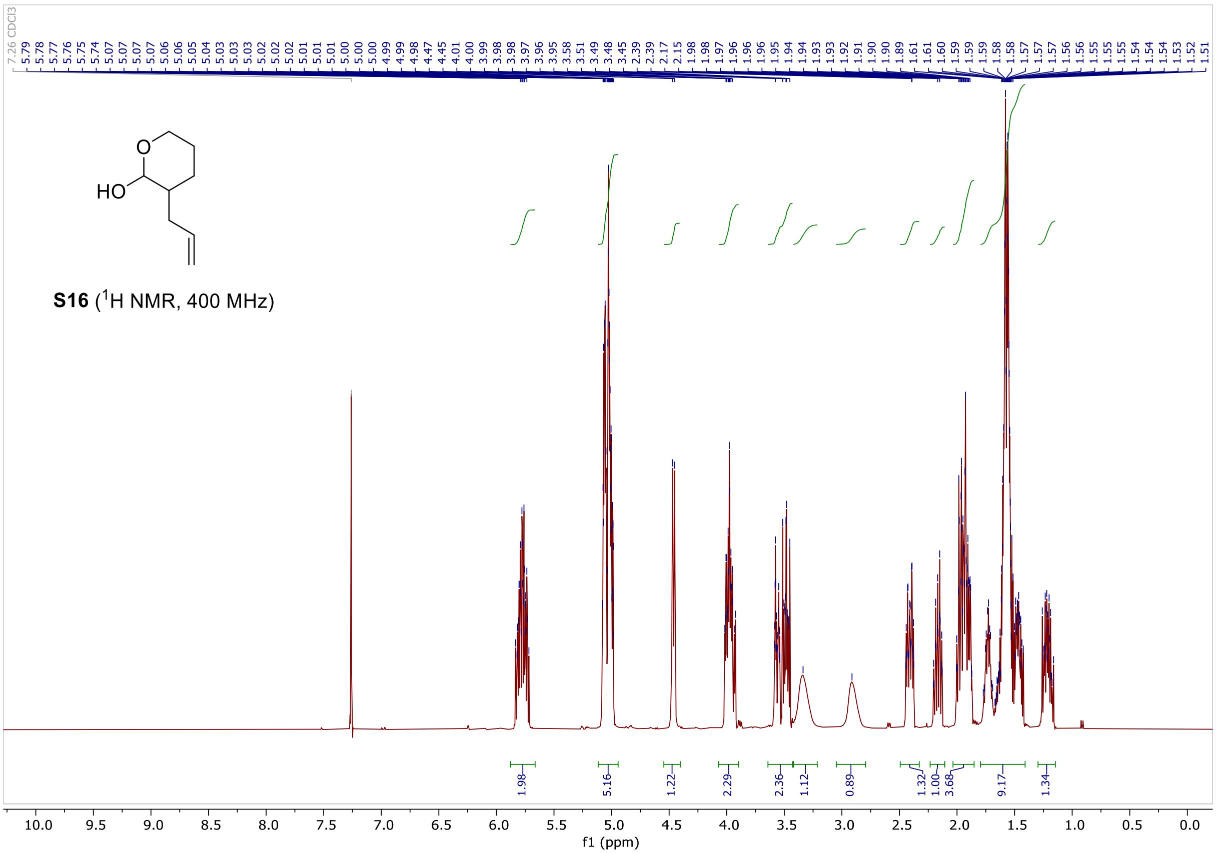
S16 (1H NMR, 400 MHz)
f1 (ppm)

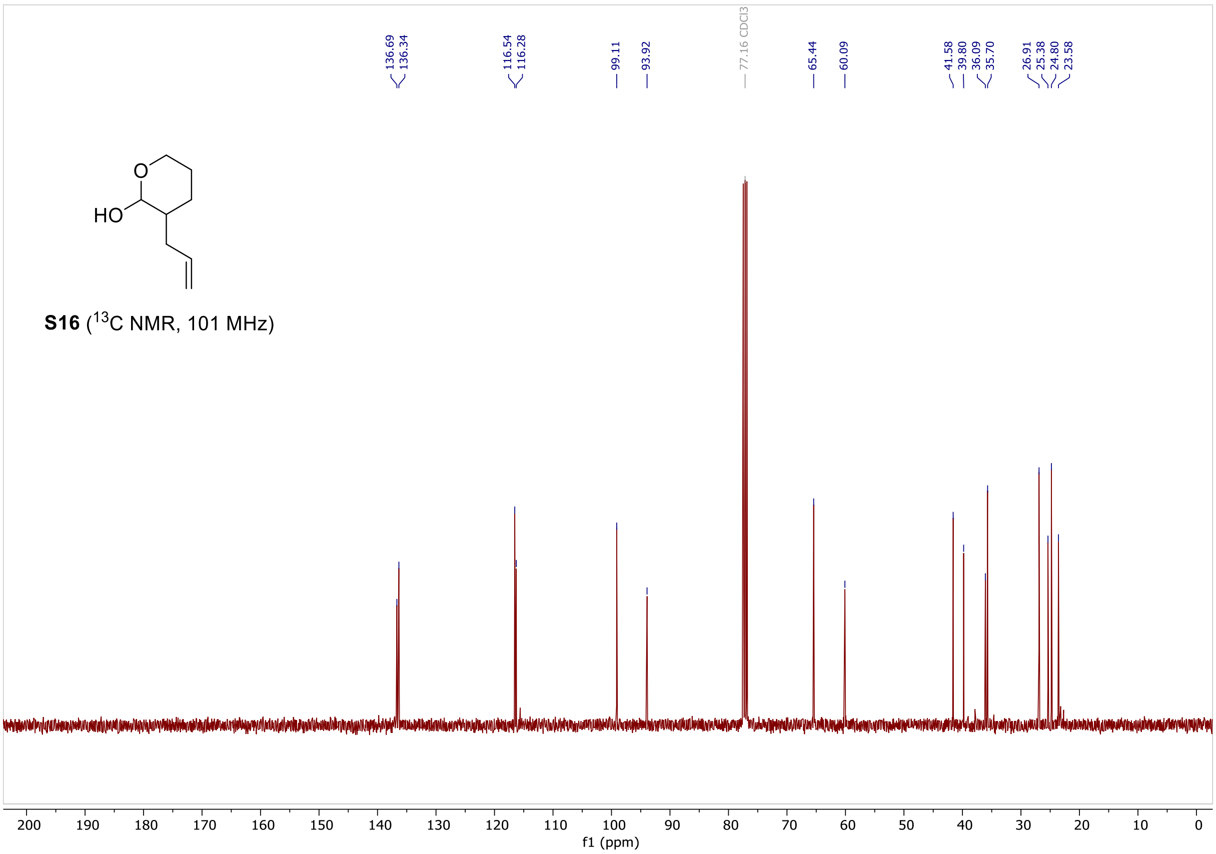
S16 (13C NMR, 101 MHz)
136.69
136.34
116.54
116.28
99.11
93.92
77.16 CDCl3
65.44
60.09
41.58
39.80
36.09
35.70
26.91
25.38
24.80
23.58
f1 (ppm)

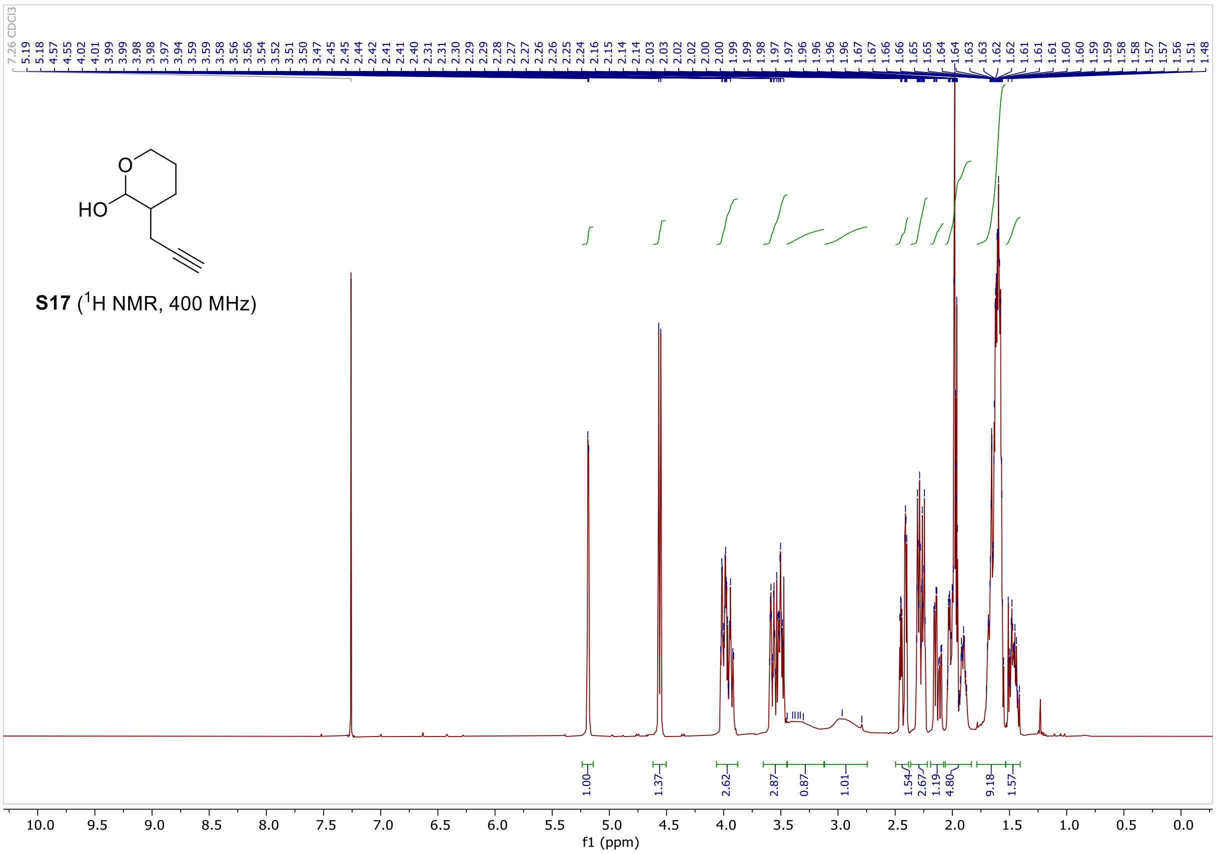

HO
O
S17 (^{1}H NMR, 400 MHz)
f1 (ppm)

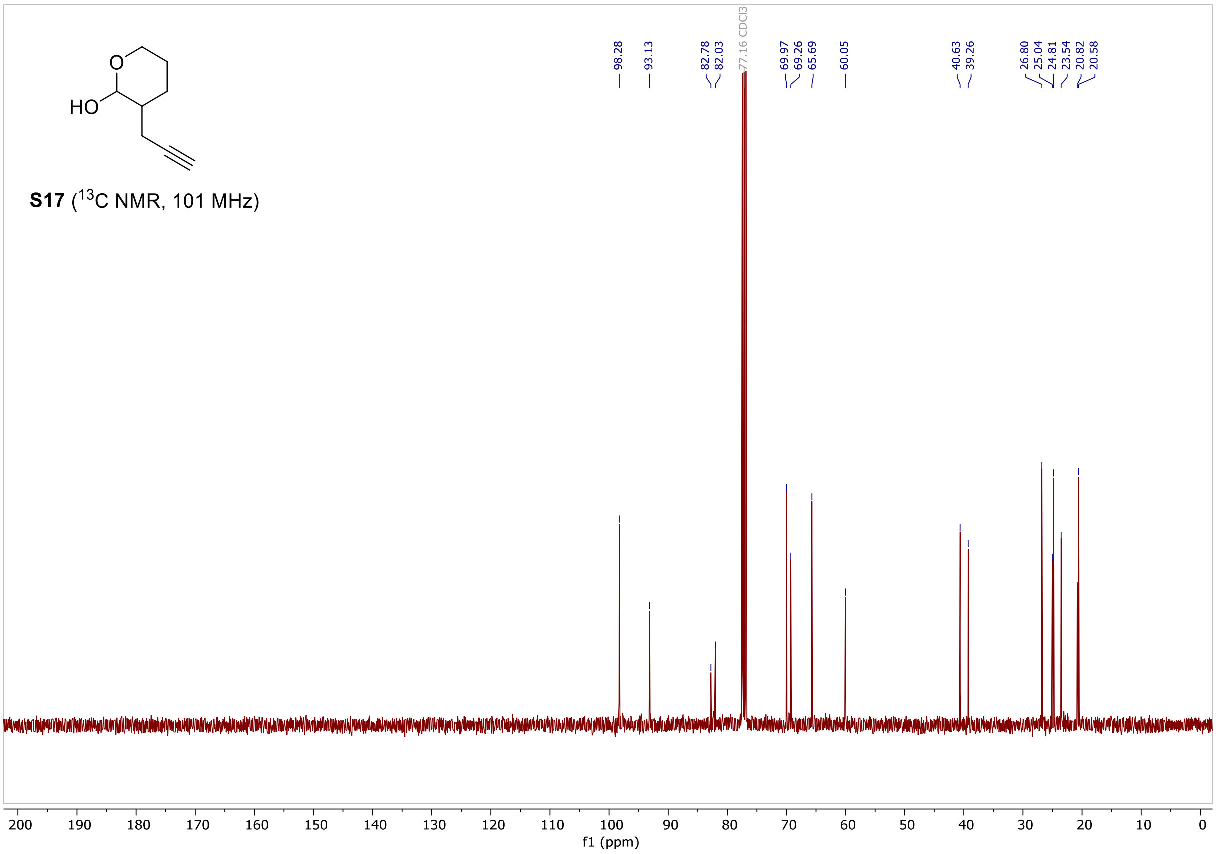

HO
O
S17 (^{13}C NMR, 101 MHz)
98.28
93.13
82.78
82.03
77.16 CDCl3
69.97
69.26
65.69
60.05
40.63
39.26
26.80
25.04
24.81
23.54
20.82
20.58
f1 (ppm)

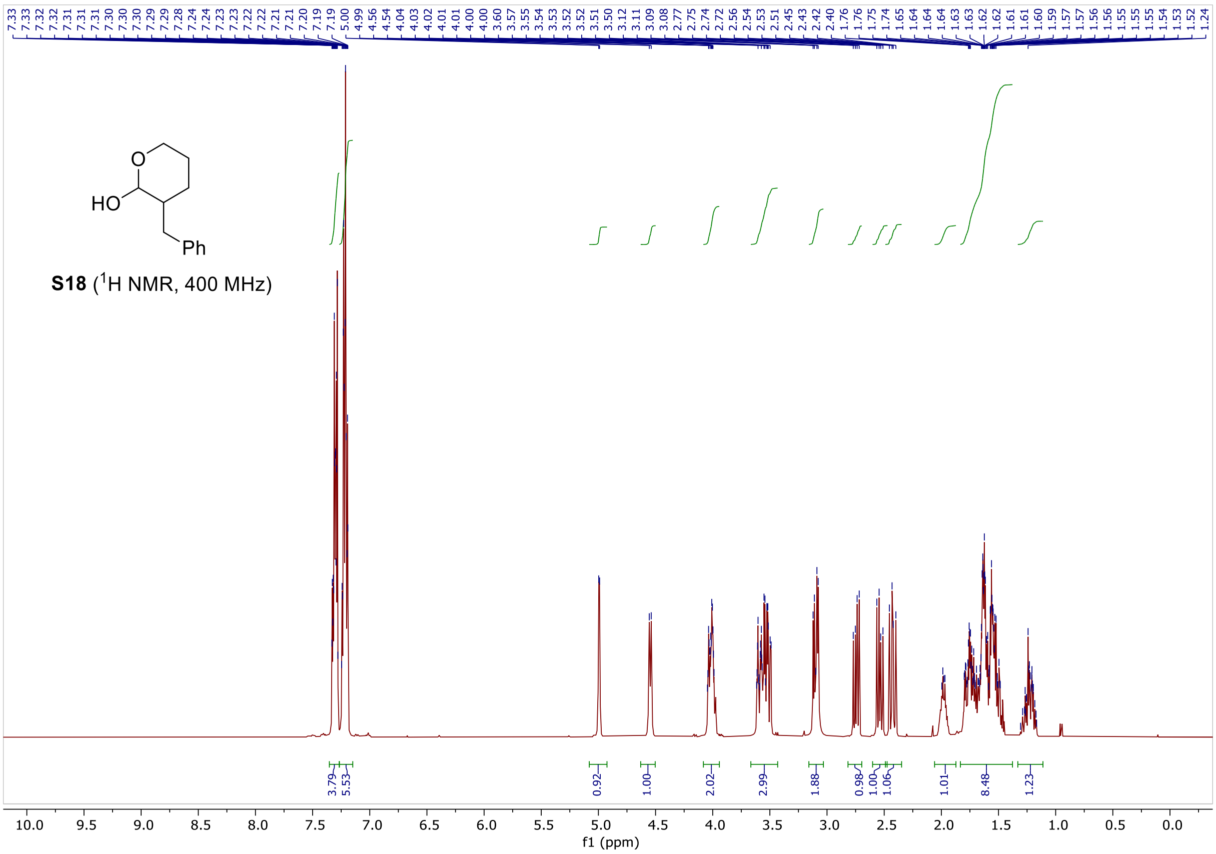
HO
O
Ph
S18 (1H NMR, 400 MHz)
f1 (ppm)

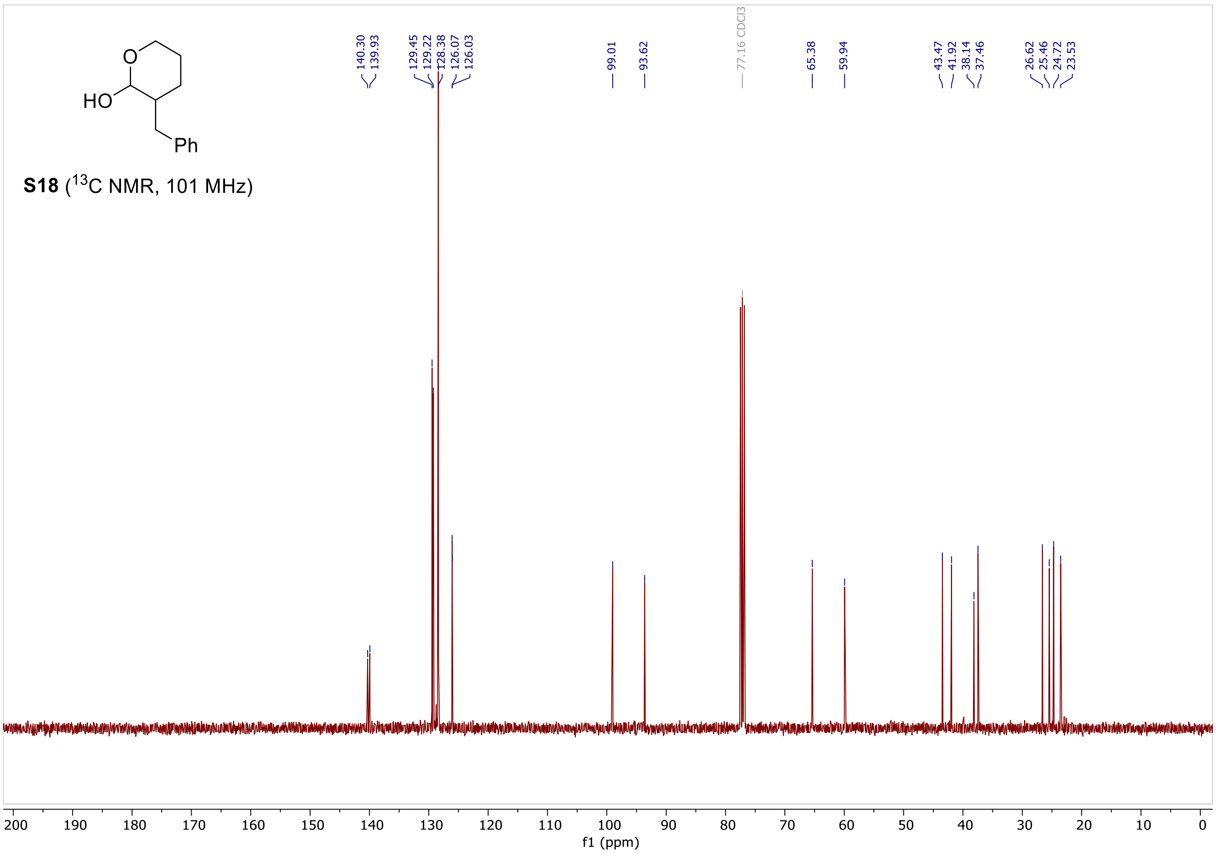
HO
O
Ph
S18 (13C NMR, 101 MHz)
77.16 CDCl3
f1 (ppm)

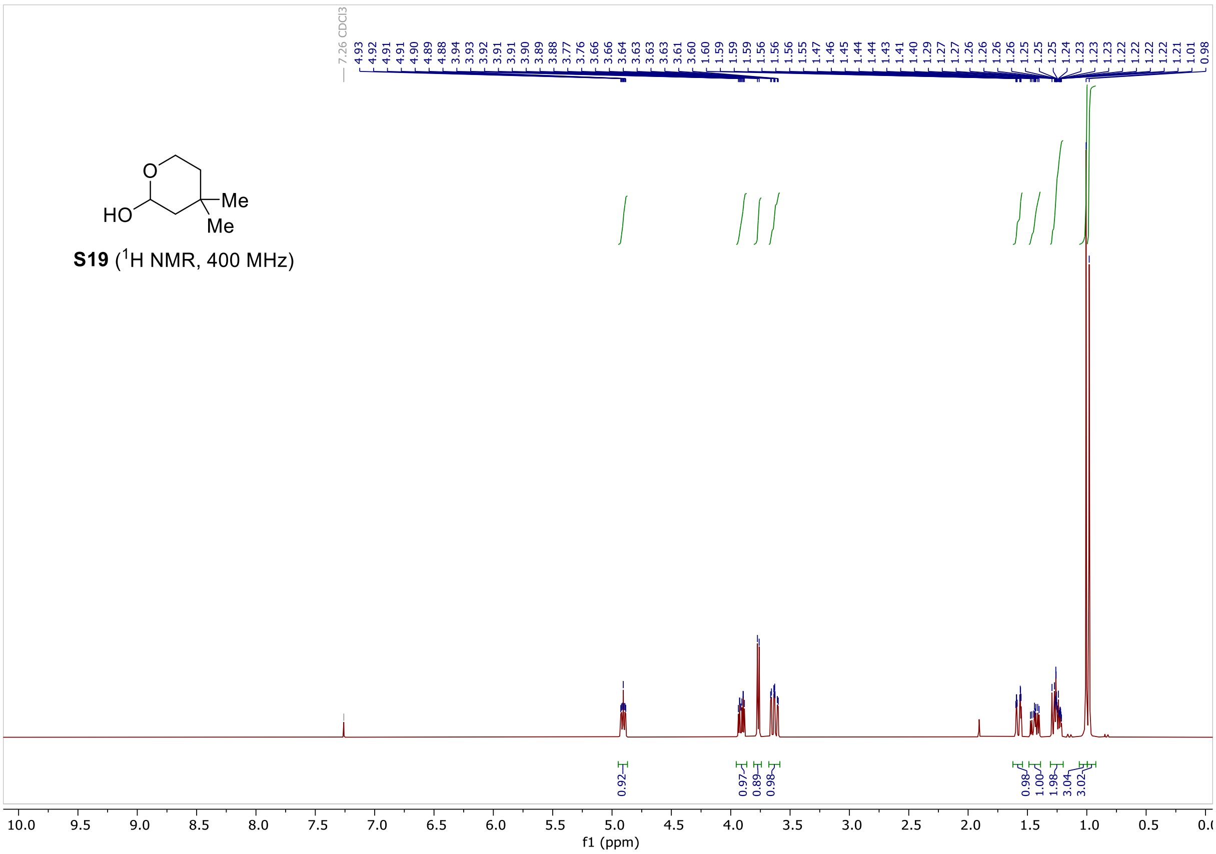

7.26 CDCl3
4.93 4.92 4.91 4.91 4.90 4.89 4.88 3.94 3.93 3.92 3.91 3.91 3.90 3.89 3.88 3.77 3.76 3.66 3.64 3.63 3.63 3.63 3.61 3.60 1.60 1.59 1.59 1.59 1.56 1.56 1.56 1.55 1.47 1.46 1.45 1.44 1.44 1.43 1.41 1.40 1.29 1.27 1.27 1.26 1.26 1.26 1.25 1.25 1.25 1.24 1.23 1.23 1.23 1.22 1.22 1.22 1.22 1.21 1.01 0.98
O
HO
Me
Me
S19 (1H NMR, 400 MHz)
0.92 0.97 0.89 0.98 0.98 1.00 1.98 3.04 3.02
10.0 9.5 9.0 8.5 8.0 7.5 7.0 6.5 6.0 5.5 5.0 4.5 4.0 3.5 3.0 2.5 2.0 1.5 1.0 0.5
f1 (ppm)

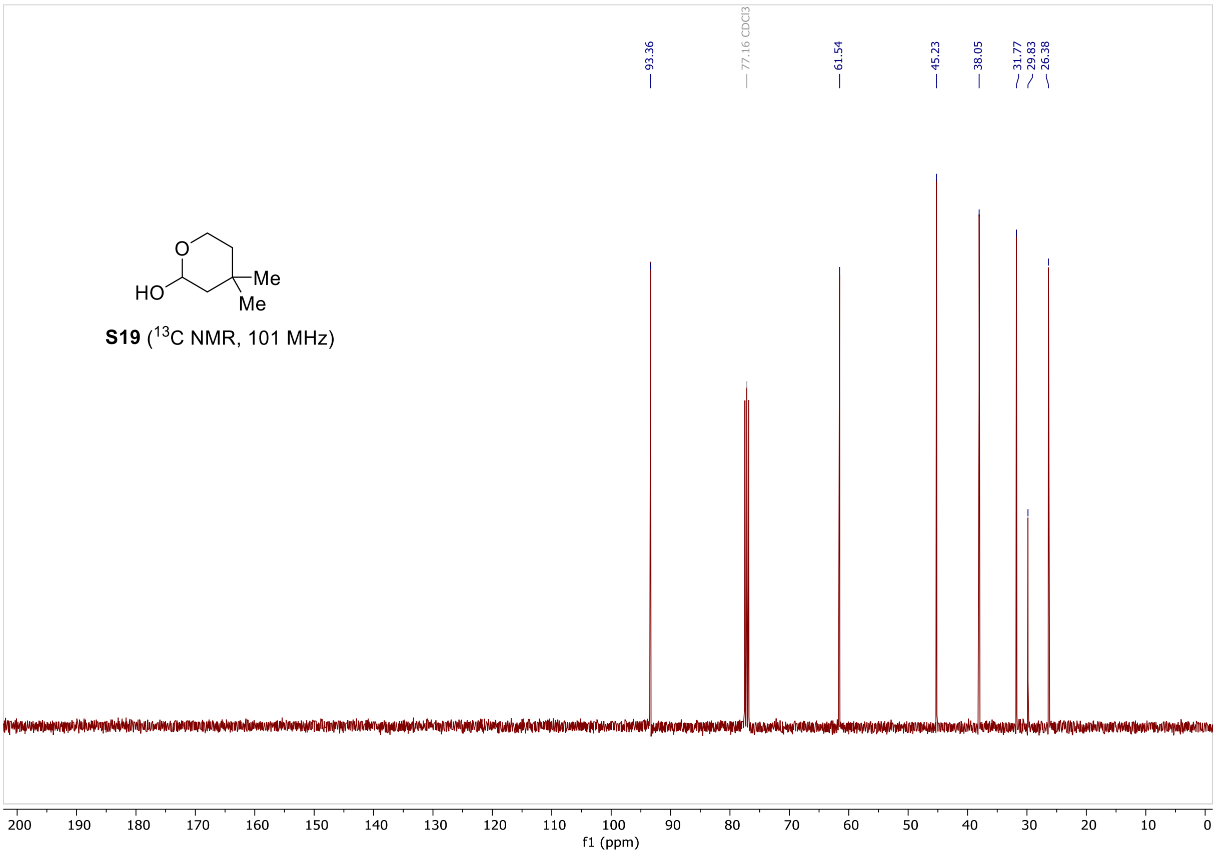

93.36
77.16 CDCl3
61.54
45.23
38.05
31.77 29.83 26.38
O
HO
Me
Me
S19 (13C NMR, 101 MHz)
200 190 180 170 160 150 140 130 120 110 100 90 80 70 60 50 40 30 20 10 0
f1 (ppm)

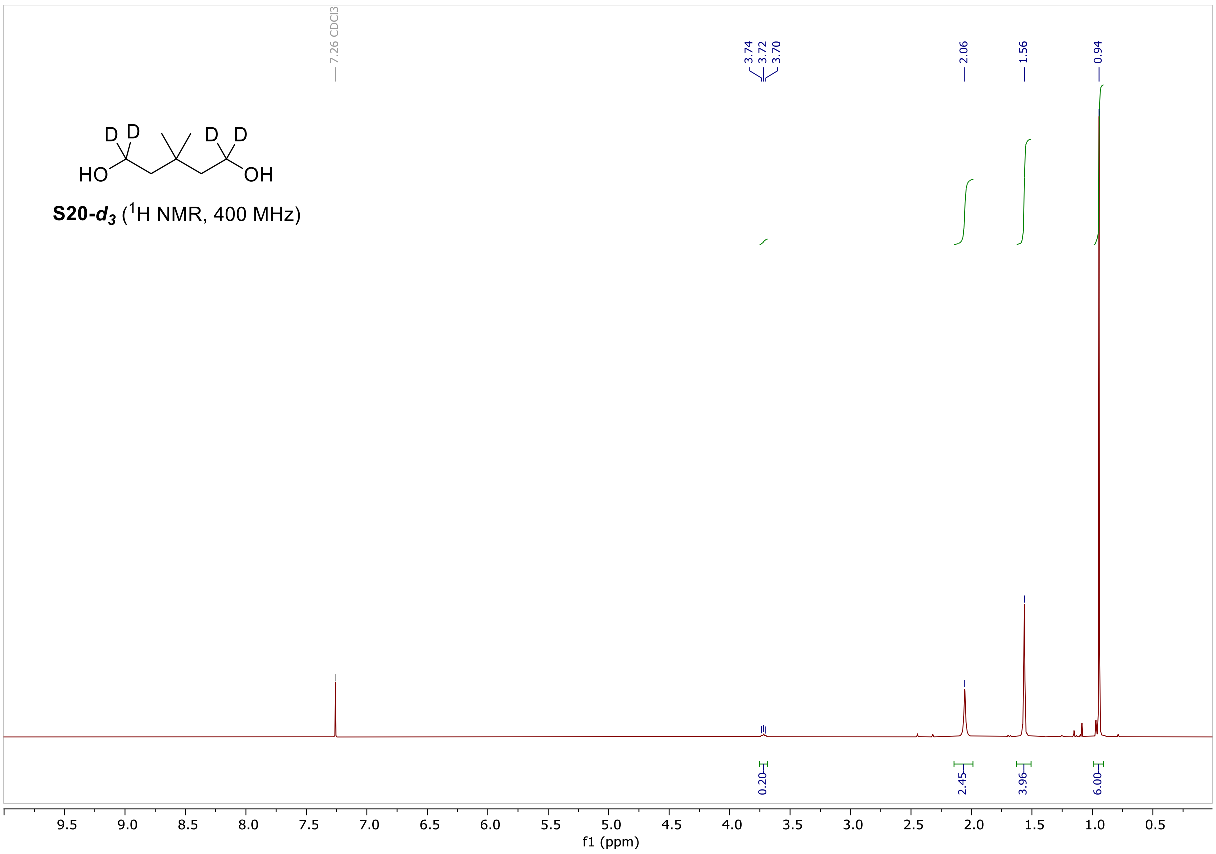
7.26 CDCl3
3.74
3.72
3.70
2.06
1.56
0.94
D D D D
HO OH
S20-d3 (1H NMR, 400 MHz)
0.20
2.45
3.96
6.00
9.5 9.0 8.5 8.0 7.5 7.0 6.5 6.0 5.5 5.0 4.5 4.0 3.5 3.0 2.5 2.0 1.5 1.0 0.5
f1 (ppm)

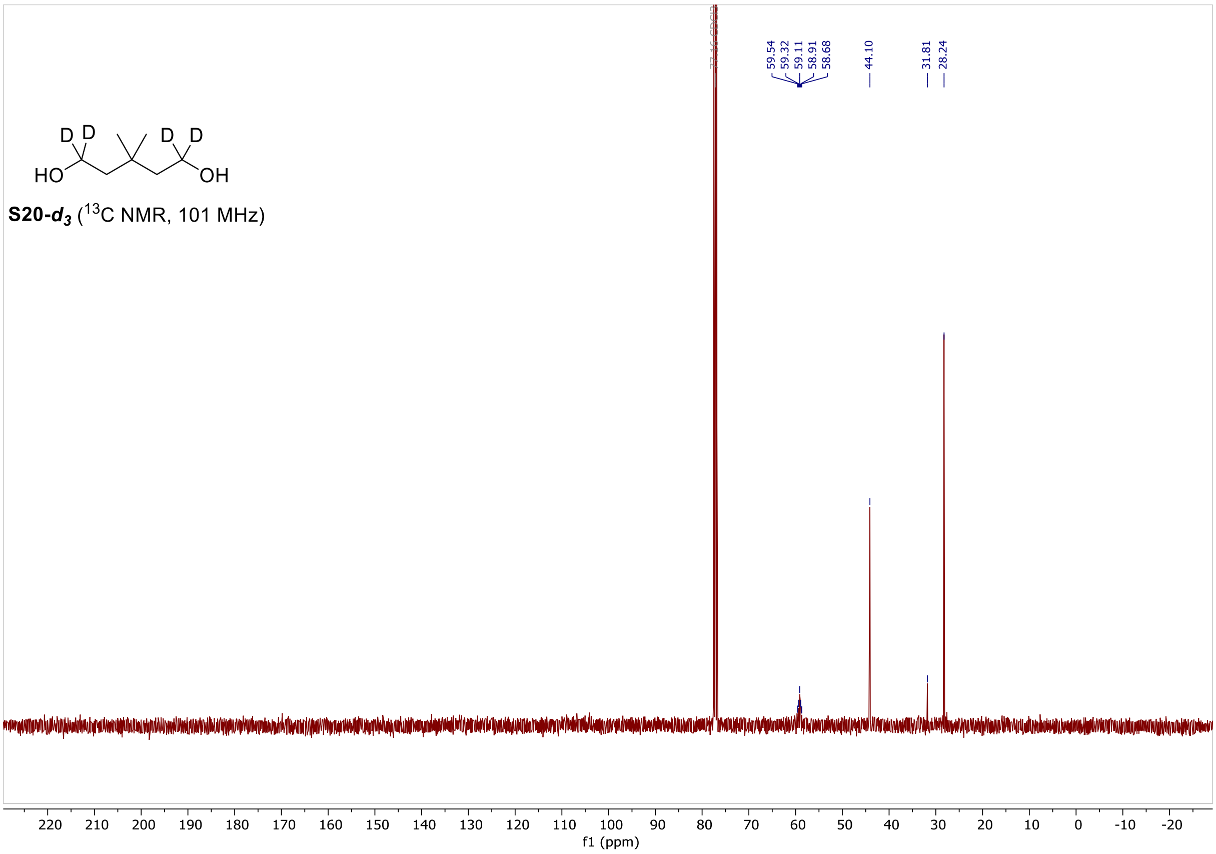
59.54
59.32
59.11
58.91
58.68
44.10
31.81
28.24
D D D D
HO OH
S20-d3 (13C NMR, 101 MHz)
220 210 200 190 180 170 160 150 140 130 120 110 100 90 80 70 60 50 40 30 20 10 0 -10 -20
f1 (ppm)

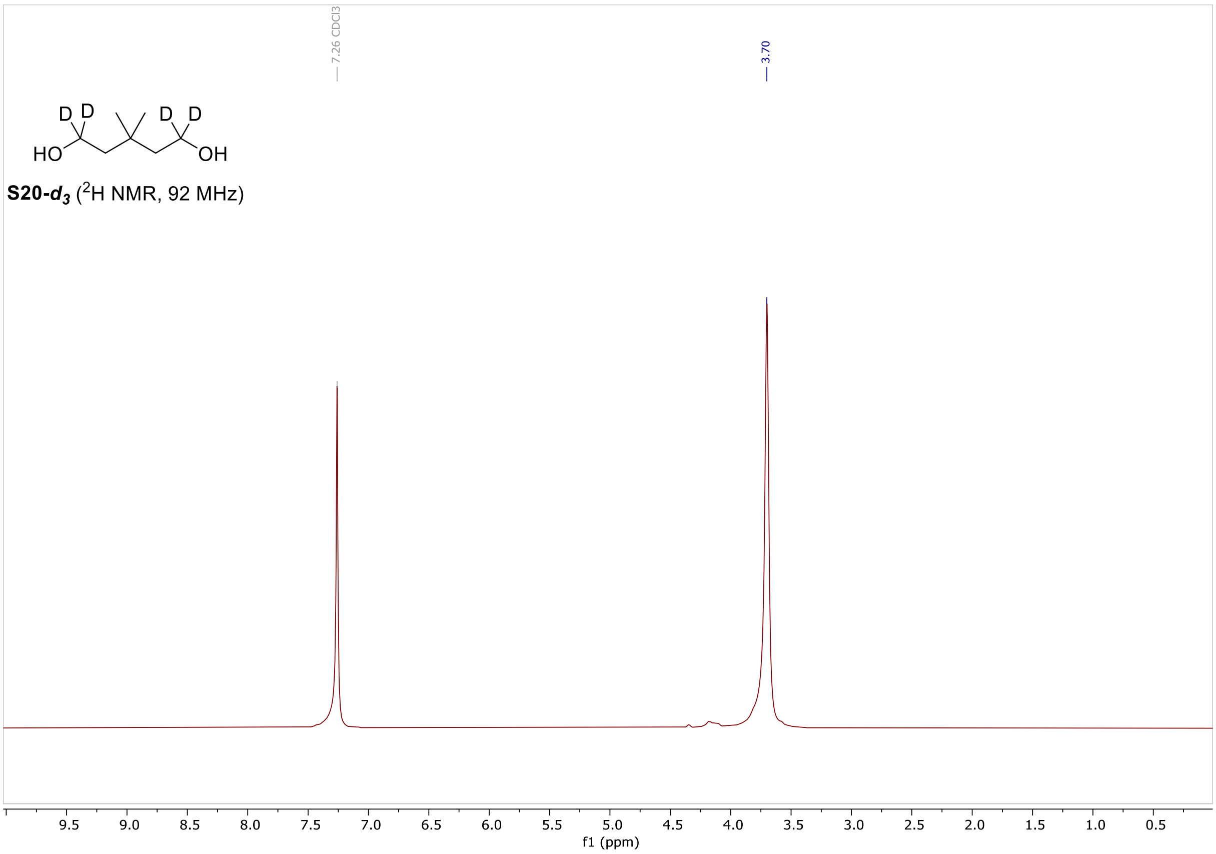
7.26 CDCl3
3.70
DD
DD
HO
OH
S20-d3 (2H NMR, 92 MHz)
9.5
9.0
8.5
8.0
7.5
7.0
6.5
6.0
5.5
5.0
4.5
4.0
3.5
3.0
2.5
2.0
1.5
1.0
0.5
f1 (ppm)

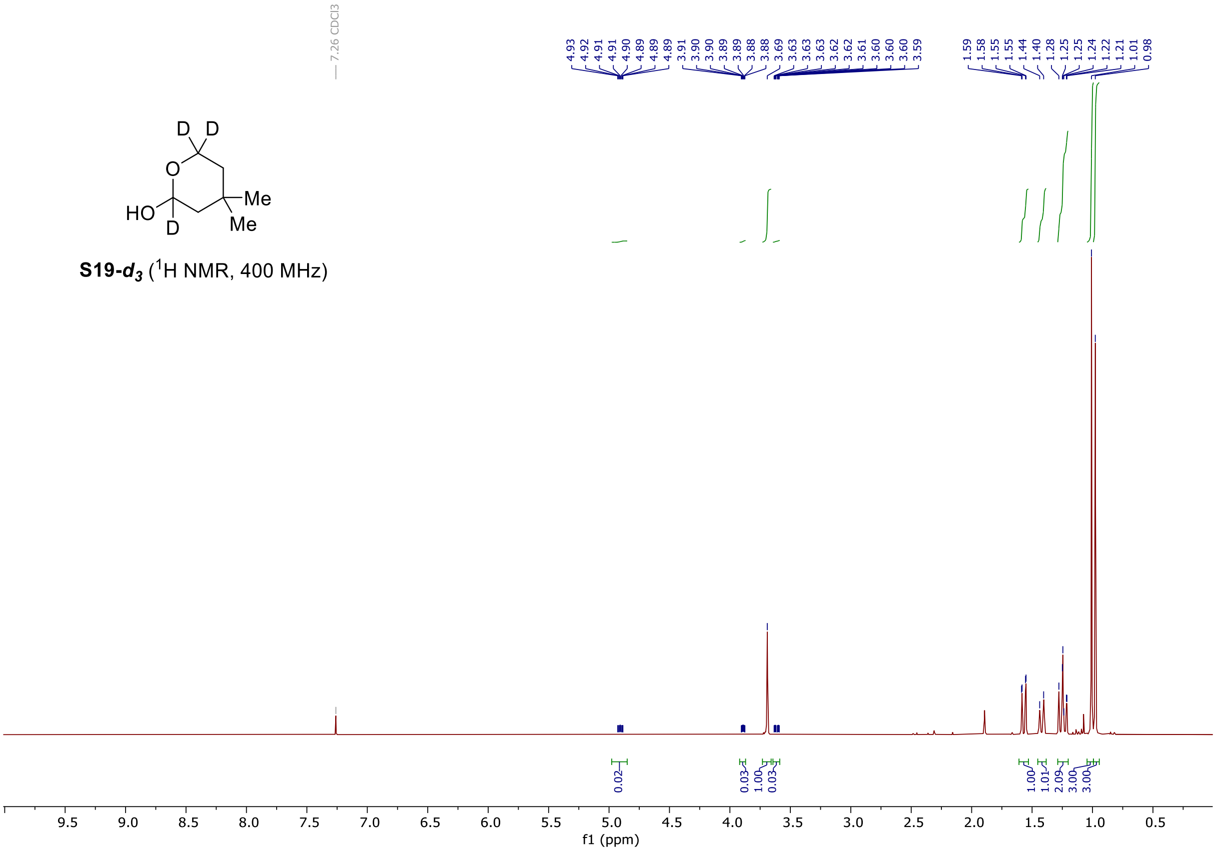

S19-d_3 (^{1}H NMR, 400 MHz)

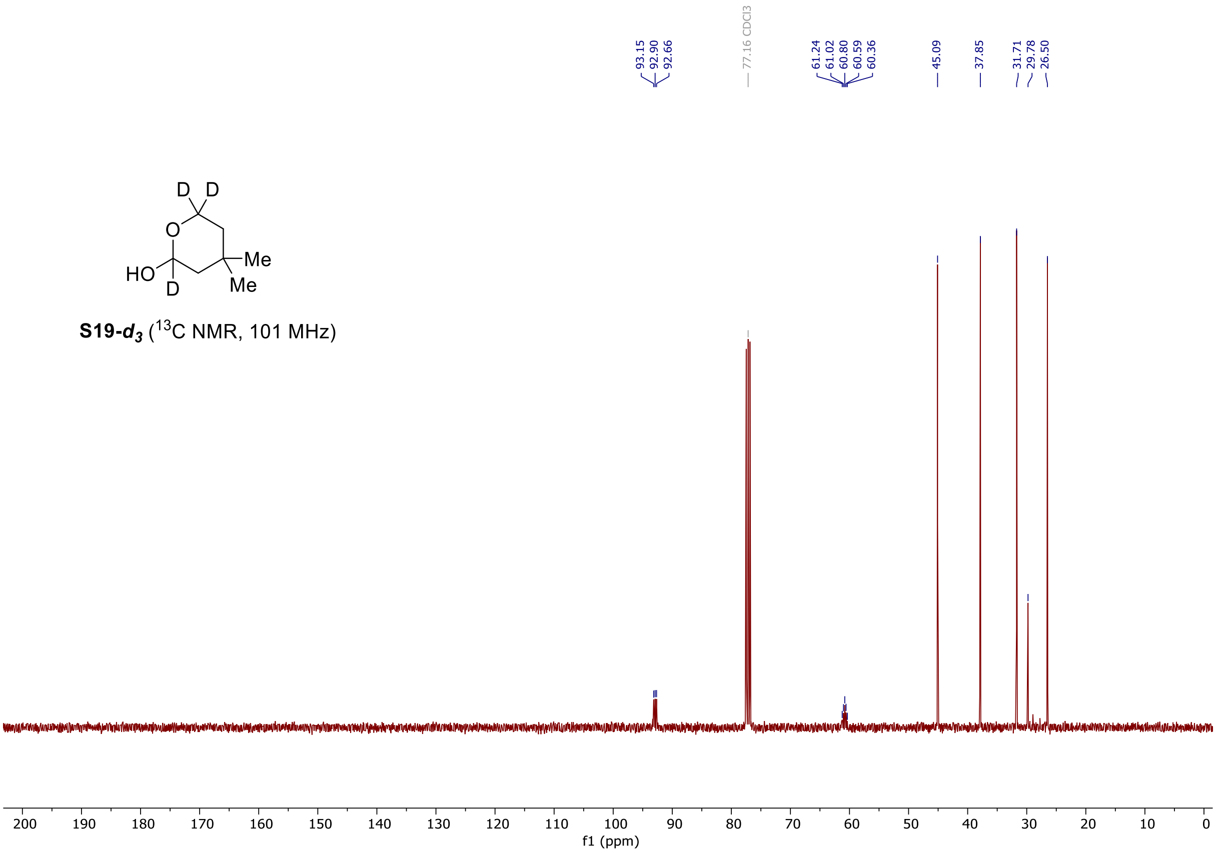

S19-d_3 (^{13}C NMR, 101 MHz)

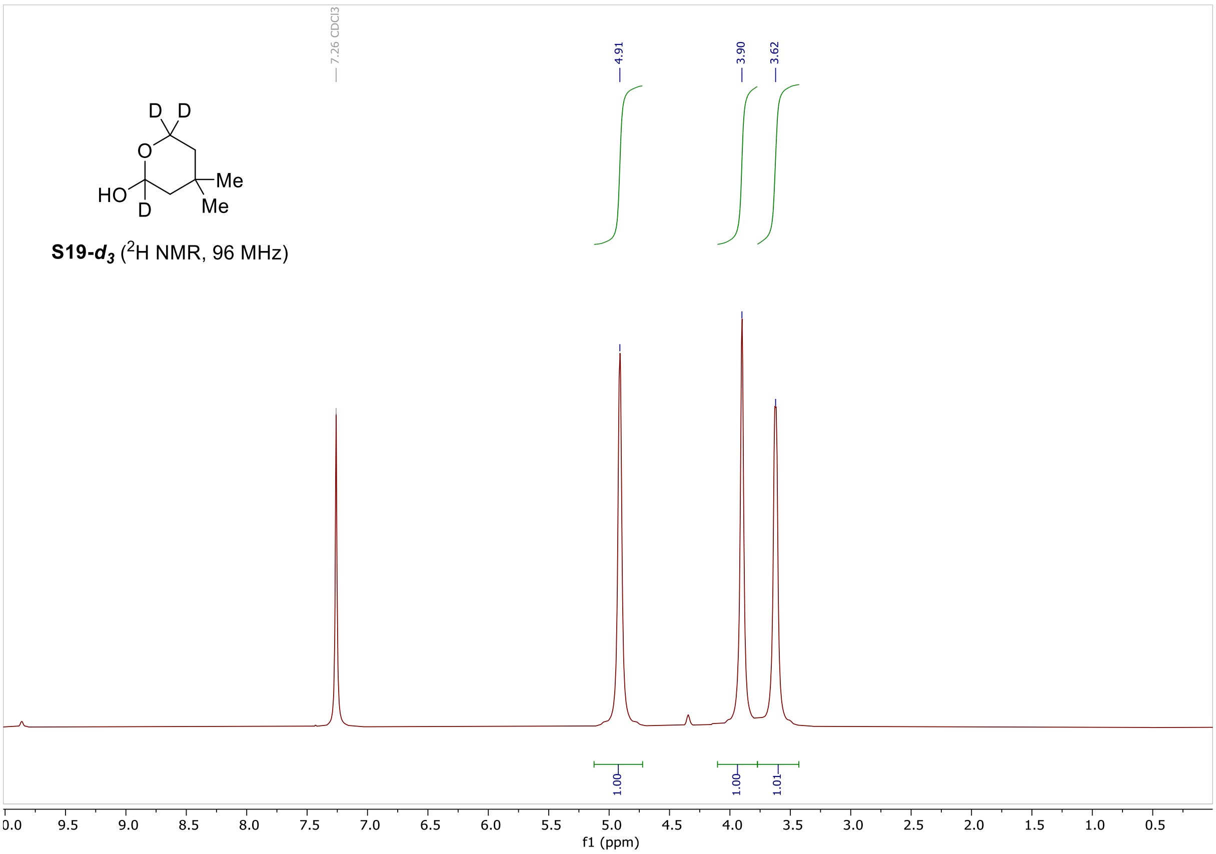

D D
O
HO
D
Me
Me
S19-d3 (2H NMR, 96 MHz)
7.26 CDCl3
4.91
3.90
3.62
1.00
1.00
1.01
f1 (ppm)

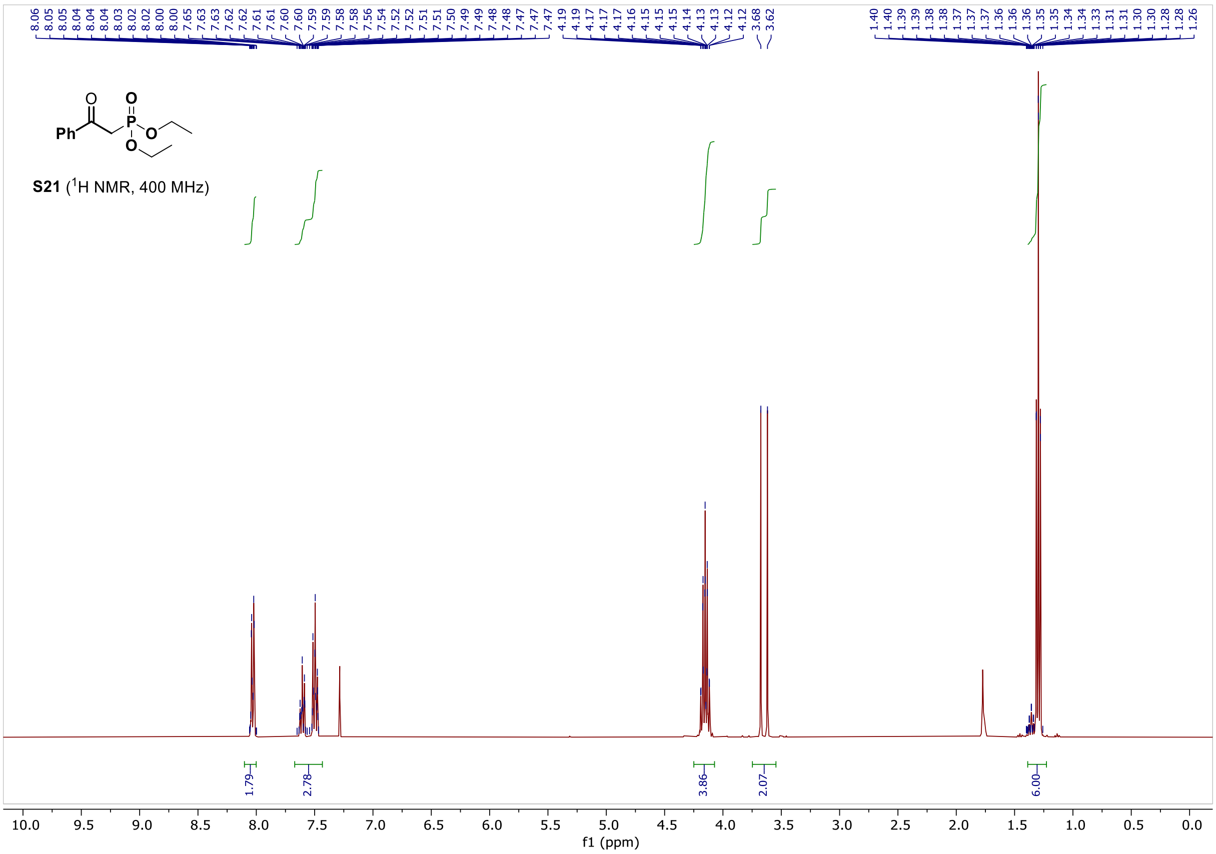
8.06 8.05 8.05 8.04 8.04 8.03 8.02 8.02 8.00 8.00 7.65 7.63 7.63 7.62 7.62 7.61 7.61 7.60 7.59 7.59 7.58 7.58 7.56 7.54 7.52 7.52 7.51 7.50 7.49 7.49 7.48 7.48 7.47 7.47 4.19 4.19 4.17 4.17 4.16 4.15 4.15 4.15 4.14 4.13 4.13 4.12 4.12 3.68 3.62
1.40 1.40 1.39 1.39 1.38 1.38 1.37 1.37 1.36 1.36 1.36 1.35 1.35 1.34 1.34 1.33 1.31 1.31 1.30 1.30 1.28 1.26
Ph
O
P
S21 (1H NMR, 400 MHz)
1.79
2.78
3.86
2.07
6.00
f1 (ppm)
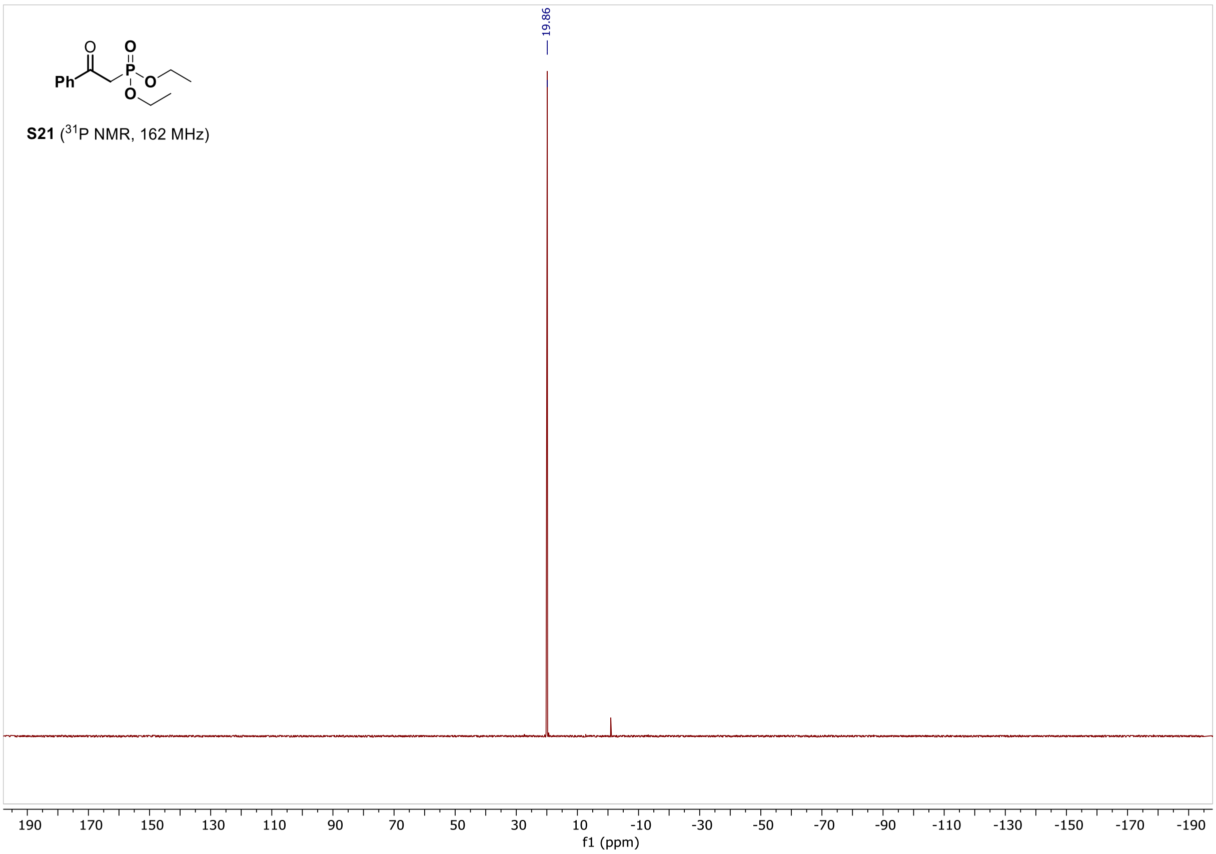
19.86
Ph
O
P
S21 (31P NMR, 162 MHz)
f1 (ppm)

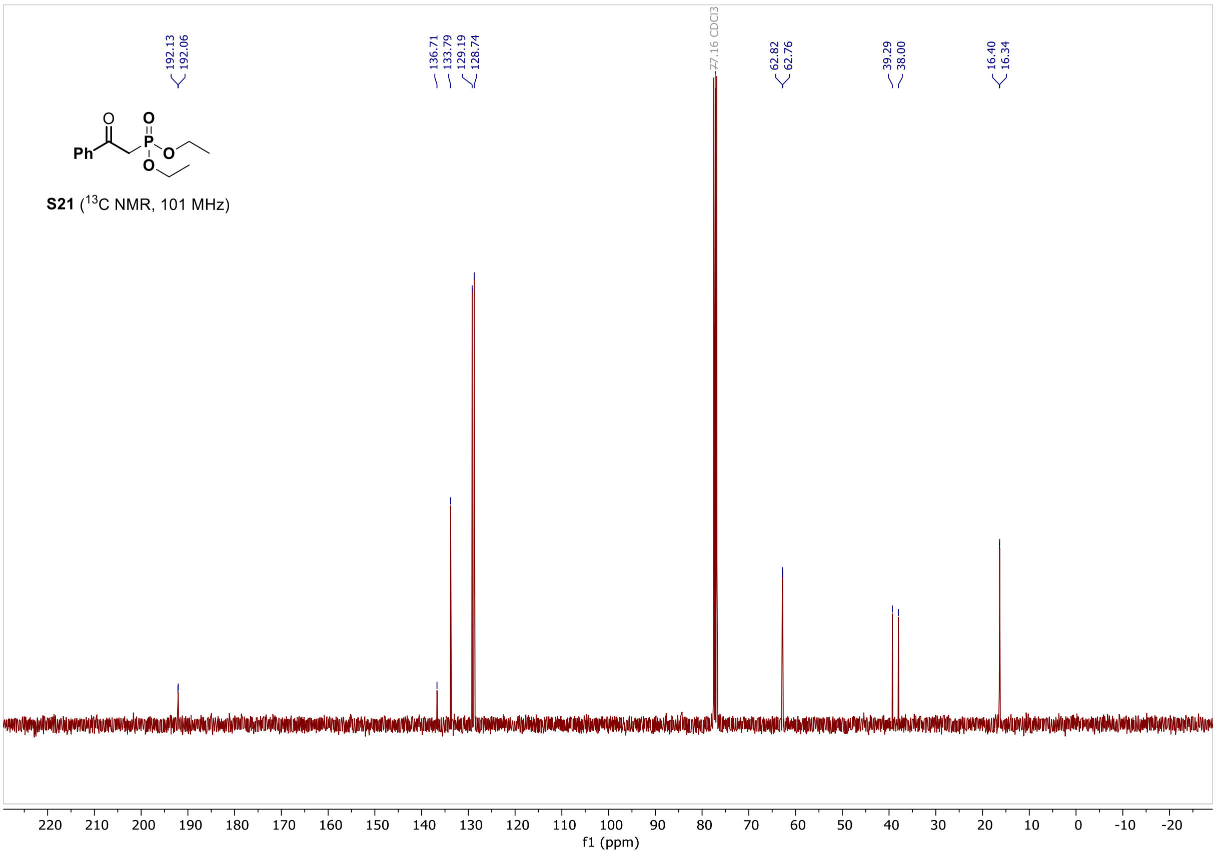

S21 (^{13}C NMR, 101 MHz)

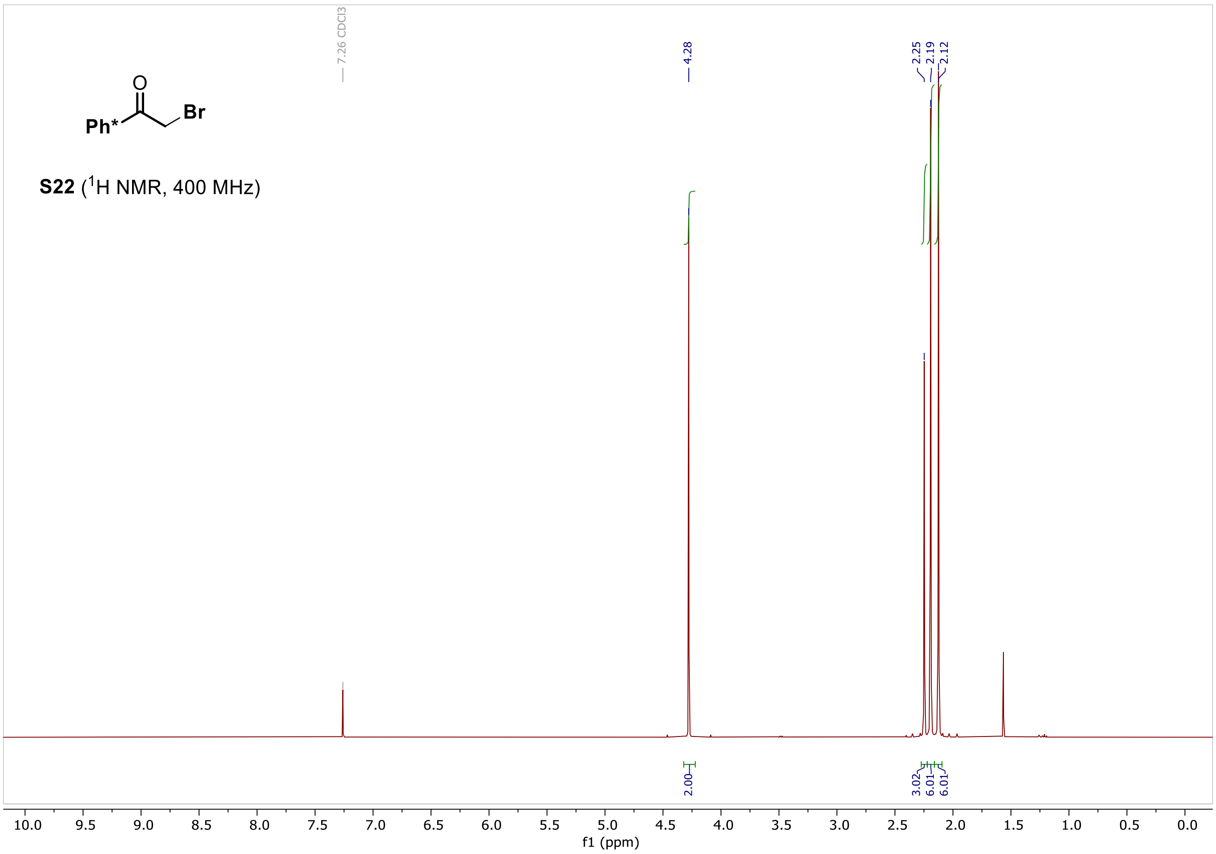

Ph*
O
Br
S22 (^{1}H NMR, 400 MHz)
7.26 CDCl3
4.28
2.25
2.19
2.12
2.00
3.02
6.01
6.01
f1 (ppm)

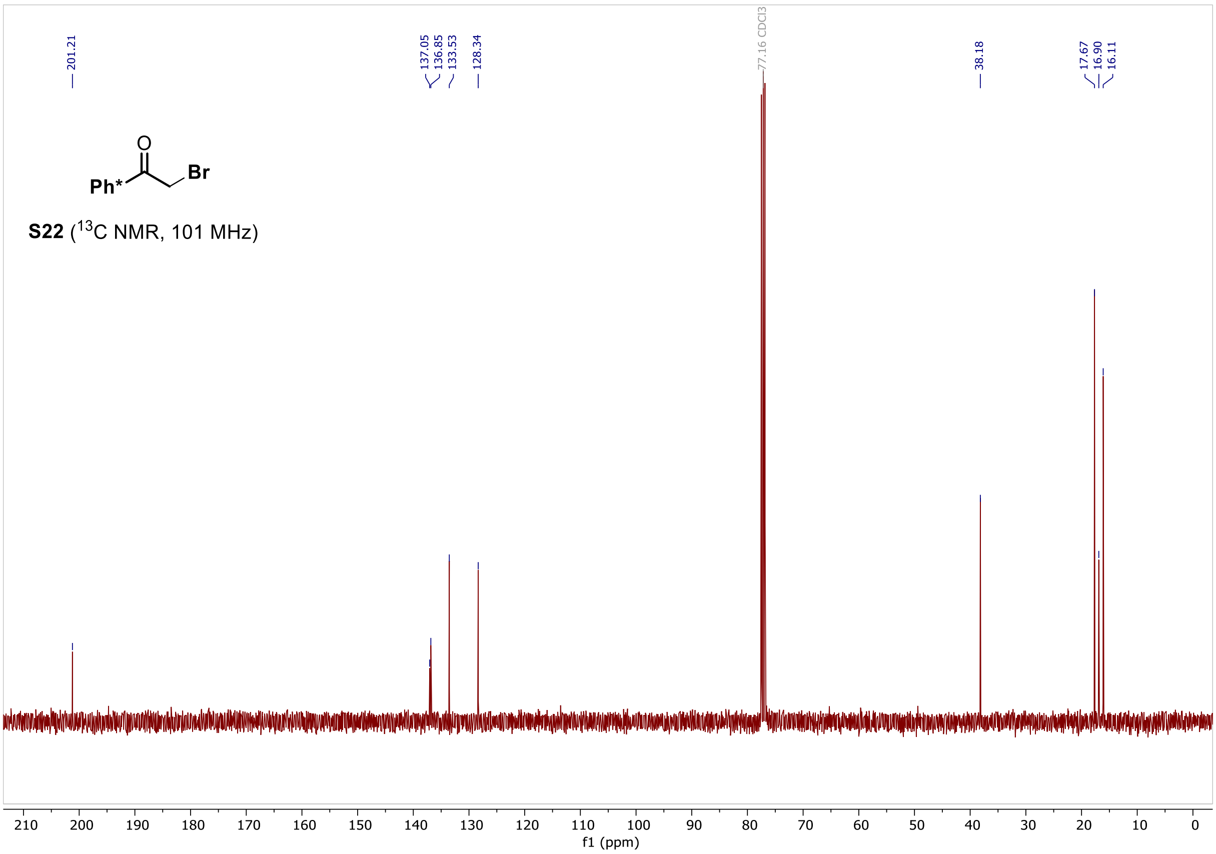

Ph*
O
Br
S22 (^{13}C NMR, 101 MHz)
201.21
137.05
136.85
133.53
128.34
77.16 CDCl3
38.18
17.67
16.90
16.11
f1 (ppm)

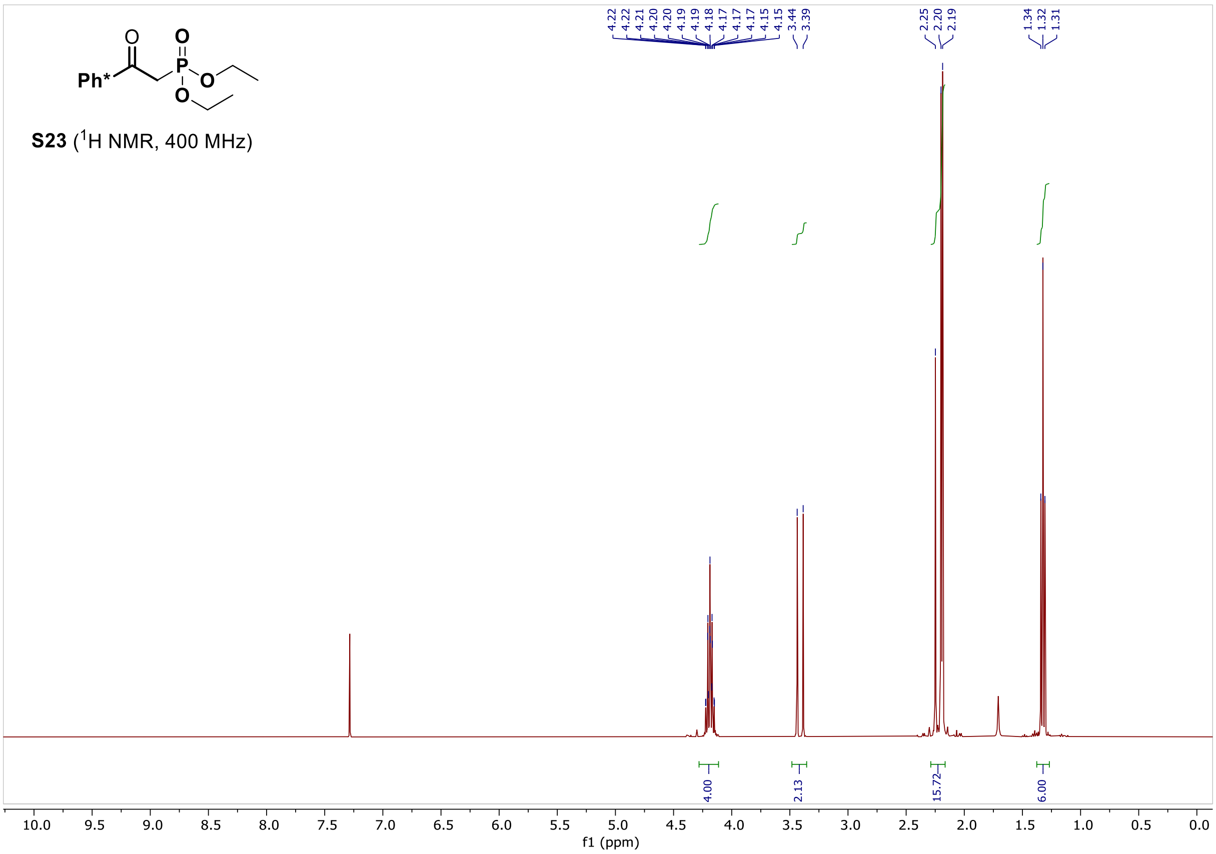
Ph*
S23 (1H NMR, 400 MHz)
4.22 4.22 4.21 4.20 4.20 4.19 4.19 4.18 4.17 4.17 4.17 4.15 4.15 3.44 3.39
2.25 2.20 2.19
1.34 1.32 1.31
4.00
2.13
15.72
6.00
f1 (ppm)

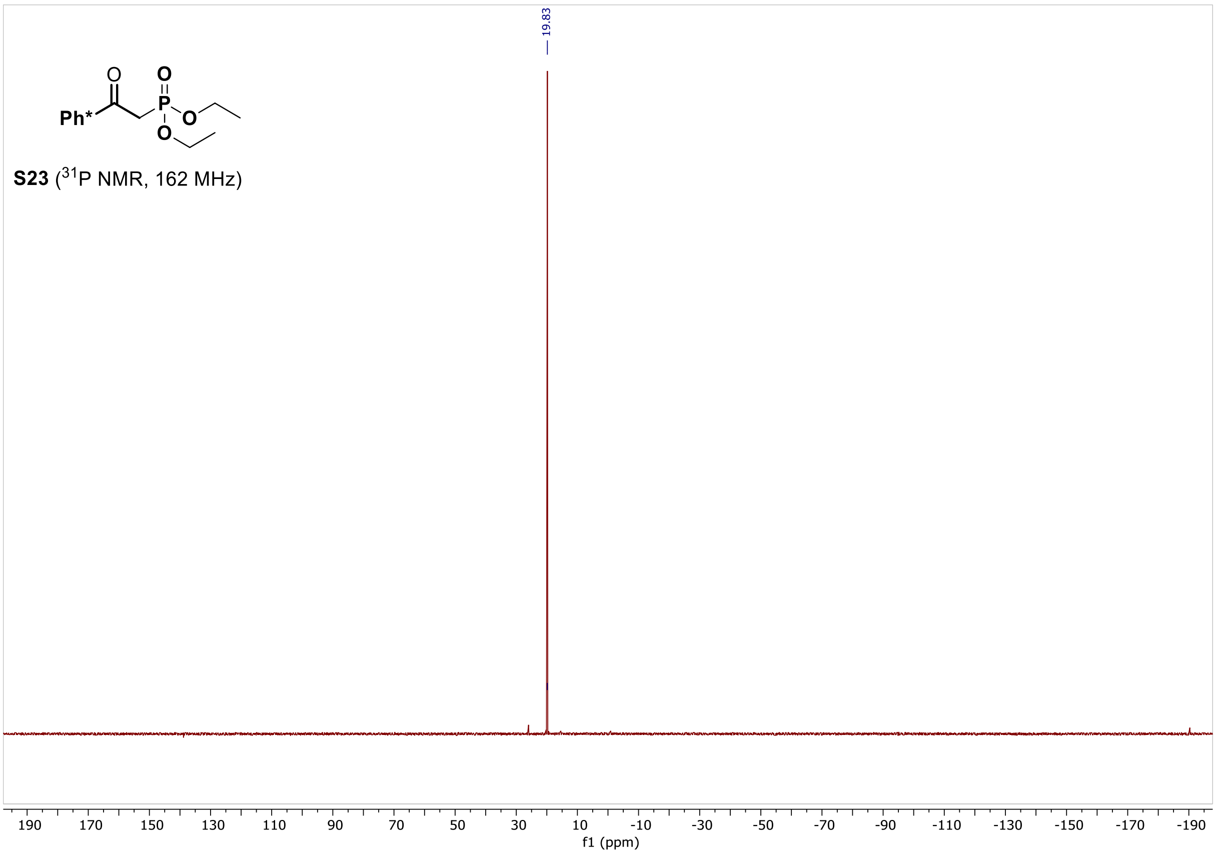
Ph*
S23 (31P NMR, 162 MHz)
19.83
f1 (ppm)

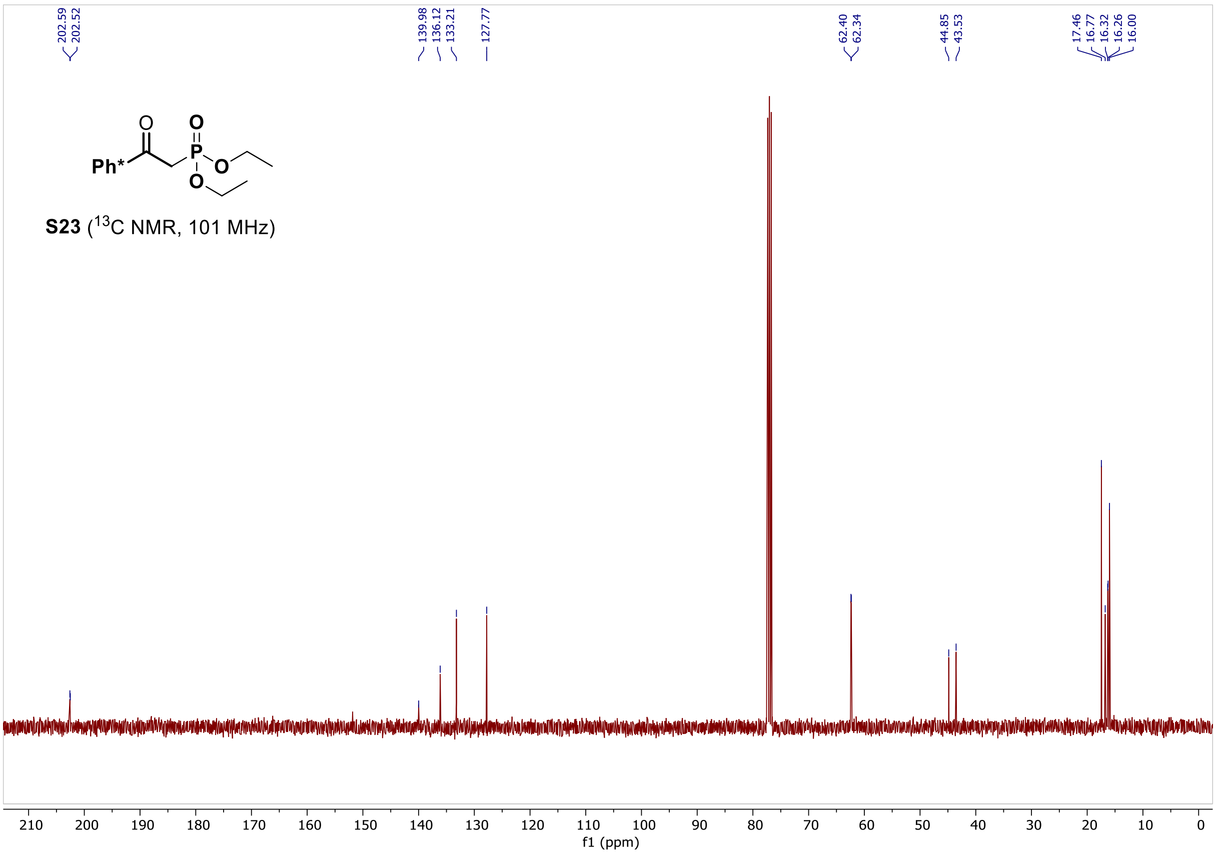
202.59
202.52
139.98
136.12
133.21
127.77
62.40
62.34
44.85
43.53
17.46
16.77
16.32
16.26
16.00
Ph*
S23 (^{13}C NMR, 101 MHz)
f1 (ppm)

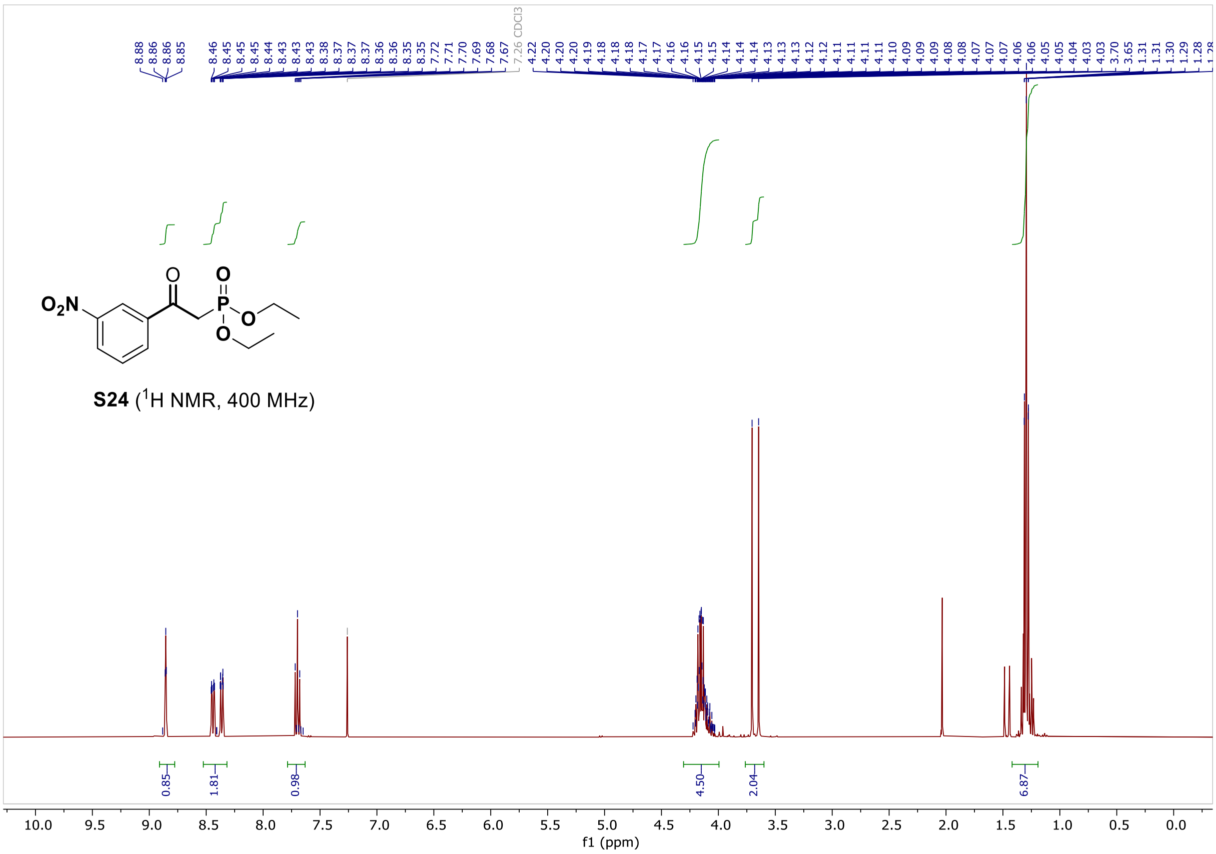

S24 (^{1}H NMR, 400 MHz)

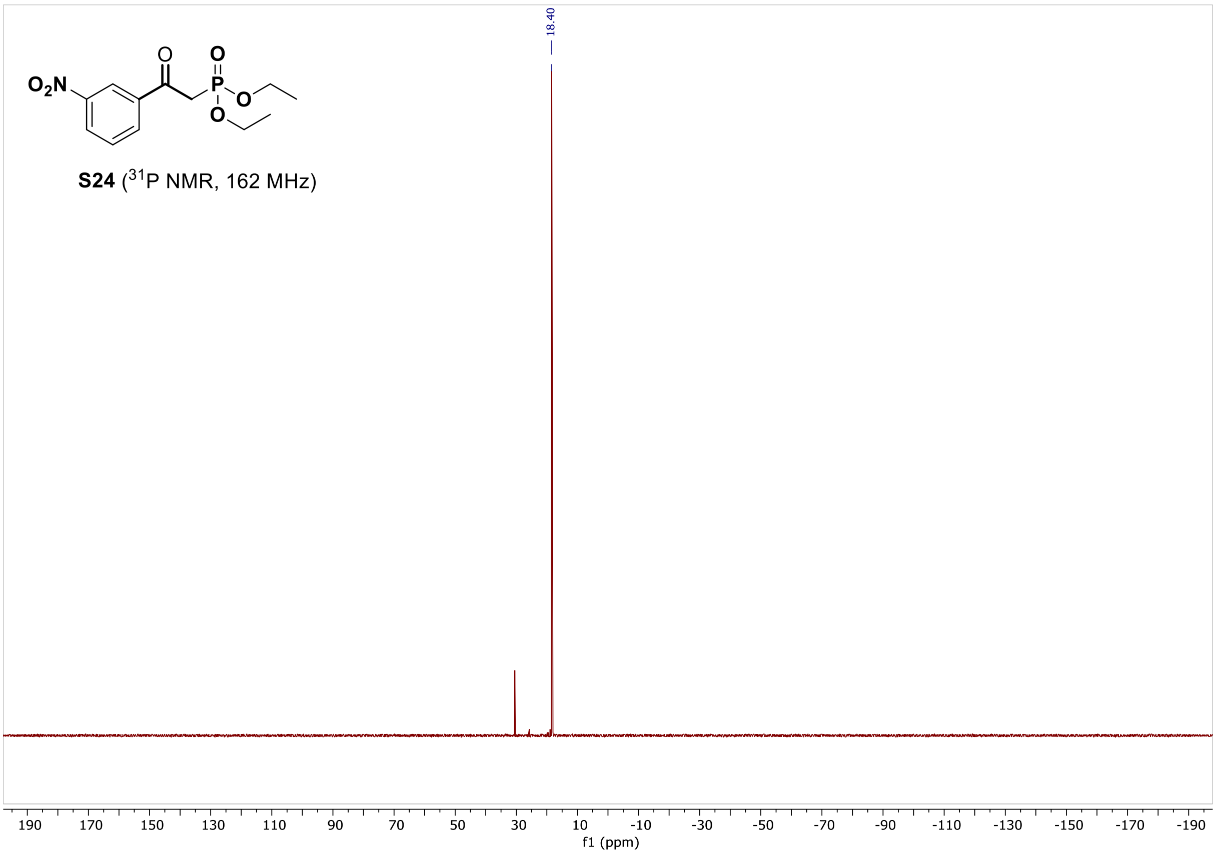

S24 (^{31}P NMR, 162 MHz)

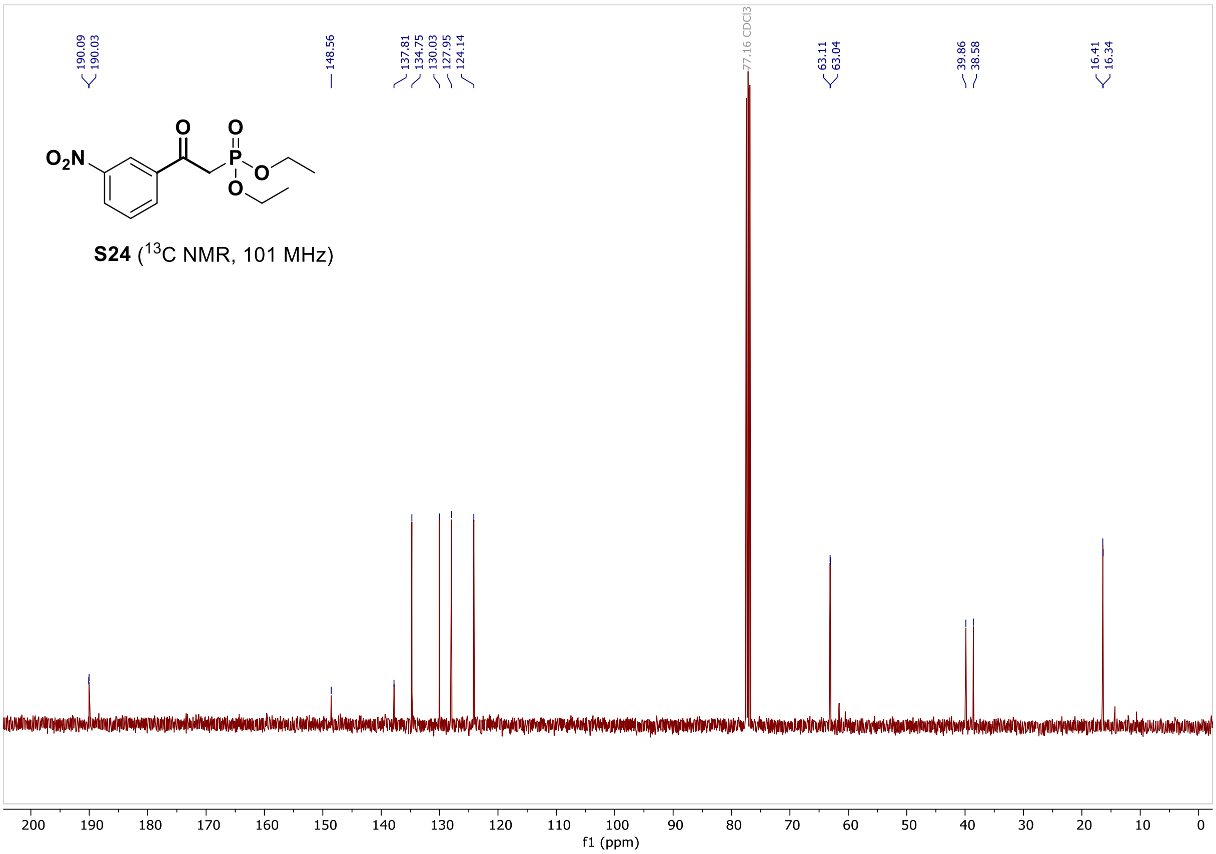

190.09
190.03
148.56
137.81
134.75
130.03
127.95
124.14
77.16 CDCl3
63.11
63.04
39.86
38.58
16.41
16.34
O2N
O
O
P
O
O
S24 (13C NMR, 101 MHz)
200
190
180
170
160
150
140
130
120
110
100
90
80
70
60
50
40
30
20
10
0
f1 (ppm)

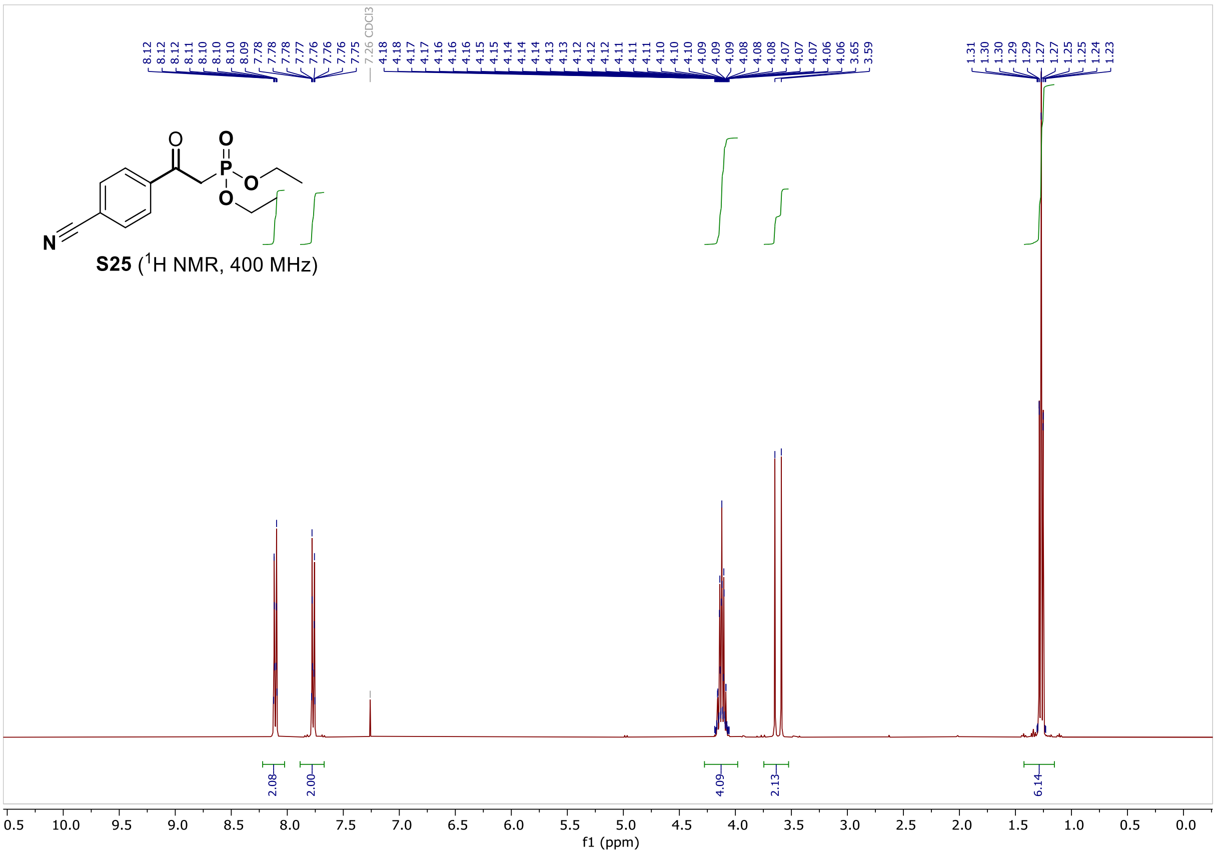

S25 (^{1}H NMR, 400 MHz)

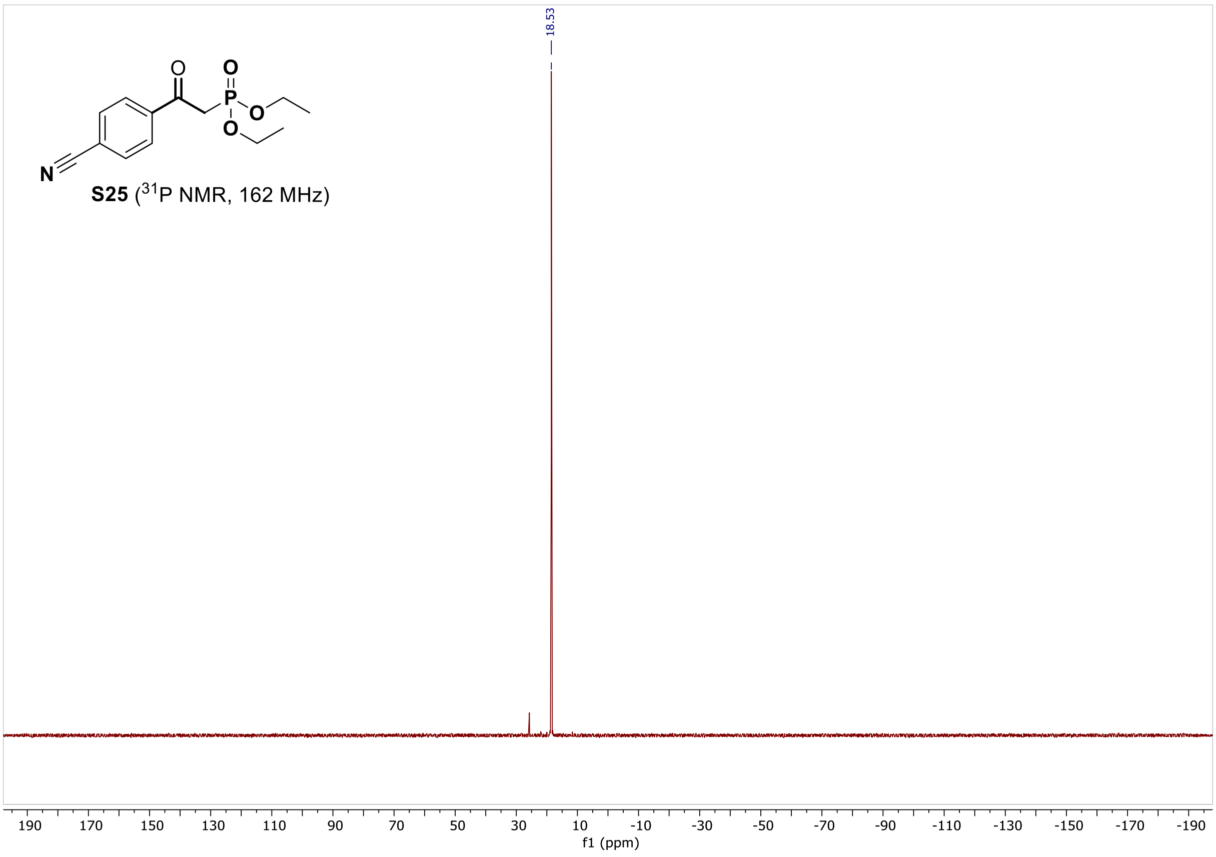

S25 (^{31}P NMR, 162 MHz)

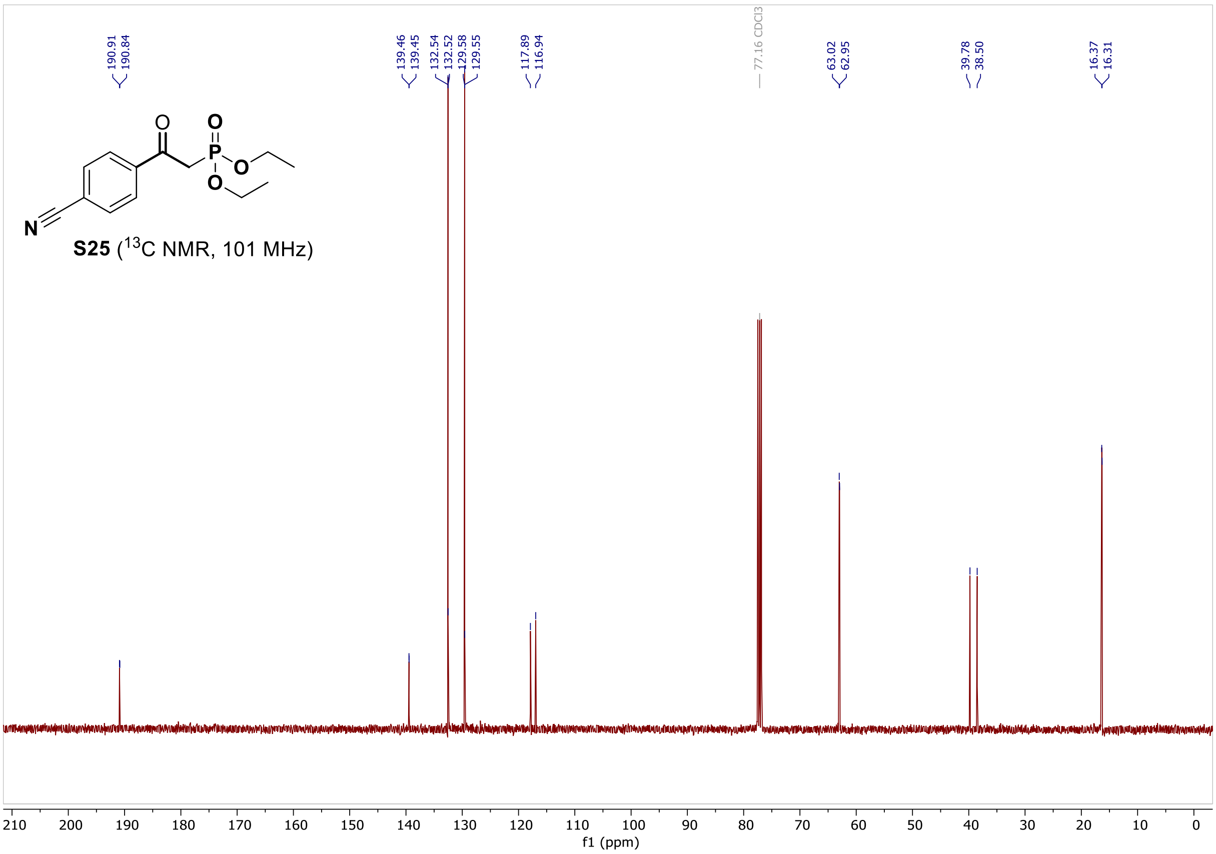
190.91
190.84
139.46
139.45
132.54
132.52
129.58
129.55
117.89
116.94
77.16 CDCl3
63.02
62.95
39.78
38.50
16.37
16.31
S25 (^{13}C NMR, 101 MHz)
f1 (ppm)

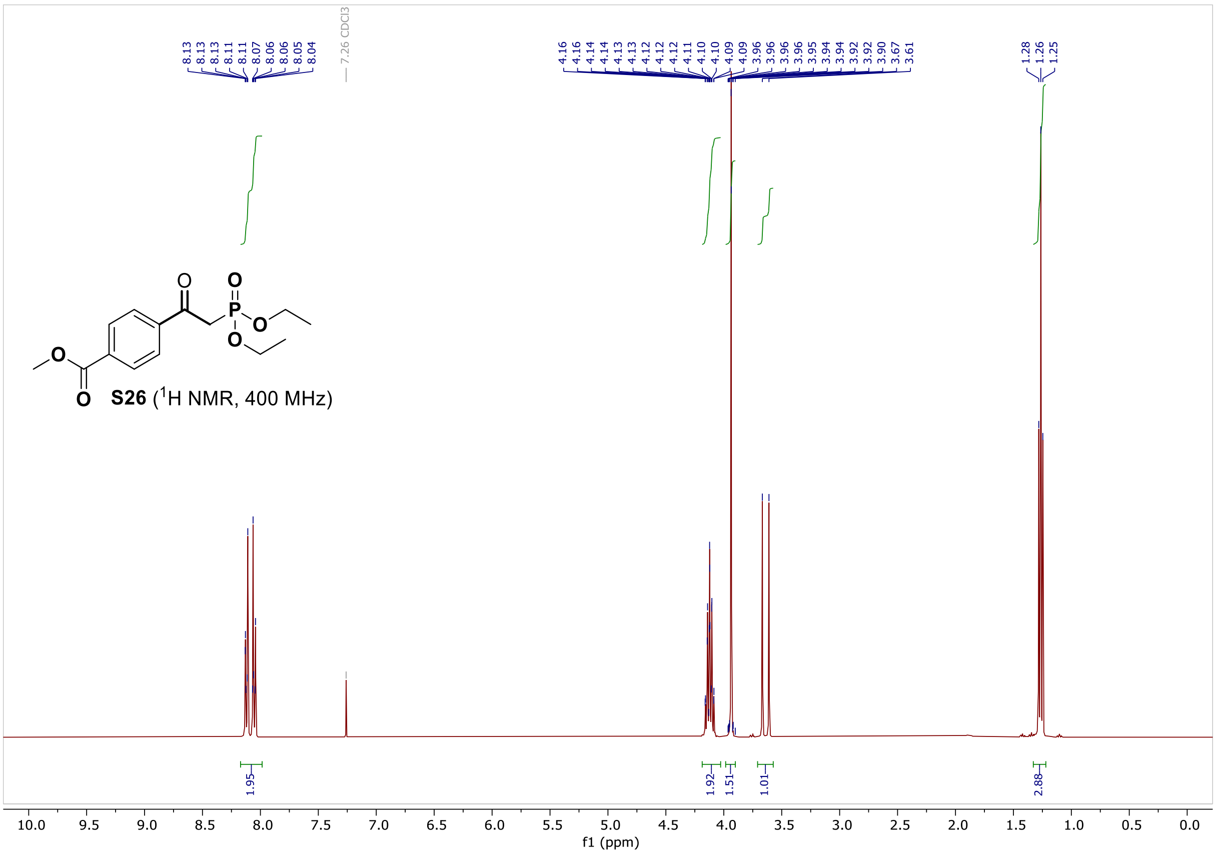
S26 (^{1}H NMR, 400 MHz)
f1 (ppm)

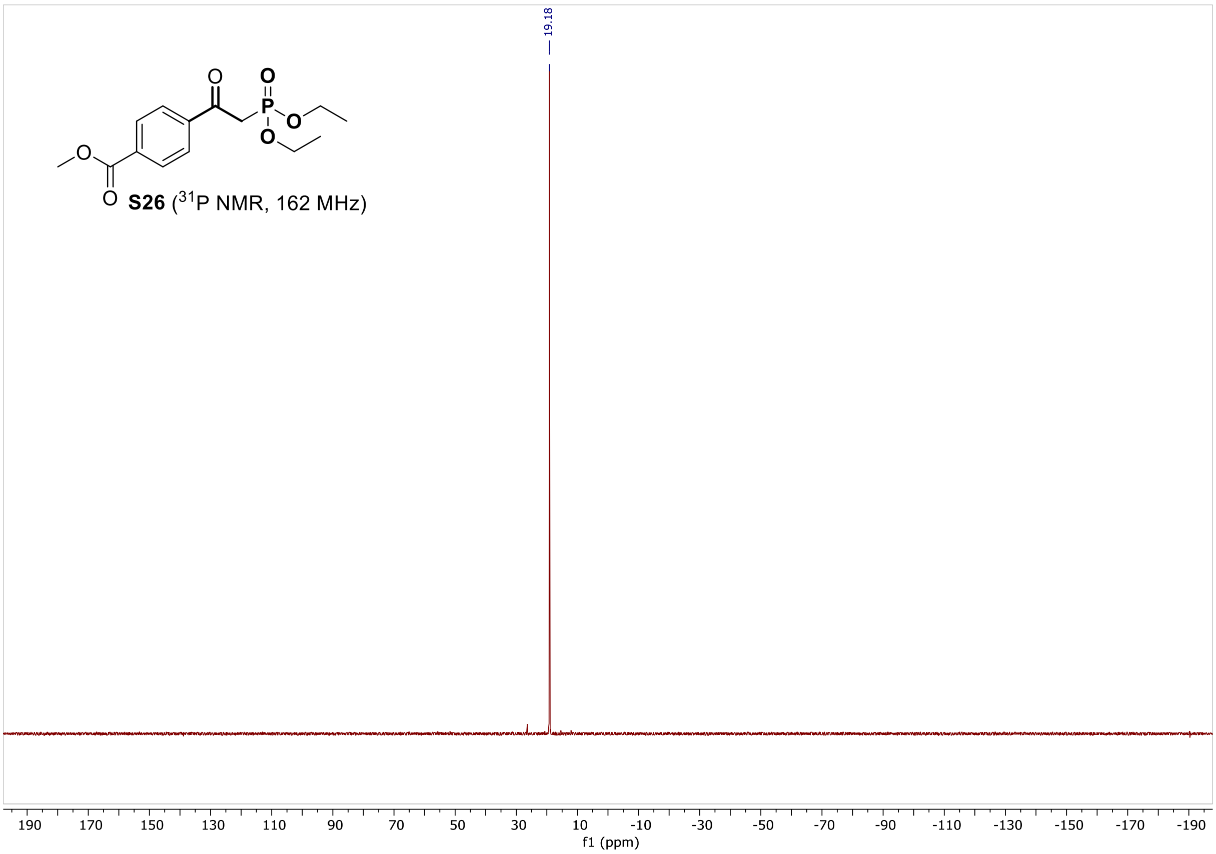
S26 (^{31}P NMR, 162 MHz)
19.18
f1 (ppm)

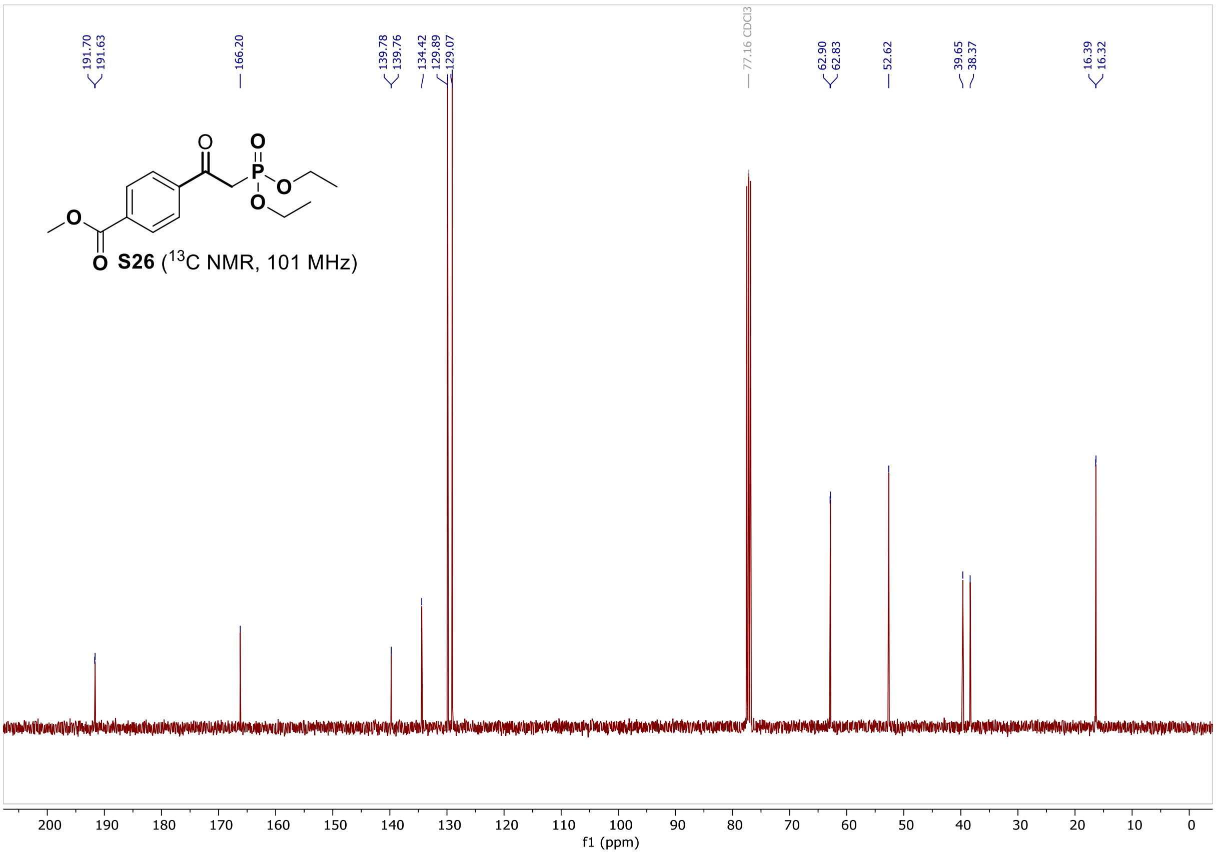

191.70
191.63
166.20
139.78
139.76
134.42
129.89
129.07
77.16 CDCl3
62.90
62.83
52.62
39.65
38.37
16.39
16.32
S26 (^{13}C NMR, 101 MHz)
f1 (ppm)

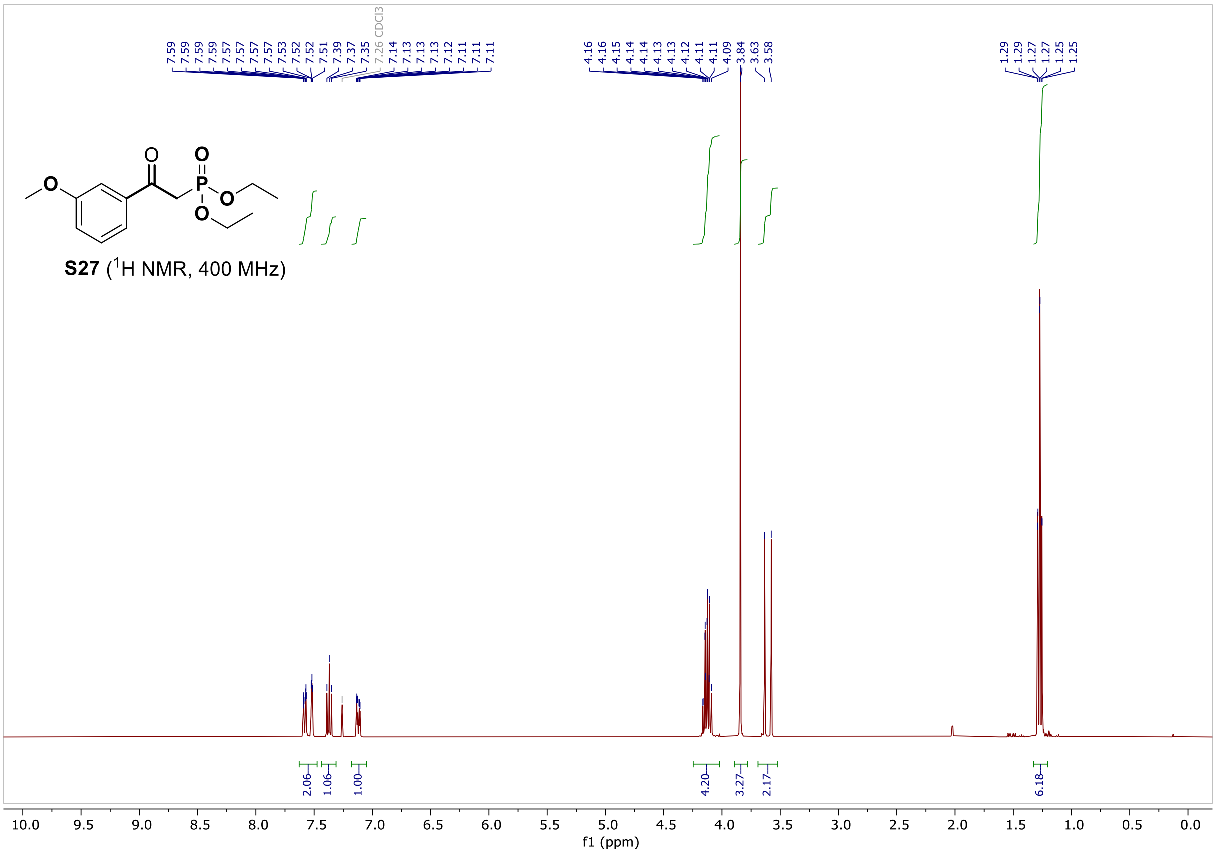

S27 (^{1}H NMR, 400 MHz)

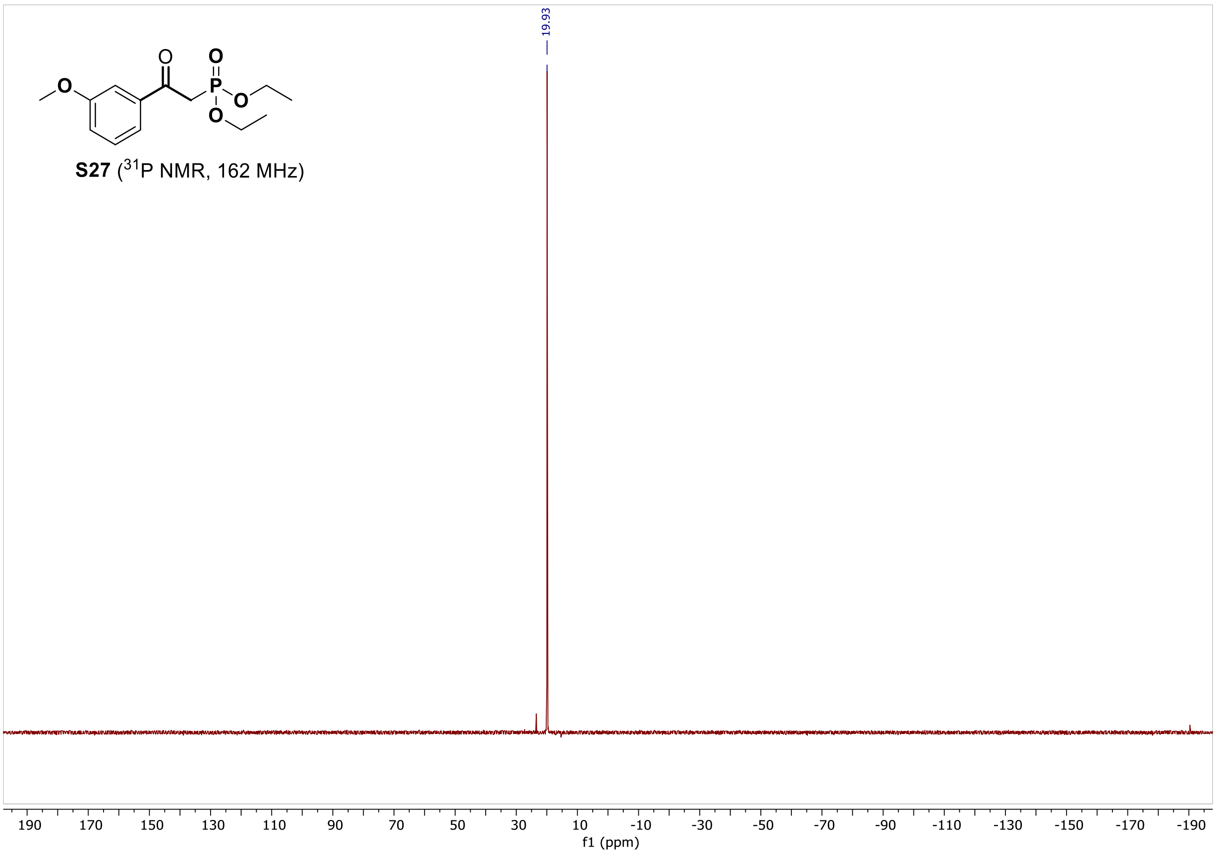

S27 (^{31}P NMR, 162 MHz)

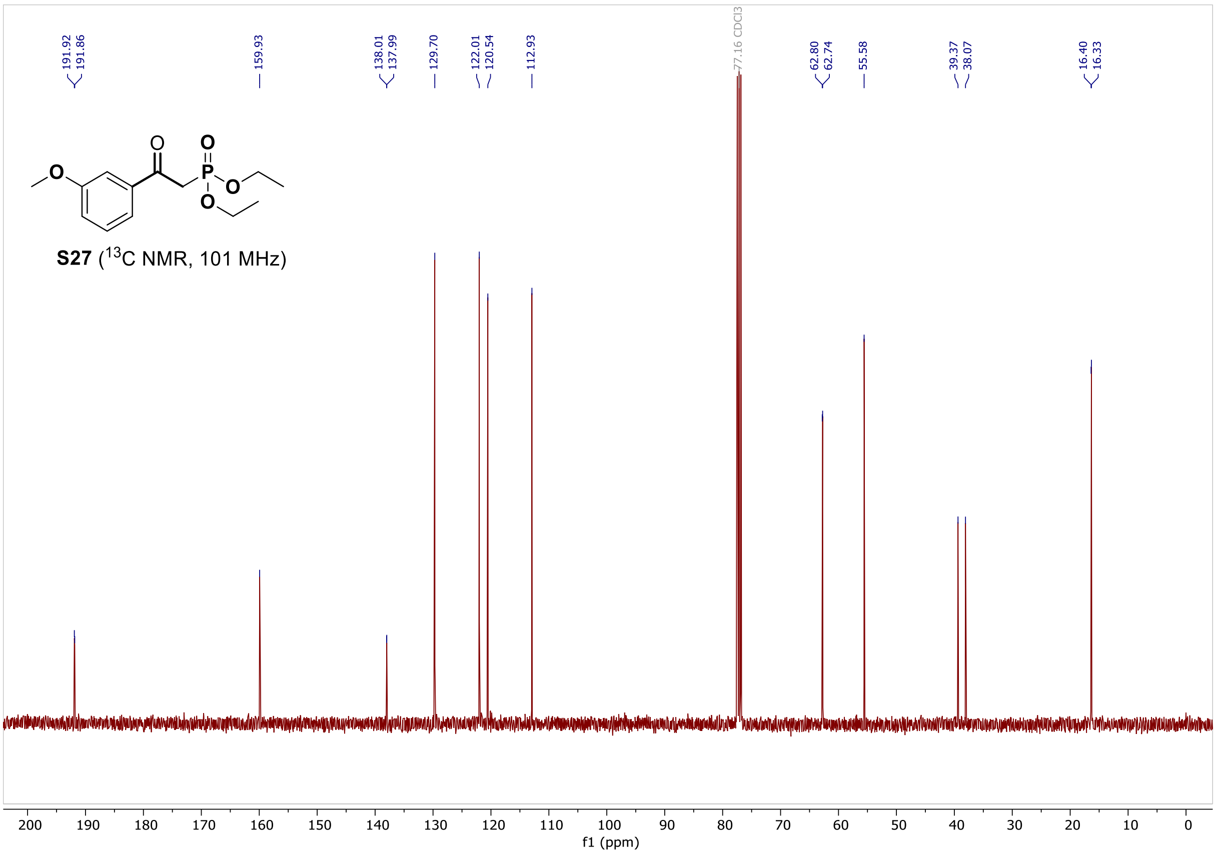

191.92
191.86
159.93
138.01
137.99
129.70
122.01
120.54
112.93
77.16 CDCl3
62.80
62.74
55.58
39.37
38.07
16.40
16.33
S27 (13C NMR, 101 MHz)
f1 (ppm)

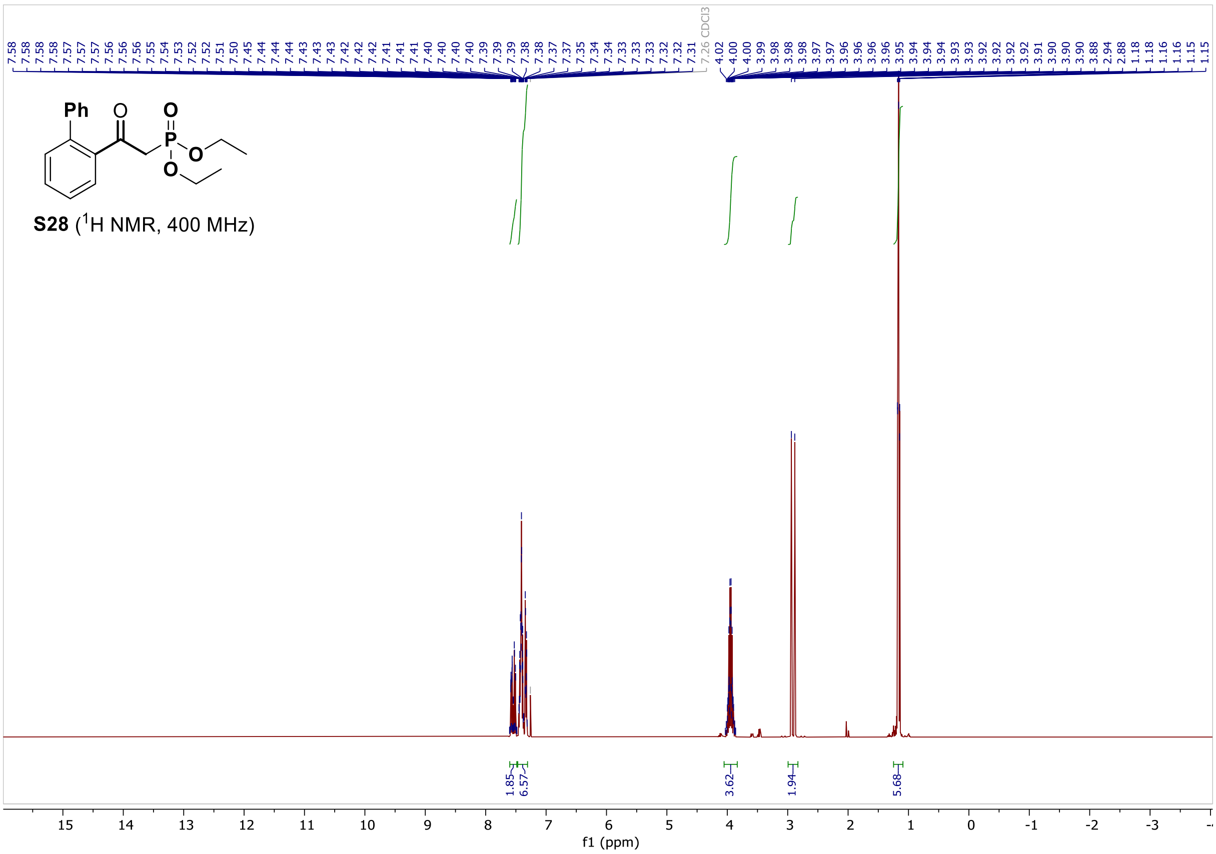
Ph
S28 (1H NMR, 400 MHz)
7.26 CDCl3
1.85
6.57
3.62
1.94
5.68
f1 (ppm)

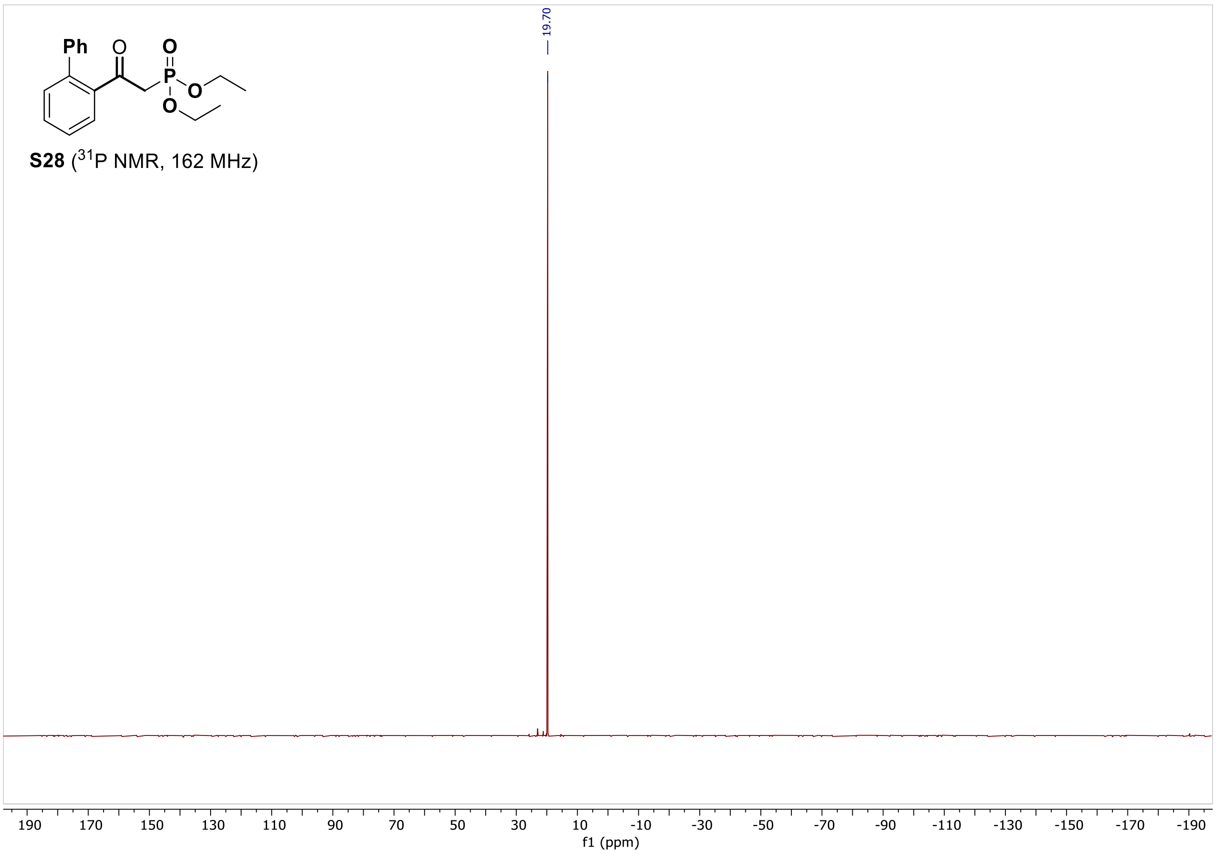
Ph
S28 (31P NMR, 162 MHz)
19.70
f1 (ppm)

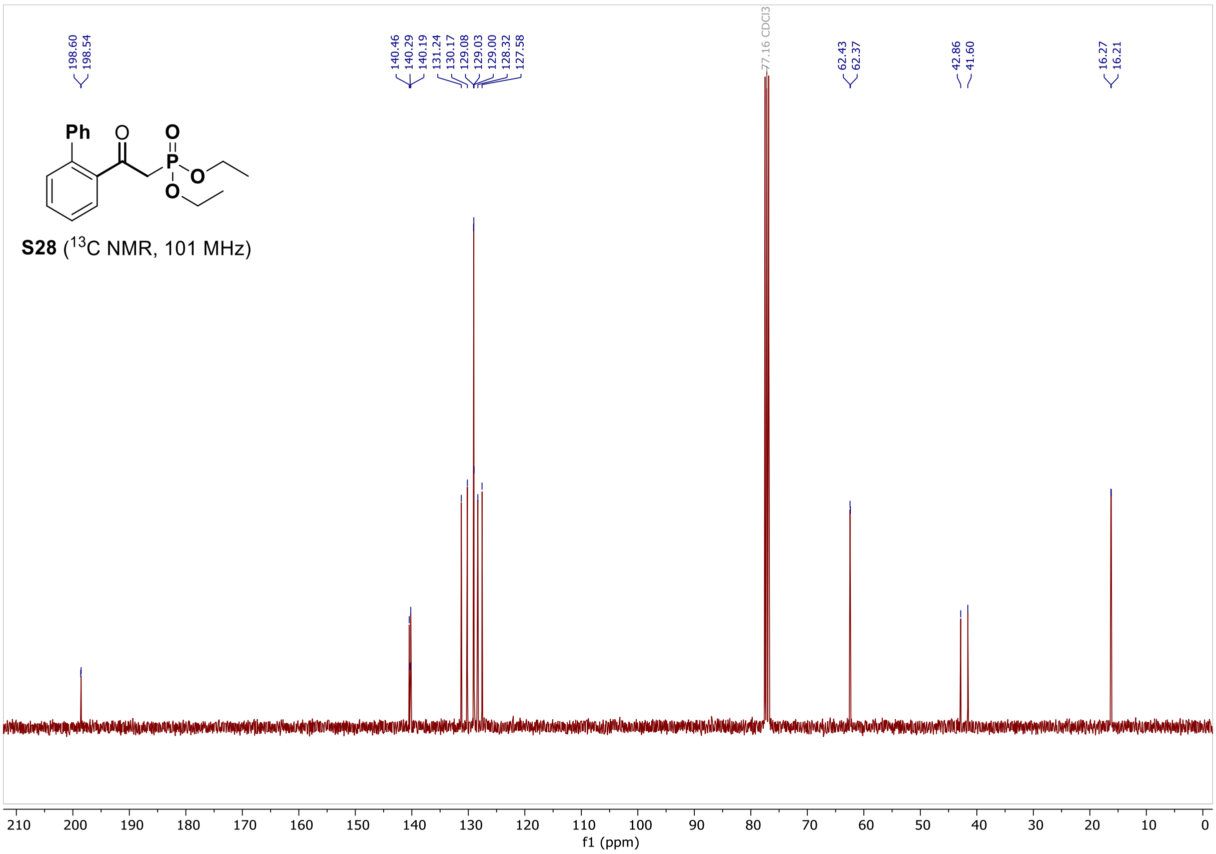
198.60
198.54
140.46
140.29
140.19
131.24
130.17
129.08
129.03
129.00
128.32
127.58
77.16 CDCl3
62.43
62.37
42.86
41.60
16.27
16.21
Ph
O
O
P
O
O
S28 (13C NMR, 101 MHz)
210 200 190 180 170 160 150 140 130 120 110 100 90 80 70 60 50 40 30 20 10 0
f1 (ppm)

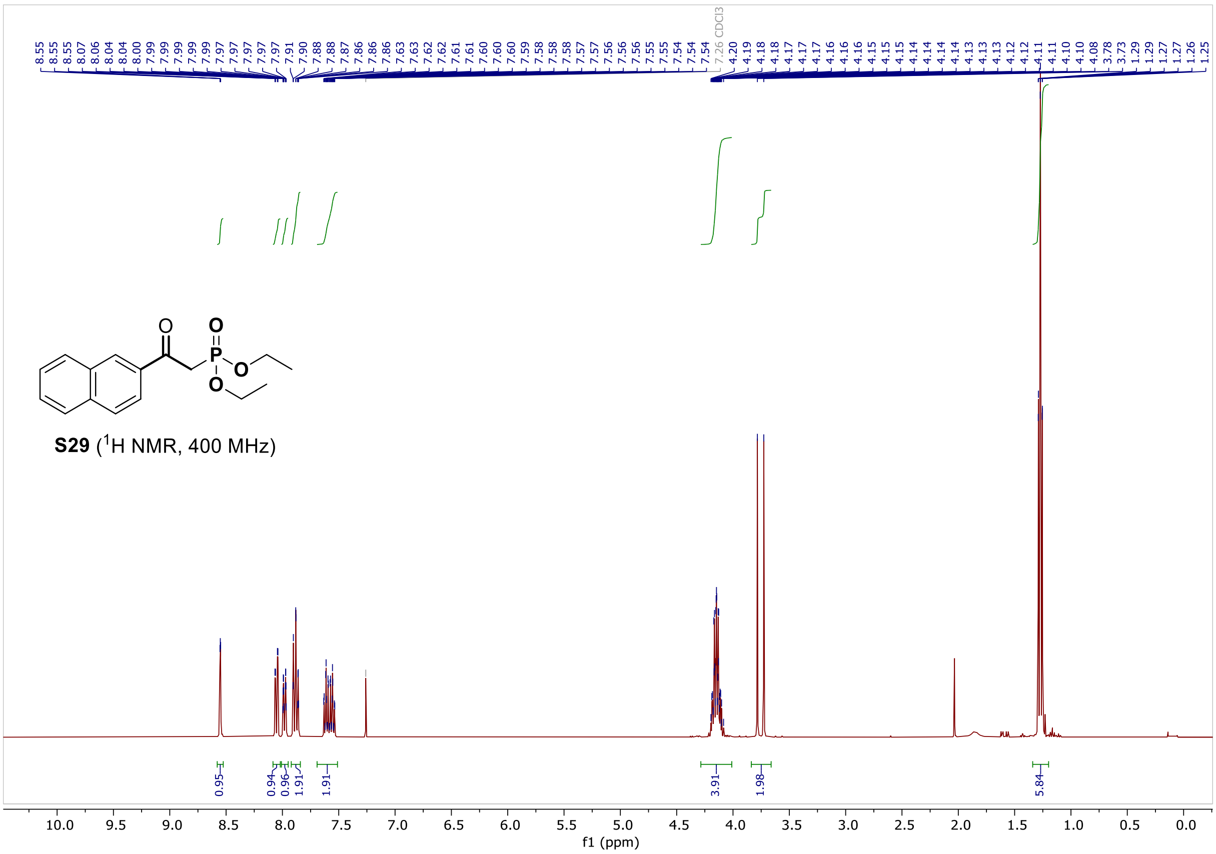

S29 (^{1}H NMR, 400 MHz)

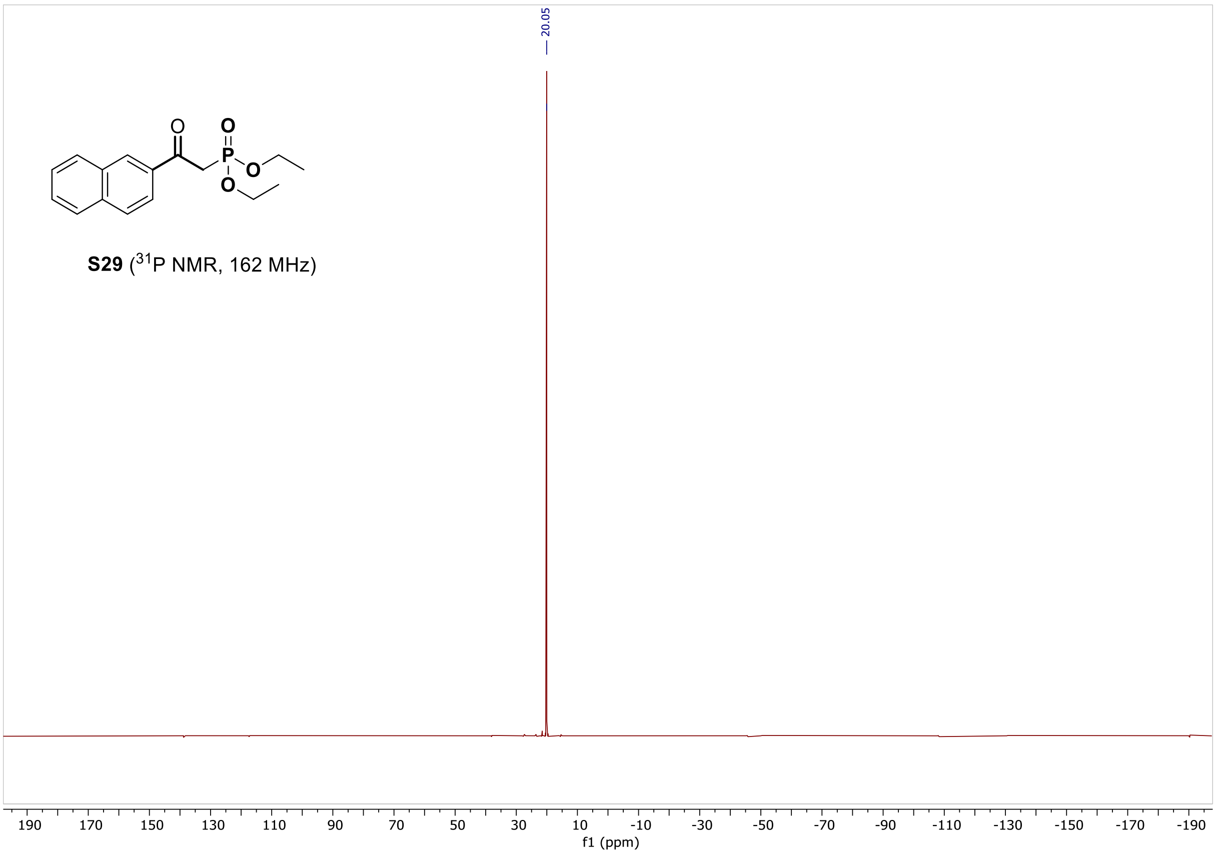

S29 (^{31}P NMR, 162 MHz)

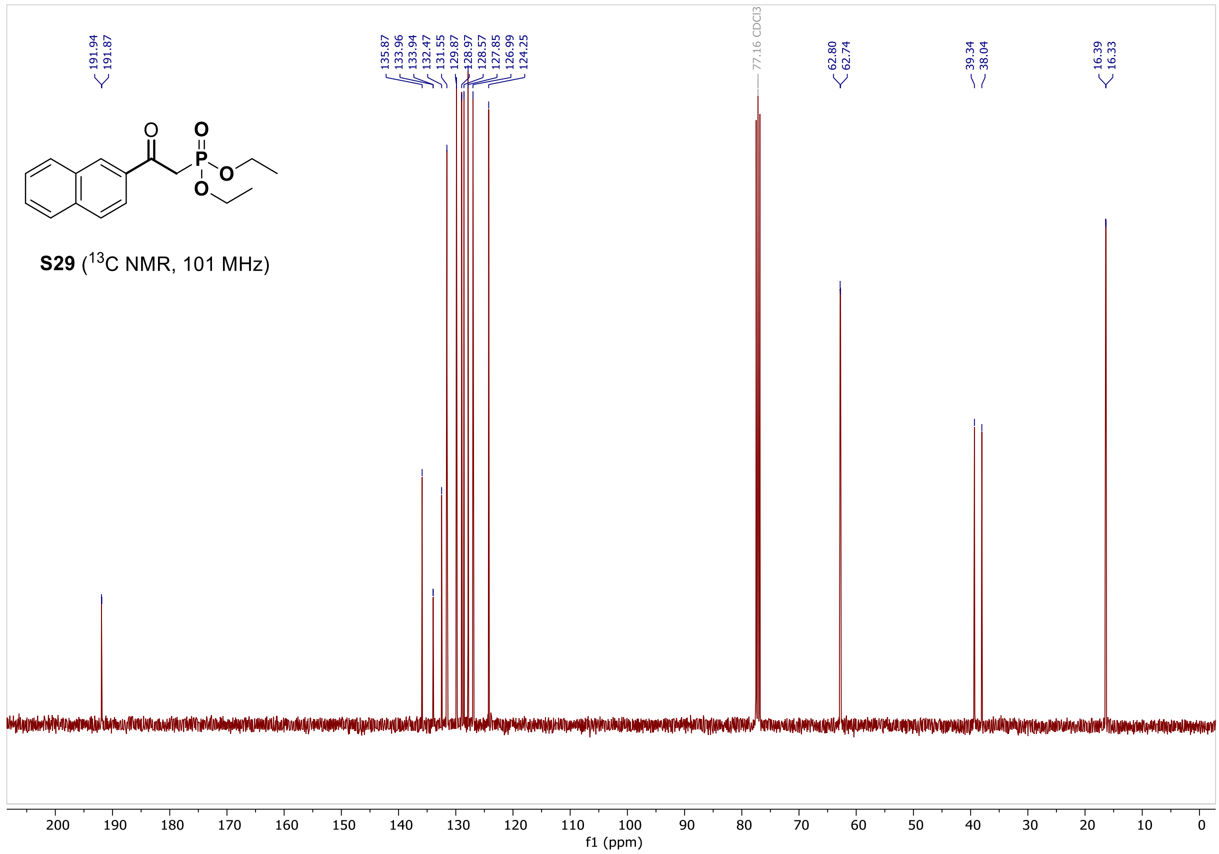
191.94
191.87
135.87
133.96
133.94
132.47
131.55
129.87
128.97
128.57
127.85
126.99
124.25
77.16 CDCl3
62.80
62.74
39.34
38.04
16.39
16.33
S29 (^{13}C NMR, 101 MHz)
f1 (ppm)

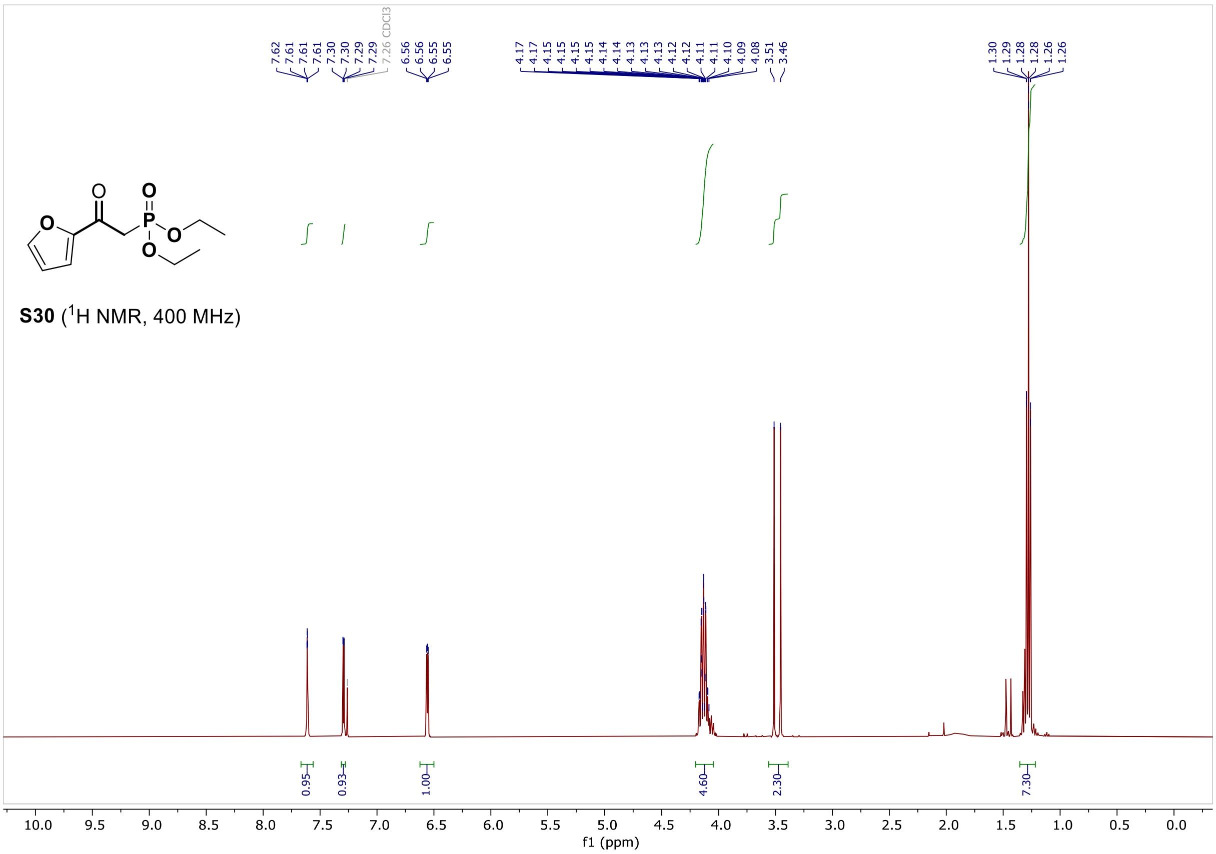
7.62
7.61
7.61
7.61
7.30
7.30
7.29
7.29
7.26 CDCl3
6.56
6.56
6.55
6.55
4.17
4.17
4.15
4.15
4.15
4.14
4.13
4.13
4.13
4.12
4.12
4.11
4.11
4.10
4.09
4.08
3.51
3.46
1.30
1.29
1.28
1.28
1.26
1.26
S30 (1H NMR, 400 MHz)
0.95
0.93
1.00
4.60
2.30
7.30
f1 (ppm)

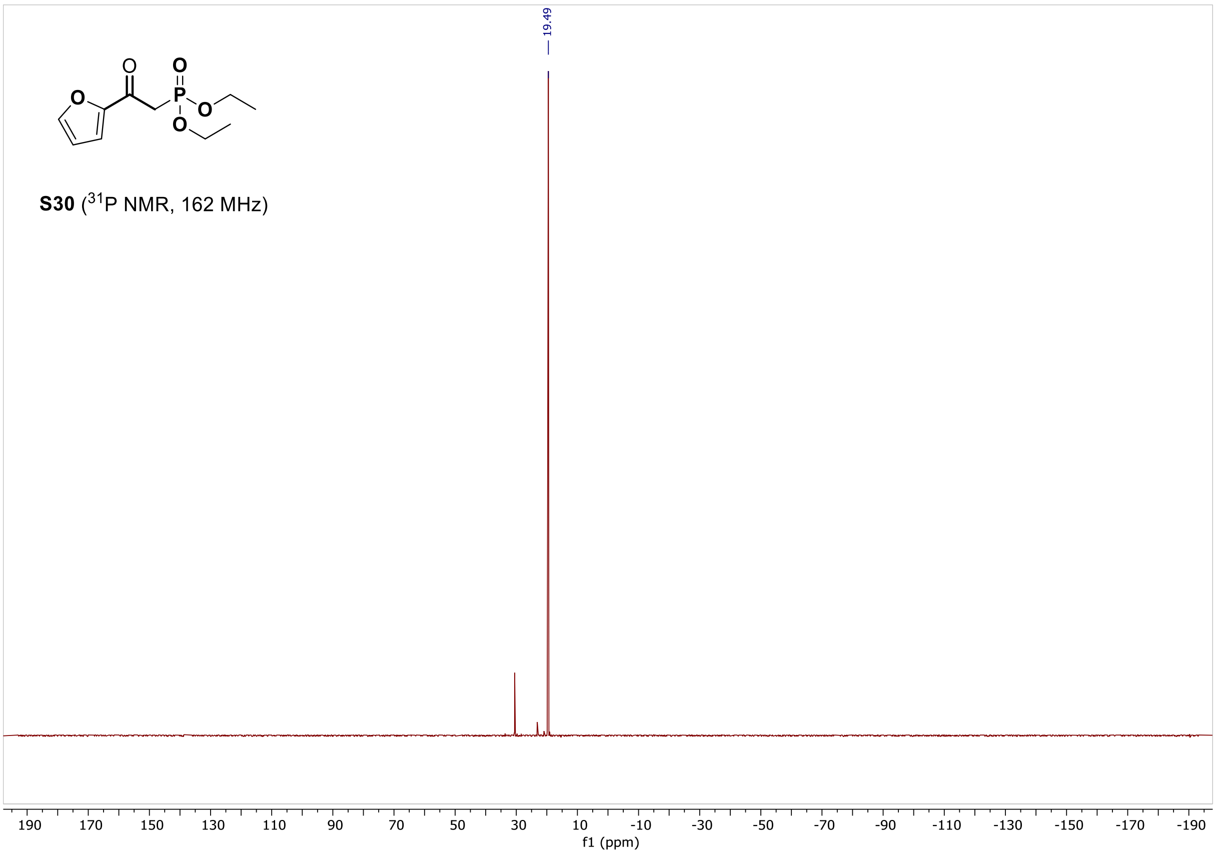
19.49
S30 (31P NMR, 162 MHz)
f1 (ppm)

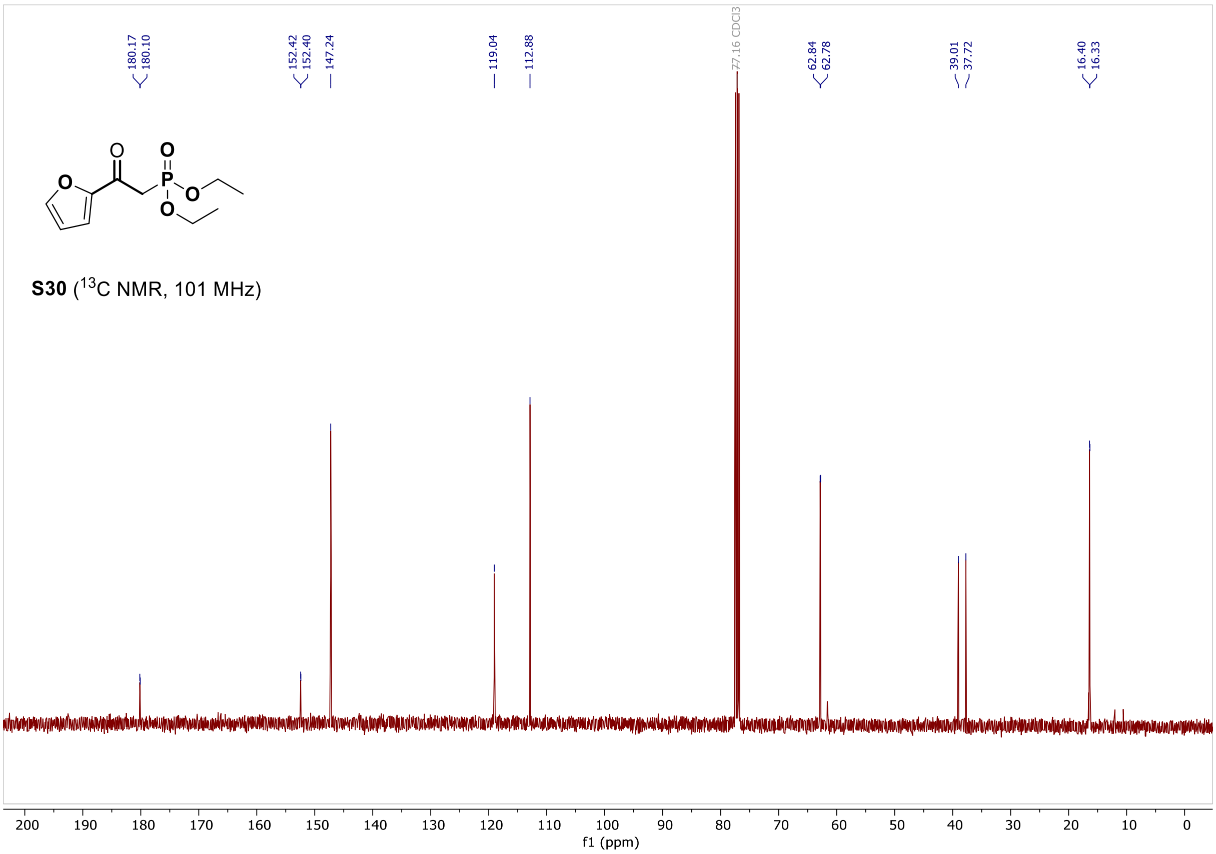

180.17
180.10
152.42
152.40
147.24
119.04
112.88
77.16 CDCl3
62.84
62.78
39.01
37.72
16.40
16.33
O
O
O
P
O
O
S30 (13C NMR, 101 MHz)
200
190
180
170
160
150
140
130
120
110
100
90
80
70
60
50
40
30
20
10
0
f1 (ppm)

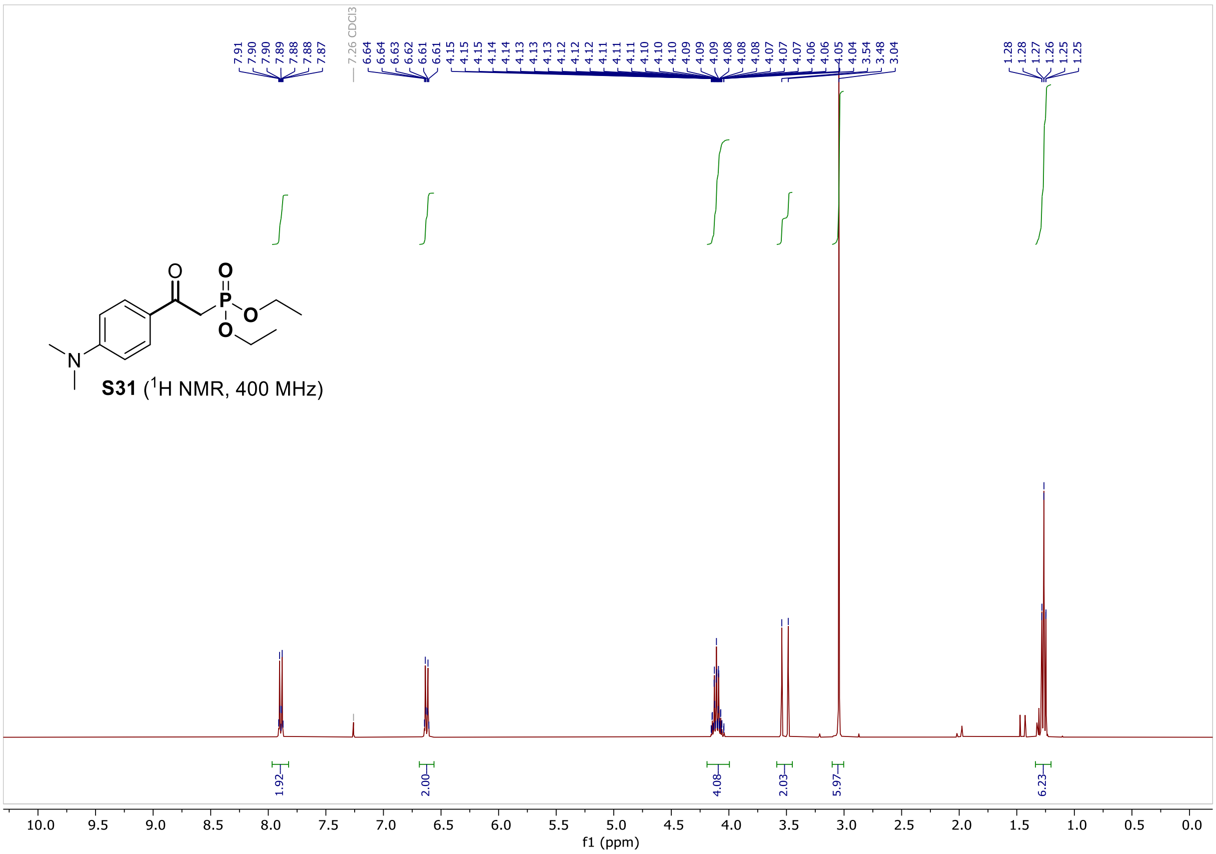

S31 (^{1}H NMR, 400 MHz)

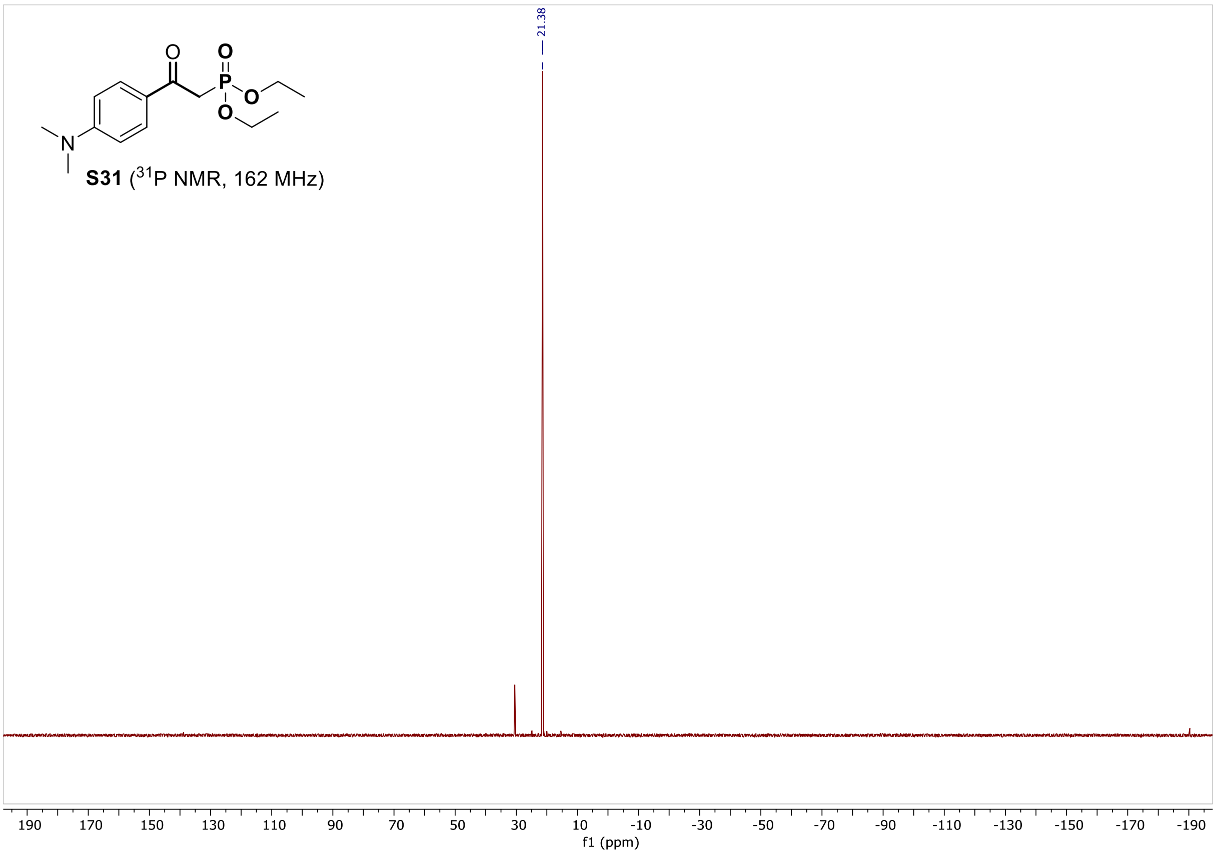

S31 (^{31}P NMR, 162 MHz)

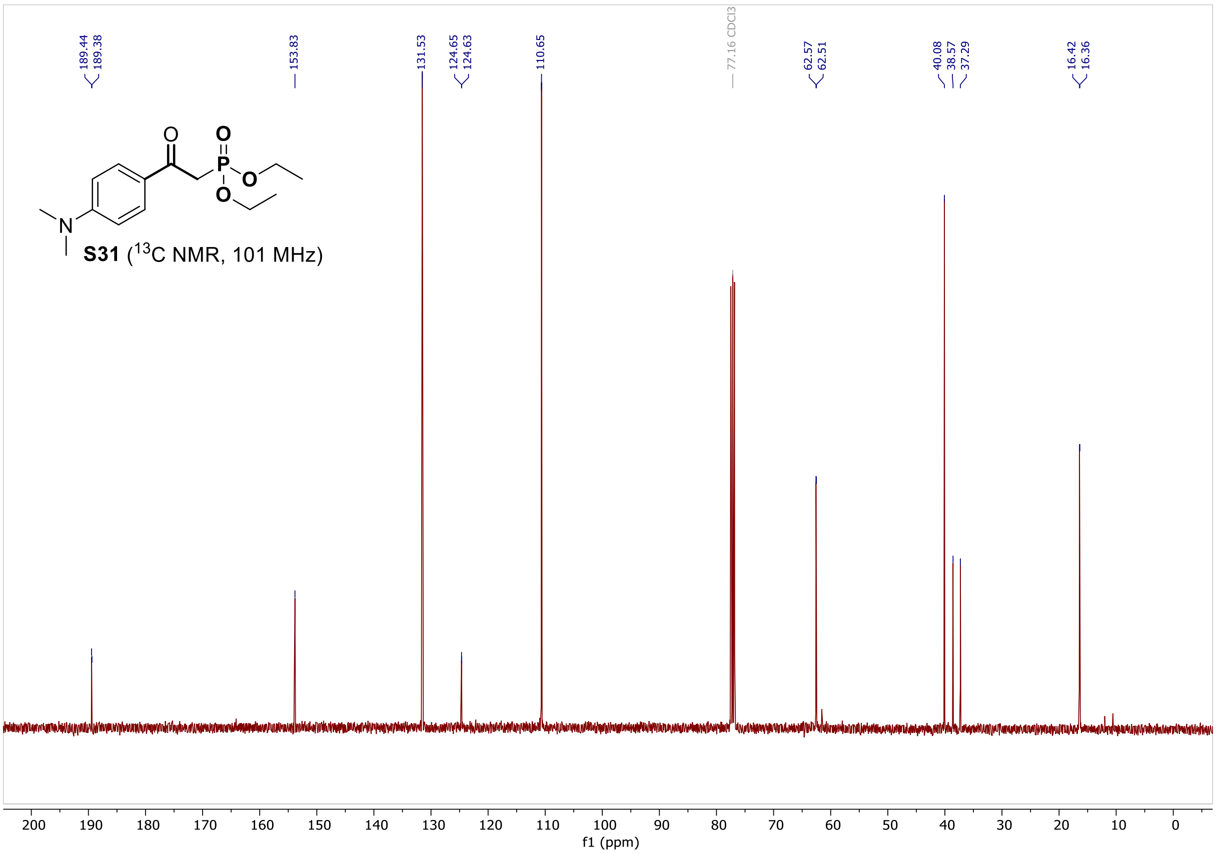
189.44
189.38
153.83
131.53
124.65
124.63
110.65
77.16 CDCl3
62.57
62.51
40.08
38.57
37.29
16.42
16.36
O
O
P
O
O
N
S31 (^{13}C NMR, 101 MHz)
200
190
180
170
160
150
140
130
120
110
100
90
80
70
60
50
40
30
20
10
0
f1 (ppm)

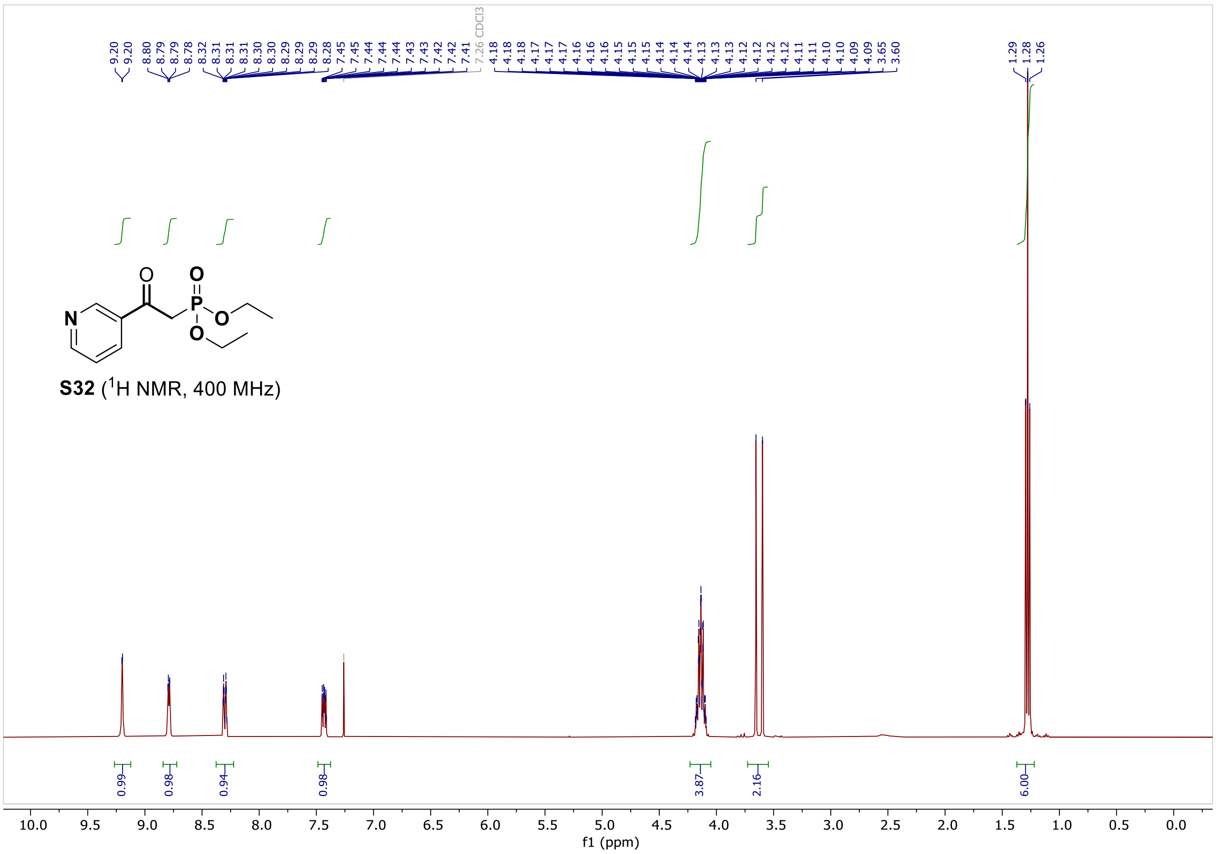

S32 (^{1}H NMR, 400 MHz)

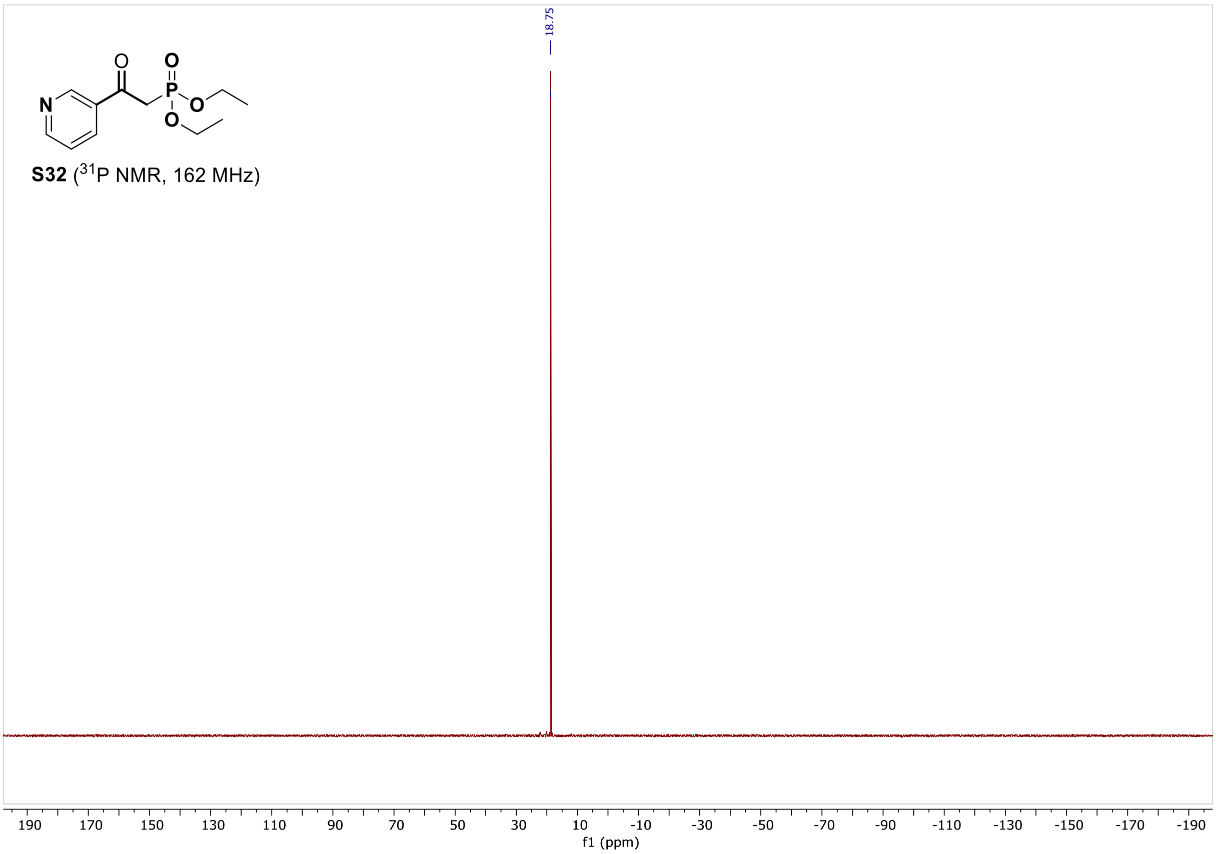

S32 (^{31}P NMR, 162 MHz)

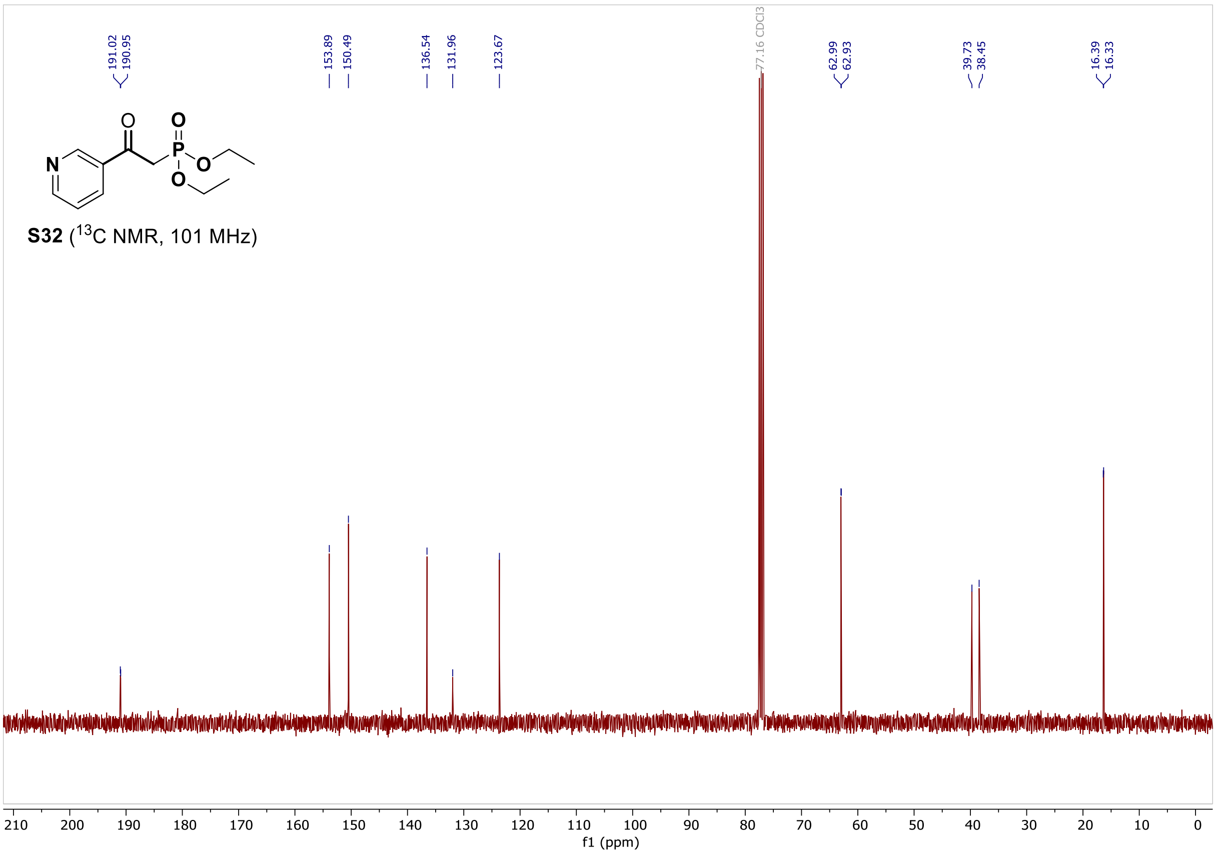
191.02
190.95
153.89
150.49
136.54
131.96
123.67
77.16 CDCl3
62.99
62.93
39.73
38.45
16.39
16.33
O
O
N
P
O
O
S32 (^{13}C NMR, 101 MHz)
210 200 190 180 170 160 150 140 130 120 110 100 90 80 70 60 50 40 30 20 10 0
f1 (ppm)

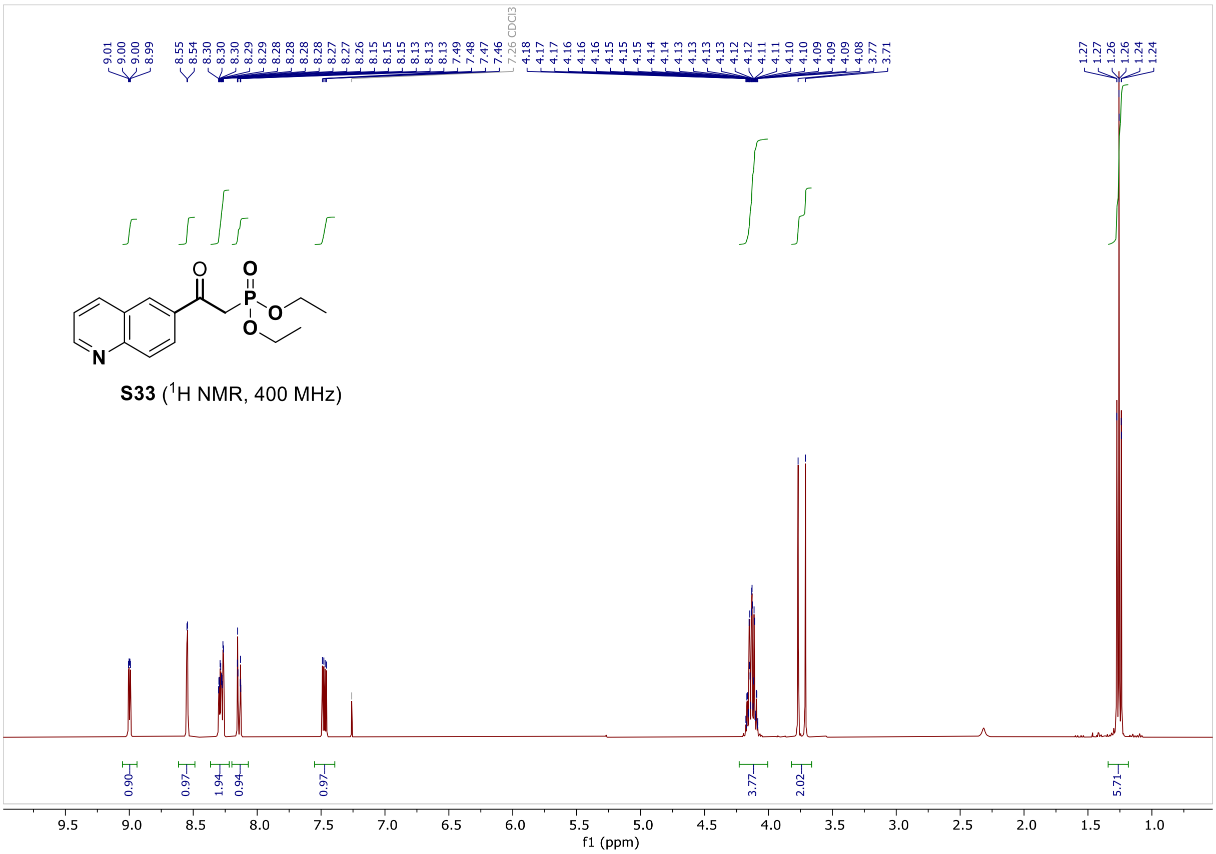
9.01
9.00
9.00
8.99
8.55
8.54
8.30
8.30
8.29
8.28
8.28
8.28
8.27
8.27
8.26
8.15
8.15
8.13
8.13
8.13
7.49
7.48
7.47
7.46
7.26 CDCl3
4.18
4.17
4.17
4.16
4.16
4.16
4.15
4.15
4.15
4.14
4.14
4.13
4.13
4.13
4.13
4.12
4.12
4.11
4.11
4.10
4.10
4.09
4.09
4.09
4.08
3.77
3.71
1.27
1.27
1.26
1.26
1.24
1.24
O
O
P
O
O
N
S33 (1H NMR, 400 MHz)
0.90
0.97
1.94
0.94
0.97
3.77
2.02
5.71
9.5
9.0
8.5
8.0
7.5
7.0
6.5
6.0
5.5
5.0
4.5
4.0
3.5
3.0
2.5
2.0
1.5
1.0
f1 (ppm)

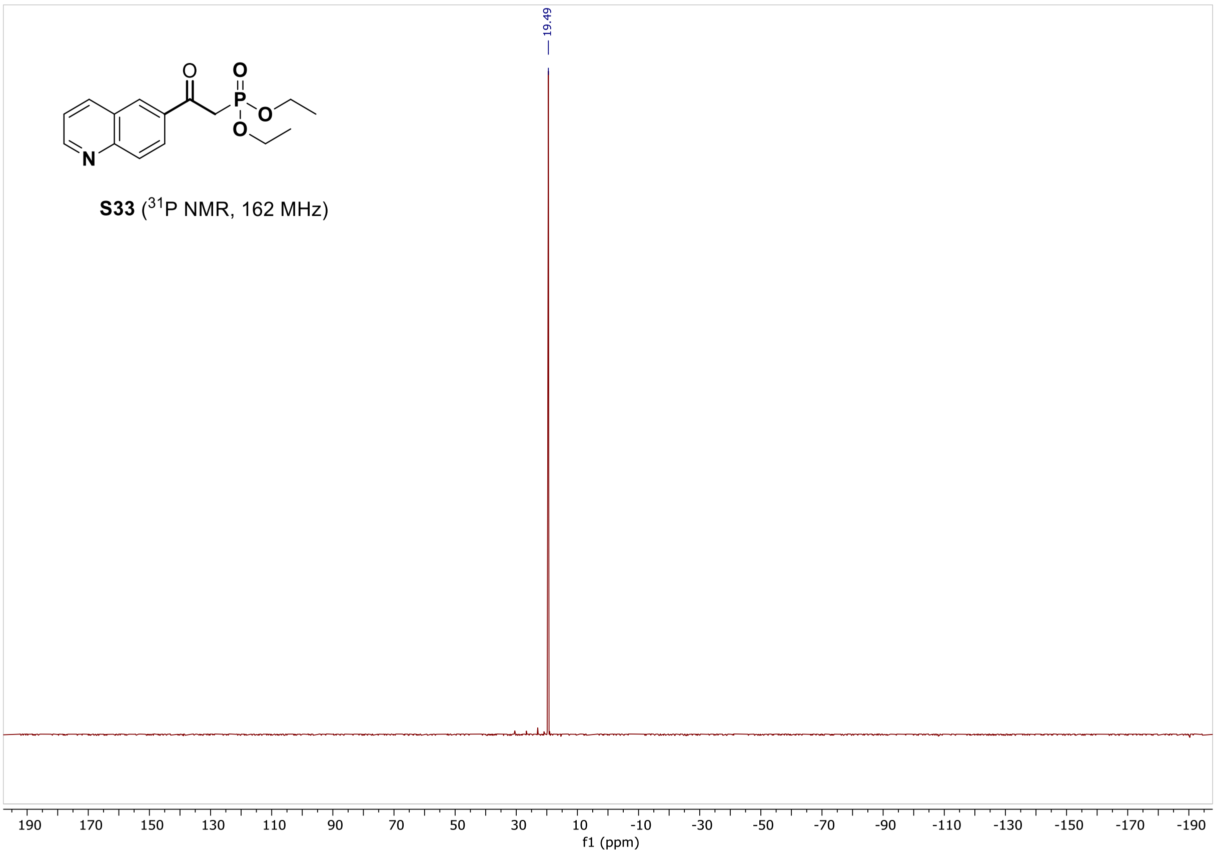
19.49
O
O
P
O
O
N
S33 (31P NMR, 162 MHz)
190
170
150
130
110
90
70
50
30
10
-10
-30
-50
-70
-90
-110
-130
-150
-170
-190
f1 (ppm)

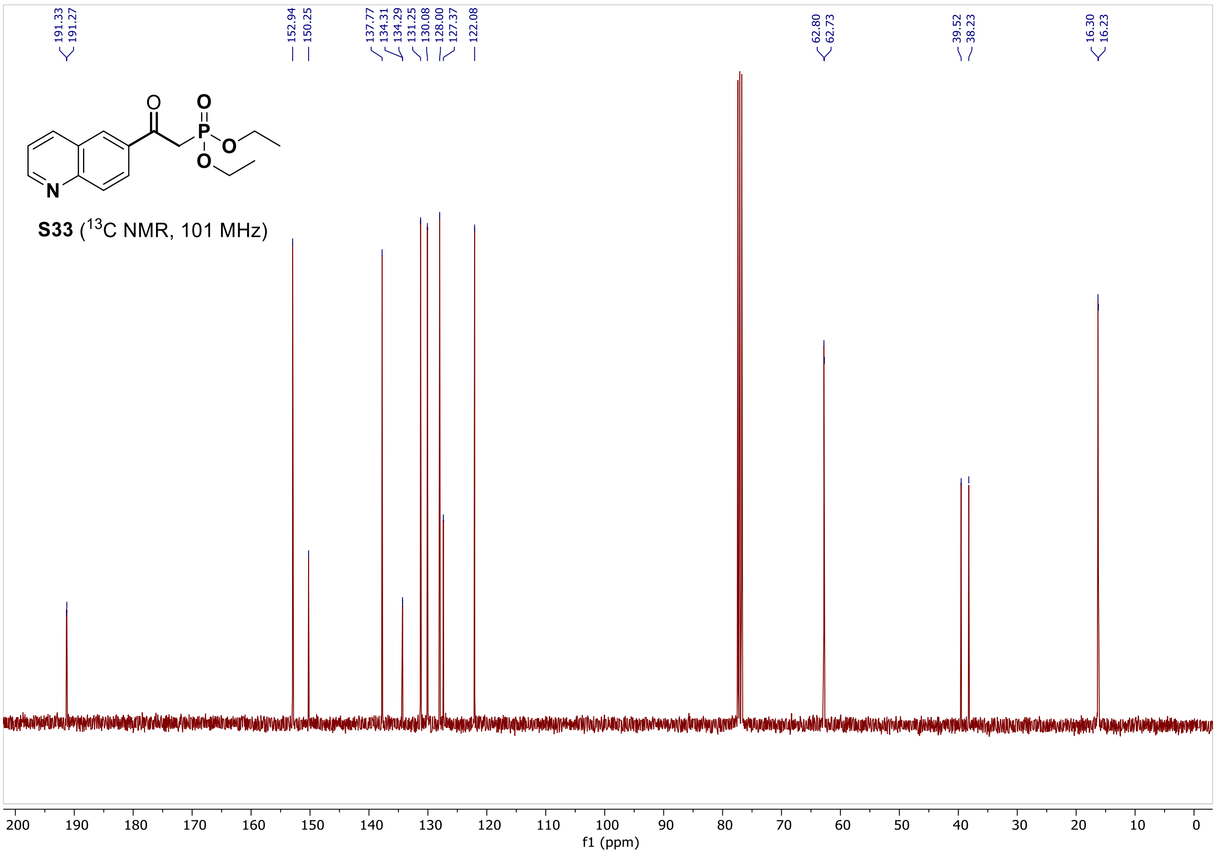
191.33
191.27
152.94
150.25
137.77
134.31
134.29
131.25
130.08
128.00
127.37
122.08
62.80
62.73
39.52
38.23
16.30
16.23
O
O
P
O
O
N
S33 (13C NMR, 101 MHz)
200
190
180
170
160
150
140
130
120
110
100
90
80
70
60
50
40
30
20
10
0
f1 (ppm)

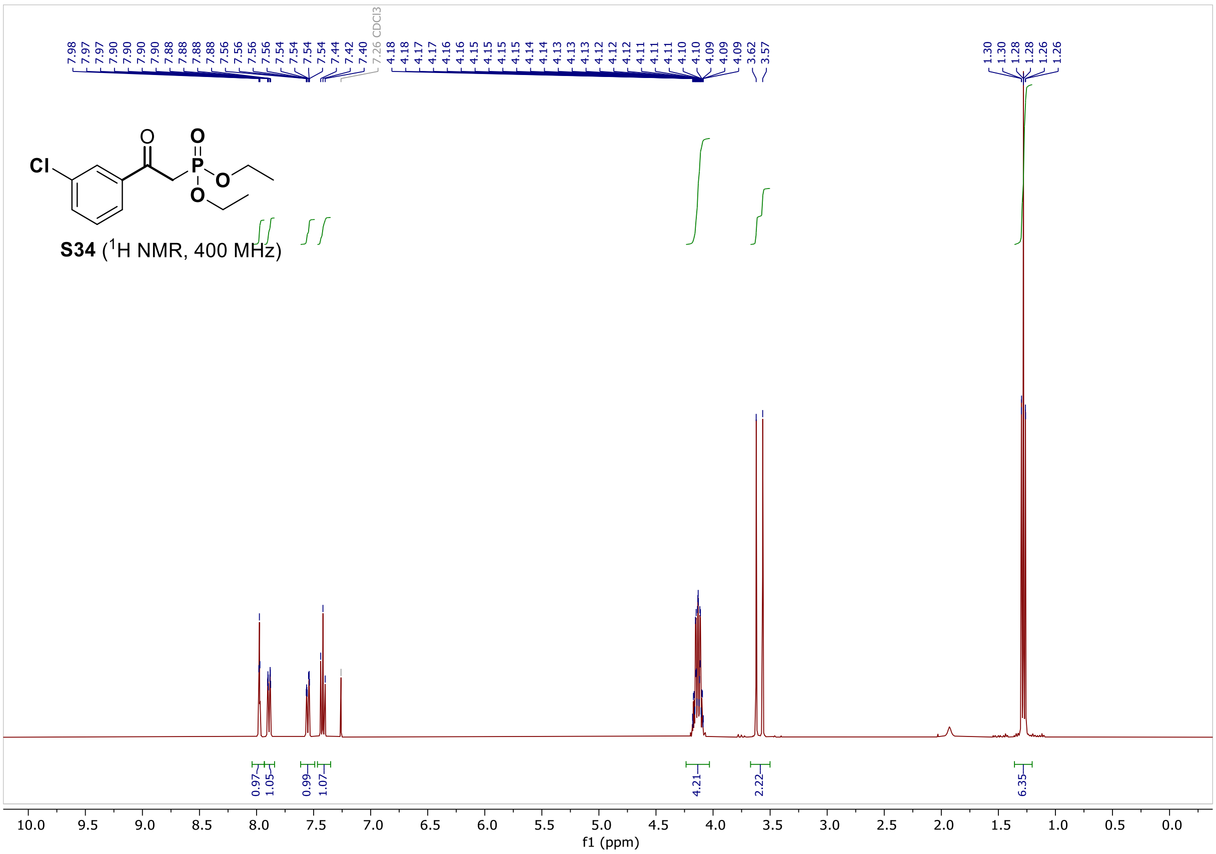

S34 (^{1}H NMR, 400 MHz)

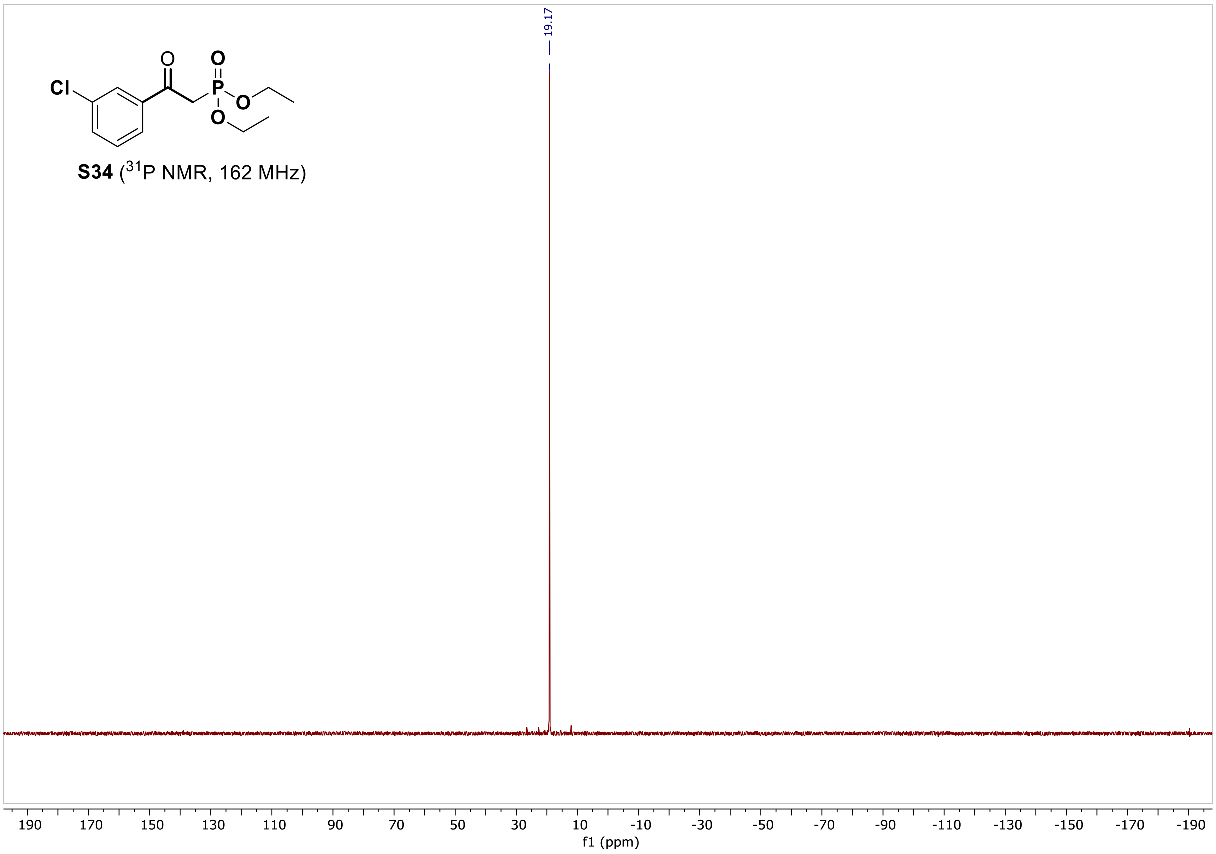

S34 (^{31}P NMR, 162 MHz)

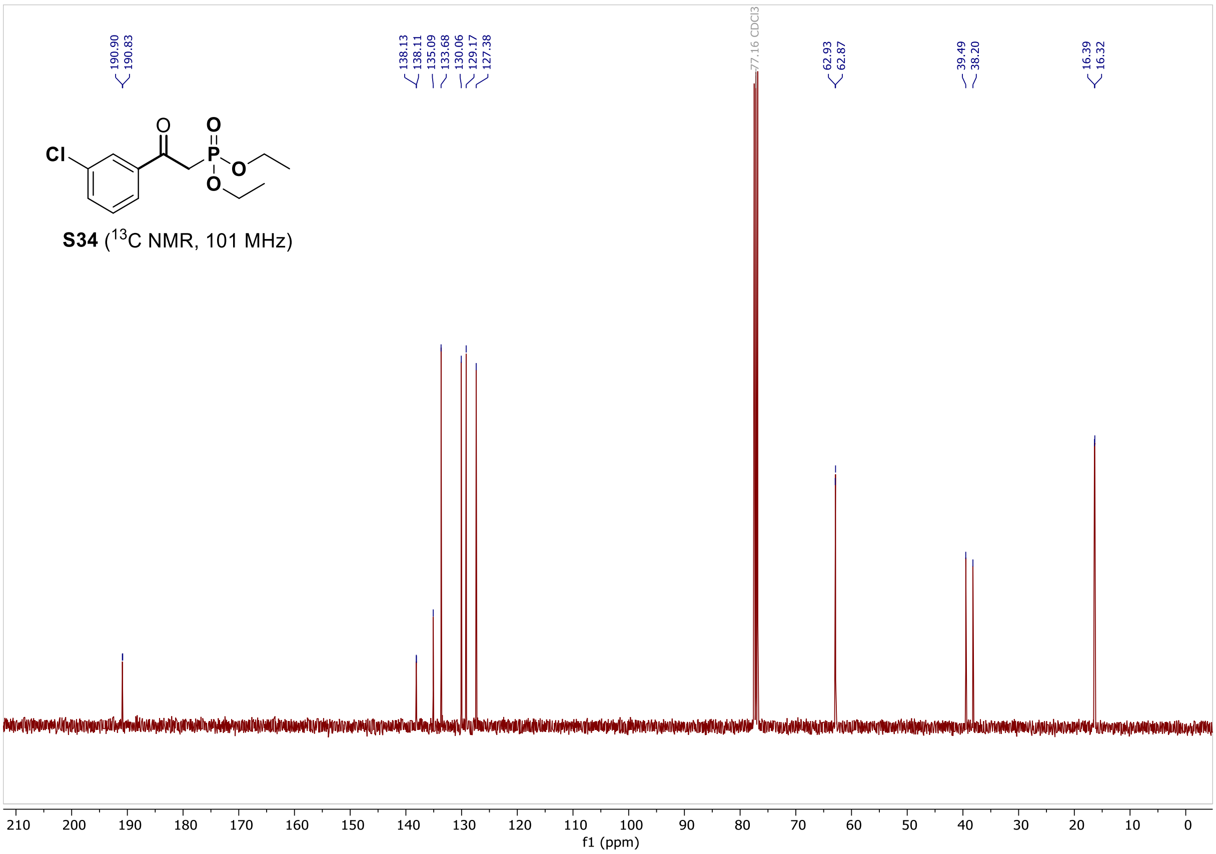
190.90
190.83
138.13
138.11
135.09
133.68
130.06
129.17
127.38
77.16 CDCl3
62.93
62.87
39.49
38.20
16.39
16.32
Cl
O
O
P
O
O
S34 (^{13}C NMR, 101 MHz)
210
200
190
180
170
160
150
140
130
120
110
100
90
80
70
60
50
40
30
20
10
0
f1 (ppm)

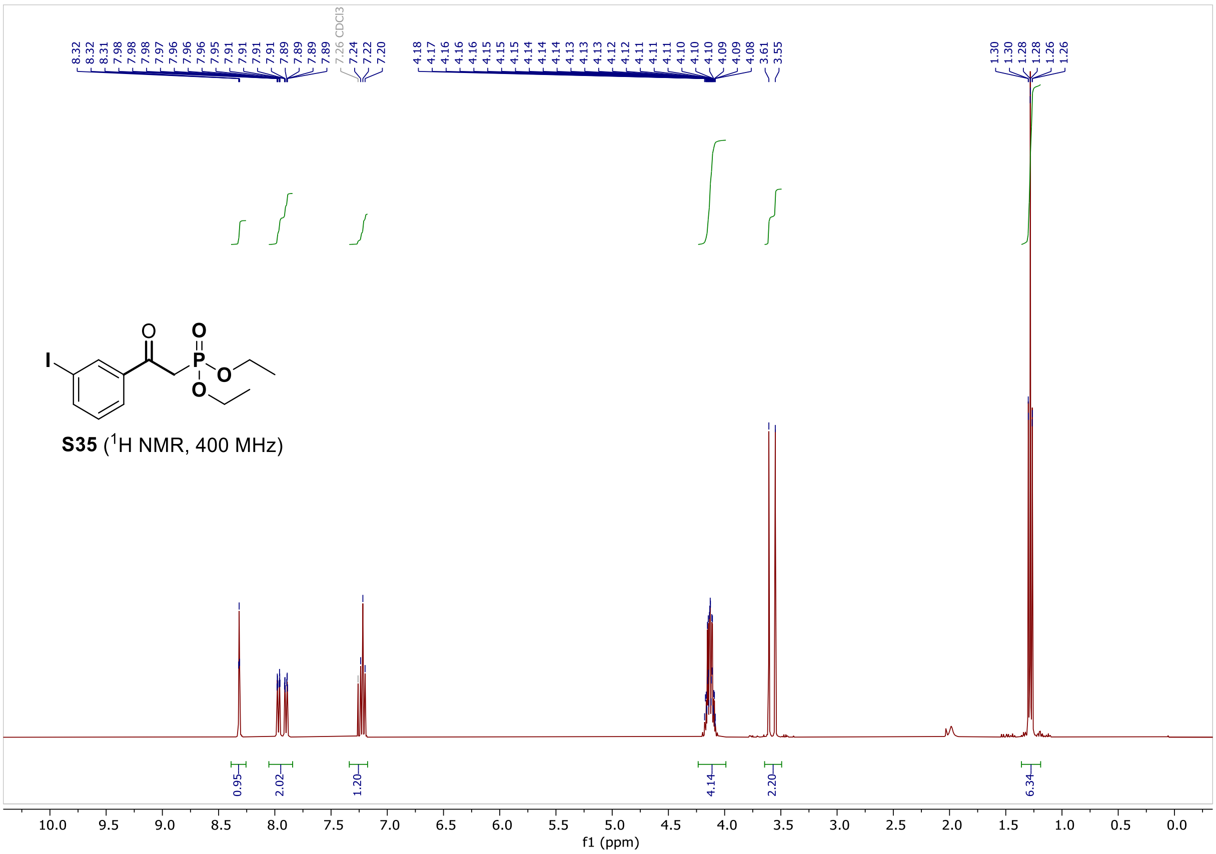

S35 (^{1}H NMR, 400 MHz)

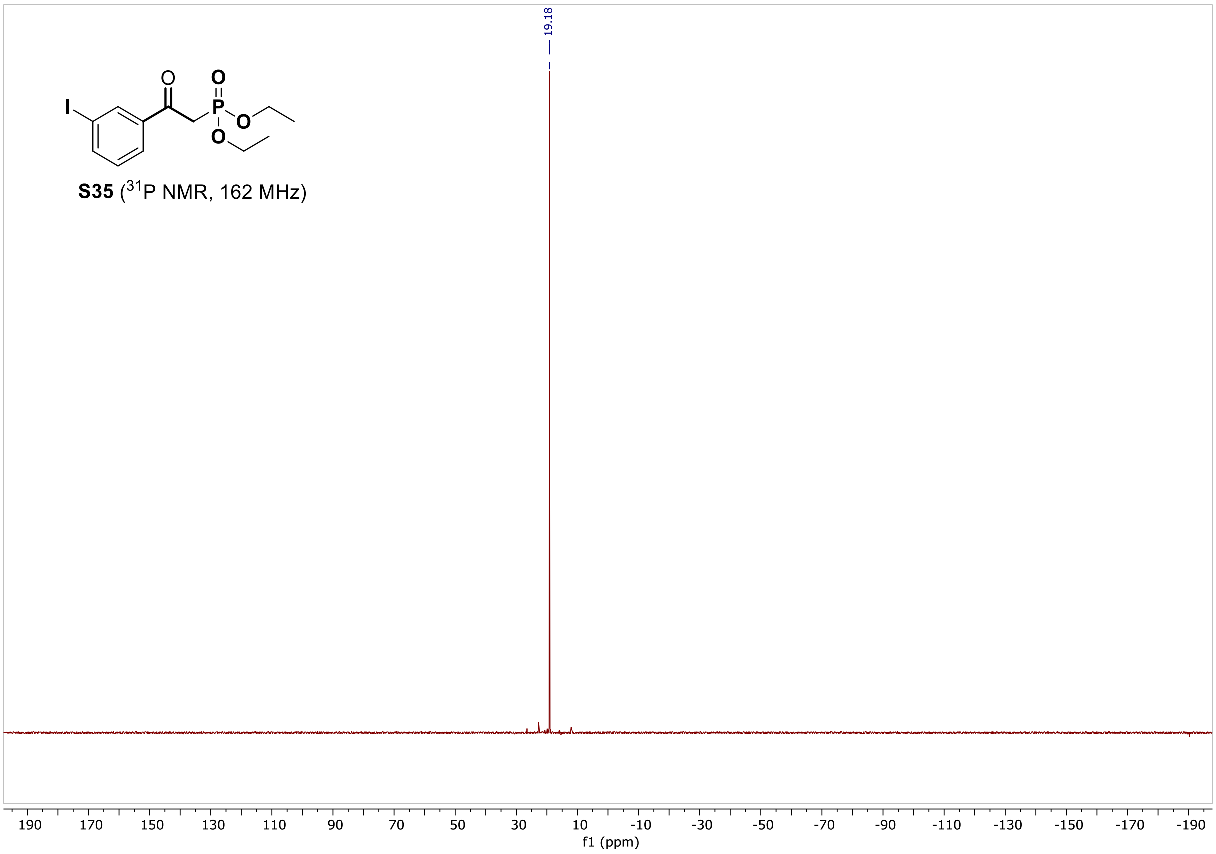

S35 (^{31}P NMR, 162 MHz)

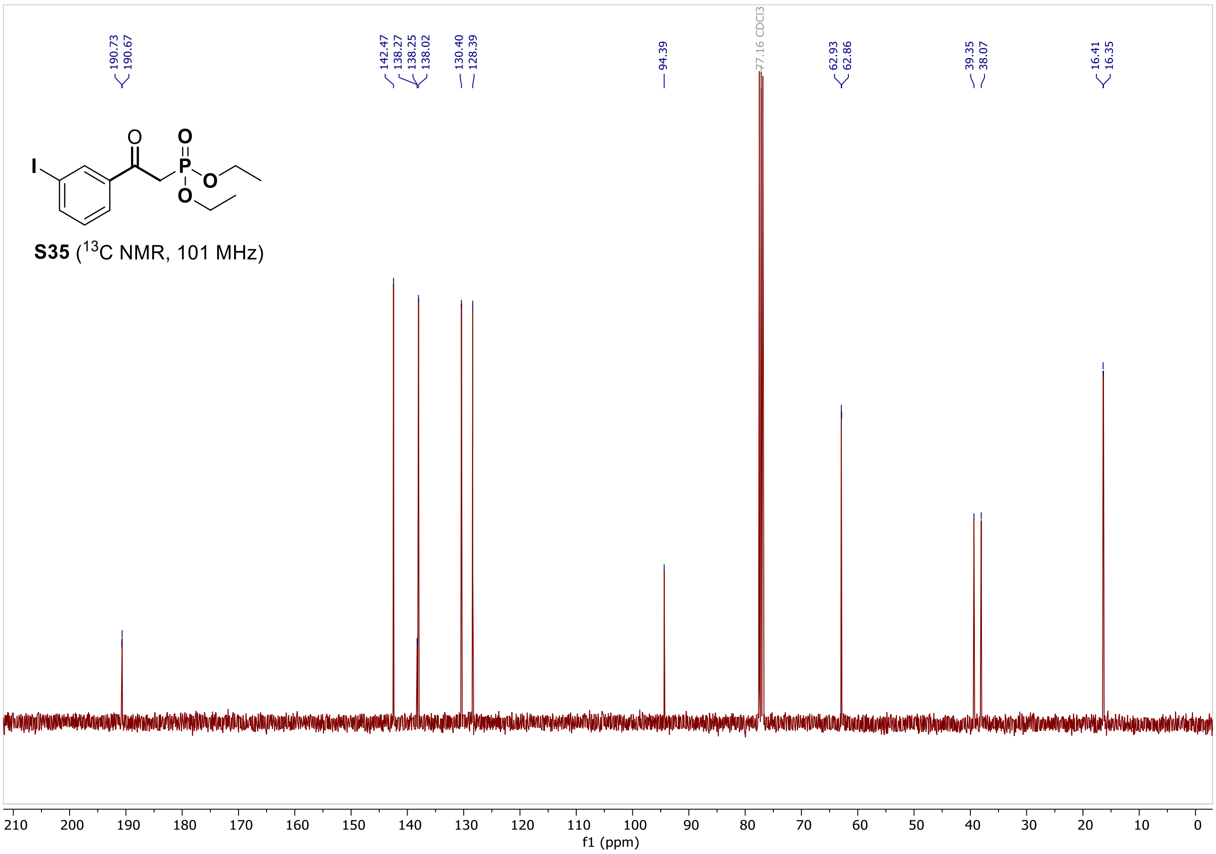

190.73
190.67
142.47
138.27
138.25
138.02
130.40
128.39
94.39
77.16 CDCl3
62.93
62.86
39.35
38.07
16.41
16.35
S35 (13C NMR, 101 MHz)
210 200 190 180 170 160 150 140 130 120 110 100 90 80 70 60 50 40 30 20 10 0
f1 (ppm)

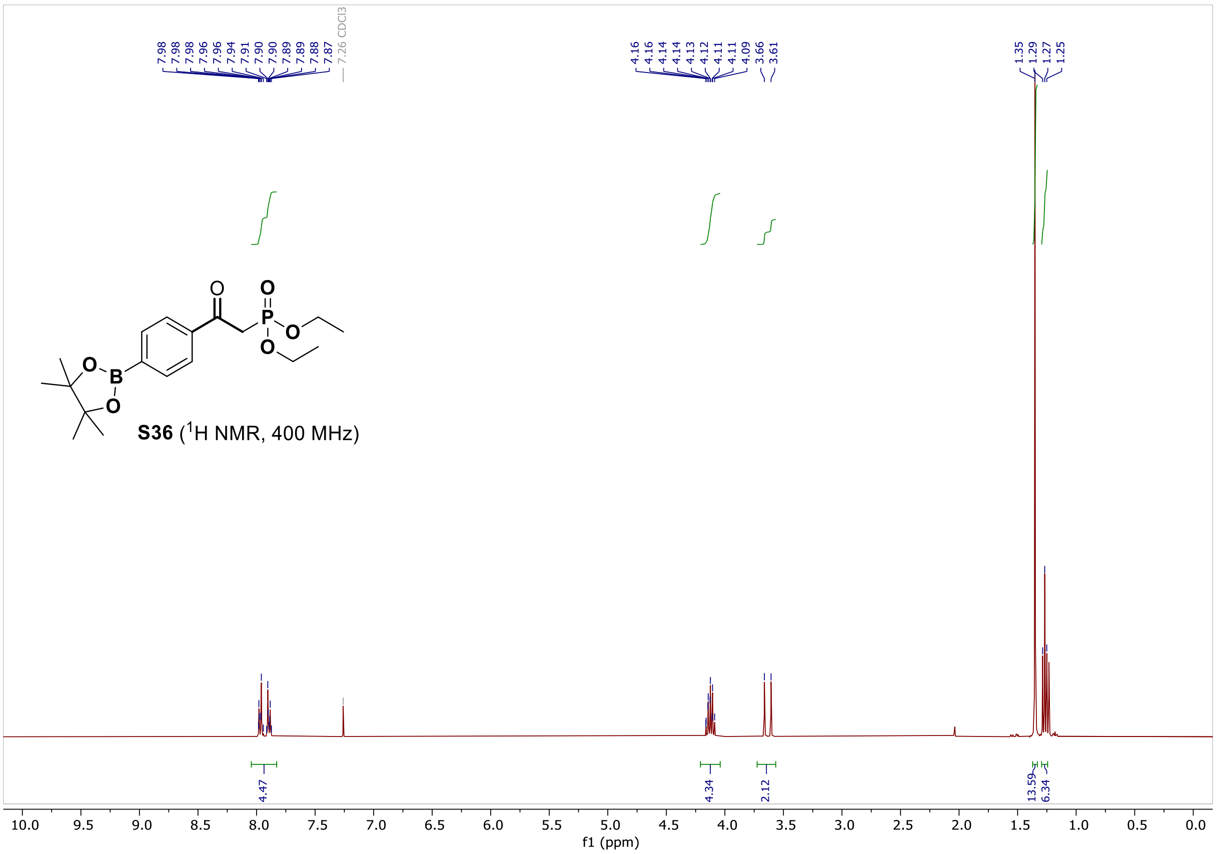
S36 (1H NMR, 400 MHz)
f1 (ppm)

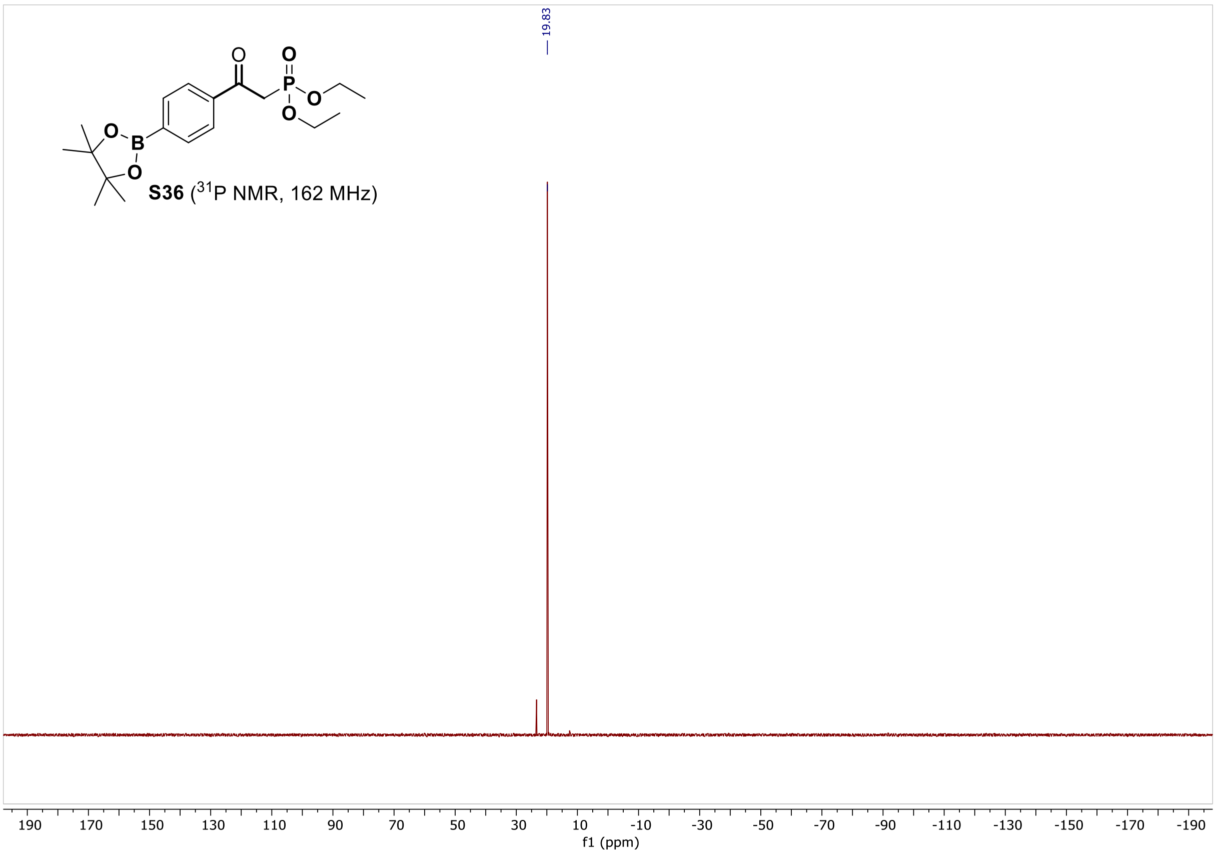
S36 (31P NMR, 162 MHz)
19.83
f1 (ppm)

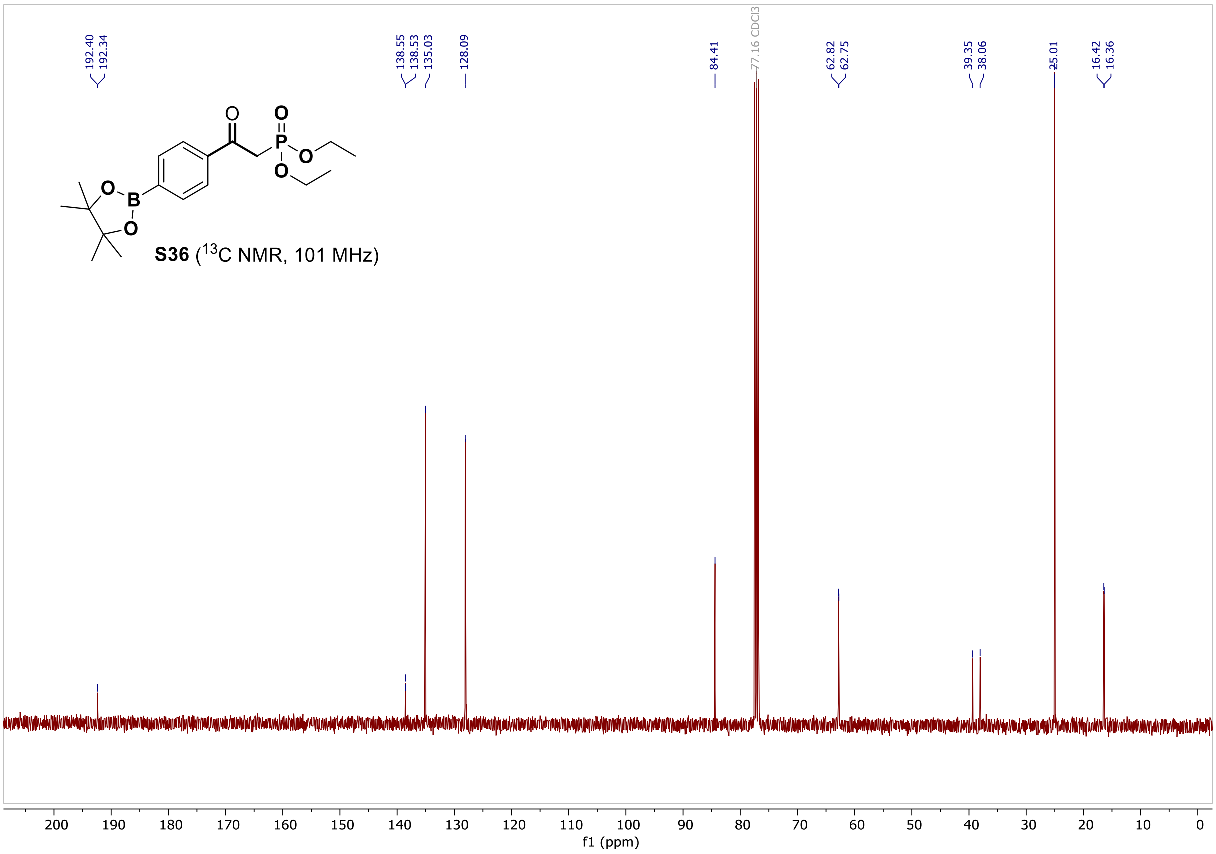
192.40
192.34
138.55
138.53
135.03
128.09
84.41
77.16 CDCl3
62.82
62.75
39.35
38.06
25.01
16.42
16.36
S36 (^{13}C NMR, 101 MHz)
f1 (ppm)

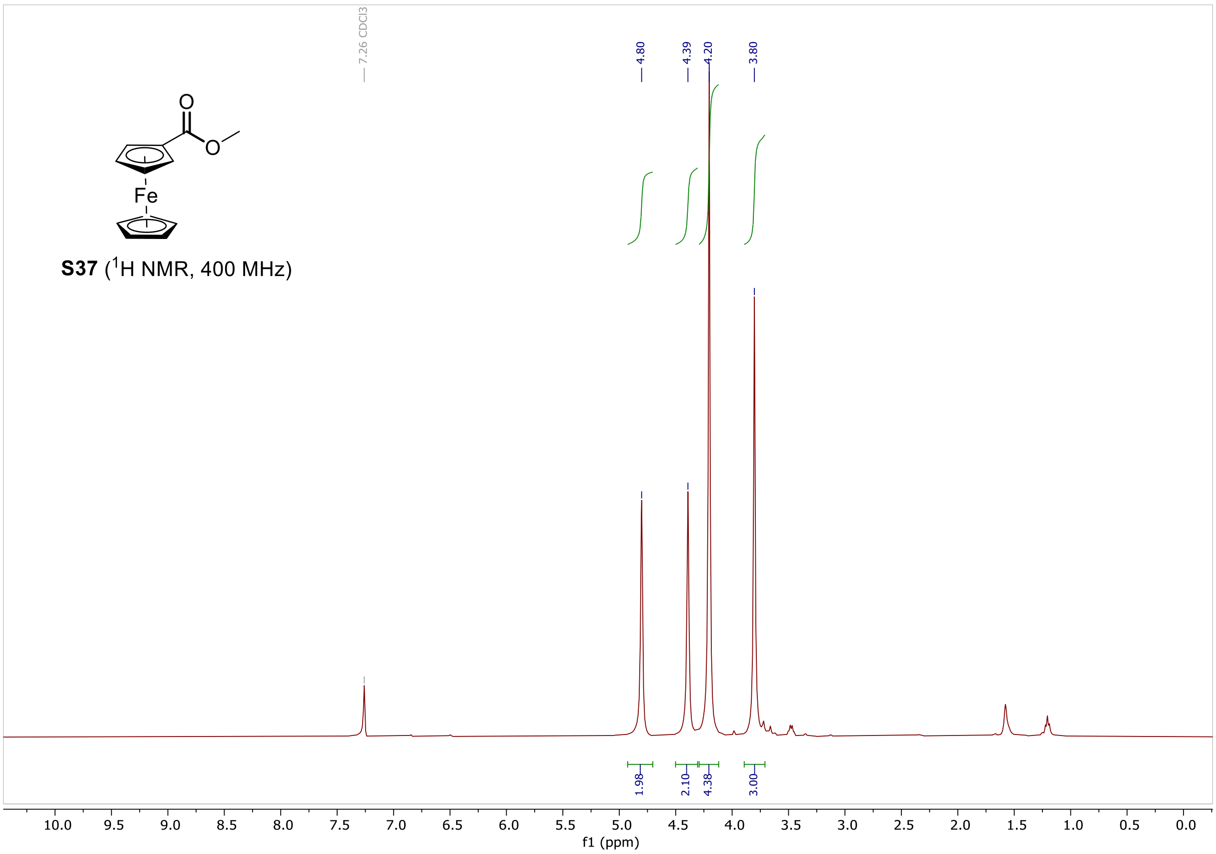

S37 (^{1}H NMR, 400 MHz)

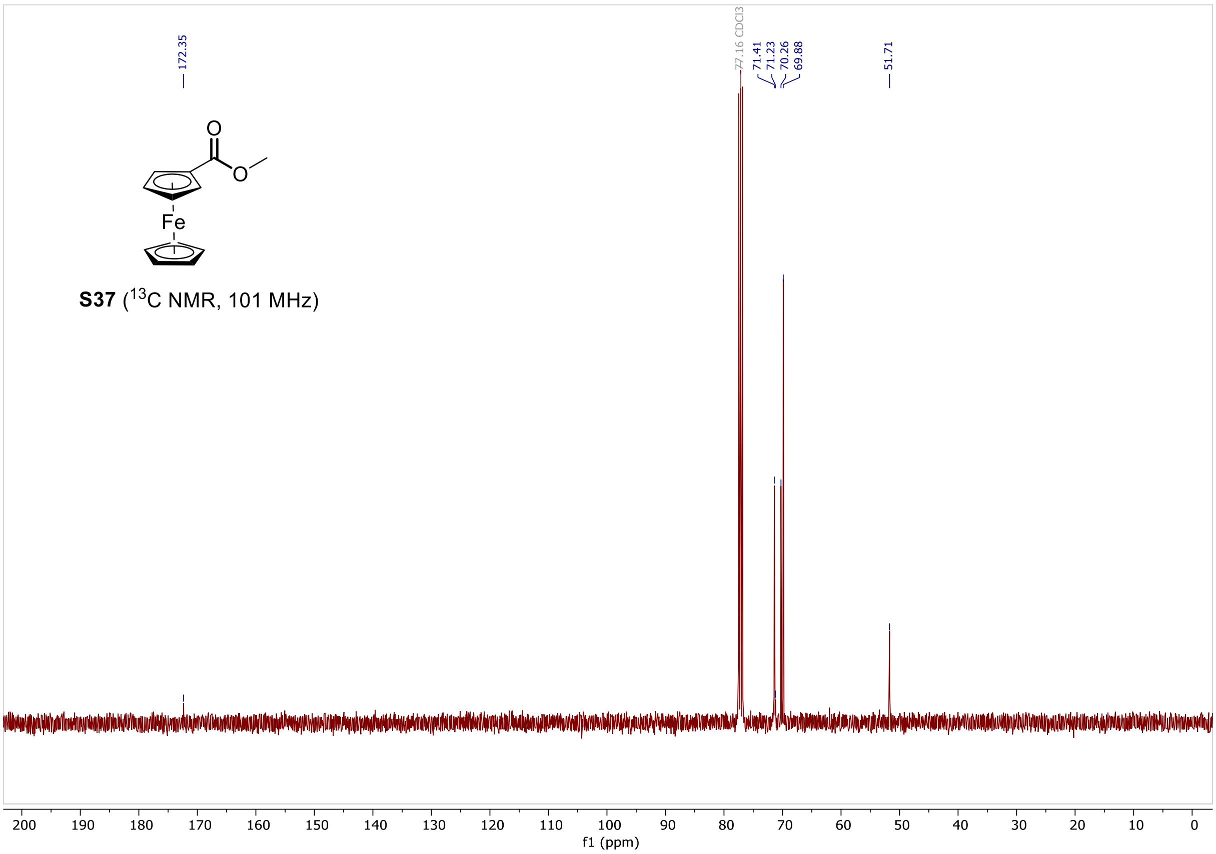

S37 (^{13}C NMR, 101 MHz)

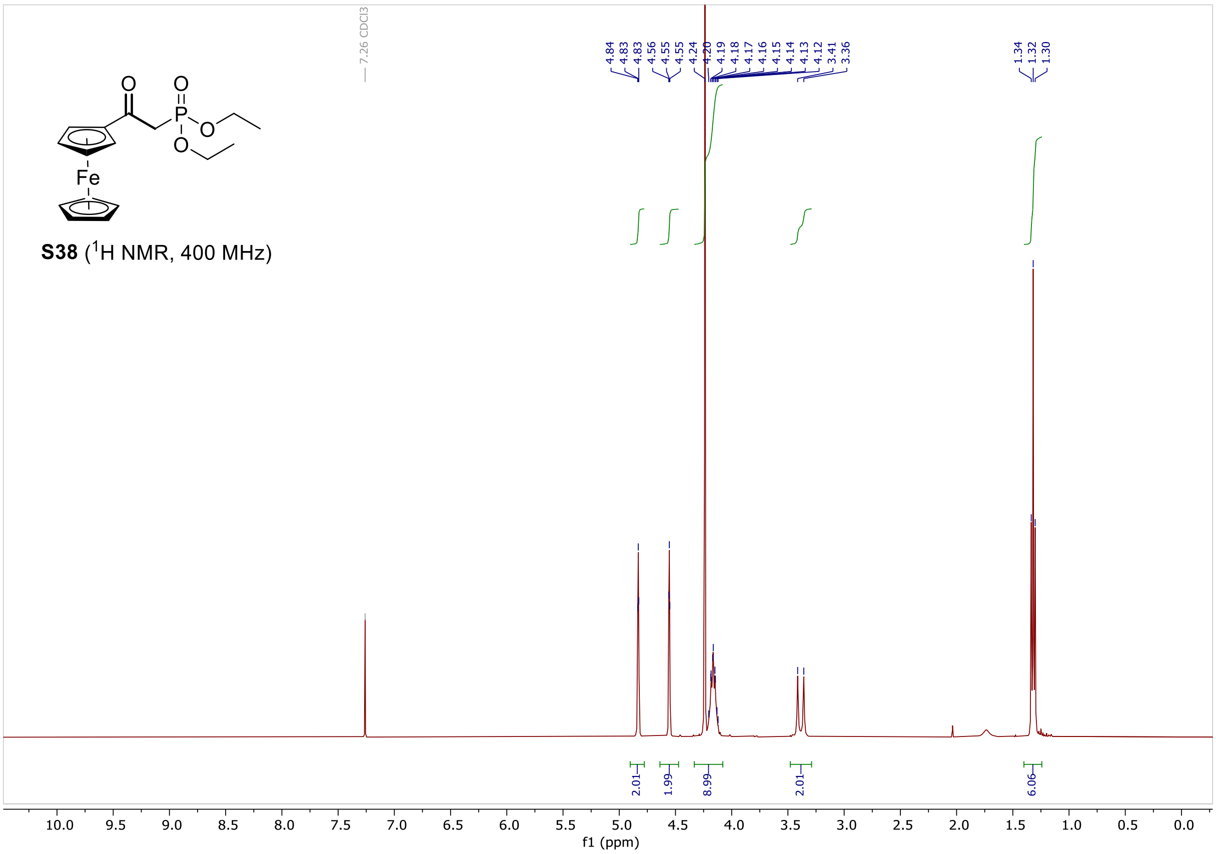

S38 (¹H NMR, 400 MHz)
7.26 CDCl3
4.84
4.83
4.83
4.56
4.55
4.55
4.24
4.20
4.19
4.18
4.17
4.16
4.15
4.14
4.13
4.12
3.41
3.36
1.34
1.32
1.30
2.01
1.99
8.99
2.01
6.06
f1 (ppm)

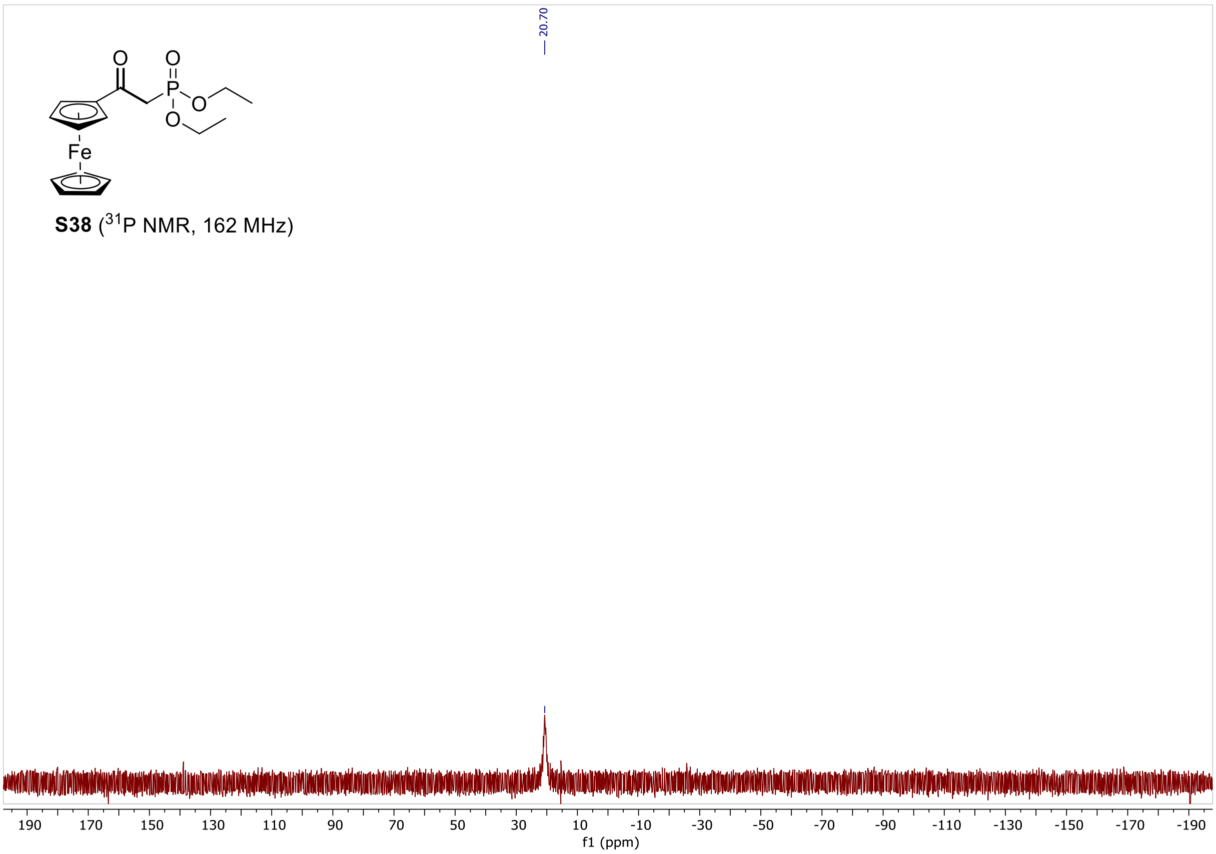

S38 (³¹P NMR, 162 MHz)
20.70
f1 (ppm)

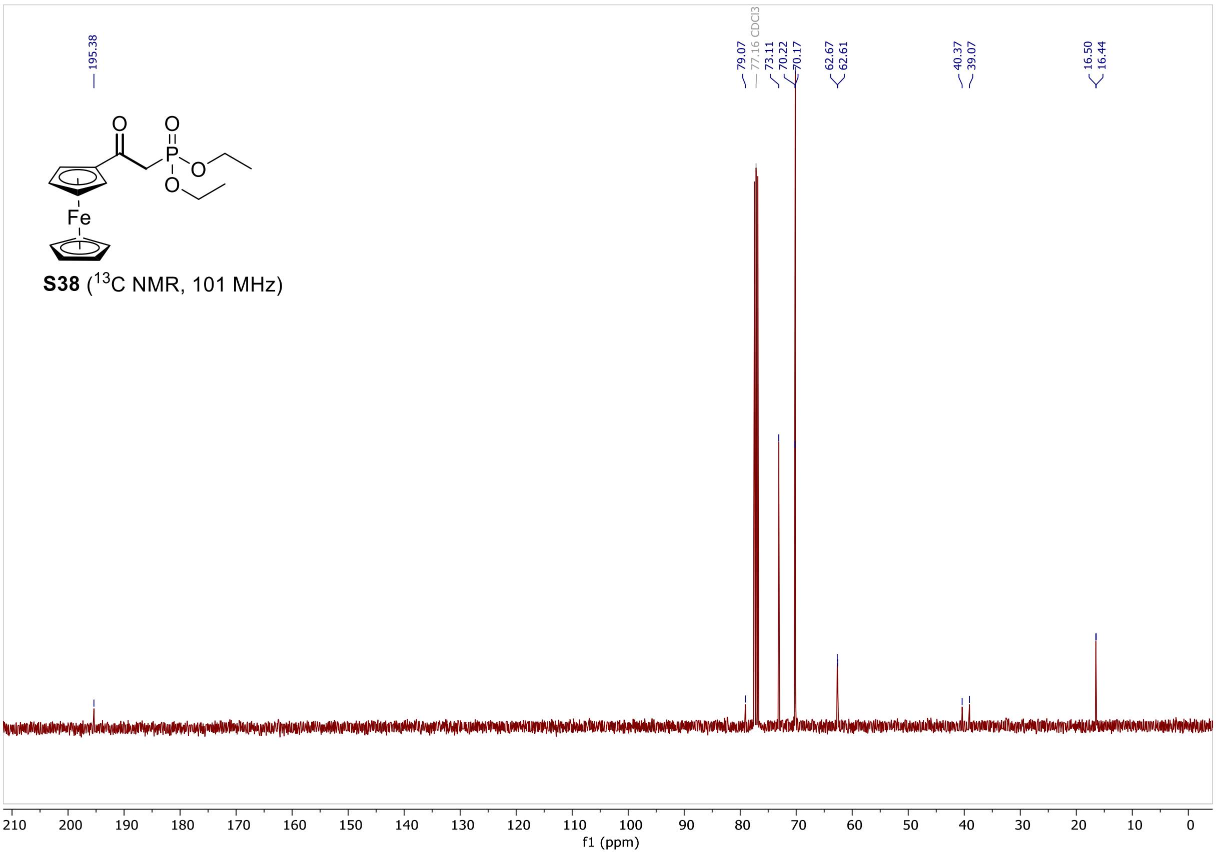

S38 (^{13}C NMR, 101 MHz)

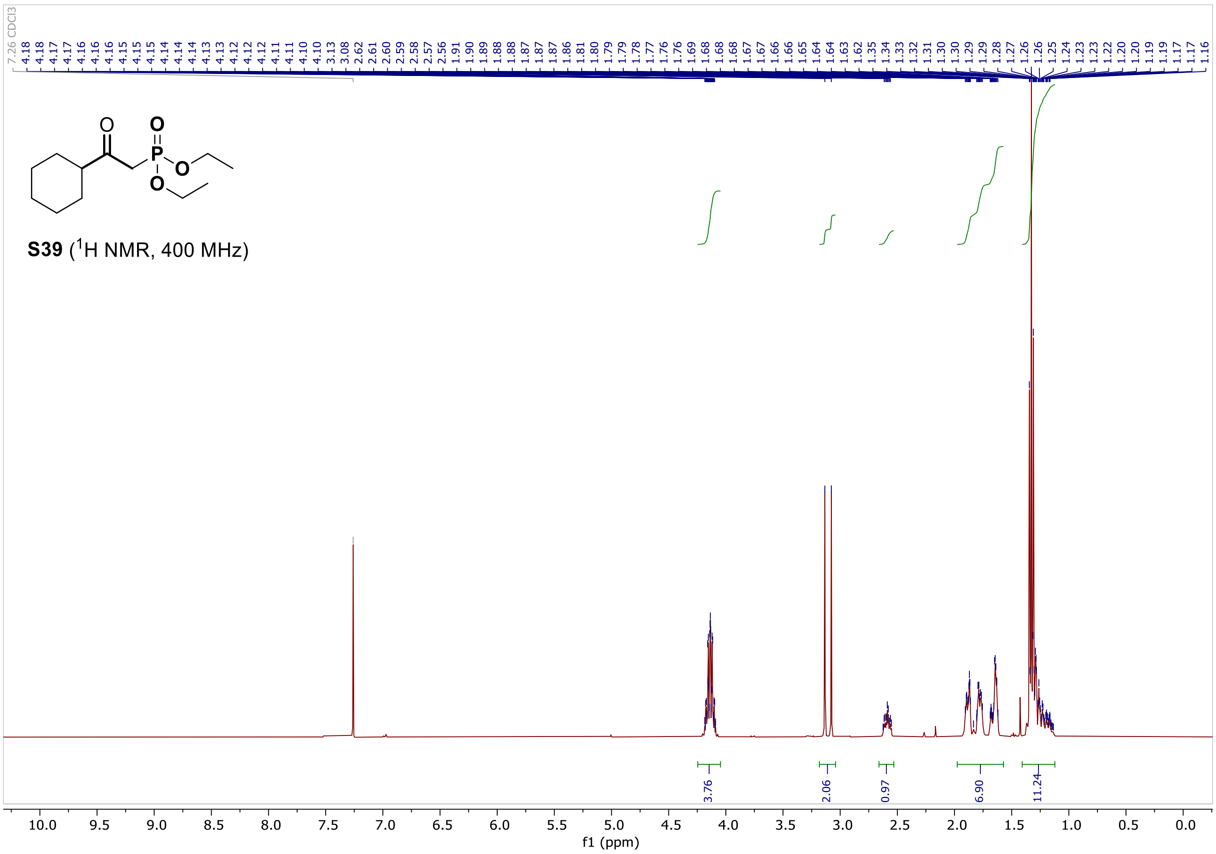
S39 (1H NMR, 400 MHz)
f1 (ppm)

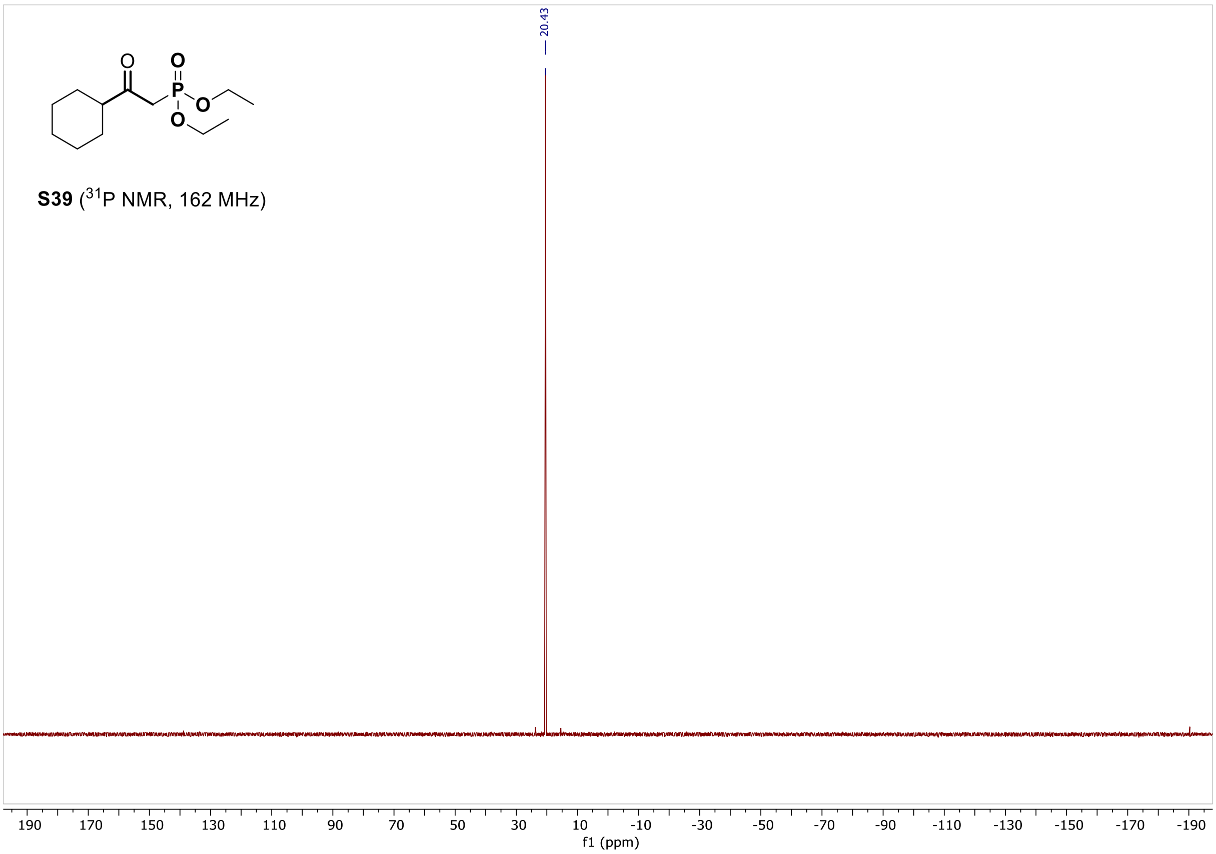
S39 (31P NMR, 162 MHz)
20.43
f1 (ppm)

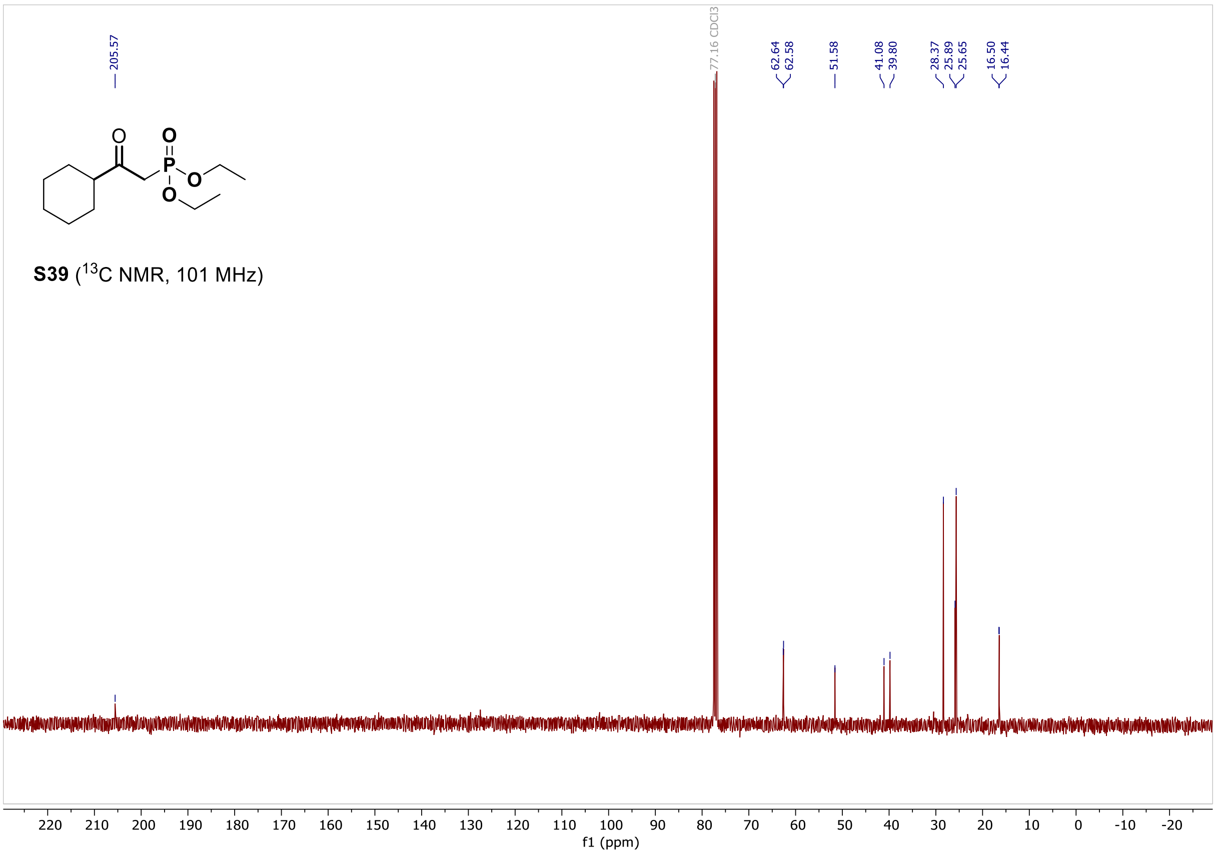
205.57
77.16 CDCl3
62.64
62.58
51.58
41.08
39.80
28.37
25.89
25.65
16.50
16.44
S39 (13C NMR, 101 MHz)
220 210 200 190 180 170 160 150 140 130 120 110 100 90 80 70 60 50 40 30 20 10 0 -10 -20
f1 (ppm)

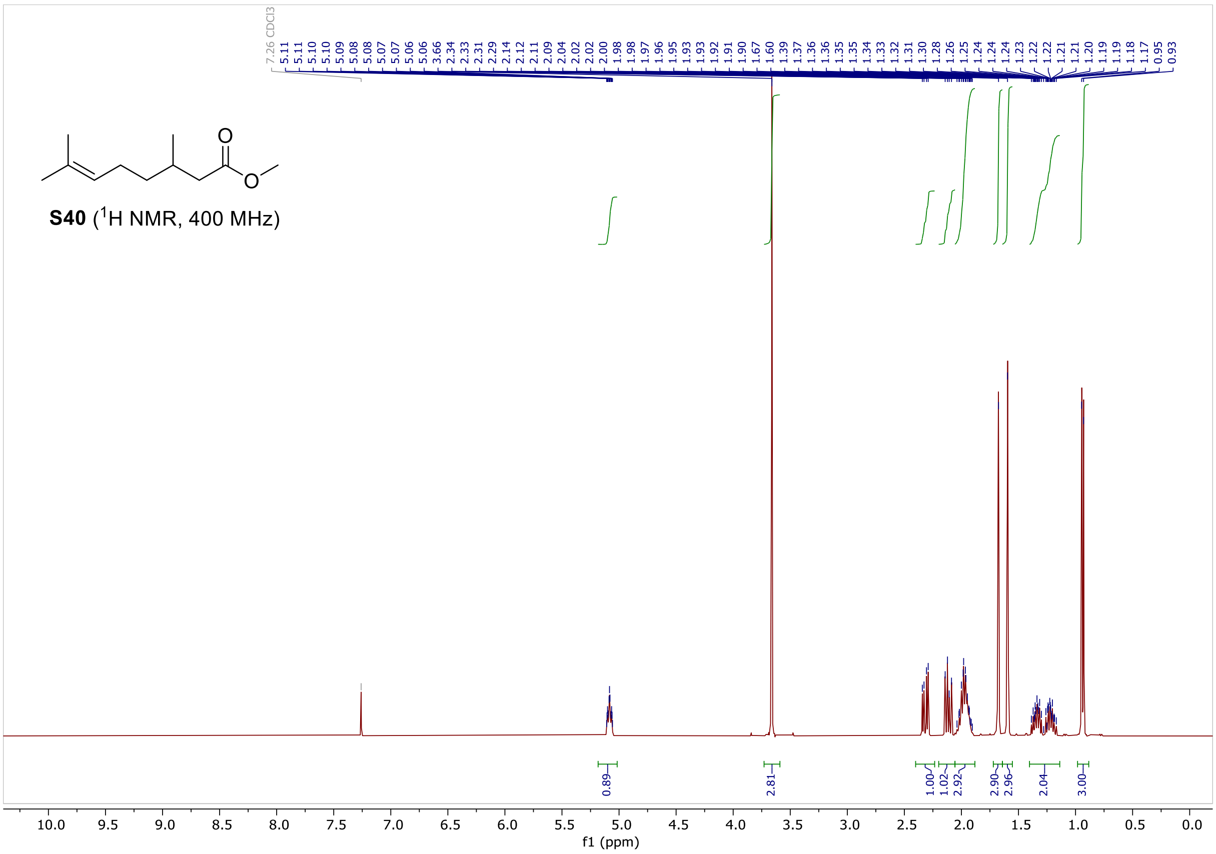

S40 (^{1}H NMR, 400 MHz)

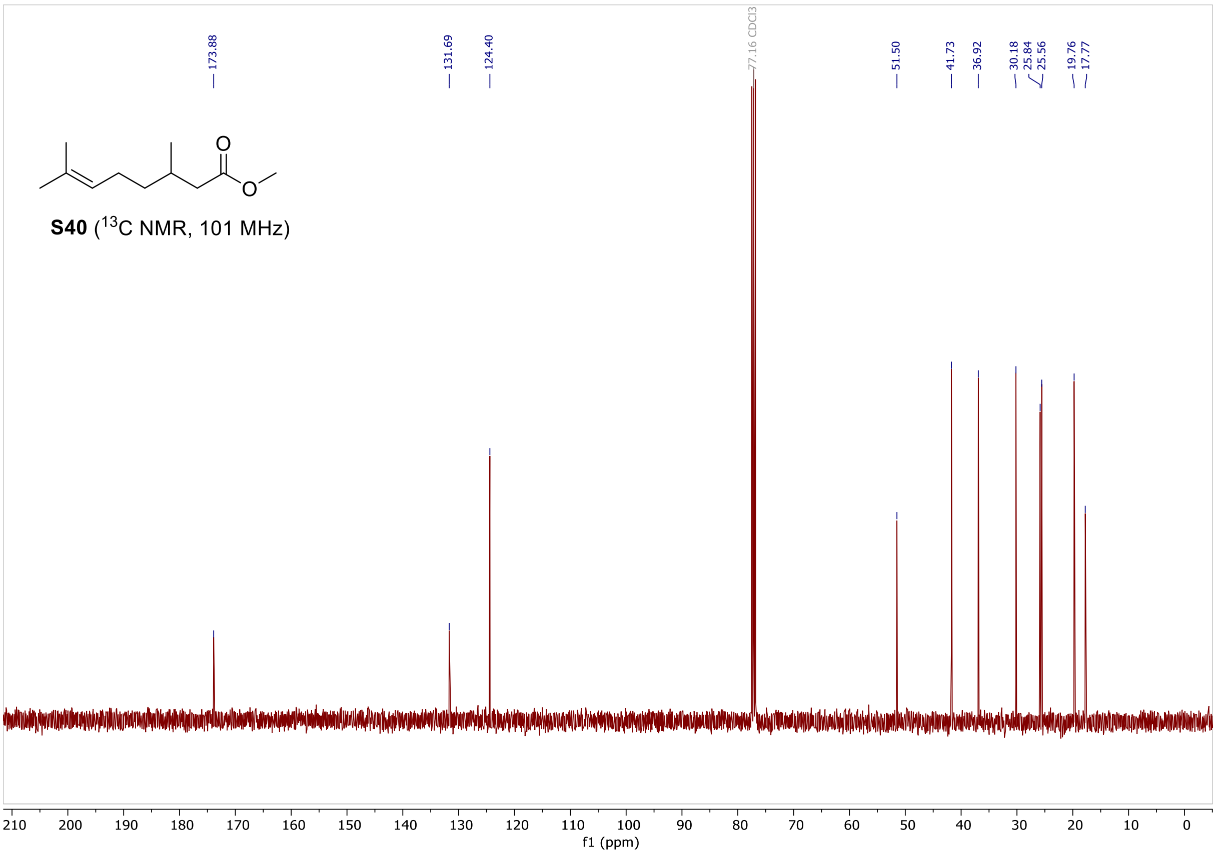

S40 (^{13}C NMR, 101 MHz)

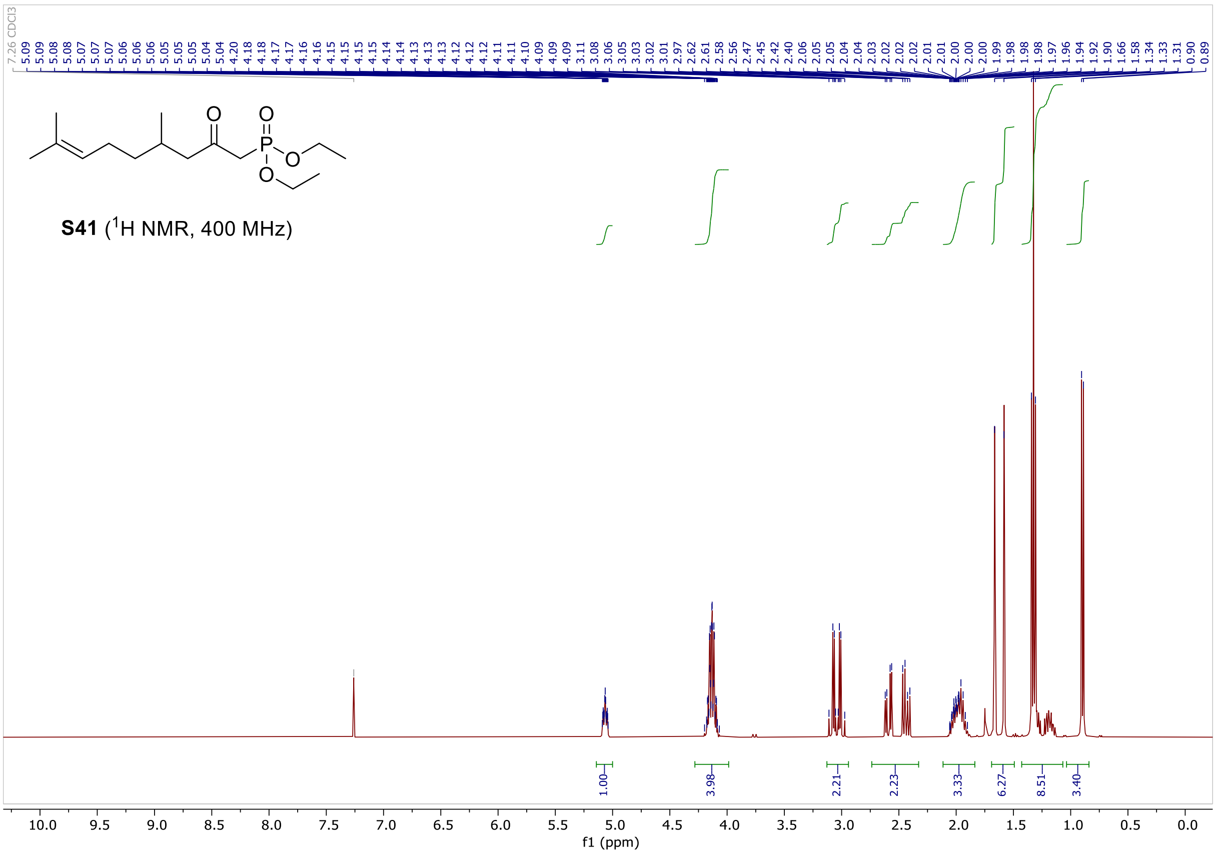
S41 (^{1}H NMR, 400 MHz)
f1 (ppm)

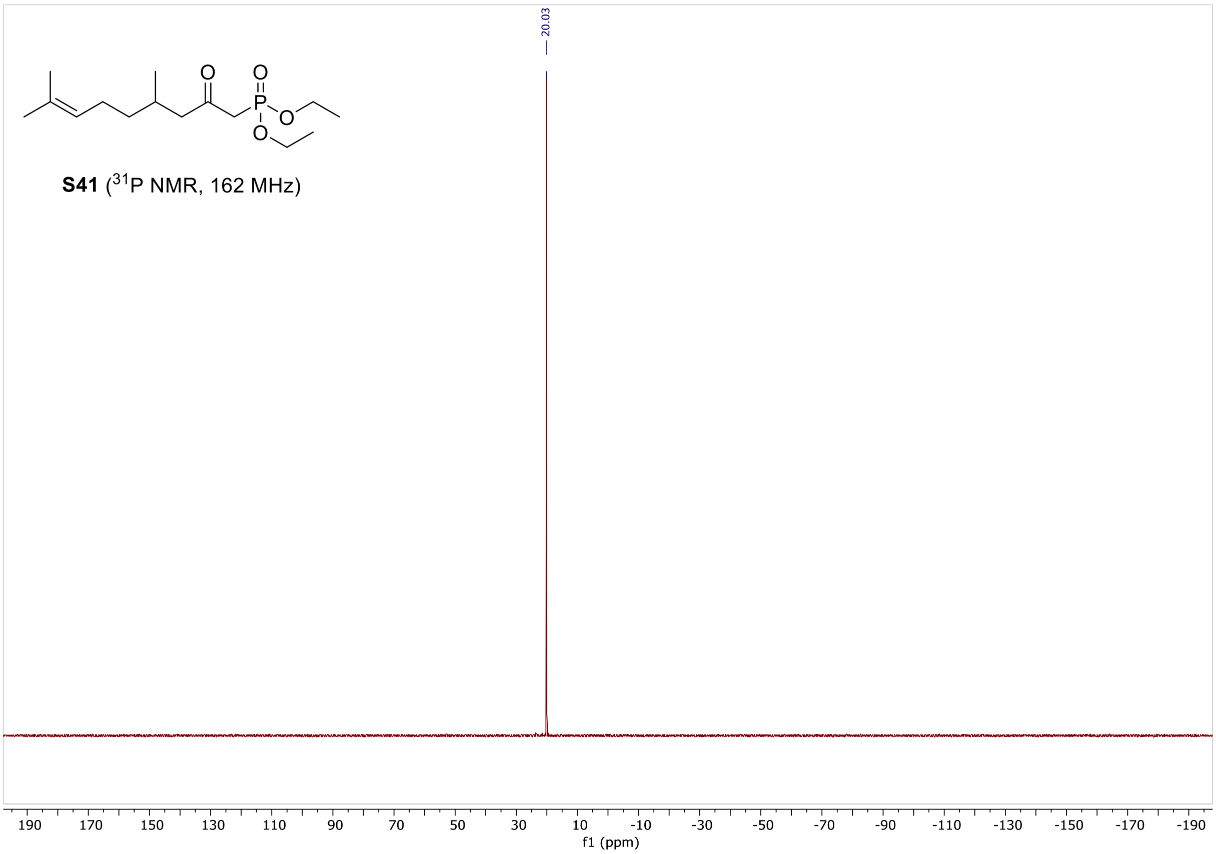
S41 (^{31}P NMR, 162 MHz)
20.03
f1 (ppm)

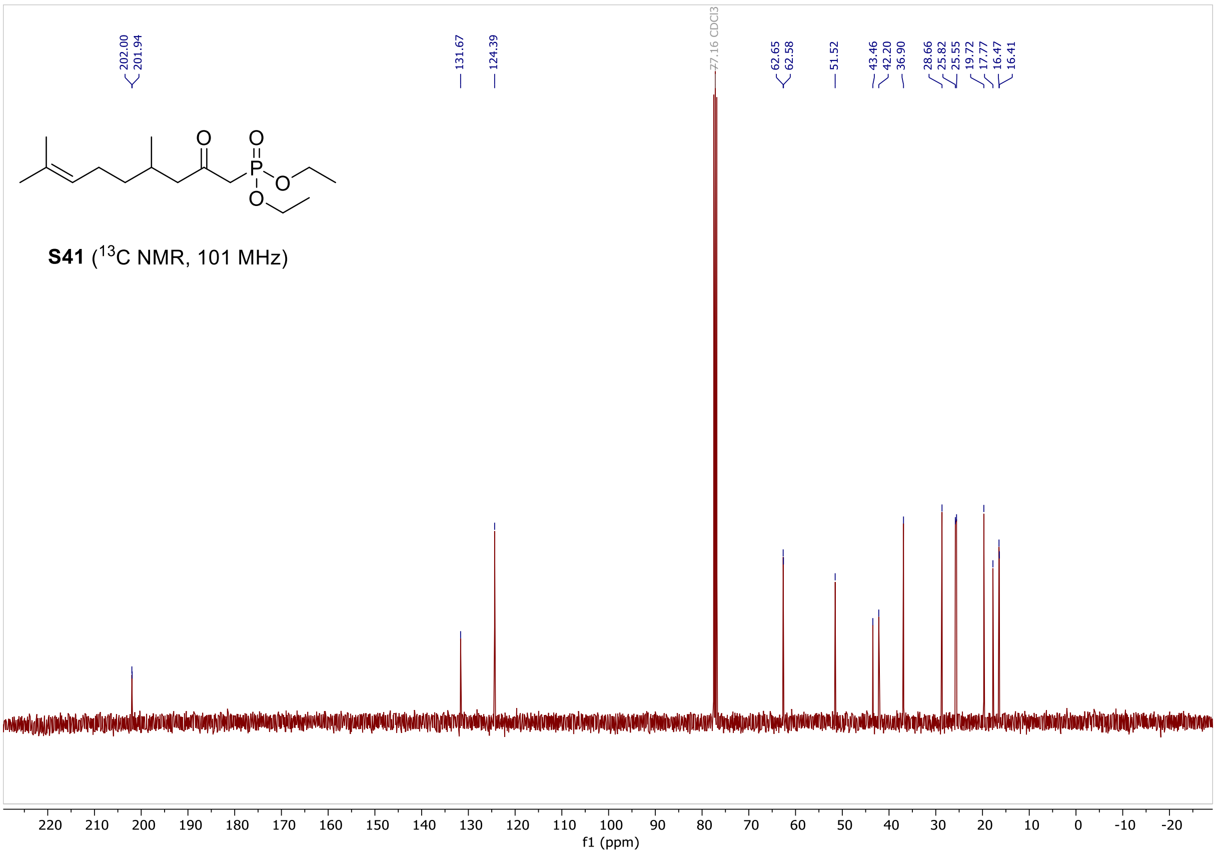

S41 (^{13}C NMR, 101 MHz)

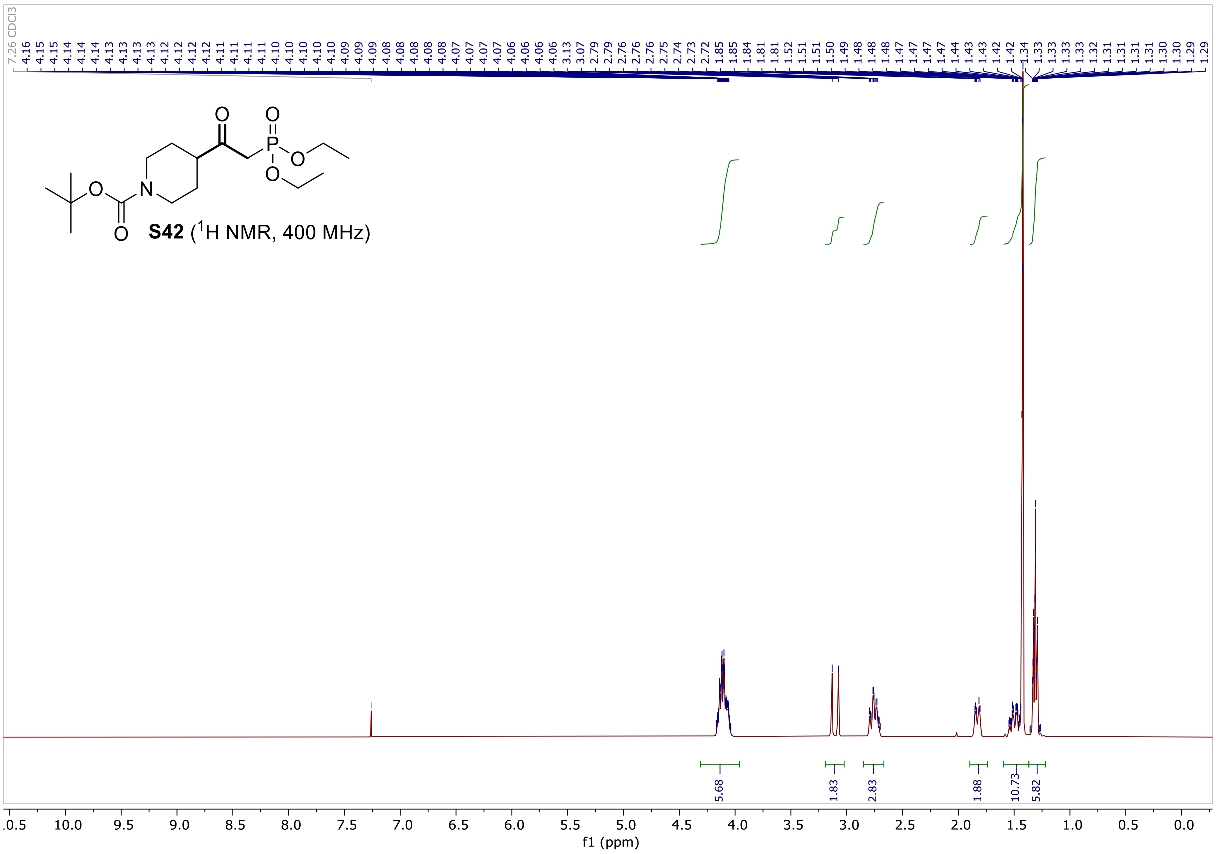
S42 (^{1}H NMR, 400 MHz)
f1 (ppm)

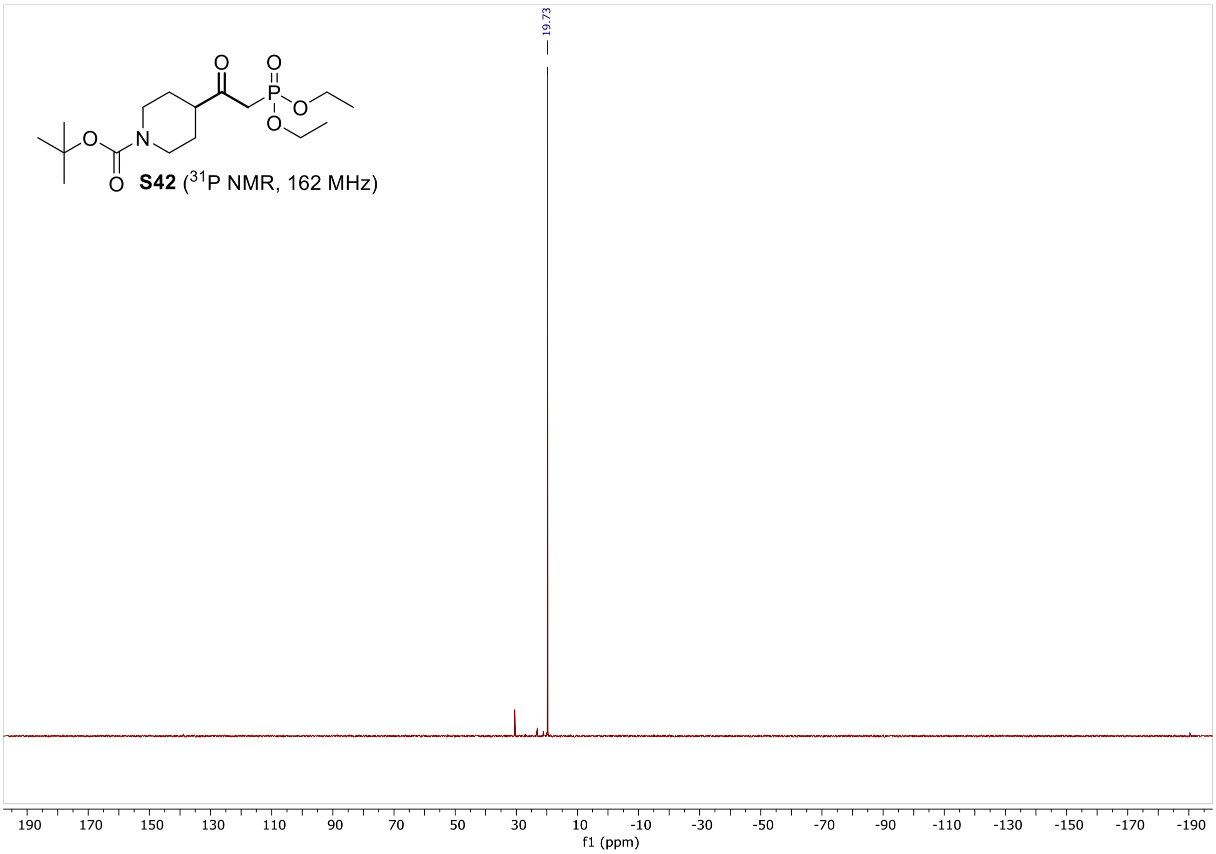
19.73
S42 (^{31}P NMR, 162 MHz)
f1 (ppm)

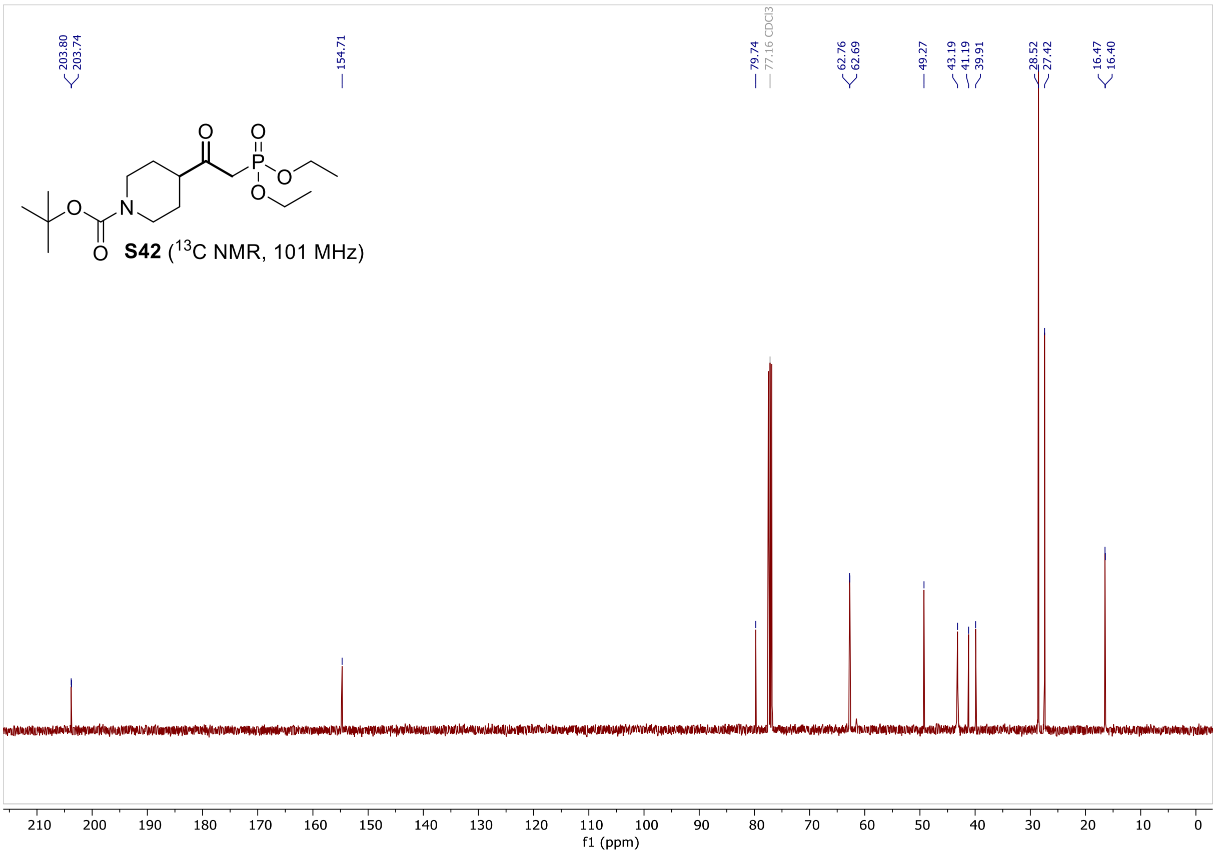
203.80
203.74
154.71
79.74
77.16 CDCl3
62.76
62.69
49.27
43.19
41.19
39.91
28.52
27.42
16.47
16.40
S42 (13C NMR, 101 MHz)
210
200
190
180
170
160
150
140
130
120
110
100
90
80
70
60
50
40
30
20
10
0
f1 (ppm)

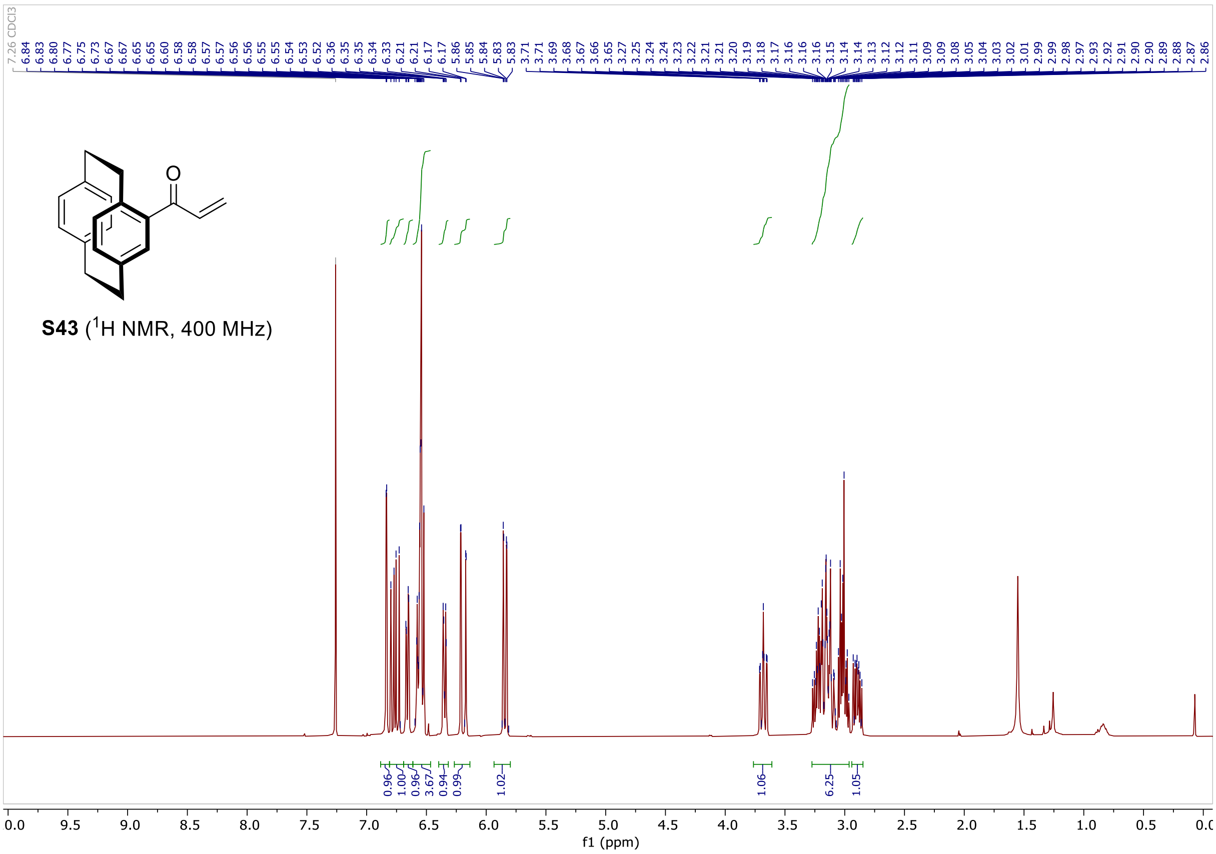
S43 (^{1}H NMR, 400 MHz)
f1 (ppm)

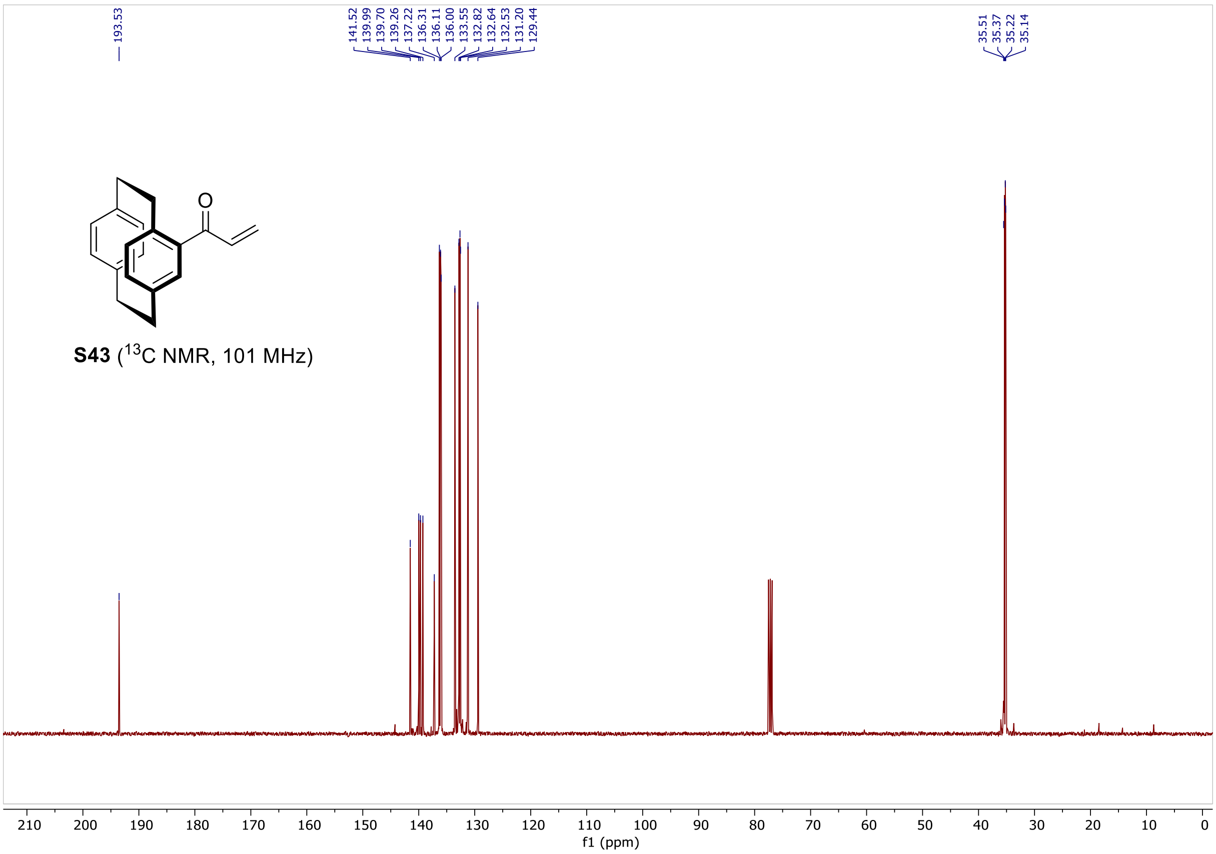
S43 (^{13}C NMR, 101 MHz)
193.53
141.52
139.99
139.70
139.26
137.22
136.31
136.11
136.00
133.55
132.82
132.64
132.53
131.20
129.44
35.51
35.37
35.22
35.14
f1 (ppm)

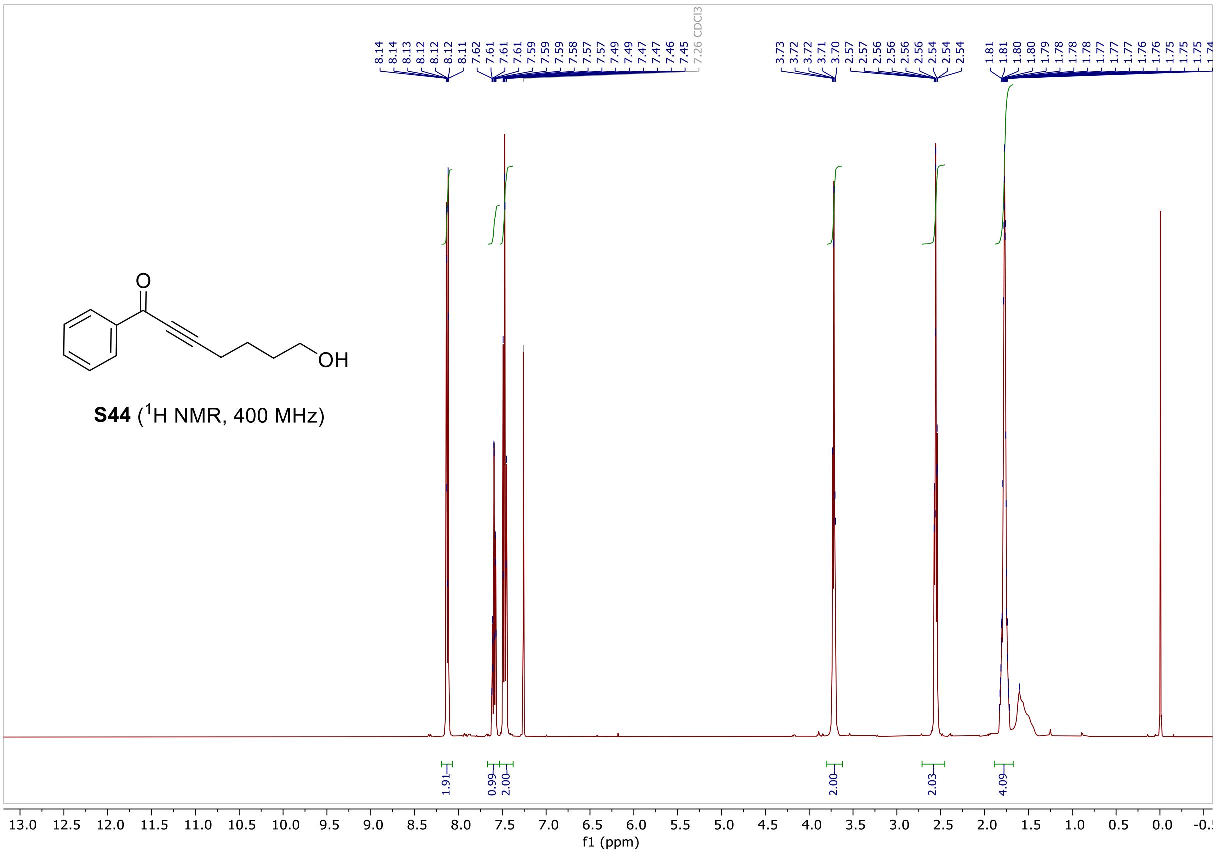
O
OH
S44 (1H NMR, 400 MHz)
7.26 CDCl3
f1 (ppm)

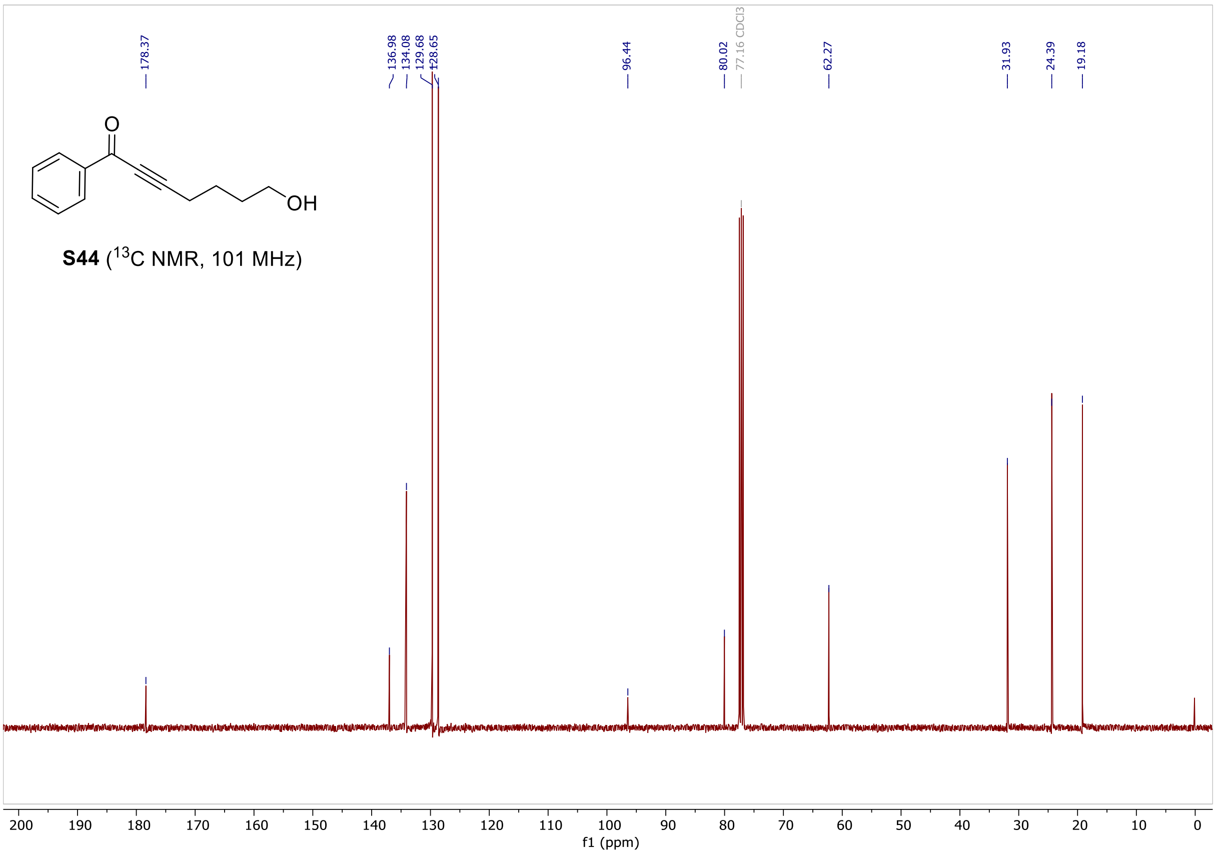
178.37
136.98
134.08
129.68
128.65
96.44
80.02
77.16 CDCl3
62.27
31.93
24.39
19.18
O
OH
S44 (13C NMR, 101 MHz)
f1 (ppm)

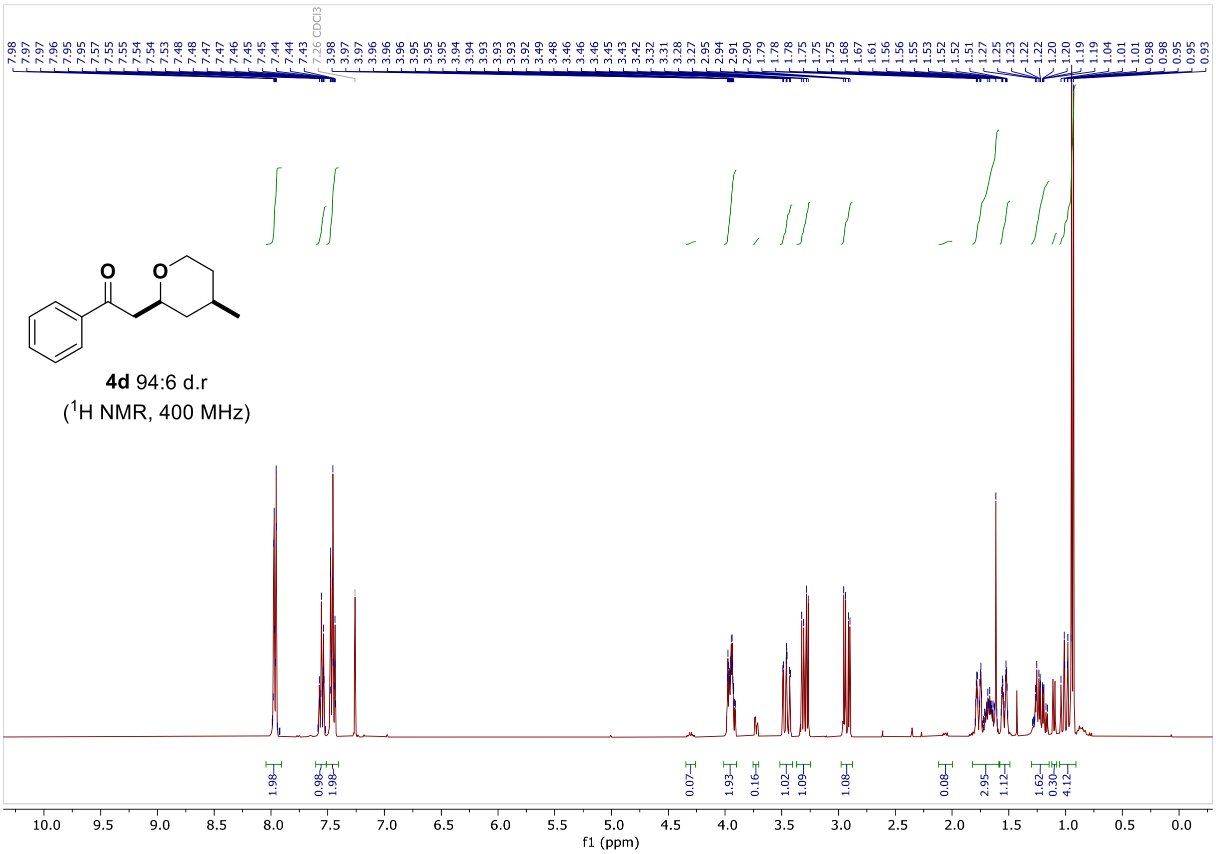
7.98 7.97 7.97 7.96 7.95 7.95 7.57 7.55 7.55 7.54 7.54 7.53 7.48 7.48 7.47 7.47 7.46 7.45 7.45 7.44 7.44 7.43 7.26 CDCl3 3.98 3.97 3.97 3.96 3.96 3.95 3.95 3.95 3.94 3.94 3.93 3.93 3.93 3.92 3.49 3.48 3.46 3.46 3.45 3.43 3.42 3.32 3.31 3.28 3.27 2.95 2.94 2.91 2.90 1.79 1.78 1.78 1.75 1.75 1.75 1.68 1.67 1.61 1.56 1.56 1.55 1.53 1.52 1.52 1.51 1.27 1.25 1.23 1.22 1.22 1.20 1.20 1.19 1.19 1.04 1.01 1.01 0.98 0.98 0.95 0.93
O
O
4d 94:6 d.r
(1H NMR, 400 MHz)
1.98 0.98 1.98 0.07 1.93 0.16 1.02 1.09 1.08 0.08 2.95 1.12 1.62 0.30 4.12
10.0 9.5 9.0 8.5 8.0 7.5 7.0 6.5 6.0 5.5 5.0 4.5 4.0 3.5 3.0 2.5 2.0 1.5 1.0 0.5 0.0
f1 (ppm)

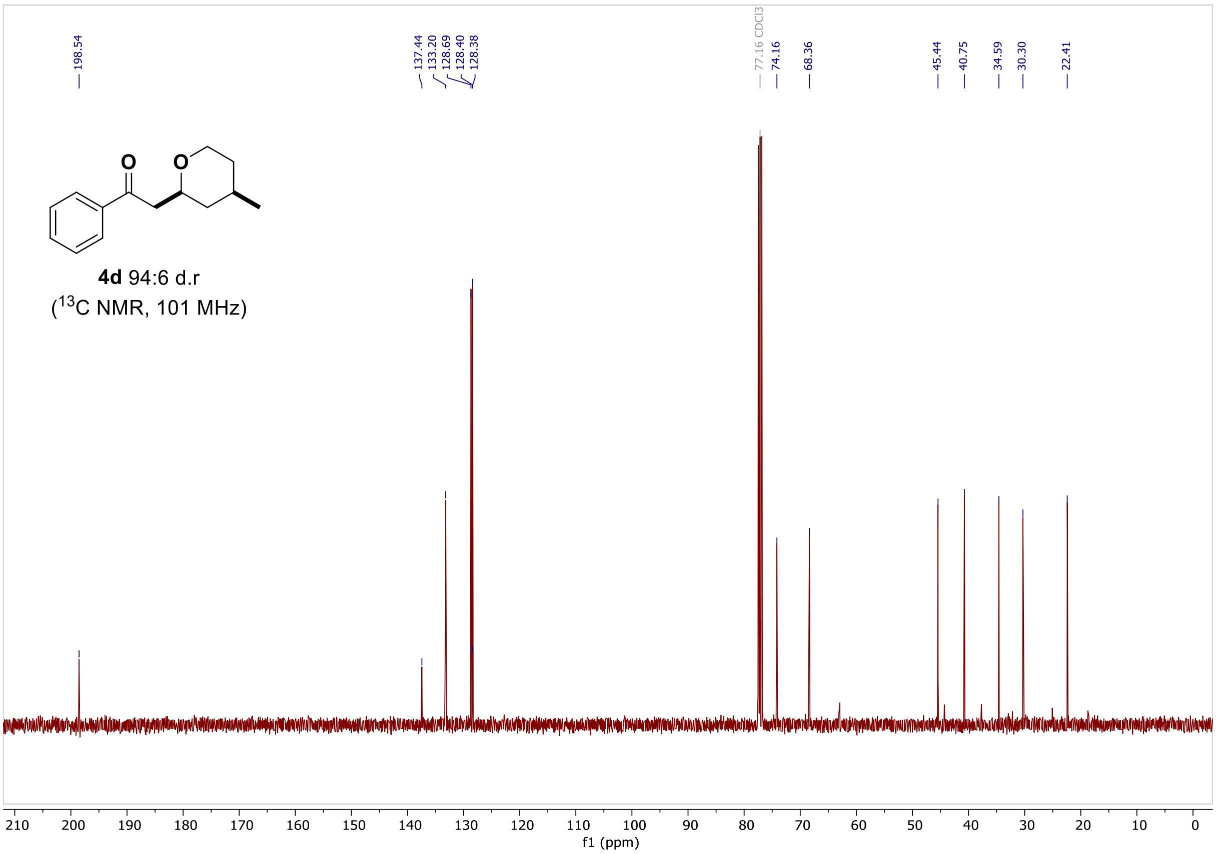
198.54
137.44 133.20 128.69 128.40 128.38
77.16 CDCl3
74.16
68.36
45.44 40.75 34.59 30.30 22.41
O
O
4d 94:6 d.r
(13C NMR, 101 MHz)
210 200 190 180 170 160 150 140 130 120 110 100 90 80 70 60 50 40 30 20 10 0
f1 (ppm)

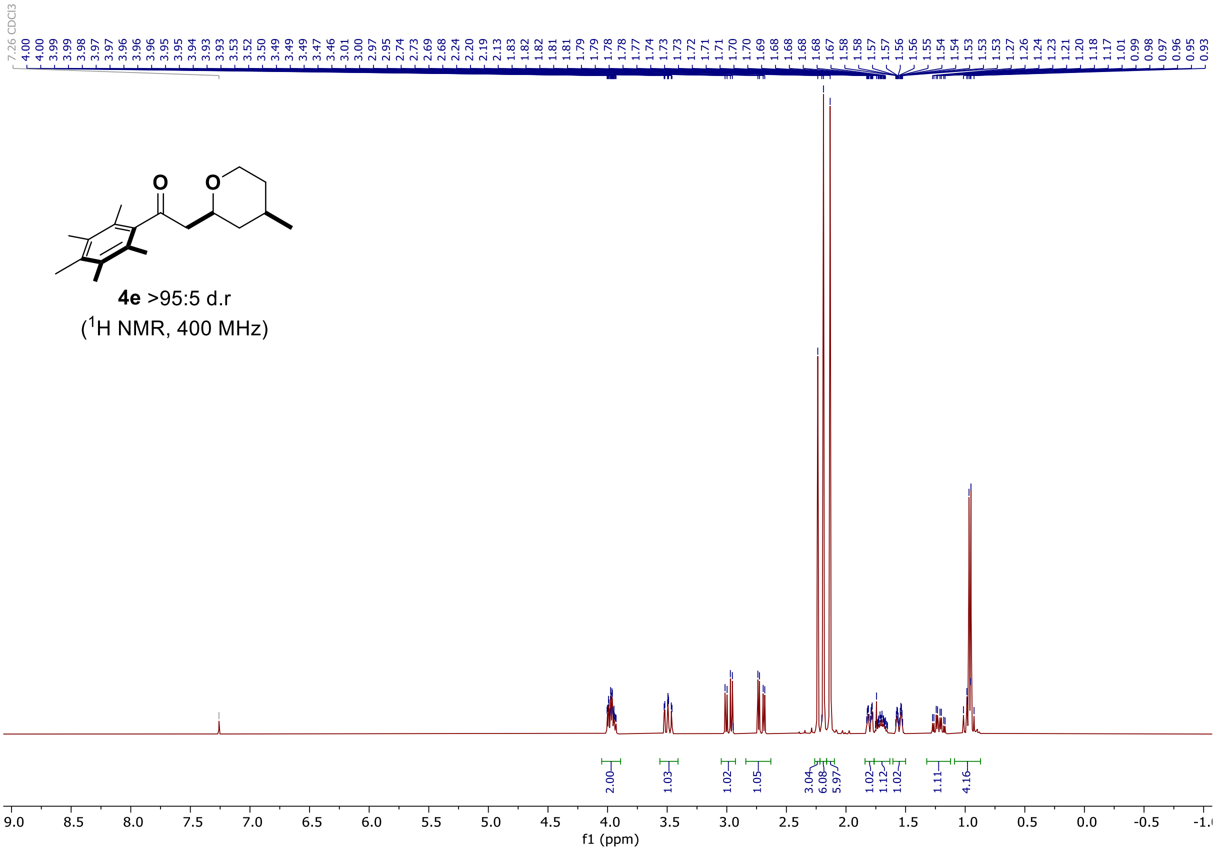
7.26 CDCl3
4.00 4.00 3.99 3.98 3.97 3.97 3.96 3.96 3.95 3.95 3.94 3.93 3.93 3.53 3.52 3.50 3.49 3.49 3.47 3.46 3.01 3.00 2.97 2.95 2.74 2.73 2.69 2.68 2.24 2.20 2.19 2.13 1.83 1.82 1.82 1.81 1.81 1.79 1.79 1.78 1.78 1.77 1.74 1.73 1.73 1.72 1.71 1.71 1.70 1.70 1.69 1.68 1.68 1.68 1.67 1.58 1.58 1.57 1.57 1.56 1.56 1.55 1.54 1.54 1.53 1.53 1.53 1.27 1.26 1.24 1.23 1.21 1.20 1.18 1.17 1.01 0.99 0.98 0.97 0.96 0.95 0.93
O
O
4e >95:5 d.r
(^{1}H NMR, 400 MHz)
2.00 1.03 1.02 1.05 3.04 6.08 5.97 1.02 1.12 1.02 1.11 4.16
f1 (ppm)

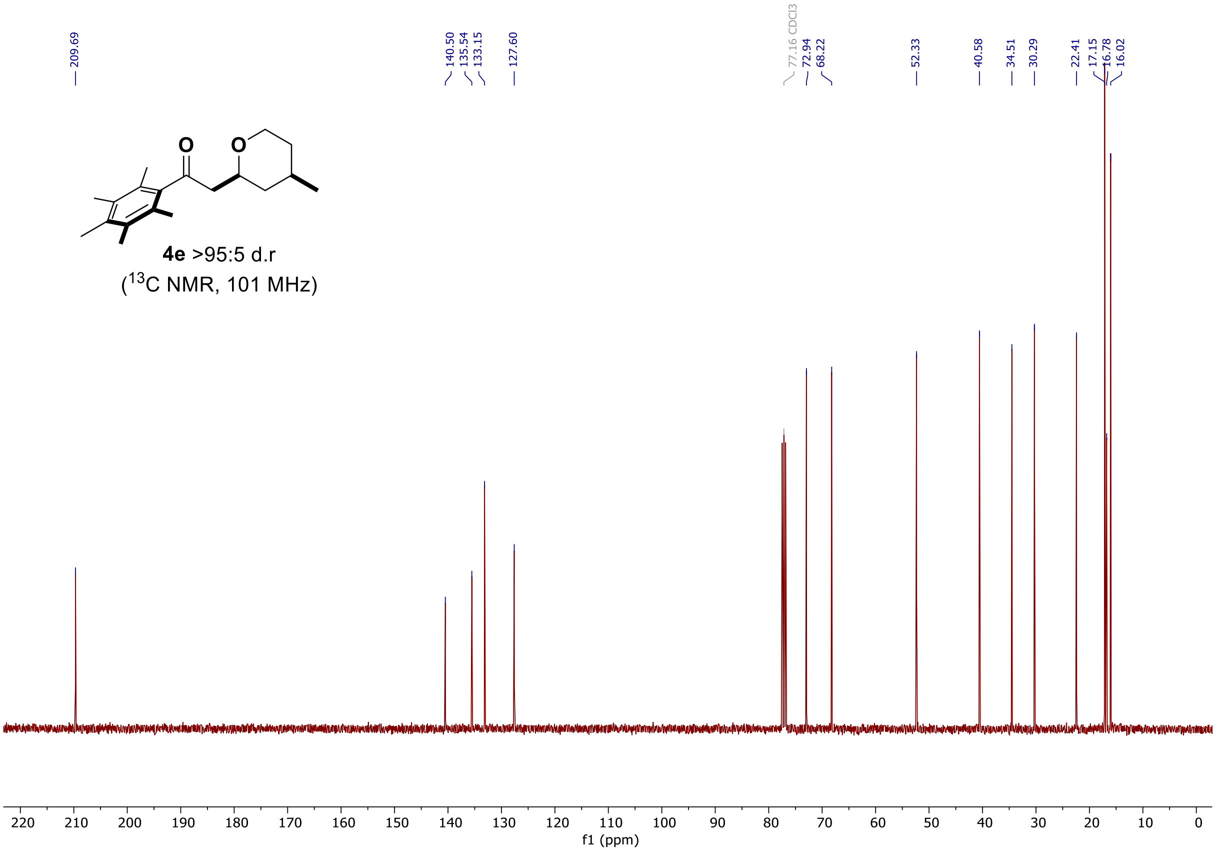
209.69
140.50 135.54 133.15 127.60
77.16 CDCl3 72.94 68.22
52.33 40.58 34.51 30.29 22.41 17.15 16.78 16.02
O
O
4e >95:5 d.r
(^{13}C NMR, 101 MHz)
f1 (ppm)

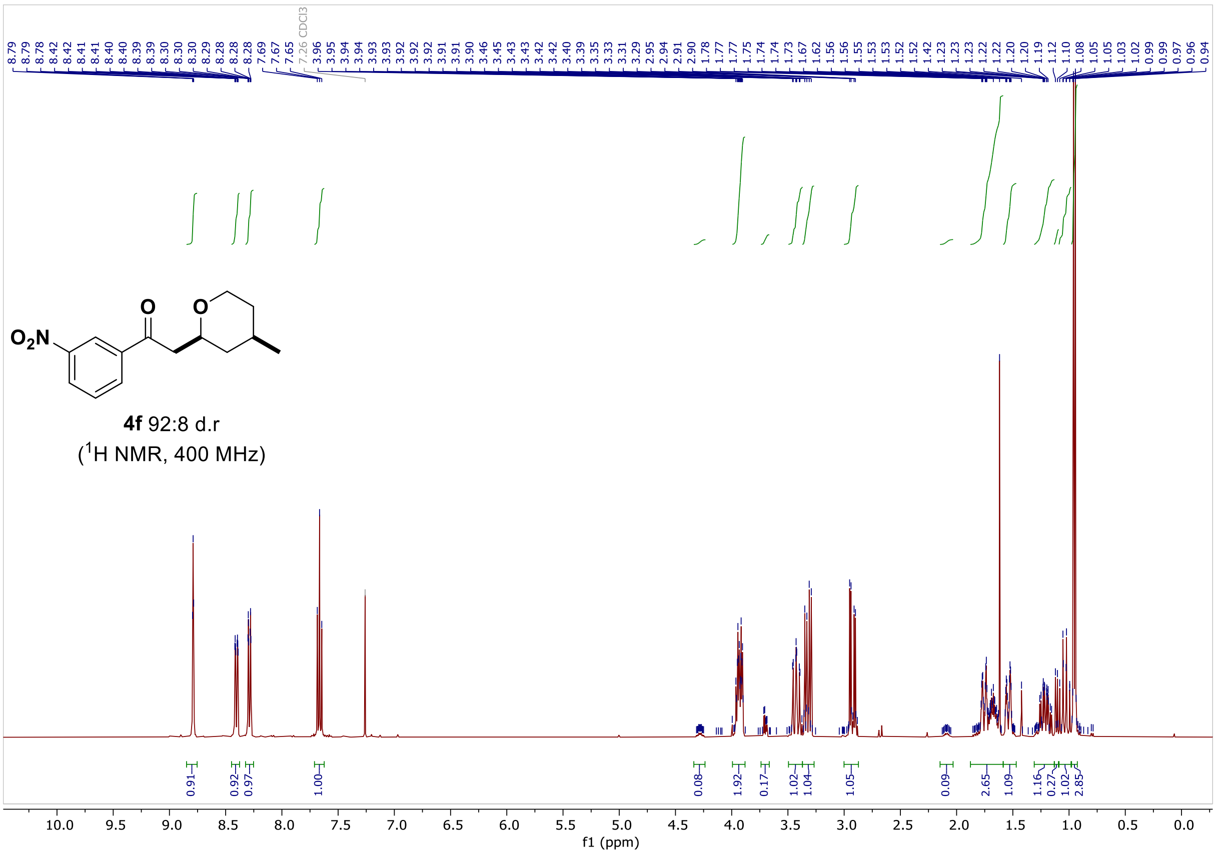
O_2N
O
O
4f 92:8 d.r
(^{1}H NMR, 400 MHz)
8.79 8.79 8.78 8.42 8.42 8.41 8.41 8.40 8.40 8.39 8.39 8.30 8.30 8.29 8.28 8.28 8.28 7.69 7.67 7.65 7.26 CDCl3 3.96 3.95 3.94 3.94 3.93 3.93 3.92 3.92 3.92 3.91 3.91 3.90 3.46 3.45 3.43 3.43 3.42 3.40 3.39 3.35 3.33 3.31 3.29 2.95 2.94 2.91 2.90 1.78 1.77 1.75 1.74 1.74 1.73 1.67 1.62 1.56 1.56 1.55 1.53 1.53 1.52 1.52 1.42 1.23 1.23 1.23 1.22 1.22 1.20 1.19 1.12 1.10 1.08 1.05 1.05 1.03 1.02 0.99 0.99 0.97 0.96 0.94
0.91 0.92 0.97 1.00 0.08 1.92 0.17 1.02 1.04 1.05 0.09 2.65 1.09 1.16 0.27 1.02 2.85
10.0 9.5 9.0 8.5 8.0 7.5 7.0 6.5 6.0 5.5 5.0 4.5 4.0 3.5 3.0 2.5 2.0 1.5 1.0 0.5 0.0
f1 (ppm)

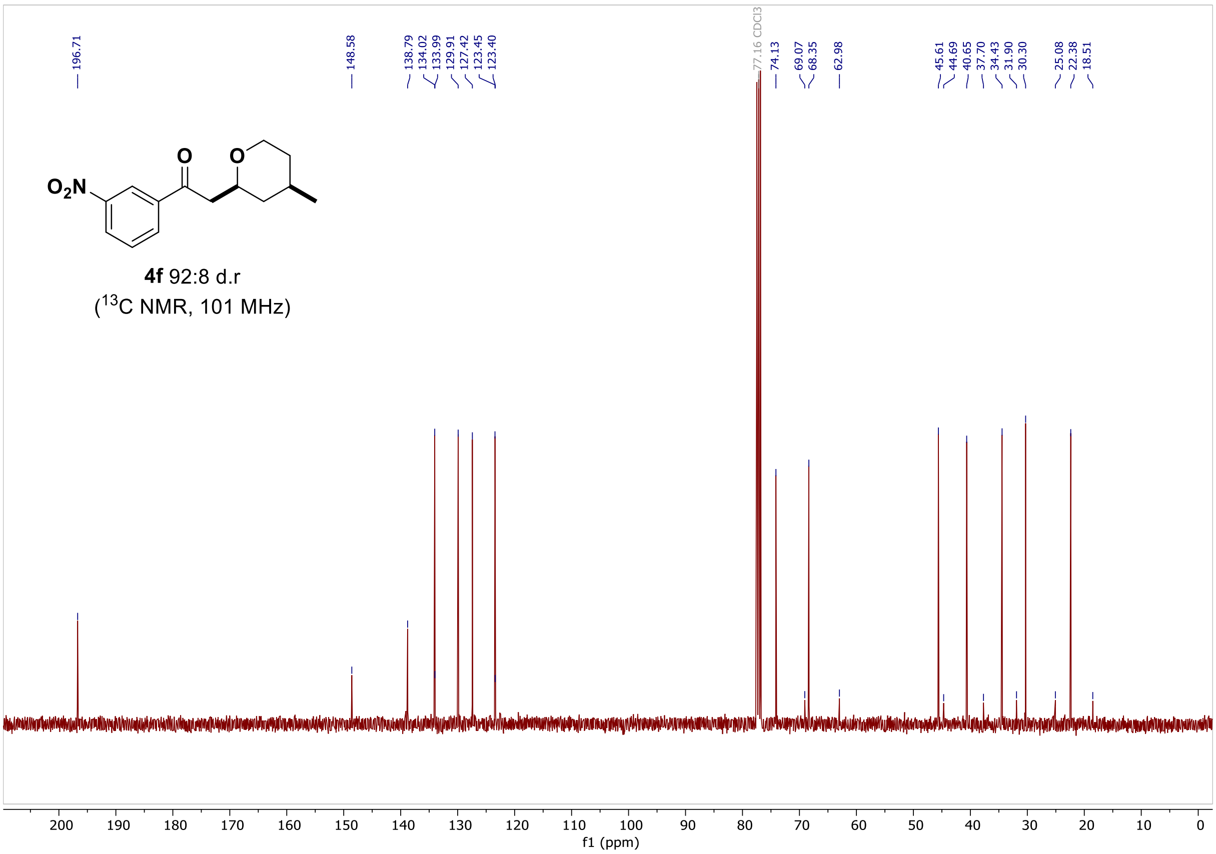
O_2N
O
O
4f 92:8 d.r
(^{13}C NMR, 101 MHz)
196.71 148.58 138.79 134.02 133.99 129.91 127.42 123.45 123.40 77.16 CDCl3 74.13 69.07 68.35 62.98 45.61 44.69 40.65 37.70 34.43 31.90 30.30 25.08 22.38 18.51
200 190 180 170 160 150 140 130 120 110 100 90 80 70 60 50 40 30 20 10 0
f1 (ppm)

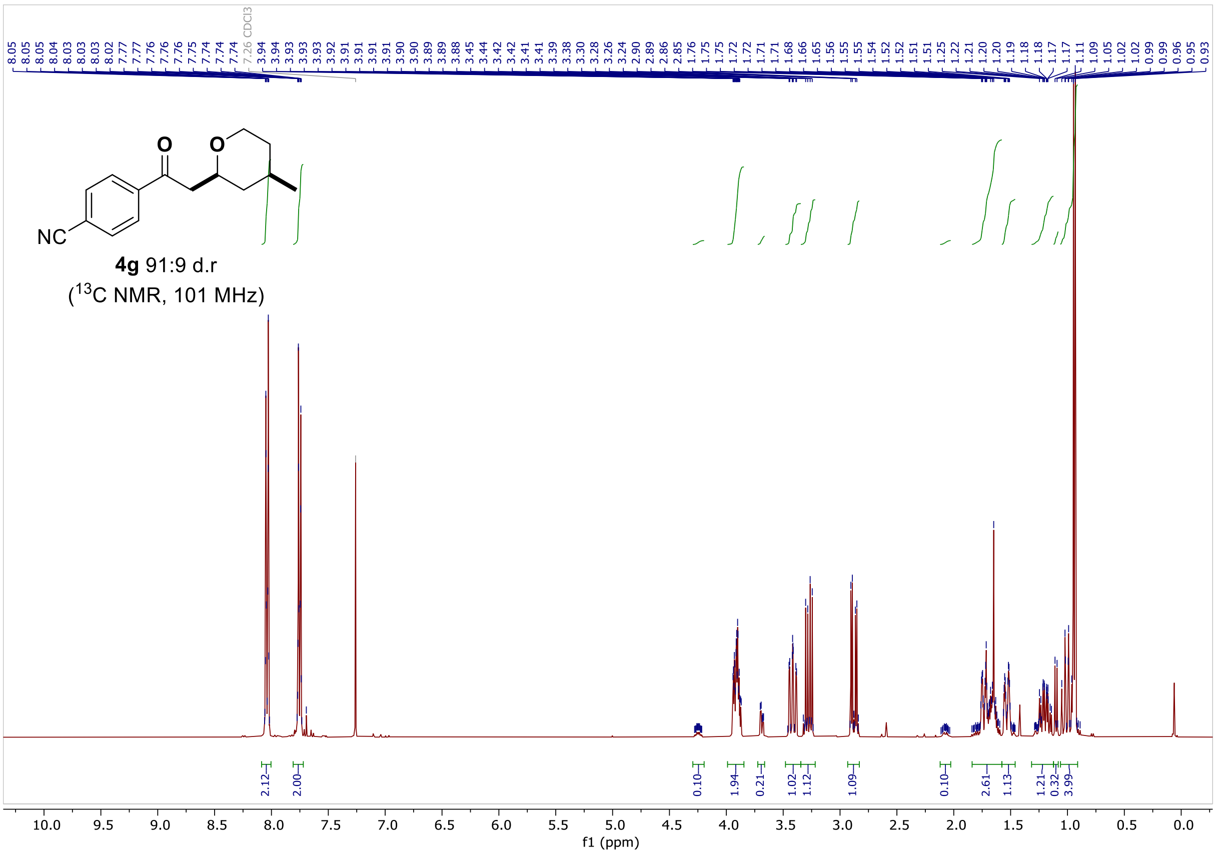
8.05 8.05 8.05 8.04 8.03 8.03 8.03 8.02 7.77 7.77 7.76 7.76 7.76 7.75 7.74 7.74 7.74
7.26 CDCl3
3.94 3.94 3.93 3.93 3.93 3.92 3.91 3.91 3.91 3.90 3.90 3.89 3.89 3.88 3.45 3.44 3.42 3.42 3.41 3.41 3.39 3.38 3.30 3.28 3.26 3.24 2.90 2.89 2.86 2.85 1.76 1.75 1.75 1.72 1.72 1.71 1.71 1.68 1.66 1.65 1.56 1.55 1.55 1.54 1.52 1.52 1.51 1.51 1.25 1.22 1.21 1.20 1.20 1.19 1.18 1.18 1.17 1.17 1.11 1.09 1.05 1.02 1.02 0.99 0.99 0.96 0.95 0.93
O
O
NC
4g 91:9 d.r
(13C NMR, 101 MHz)
2.12
2.00
0.10
1.94
0.21
1.02
1.12
1.09
0.10
2.61
1.13
1.21
0.32
3.99
10.0 9.5 9.0 8.5 8.0 7.5 7.0 6.5 6.0 5.5 5.0 4.5 4.0 3.5 3.0 2.5 2.0 1.5 1.0 0.5 0.0
f1 (ppm)

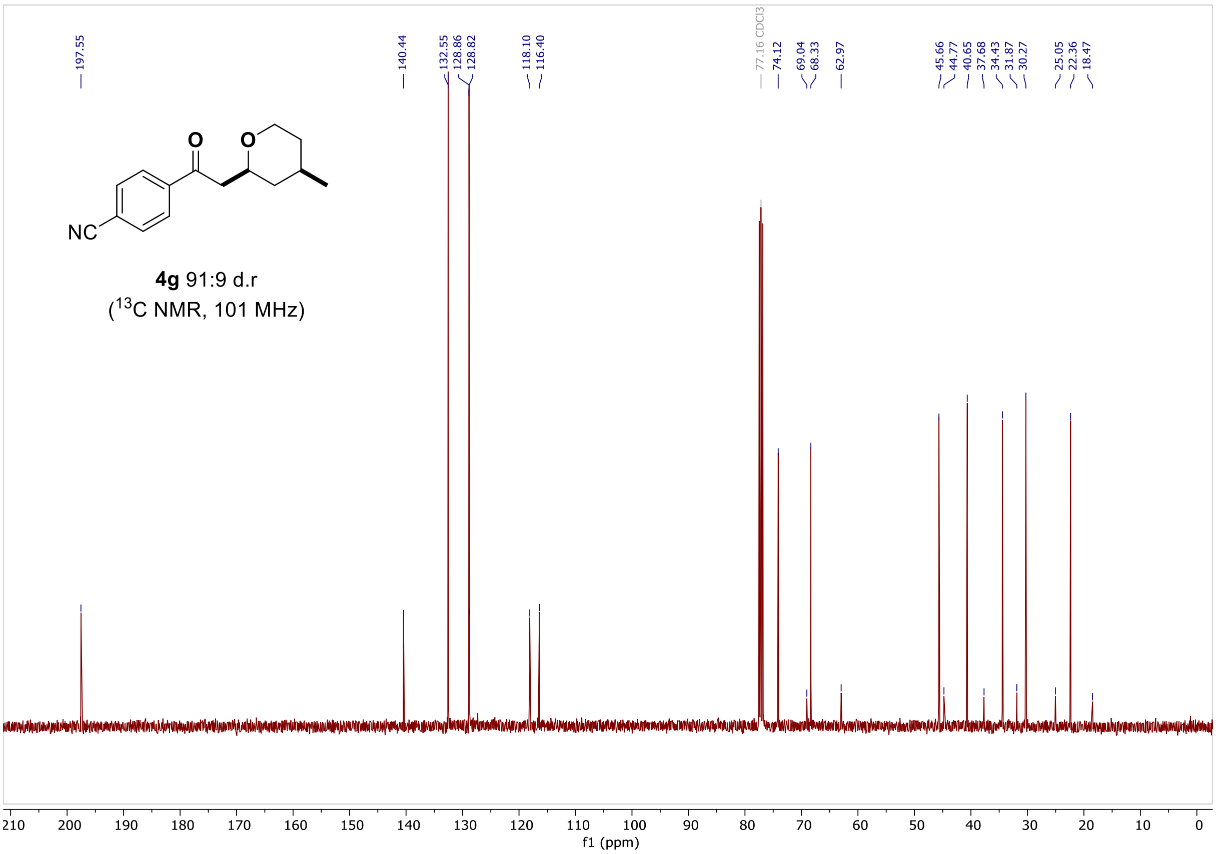
197.55
140.44
132.55
128.86
128.82
118.10
116.40
77.16 CDCl3
74.12
69.04
68.33
62.97
45.66
44.77
40.65
37.68
34.43
31.87
30.27
25.05
22.36
18.47
O
O
NC
4g 91:9 d.r
(13C NMR, 101 MHz)
210 200 190 180 170 160 150 140 130 120 110 100 90 80 70 60 50 40 30 20 10 0
f1 (ppm)

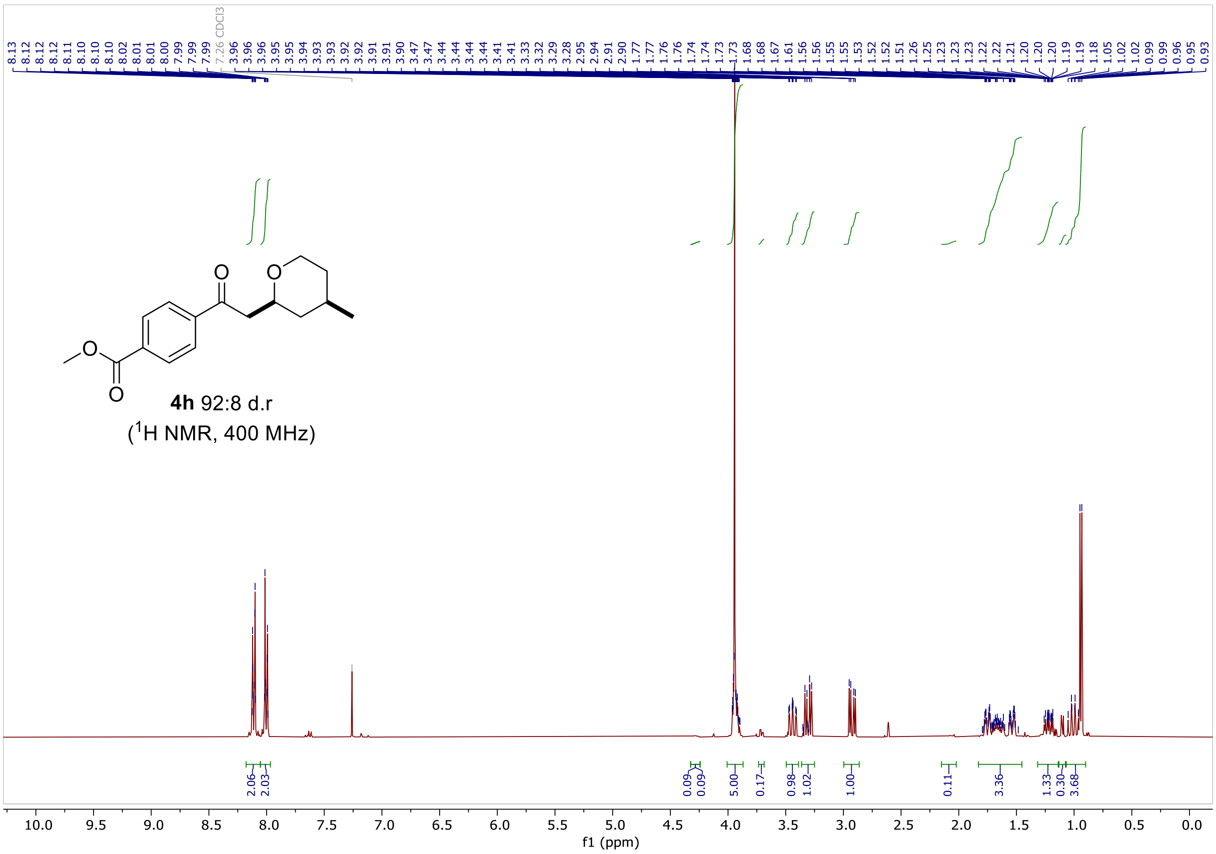
4h 92:8 d.r
(1H NMR, 400 MHz)
f1 (ppm)

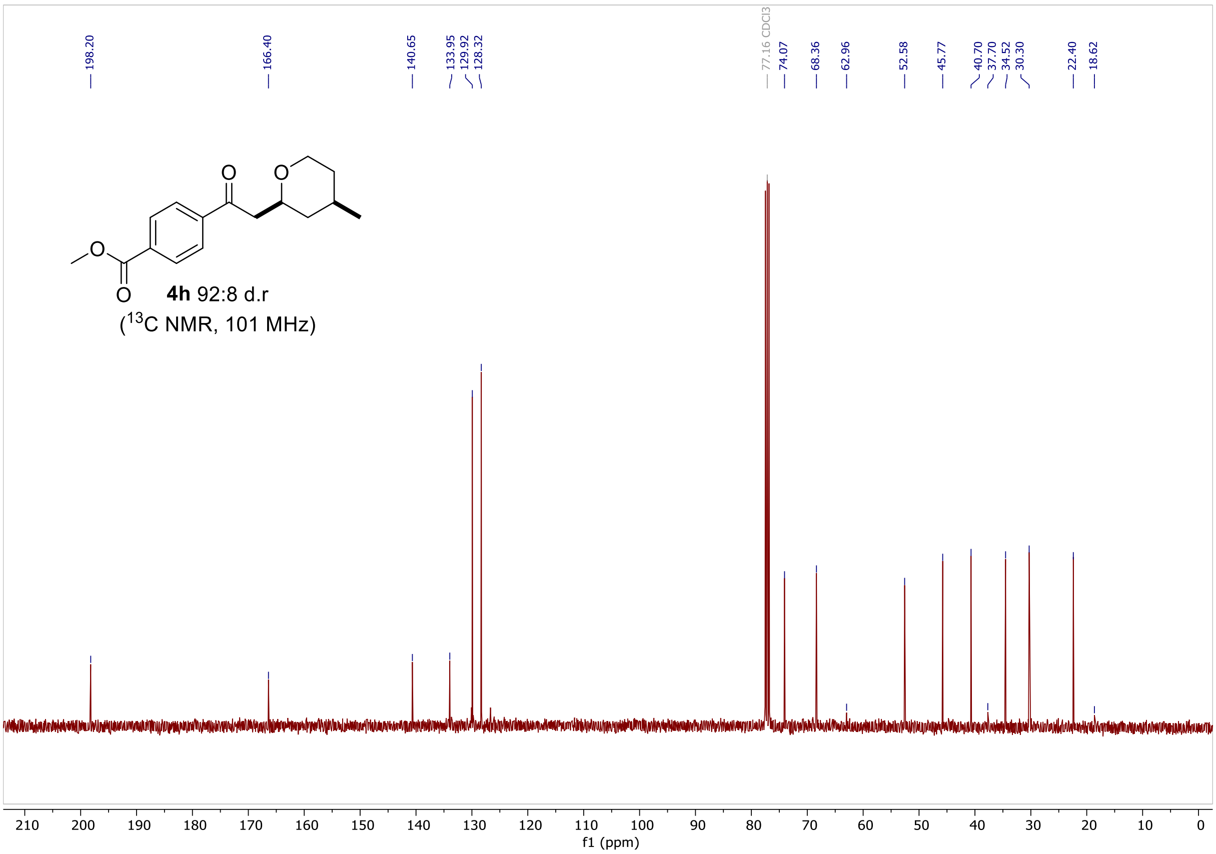
4h 92:8 d.r
(13C NMR, 101 MHz)
198.20
166.40
140.65
133.95
129.92
128.32
77.16 CDCl3
74.07
68.36
62.96
52.58
45.77
40.70
37.70
34.52
30.30
22.40
18.62
f1 (ppm)

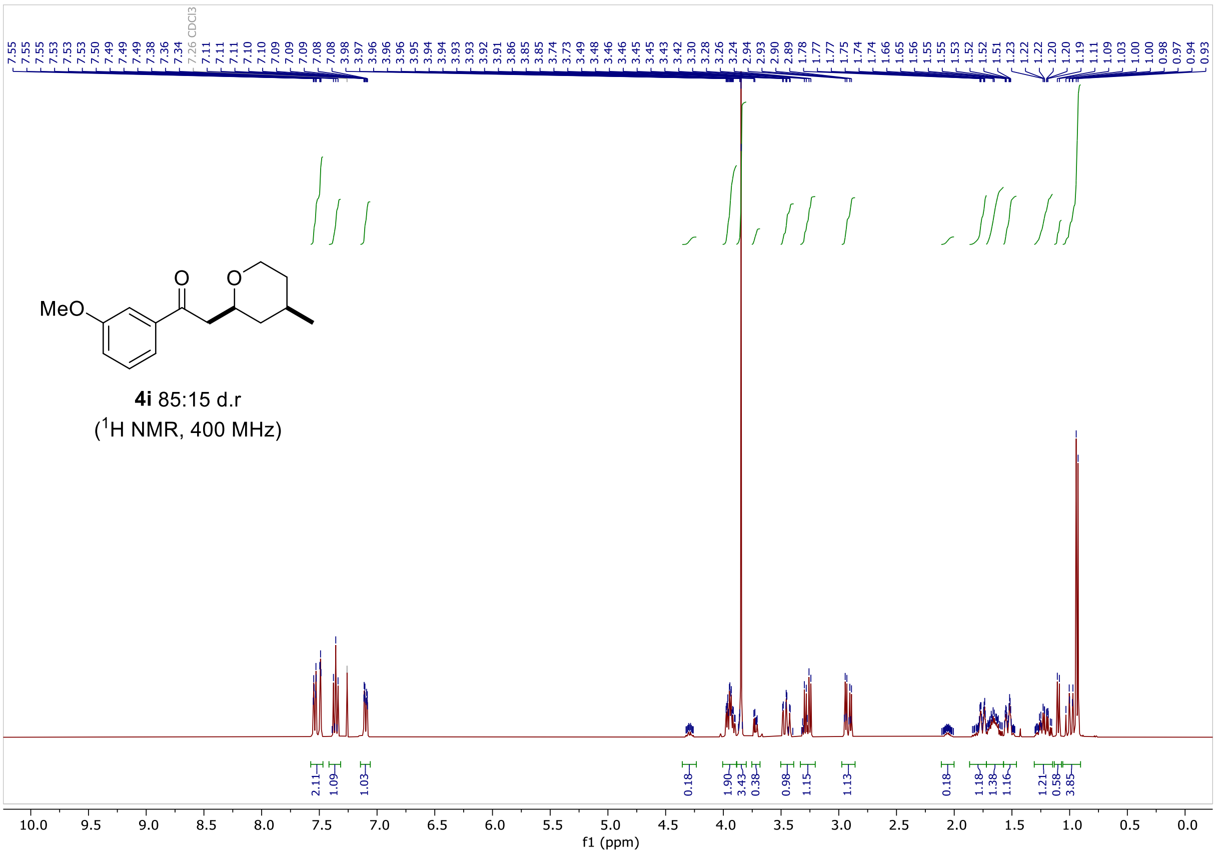
MeO
O
O
4i 85:15 d.r
(^{1}H NMR, 400 MHz)
7.26 CDCl3
2.11
1.09
1.03
0.18
1.90
3.43
0.38
0.98
1.15
1.13
0.18
1.18
1.38
1.16
1.21
0.58
3.85
f1 (ppm)

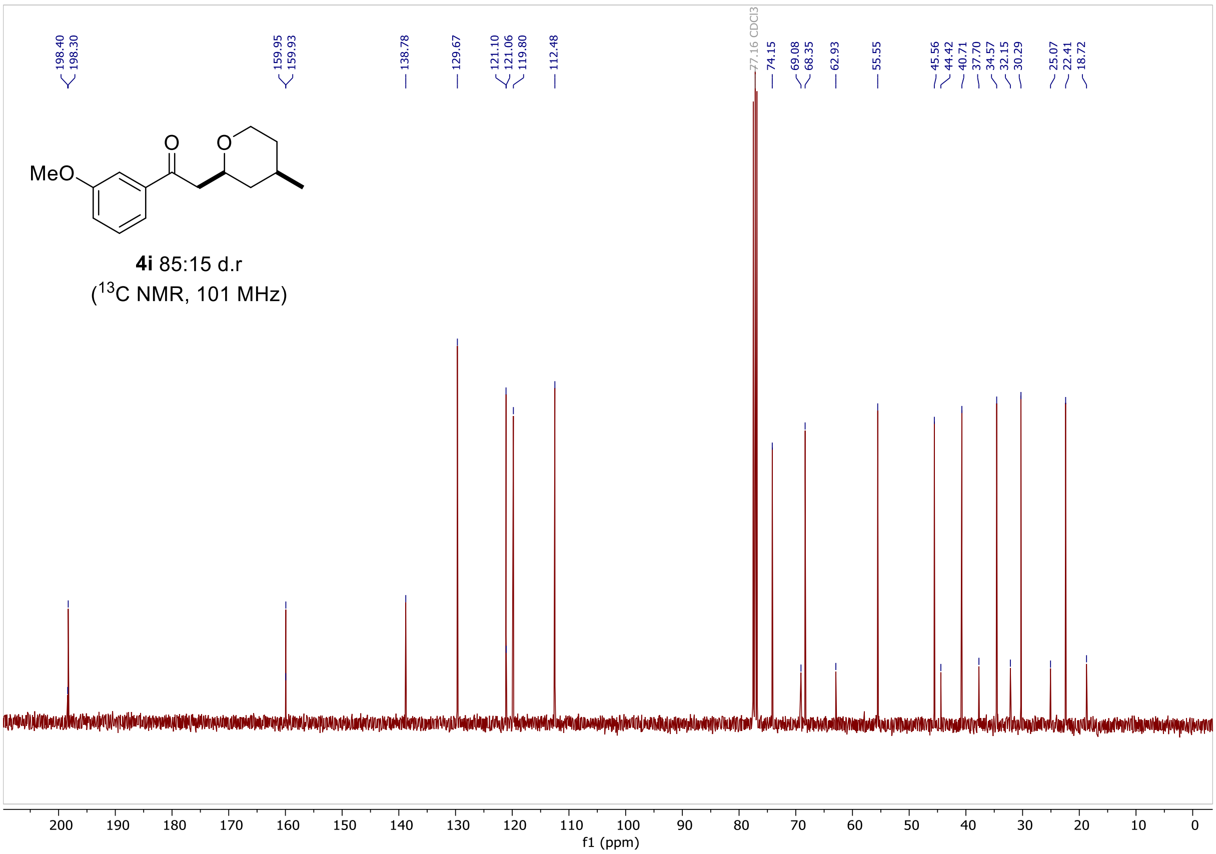
198.40
198.30
159.95
159.93
138.78
129.67
121.10
121.06
119.80
112.48
77.16 CDCl3
74.15
69.08
68.35
62.93
55.55
45.56
44.42
40.71
37.70
34.57
32.15
30.29
25.07
22.41
18.72
MeO
O
O
4i 85:15 d.r
(^{13}C NMR, 101 MHz)
f1 (ppm)

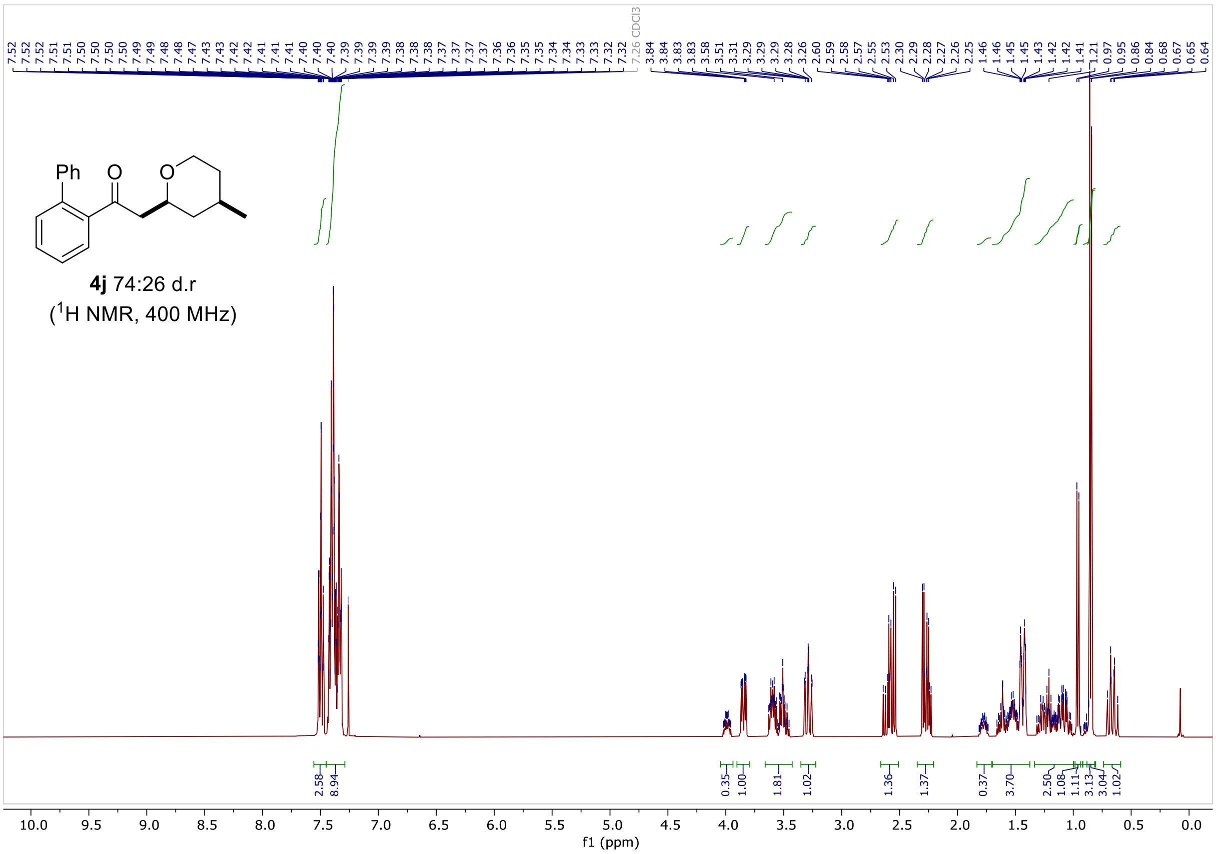
Ph
O
O
4j 74:26 d.r
(1H NMR, 400 MHz)
f1 (ppm)
7.26 CDCl3

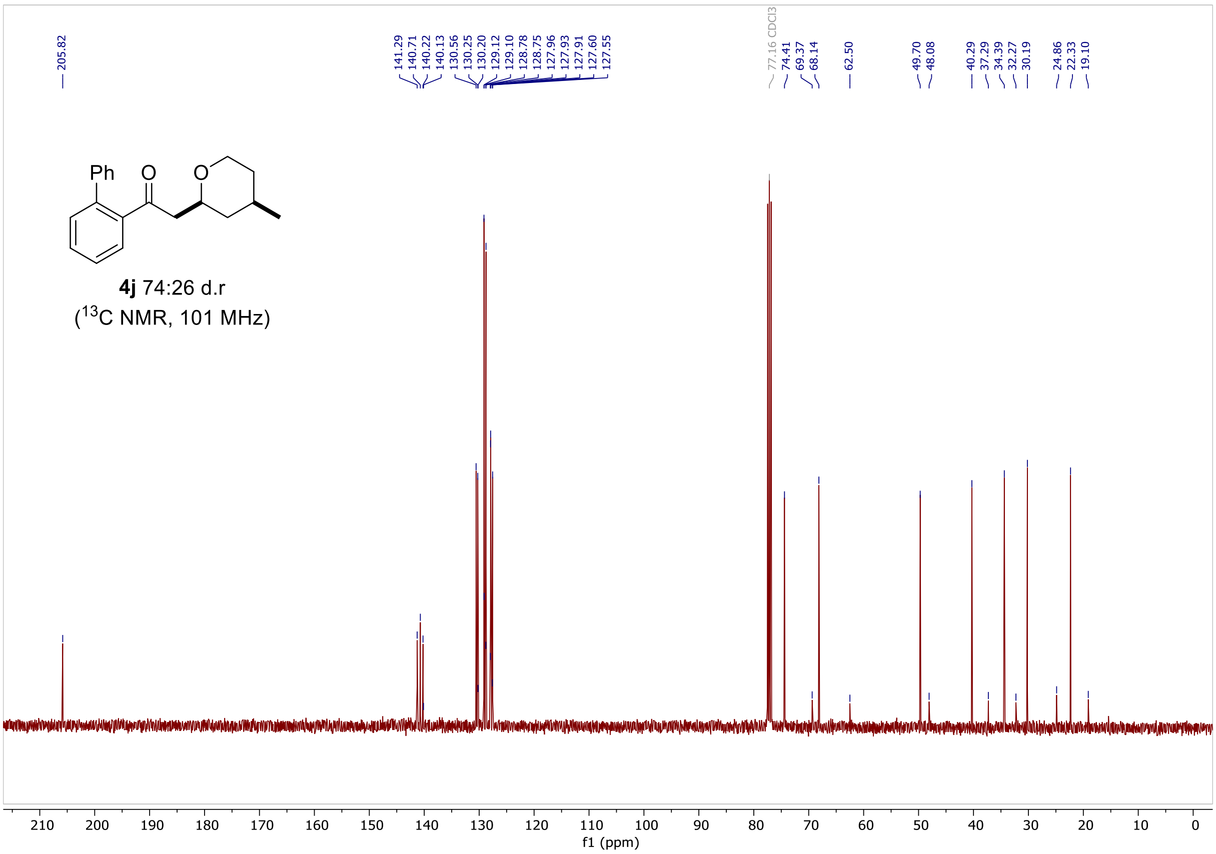
Ph
O
O
4j 74:26 d.r
(13C NMR, 101 MHz)
f1 (ppm)
205.82
141.29
140.71
140.22
140.13
130.56
130.25
130.20
129.12
129.10
128.78
128.75
127.96
127.93
127.91
127.60
127.55
77.16 CDCl3
74.41
69.37
68.14
62.50
49.70
48.08
40.29
37.29
34.39
32.27
30.19
24.86
22.33
19.10

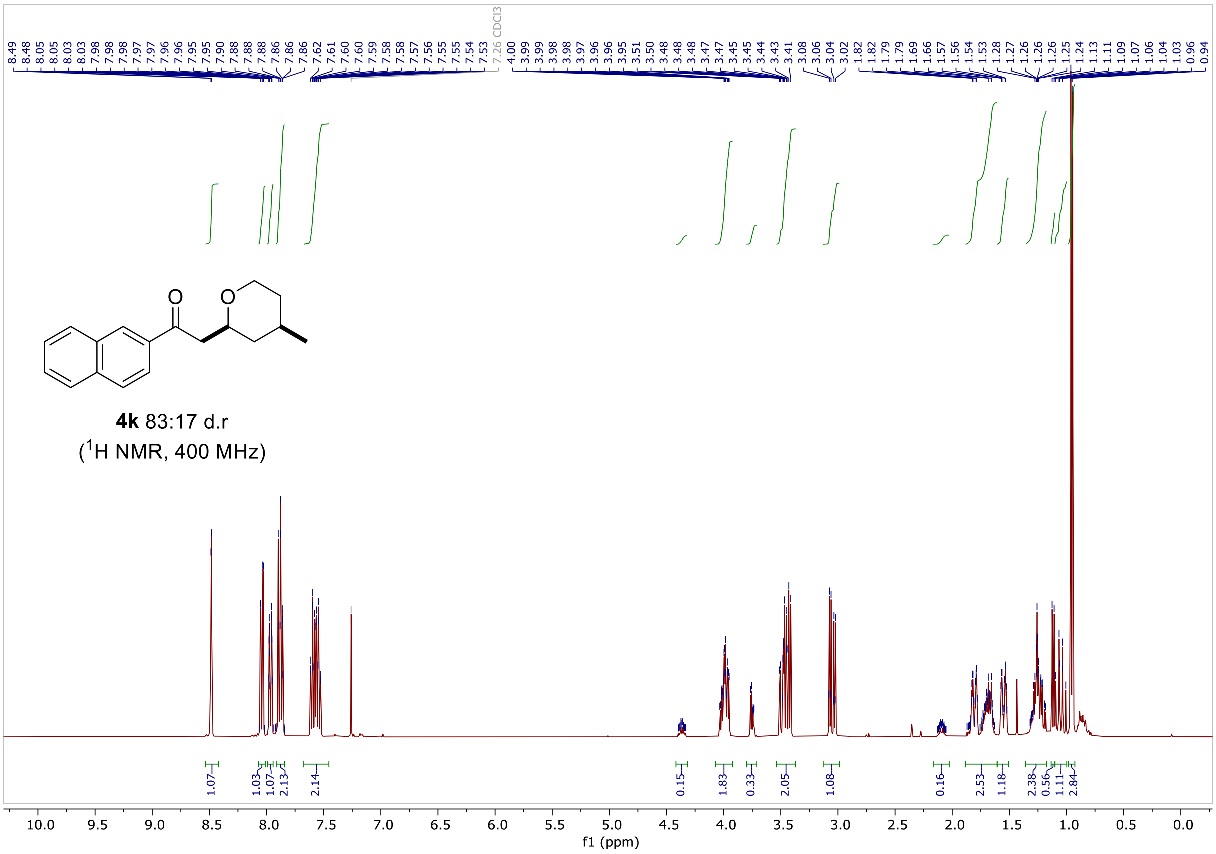

4k 83:17 d.r
(^{1}H NMR, 400 MHz)
f1 (ppm)

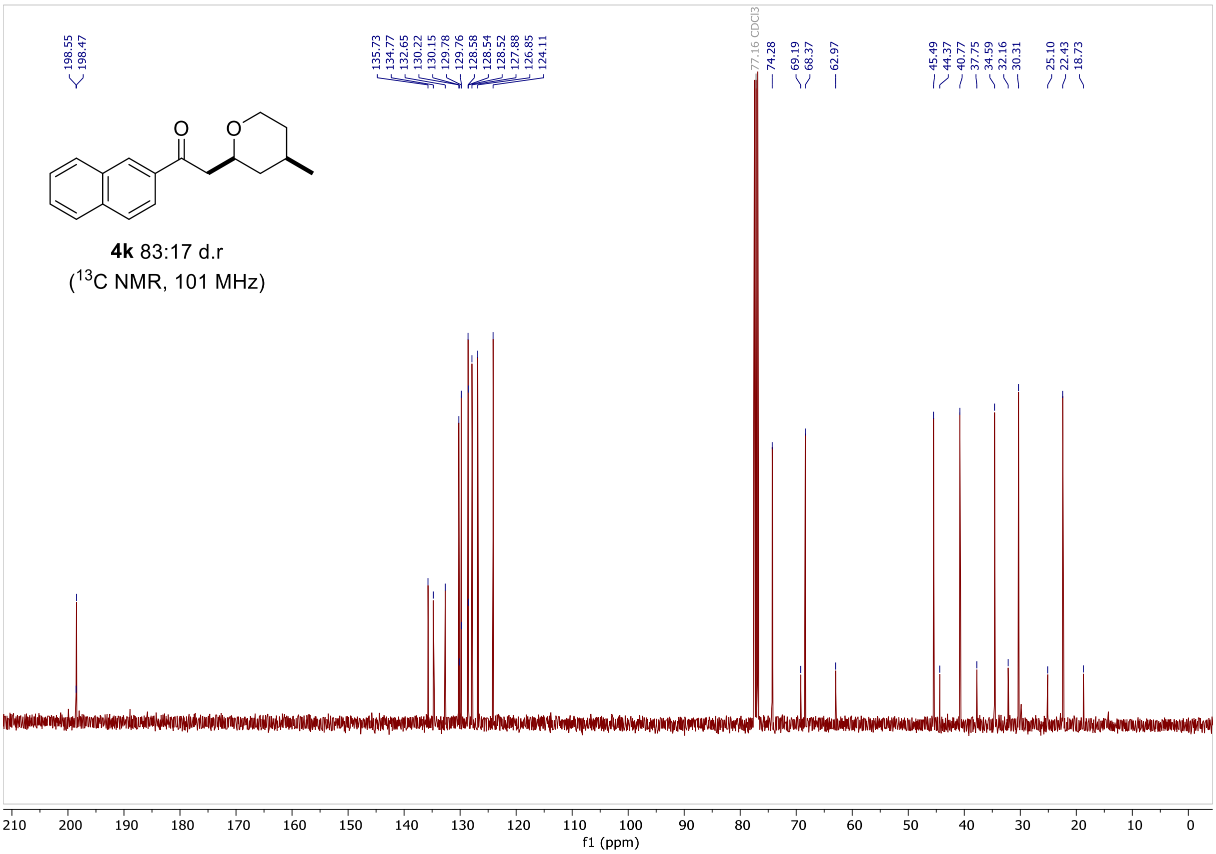

4k 83:17 d.r
(^{13}C NMR, 101 MHz)
198.55
198.47
135.73
134.77
132.65
130.22
130.15
129.78
129.76
128.58
128.54
128.52
127.88
126.85
124.11
77.16 CDCl3
74.28
69.19
68.37
62.97
45.49
44.37
40.77
37.75
34.59
32.16
30.31
25.10
22.43
18.73
f1 (ppm)

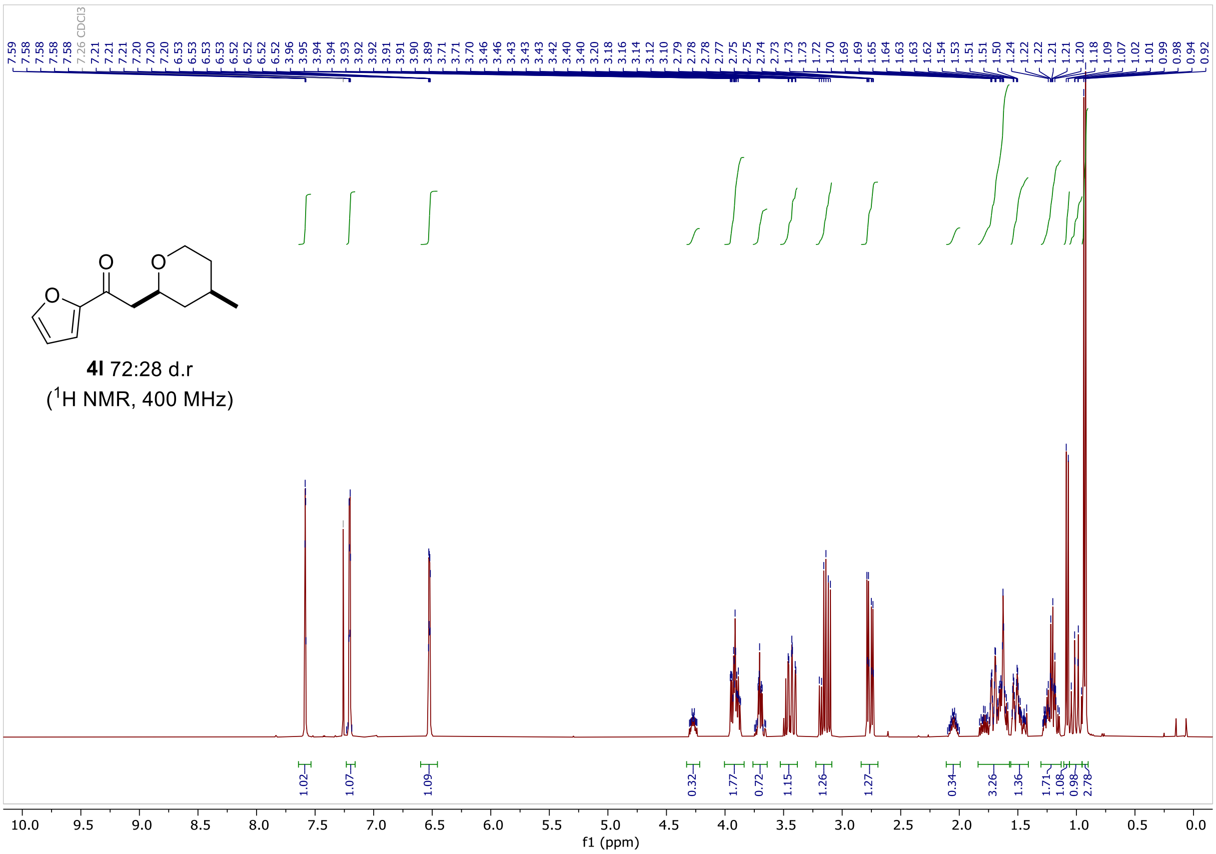
7.59 7.58 7.58 7.58 7.58 7.26 CDCl3 7.21 7.21 7.21 7.20 7.20 6.53 6.53 6.53 6.53 6.52 6.52 6.52 3.96 3.95 3.94 3.94 3.93 3.92 3.92 3.91 3.91 3.90 3.89 3.71 3.71 3.70 3.46 3.43 3.43 3.43 3.42 3.40 3.40 3.20 3.18 3.16 3.14 3.12 3.10 2.79 2.78 2.77 2.75 2.75 2.74 2.73 1.73 1.73 1.72 1.70 1.69 1.69 1.65 1.64 1.63 1.63 1.62 1.54 1.53 1.51 1.51 1.50 1.24 1.22 1.22 1.21 1.21 1.20 1.18 1.09 1.07 1.02 1.01 0.99 0.94 0.92
O
O
O
4l 72:28 d.r
(1H NMR, 400 MHz)
1.02 1.07 1.09 0.32 1.77 0.72 1.15 1.26 1.27 0.34 3.26 1.36 1.71 1.08 0.98 2.78
10.0 9.5 9.0 8.5 8.0 7.5 7.0 6.5 6.0 5.5 5.0 4.5 4.0 3.5 3.0 2.5 2.0 1.5 1.0 0.5 0.0
f1 (ppm)

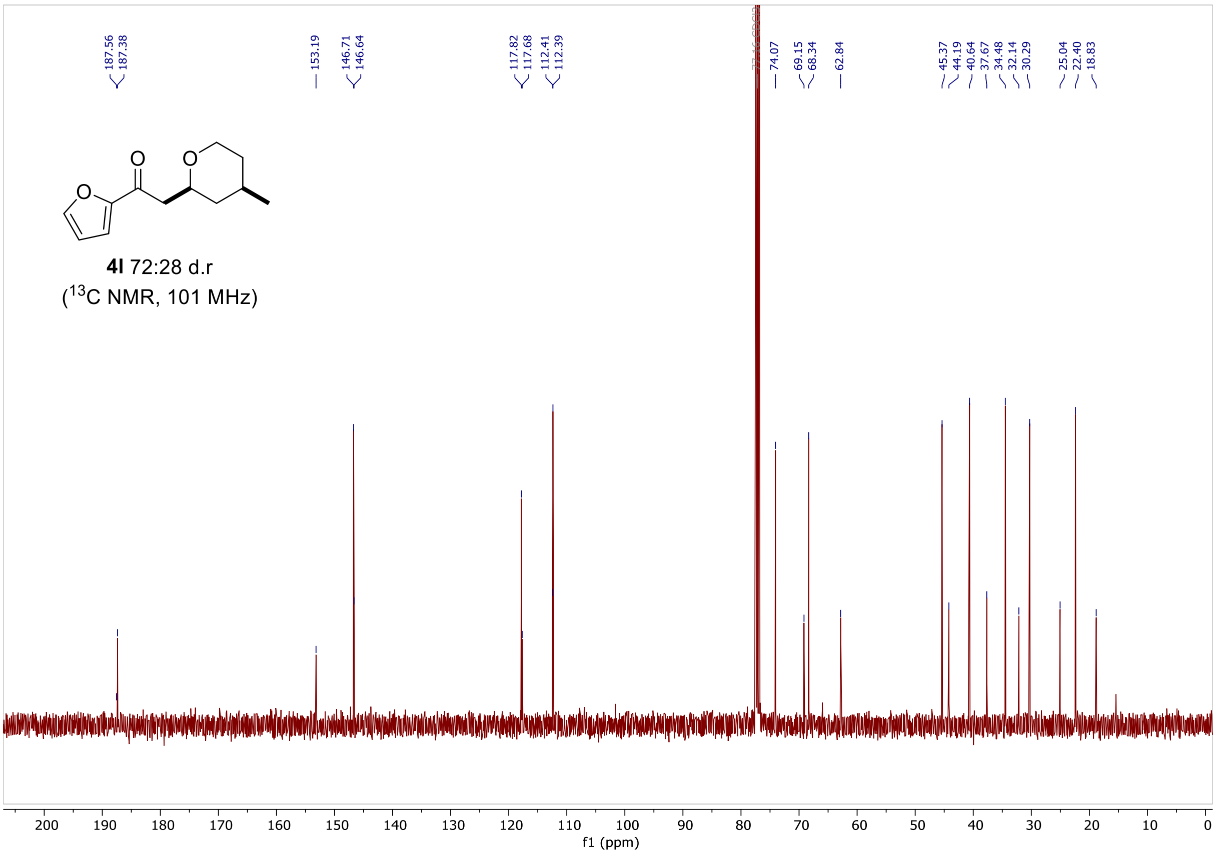
187.56 187.38 153.19 146.71 146.64 117.82 117.68 112.41 112.39 77.16 CDCl3 74.07 69.15 68.34 62.84 45.37 44.19 40.64 37.67 34.48 32.14 30.29 25.04 22.40 18.83
O
O
O
4l 72:28 d.r
(13C NMR, 101 MHz)
200 190 180 170 160 150 140 130 120 110 100 90 80 70 60 50 40 30 20 10 0
f1 (ppm)

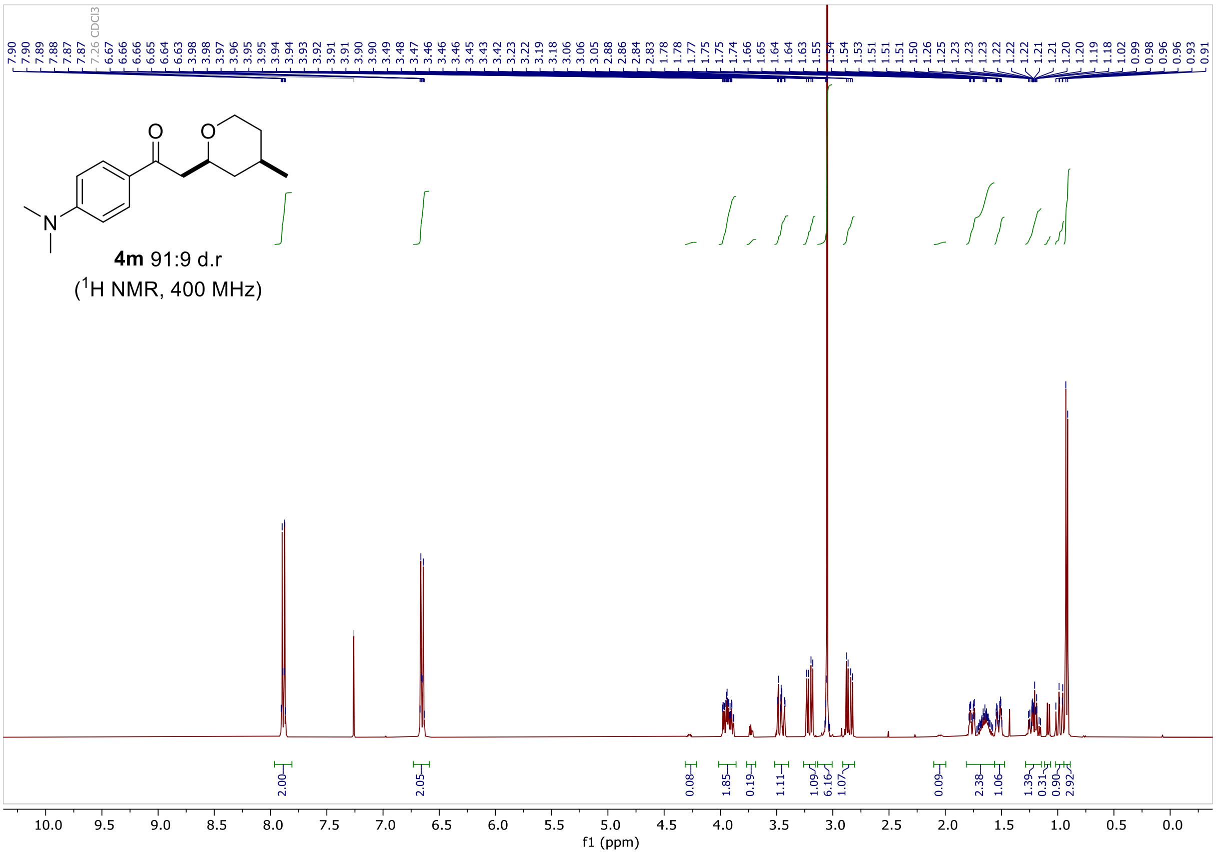
7.90 7.90 7.89 7.88 7.87 7.87 7.26 CDCl3 6.67 6.66 6.66 6.65 6.64 6.63 3.98 3.98 3.97 3.96 3.95 3.95 3.94 3.94 3.93 3.92 3.91 3.91 3.90 3.49 3.48 3.47 3.46 3.46 3.45 3.43 3.42 3.23 3.22 3.19 3.18 3.06 3.05 2.88 2.86 2.84 2.83 1.78 1.77 1.75 1.75 1.74 1.66 1.65 1.64 1.64 1.63 1.55 1.54 1.54 1.53 1.51 1.51 1.51 1.50 1.26 1.25 1.23 1.23 1.23 1.22 1.22 1.22 1.21 1.21 1.20 1.20 1.19 1.18 1.02 0.99 0.98 0.96 0.96 0.93 0.91
4m 91:9 d.r
(1H NMR, 400 MHz)
2.00 2.05 0.08 1.85 0.19 1.11 1.09 6.16 1.07 0.09 2.38 1.06 1.39 0.31 0.90 2.92
10.0 9.5 9.0 8.5 8.0 7.5 7.0 6.5 6.0 5.5 5.0 4.5 4.0 3.5 3.0 2.5 2.0 1.5 1.0 0.5 0.0
f1 (ppm)

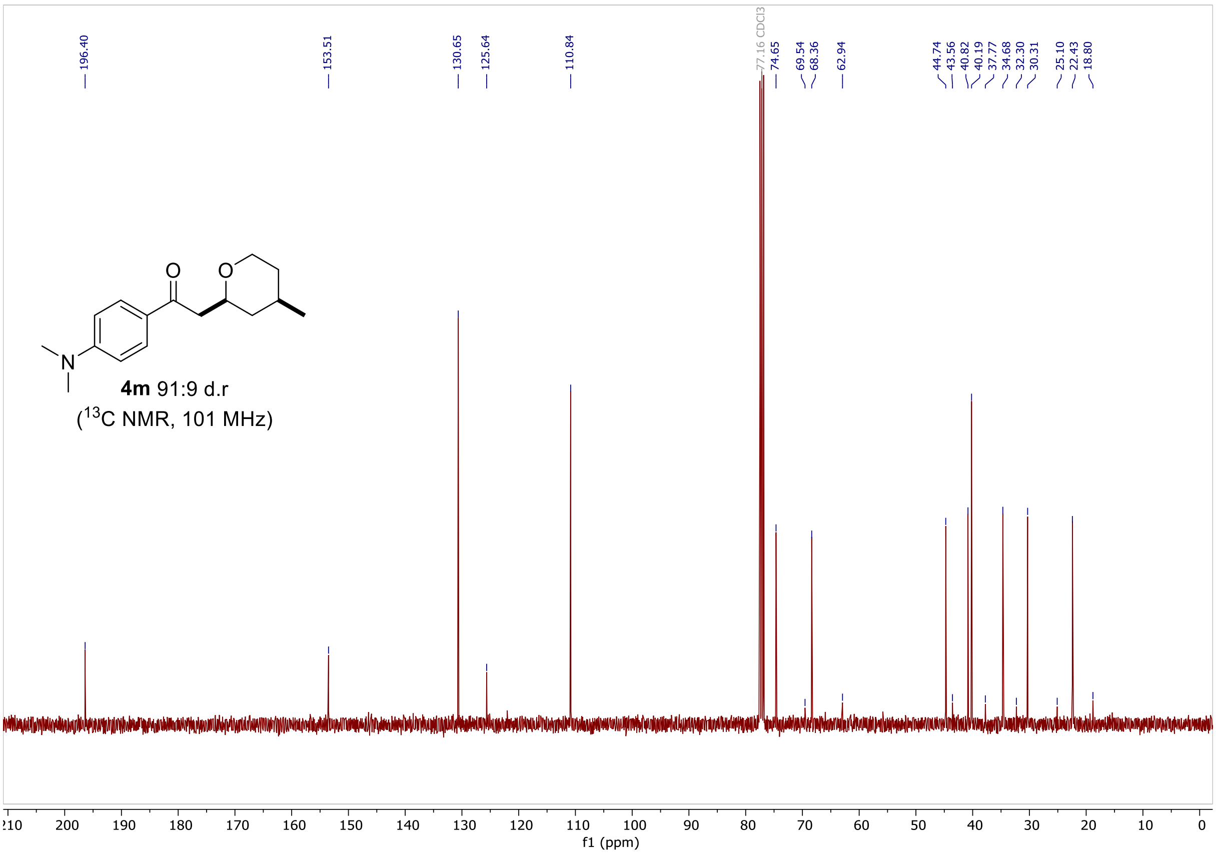
196.40 153.51 130.65 125.64 110.84 77.16 CDCl3 74.65 69.54 68.36 62.94 44.74 43.56 40.82 40.19 37.77 34.68 32.30 30.31 25.10 22.43 18.80
4m 91:9 d.r
(13C NMR, 101 MHz)
210 200 190 180 170 160 150 140 130 120 110 100 90 80 70 60 50 40 30 20 10 0
f1 (ppm)

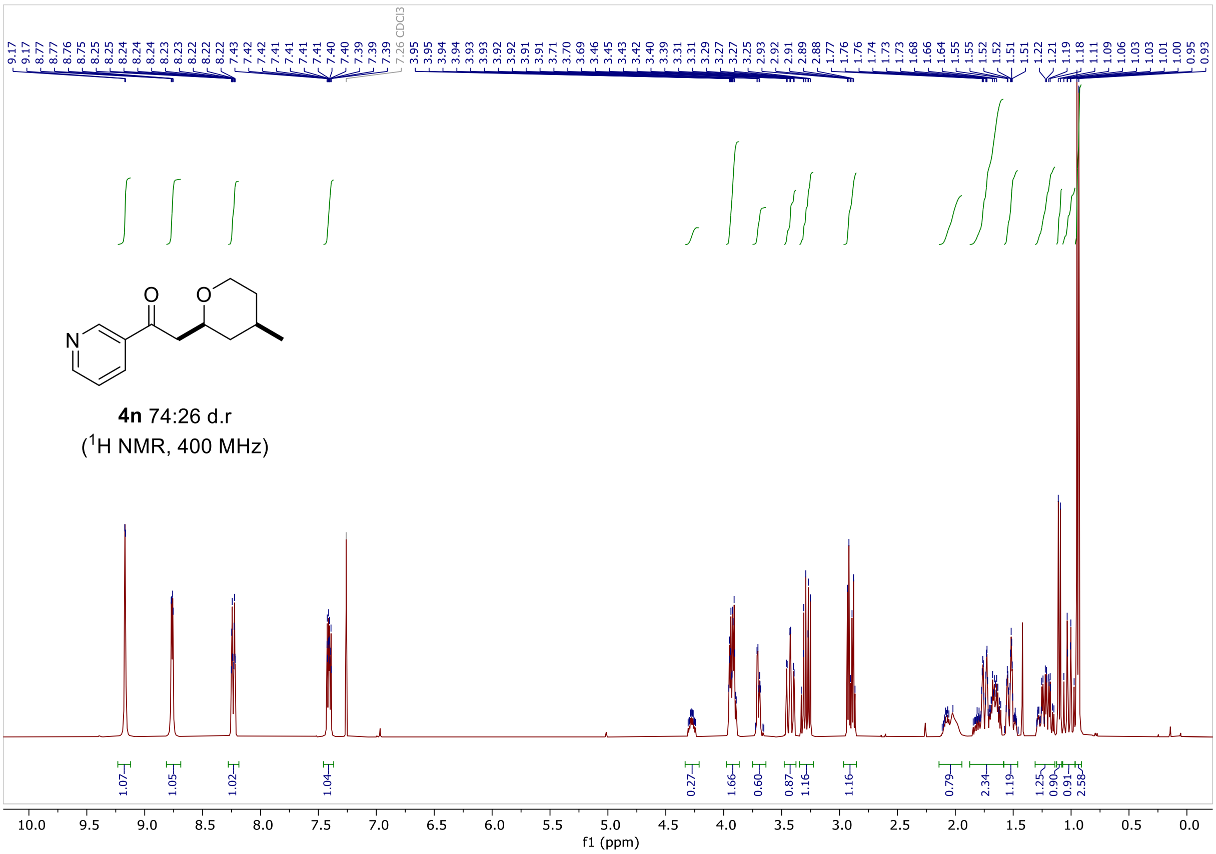

4n 74:26 d.r
(^{1}H NMR, 400 MHz)

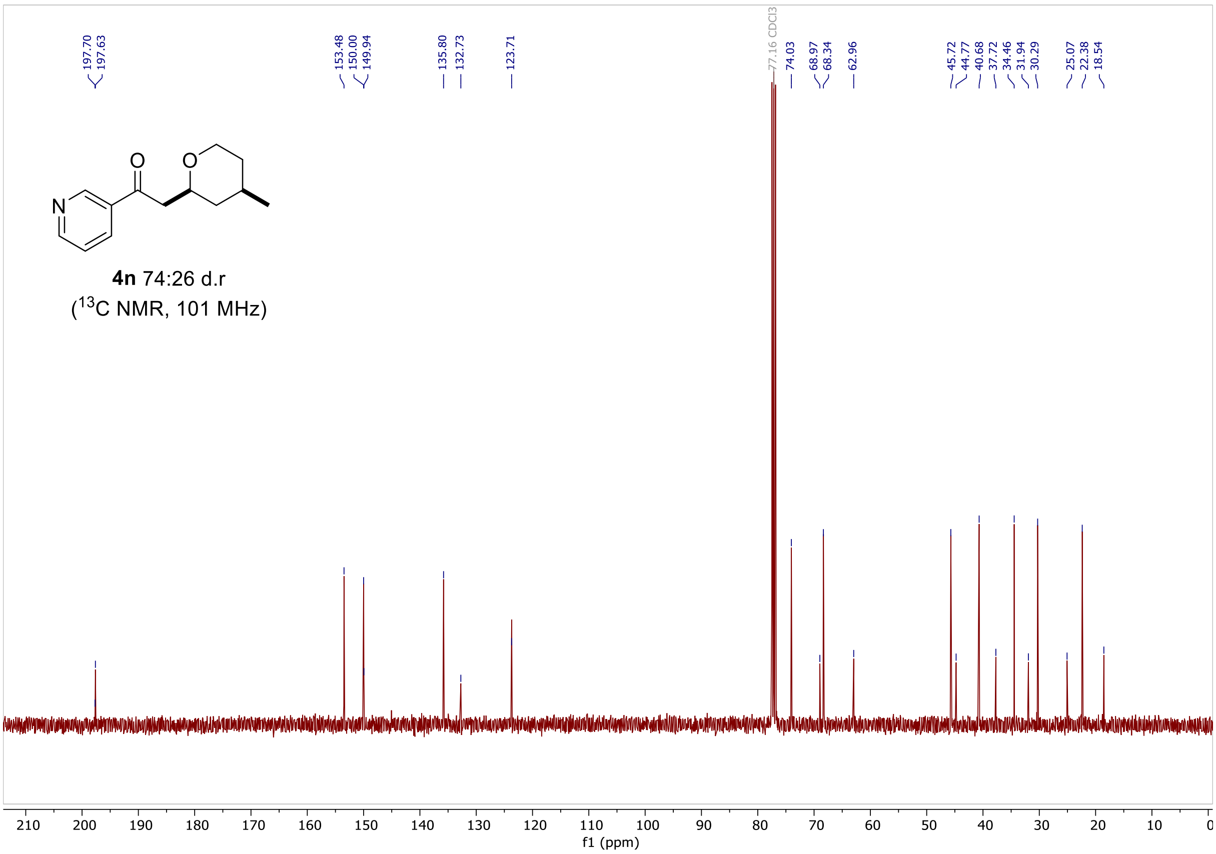

4n 74:26 d.r
(^{13}C NMR, 101 MHz)

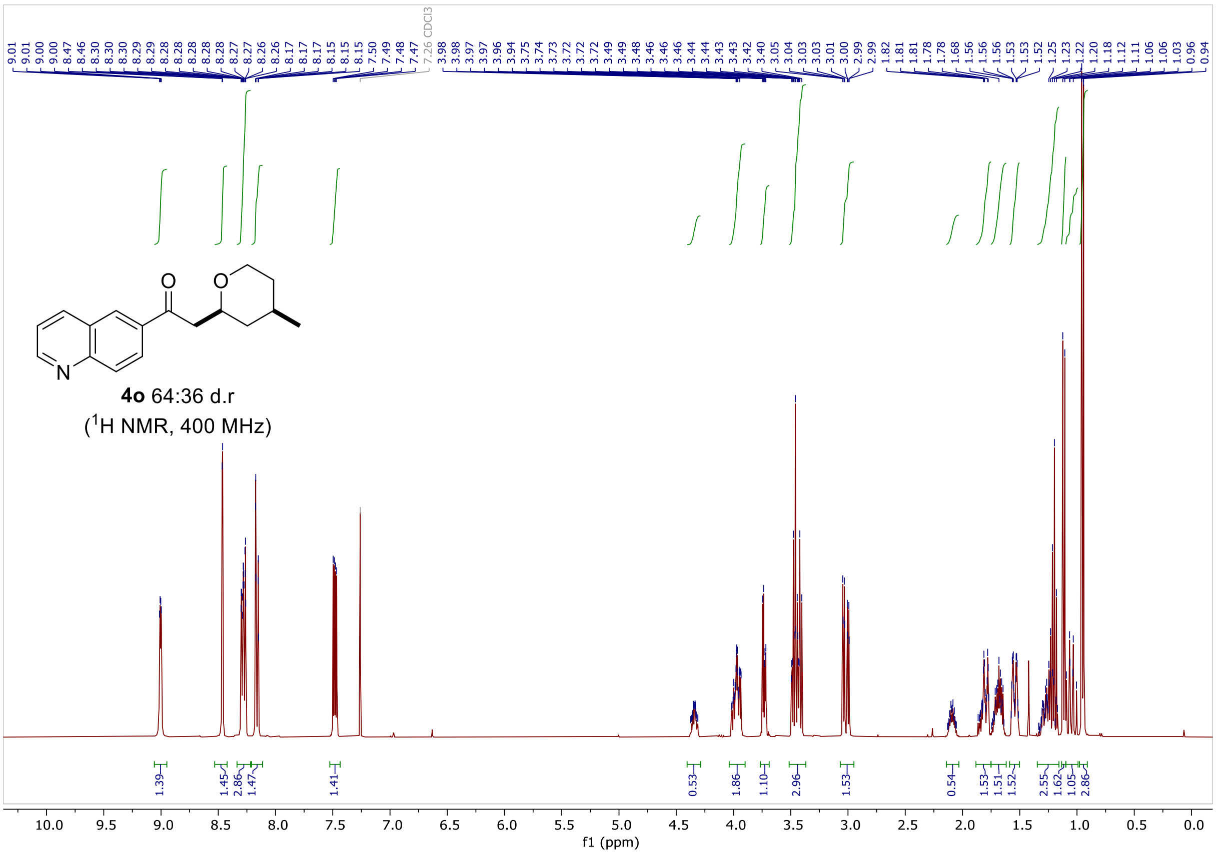
4o 64:36 d.r
(1H NMR, 400 MHz)
f1 (ppm)

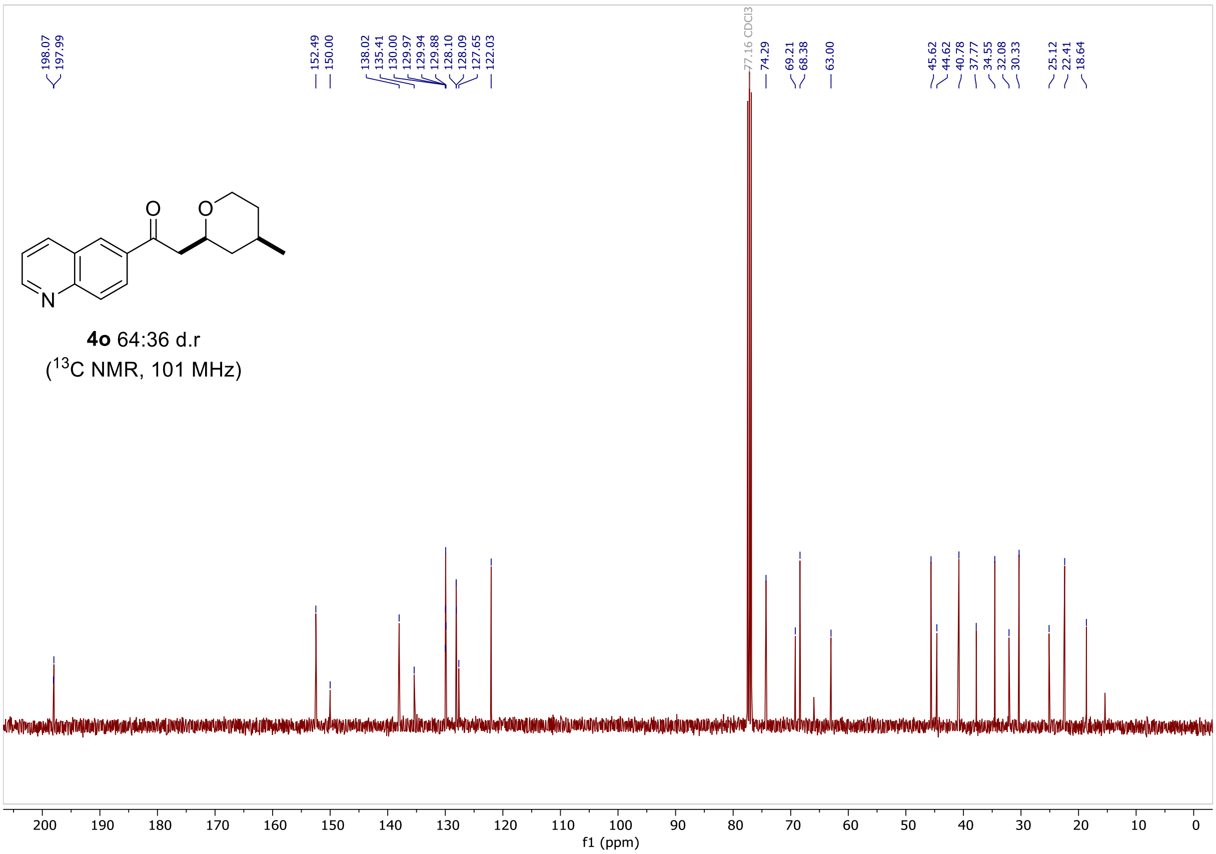
4o 64:36 d.r
(13C NMR, 101 MHz)
f1 (ppm)

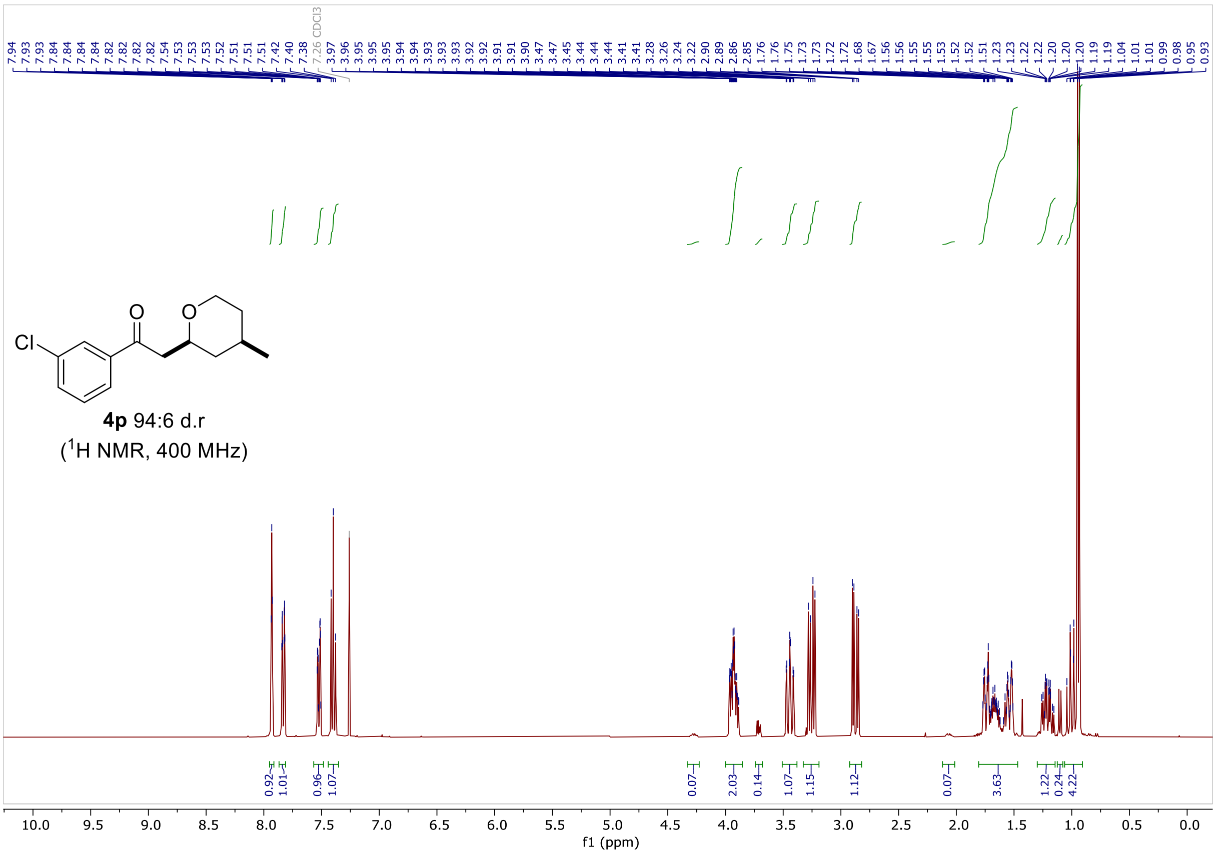
4p 94:6 d.r
(1H NMR, 400 MHz)
f1 (ppm)

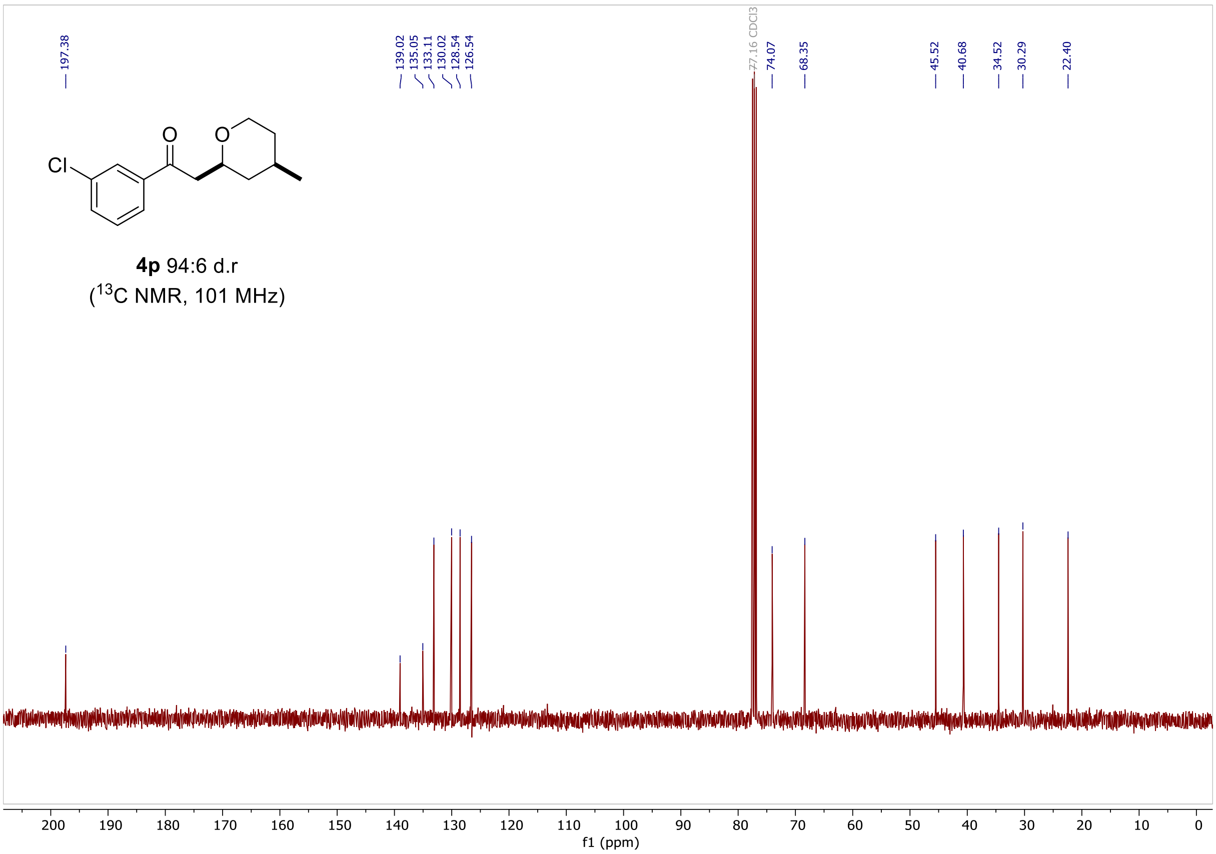
197.38
139.02
135.05
133.11
130.02
128.54
126.54
77.16 CDCl3
74.07
68.35
45.52
40.68
34.52
30.29
22.40
4p 94:6 d.r
(13C NMR, 101 MHz)
f1 (ppm)

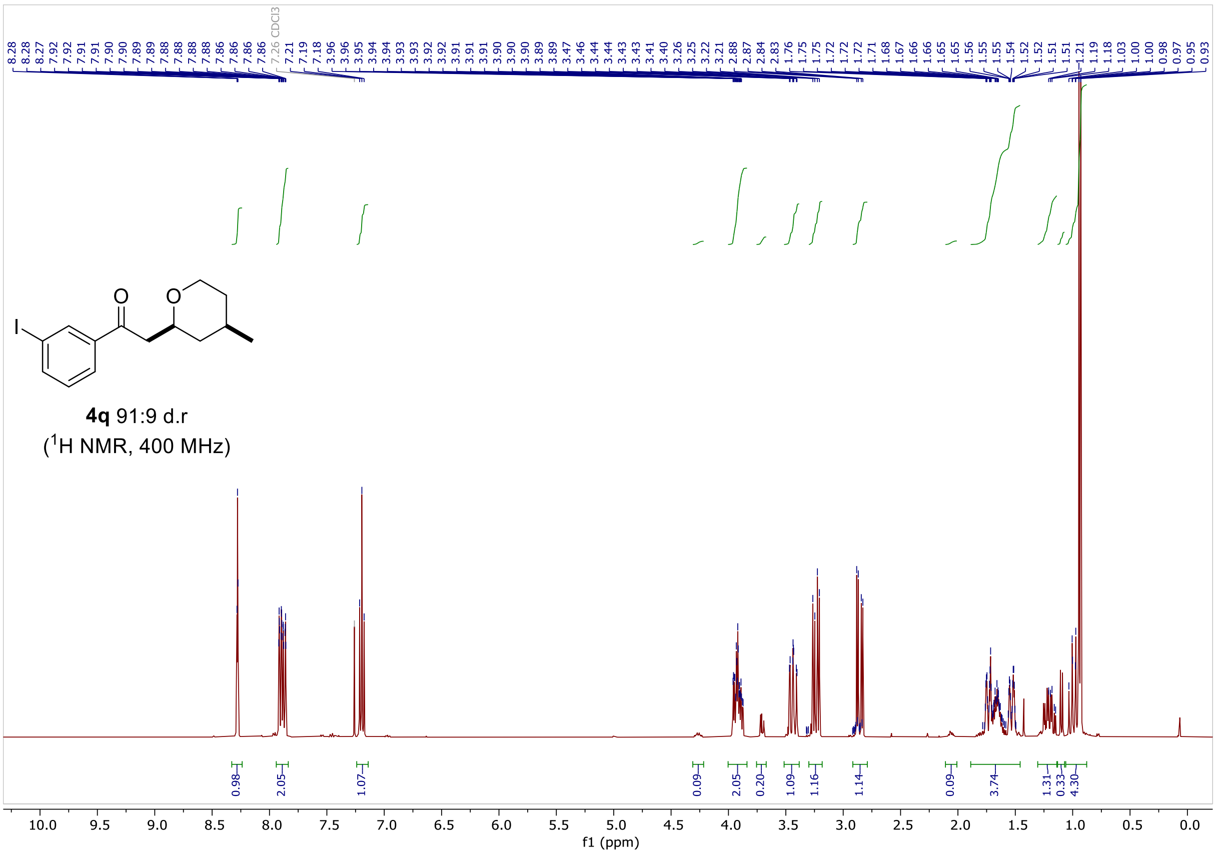
8.28 8.28 8.27 7.92 7.92 7.91 7.91 7.90 7.90 7.89 7.89 7.88 7.88 7.88 7.86 7.86 7.86 7.26 CDCl3 7.21 7.19 7.18 3.96 3.96 3.95 3.95 3.94 3.94 3.93 3.93 3.92 3.92 3.91 3.91 3.91 3.90 3.90 3.90 3.89 3.89 3.47 3.46 3.44 3.44 3.43 3.43 3.41 3.40 3.26 3.25 3.22 3.21 2.88 2.87 2.84 2.83 1.76 1.75 1.75 1.72 1.72 1.72 1.71 1.68 1.67 1.66 1.65 1.65 1.56 1.55 1.55 1.54 1.52 1.52 1.51 1.51 1.21 1.19 1.18 1.03 1.00 1.00 0.98 0.97 0.95 0.93
O
O
I
4q 91:9 d.r
(1H NMR, 400 MHz)
0.98 2.05 1.07 0.09 2.05 0.20 1.09 1.16 1.14 0.09 3.74 1.31 0.33 4.30
f1 (ppm)

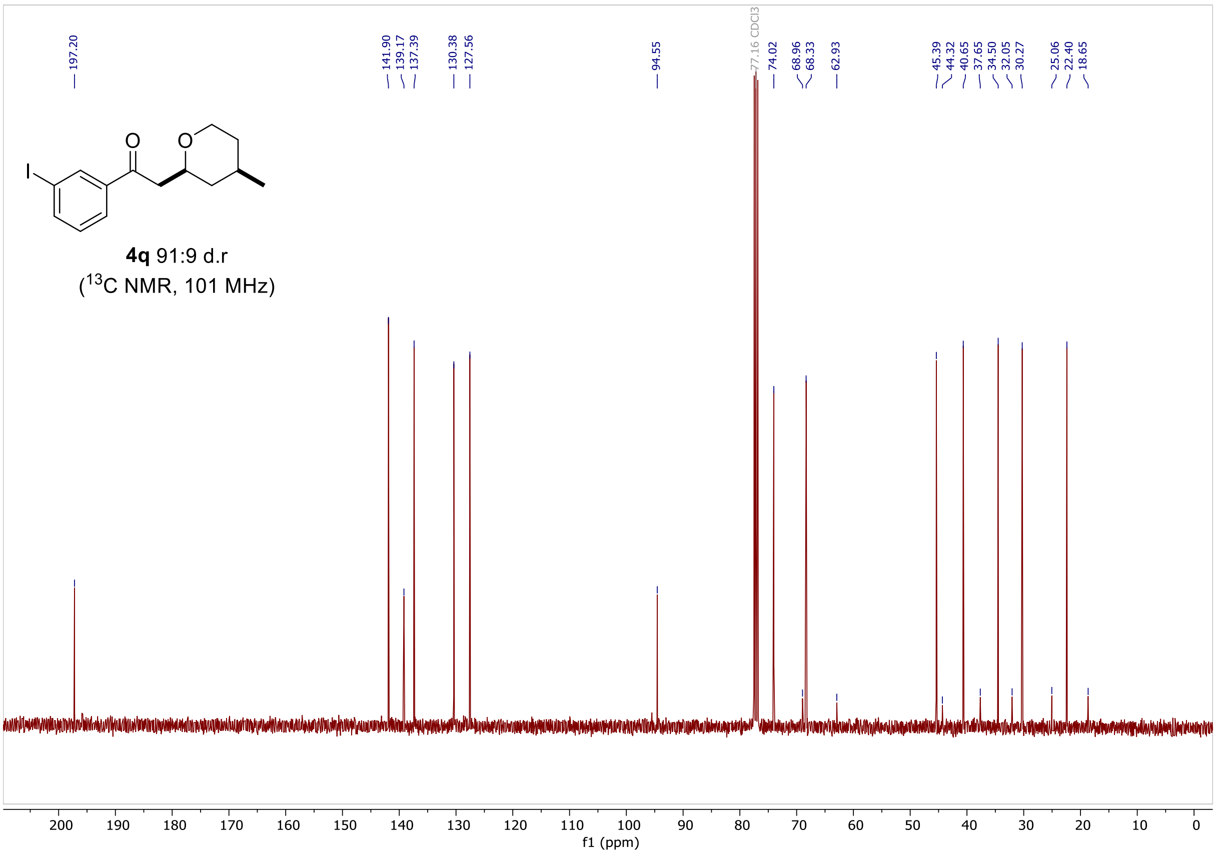
197.20
141.90 139.17 137.39 130.38 127.56
94.55
77.16 CDCl3
74.02 68.96 68.33 62.93
45.39 44.32 40.65 37.65 34.50 32.05 30.27 25.06 22.40 18.65
O
O
I
4q 91:9 d.r
(13C NMR, 101 MHz)
f1 (ppm)

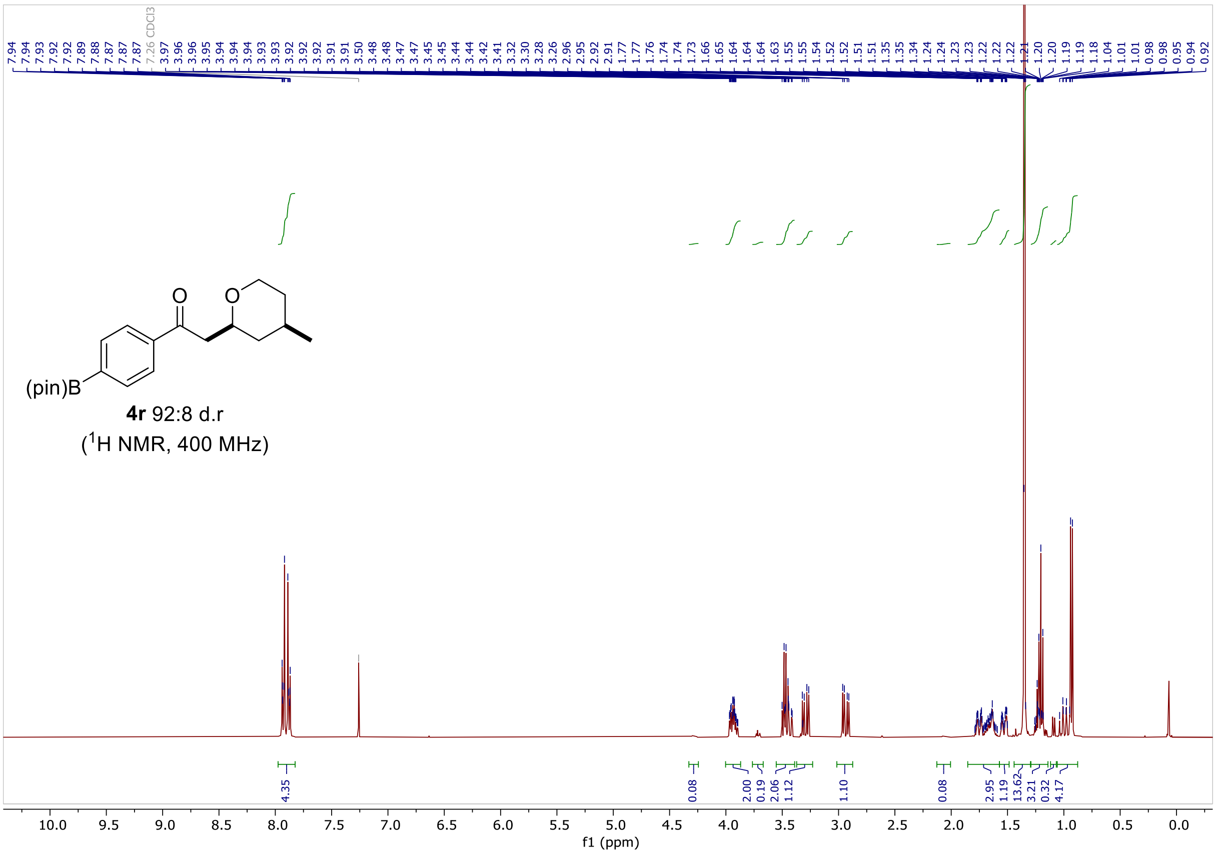

4r 92:8 d.r
(^{1}H NMR, 400 MHz)

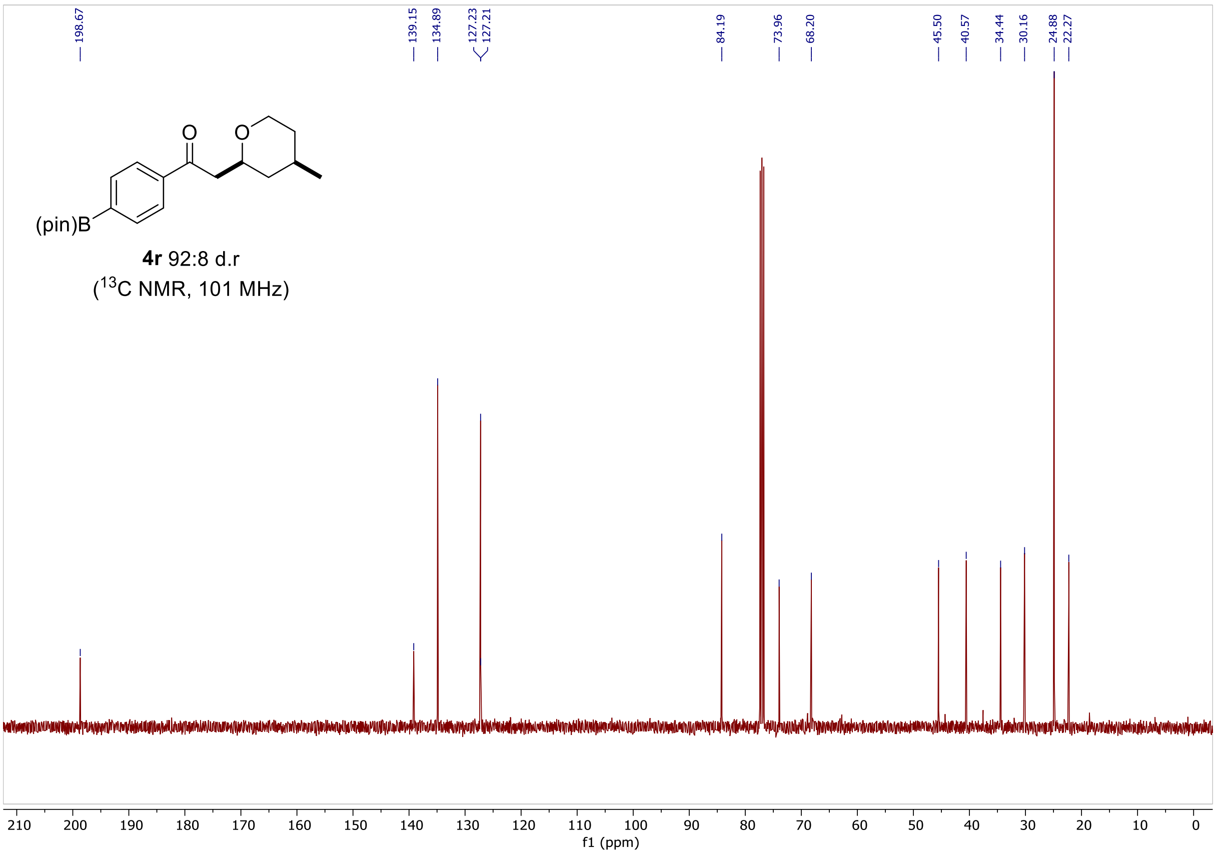

4r 92:8 d.r
(^{13}C NMR, 101 MHz)

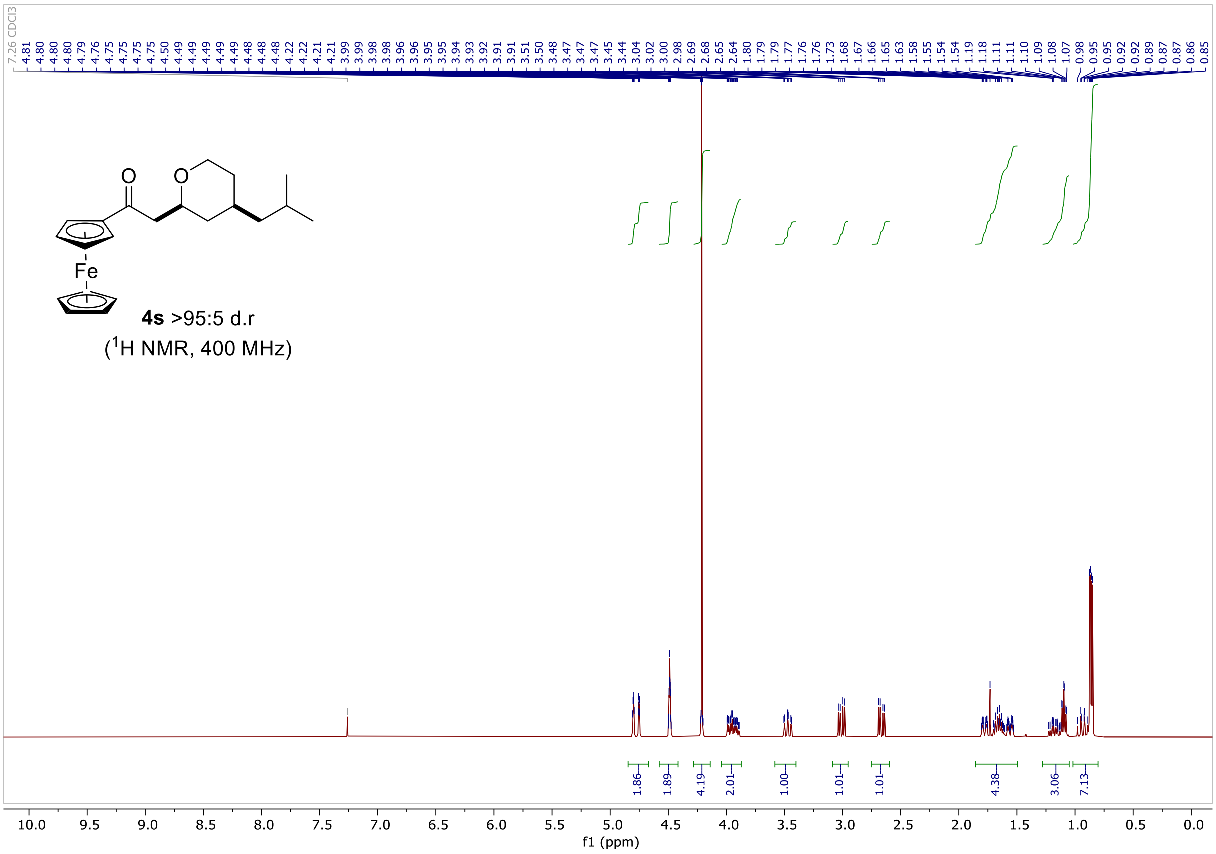
7.26 CDCl3
4.81 4.80 4.80 4.80 4.79 4.76 4.75 4.75 4.75 4.50 4.49 4.49 4.49 4.49 4.48 4.48 4.22 4.22 4.21 4.21 3.99 3.99 3.98 3.98 3.96 3.96 3.95 3.95 3.94 3.93 3.92 3.91 3.91 3.51 3.50 3.48 3.47 3.47 3.45 3.44 3.04 3.02 3.00 2.98 2.69 2.68 2.65 2.64 1.80 1.79 1.79 1.77 1.76 1.76 1.73 1.68 1.67 1.66 1.65 1.63 1.58 1.55 1.54 1.54 1.19 1.18 1.11 1.11 1.10 1.09 1.08 1.07 0.98 0.95 0.95 0.92 0.92 0.89 0.87 0.86 0.85
Fe
4s >95:5 d.r
(^{1}H NMR, 400 MHz)
1.86 1.89 4.19 2.01 1.00 1.01 1.01 4.38 3.06 7.13
f1 (ppm)

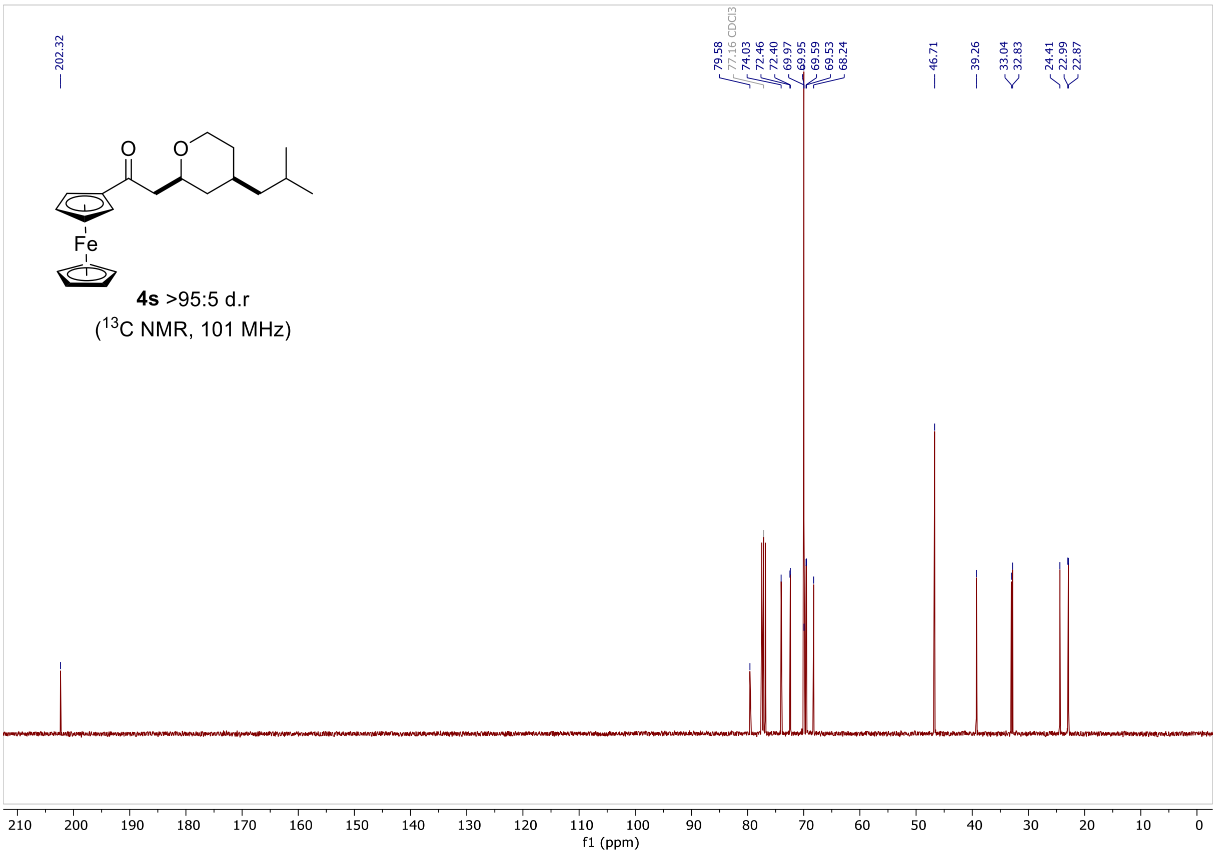
202.32
79.58 77.16 CDCl3 74.03 72.46 72.40 69.97 69.95 69.59 69.53 68.24
46.71 39.26 33.04 32.83 24.41 22.99 22.87
Fe
4s >95:5 d.r
(^{13}C NMR, 101 MHz)
f1 (ppm)

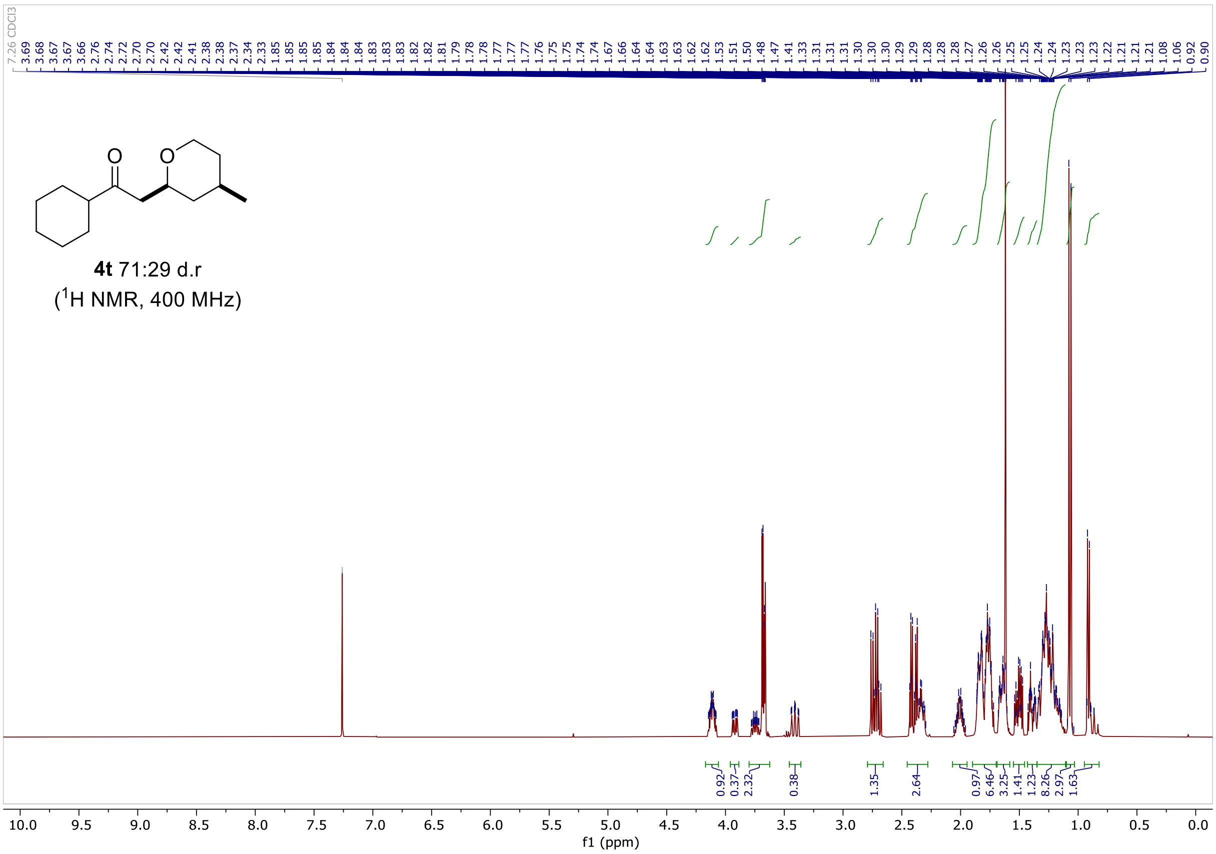

4t 71:29 d.r
(^{1}H NMR, 400 MHz)

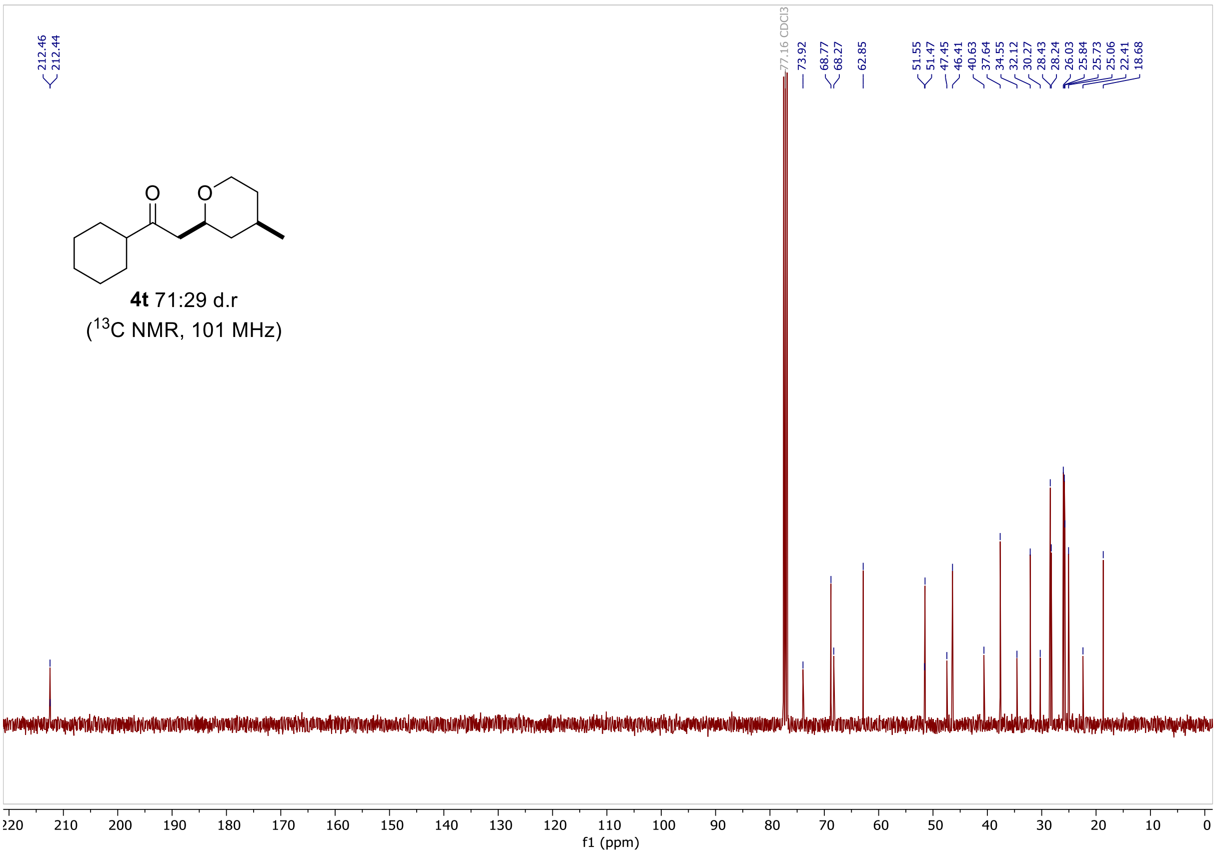

4t 71:29 d.r
(^{13}C NMR, 101 MHz)

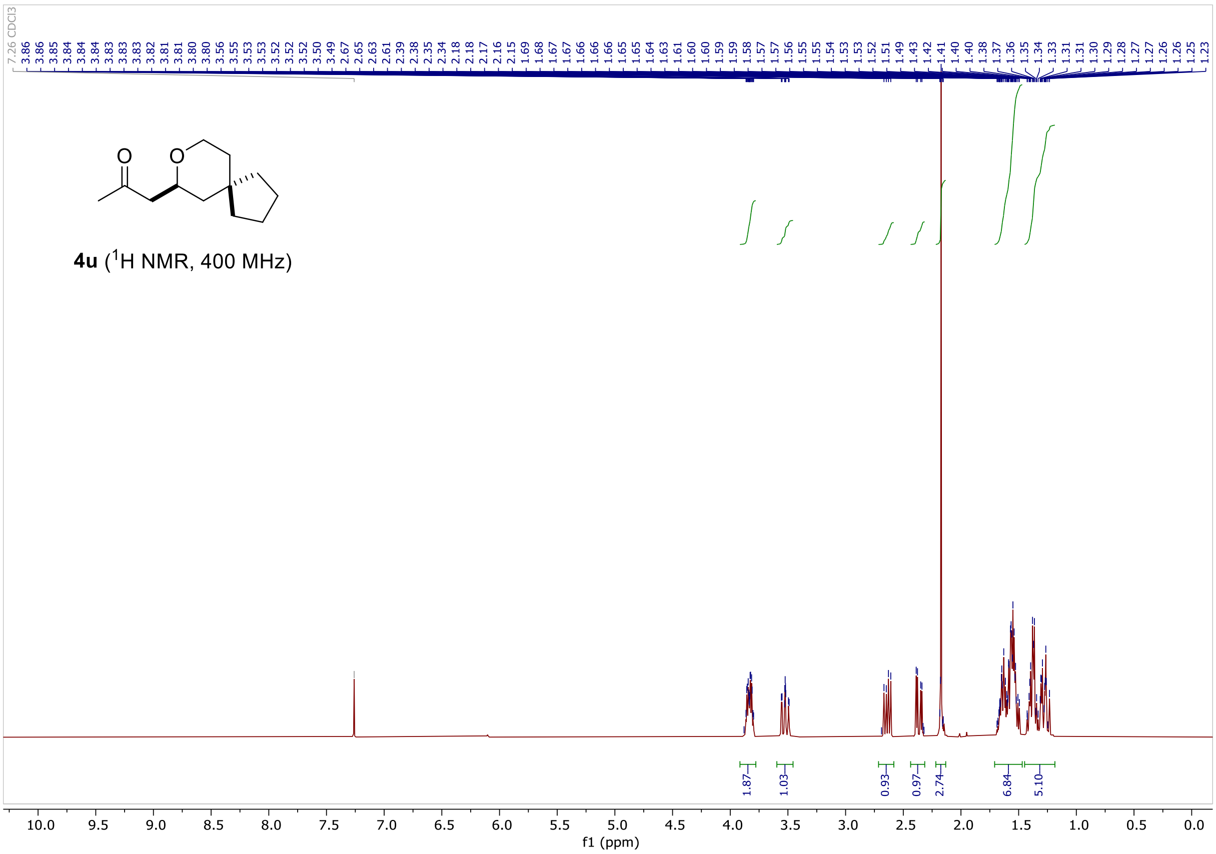

4u (1H NMR, 400 MHz)
7.26 CDCl3
f1 (ppm)
1.87
1.03
0.93
0.97
2.74
6.84
5.10

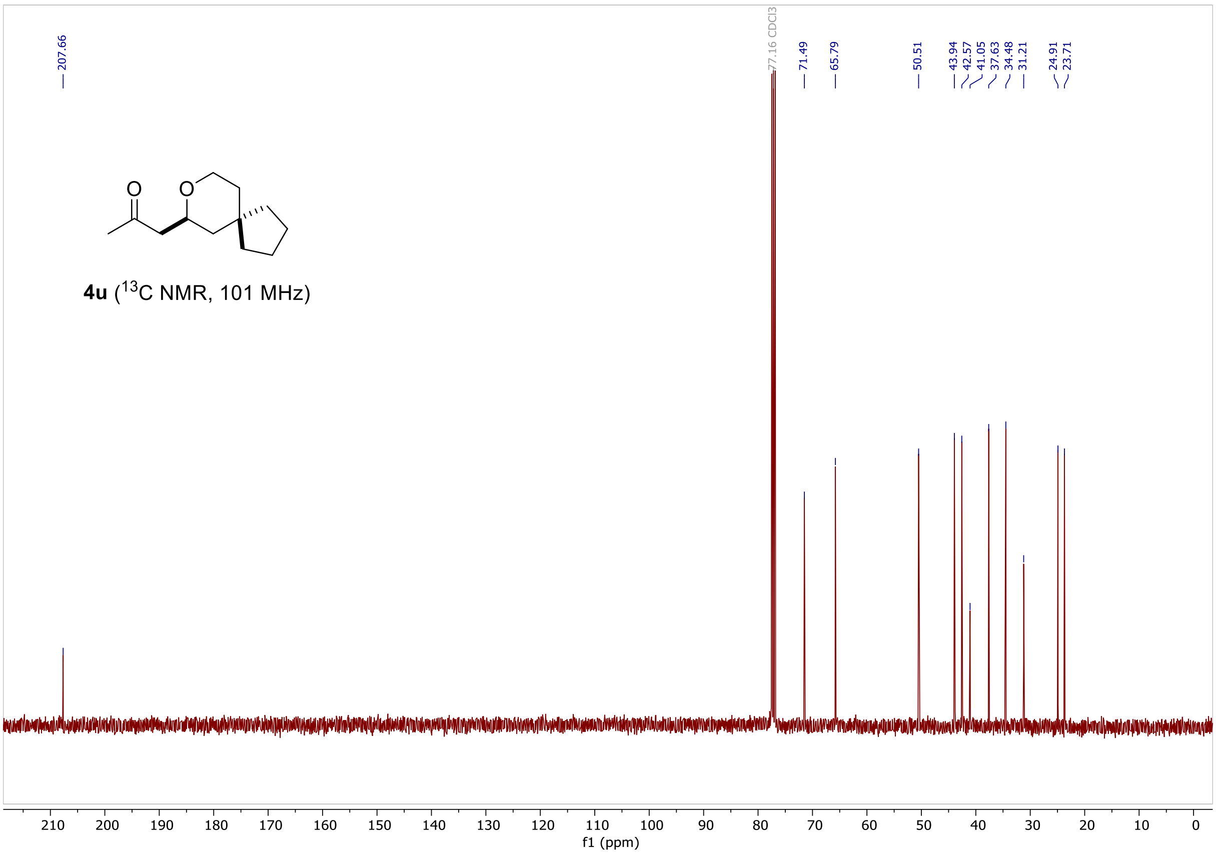

4u (13C NMR, 101 MHz)
207.66
77.16 CDCl3
71.49
65.79
50.51
43.94
42.57
41.05
37.63
34.48
31.21
24.91
23.71
f1 (ppm)

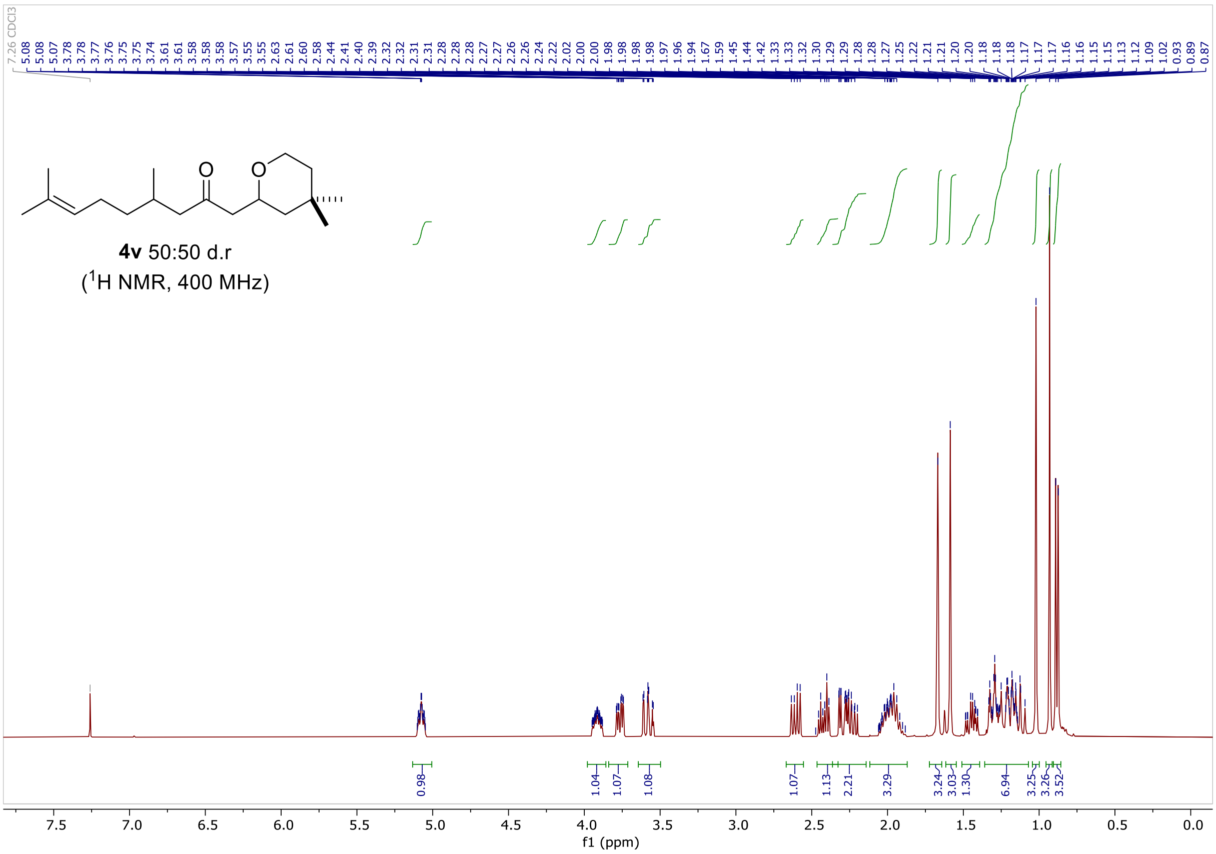

7.26 CDCl3
5.08
5.08
5.07
3.78
3.78
3.77
3.76
3.75
3.75
3.74
3.61
3.61
3.58
3.58
3.58
3.57
3.55
3.55
2.63
2.61
2.60
2.58
2.44
2.41
2.40
2.39
2.32
2.32
2.31
2.31
2.28
2.28
2.28
2.27
2.27
2.26
2.26
2.24
2.22
2.02
2.00
2.00
1.98
1.98
1.98
1.98
1.97
1.96
1.94
1.67
1.59
1.45
1.44
1.42
1.33
1.33
1.32
1.30
1.29
1.29
1.28
1.28
1.27
1.25
1.22
1.21
1.21
1.20
1.20
1.18
1.18
1.18
1.17
1.17
1.17
1.17
1.16
1.16
1.15
1.15
1.13
1.12
1.09
1.02
0.93
0.89
0.87
O
O
4v 50:50 d.r
(1H NMR, 400 MHz)
0.98
1.04
1.07
1.08
1.07
1.13
2.21
3.29
3.24
3.03
1.30
6.94
3.25
3.26
3.52
7.5 7.0 6.5 6.0 5.5 5.0 4.5 4.0 3.5 3.0 2.5 2.0 1.5 1.0 0.5 0.0
f1 (ppm)

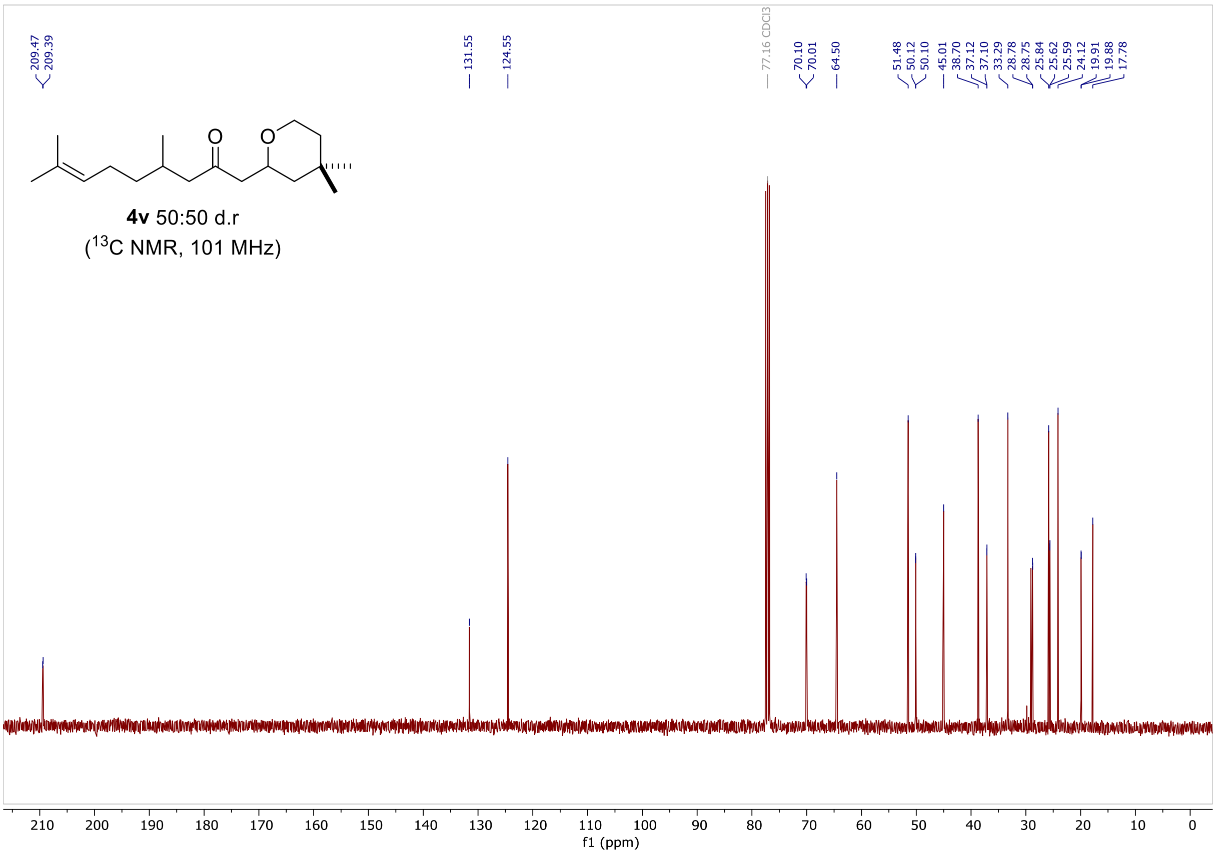

209.47
209.39
131.55
124.55
77.16 CDCl3
70.10
70.01
64.50
51.48
50.12
50.10
45.01
38.70
37.12
37.10
33.29
28.78
28.75
25.84
25.62
25.59
24.12
19.91
19.88
17.78
O
O
4v 50:50 d.r
(13C NMR, 101 MHz)
210 200 190 180 170 160 150 140 130 120 110 100 90 80 70 60 50 40 30 20 10 0
f1 (ppm)

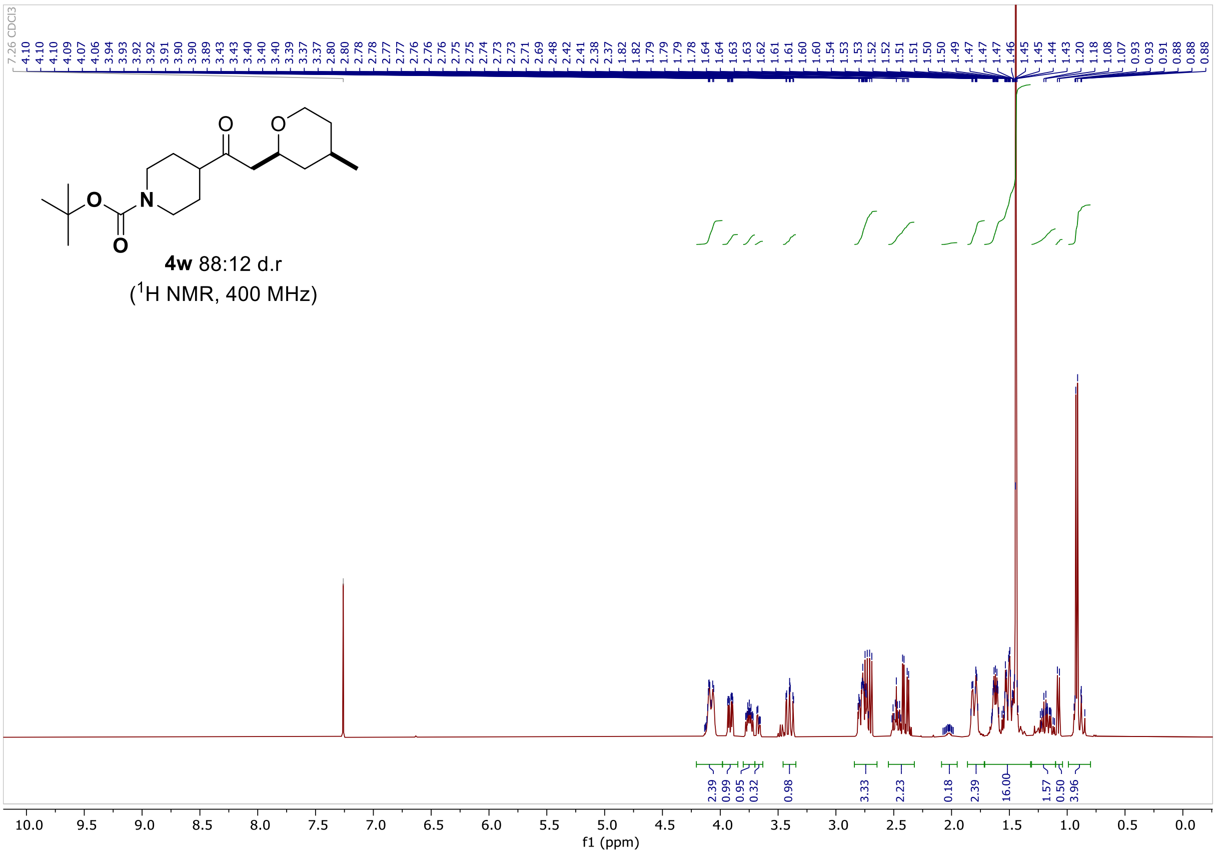
4w 88:12 d.r
(1H NMR, 400 MHz)
7.26 CDCl3
4.10
4.10
4.10
4.09
4.07
4.06
3.94
3.93
3.92
3.92
3.91
3.90
3.90
3.89
3.43
3.43
3.40
3.40
3.39
3.37
3.37
2.80
2.80
2.78
2.78
2.77
2.77
2.76
2.76
2.76
2.75
2.75
2.74
2.73
2.73
2.71
2.69
2.48
2.42
2.41
2.38
2.37
1.82
1.82
1.82
1.79
1.79
1.79
1.78
1.64
1.64
1.63
1.63
1.62
1.61
1.61
1.60
1.60
1.54
1.53
1.53
1.52
1.52
1.51
1.51
1.50
1.50
1.49
1.47
1.47
1.47
1.46
1.45
1.45
1.44
1.43
1.20
1.18
1.08
1.07
0.93
0.93
0.91
0.88
0.88
2.39
0.99
0.95
0.32
0.98
3.33
2.23
0.18
2.39
16.00
1.57
0.50
3.96
10.0
9.5
9.0
8.5
8.0
7.5
7.0
6.5
6.0
5.5
5.0
4.5
4.0
3.5
3.0
2.5
2.0
1.5
1.0
0.5
0.0
f1 (ppm)

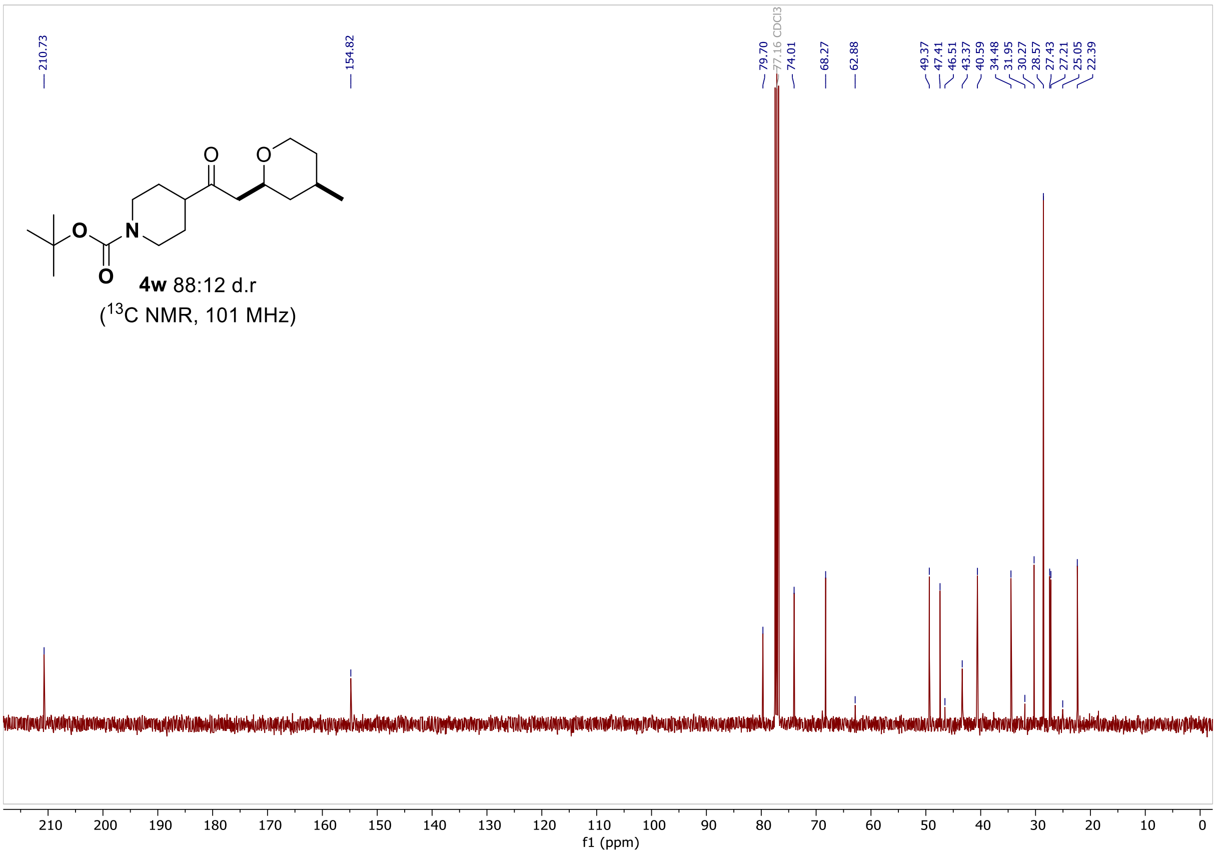
4w 88:12 d.r
(13C NMR, 101 MHz)
210.73
154.82
79.70
77.16 CDCl3
74.01
68.27
62.88
49.37
47.41
46.51
43.37
40.59
34.48
31.95
30.27
28.57
27.43
27.21
25.05
22.39
210
200
190
180
170
160
150
140
130
120
110
100
90
80
70
60
50
40
30
20
10
0
f1 (ppm)

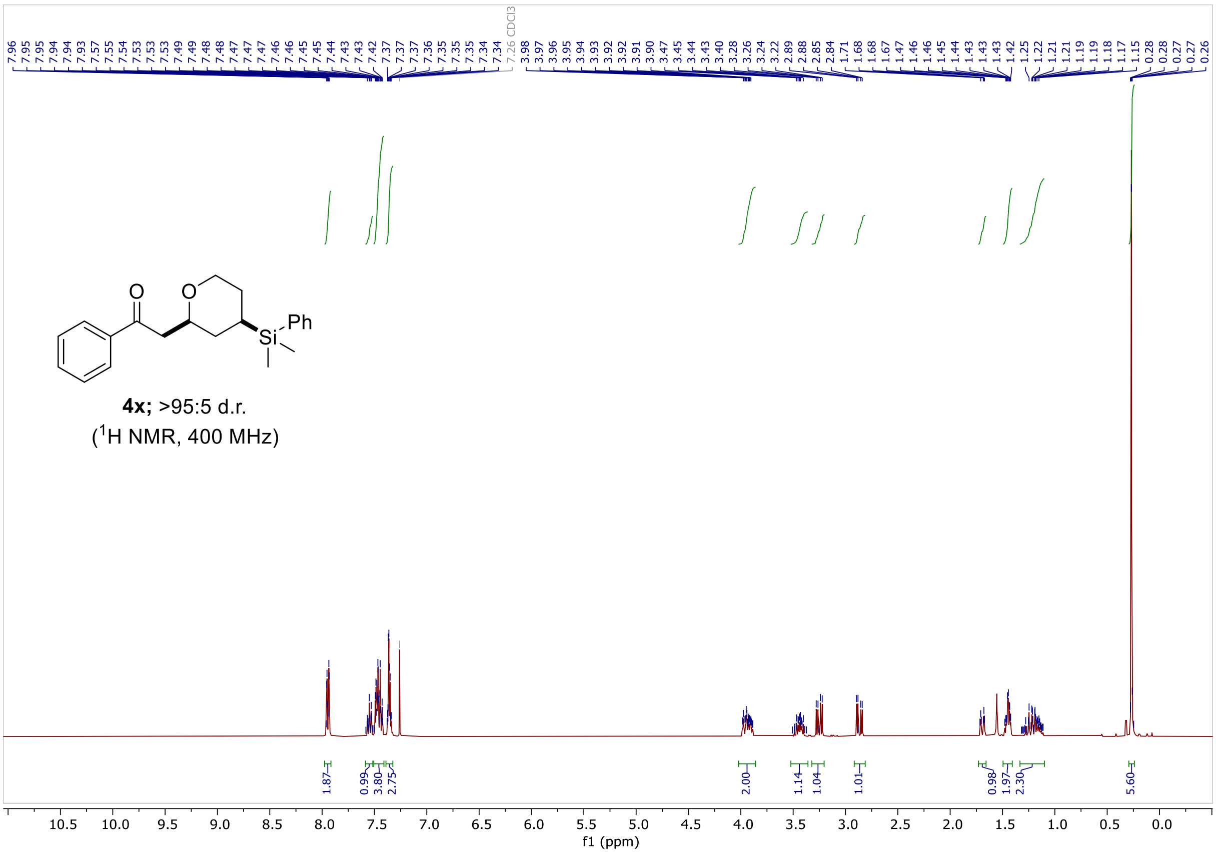
4x; >95:5 d.r.
(1H NMR, 400 MHz)
f1 (ppm)

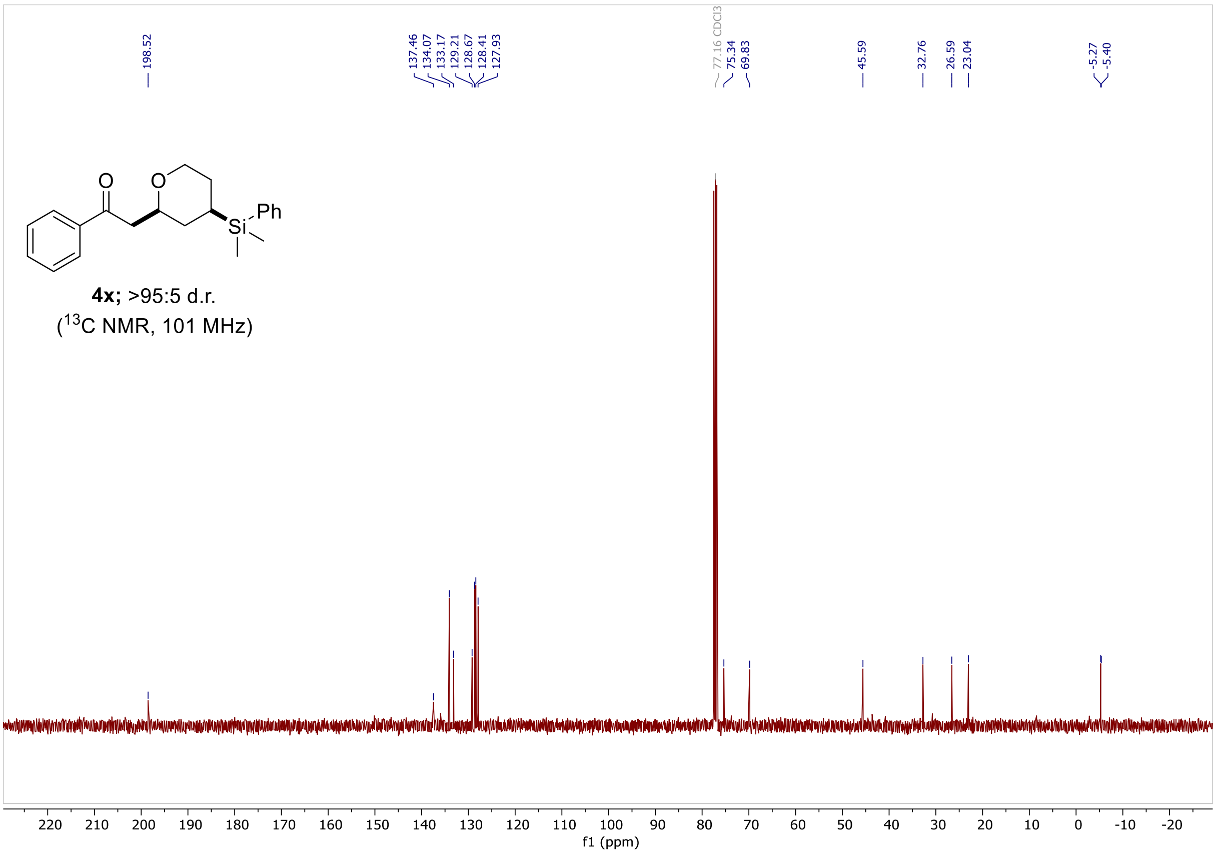
4x; >95:5 d.r.
(13C NMR, 101 MHz)
f1 (ppm)

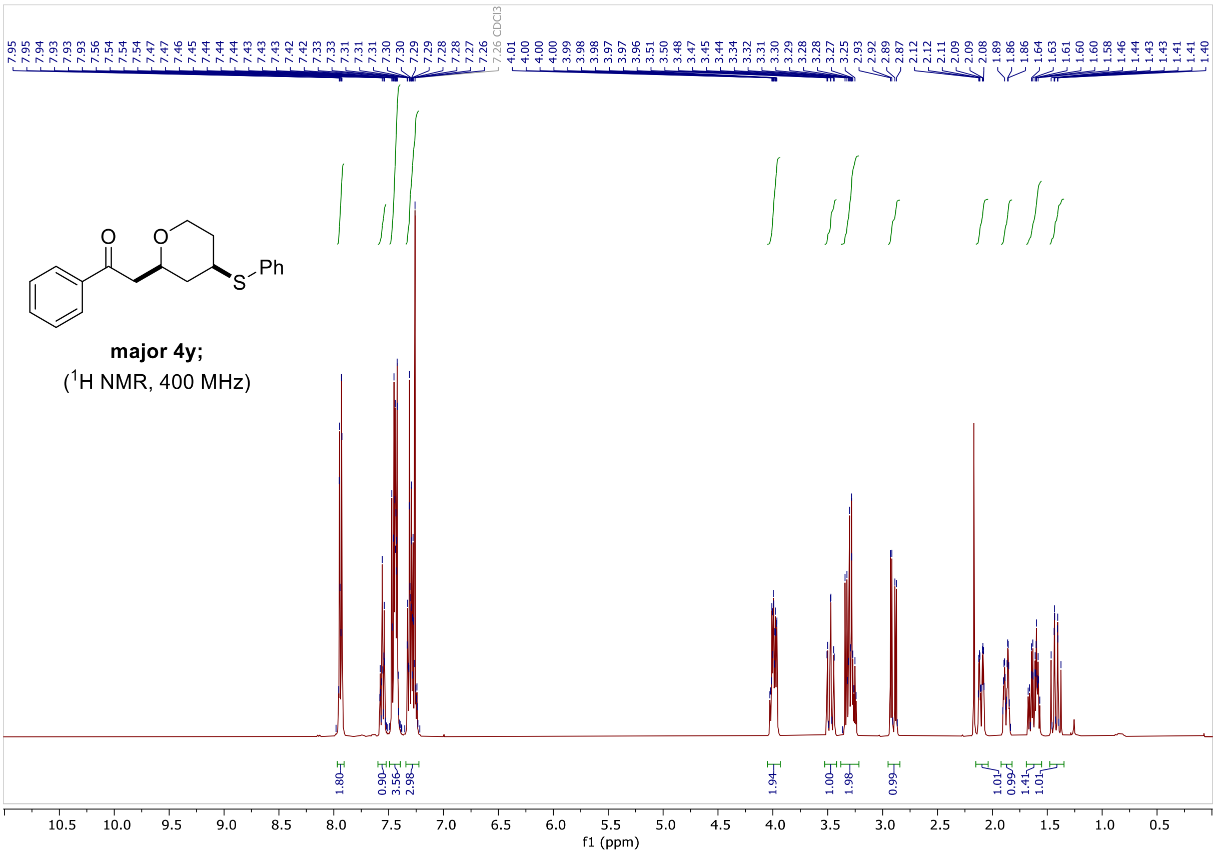

O
O
S
Ph
major 4y;
(1H NMR, 400 MHz)
f1 (ppm)

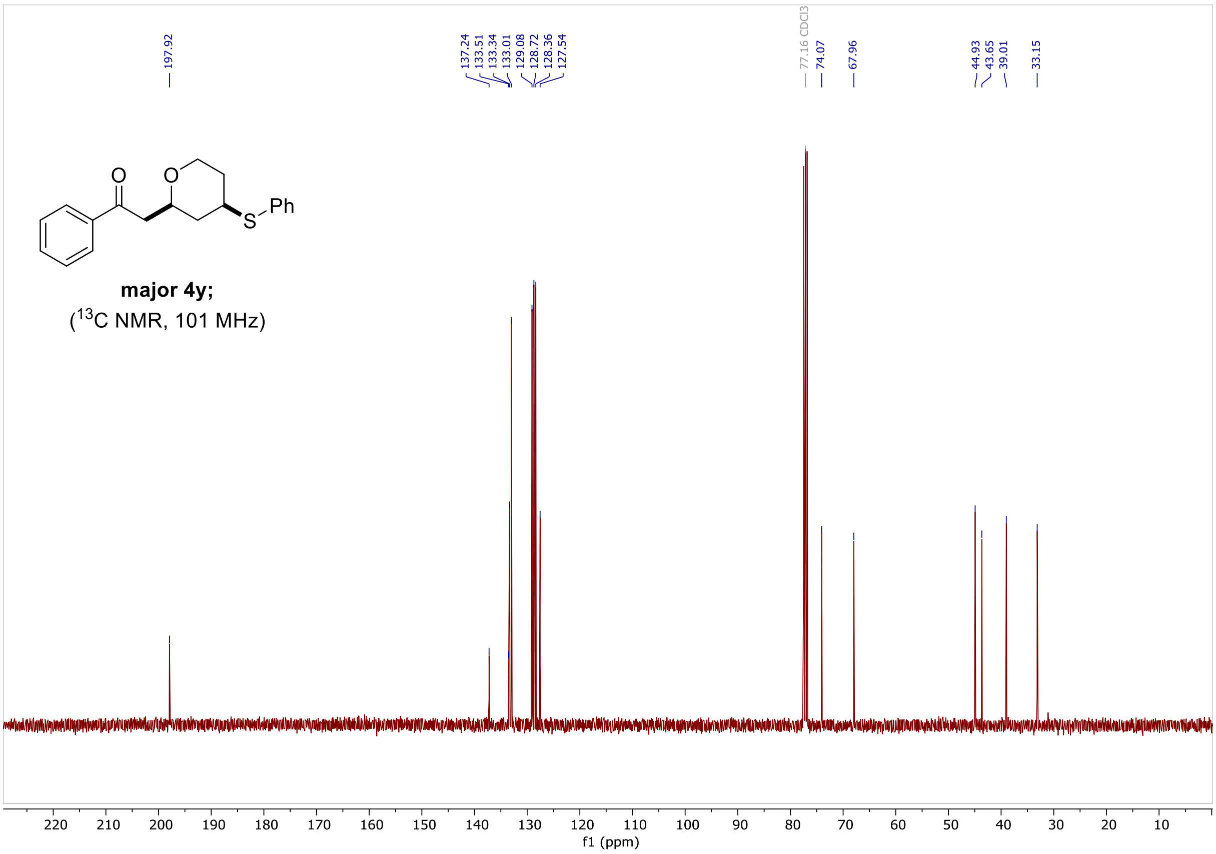

197.92
137.24
133.51
133.34
133.01
129.08
128.72
128.36
127.54
77.16 CDCl3
74.07
67.96
44.93
43.65
39.01
33.15
O
O
S
Ph
major 4y;
(13C NMR, 101 MHz)
f1 (ppm)

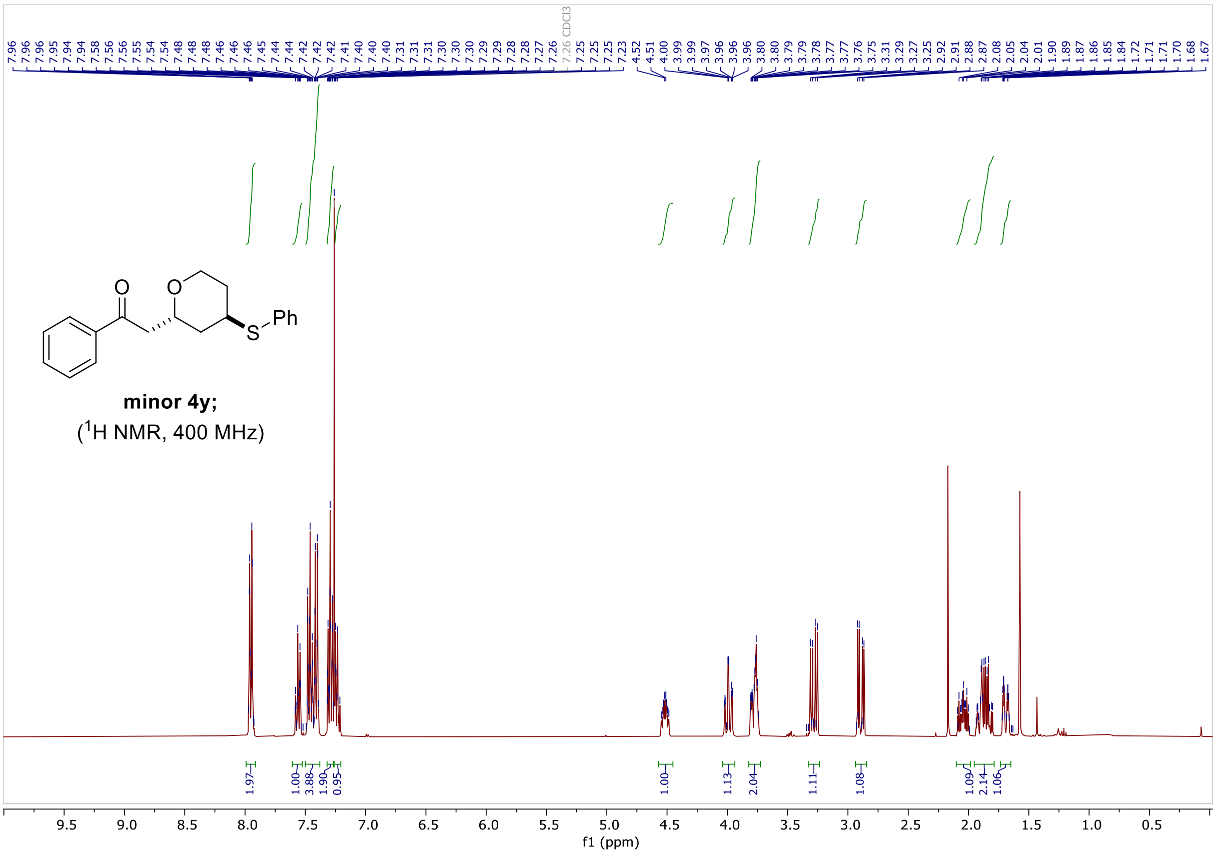
minor 4y;
(1H NMR, 400 MHz)
f1 (ppm)

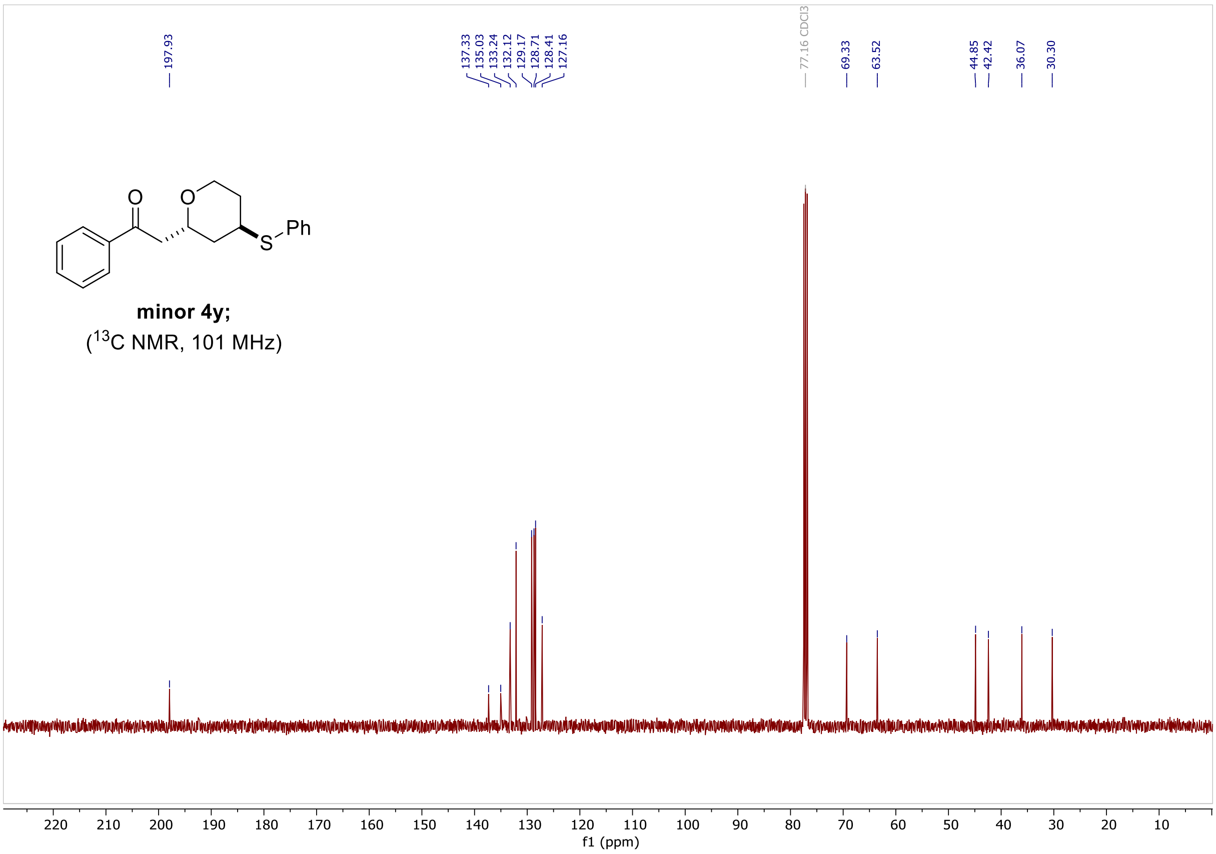
minor 4y;
(13C NMR, 101 MHz)
197.93
137.33
135.03
133.24
132.12
129.17
128.71
128.41
127.16
77.16 CDCl3
69.33
63.52
44.85
42.42
36.07
30.30
f1 (ppm)

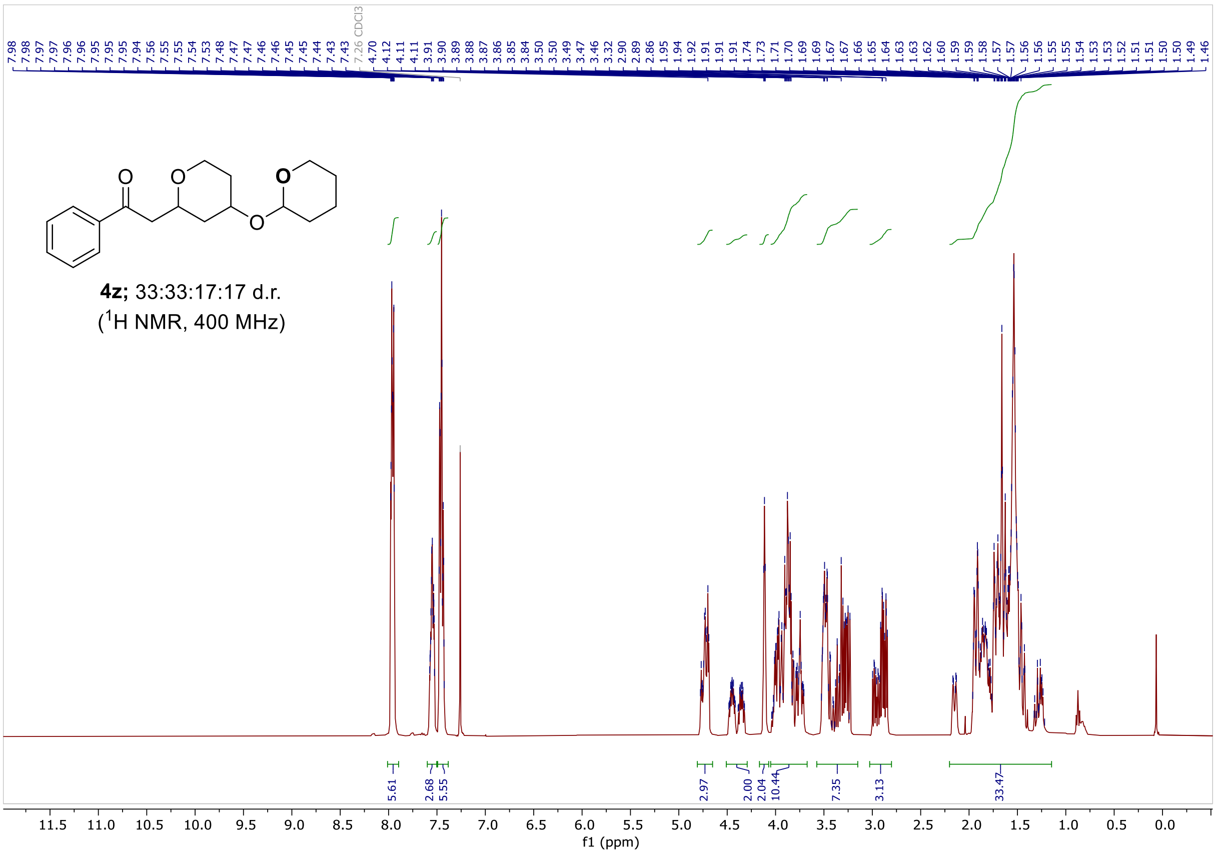
4z; 33:33:17:17 d.r.
(1H NMR, 400 MHz)
f1 (ppm)

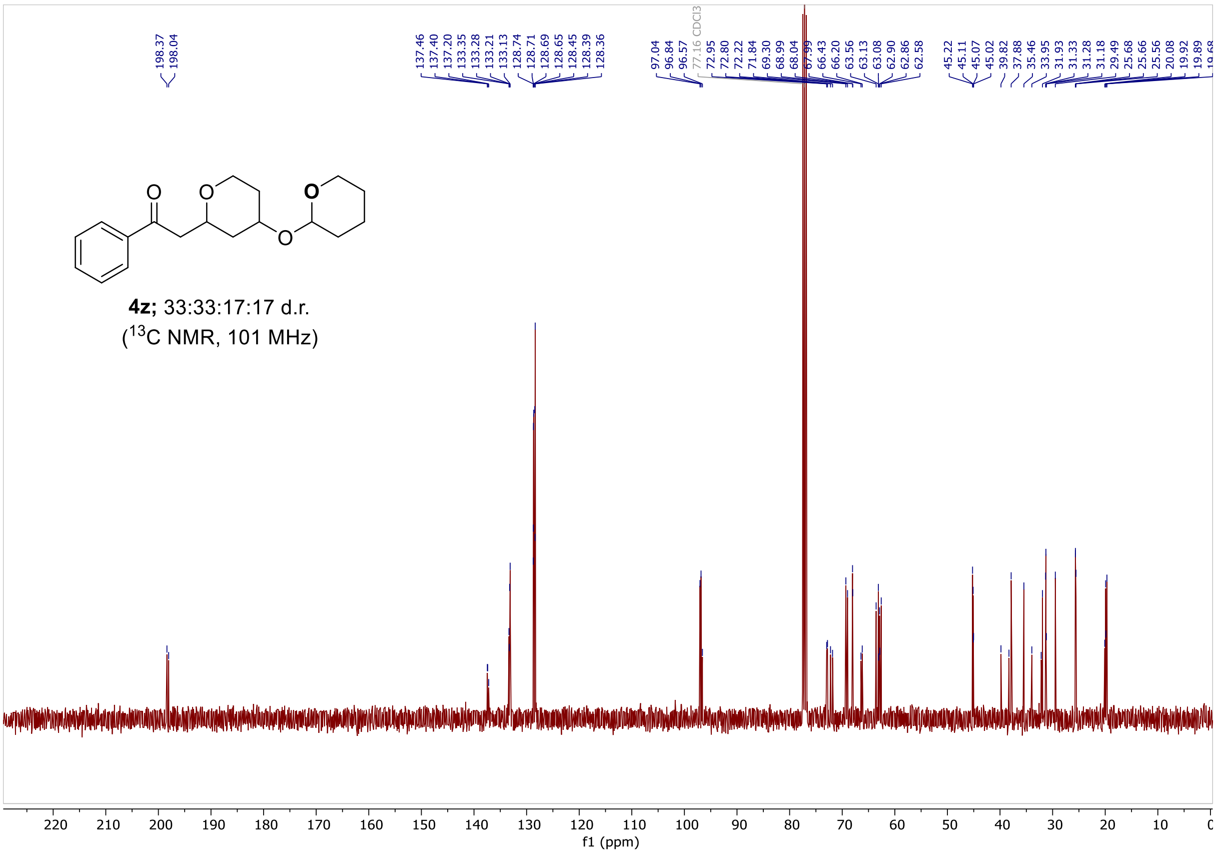
4z; 33:33:17:17 d.r.
(13C NMR, 101 MHz)
f1 (ppm)

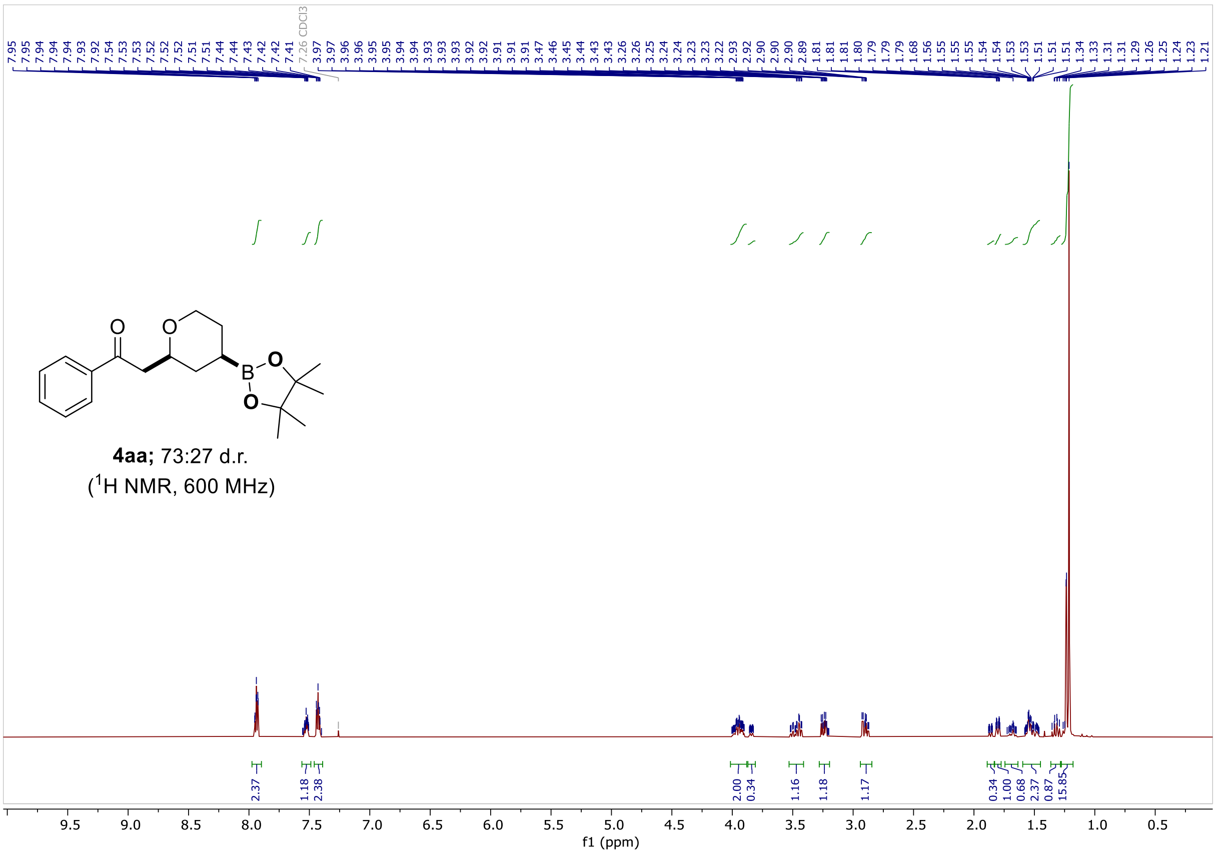

4aa; 73:27 d.r.
(1H NMR, 600 MHz)
f1 (ppm)

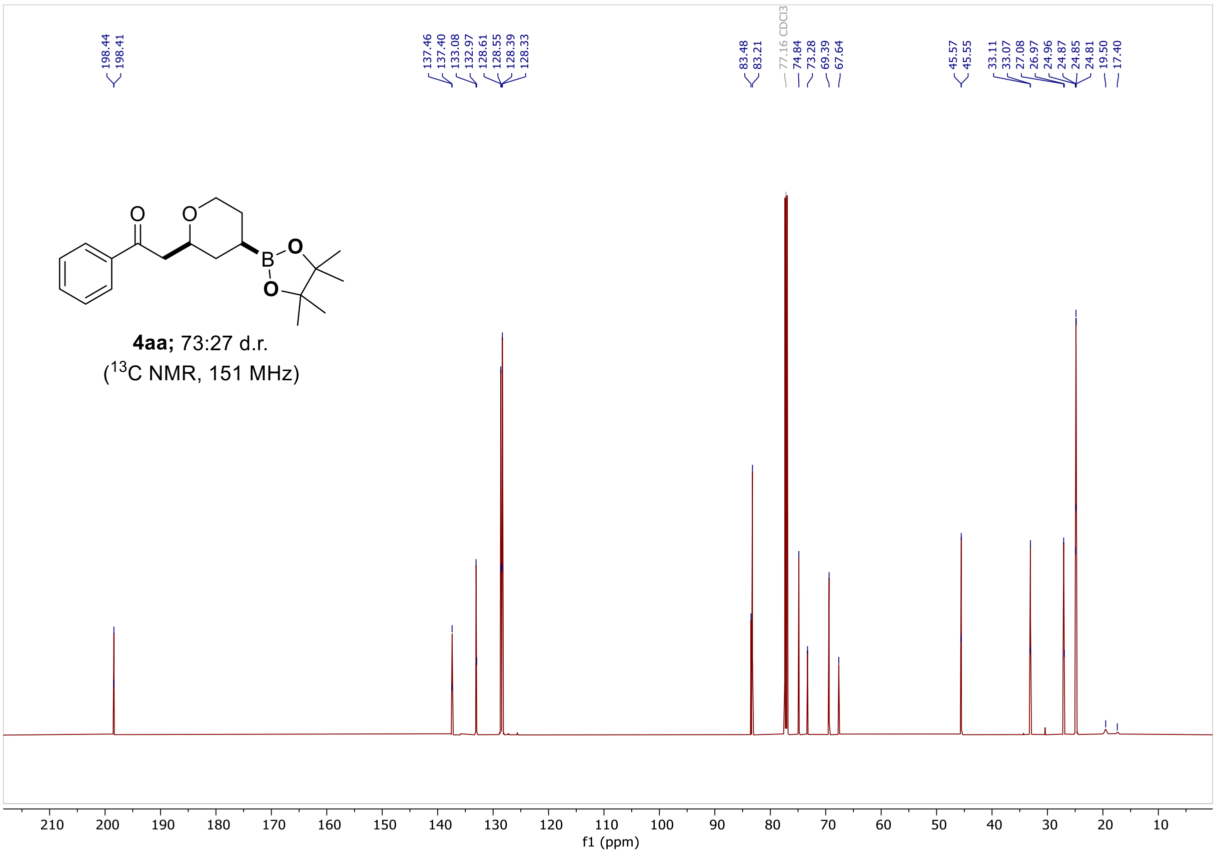

4aa; 73:27 d.r.
(13C NMR, 151 MHz)
f1 (ppm)

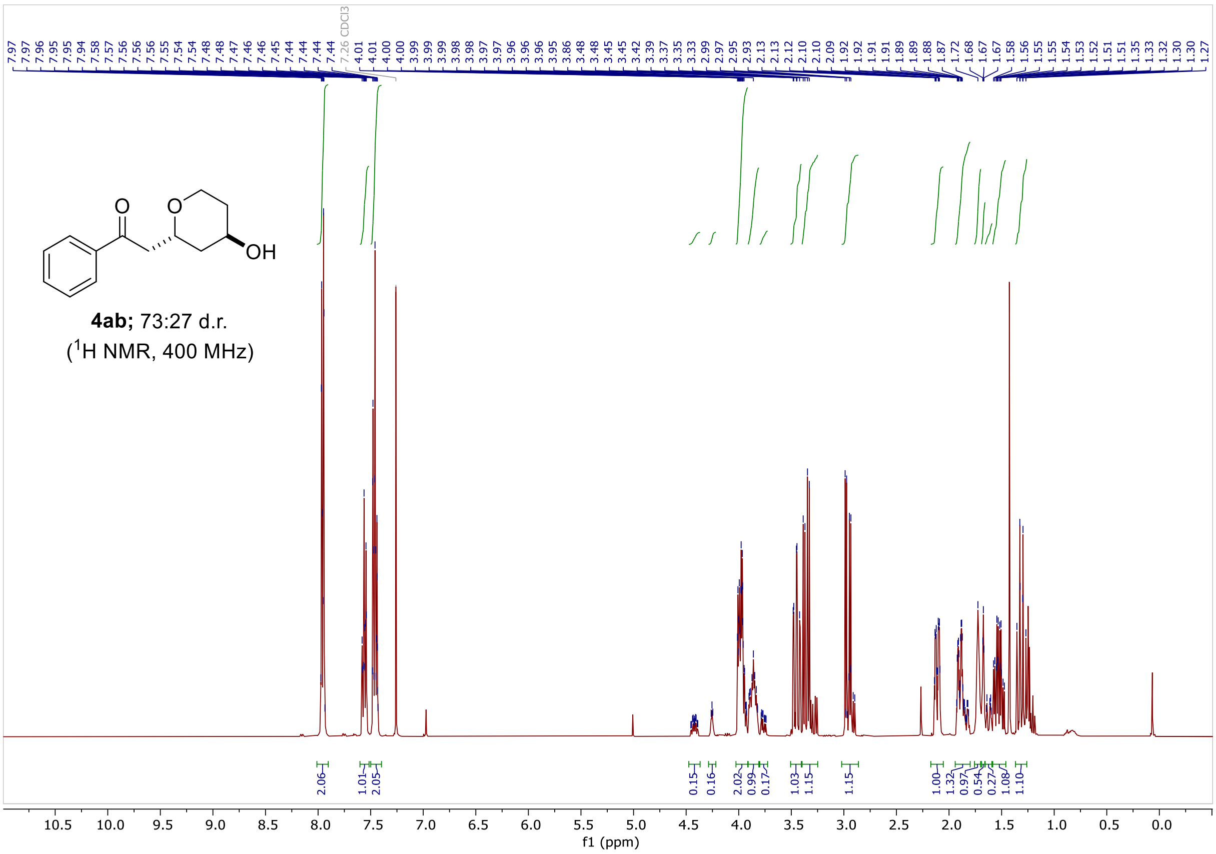
4ab; 73:27 d.r.
(1H NMR, 400 MHz)
7.97 7.97 7.96 7.95 7.95 7.94 7.58 7.57 7.56 7.56 7.55 7.54 7.54 7.48 7.48 7.47 7.46 7.46 7.45 7.44 7.44 7.44 7.26 CDCl3 4.01 4.01 4.00 4.00 3.99 3.99 3.99 3.98 3.98 3.97 3.97 3.96 3.96 3.96 3.95 3.86 3.48 3.48 3.45 3.45 3.42 3.39 3.37 3.35 3.33 2.99 2.97 2.95 2.93 2.13 2.13 2.12 2.10 2.10 2.09 1.92 1.92 1.91 1.91 1.89 1.89 1.88 1.87 1.72 1.68 1.67 1.67 1.58 1.56 1.55 1.55 1.54 1.53 1.52 1.51 1.51 1.35 1.33 1.32 1.30 1.27
2.06 1.01 2.05 0.15 0.16 2.02 0.99 0.17 1.03 1.15 1.15 1.00 1.32 0.97 0.54 0.27 1.08 1.10
f1 (ppm)

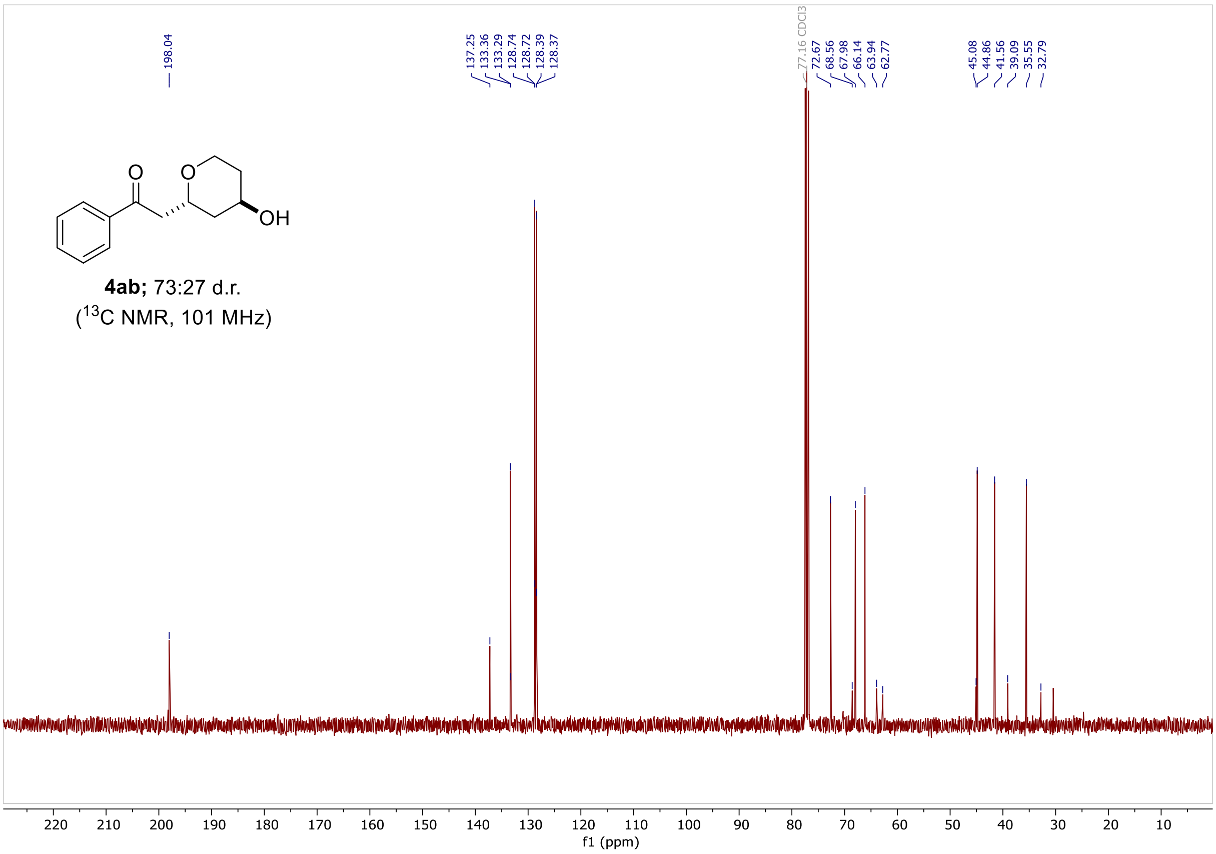
4ab; 73:27 d.r.
(13C NMR, 101 MHz)
198.04
137.25 133.36 133.29 128.74 128.72 128.39 128.37
77.16 CDCl3 72.67 68.56 67.98 66.14 63.94 62.77
45.08 44.86 41.56 39.09 35.55 32.79
f1 (ppm)

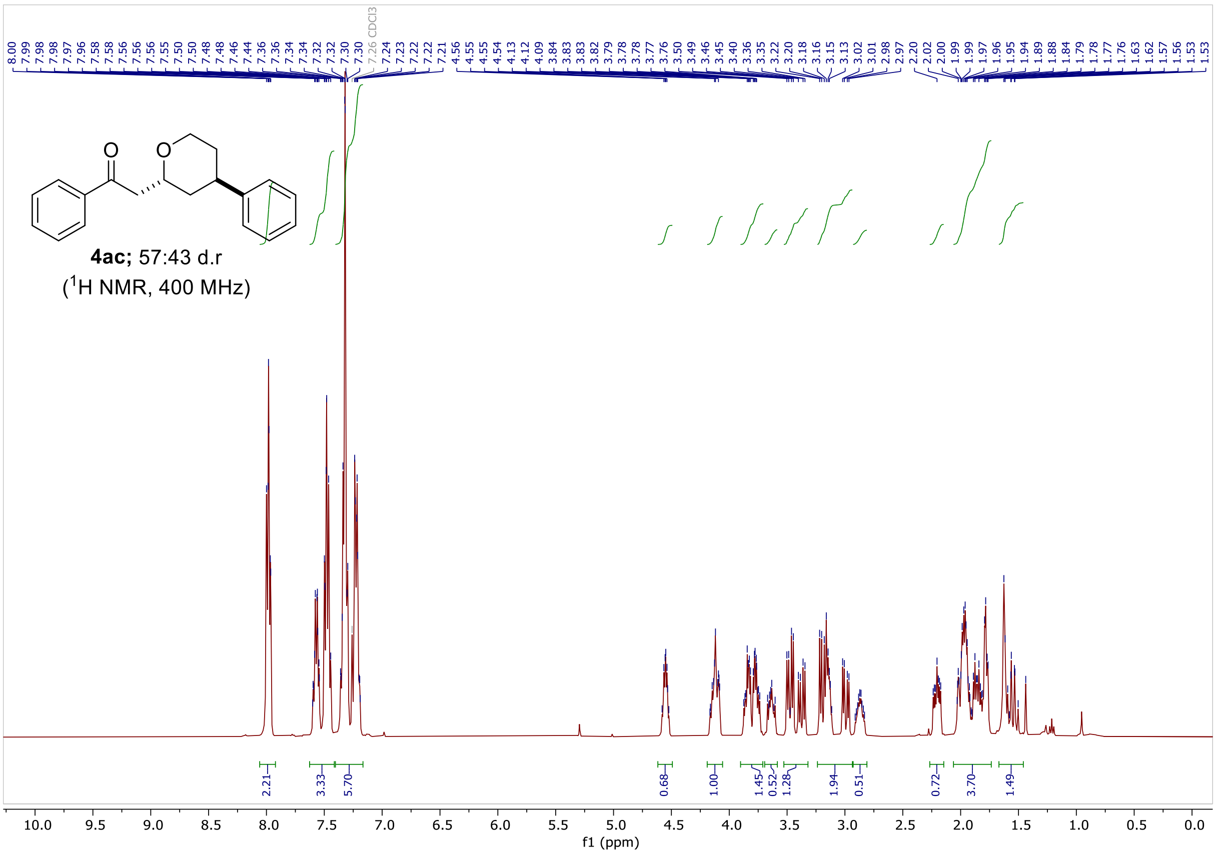

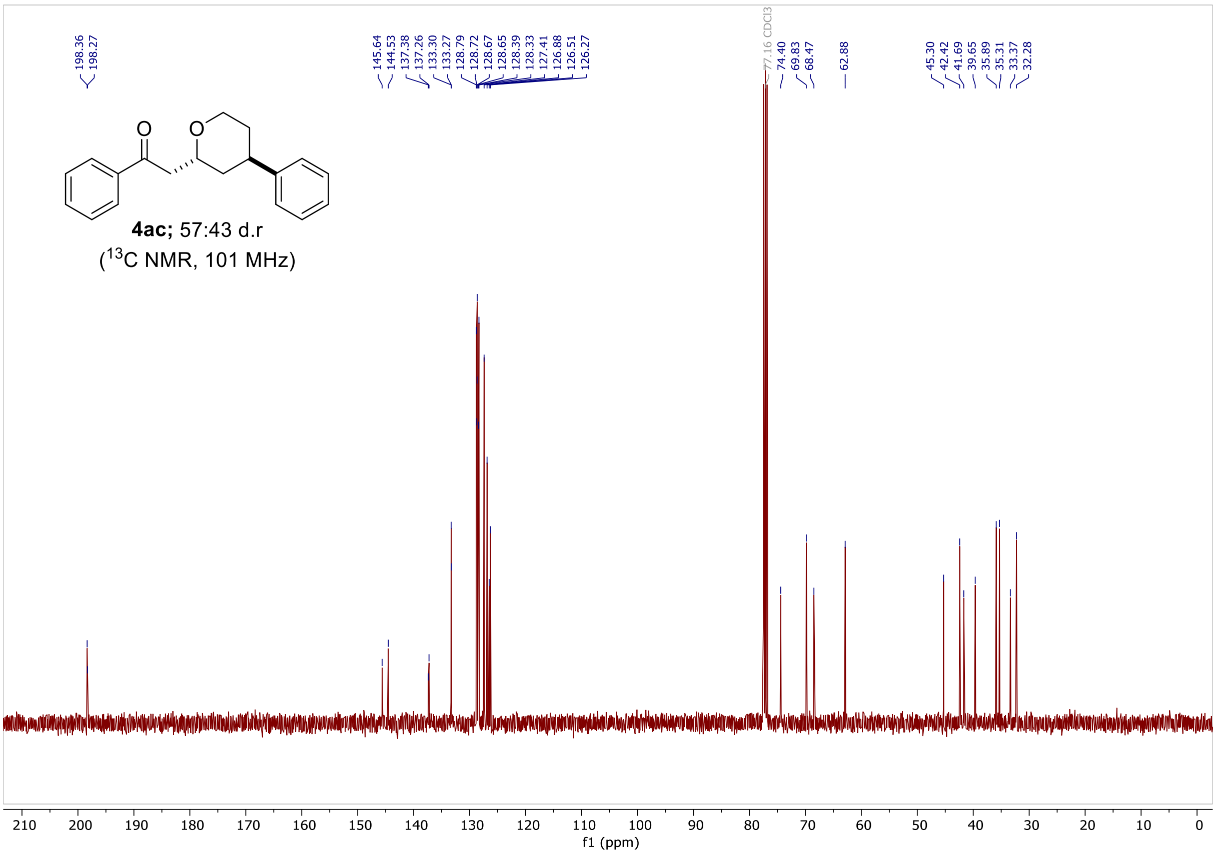

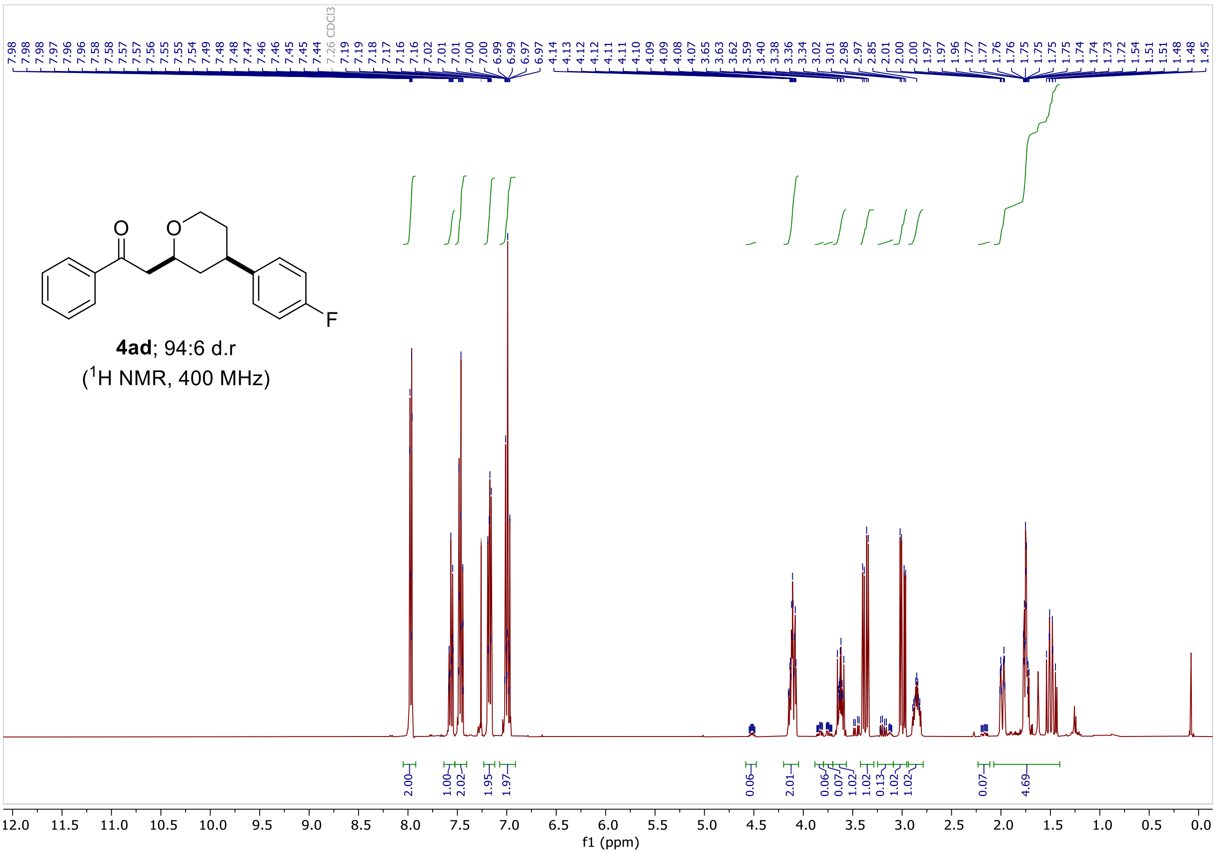
7.98 7.98 7.98 7.97 7.96 7.96 7.58 7.58 7.57 7.57 7.56 7.55 7.55 7.54 7.49 7.48 7.48 7.47 7.46 7.46 7.45 7.45 7.44 7.26 CDCl3 7.19 7.19 7.18 7.17 7.16 7.16 7.02 7.01 7.01 7.00 6.99 6.99 6.97 6.97 4.14 4.13 4.12 4.11 4.11 4.10 4.09 4.09 4.08 4.07 3.65 3.63 3.62 3.59 3.40 3.38 3.36 3.34 3.02 3.01 2.98 2.97 2.85 2.01 2.00 1.97 1.97 1.96 1.77 1.77 1.76 1.75 1.75 1.75 1.75 1.74 1.73 1.72 1.54 1.51 1.51 1.48 1.45
O
O
F
4ad; 94:6 d.r
(1H NMR, 400 MHz)
2.00 1.00 2.02 1.95 1.97 0.06 2.01 0.06 0.07 1.02 0.13 1.02 1.02 0.07 4.69
12.0 11.5 11.0 10.5 10.0 9.5 9.0 8.5 8.0 7.5 7.0 6.5 6.0 5.5 5.0 4.5 4.0 3.5 3.0 2.5 2.0 1.5 1.0 0.5 0.0
f1 (ppm)

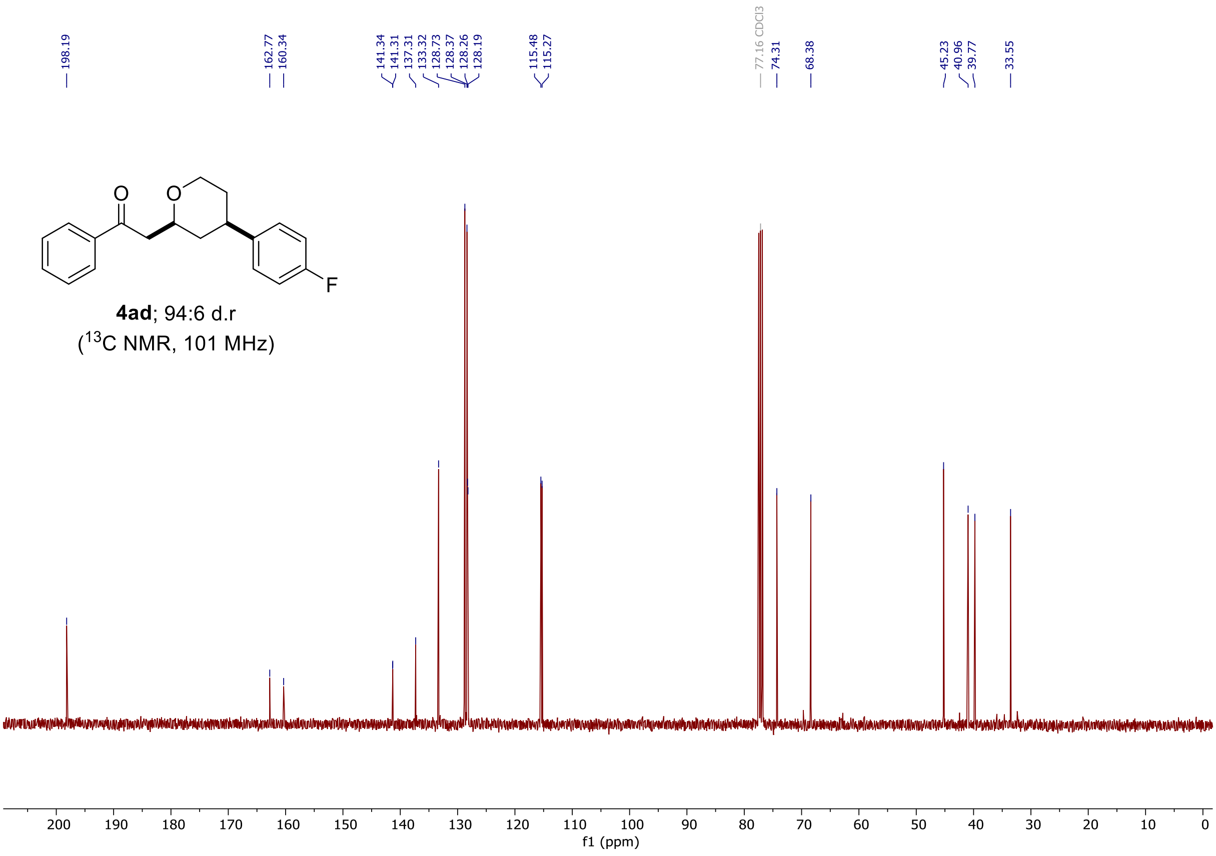
198.19
162.77 160.34
141.34 141.31 137.31 133.32 128.73 128.37 128.26 128.19
115.48 115.27
77.16 CDCl3 74.31 68.38
45.23 40.96 39.77 33.55
O
O
F
4ad; 94:6 d.r
(13C NMR, 101 MHz)
200 190 180 170 160 150 140 130 120 110 100 90 80 70 60 50 40 30 20 10 0
f1 (ppm)

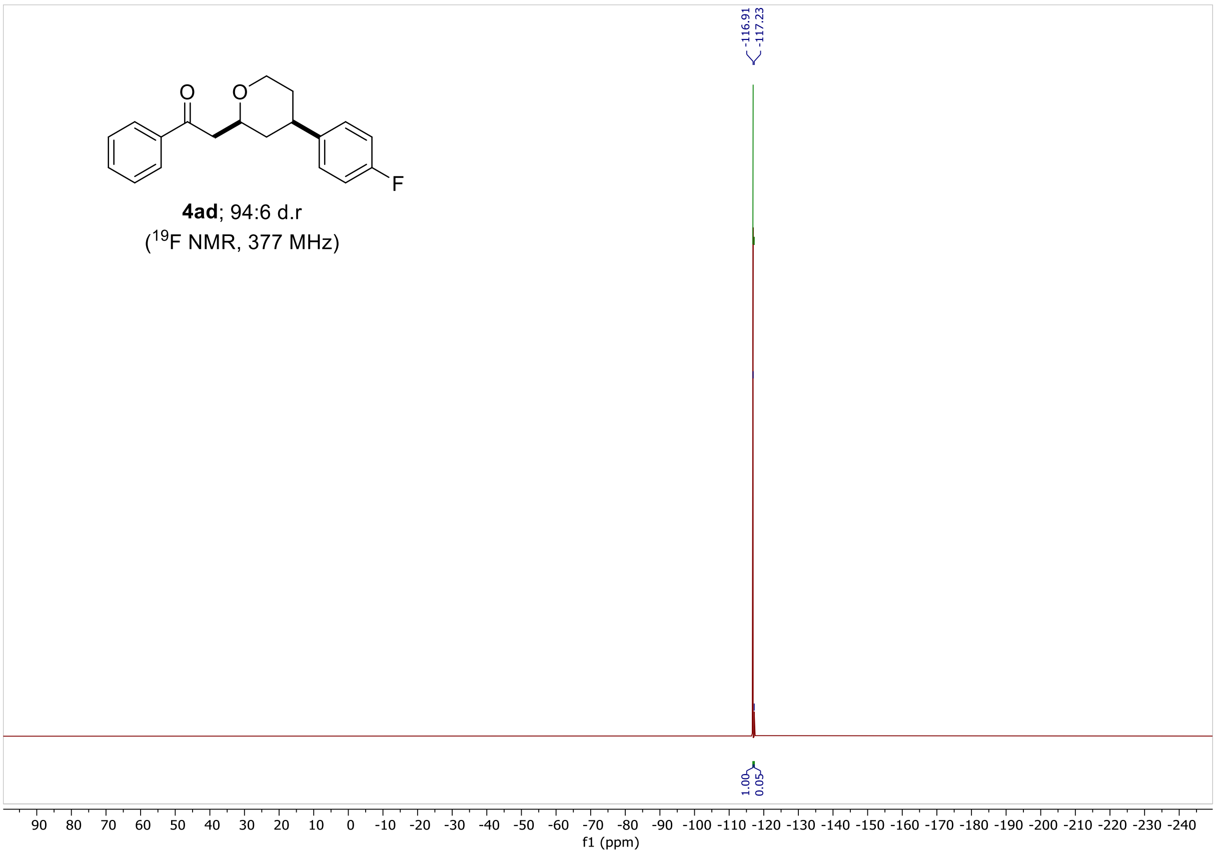
4ad; 94:6 d.r
(^{19}F NMR, 377 MHz)
-116.91
-117.23
1.00
0.05
90 80 70 60 50 40 30 20 10 0 -10 -20 -30 -40 -50 -60 -70 -80 -90 -100 -110 -120 -130 -140 -150 -160 -170 -180 -190 -200 -210 -220 -230 -240
f1 (ppm)

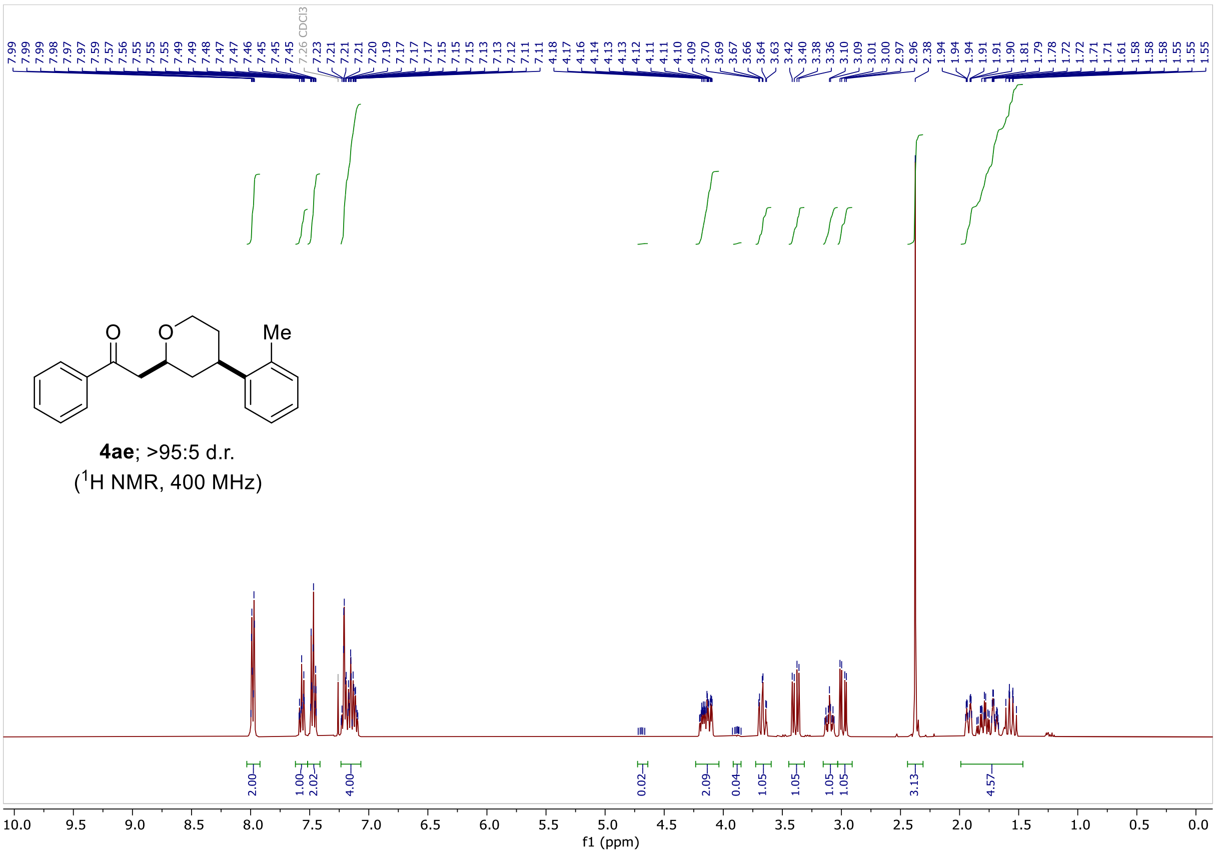
4ae; >95:5 d.r.
(1H NMR, 400 MHz)
f1 (ppm)

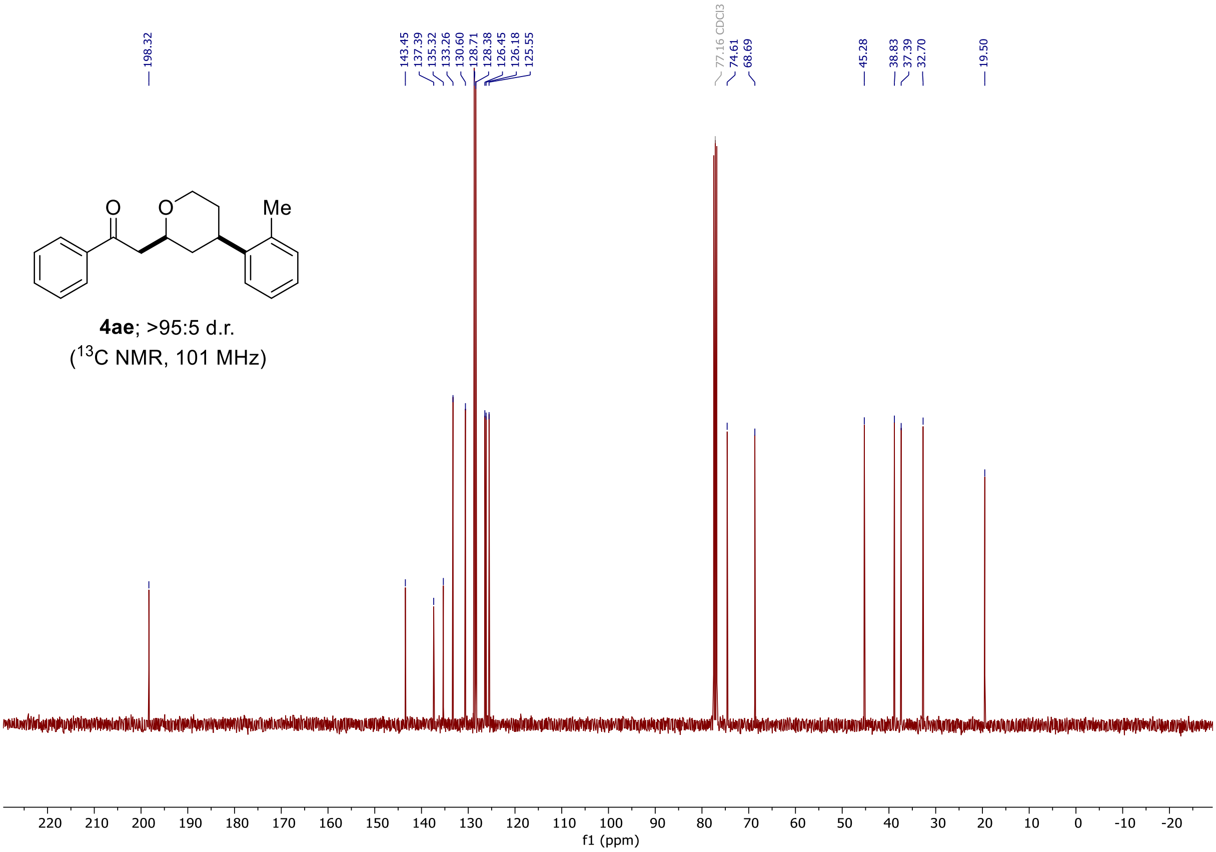
4ae; >95:5 d.r.
(13C NMR, 101 MHz)
198.32
143.45
137.39
135.32
133.26
130.60
128.71
128.38
126.45
126.18
125.55
77.16 CDCl3
74.61
68.69
45.28
38.83
37.39
32.70
19.50
f1 (ppm)

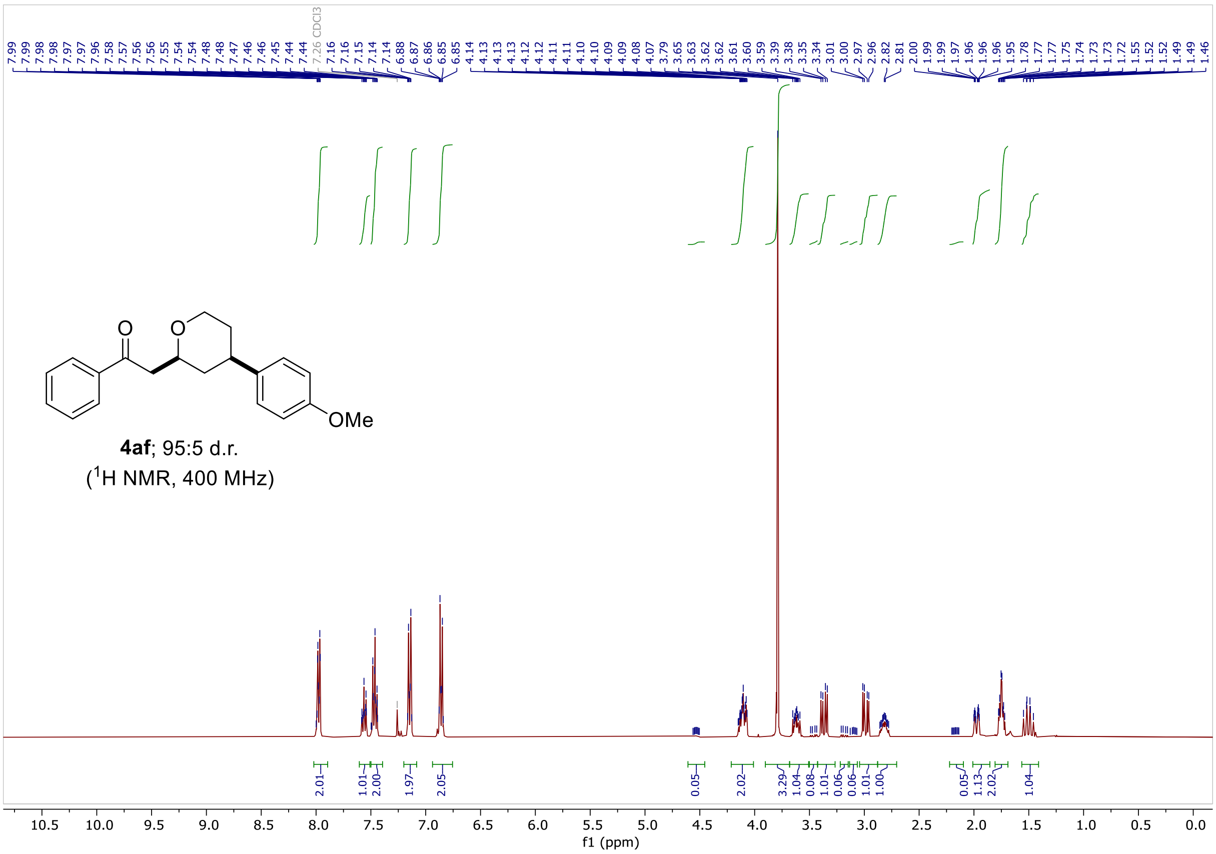
4af; 95:5 d.r.
(1H NMR, 400 MHz)
OMe
f1 (ppm)

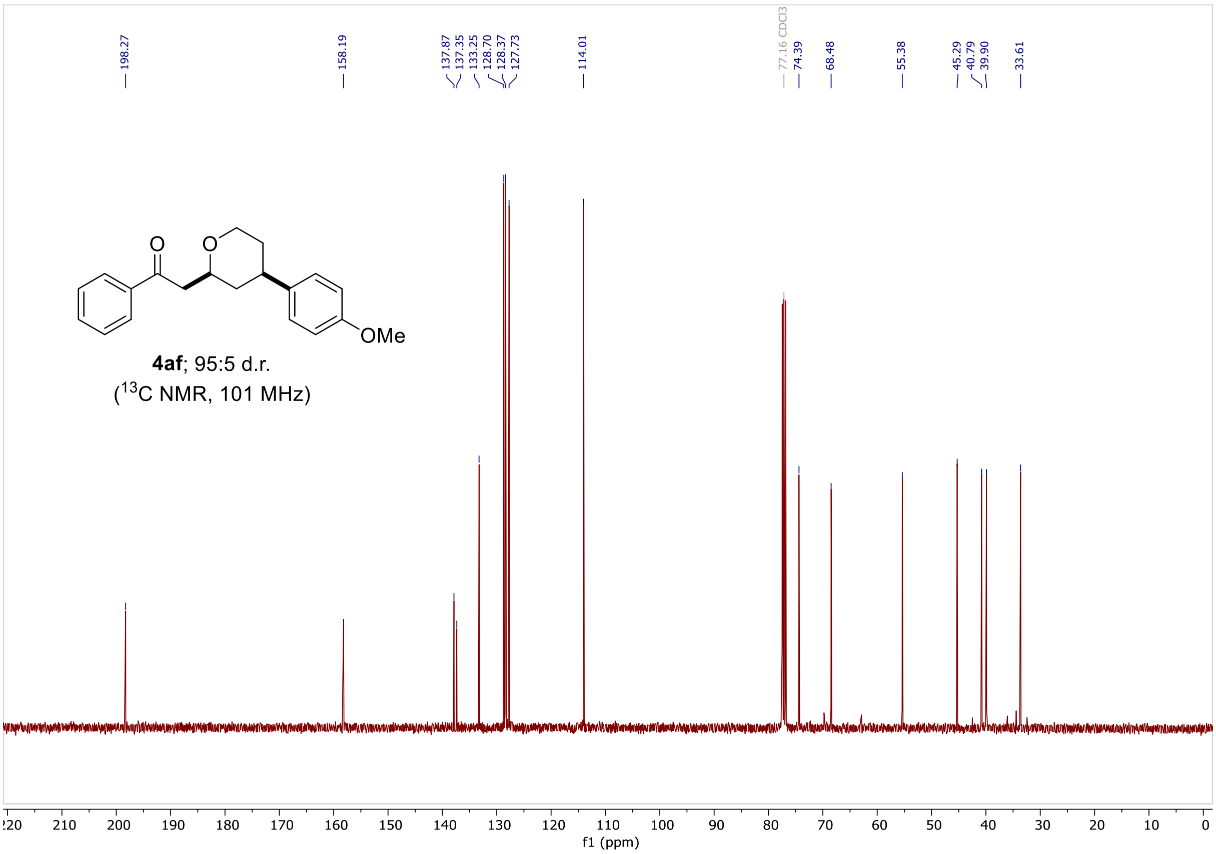
4af; 95:5 d.r.
(13C NMR, 101 MHz)
OMe
198.27
158.19
137.87
137.35
133.25
128.70
128.37
127.73
114.01
77.16 CDCl3
74.39
68.48
55.38
45.29
40.79
39.90
33.61
f1 (ppm)

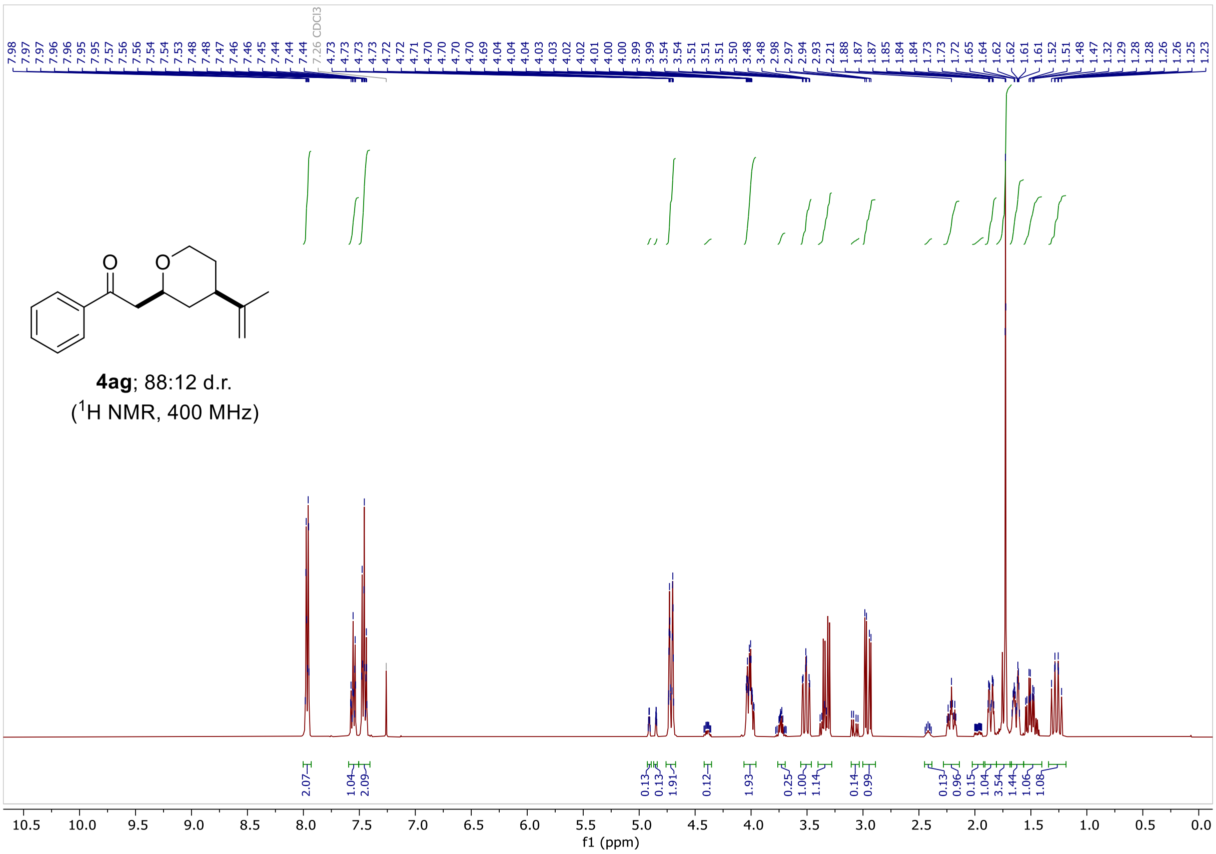
7.98 7.97 7.97 7.96 7.96 7.95 7.95 7.57 7.56 7.56 7.54 7.54 7.53 7.48 7.47 7.46 7.46 7.45 7.44 7.44 7.44 7.26 CDCl3 4.73 4.73 4.73 4.73 4.72 4.72 4.71 4.70 4.70 4.70 4.70 4.69 4.04 4.04 4.04 4.03 4.03 4.02 4.02 4.01 4.00 4.00 3.99 3.99 3.54 3.54 3.51 3.51 3.51 3.50 3.48 3.48 2.98 2.97 2.94 2.93 2.21 1.88 1.87 1.87 1.85 1.84 1.84 1.73 1.73 1.72 1.65 1.64 1.62 1.62 1.61 1.61 1.52 1.51 1.48 1.47 1.32 1.29 1.28 1.28 1.26 1.25 1.23
O
O
4ag; 88:12 d.r.
(1H NMR, 400 MHz)
2.07 1.04 2.09 0.13 0.13 1.91 0.12 1.93 0.25 1.00 1.14 0.14 0.99 0.13 0.96 0.15 1.04 3.54 1.44 1.06 1.08
10.5 10.0 9.5 9.0 8.5 8.0 7.5 7.0 6.5 6.0 5.5 5.0 4.5 4.0 3.5 3.0 2.5 2.0 1.5 1.0 0.5 0.0
f1 (ppm)

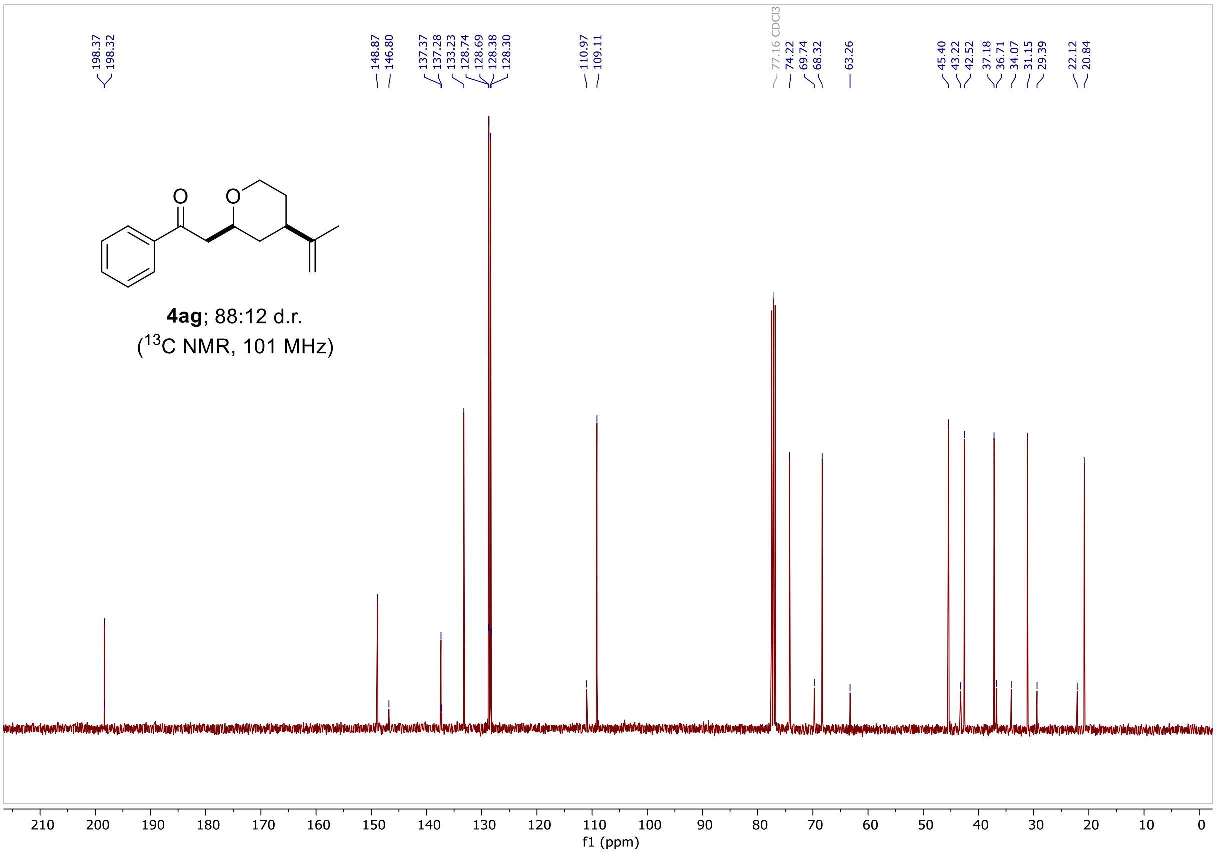
198.37 198.32
148.87 146.80
137.37 137.28 133.23 128.74 128.69 128.38 128.30
110.97 109.11
77.16 CDCl3
74.22 69.74 68.32
63.26
45.40 43.22 42.52 37.18 36.71 34.07 31.15 29.39
22.12 20.84
O
O
4ag; 88:12 d.r.
(13C NMR, 101 MHz)
210 200 190 180 170 160 150 140 130 120 110 100 90 80 70 60 50 40 30 20 10 0
f1 (ppm)

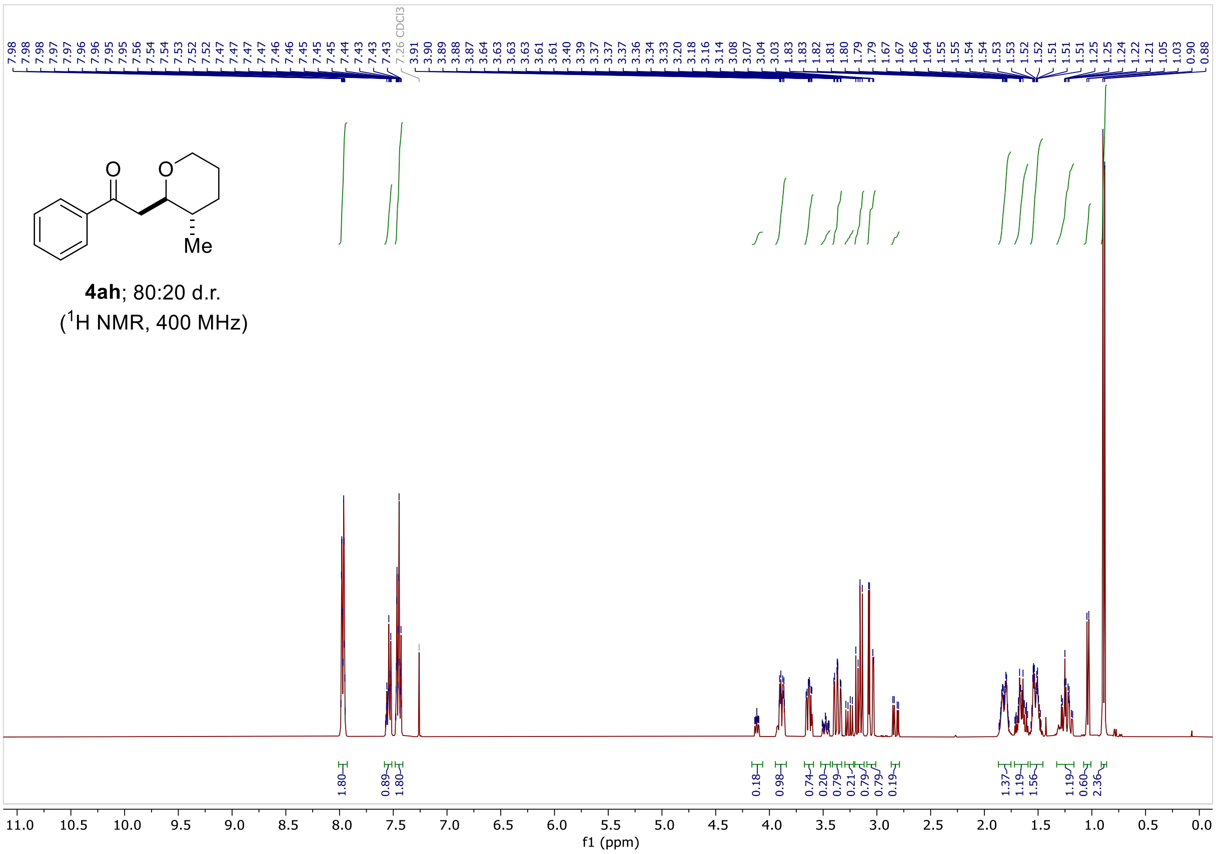
7.98 7.98 7.98 7.97 7.97 7.96 7.96 7.95 7.95 7.56 7.54 7.54 7.53 7.52 7.52 7.47 7.47 7.47 7.46 7.46 7.45 7.45 7.45 7.44 7.43 7.43 7.26 CDCl3 3.91 3.90 3.89 3.88 3.87 3.64 3.63 3.63 3.63 3.61 3.61 3.40 3.39 3.37 3.37 3.37 3.36 3.34 3.33 3.20 3.18 3.16 3.14 3.08 3.07 3.04 3.03 1.83 1.83 1.82 1.81 1.80 1.79 1.79 1.67 1.67 1.66 1.64 1.55 1.55 1.54 1.54 1.53 1.53 1.52 1.52 1.51 1.51 1.51 1.25 1.25 1.24 1.22 1.21 1.05 1.03 0.90 0.88
O
O
Me
4ah; 80:20 d.r.
(1H NMR, 400 MHz)
1.80 0.89 1.80 0.18 0.98 0.74 0.20 0.79 0.21 0.79 0.79 0.19 1.37 1.19 1.56 1.19 0.60 2.36
f1 (ppm)

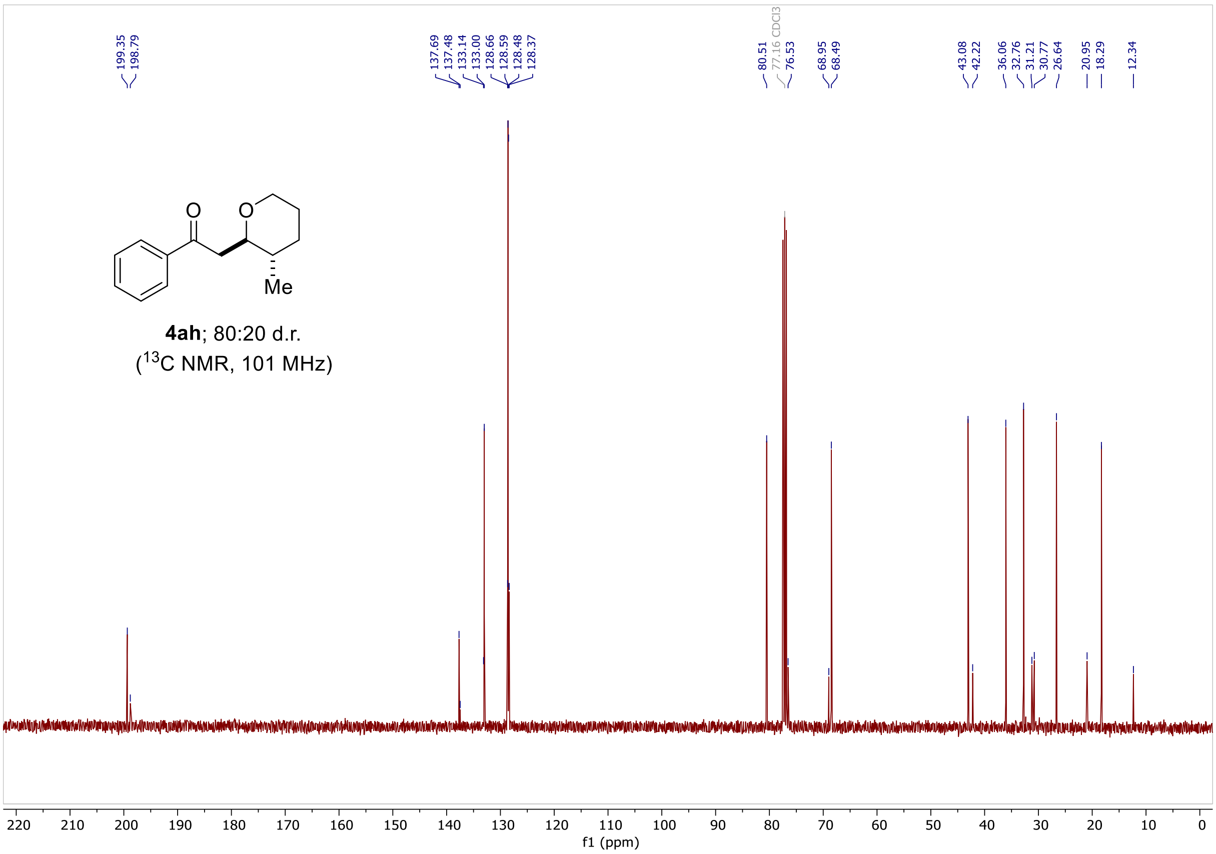
199.35 198.79 137.69 137.48 133.14 133.00 128.66 128.59 128.48 128.37 80.51 77.16 CDCl3 76.53 68.95 68.49 43.08 42.22 36.06 32.76 31.21 30.77 26.64 20.95 18.29 12.34
O
O
Me
4ah; 80:20 d.r.
(13C NMR, 101 MHz)
f1 (ppm)

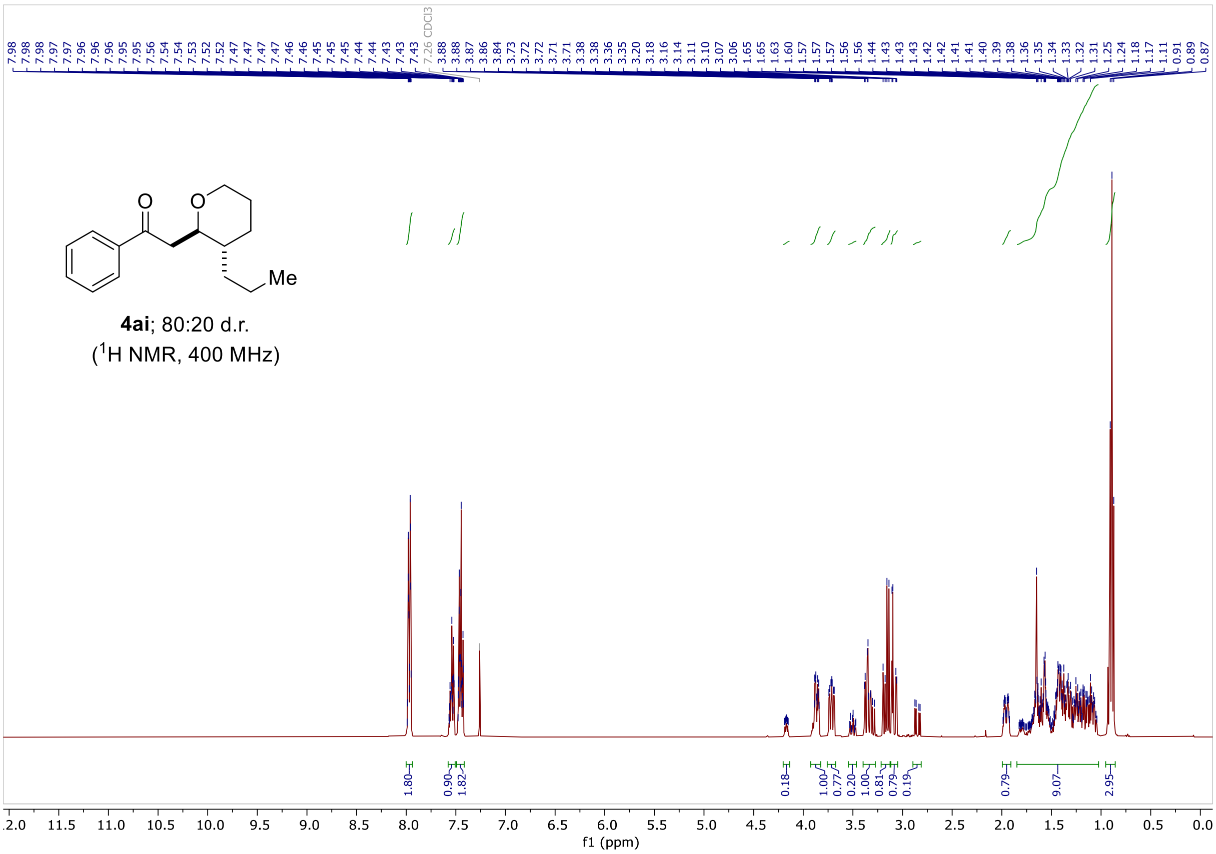

4ai; 80:20 d.r.
(^{1}H NMR, 400 MHz)

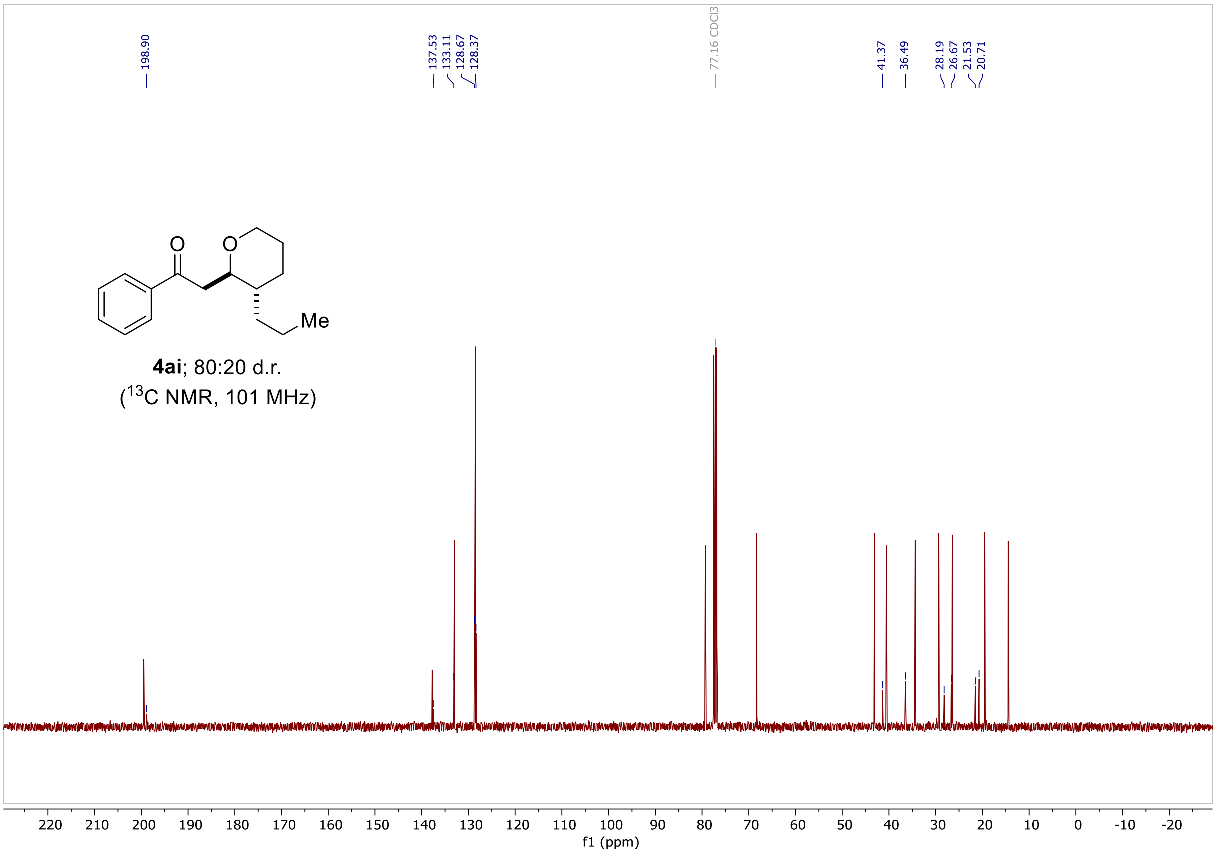

4ai; 80:20 d.r.
(^{13}C NMR, 101 MHz)

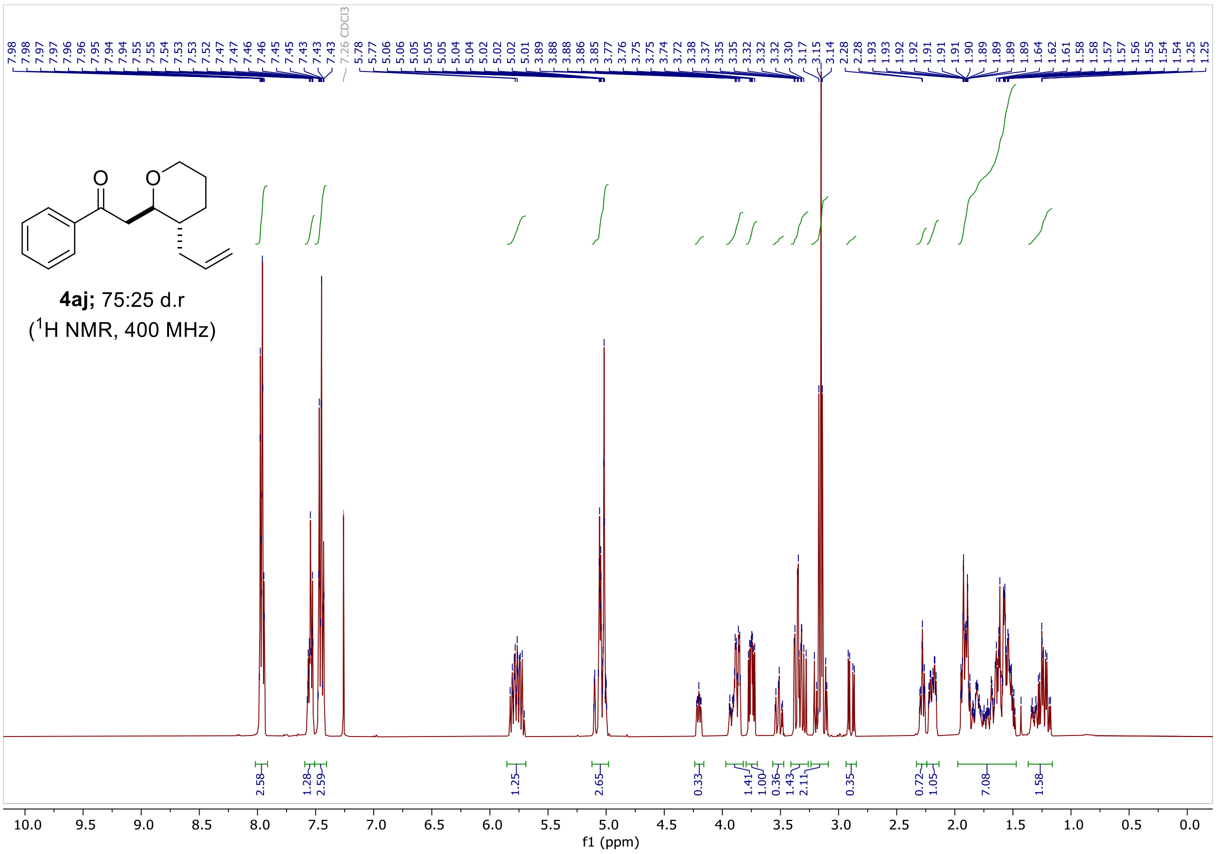
4aj; 75:25 d.r
(1H NMR, 400 MHz)
f1 (ppm)

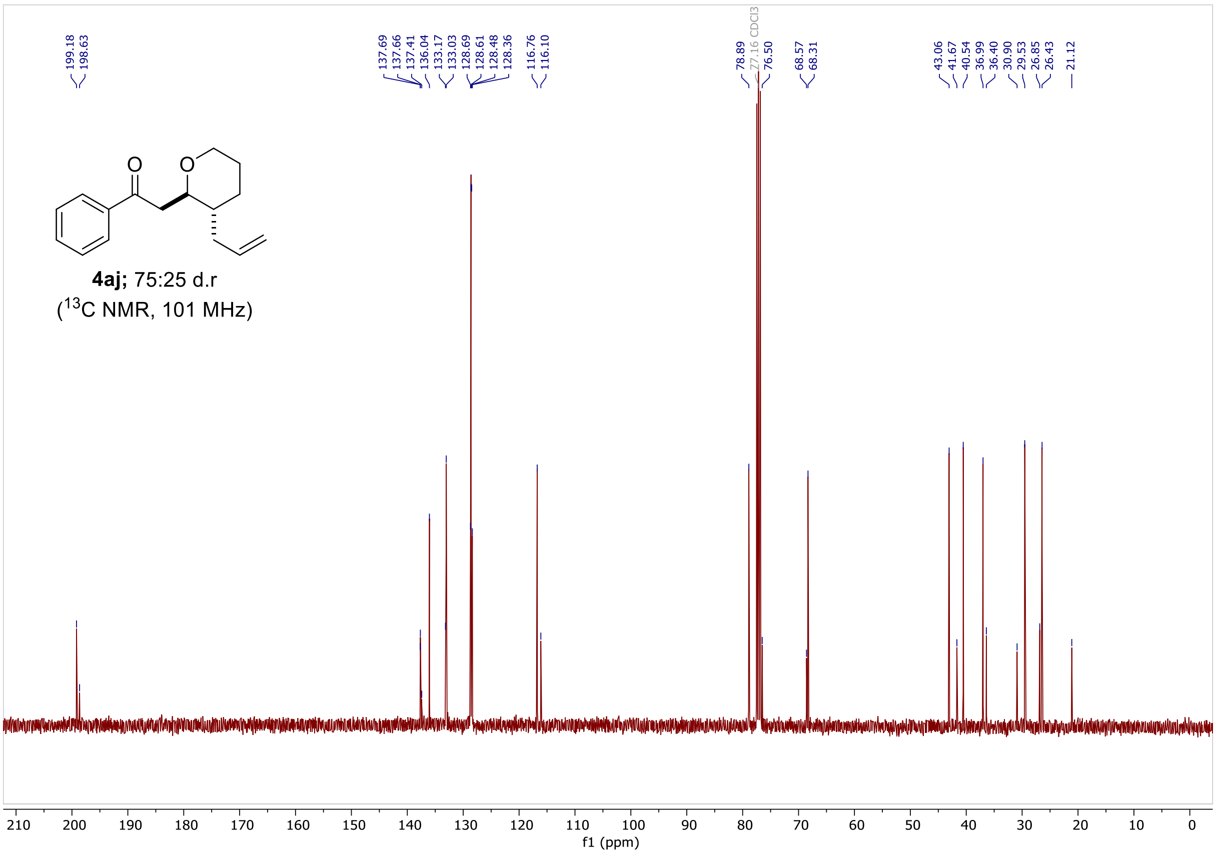
4aj; 75:25 d.r
(13C NMR, 101 MHz)
f1 (ppm)

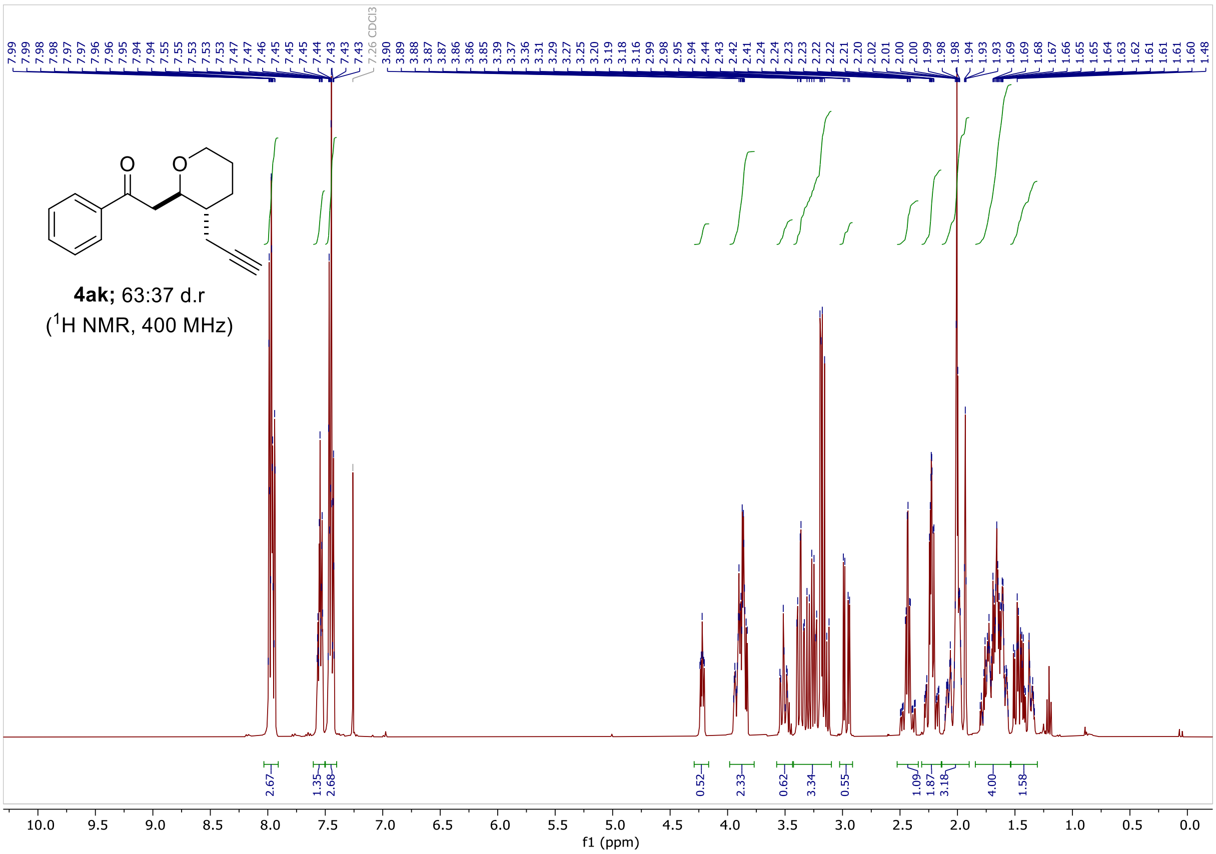
7.99 7.99 7.98 7.98 7.97 7.97 7.96 7.95 7.95 7.94 7.94 7.55 7.55 7.53 7.53 7.47 7.46 7.45 7.45 7.45 7.44 7.43 7.43 7.43
7.26 CDCl3
3.90 3.89 3.88 3.87 3.87 3.86 3.85 3.39 3.37 3.36 3.31 3.29 3.27 3.25 3.20 3.19 3.18 3.16 2.99 2.98 2.95 2.94 2.44 2.43 2.42 2.41 2.24 2.24 2.23 2.23 2.22 2.22 2.21 2.20 2.02 2.01 2.00 2.00 1.99 1.98 1.98 1.94 1.93 1.93 1.69 1.69 1.68 1.67 1.66 1.65 1.65 1.64 1.63 1.62 1.61 1.61 1.61 1.60 1.48
O
O
4ak; 63:37 d.r
(^{1}H NMR, 400 MHz)
2.67
1.35
2.68
0.52
2.33
0.62
3.34
0.55
1.09
1.87
3.18
4.00
1.58
10.0 9.5 9.0 8.5 8.0 7.5 7.0 6.5 6.0 5.5 5.0 4.5 4.0 3.5 3.0 2.5 2.0 1.5 1.0 0.5 0.0
f1 (ppm)

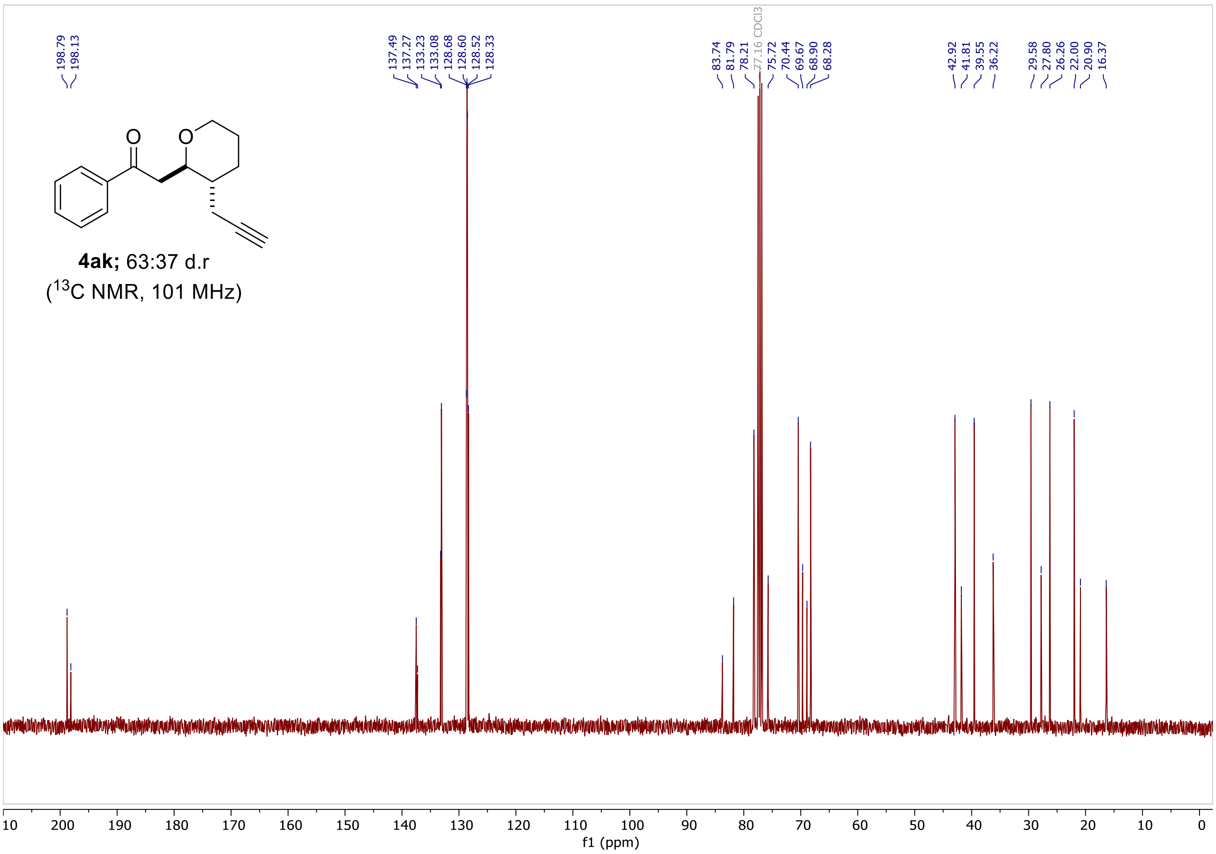
198.79 198.13
137.49 137.27 133.23 133.08 128.68 128.60 128.52 128.33
83.74 81.79 78.21
77.16 CDCl3
75.72 70.44 69.67 68.90 68.28
42.92 41.81 39.55 36.22
29.58 27.80 26.26 22.00 20.90 16.37
O
O
4ak; 63:37 d.r
(^{13}C NMR, 101 MHz)
10 200 190 180 170 160 150 140 130 120 110 100 90 80 70 60 50 40 30 20 10 0
f1 (ppm)

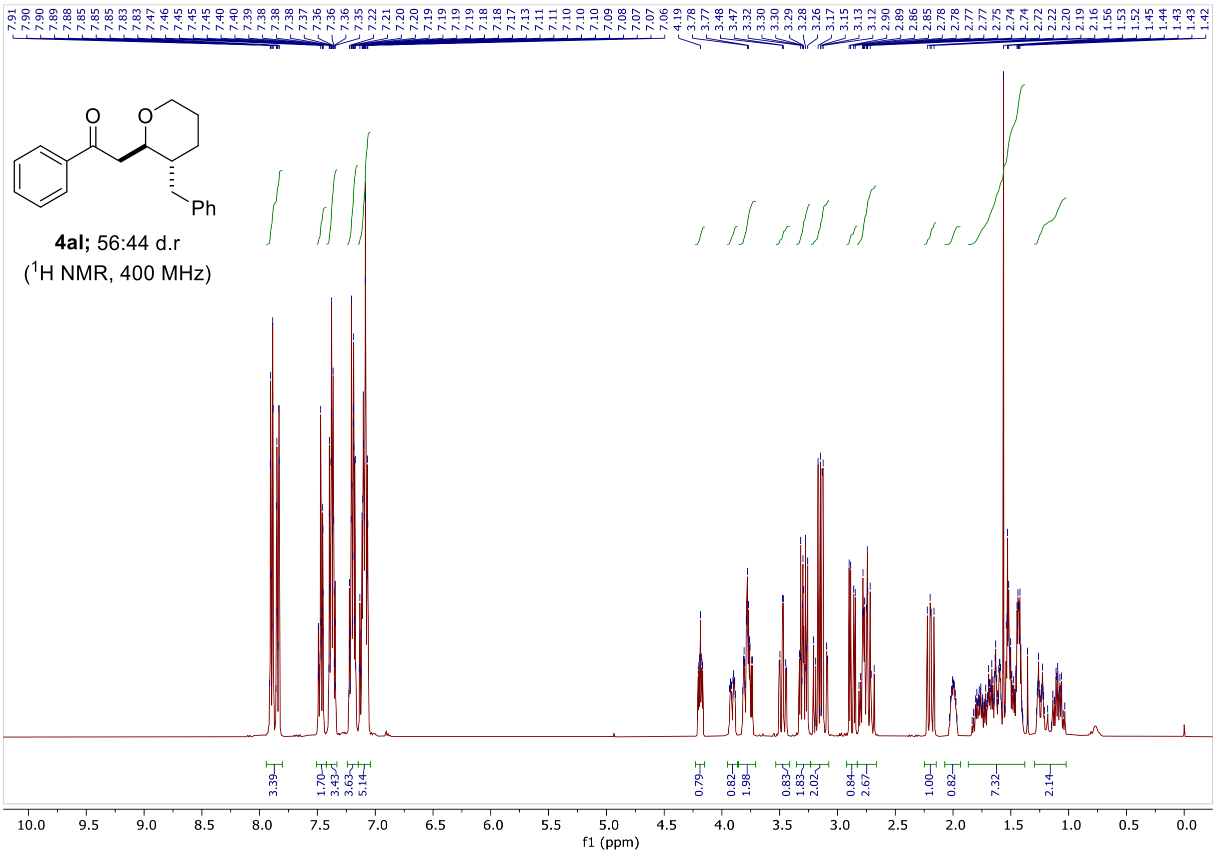
4al; 56:44 d.r
(1H NMR, 400 MHz)
f1 (ppm)

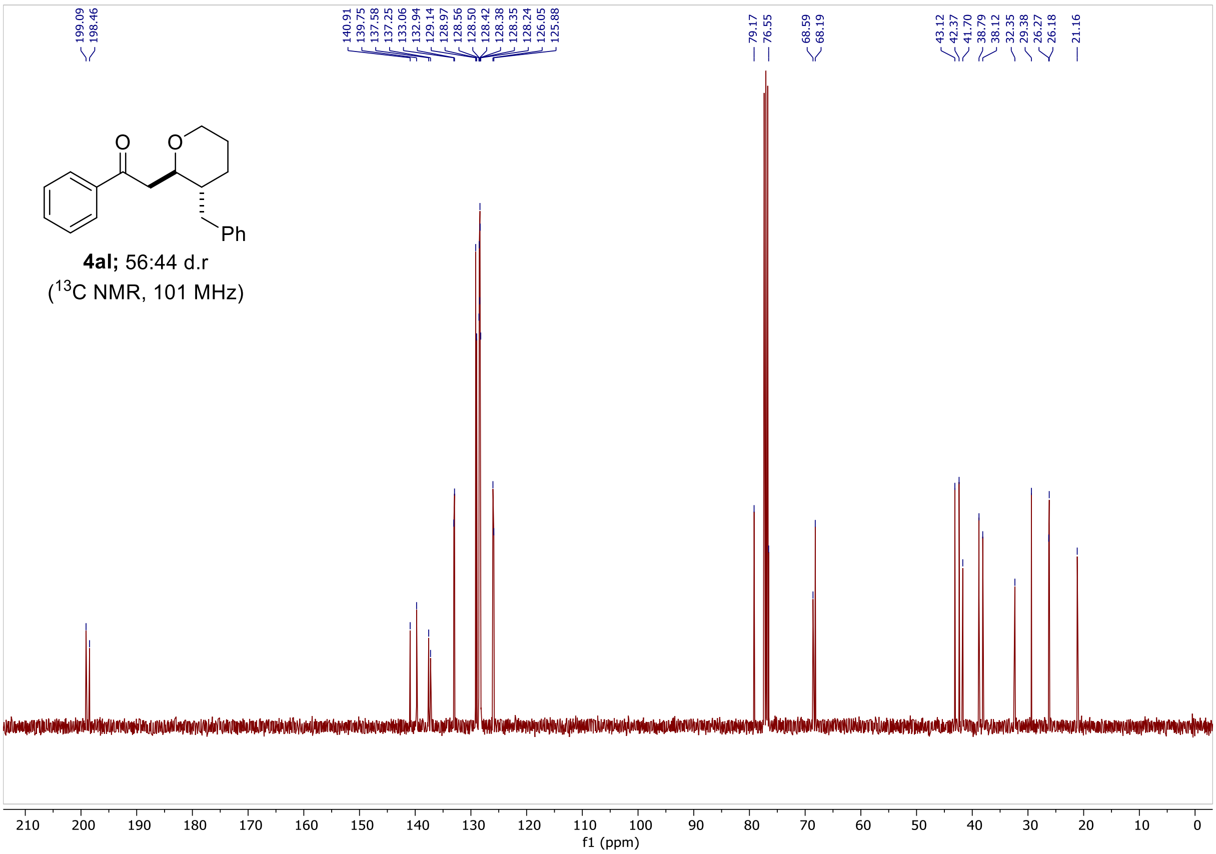
4al; 56:44 d.r
(13C NMR, 101 MHz)
199.09
198.46
140.91
139.75
137.58
137.25
133.06
132.94
129.14
128.97
128.56
128.50
128.42
128.38
128.35
128.24
126.05
125.88
79.17
76.55
68.59
68.19
43.12
42.37
41.70
38.79
38.12
32.35
29.38
26.27
26.18
21.16
f1 (ppm)

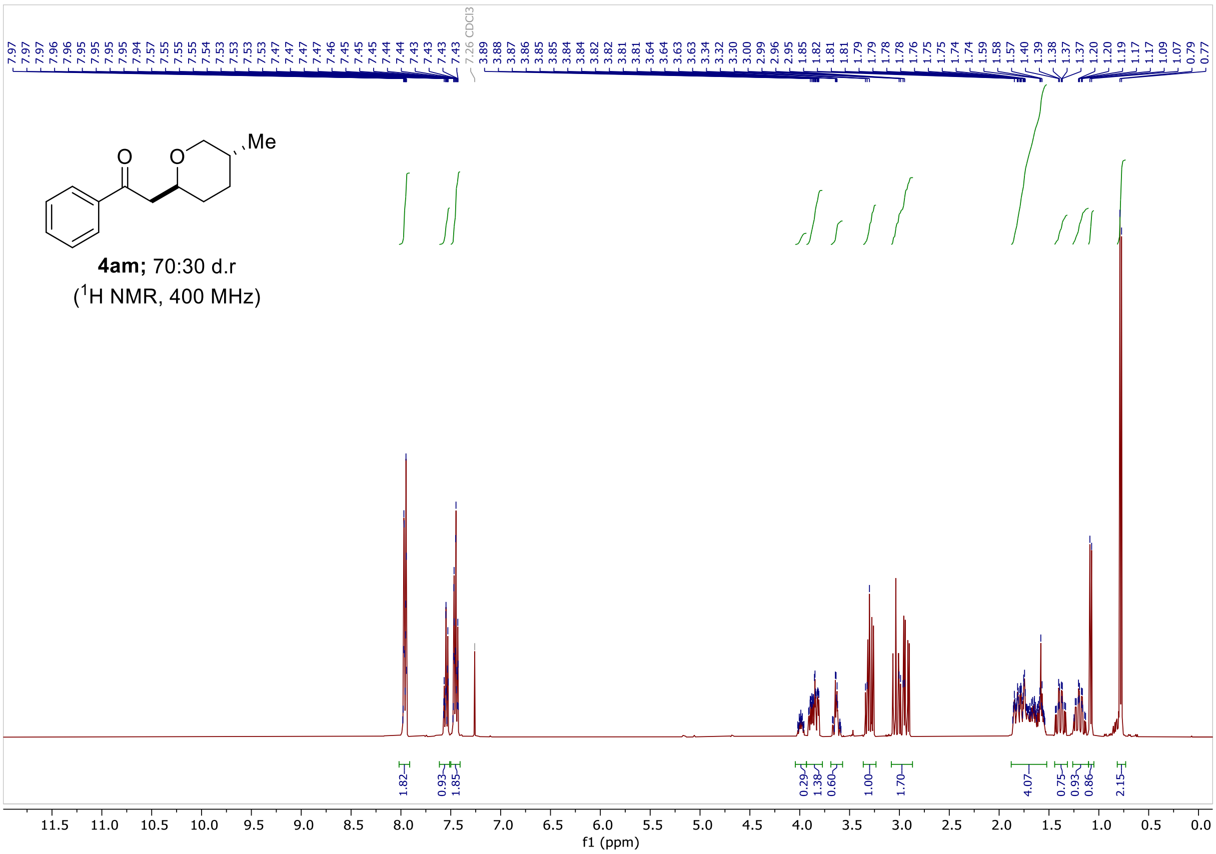

4am; 70:30 d.r
(1H NMR, 400 MHz)
f1 (ppm)

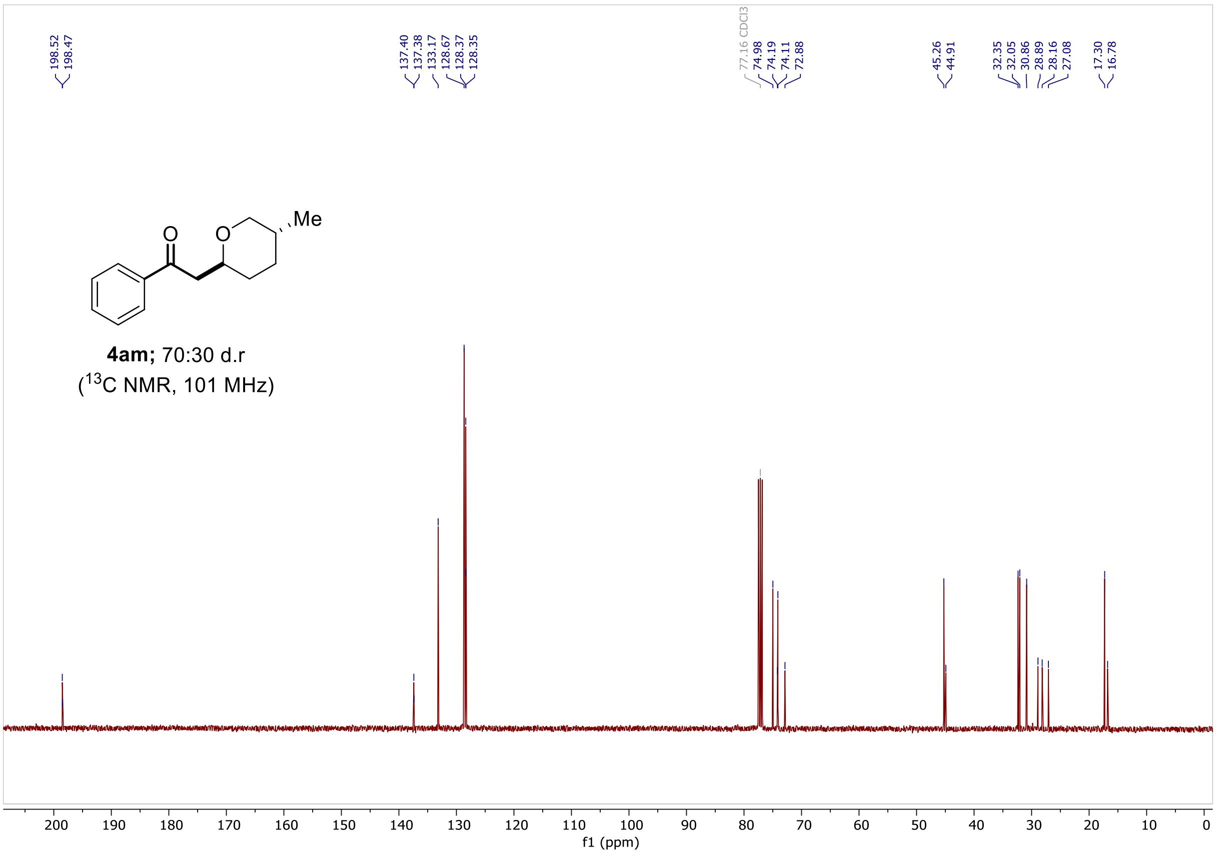

4am; 70:30 d.r
(13C NMR, 101 MHz)
f1 (ppm)

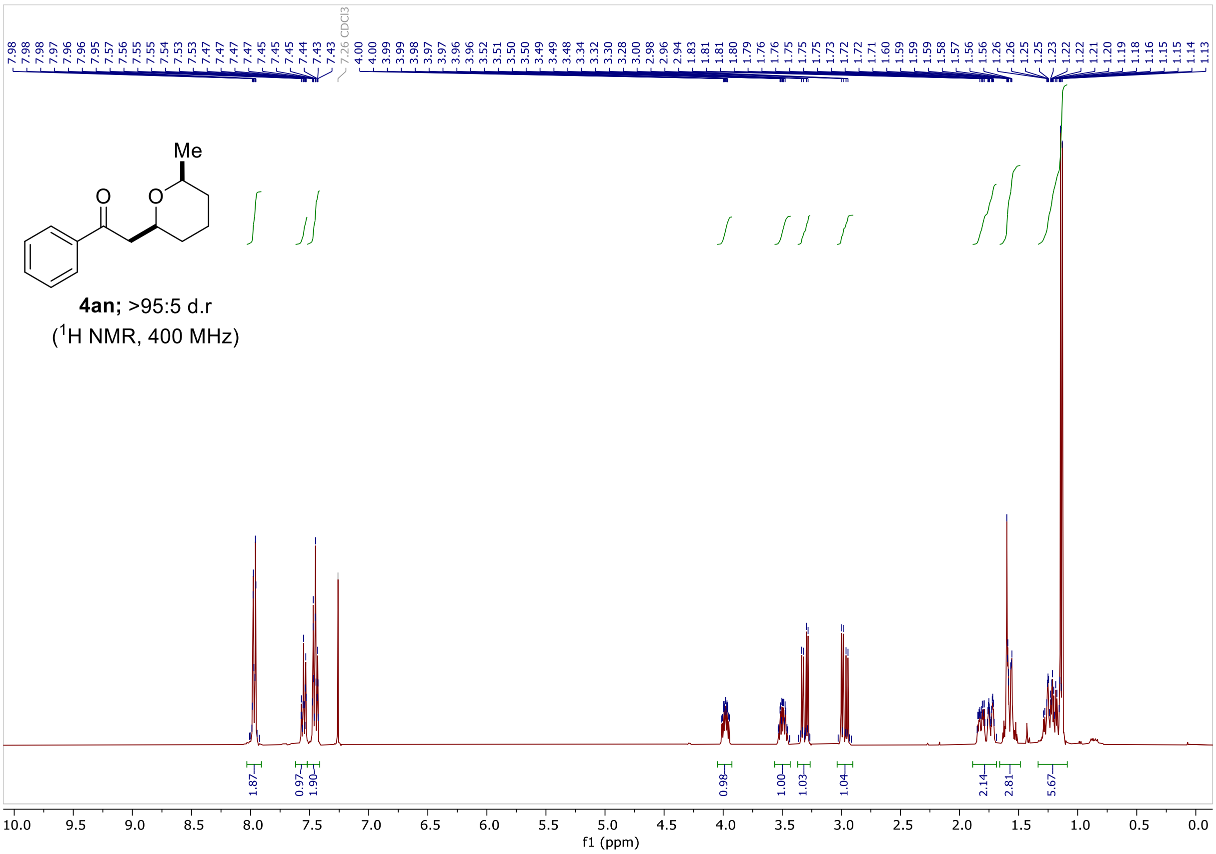

7.98
7.98
7.98
7.97
7.96
7.96
7.95
7.57
7.56
7.55
7.55
7.54
7.53
7.53
7.47
7.47
7.47
7.45
7.45
7.45
7.44
7.43
7.43
7.26 CDCl3
4.00
4.00
3.99
3.99
3.98
3.97
3.97
3.96
3.96
3.52
3.51
3.50
3.50
3.49
3.49
3.48
3.34
3.32
3.30
3.28
3.00
2.98
2.96
2.94
1.83
1.81
1.81
1.80
1.79
1.76
1.76
1.75
1.75
1.75
1.73
1.72
1.72
1.71
1.60
1.59
1.59
1.59
1.58
1.57
1.56
1.56
1.26
1.26
1.25
1.25
1.23
1.22
1.22
1.21
1.20
1.19
1.18
1.16
1.15
1.15
1.14
1.13
Me
O
O
4an; >95:5 d.r
(1H NMR, 400 MHz)
1.87
0.97
1.90
0.98
1.00
1.03
1.04
2.14
2.81
5.67
10.0
9.5
9.0
8.5
8.0
7.5
7.0
6.5
6.0
5.5
5.0
4.5
4.0
3.5
3.0
2.5
2.0
1.5
1.0
0.5
0.0
f1 (ppm)

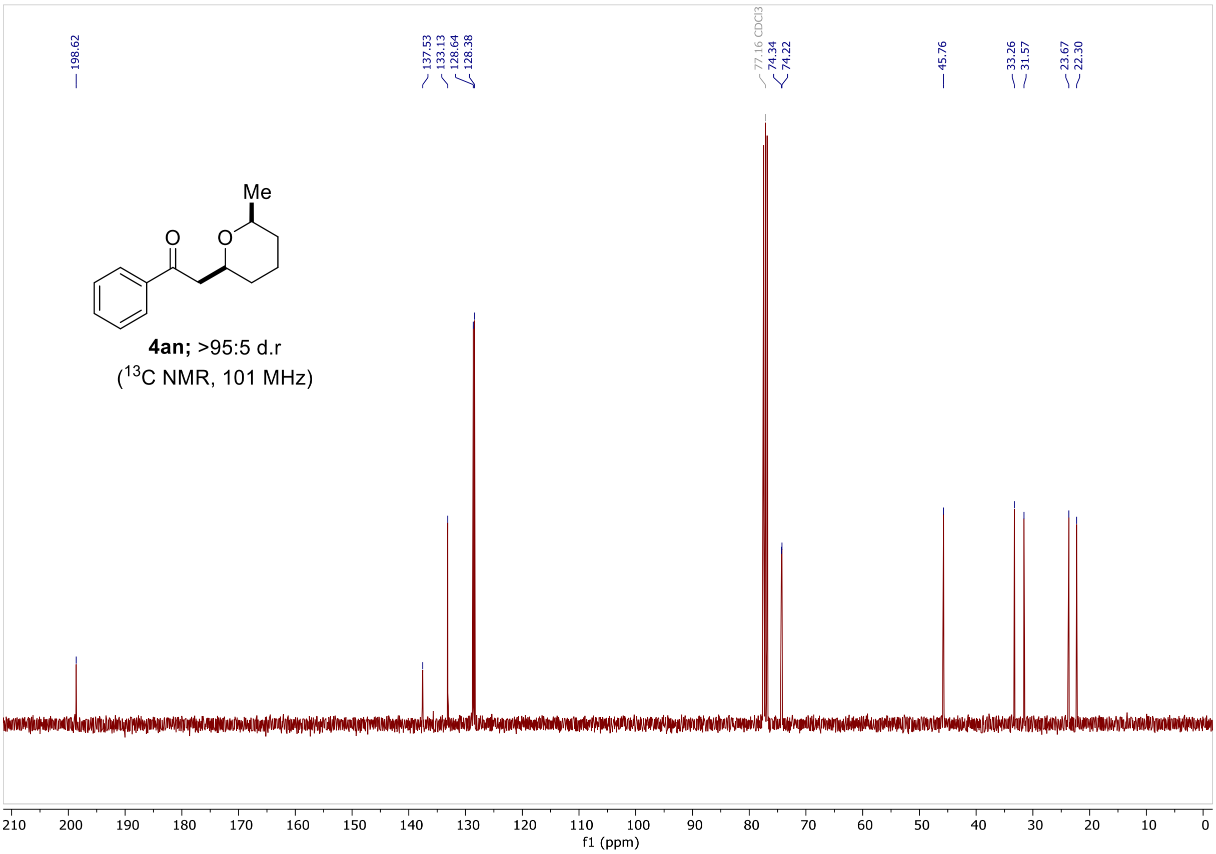

198.62
137.53
133.13
128.64
128.38
77.16 CDCl3
74.34
74.22
45.76
33.26
31.57
23.67
22.30
Me
O
O
4an; >95:5 d.r
(13C NMR, 101 MHz)
210
200
190
180
170
160
150
140
130
120
110
100
90
80
70
60
50
40
30
20
10
0
f1 (ppm)

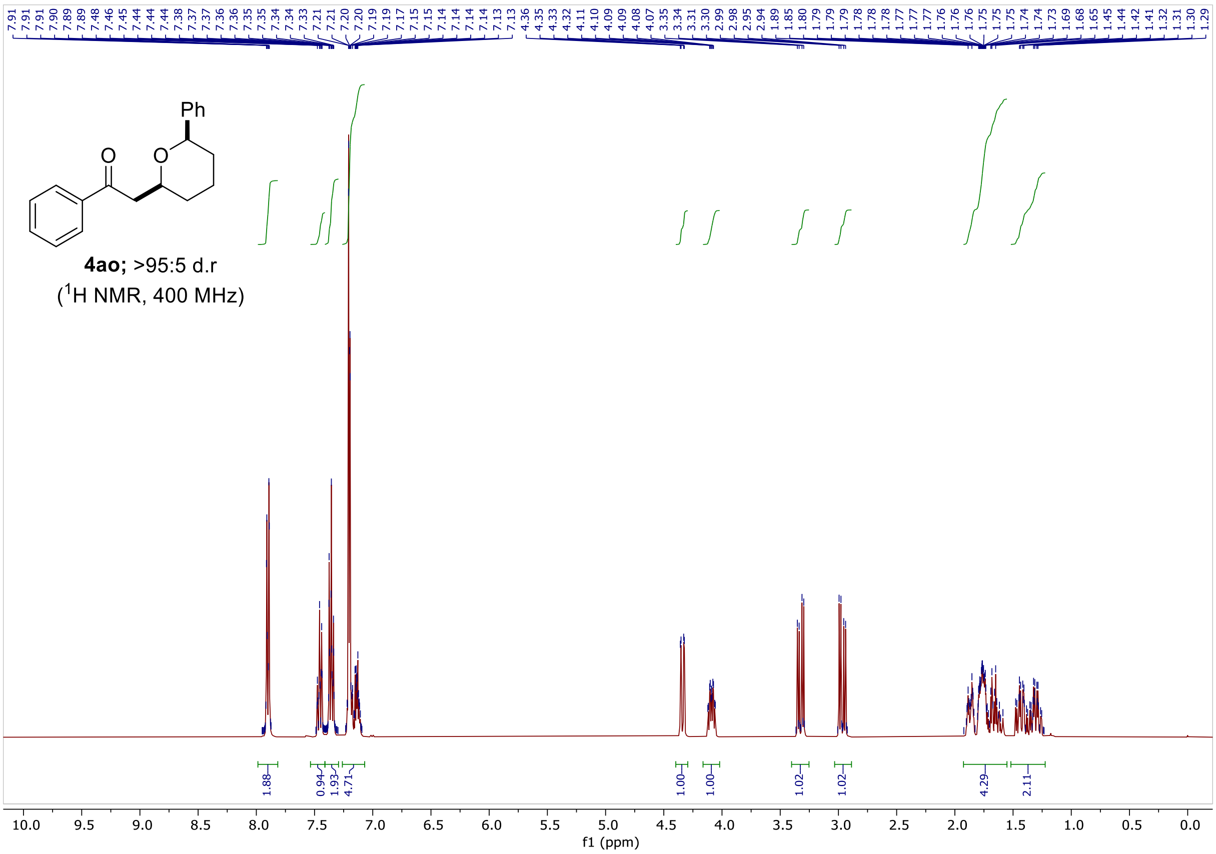
7.91 7.91 7.90 7.89 7.89 7.48 7.46 7.45 7.44 7.44 7.38 7.37 7.37 7.36 7.36 7.35 7.35 7.34 7.34 7.33 7.21 7.21 7.20 7.20 7.19 7.19 7.17 7.15 7.15 7.14 7.14 7.14 7.14 7.13 7.13 4.36 4.35 4.33 4.32 4.11 4.10 4.09 4.09 4.08 4.07 3.35 3.34 3.31 3.30 2.99 2.98 2.95 2.94 1.89 1.85 1.80 1.79 1.79 1.79 1.78 1.78 1.78 1.77 1.77 1.76 1.76 1.75 1.75 1.75 1.74 1.74 1.73 1.69 1.68 1.65 1.45 1.44 1.42 1.41 1.32 1.31 1.30 1.29
Ph
O
O
4ao; >95:5 d.r
(1H NMR, 400 MHz)
1.88
0.94
1.93
4.71
1.00
1.00
1.02
1.02
4.29
2.11
10.0 9.5 9.0 8.5 8.0 7.5 7.0 6.5 6.0 5.5 5.0 4.5 4.0 3.5 3.0 2.5 2.0 1.5 1.0 0.5 0.0
f1 (ppm)

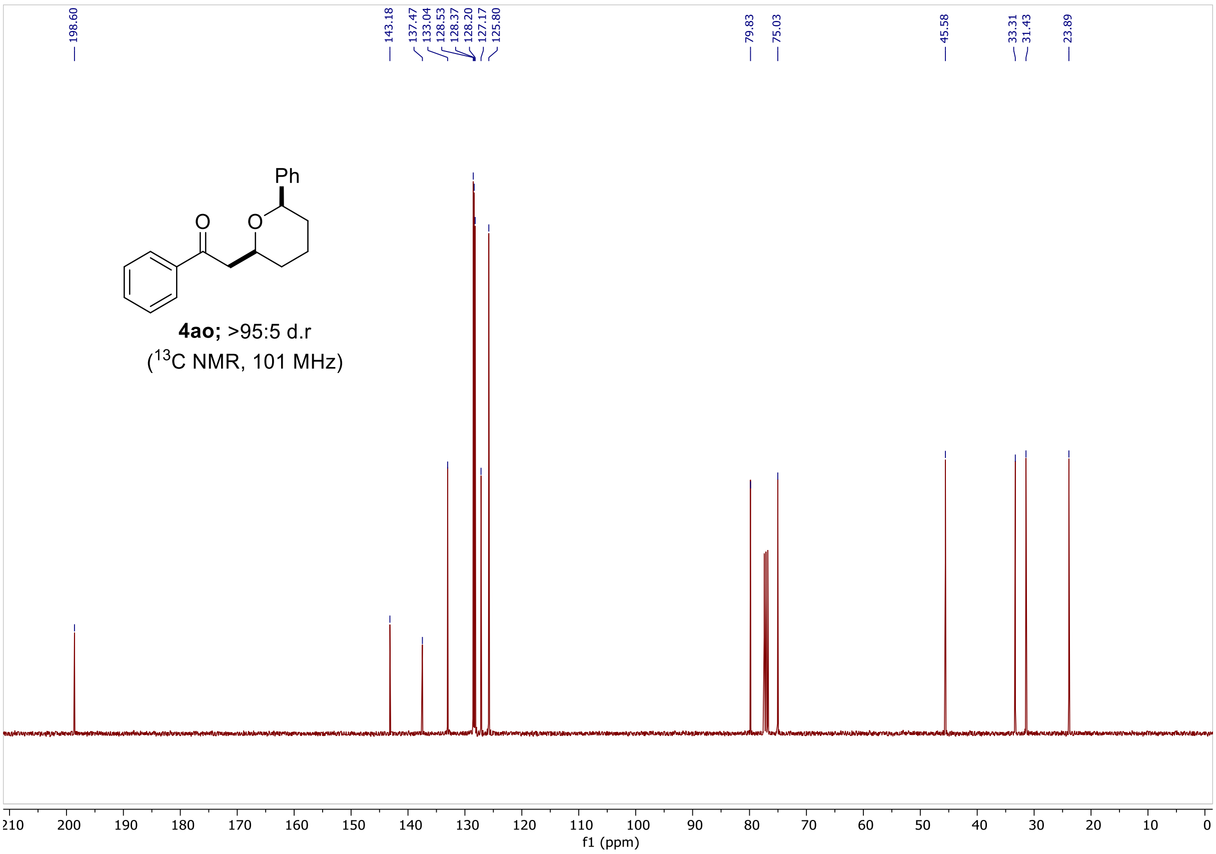
198.60
143.18
137.47
133.04
128.53
128.37
128.20
127.17
125.80
79.83
75.03
45.58
33.31
31.43
23.89
Ph
O
O
4ao; >95:5 d.r
(13C NMR, 101 MHz)
210 200 190 180 170 160 150 140 130 120 110 100 90 80 70 60 50 40 30 20 10 0
f1 (ppm)

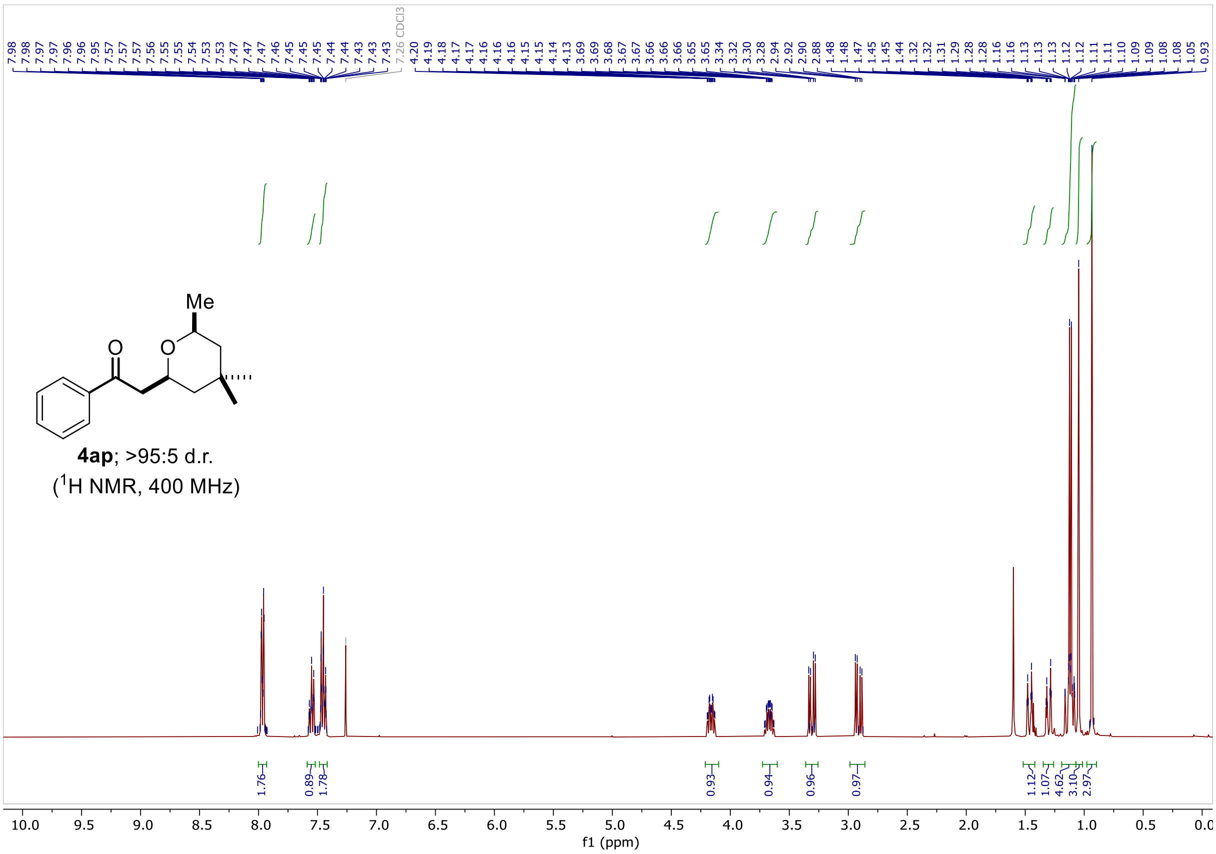
7.98
7.98
7.97
7.97
7.96
7.95
7.57
7.57
7.57
7.56
7.55
7.55
7.54
7.53
7.53
7.47
7.47
7.46
7.45
7.45
7.45
7.44
7.44
7.43
7.43
7.43
7.26 CDCl3
4.20
4.19
4.18
4.17
4.17
4.16
4.16
4.16
4.15
4.15
4.14
4.13
3.69
3.69
3.68
3.67
3.67
3.66
3.66
3.66
3.65
3.65
3.34
3.32
3.30
3.28
2.94
2.92
2.90
2.88
1.48
1.48
1.47
1.45
1.45
1.44
1.32
1.32
1.31
1.29
1.28
1.28
1.16
1.13
1.13
1.13
1.12
1.12
1.11
1.11
1.10
1.09
1.09
1.08
1.08
1.05
0.93
Me
O
O
4ap; >95:5 d.r.
(1H NMR, 400 MHz)
1.76
0.89
1.78
0.93
0.94
0.96
0.97
1.12
1.07
4.62
3.10
2.97
f1 (ppm)

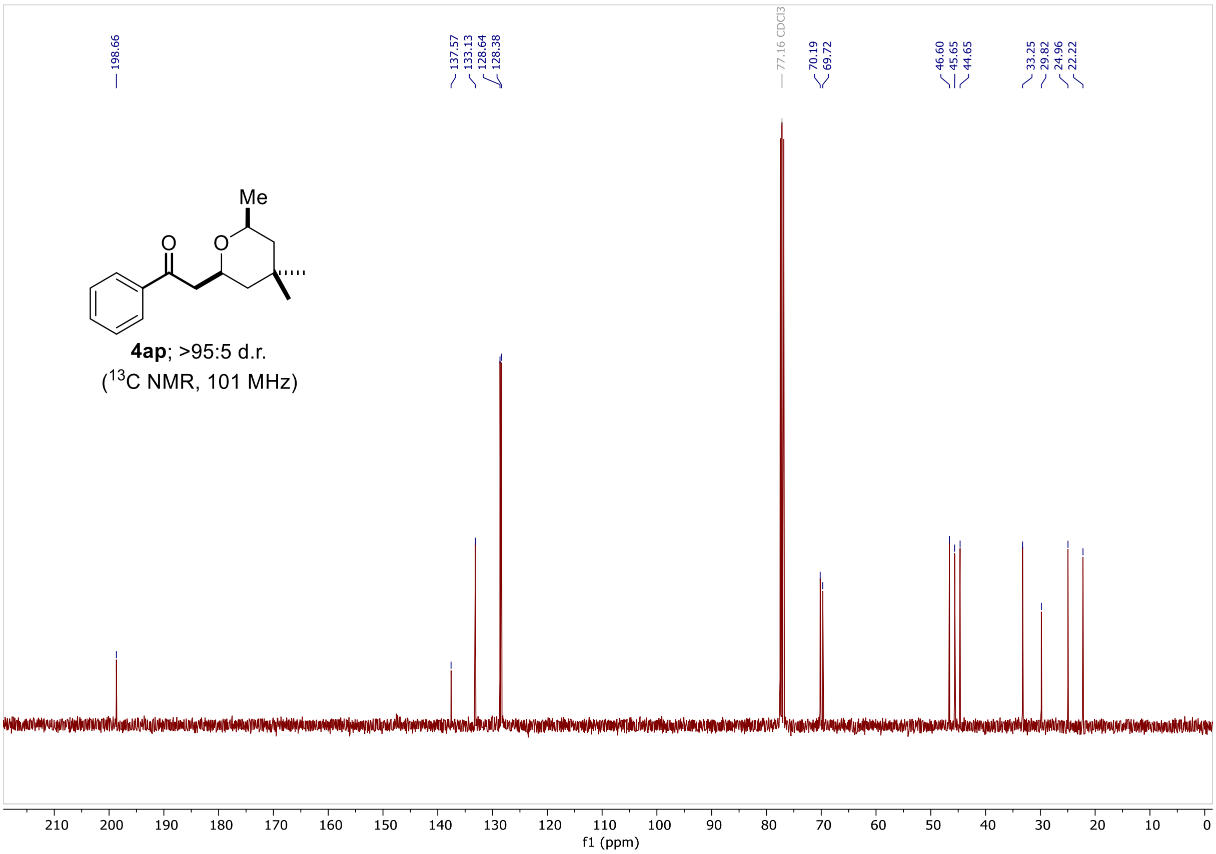
198.66
137.57
133.13
128.64
128.38
77.16 CDCl3
70.19
69.72
46.60
45.65
44.65
33.25
29.82
24.96
22.22
Me
O
O
4ap; >95:5 d.r.
(13C NMR, 101 MHz)
f1 (ppm)

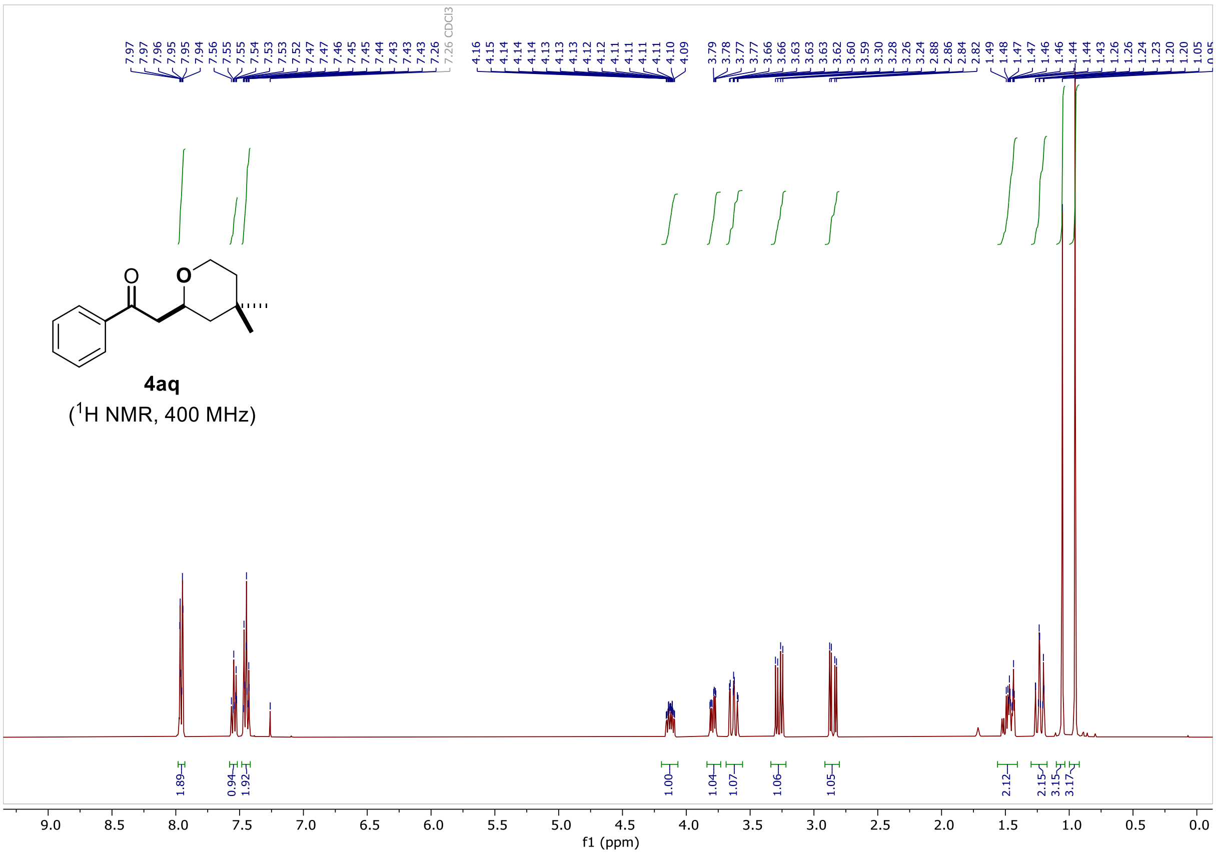
4aq
(¹H NMR, 400 MHz)
f1 (ppm)

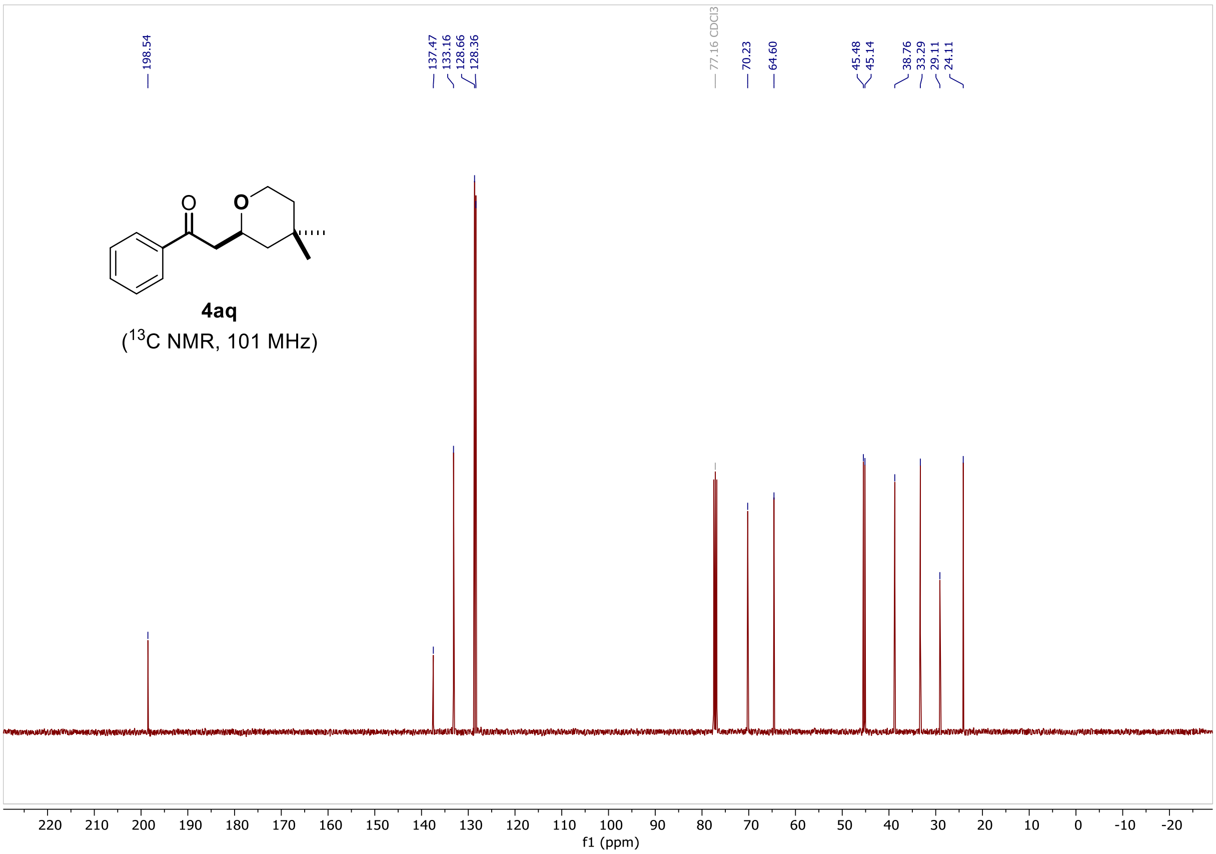
4aq
(¹³C NMR, 101 MHz)
198.54
137.47
133.16
128.66
128.36
77.16 CDCl3
70.23
64.60
45.48
45.14
38.76
33.29
29.11
24.11
f1 (ppm)

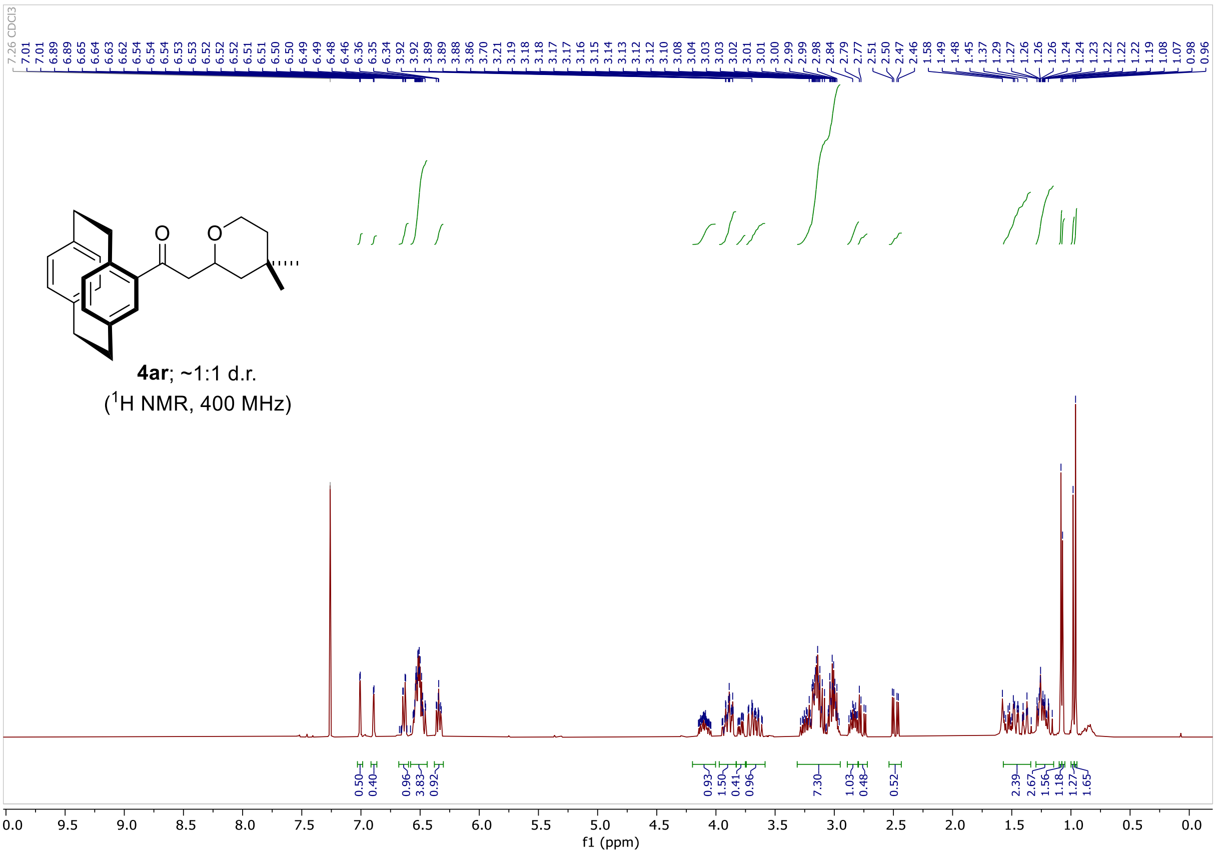

4ar; ~1:1 d.r.
(1H NMR, 400 MHz)
f1 (ppm)

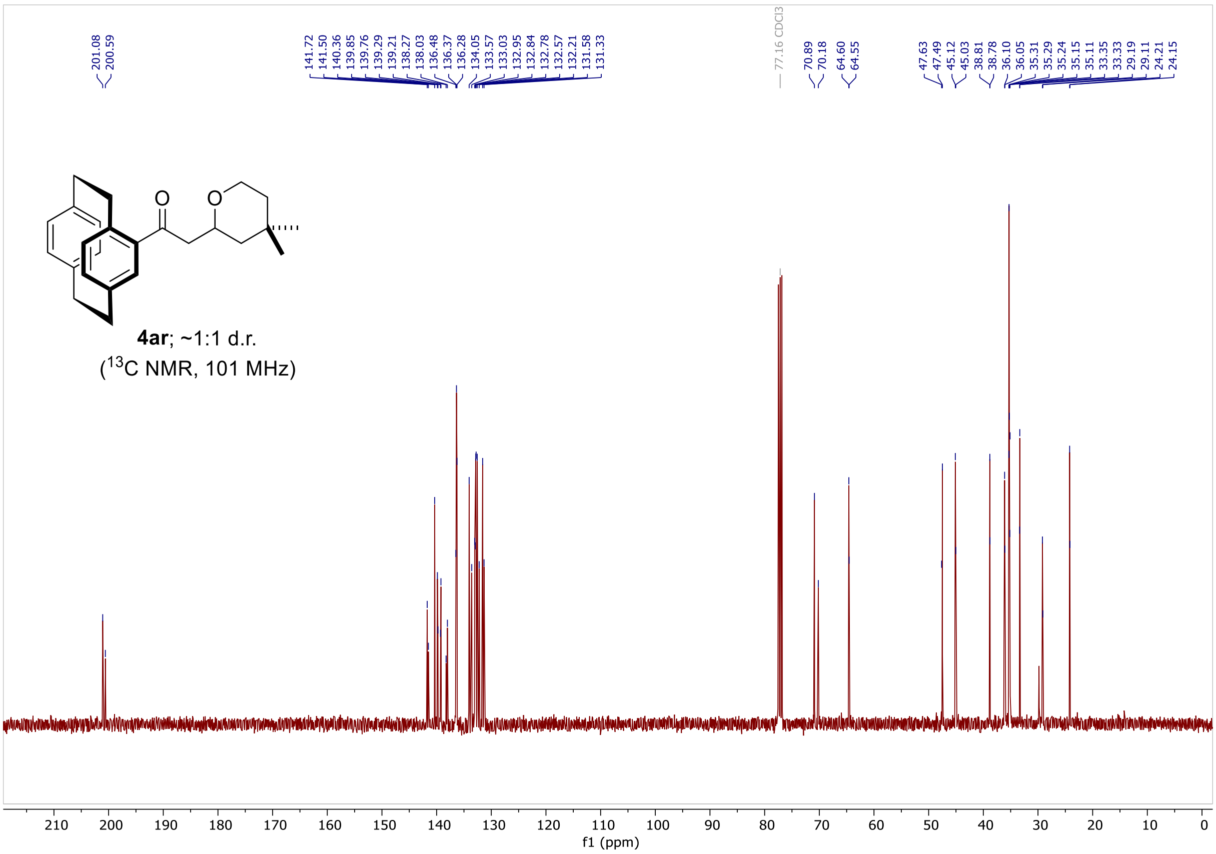

4ar; ~1:1 d.r.
(13C NMR, 101 MHz)
f1 (ppm)

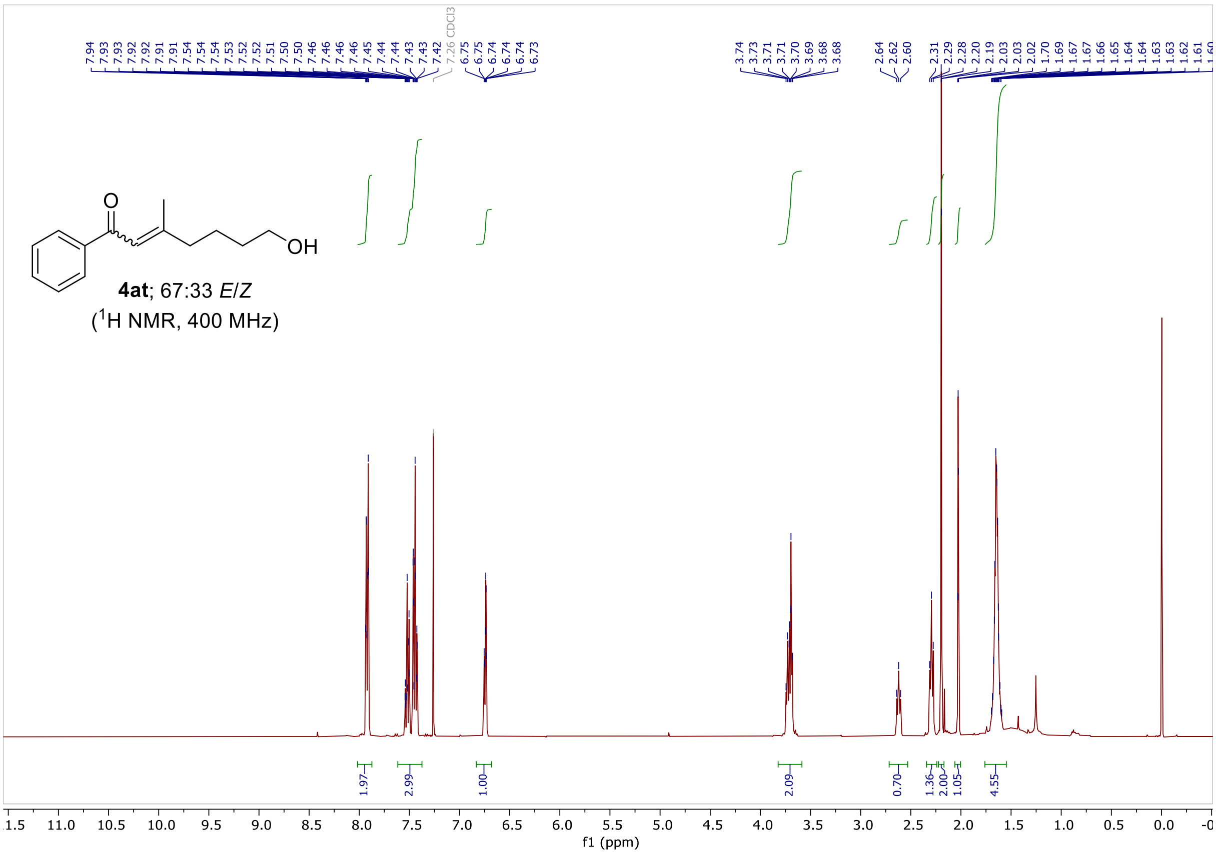

4at; 67:33 E/Z
(1H NMR, 400 MHz)
f1 (ppm)

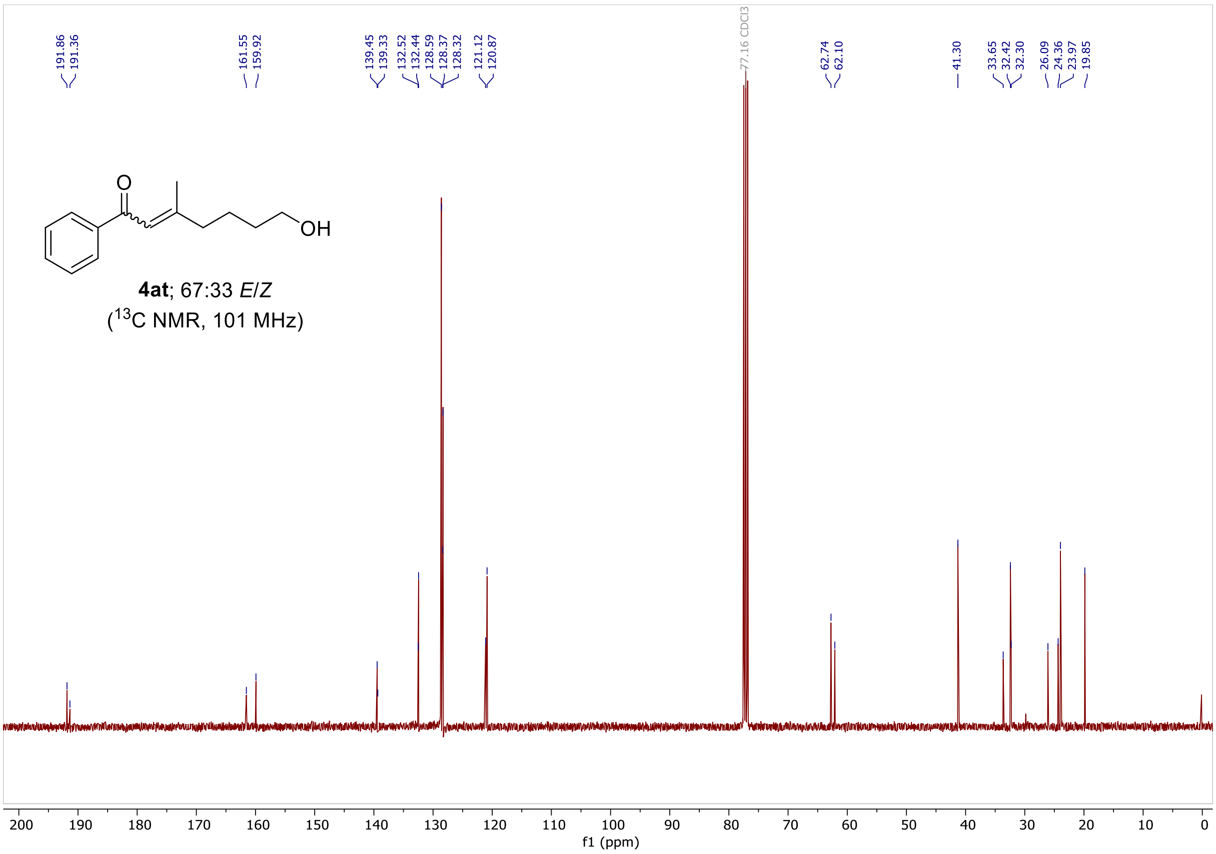

4at; 67:33 E/Z
(13C NMR, 101 MHz)
f1 (ppm)

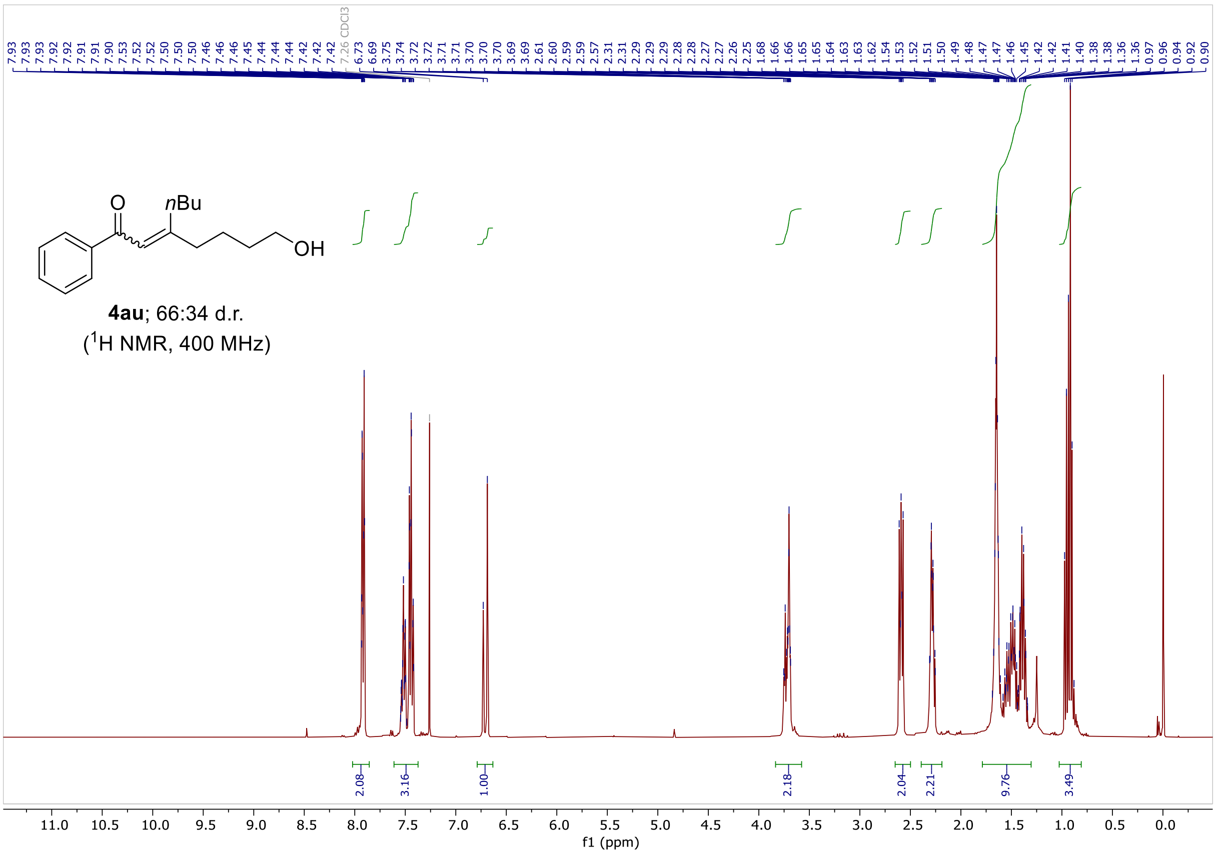

O
nBu
OH
4au; 66:34 d.r.
(1H NMR, 400 MHz)
f1 (ppm)

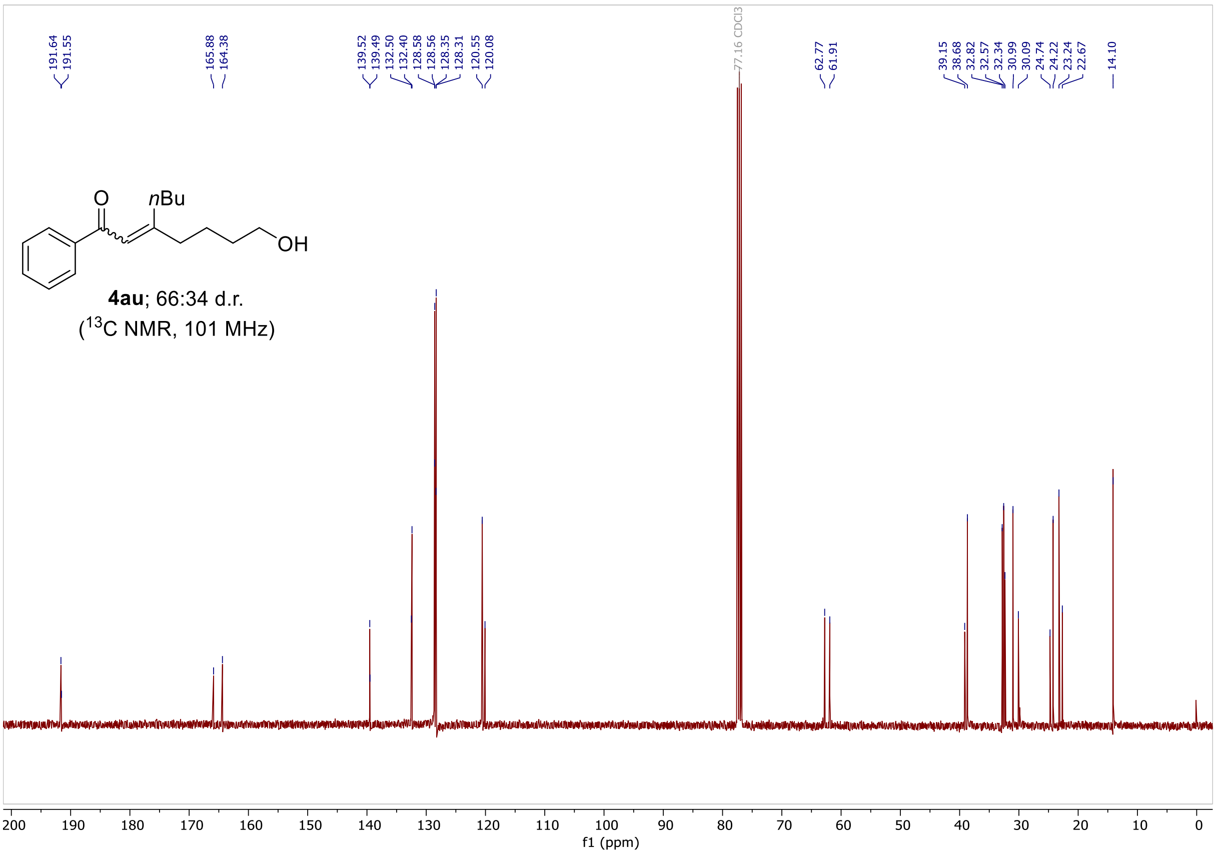

O
nBu
OH
4au; 66:34 d.r.
(13C NMR, 101 MHz)
f1 (ppm)

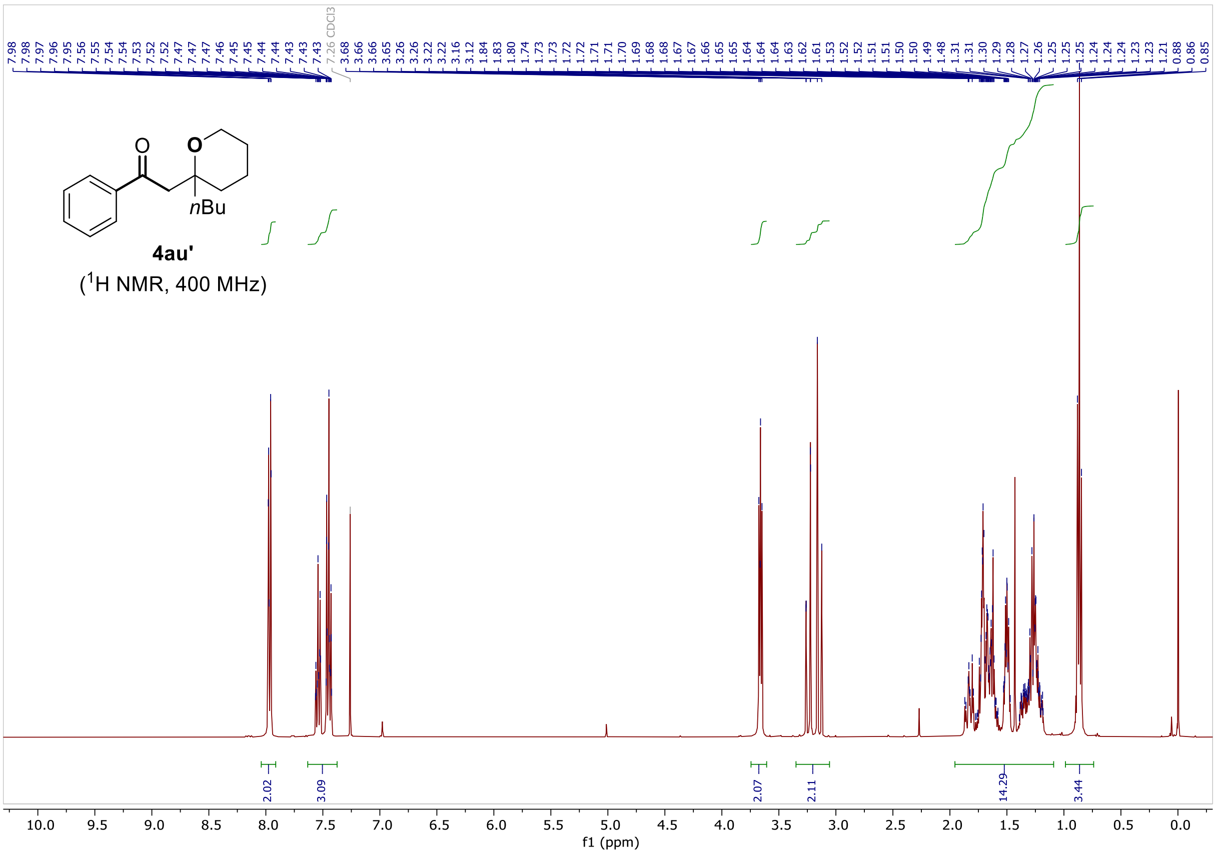
4au'
(¹H NMR, 400 MHz)
2.02
3.09
2.07
2.11
14.29
3.44
f1 (ppm)

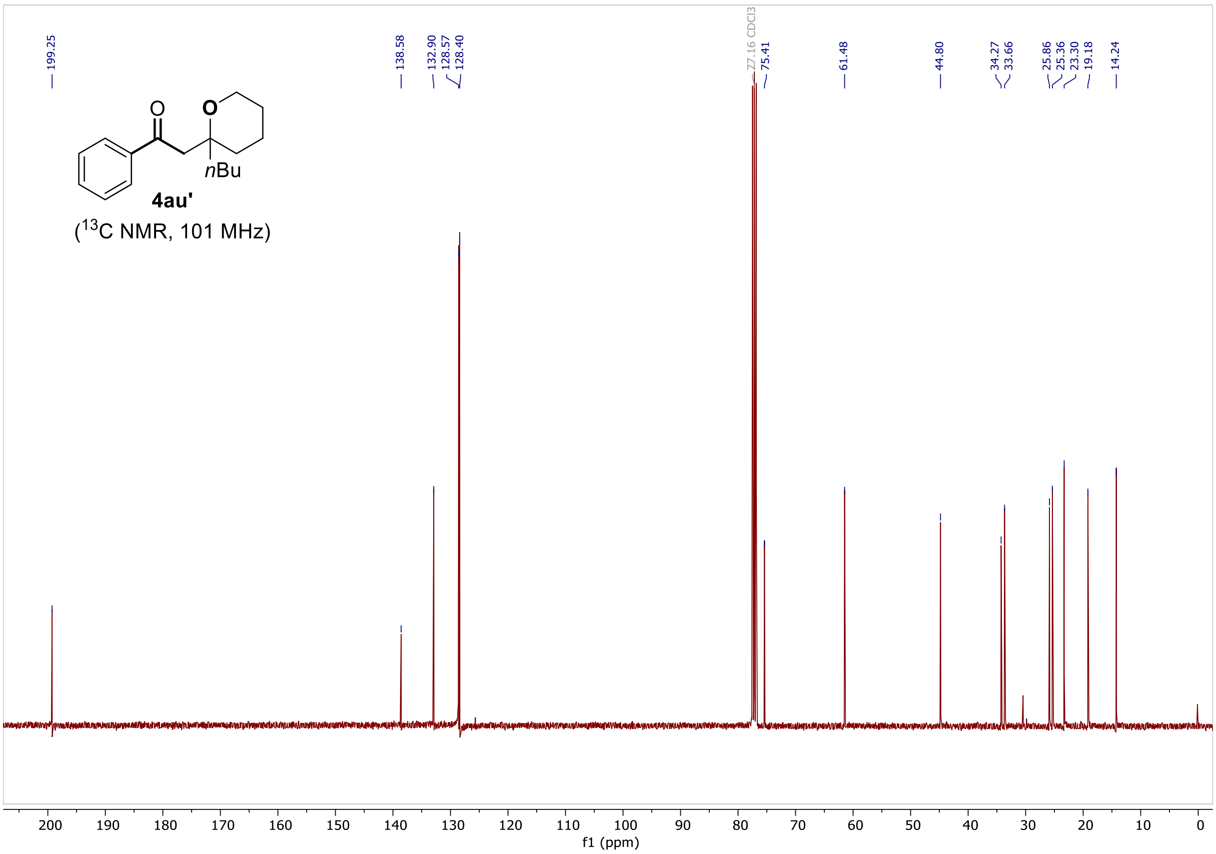
199.25
138.58
132.90
128.57
128.40
77.16 CDCl3
75.41
61.48
44.80
34.27
33.66
25.86
25.36
23.30
19.18
14.24
4au'
(¹³C NMR, 101 MHz)
f1 (ppm)

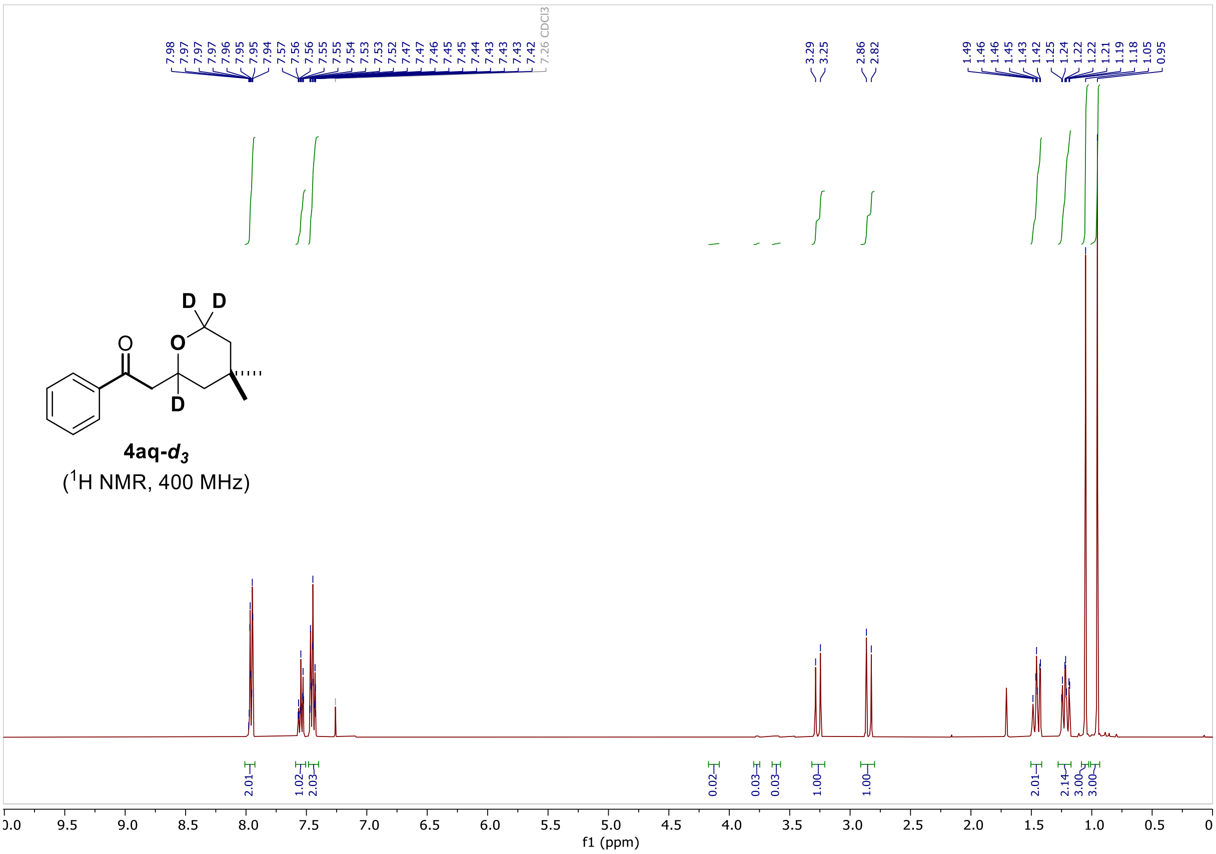
7.98
7.97
7.97
7.97
7.96
7.95
7.95
7.94
7.57
7.56
7.56
7.55
7.55
7.54
7.53
7.53
7.52
7.47
7.47
7.46
7.45
7.45
7.44
7.43
7.43
7.43
7.42
7.26 CDCl3
3.29
3.25
2.86
2.82
1.49
1.46
1.46
1.45
1.43
1.42
1.25
1.24
1.22
1.22
1.21
1.19
1.18
1.05
0.95
D
D
O
O
D
4aq-d3
(1H NMR, 400 MHz)
2.01
1.02
2.03
0.02
0.03
0.03
1.00
1.00
2.01
2.14
3.00
3.00
f1 (ppm)

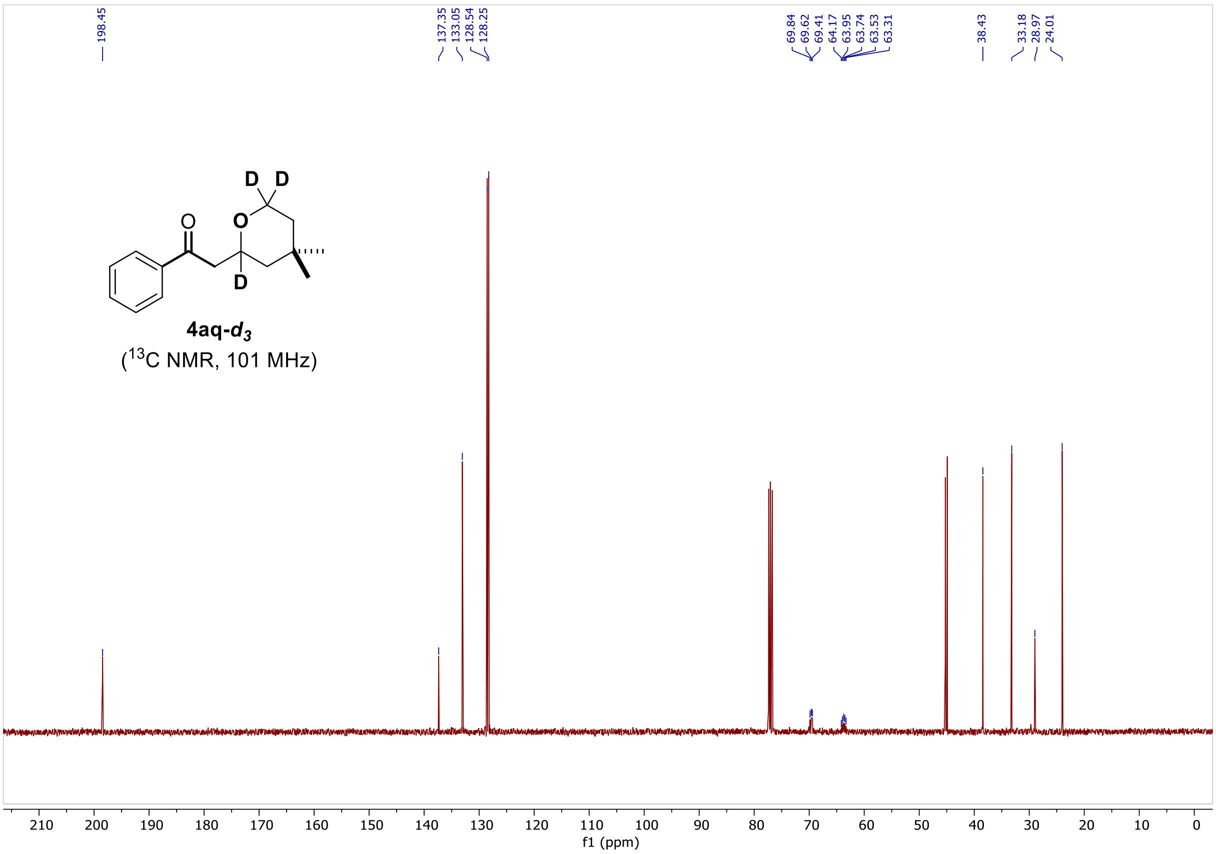
198.45
137.35
133.05
128.54
128.25
69.84
69.62
69.41
64.17
63.95
63.74
63.53
63.31
38.43
33.18
28.97
24.01
D
D
O
O
D
4aq-d3
(13C NMR, 101 MHz)
f1 (ppm)

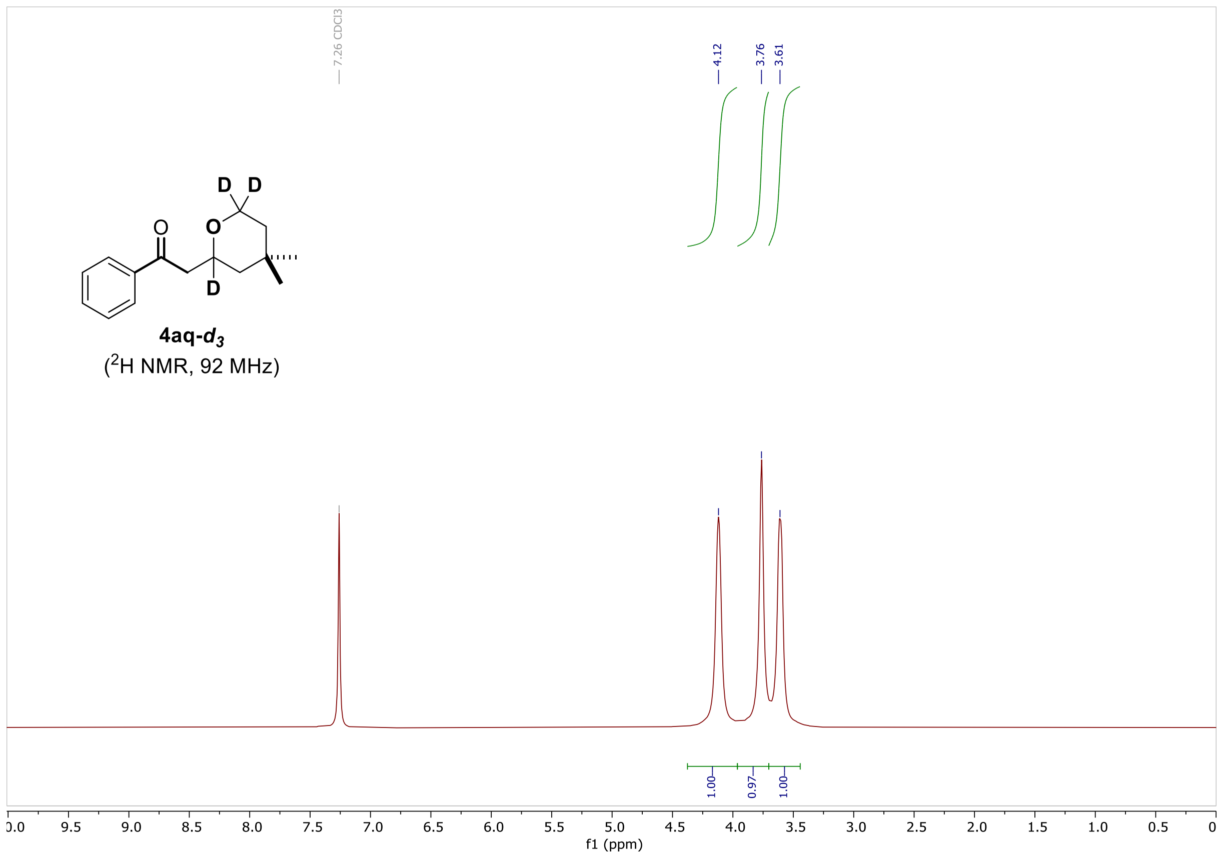

7.26 CDCl3
4.12
3.76
3.61
D D
O
O
D
4aq-d3
(2H NMR, 92 MHz)
1.00
0.97
1.00
f1 (ppm)

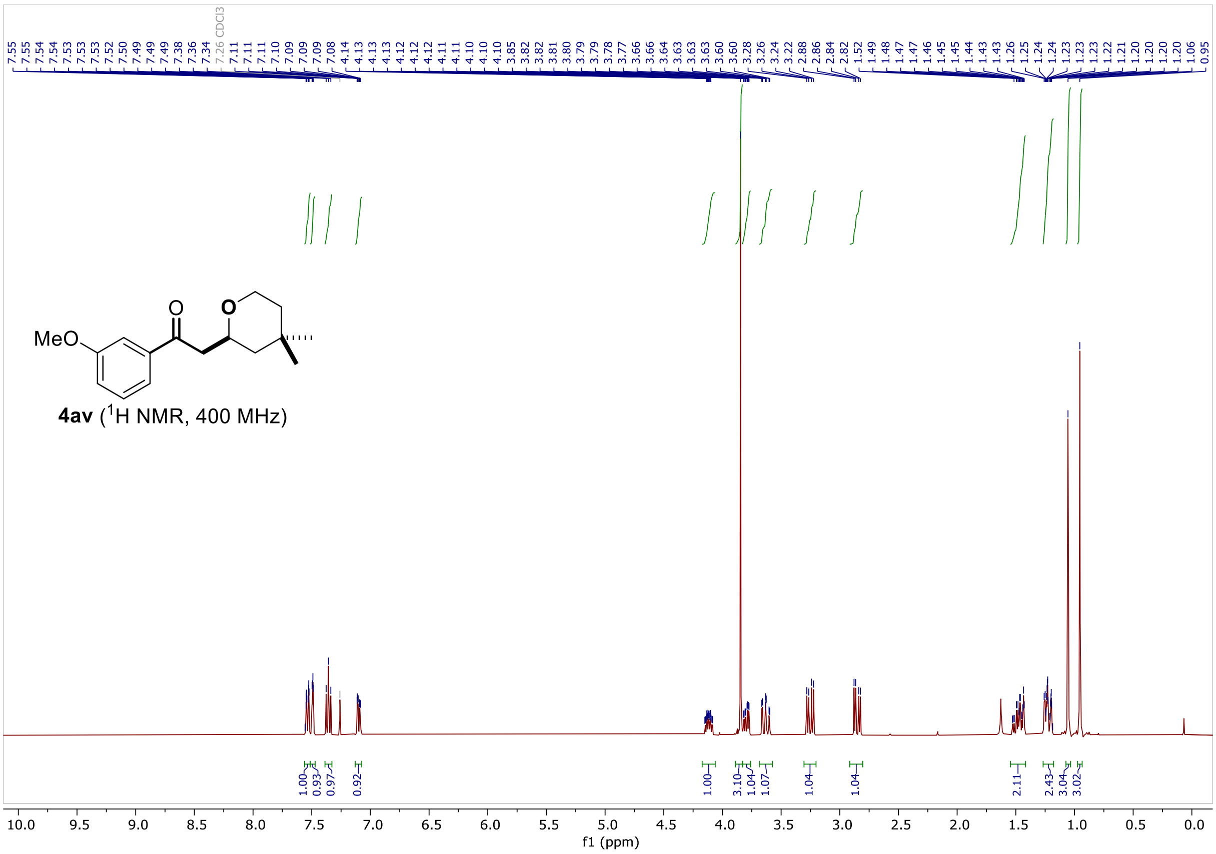
7.55 7.55 7.54 7.54 7.53 7.53 7.53 7.52 7.50 7.49 7.49 7.49 7.38 7.36 7.34 7.26 CDCl3 7.11 7.11 7.11 7.11 7.10 7.09 7.09 7.09 7.08 4.14 4.13 4.13 4.13 4.12 4.12 4.12 4.11 4.11 4.10 4.10 3.85 3.82 3.82 3.81 3.80 3.79 3.79 3.78 3.77 3.66 3.66 3.64 3.63 3.63 3.63 3.60 3.60 3.28 3.26 3.24 3.22 2.88 2.86 2.84 2.82 1.52 1.49 1.48 1.47 1.46 1.45 1.45 1.44 1.43 1.43 1.26 1.25 1.24 1.24 1.23 1.23 1.23 1.22 1.21 1.20 1.20 1.20 1.20 1.06 0.95
MeO
O
O
4av (1H NMR, 400 MHz)
1.00 0.93 0.97 0.92 1.00 3.10 1.04 1.07 1.04 1.04 2.11 2.43 3.04 3.02
10.0 9.5 9.0 8.5 8.0 7.5 7.0 6.5 6.0 5.5 5.0 4.5 4.0 3.5 3.0 2.5 2.0 1.5 1.0 0.5 0.0
f1 (ppm)

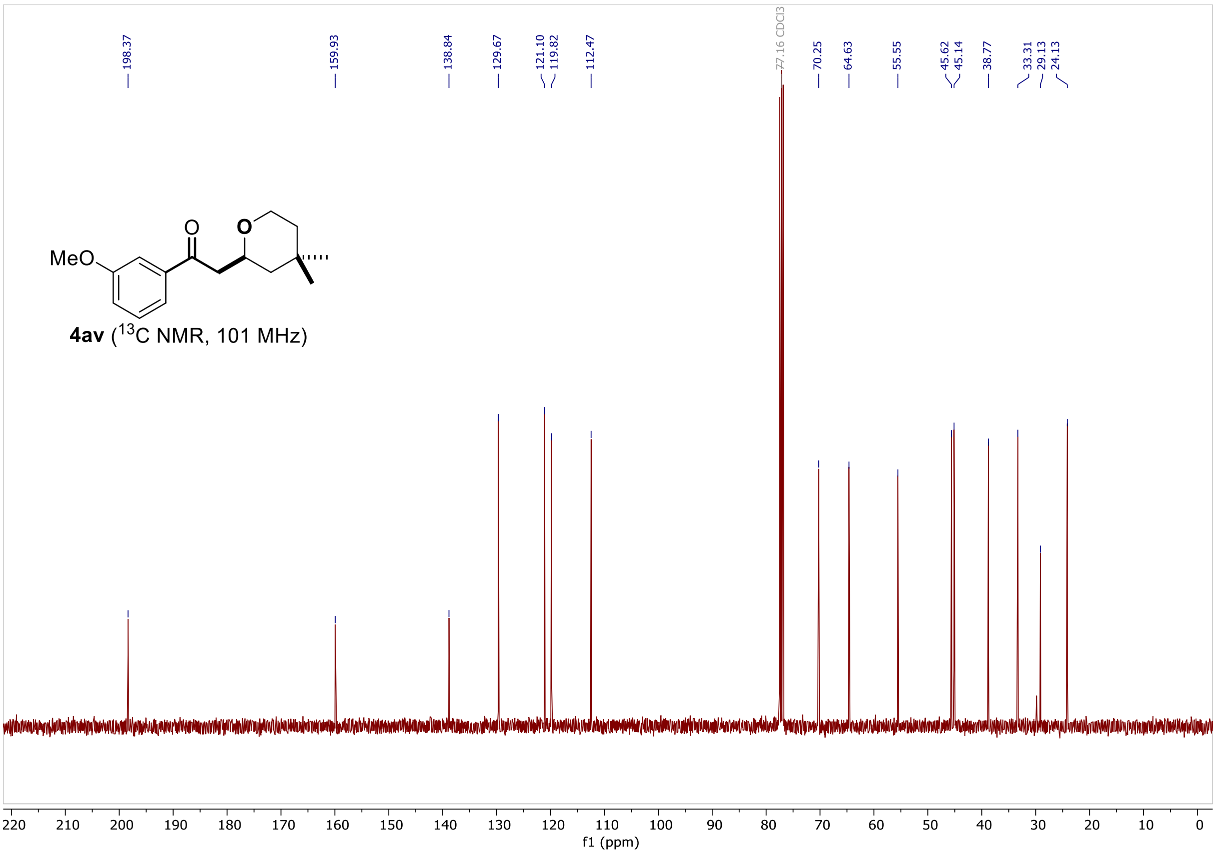
198.37 159.93 138.84 129.67 121.10 119.82 112.47 77.16 CDCl3 70.25 64.63 55.55 45.62 45.14 38.77 33.31 29.13 24.13
MeO
O
O
4av (13C NMR, 101 MHz)
220 210 200 190 180 170 160 150 140 130 120 110 100 90 80 70 60 50 40 30 20 10 0
f1 (ppm)

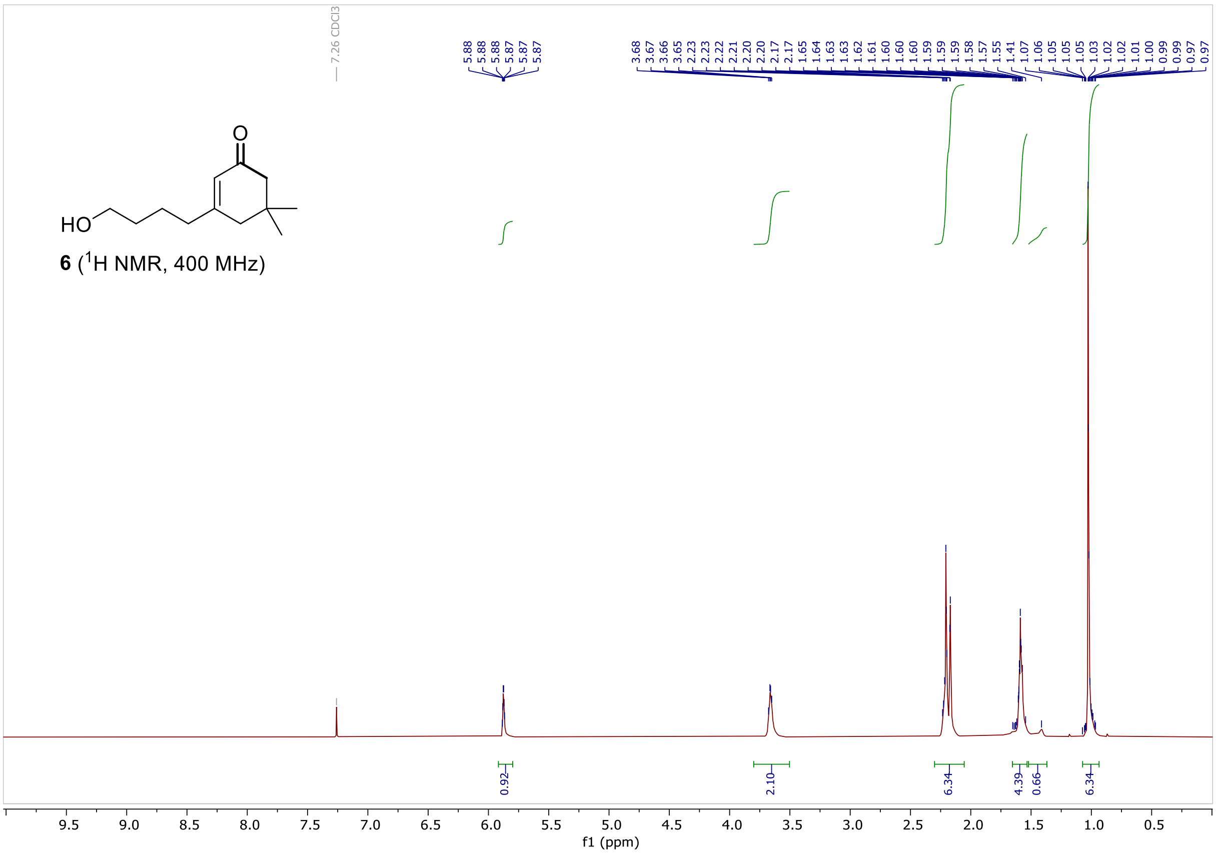

HO
O
6 (^{1}H NMR, 400 MHz)
f1 (ppm)

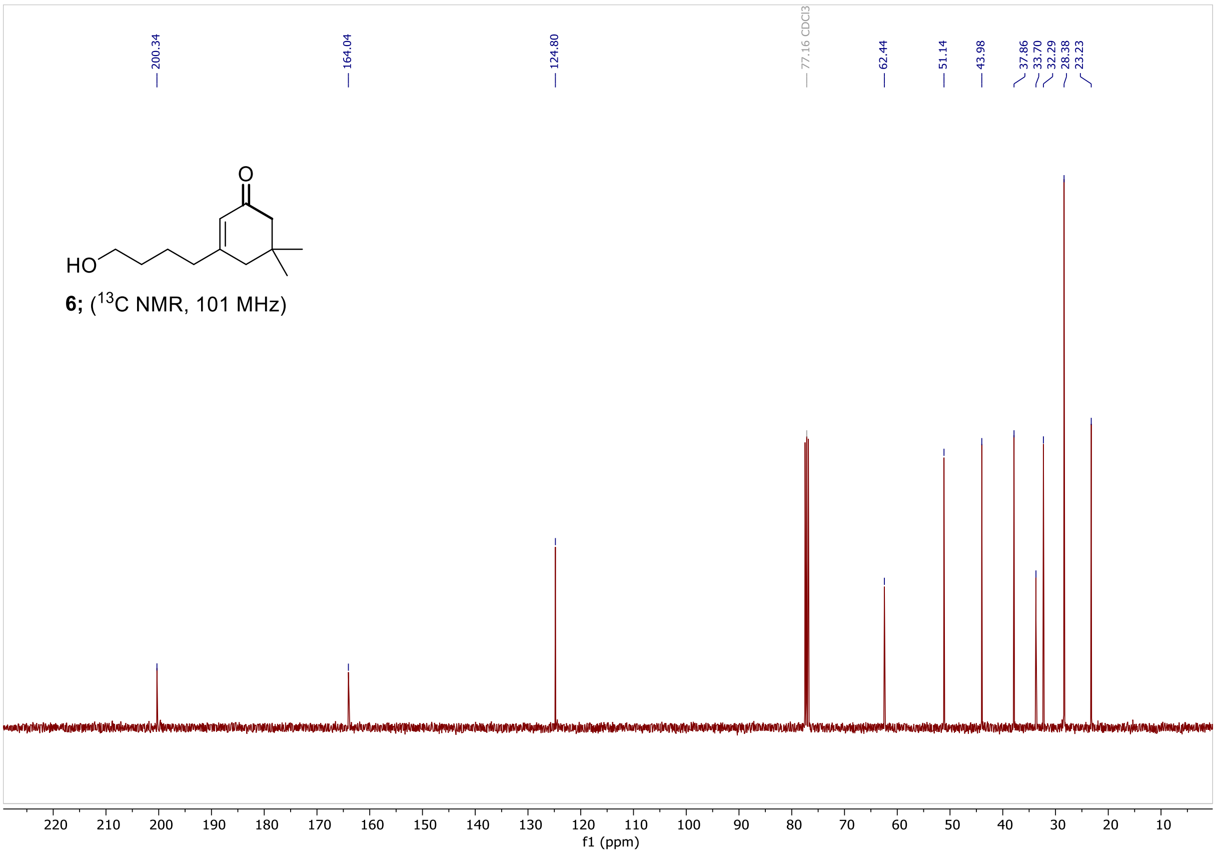

HO
O
6; (^{13}C NMR, 101 MHz)
200.34
164.04
124.80
77.16 CDCl3
62.44
51.14
43.98
37.86
33.70
32.29
28.38
23.23
f1 (ppm)

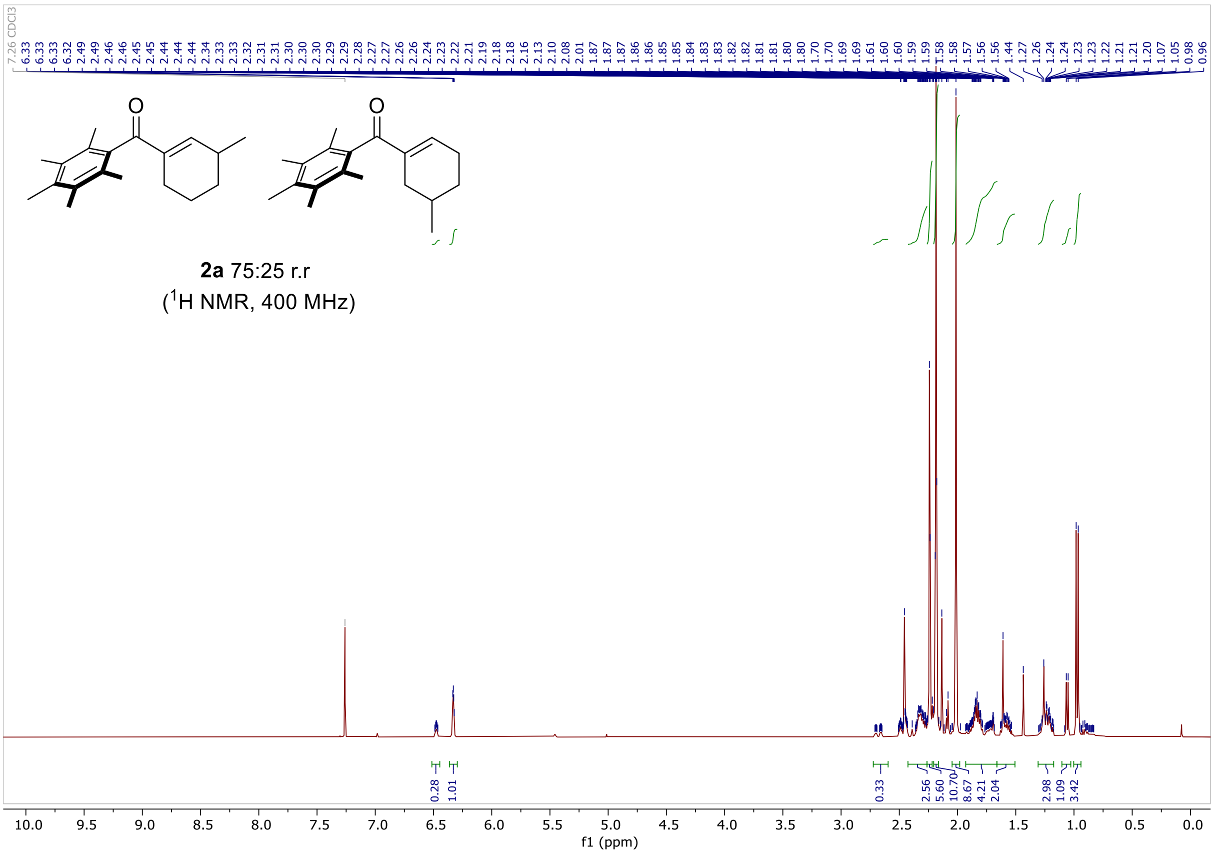

2a 75:25 r.r
(1H NMR, 400 MHz)
f1 (ppm)

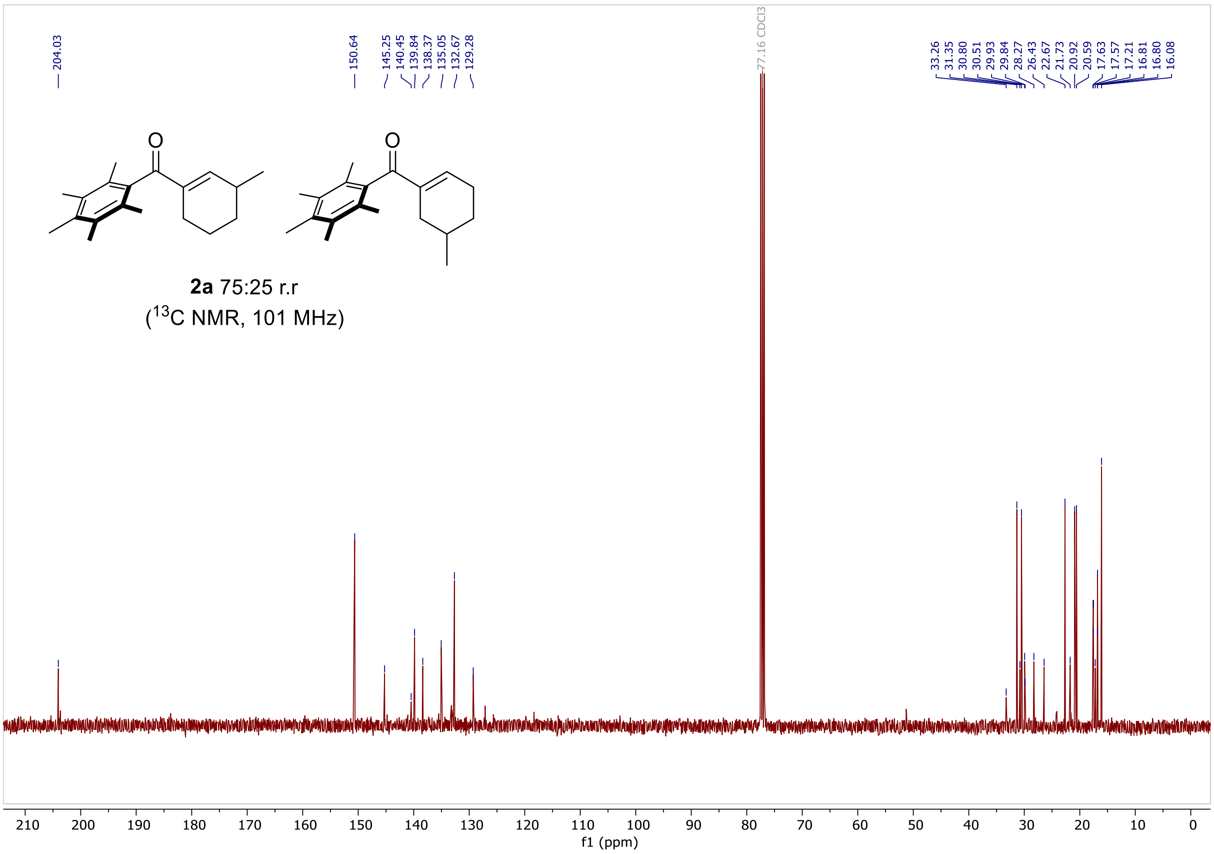

2a 75:25 r.r
(13C NMR, 101 MHz)
f1 (ppm)

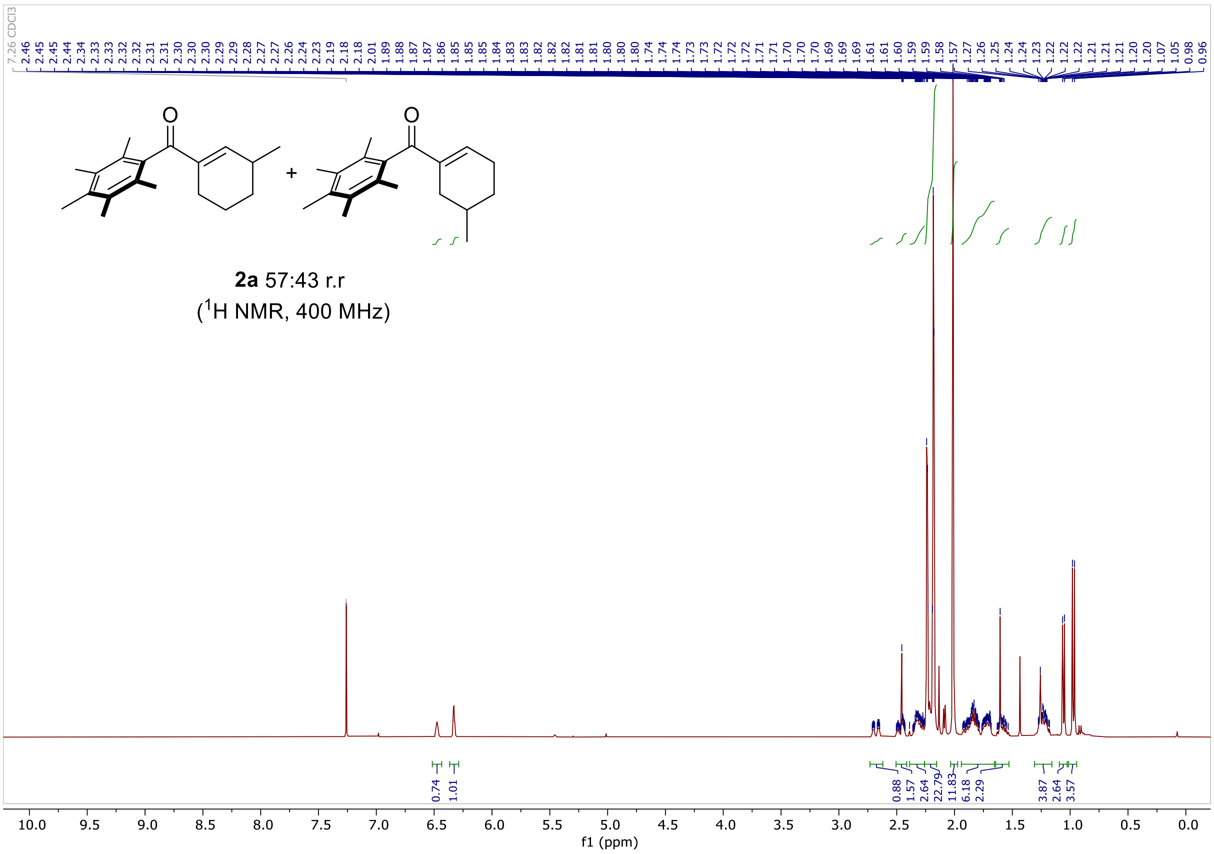

2a 57:43 r.r
(^{1}H NMR, 400 MHz)

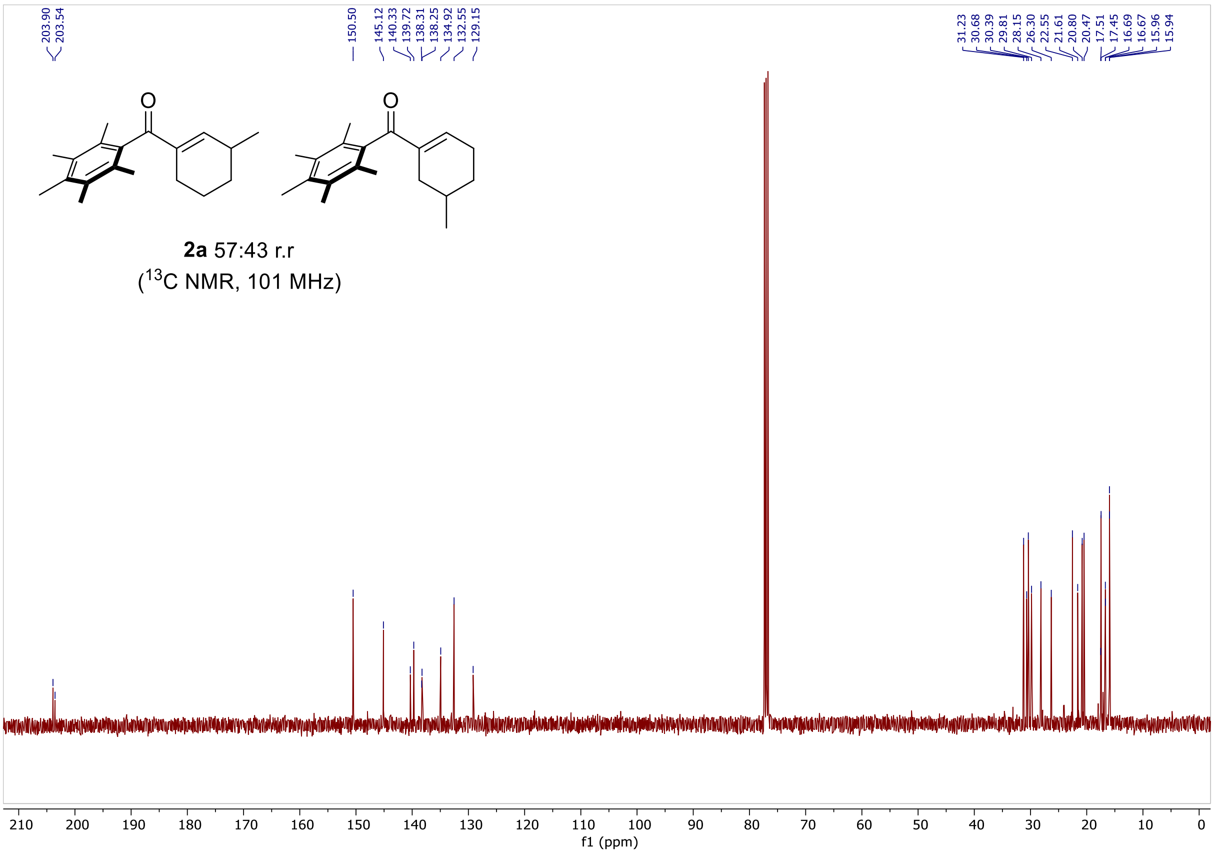

2a 57:43 r.r
(^{13}C NMR, 101 MHz)

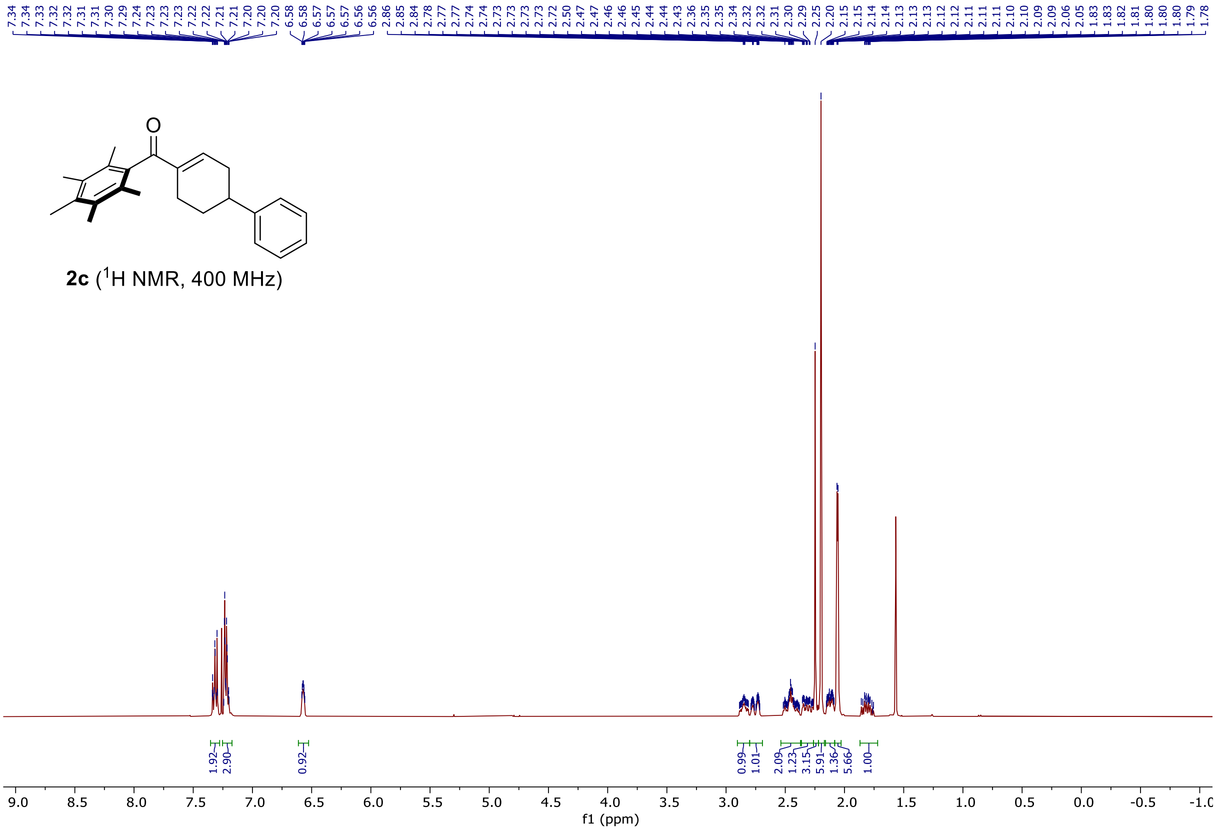

2c (^{1}H NMR, 400 MHz)

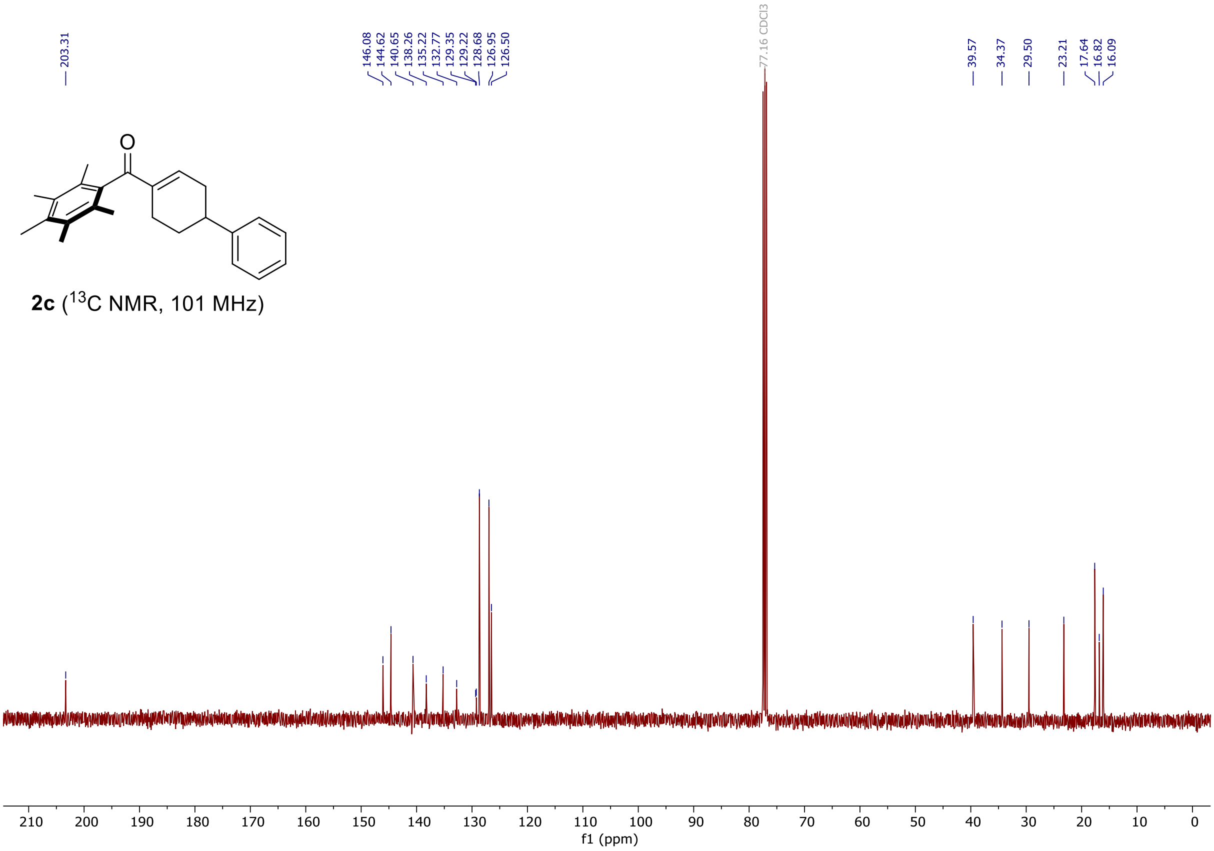

2c (^{13}C NMR, 101 MHz)

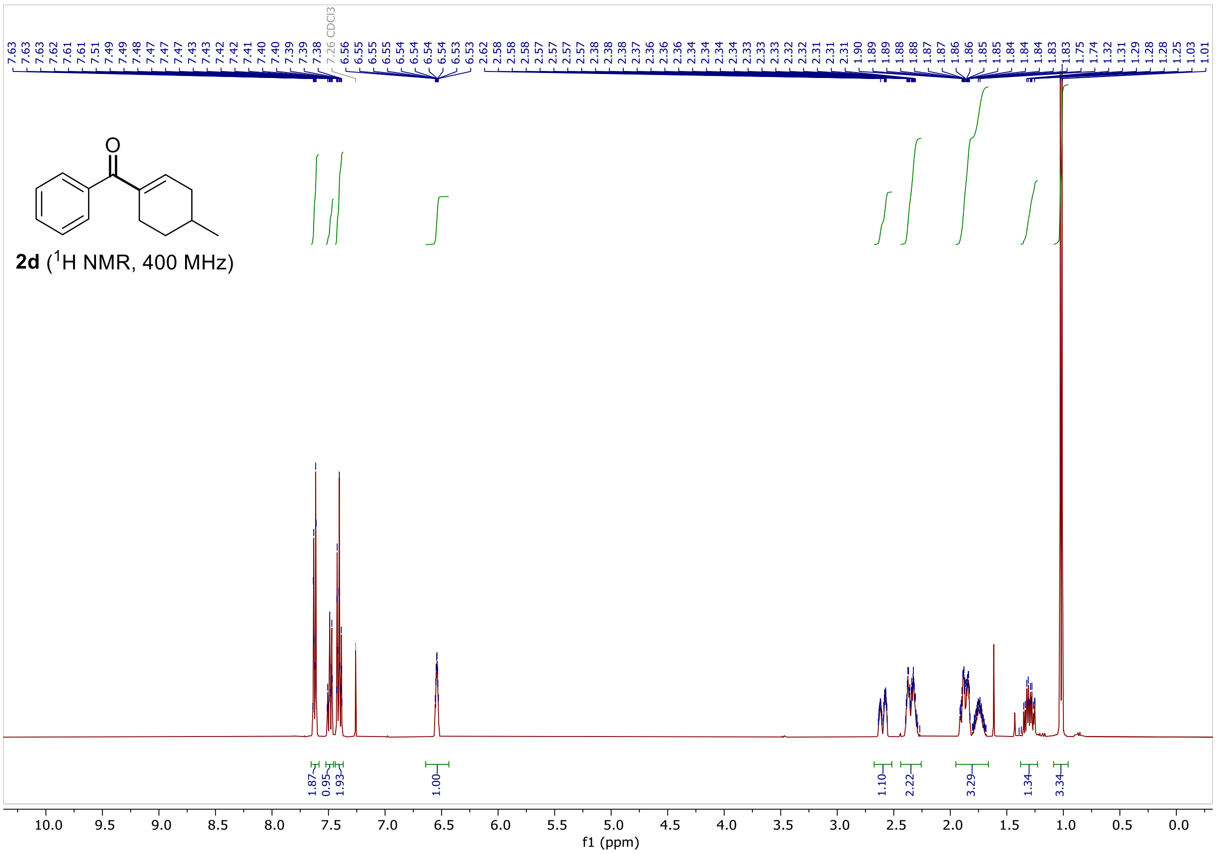
7.63 7.63 7.62 7.61 7.61 7.51 7.49 7.49 7.48 7.47 7.47 7.47 7.43 7.43 7.42 7.42 7.41 7.40 7.40 7.39 7.39 7.38 7.26 CDCl3 6.56 6.55 6.55 6.55 6.54 6.54 6.54 6.54 6.53 6.53 2.62 2.58 2.58 2.58 2.57 2.57 2.57 2.38 2.38 2.38 2.37 2.36 2.36 2.36 2.34 2.34 2.34 2.33 2.33 2.33 2.32 2.32 2.31 2.31 1.90 1.89 1.89 1.88 1.88 1.87 1.87 1.86 1.86 1.85 1.85 1.84 1.84 1.84 1.83 1.83 1.75 1.74 1.32 1.31 1.29 1.28 1.28 1.25 1.03 1.01
2d (1H NMR, 400 MHz)
1.87 0.95 1.93 1.00 1.10 2.22 3.29 1.34 3.34
10.0 9.5 9.0 8.5 8.0 7.5 7.0 6.5 6.0 5.5 5.0 4.5 4.0 3.5 3.0 2.5 2.0 1.5 1.0 0.5 0.0
f1 (ppm)

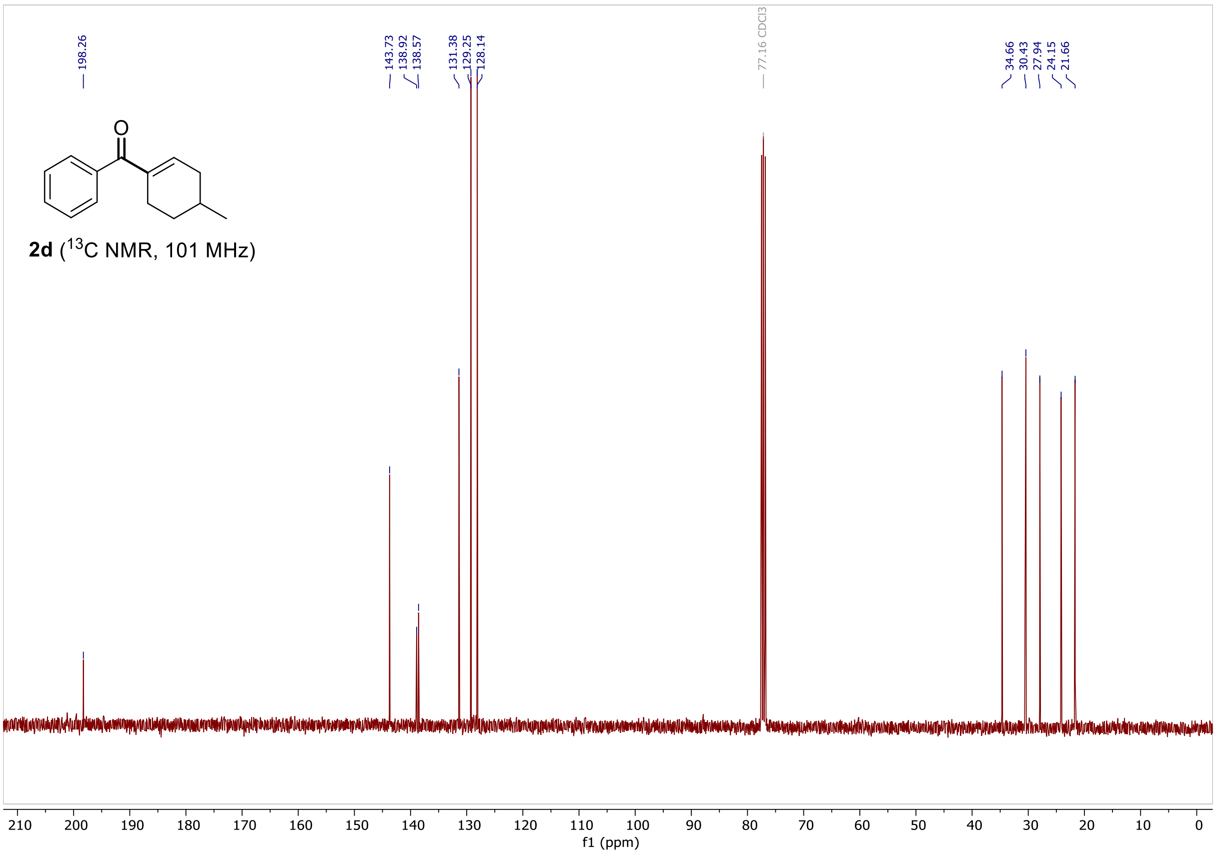
198.26
143.73 138.92 138.57 131.38 129.25 128.14
77.16 CDCl3
34.66 30.43 27.94 24.15 21.66
2d (13C NMR, 101 MHz)
210 200 190 180 170 160 150 140 130 120 110 100 90 80 70 60 50 40 30 20 10 0
f1 (ppm)

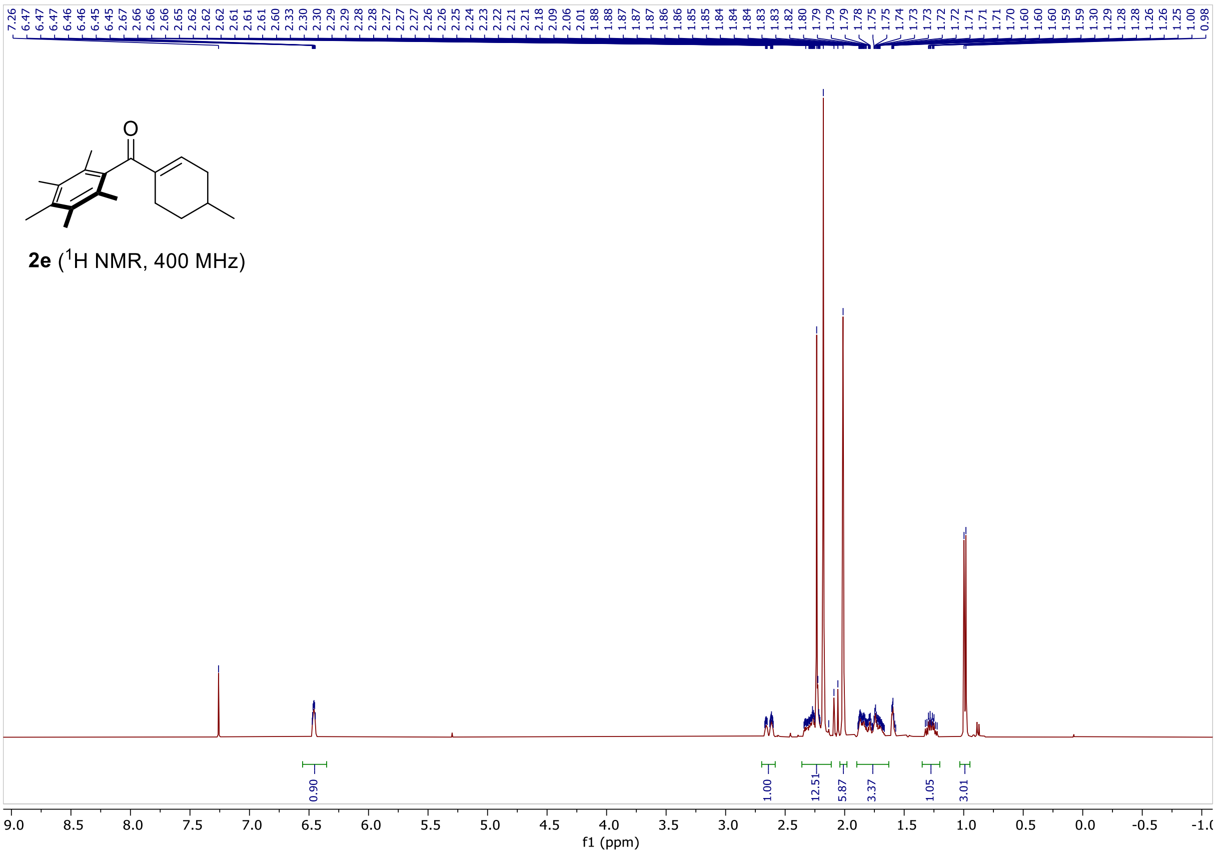

2e (¹H NMR, 400 MHz)

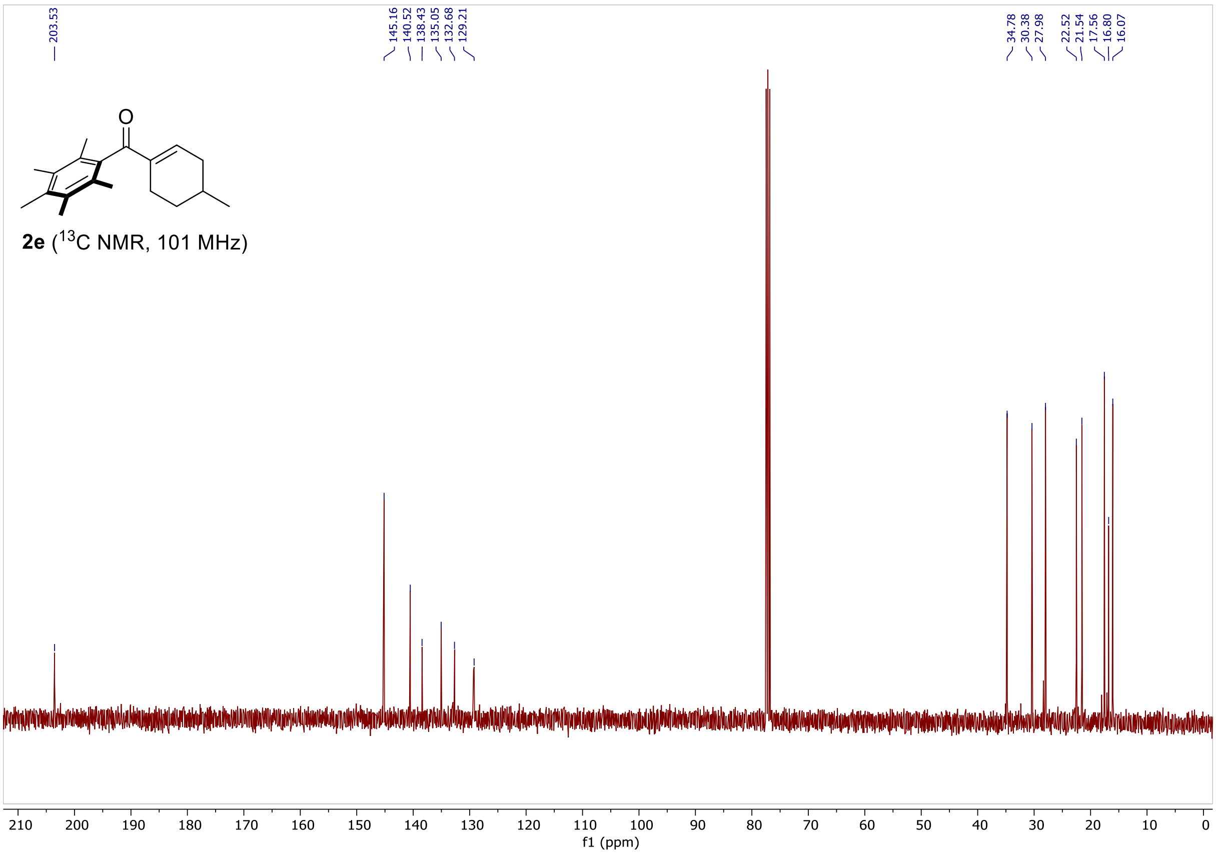

2e (¹³C NMR, 101 MHz)

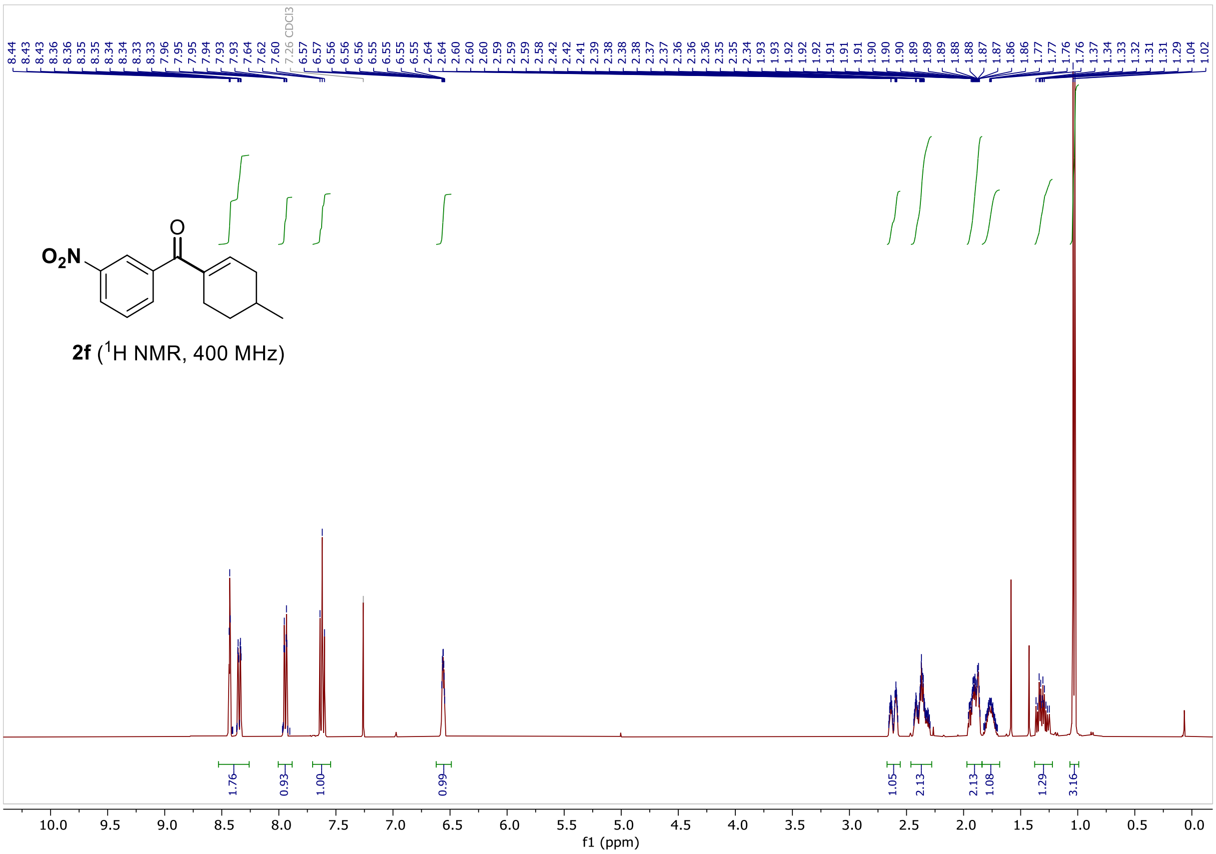

2f (¹H NMR, 400 MHz)
f1 (ppm)

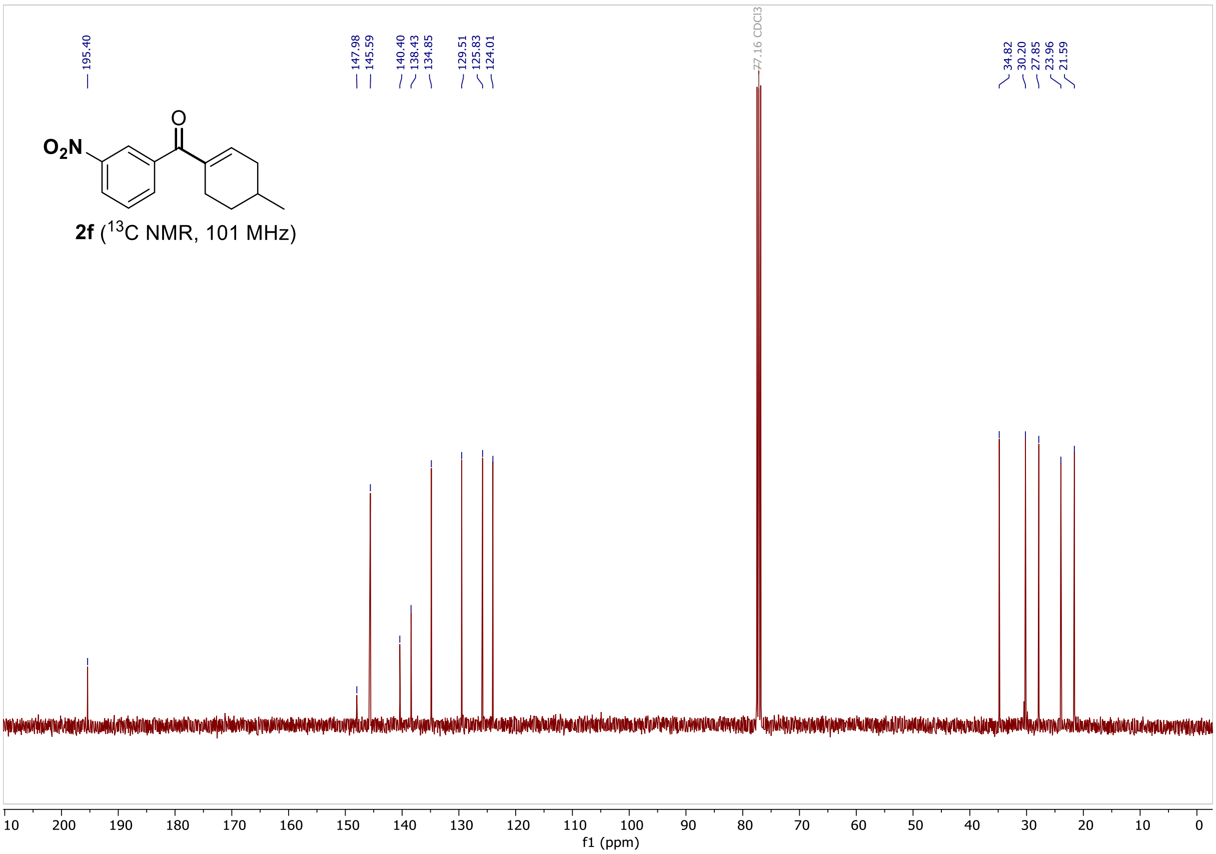

2f (¹³C NMR, 101 MHz)
195.40
147.98
145.59
140.40
138.43
134.85
129.51
125.83
124.01
77.16 CDCl3
34.82
30.20
27.85
23.96
21.59
f1 (ppm)

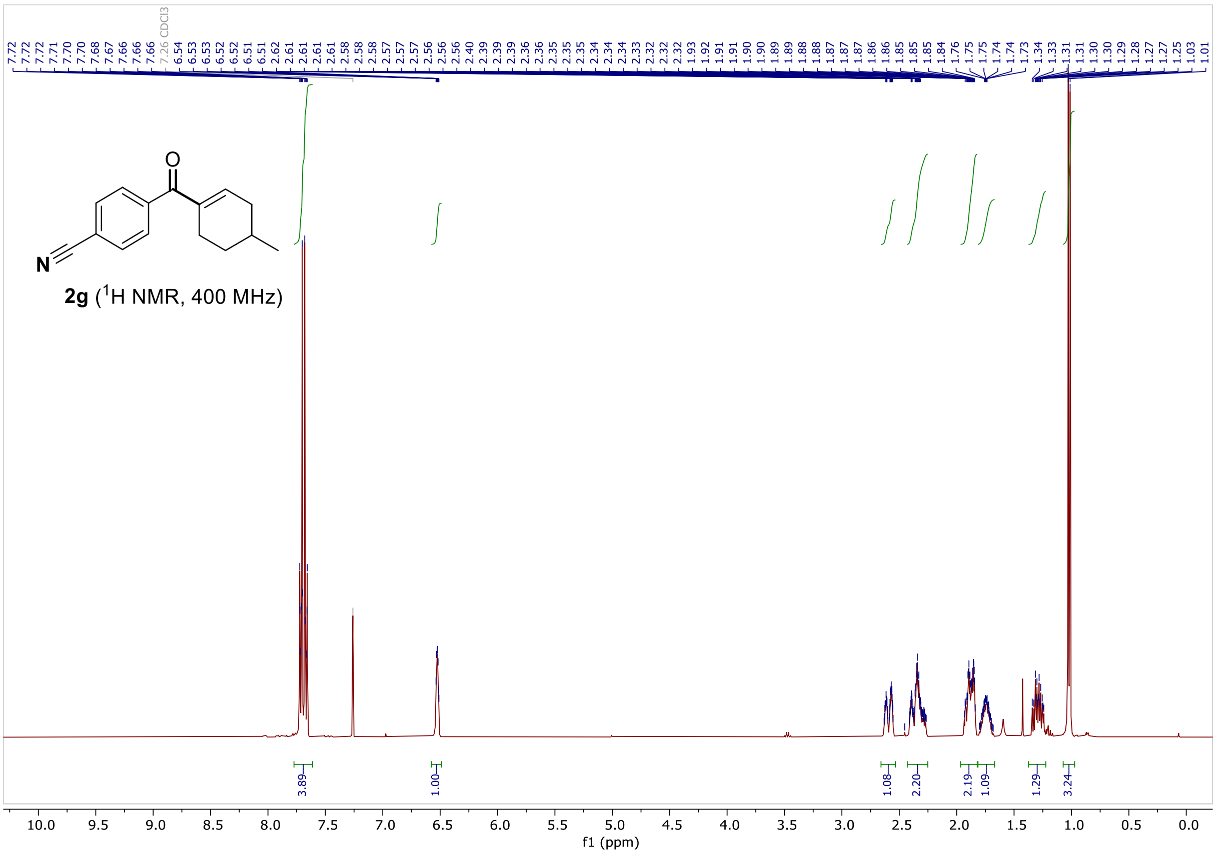
2g (1H NMR, 400 MHz)
f1 (ppm)
7.26 CDCl3
3.89
1.00
1.08
2.20
2.19
1.09
1.29
3.24

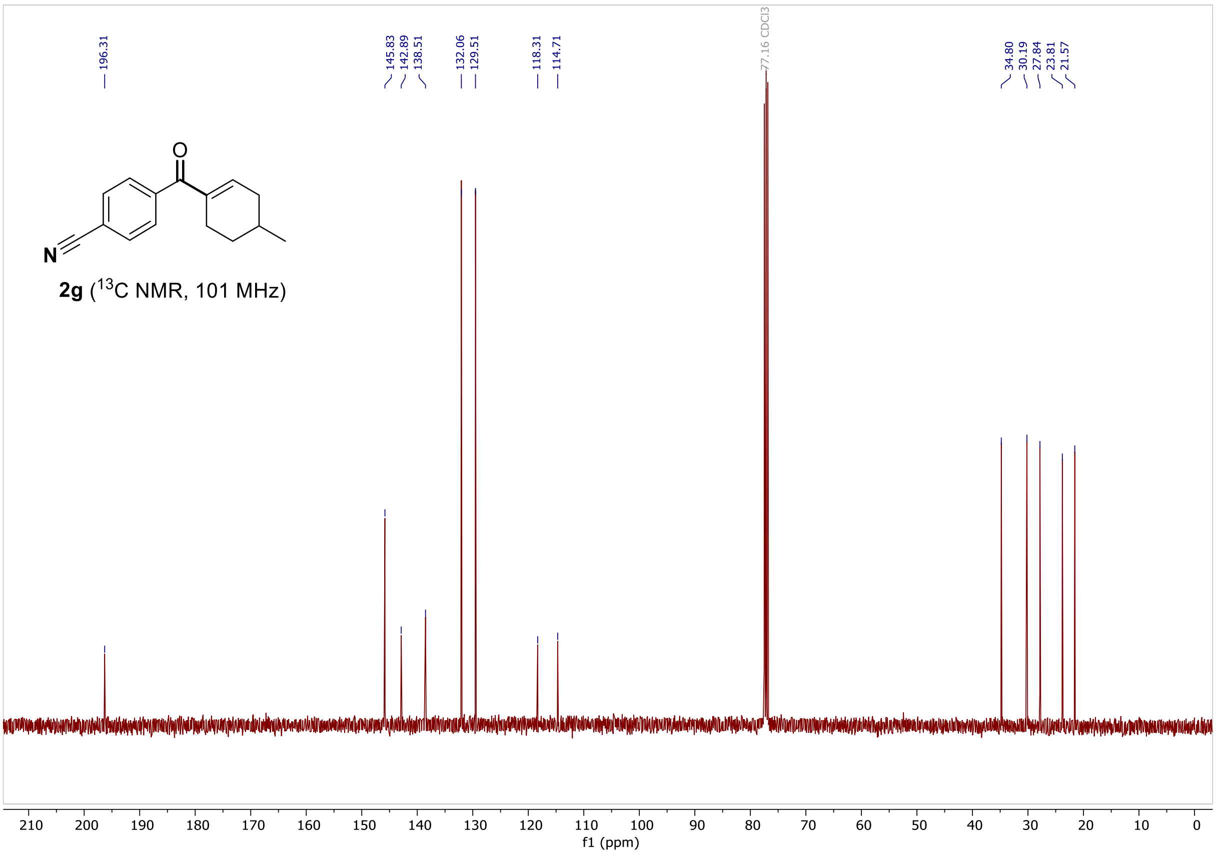
2g (13C NMR, 101 MHz)
f1 (ppm)
196.31
145.83
142.89
138.51
132.06
129.51
118.31
114.71
77.16 CDCl3
34.80
30.19
27.84
23.81
21.57

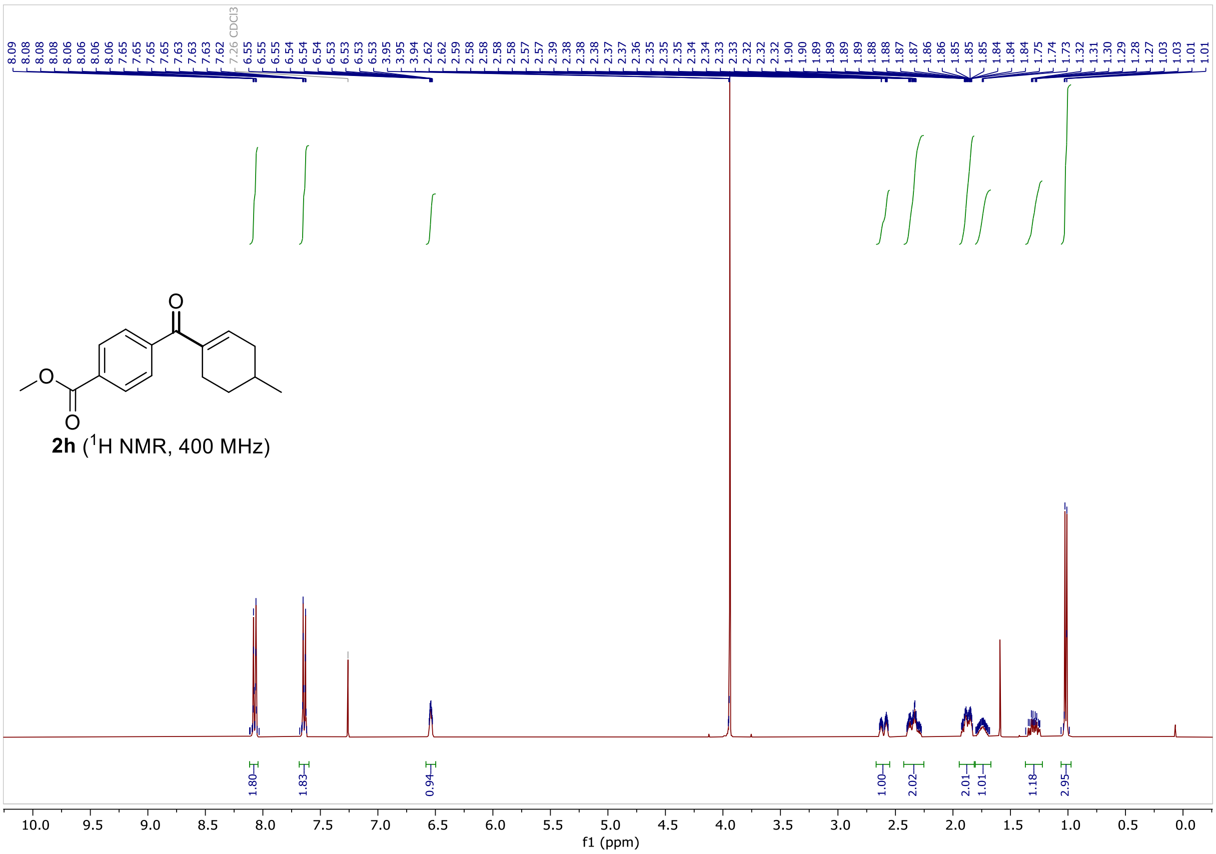

2h (1H NMR, 400 MHz)
f1 (ppm)

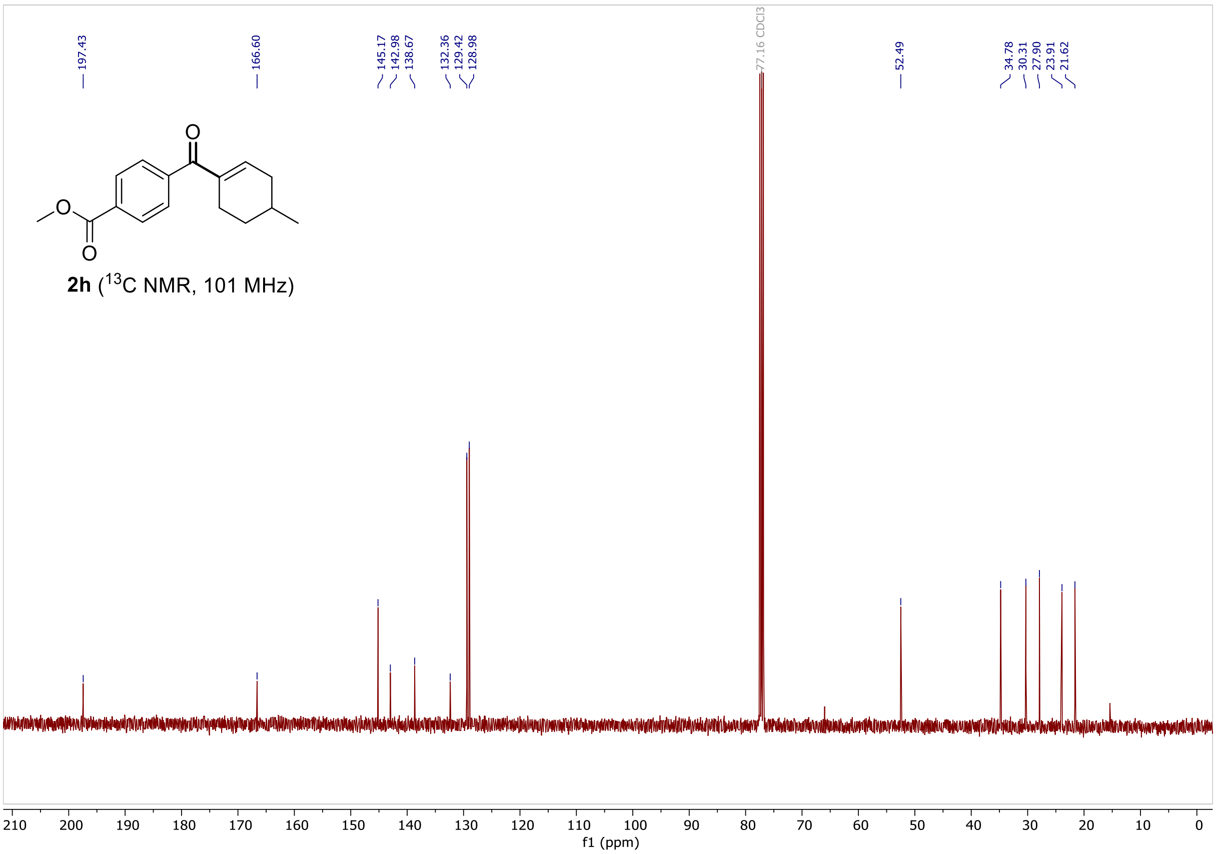

2h (13C NMR, 101 MHz)
f1 (ppm)

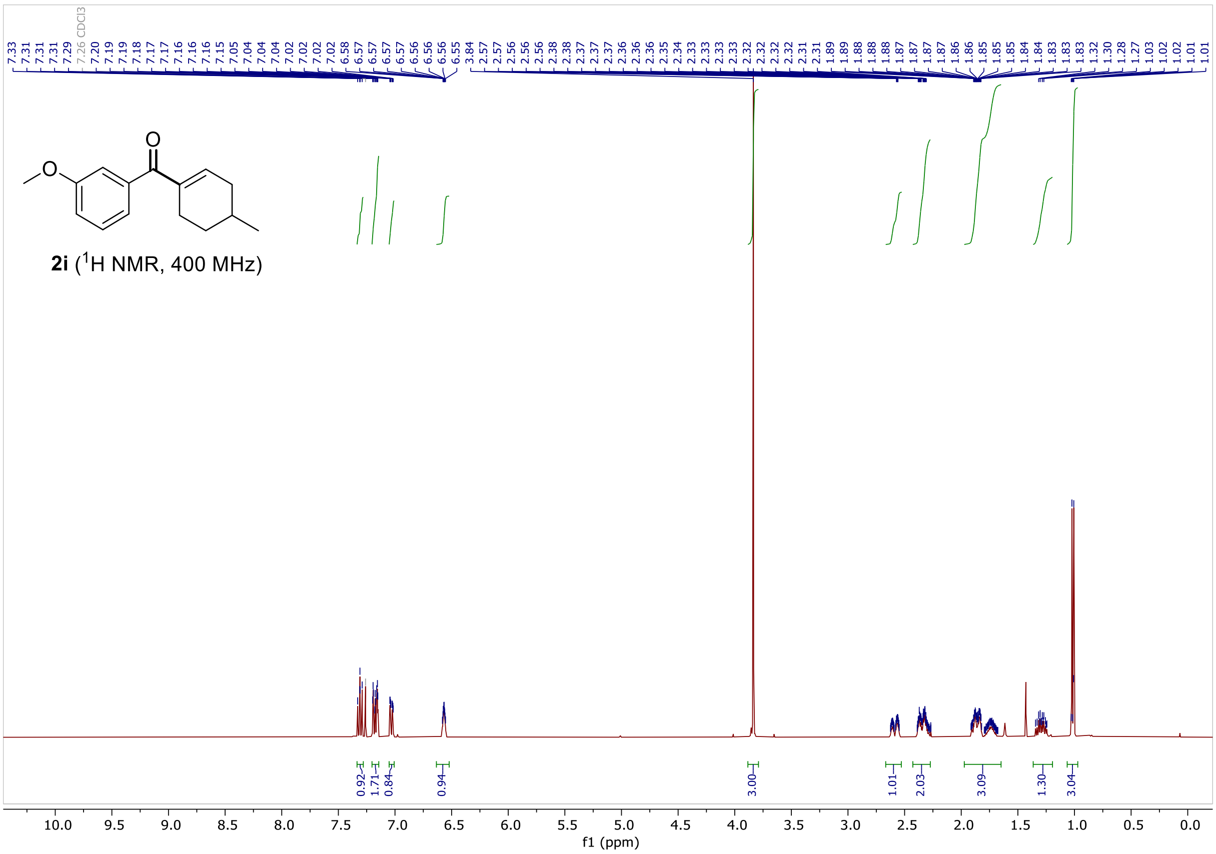
2i (1H NMR, 400 MHz)
f1 (ppm)

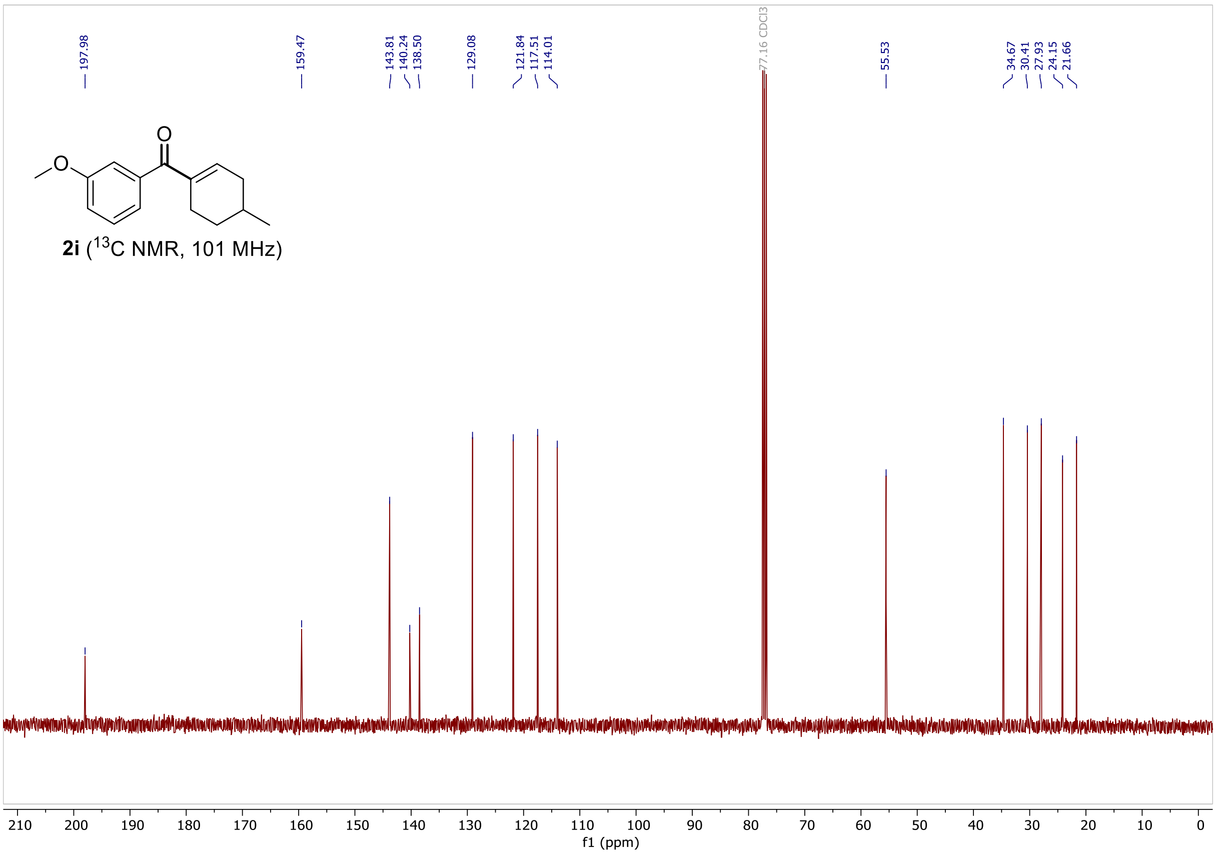
197.98
159.47
143.81
140.24
138.50
129.08
121.84
117.51
114.01
77.16 CDCl3
55.53
34.67
30.41
27.93
24.15
21.66
2i (13C NMR, 101 MHz)
f1 (ppm)

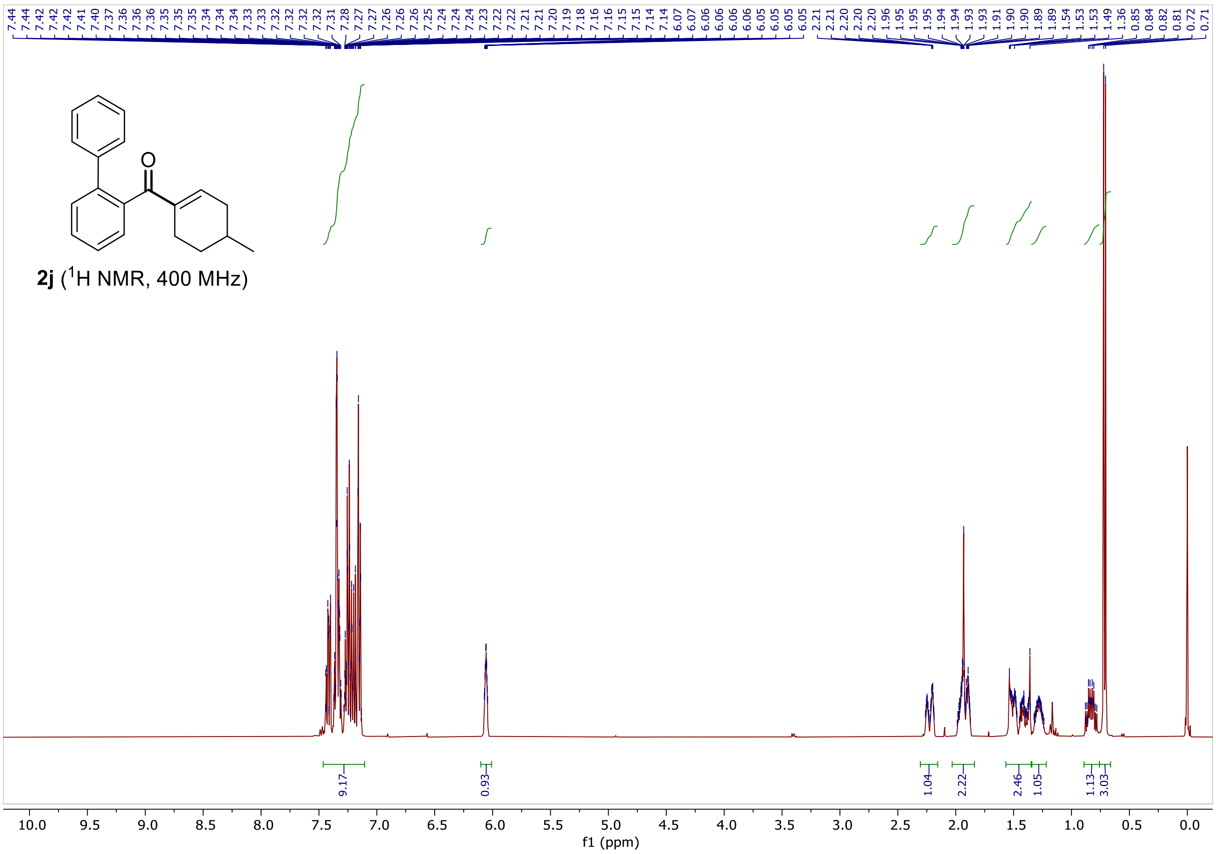

2j (^{1}H NMR, 400 MHz)

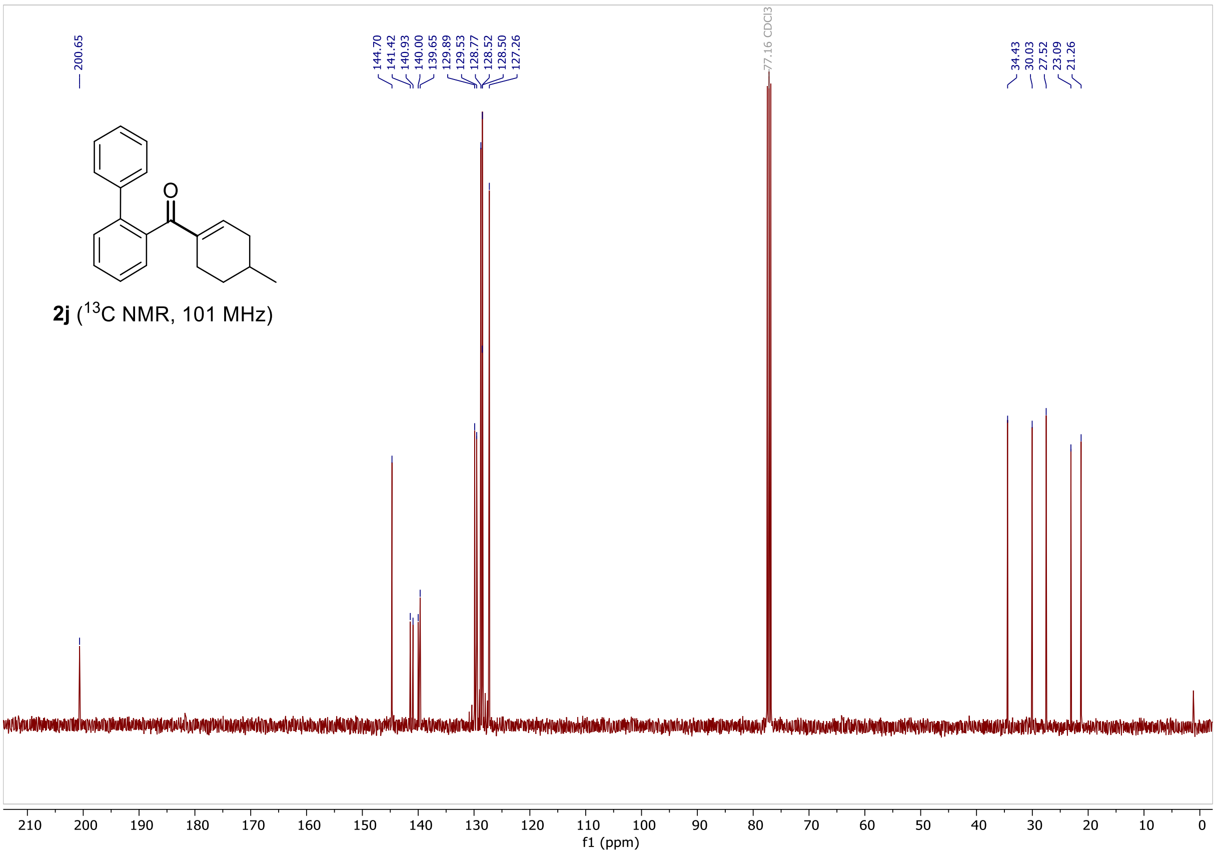

2j (^{13}C NMR, 101 MHz)

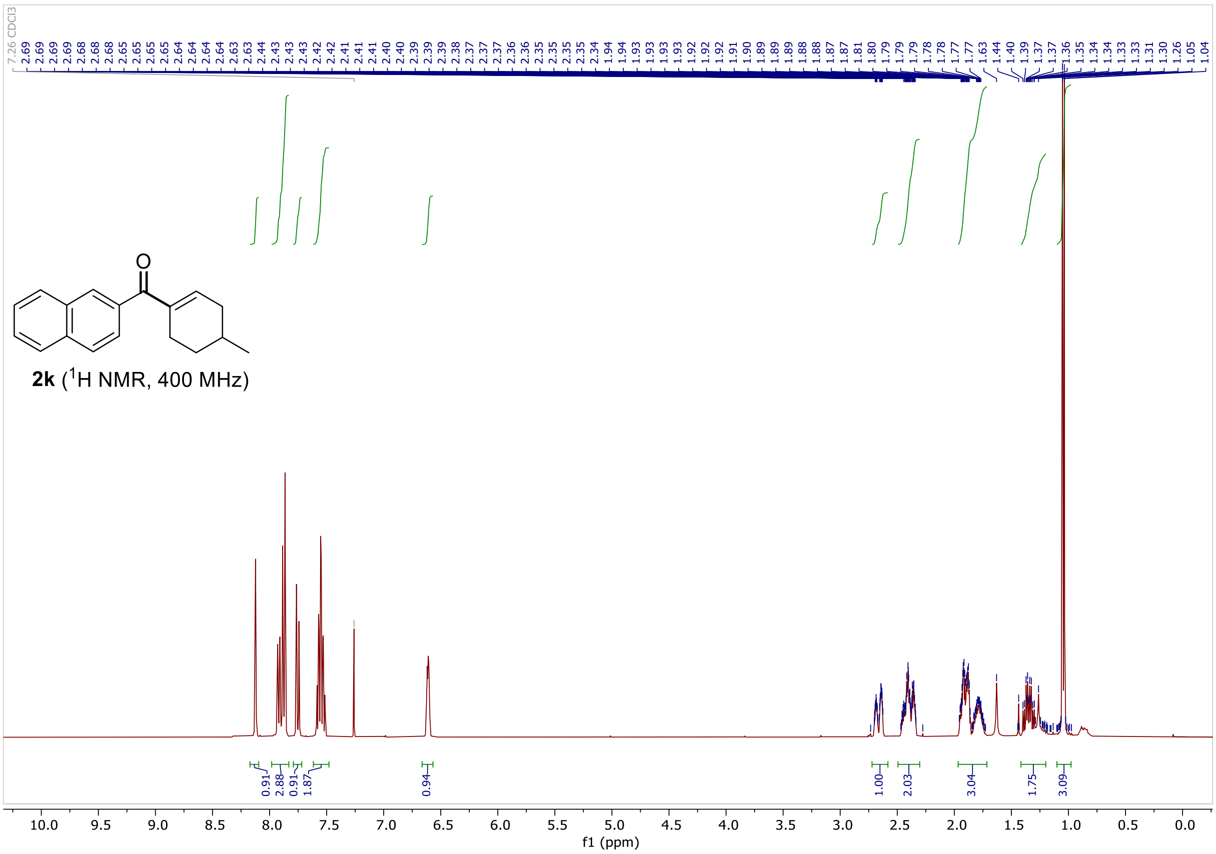
7.26 CDCl3
2.69 2.69 2.69 2.69 2.68 2.68 2.65 2.65 2.65 2.64 2.64 2.64 2.63 2.63 2.44 2.43 2.43 2.43 2.42 2.42 2.41 2.41 2.40 2.39 2.39 2.39 2.38 2.37 2.37 2.37 2.36 2.35 2.35 2.35 2.34 1.94 1.94 1.93 1.93 1.93 1.93 1.92 1.92 1.92 1.91 1.90 1.89 1.89 1.89 1.88 1.87 1.87 1.81 1.80 1.79 1.79 1.79 1.78 1.78 1.77 1.77 1.63 1.44 1.40 1.39 1.37 1.37 1.36 1.35 1.34 1.34 1.33 1.33 1.31 1.30 1.26 1.05 1.04
O
2k (1H NMR, 400 MHz)
0.91 2.88 0.91 1.87 0.94 1.00 2.03 3.04 1.75 3.09
10.0 9.5 9.0 8.5 8.0 7.5 7.0 6.5 6.0 5.5 5.0 4.5 4.0 3.5 3.0 2.5 2.0 1.5 1.0 0.5 0.0
f1 (ppm)

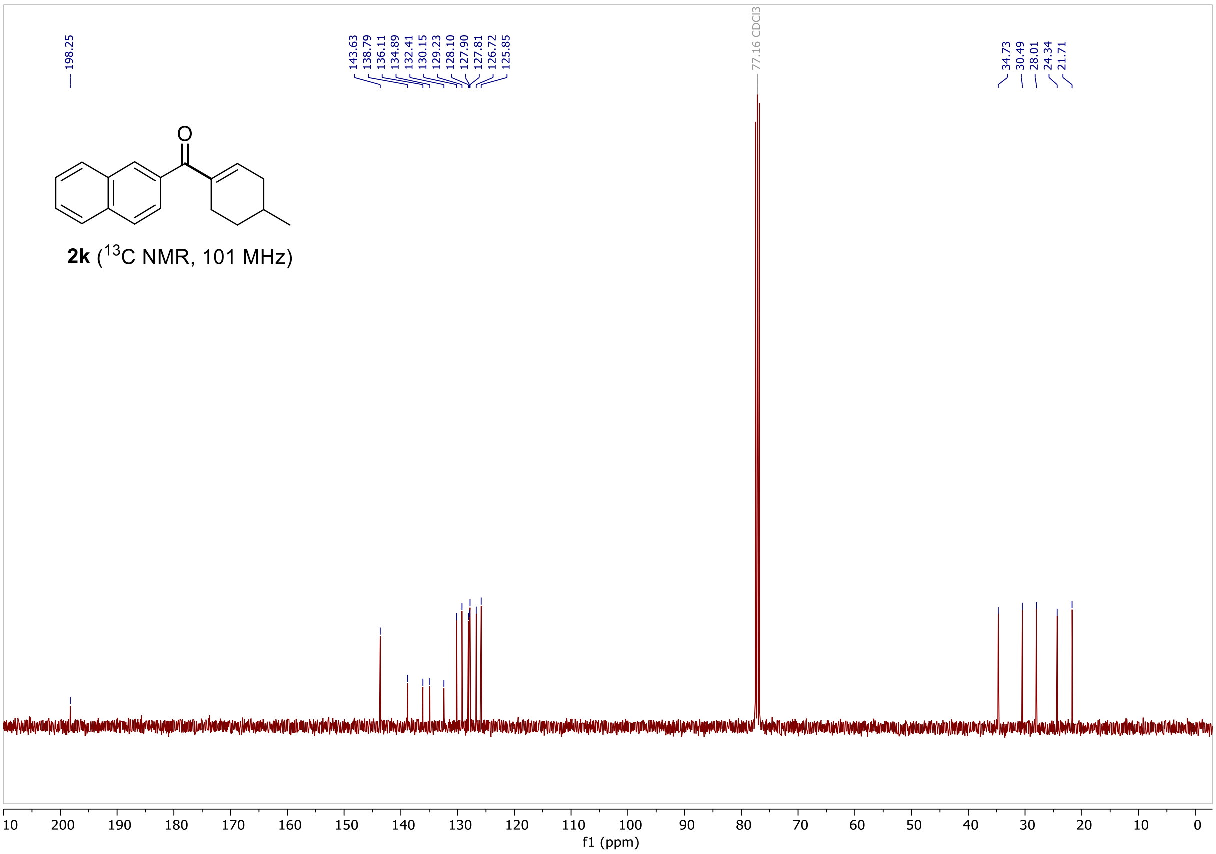
198.25
143.63 138.79 136.11 134.89 132.41 130.15 129.23 128.10 127.90 127.81 126.72 125.85
77.16 CDCl3
34.73 30.49 28.01 24.34 21.71
O
2k (13C NMR, 101 MHz)
10 200 190 180 170 160 150 140 130 120 110 100 90 80 70 60 50 40 30 20 10 0
f1 (ppm)

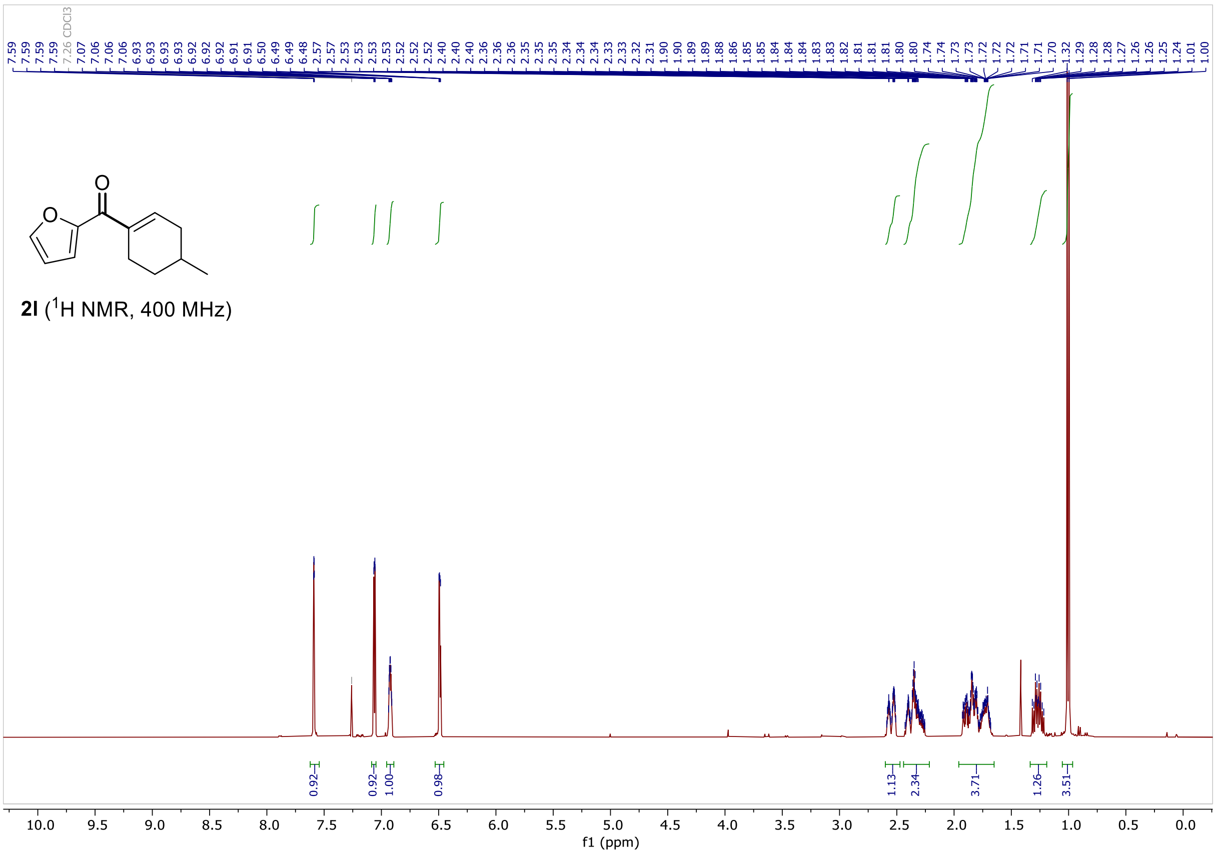

2l (^{1}H NMR, 400 MHz)

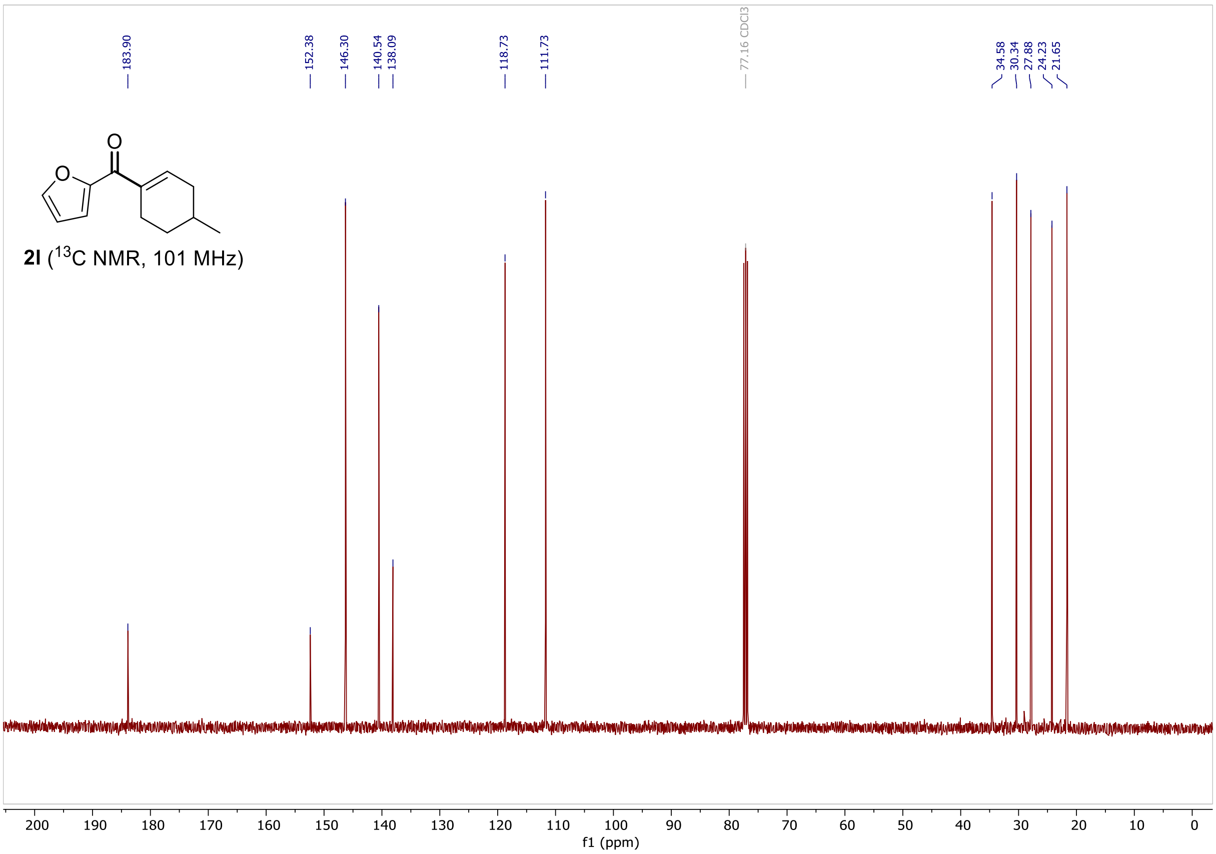

2l (^{13}C NMR, 101 MHz)

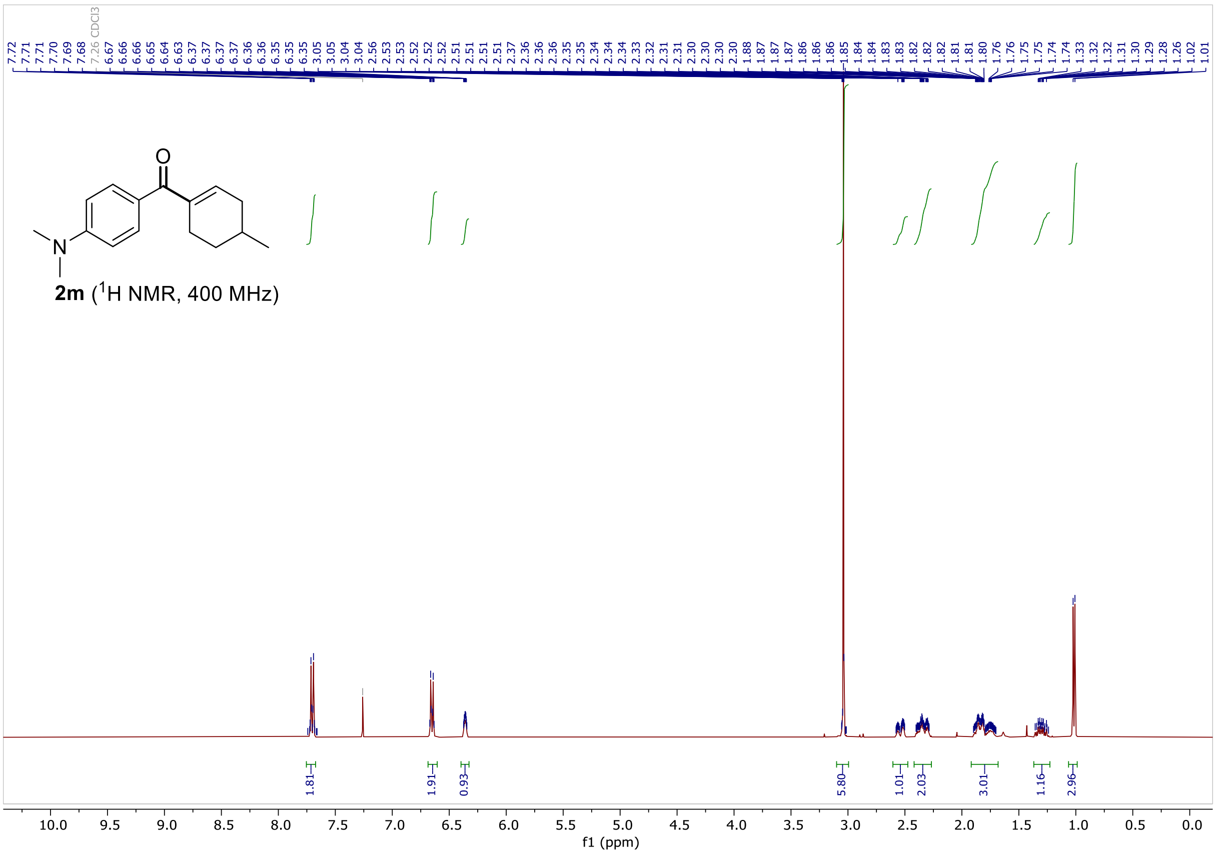
7.72 7.71 7.71 7.70 7.69 7.68 7.26 CDCl3 6.67 6.66 6.66 6.65 6.64 6.63 6.37 6.37 6.37 6.37 6.36 6.36 6.35 6.35 6.35 3.05 3.05 3.04 3.04 2.56 2.53 2.53 2.52 2.52 2.52 2.51 2.51 2.51 2.51 2.37 2.36 2.36 2.35 2.35 2.34 2.34 2.34 2.33 2.32 2.31 2.31 2.30 2.30 2.30 2.30 1.88 1.87 1.87 1.87 1.86 1.86 1.86 1.85 1.84 1.84 1.83 1.83 1.82 1.82 1.82 1.81 1.81 1.80 1.76 1.76 1.75 1.75 1.74 1.74 1.33 1.33 1.32 1.32 1.31 1.30 1.29 1.28 1.26 1.02 1.01
O
N
2m (1H NMR, 400 MHz)
1.81 1.91 0.93 5.80 1.01 2.03 3.01 1.16 2.96
10.0 9.5 9.0 8.5 8.0 7.5 7.0 6.5 6.0 5.5 5.0 4.5 4.0 3.5 3.0 2.5 2.0 1.5 1.0 0.5 0.0
f1 (ppm)

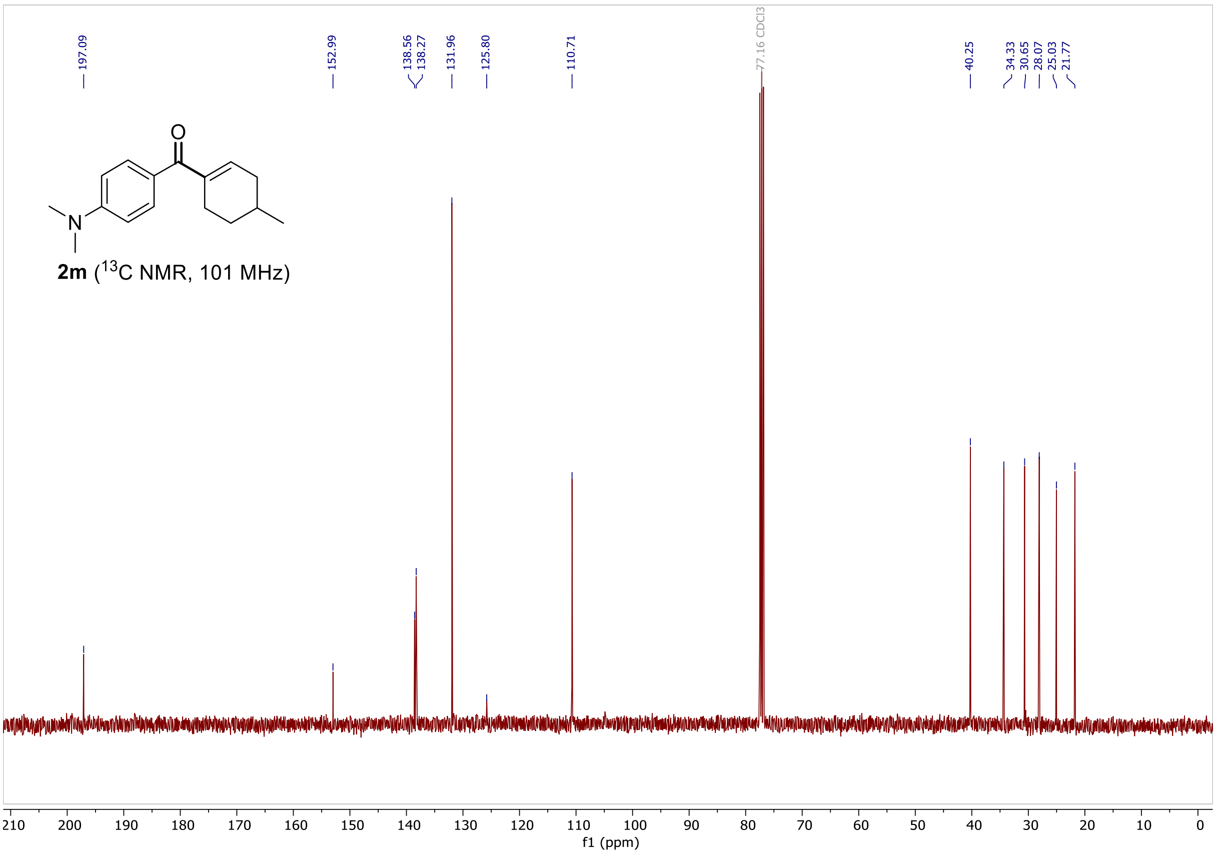
197.09 152.99 138.56 138.27 131.96 125.80 110.71 77.16 CDCl3 40.25 34.33 30.65 28.07 25.03 21.77
O
N
2m (13C NMR, 101 MHz)
210 200 190 180 170 160 150 140 130 120 110 100 90 80 70 60 50 40 30 20 10 0
f1 (ppm)

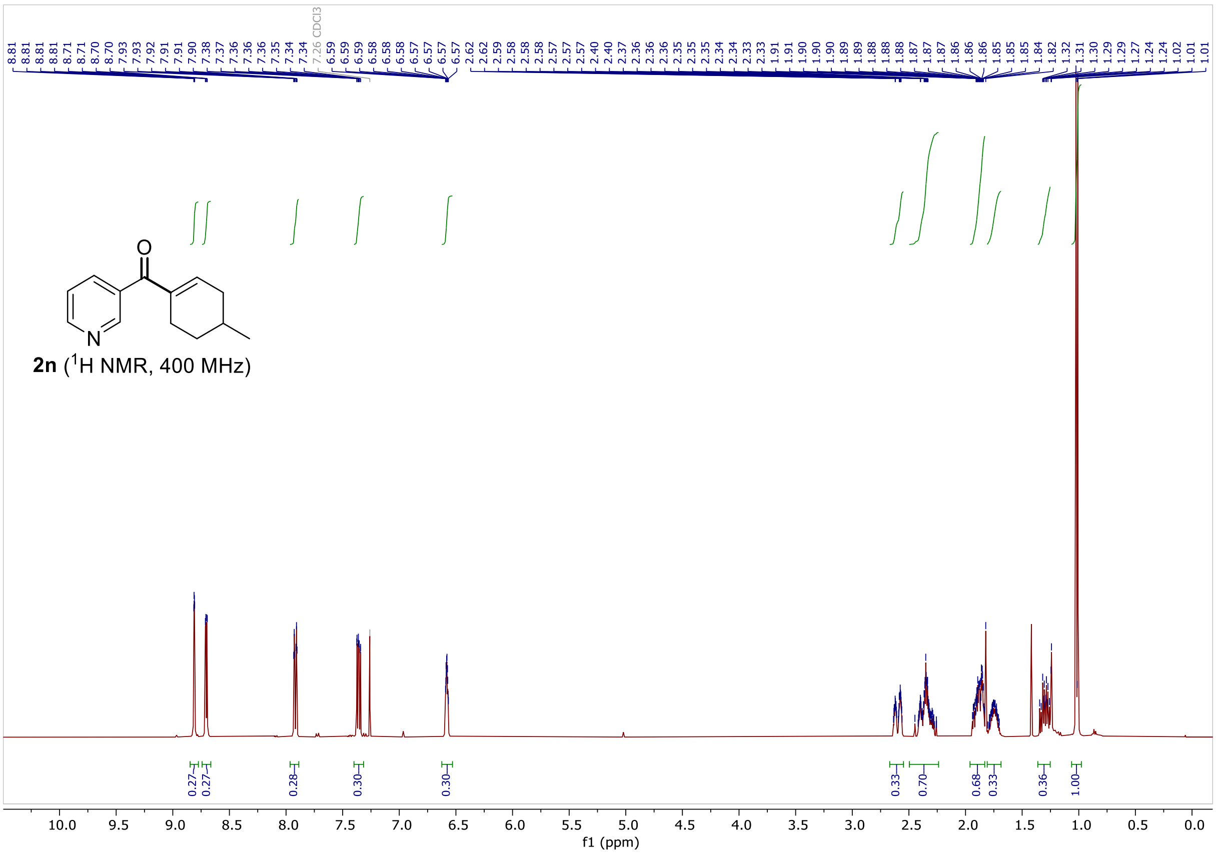
2n (1H NMR, 400 MHz)
f1 (ppm)

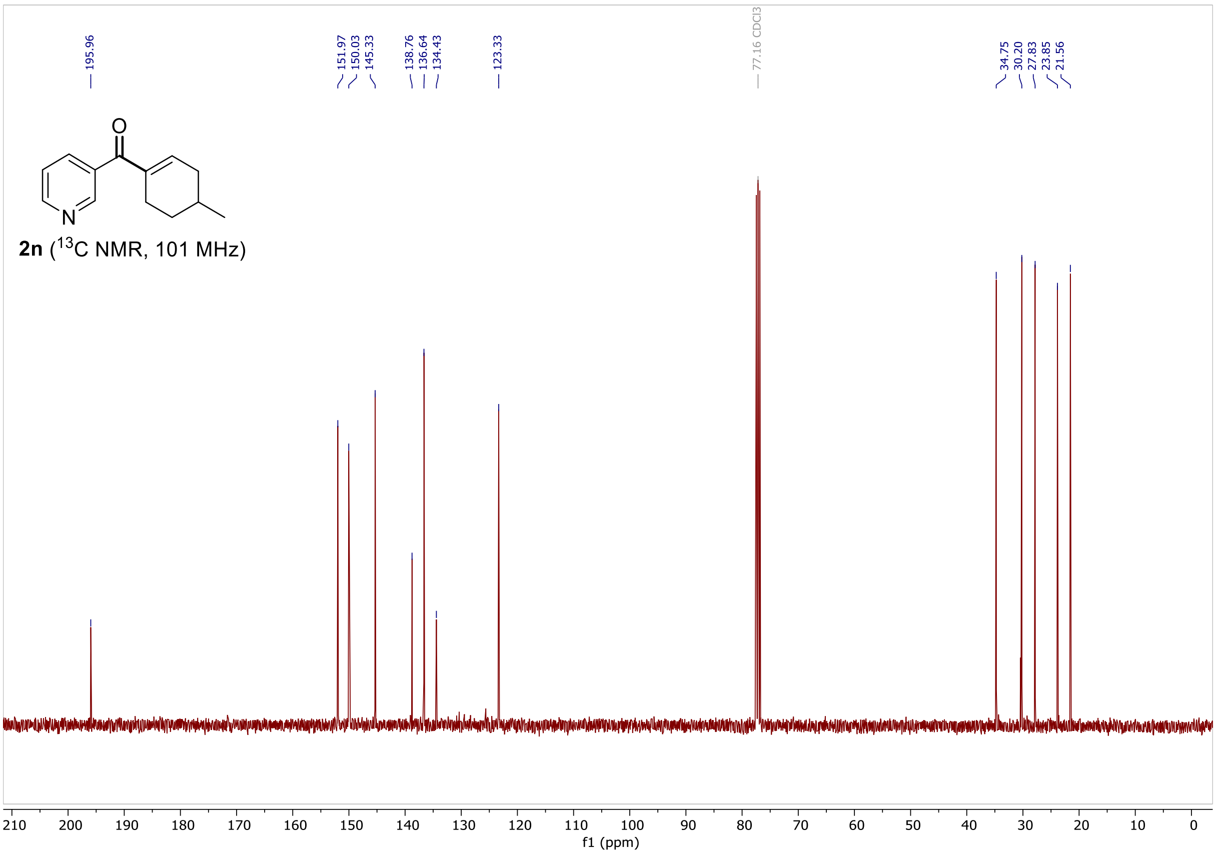
2n (13C NMR, 101 MHz)
195.96
151.97
150.03
145.33
138.76
136.64
134.43
123.33
77.16 CDCl3
34.75
30.20
27.83
23.85
21.56
f1 (ppm)

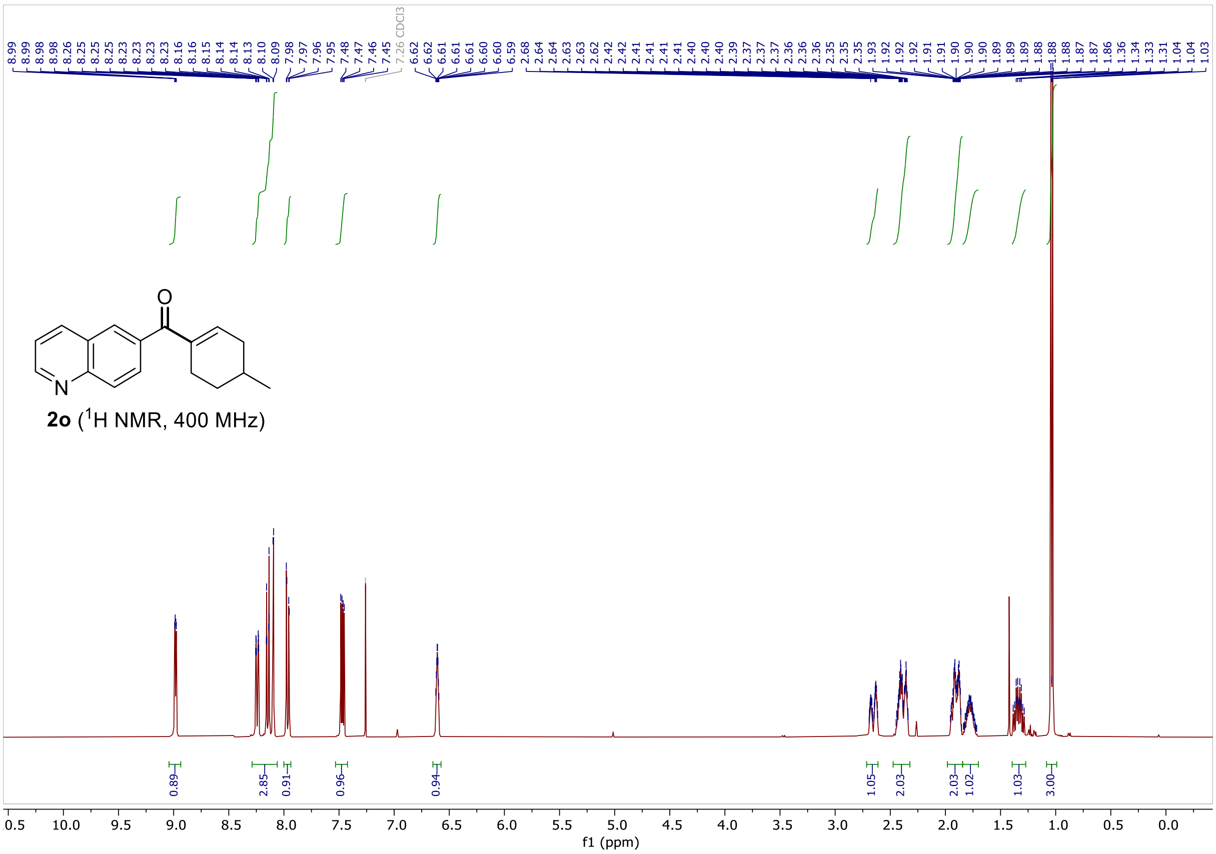
8.99 8.99 8.98 8.98 8.26 8.25 8.25 8.23 8.23 8.23 8.23 8.16 8.16 8.15 8.14 8.14 8.13 8.10 8.09 7.98 7.97 7.96 7.95 7.48 7.47 7.46 7.45 7.26 CDCl3 6.62 6.62 6.61 6.61 6.61 6.60 6.59 2.68 2.64 2.64 2.63 2.63 2.62 2.42 2.42 2.41 2.41 2.41 2.41 2.40 2.40 2.40 2.39 2.37 2.37 2.37 2.36 2.36 2.36 2.35 2.35 2.35 1.93 1.92 1.92 1.92 1.91 1.91 1.90 1.90 1.90 1.89 1.89 1.89 1.88 1.88 1.88 1.87 1.86 1.36 1.34 1.33 1.31 1.04 1.03
N
O
2o (1H NMR, 400 MHz)
0.89 2.85 0.91 0.96 0.94 1.05 2.03 2.03 1.02 1.03 3.00
f1 (ppm)

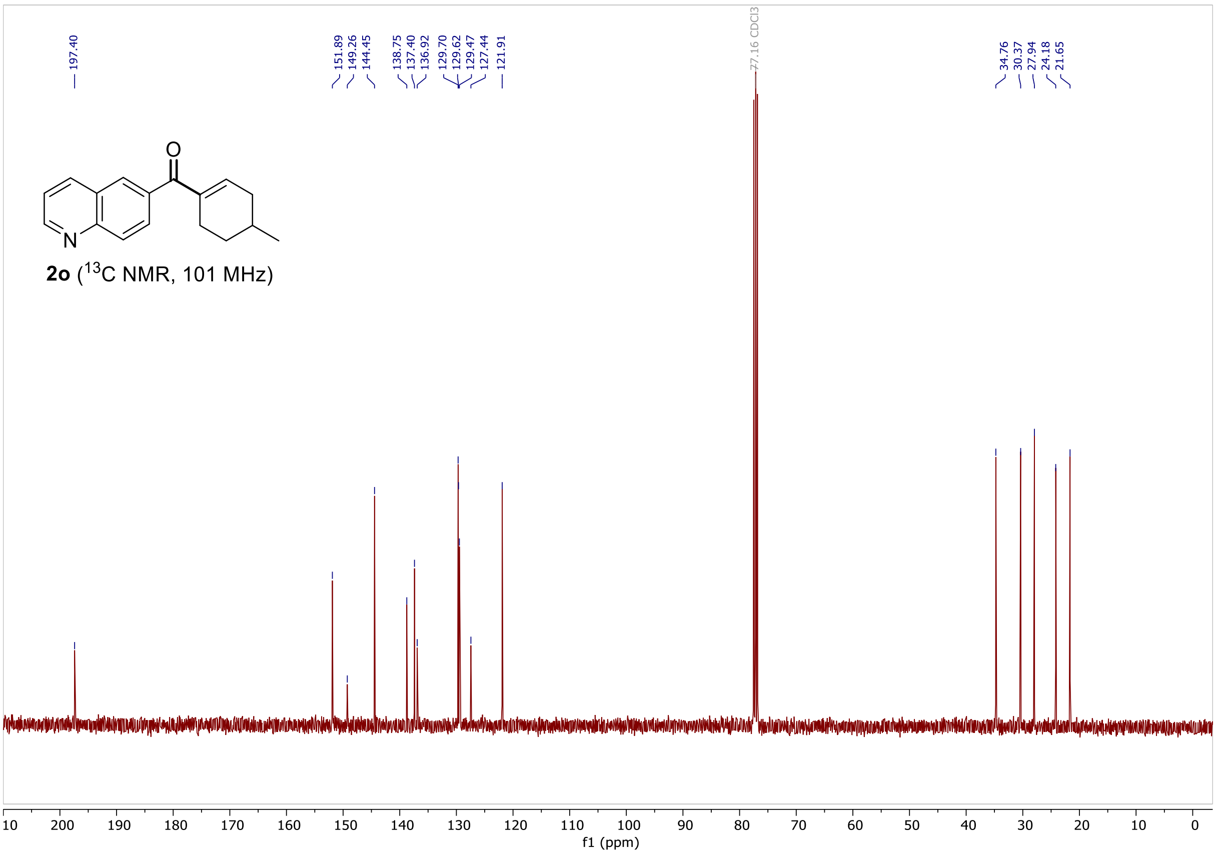
197.40
151.89 149.26 144.45 138.75 137.40 136.92 129.70 129.62 129.47 127.44 121.91
77.16 CDCl3
34.76 30.37 27.94 24.18 21.65
N
O
2o (13C NMR, 101 MHz)
f1 (ppm)

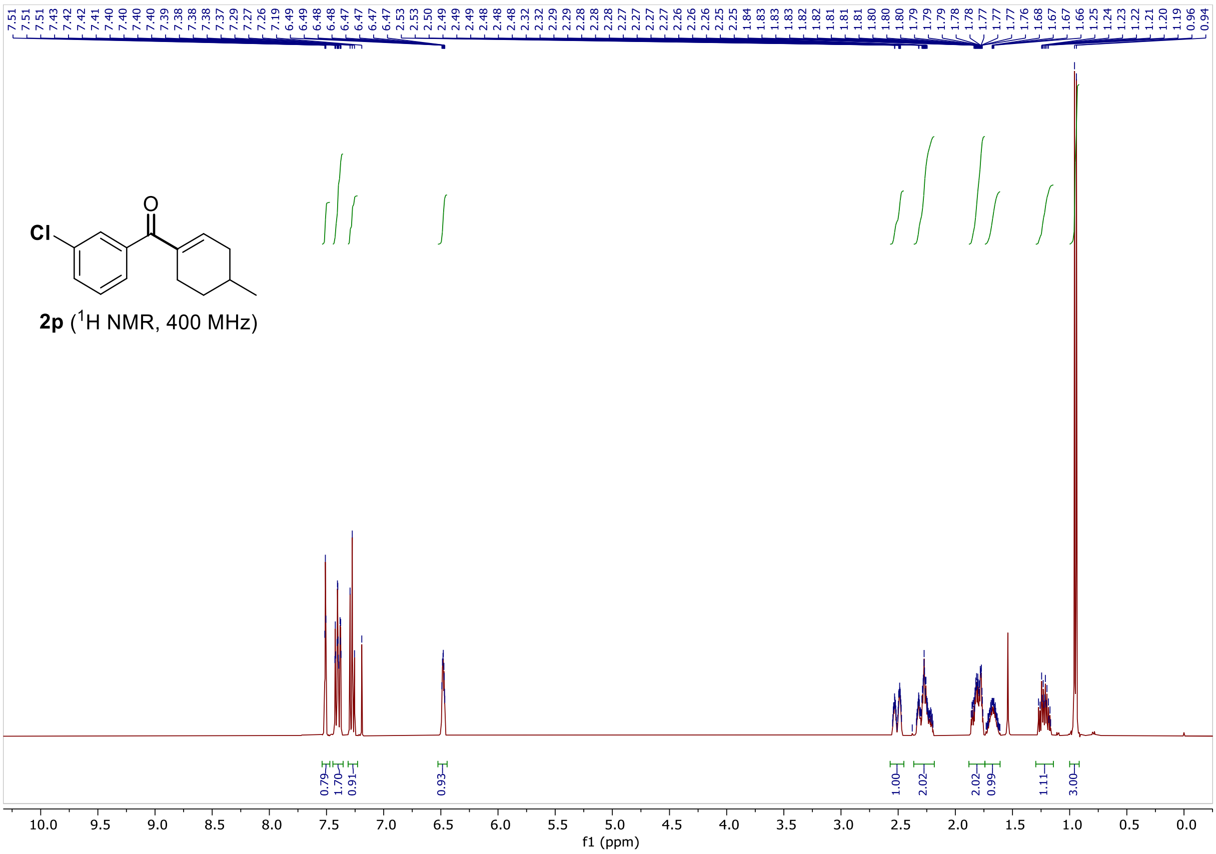
2p (¹H NMR, 400 MHz)
f1 (ppm)

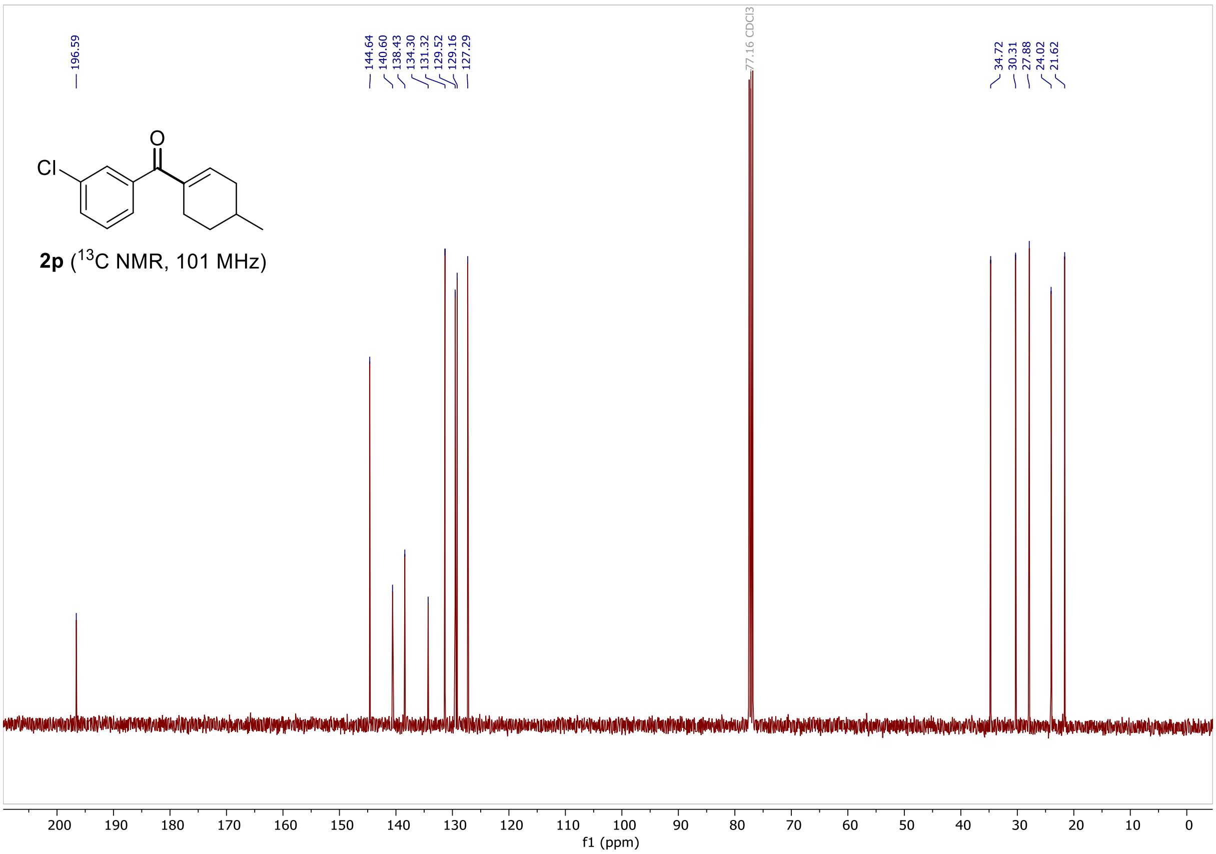
2p (¹³C NMR, 101 MHz)
196.59
144.64
140.60
138.43
134.30
131.32
129.52
129.16
127.29
77.16 CDCl3
34.72
30.31
27.88
24.02
21.62
f1 (ppm)

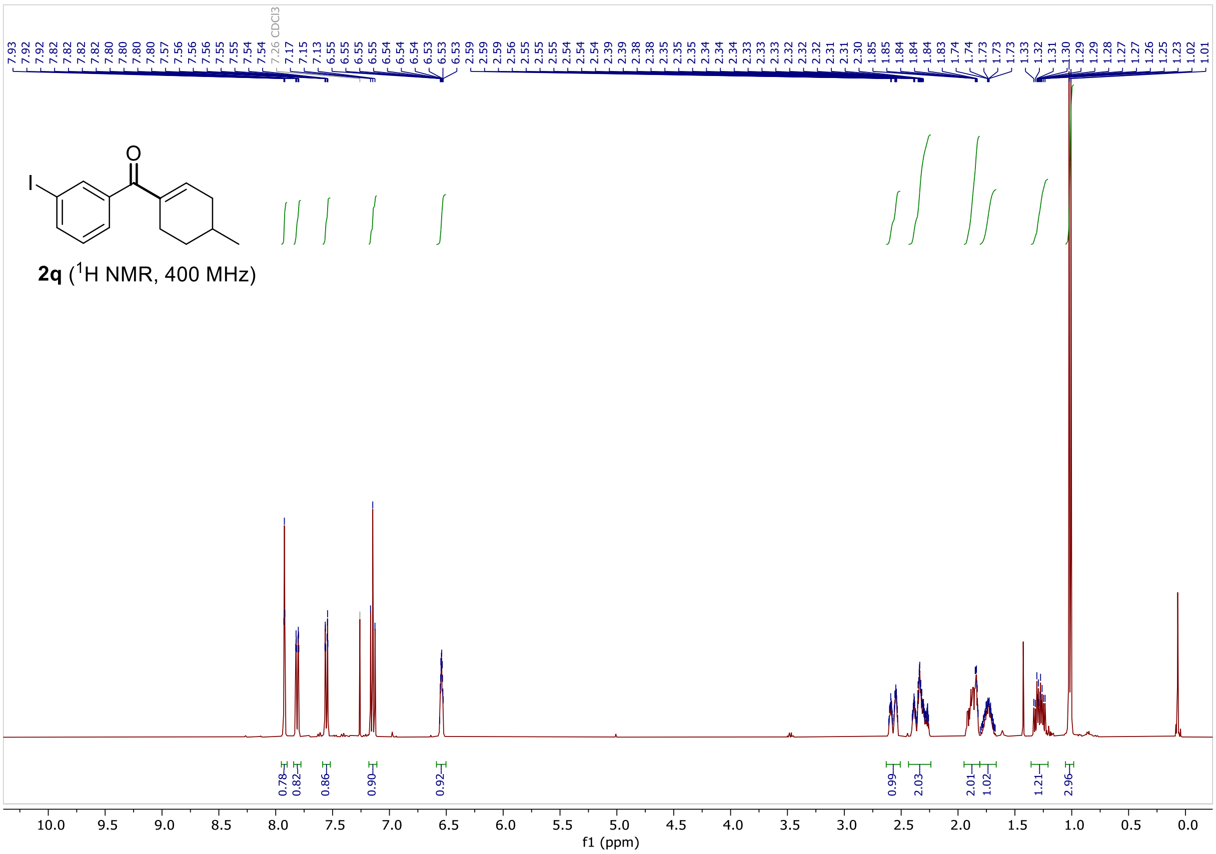
2q (1H NMR, 400 MHz)
f1 (ppm)

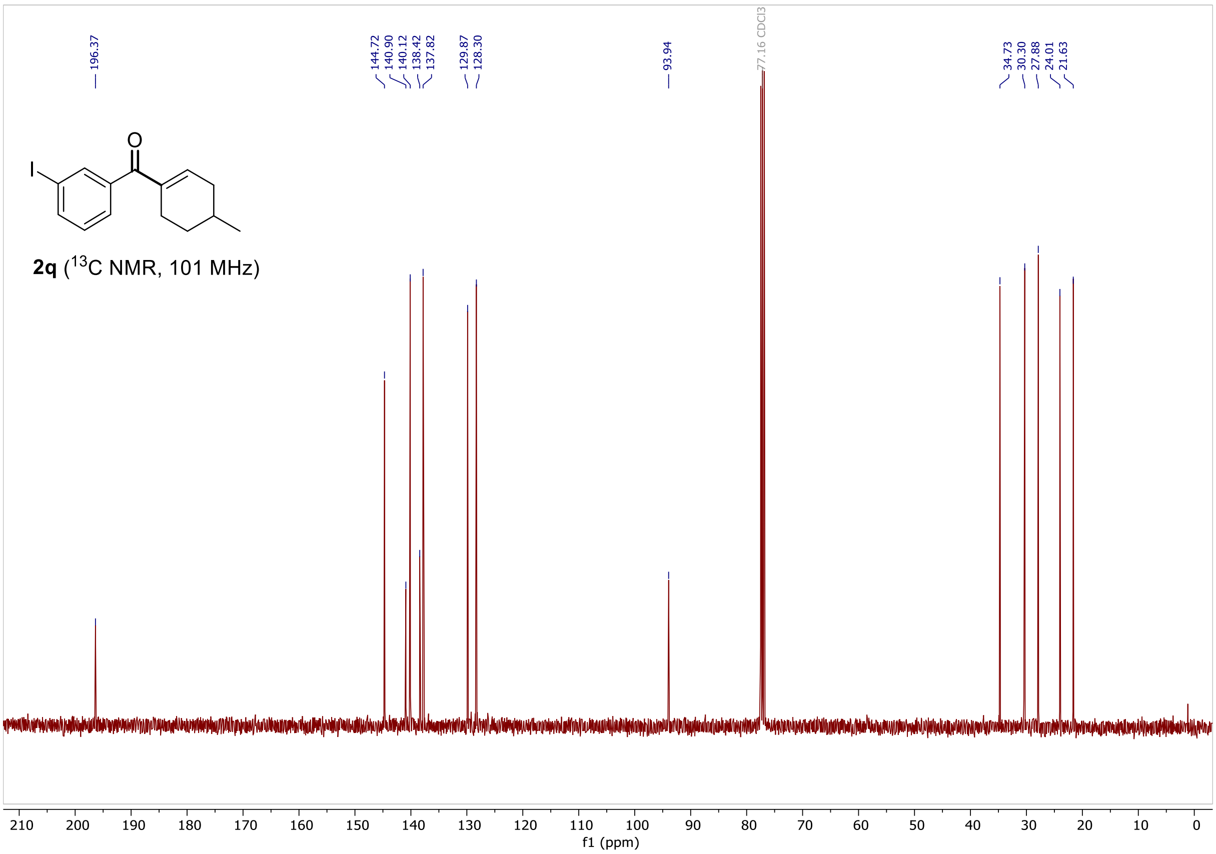
2q (13C NMR, 101 MHz)
196.37
144.72
140.90
140.12
138.42
137.82
129.87
128.30
93.94
77.16 CDCl3
34.73
30.30
27.88
24.01
21.63
f1 (ppm)

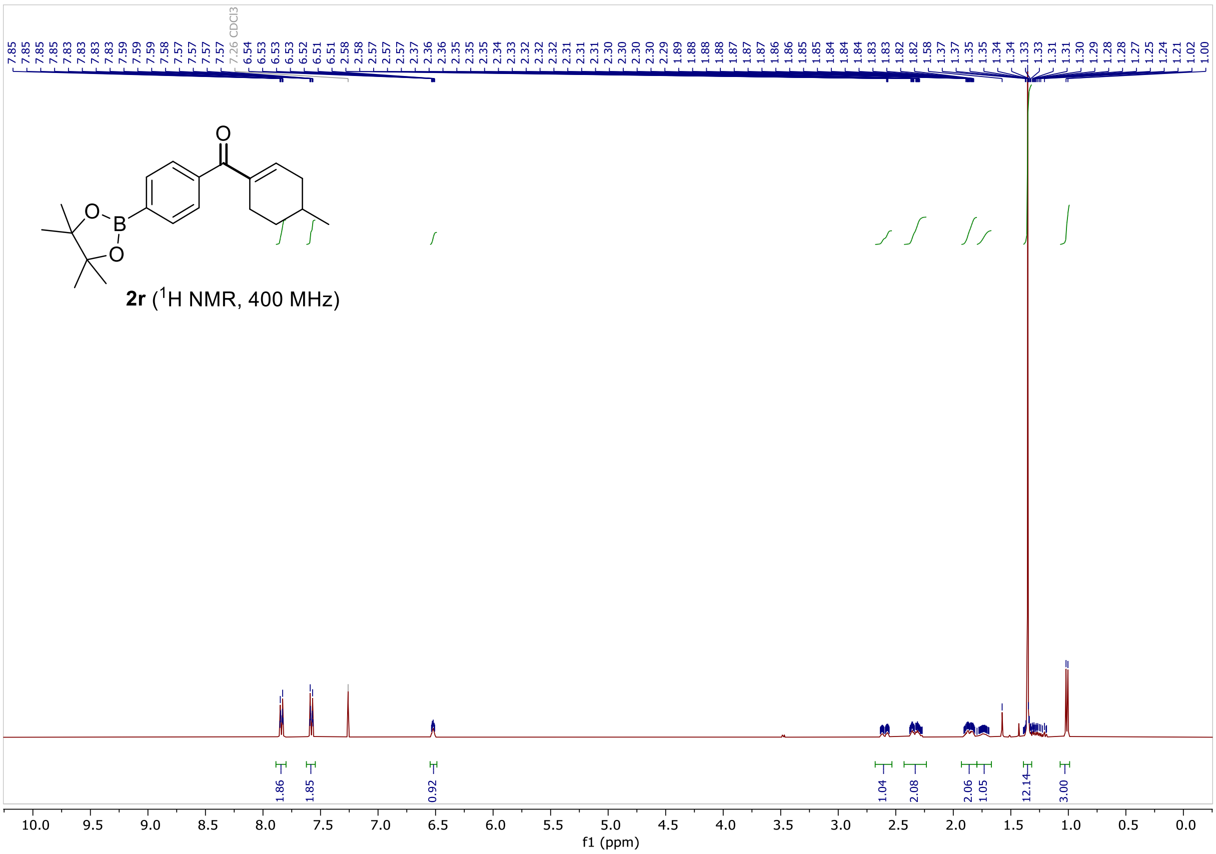
2r (¹H NMR, 400 MHz)
7.26 CDCl3
1.86
1.85
0.92
1.04
2.08
2.06
1.05
12.14
3.00
f1 (ppm)

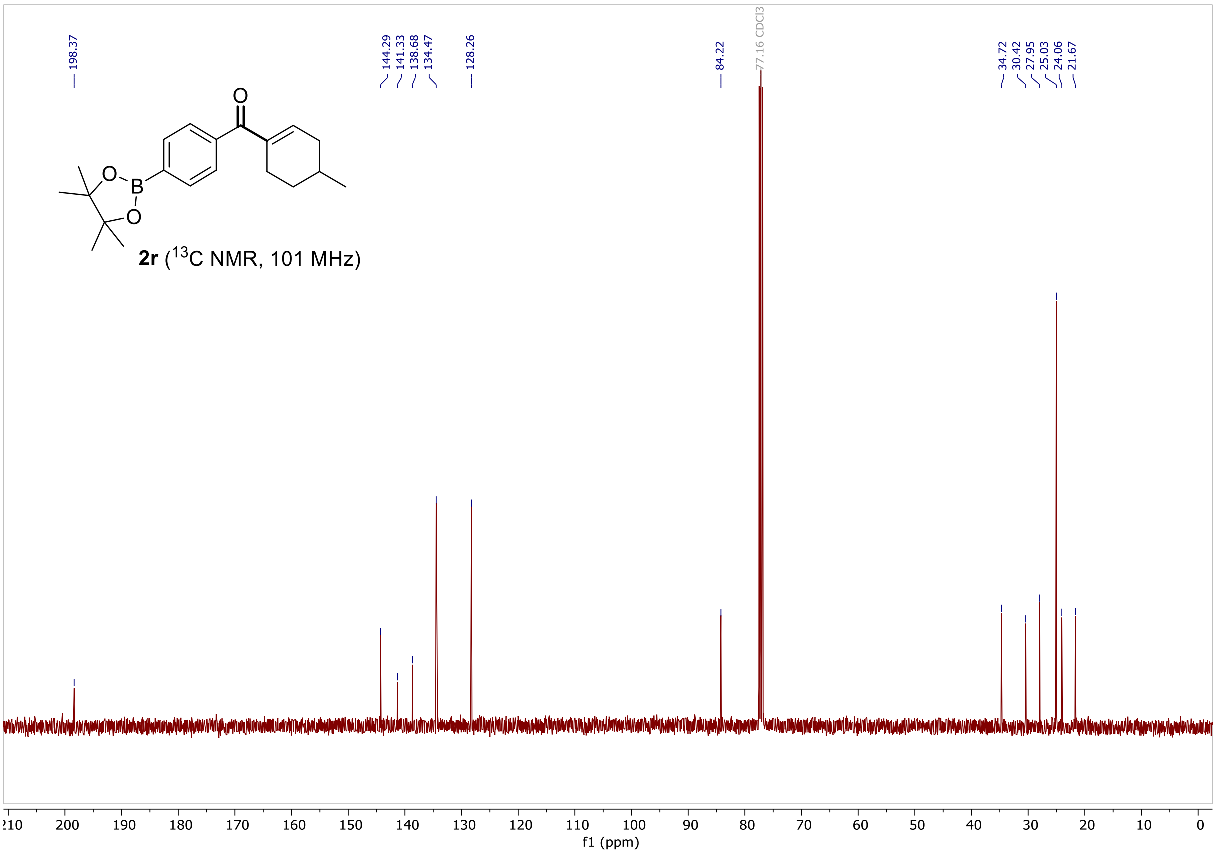
2r (¹³C NMR, 101 MHz)
198.37
144.29
141.33
138.68
134.47
128.26
84.22
77.16 CDCl3
34.72
30.42
27.95
25.03
24.06
21.67
f1 (ppm)

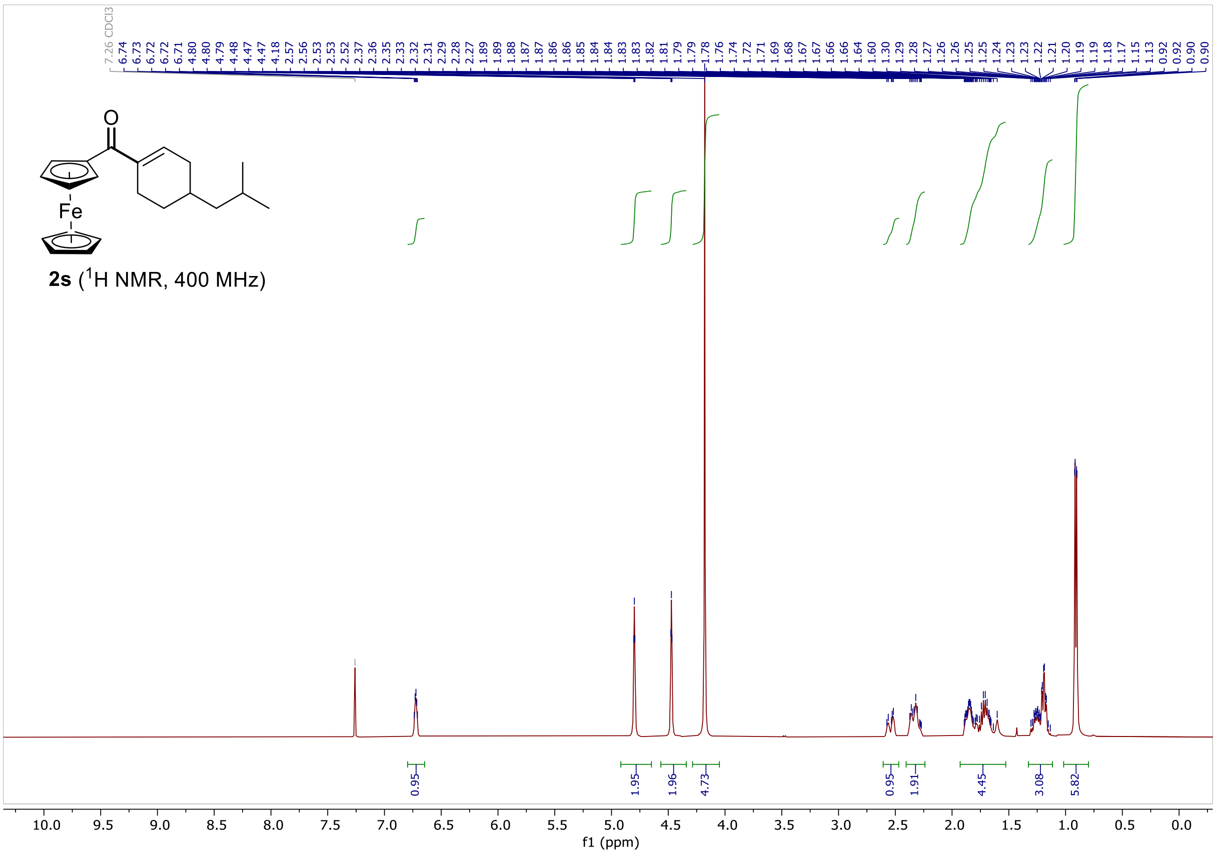

Fe
2s (1H NMR, 400 MHz)
f1 (ppm)

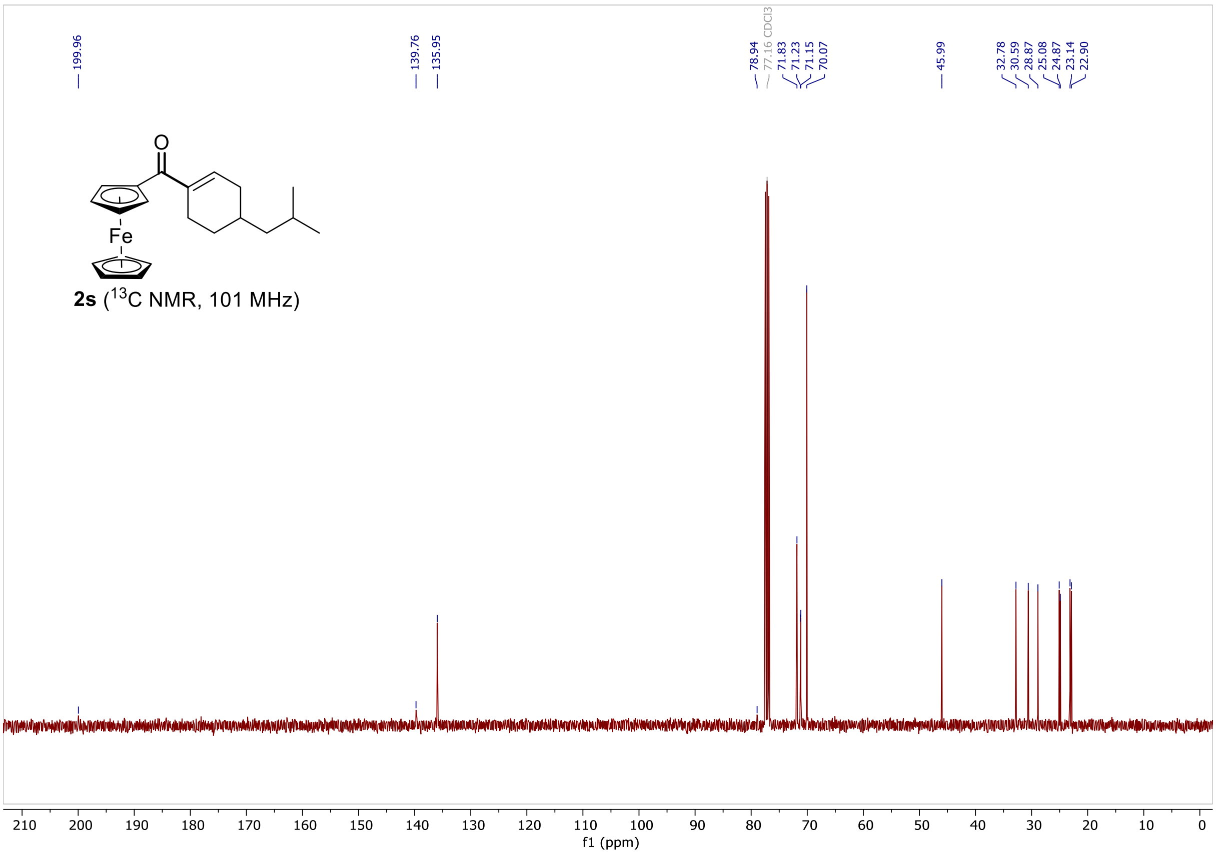

Fe
2s (13C NMR, 101 MHz)
199.96
139.76
135.95
78.94
77.16 CDCl3
71.83
71.23
71.15
70.07
45.99
32.78
30.59
28.87
25.08
24.87
23.14
22.90
f1 (ppm)

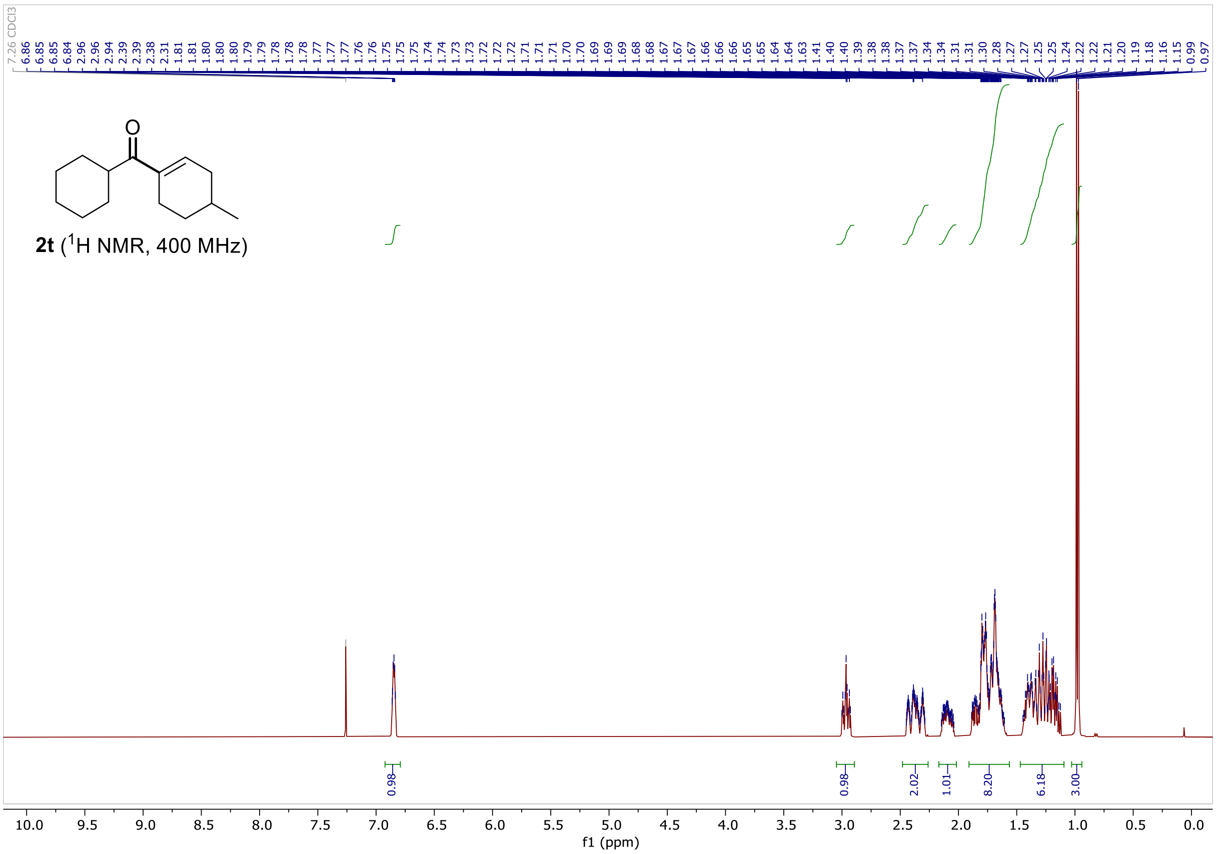
O
2t (1H NMR, 400 MHz)
7.26 CDCl3
6.86 6.85 6.85 6.84 2.96 2.96 2.94 2.39 2.39 2.38 2.31 1.81 1.81 1.80 1.80 1.80 1.79 1.79 1.78 1.78 1.78 1.77 1.77 1.77 1.76 1.75 1.75 1.75 1.74 1.74 1.73 1.73 1.72 1.72 1.72 1.71 1.71 1.71 1.70 1.70 1.69 1.69 1.69 1.68 1.68 1.67 1.67 1.67 1.66 1.66 1.66 1.65 1.65 1.64 1.64 1.63 1.41 1.40 1.40 1.39 1.38 1.38 1.37 1.37 1.34 1.34 1.31 1.31 1.30 1.28 1.27 1.27 1.25 1.25 1.24 1.22 1.22 1.22 1.21 1.20 1.19 1.18 1.16 1.15 0.99 0.97
0.98
0.98
2.02
1.01
8.20
6.18
3.00
10.0 9.5 9.0 8.5 8.0 7.5 7.0 6.5 6.0 5.5 5.0 4.5 4.0 3.5 3.0 2.5 2.0 1.5 1.0 0.5 0.0
f1 (ppm)

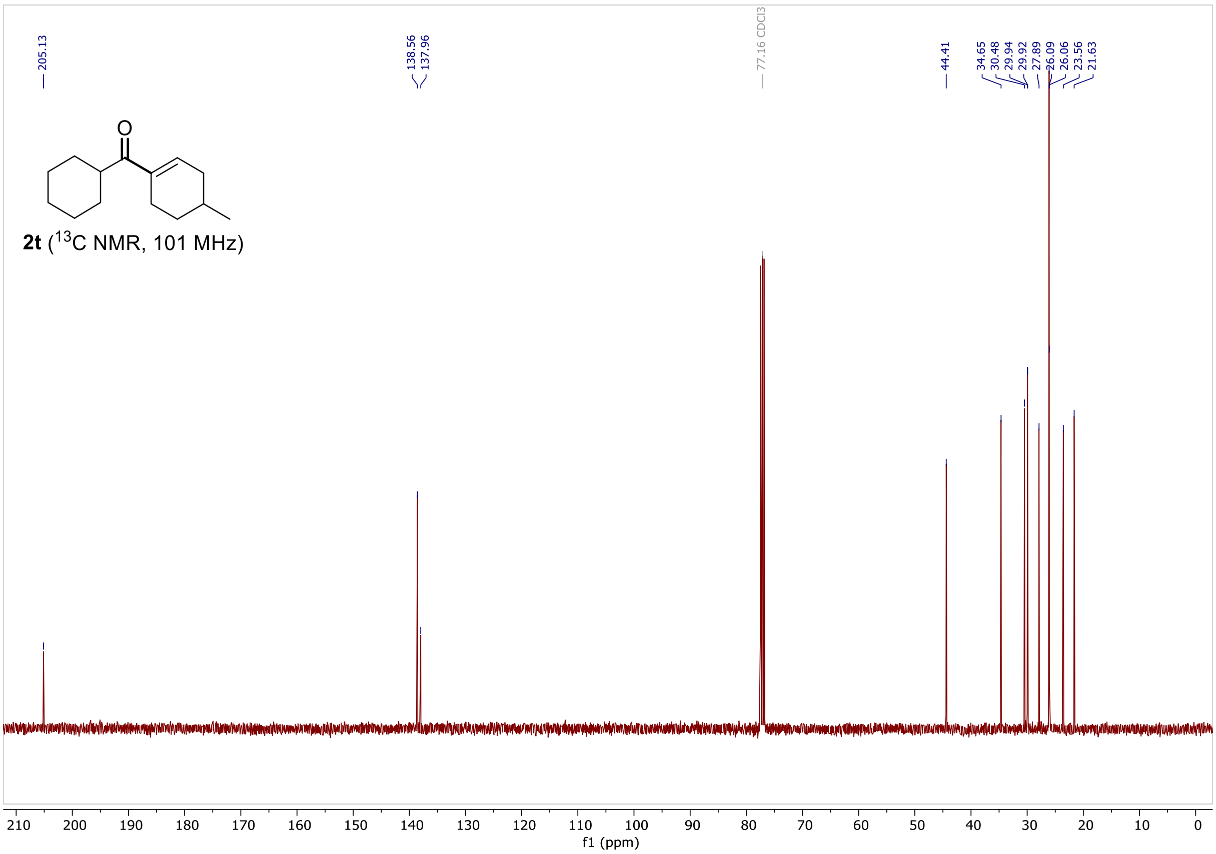
O
2t (13C NMR, 101 MHz)
205.13
138.56
137.96
77.16 CDCl3
44.41
34.65
30.48
29.94
29.92
27.89
26.09
26.06
23.56
21.63
210 200 190 180 170 160 150 140 130 120 110 100 90 80 70 60 50 40 30 20 10 0
f1 (ppm)

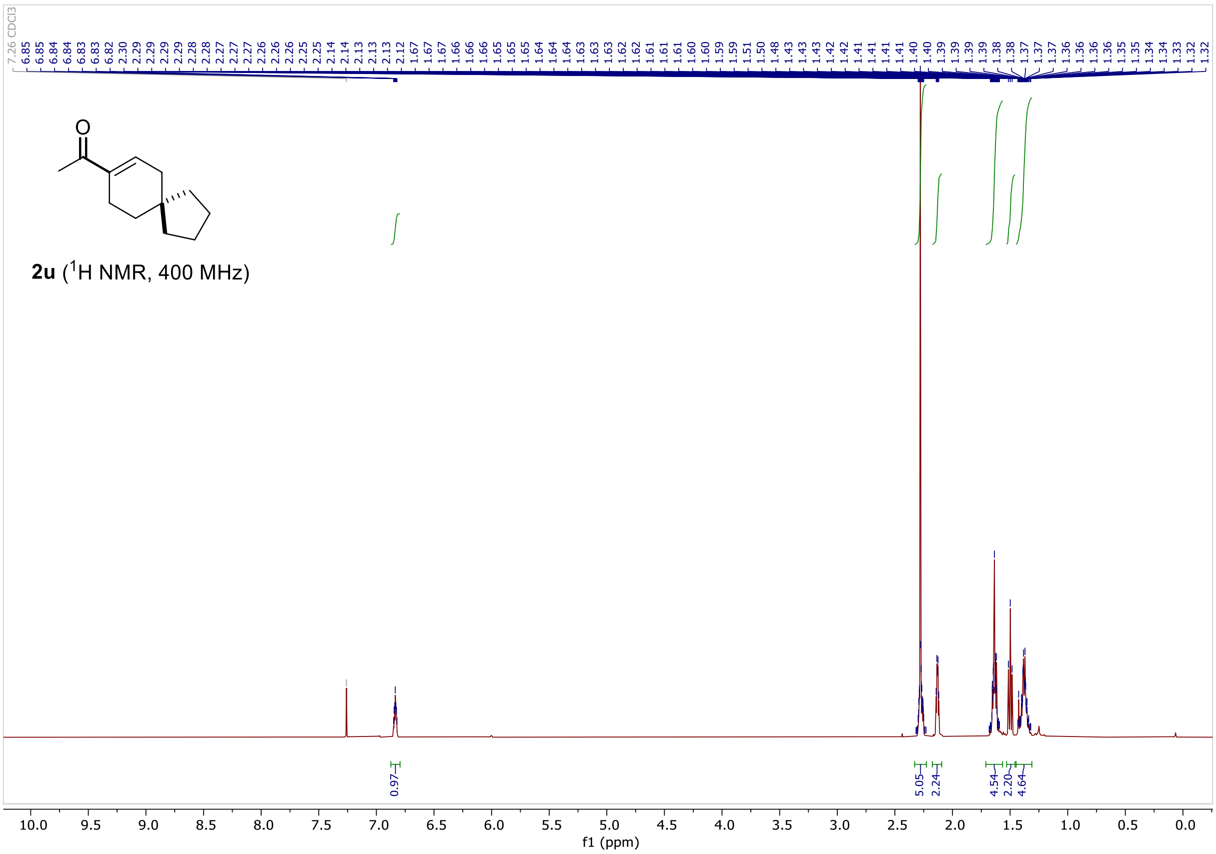

2u (^{1}H NMR, 400 MHz)

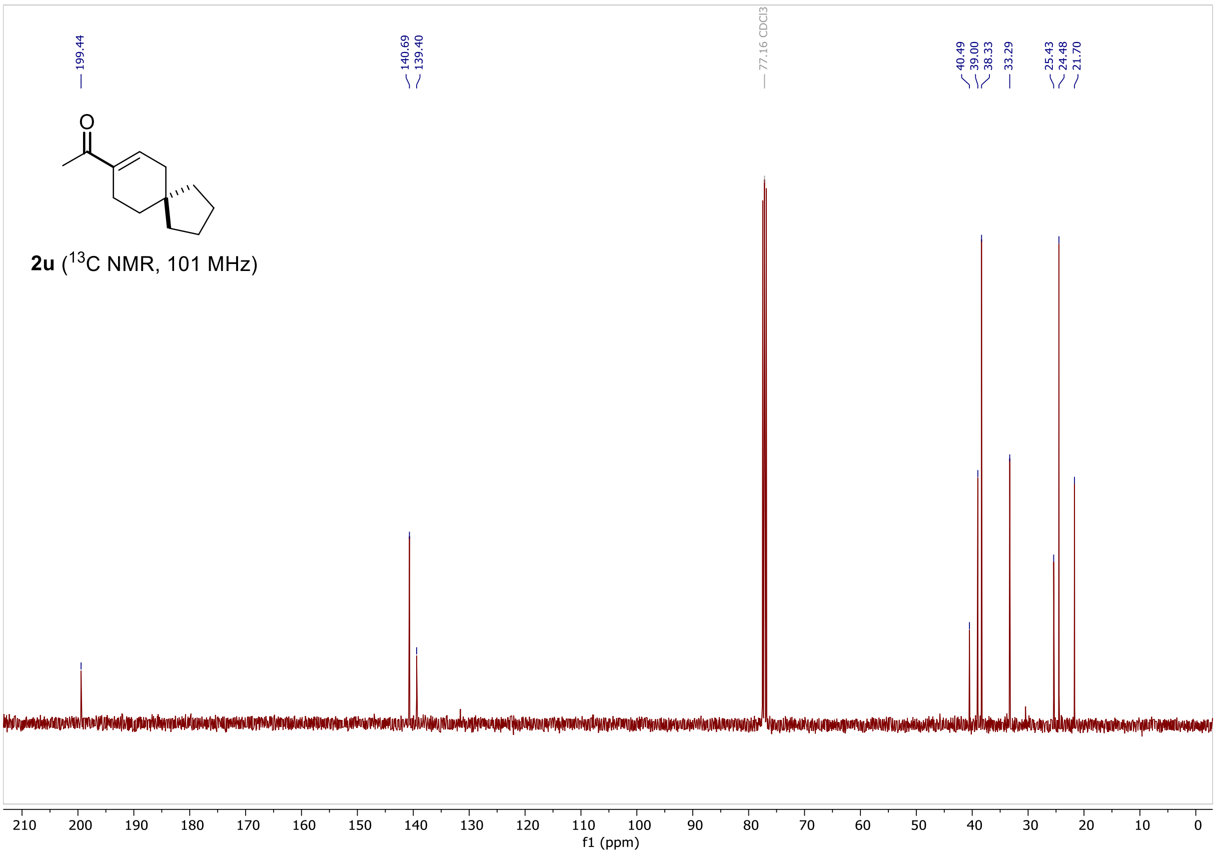

2u (^{13}C NMR, 101 MHz)

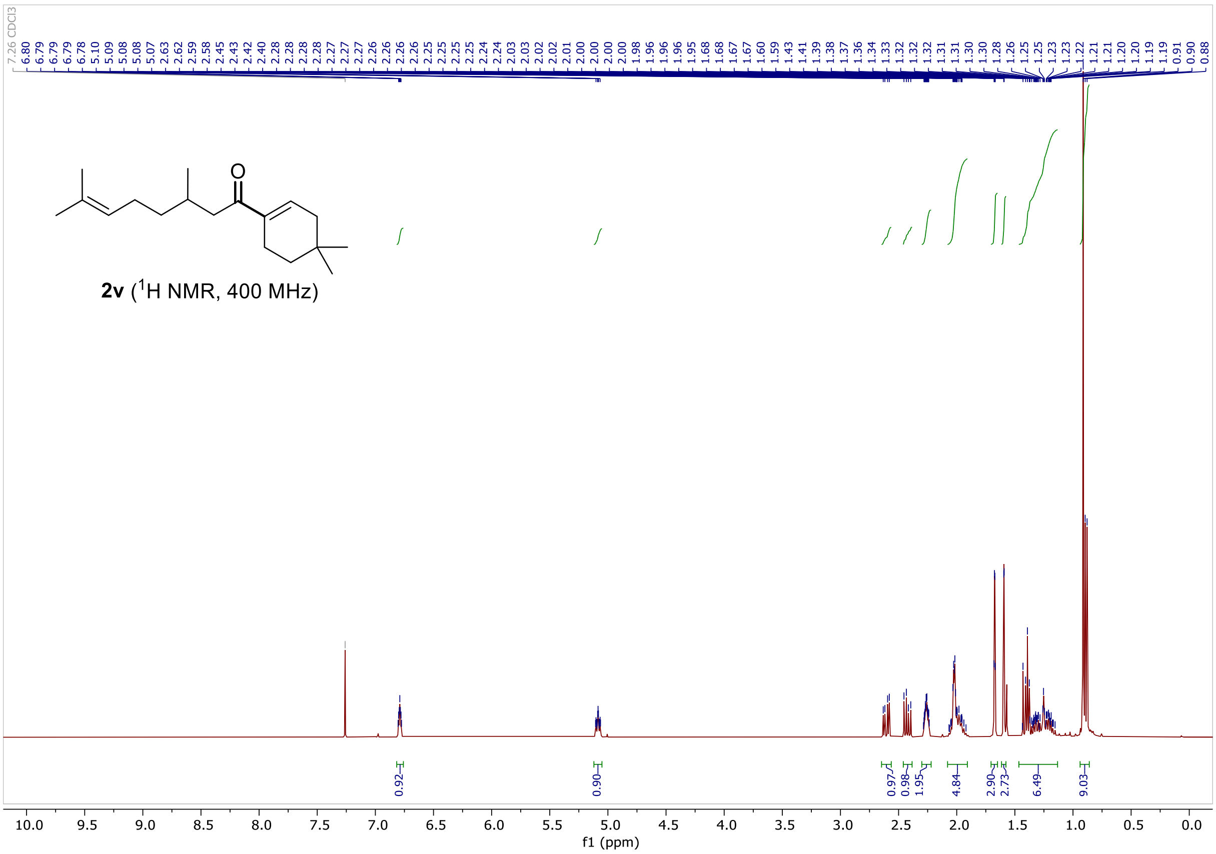
2v (1H NMR, 400 MHz)
f1 (ppm)

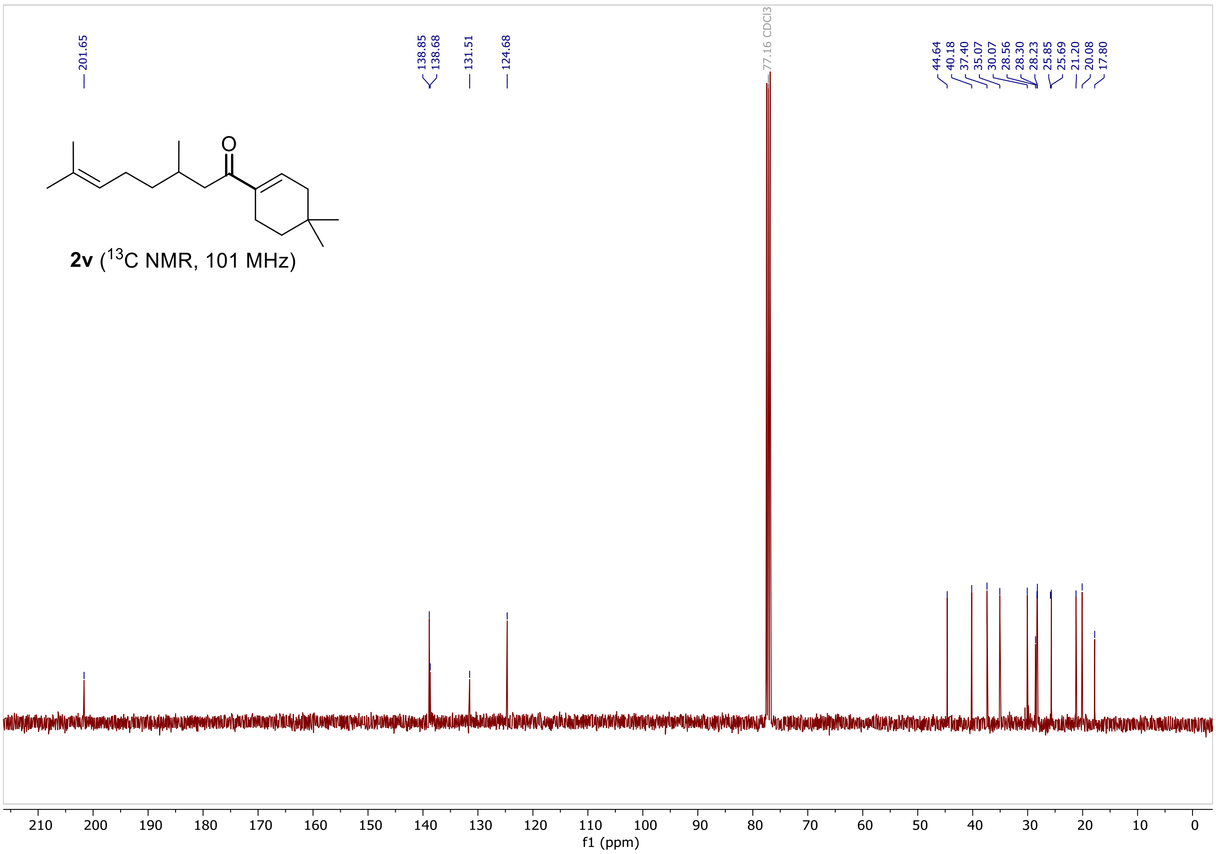
201.65
138.85
138.68
131.51
124.68
77.16 CDCl3
44.64
40.18
37.40
35.07
30.07
28.56
28.30
28.23
25.85
25.69
21.20
20.08
17.80
2v (13C NMR, 101 MHz)
f1 (ppm)

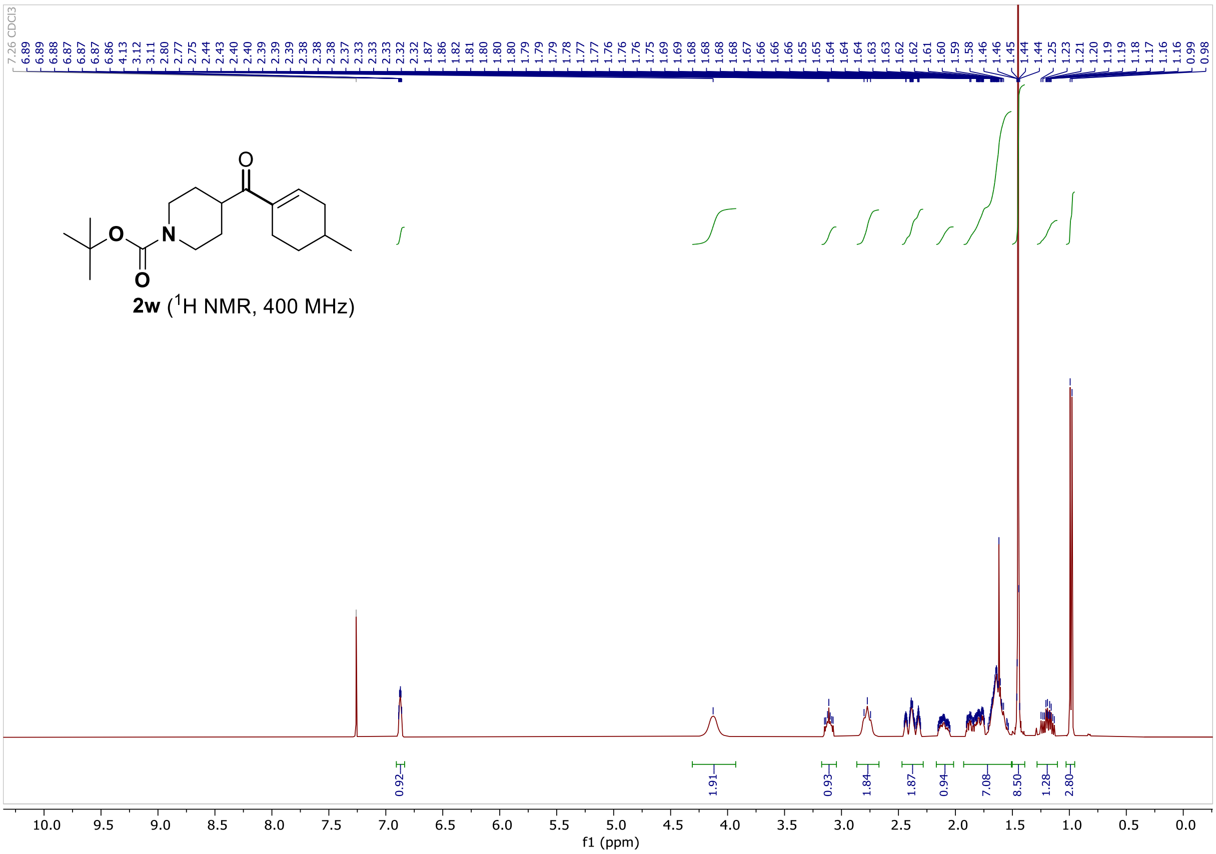
2w (1H NMR, 400 MHz)

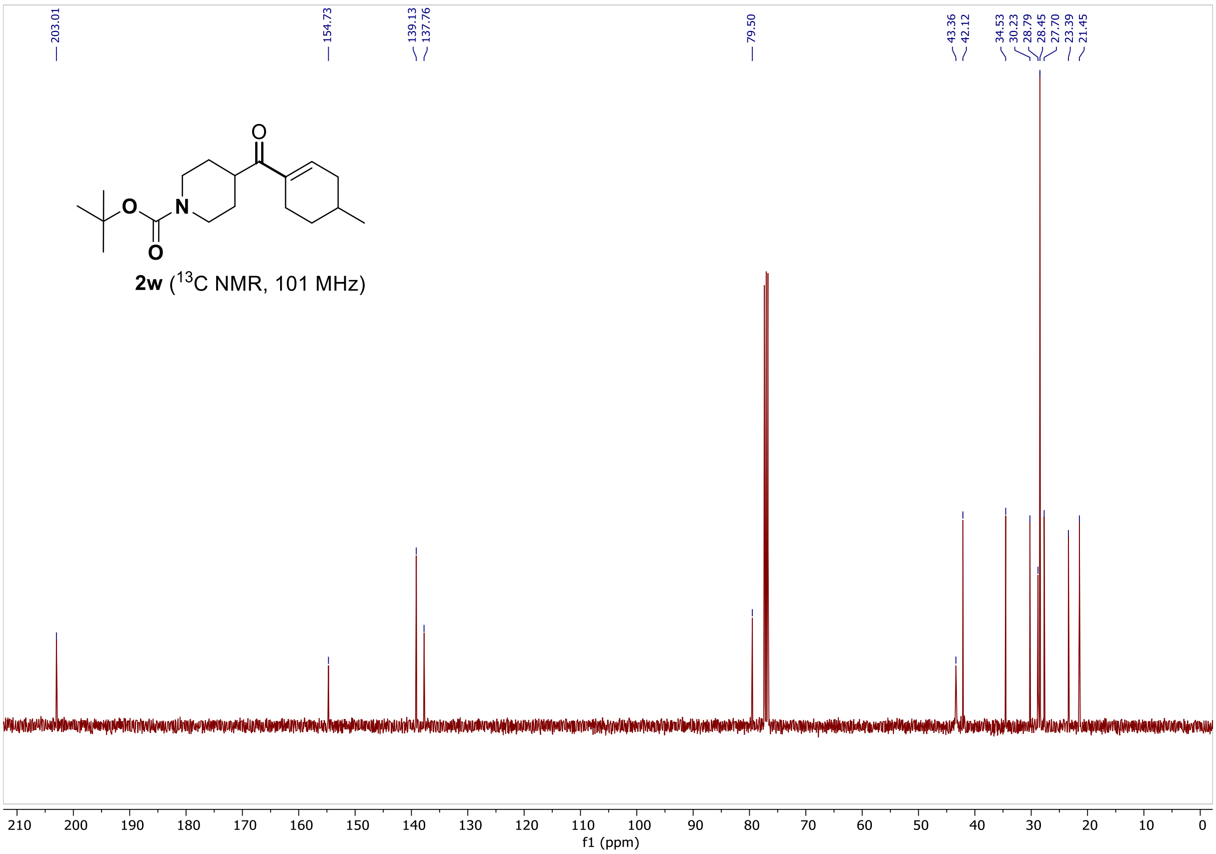
2w (13C NMR, 101 MHz)

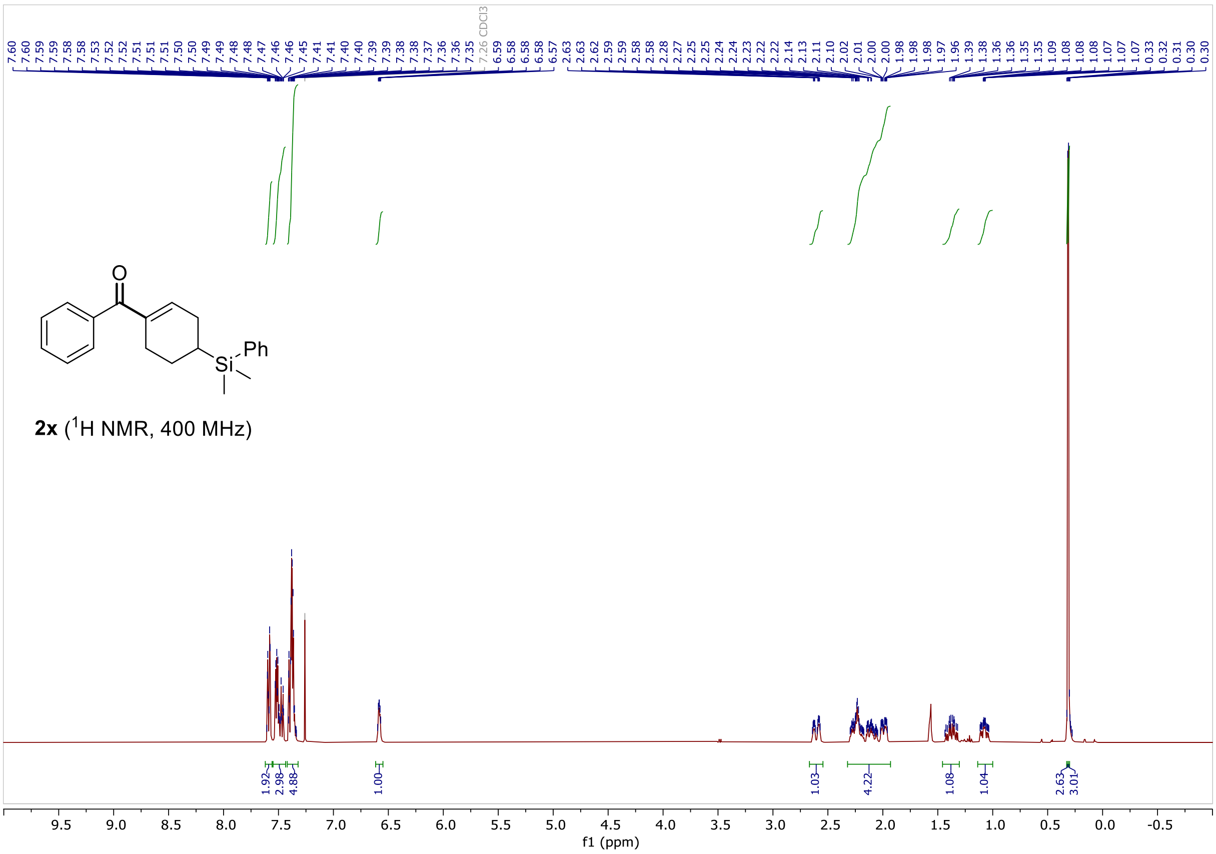
2x (1H NMR, 400 MHz)
f1 (ppm)

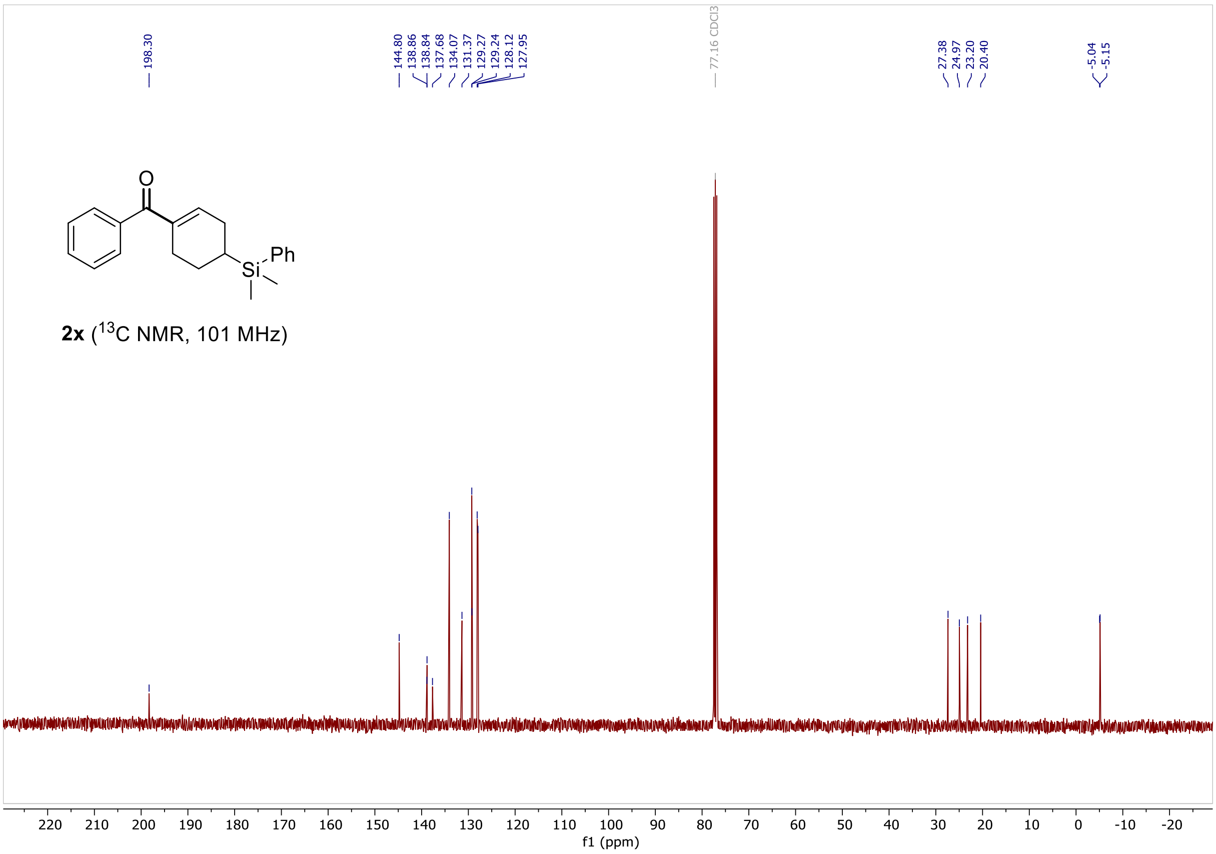
2x (13C NMR, 101 MHz)
198.30
144.80
138.86
138.84
137.68
134.07
131.37
129.27
129.24
128.12
127.95
77.16 CDCl3
27.38
24.97
23.20
20.40
-5.04
-5.15
f1 (ppm)

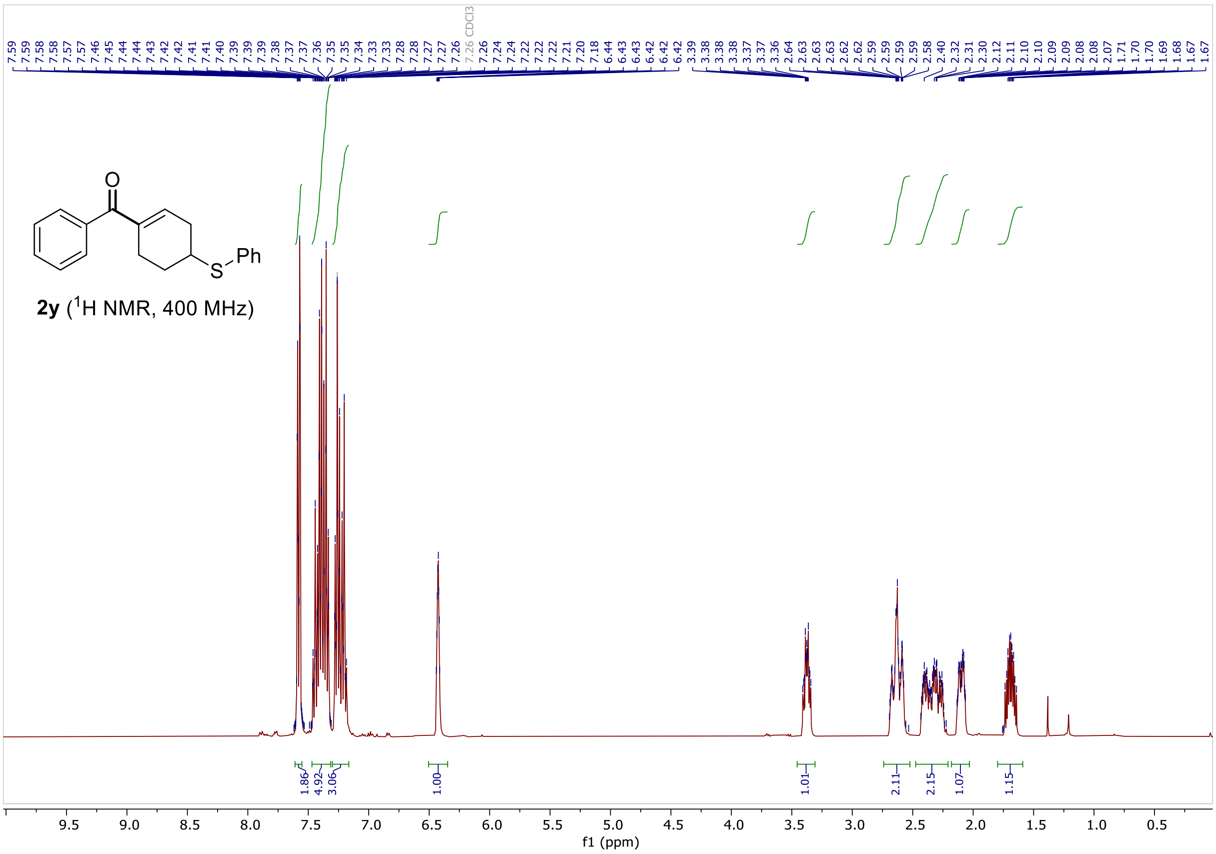
2y (1H NMR, 400 MHz)
Ph
S
O
1.86
4.92
3.06
1.00
1.01
2.11
2.15
1.07
1.15
f1 (ppm)

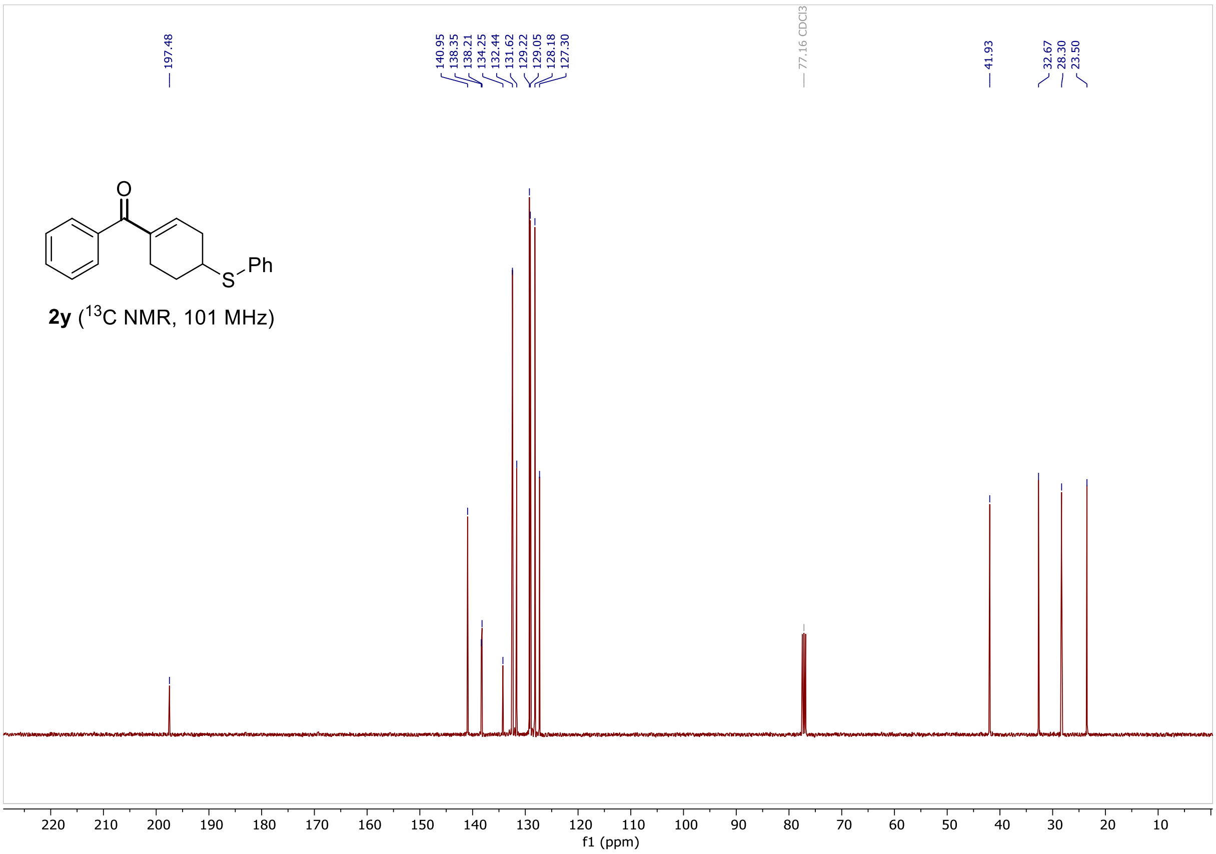
2y (13C NMR, 101 MHz)
Ph
S
O
197.48
140.95
138.35
138.21
134.25
132.44
131.62
129.22
129.05
128.18
127.30
77.16 CDCl3
41.93
32.67
28.30
23.50
f1 (ppm)

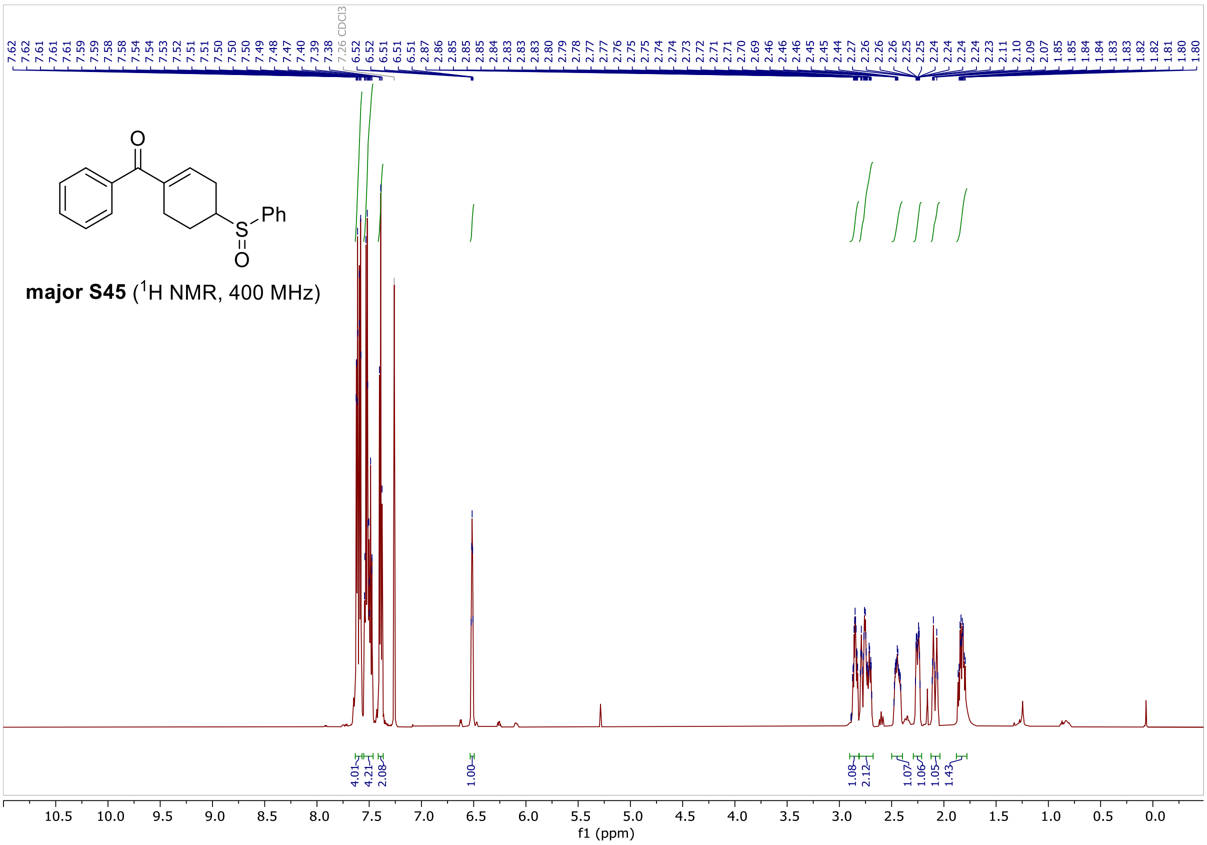

major S45 (1H NMR, 400 MHz)
O
S
Ph
O
7.62 7.62 7.61 7.61 7.59 7.59 7.58 7.58 7.54 7.54 7.53 7.52 7.51 7.51 7.50 7.50 7.49 7.48 7.47 7.40 7.39 7.38
7.26 CDCl3
6.52 6.52 6.51 6.51 6.51 2.87 2.86 2.85 2.85 2.85 2.84 2.83 2.83 2.83 2.80 2.79 2.78 2.77 2.76 2.75 2.75 2.74 2.74 2.73 2.72 2.71 2.70 2.69 2.46 2.46 2.46 2.45 2.44 2.27 2.26 2.26 2.26 2.25 2.25 2.24 2.24 2.24 2.24 2.23 2.11 2.10 2.09 2.07 1.85 1.85 1.84 1.84 1.83 1.83 1.82 1.82 1.81 1.80 1.80
4.01 4.21 2.08 1.00 1.08 2.12 1.07 1.06 1.05 1.43
10.5 10.0 9.5 9.0 8.5 8.0 7.5 7.0 6.5 6.0 5.5 5.0 4.5 4.0 3.5 3.0 2.5 2.0 1.5 1.0 0.5 0.0
f1 (ppm)

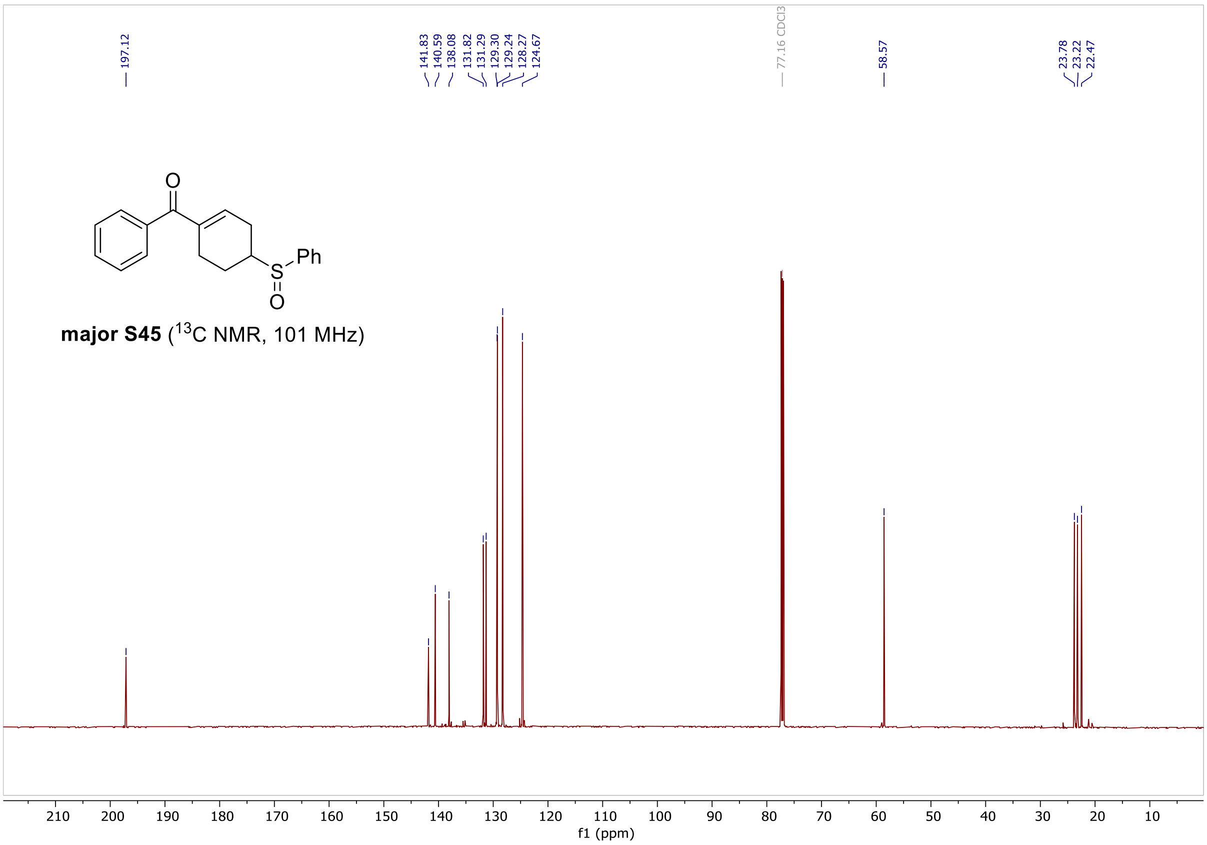

major S45 (13C NMR, 101 MHz)
O
S
Ph
O
197.12
141.83 140.59 138.08 131.82 131.29 129.30 129.24 128.27 124.67
77.16 CDCl3
58.57
23.78 23.22 22.47
210 200 190 180 170 160 150 140 130 120 110 100 90 80 70 60 50 40 30 20 10
f1 (ppm)

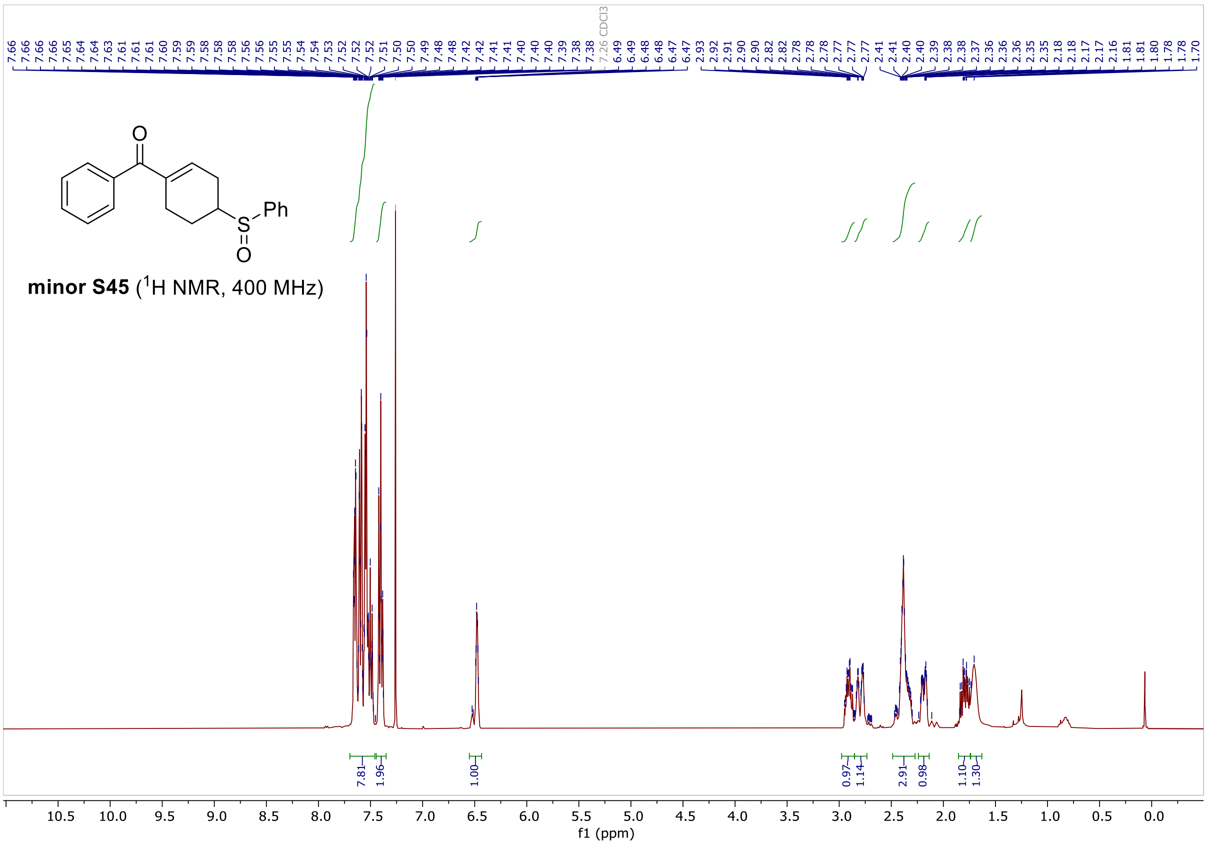
minor S45 (1H NMR, 400 MHz)
f1 (ppm)

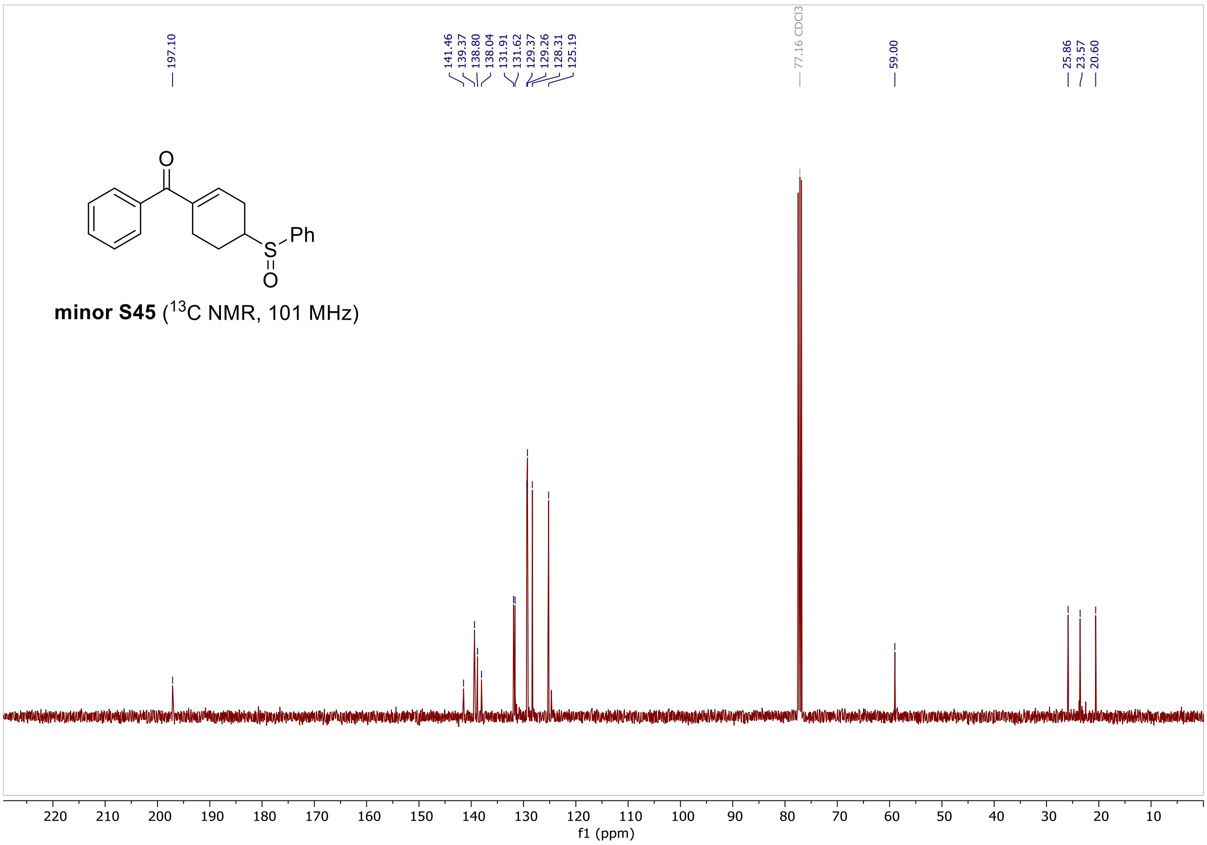
minor S45 (13C NMR, 101 MHz)
f1 (ppm)

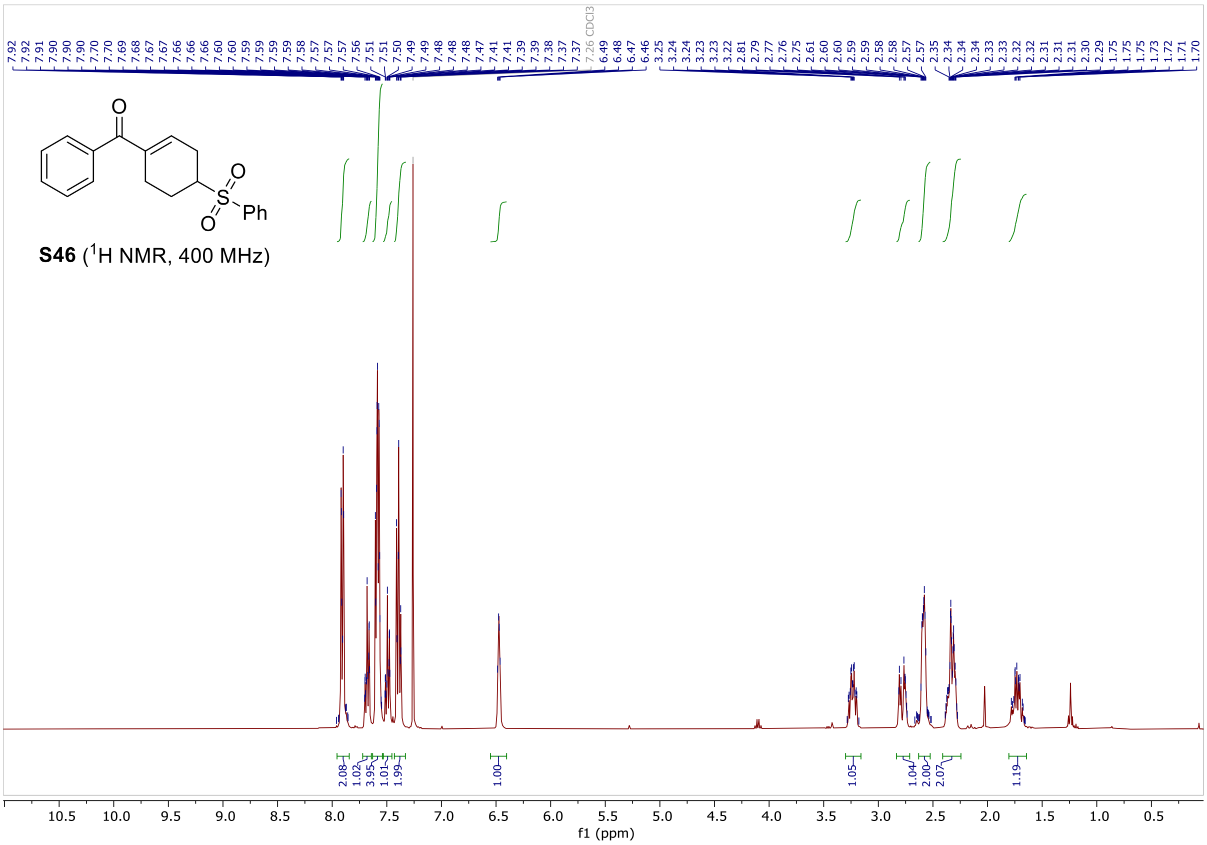
7.92 7.92 7.91 7.90 7.90 7.70 7.69 7.68 7.67 7.66 7.66 7.66 7.60 7.60 7.59 7.59 7.59 7.59 7.58 7.57 7.57 7.57 7.56 7.51 7.51 7.50 7.49 7.49 7.48 7.48 7.47 7.41 7.41 7.39 7.39 7.38 7.37 7.37 7.26 CDCl3 6.49 6.48 6.47 6.46 3.25 3.24 3.24 3.23 3.23 3.22 2.81 2.79 2.77 2.76 2.75 2.61 2.60 2.60 2.59 2.59 2.58 2.58 2.57 2.57 2.35 2.34 2.34 2.34 2.33 2.33 2.32 2.32 2.31 2.31 2.31 2.30 2.29 1.75 1.75 1.75 1.73 1.72 1.71 1.70
O
S
O
O
Ph
S46 (1H NMR, 400 MHz)
2.08 1.02 3.95 1.01 1.99 1.00 1.05 1.04 2.00 2.07 1.19
10.5 10.0 9.5 9.0 8.5 8.0 7.5 7.0 6.5 6.0 5.5 5.0 4.5 4.0 3.5 3.0 2.5 2.0 1.5 1.0 0.5
f1 (ppm)

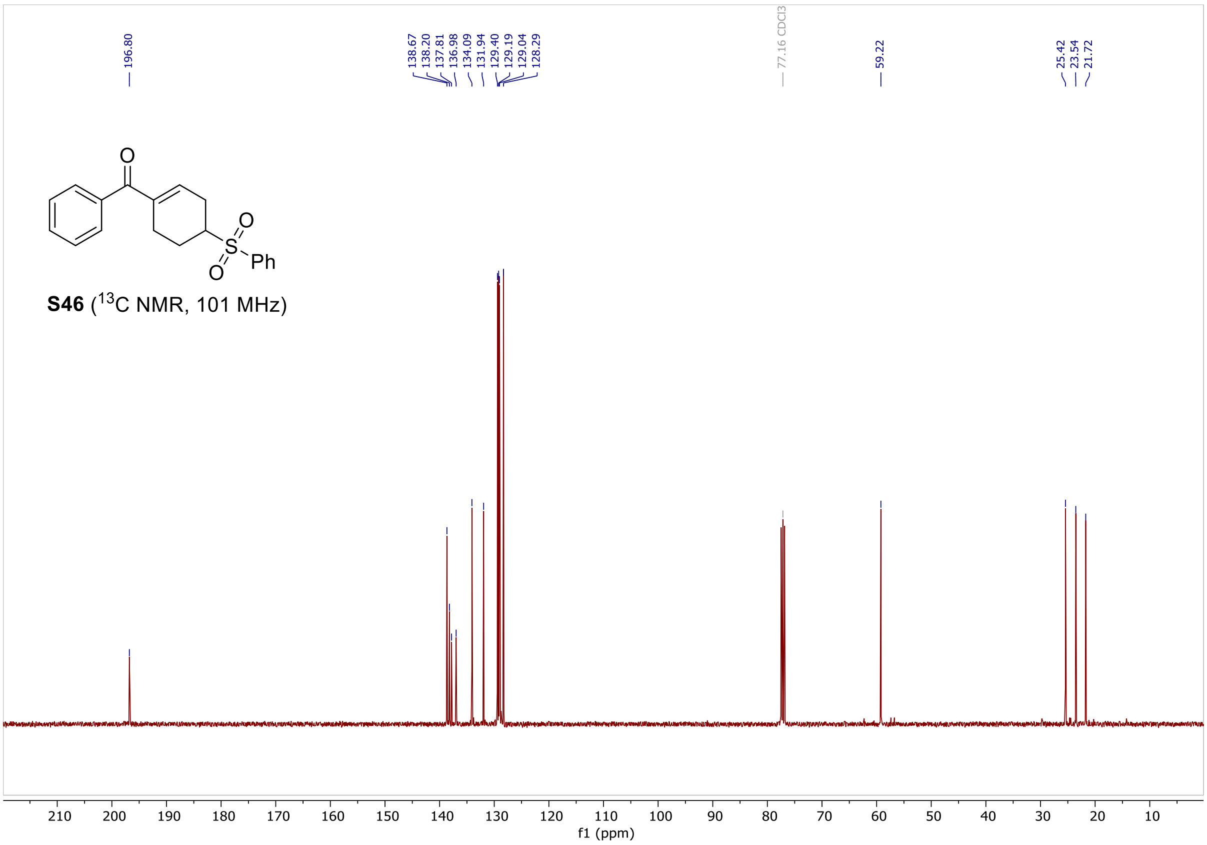
196.80
138.67 138.20 137.81 136.98 134.09 131.94 129.40 129.19 129.04 128.29
77.16 CDCl3
59.22
25.42 23.54 21.72
O
S
O
O
Ph
S46 (13C NMR, 101 MHz)
210 200 190 180 170 160 150 140 130 120 110 100 90 80 70 60 50 40 30 20 10
f1 (ppm)

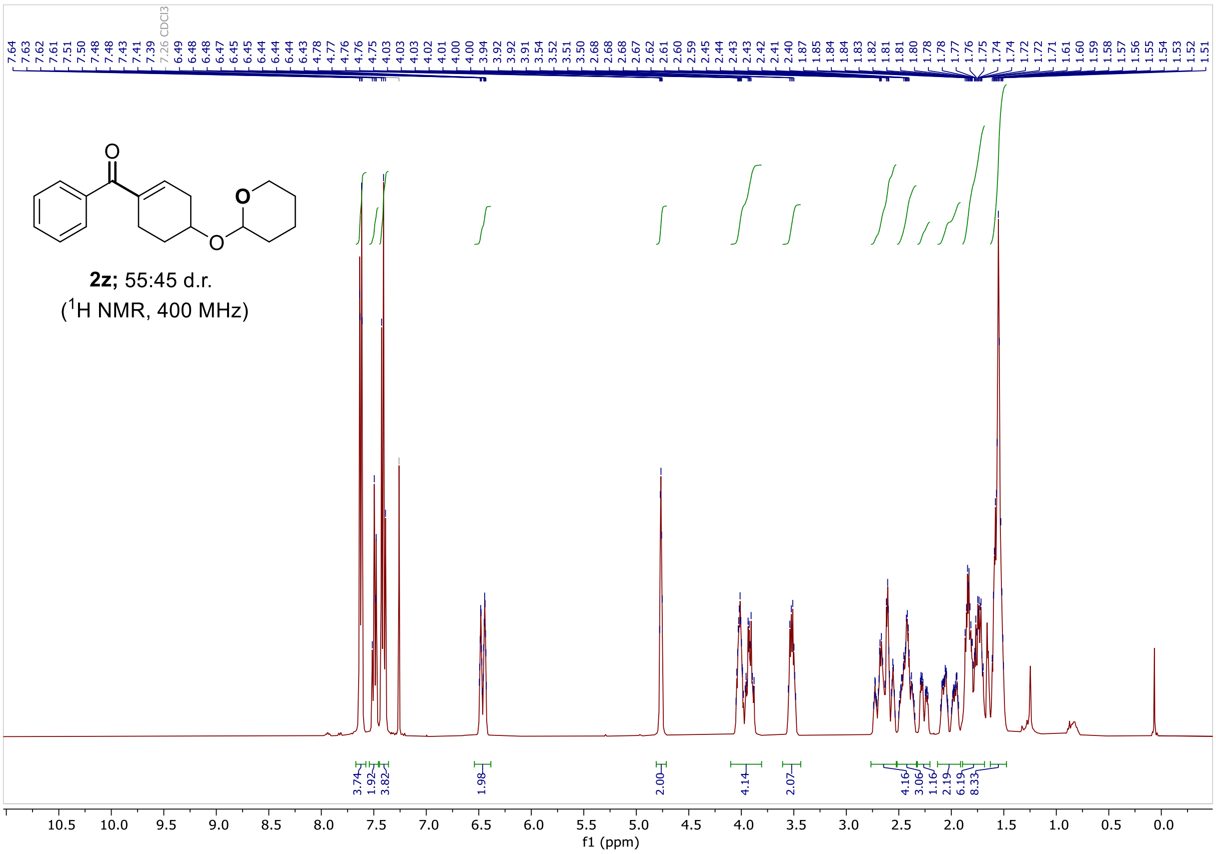

2z; 55:45 d.r.
(¹H NMR, 400 MHz)
f1 (ppm)

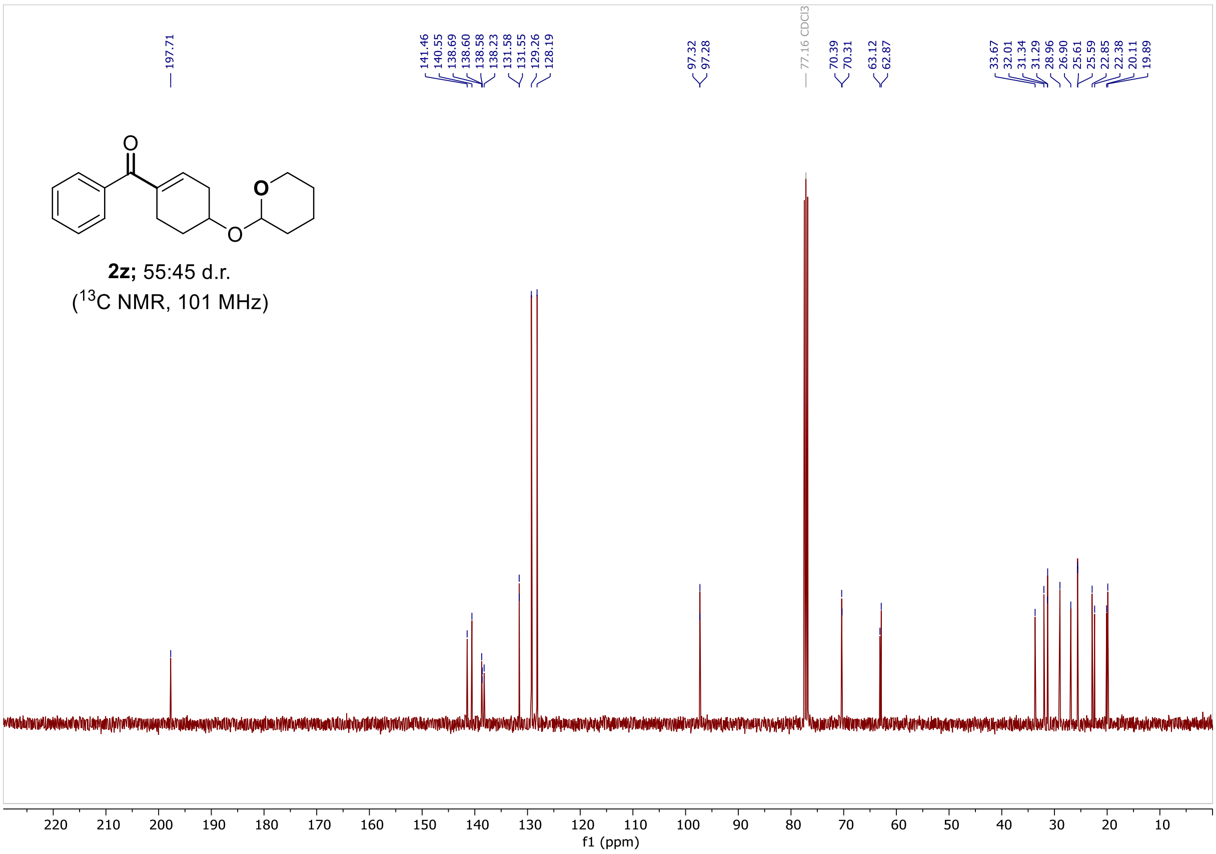

2z; 55:45 d.r.
(¹³C NMR, 101 MHz)
f1 (ppm)

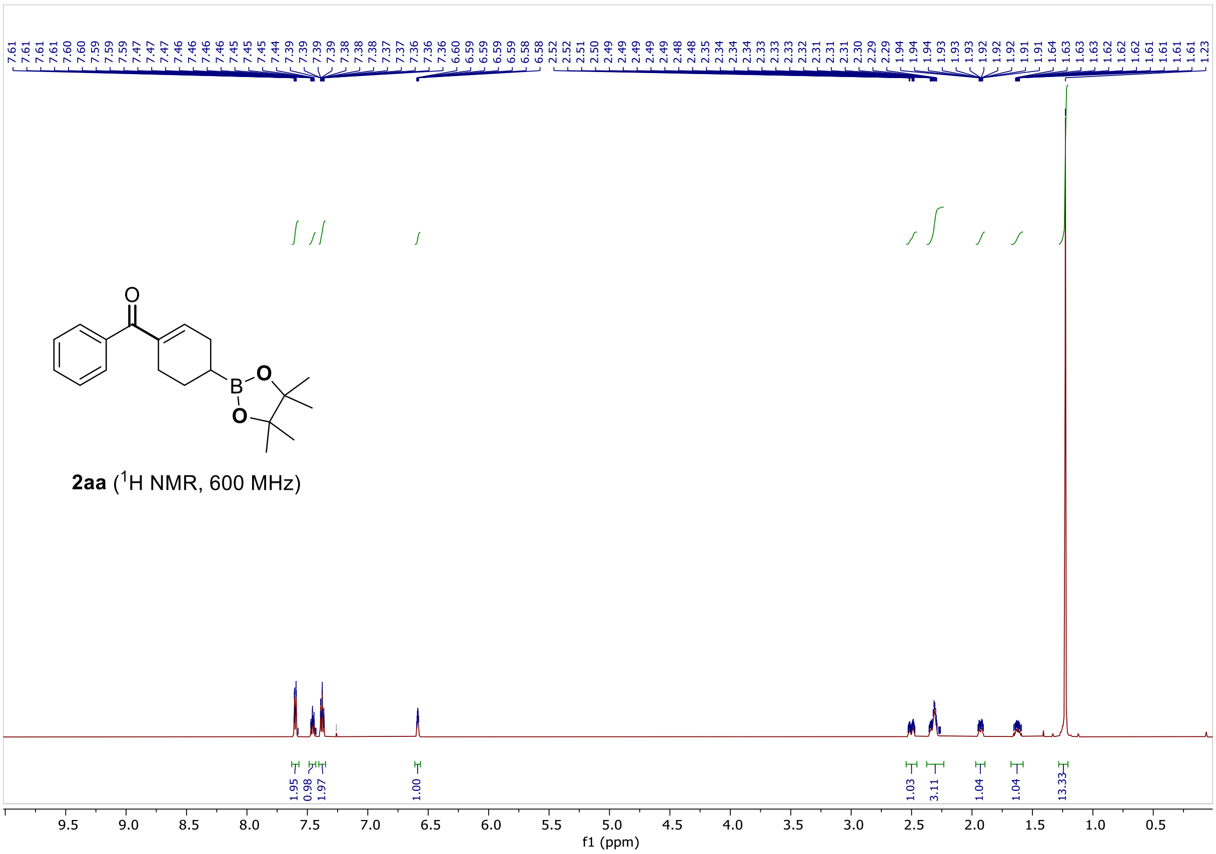
7.61 7.61 7.61 7.60 7.59 7.59 7.59 7.47 7.47 7.46 7.46 7.46 7.45 7.45 7.44 7.39 7.39 7.39 7.39 7.38 7.38 7.38 7.37 7.36 7.36 7.36 6.60 6.59 6.59 6.59 6.59 6.58 6.58 2.52 2.52 2.51 2.50 2.49 2.49 2.49 2.49 2.48 2.35 2.34 2.34 2.34 2.33 2.33 2.33 2.32 2.31 2.31 2.31 2.30 2.29 2.29 1.94 1.94 1.94 1.93 1.93 1.93 1.92 1.92 1.92 1.91 1.91 1.64 1.63 1.63 1.63 1.62 1.62 1.61 1.61 1.61 1.61 1.23
O
B
O
O
2aa (^{1}H NMR, 600 MHz)
1.95
0.98
1.97
1.00
1.03
3.11
1.04
1.04
13.33
9.5 9.0 8.5 8.0 7.5 7.0 6.5 6.0 5.5 5.0 4.5 4.0 3.5 3.0 2.5 2.0 1.5 1.0 0.5
f1 (ppm)

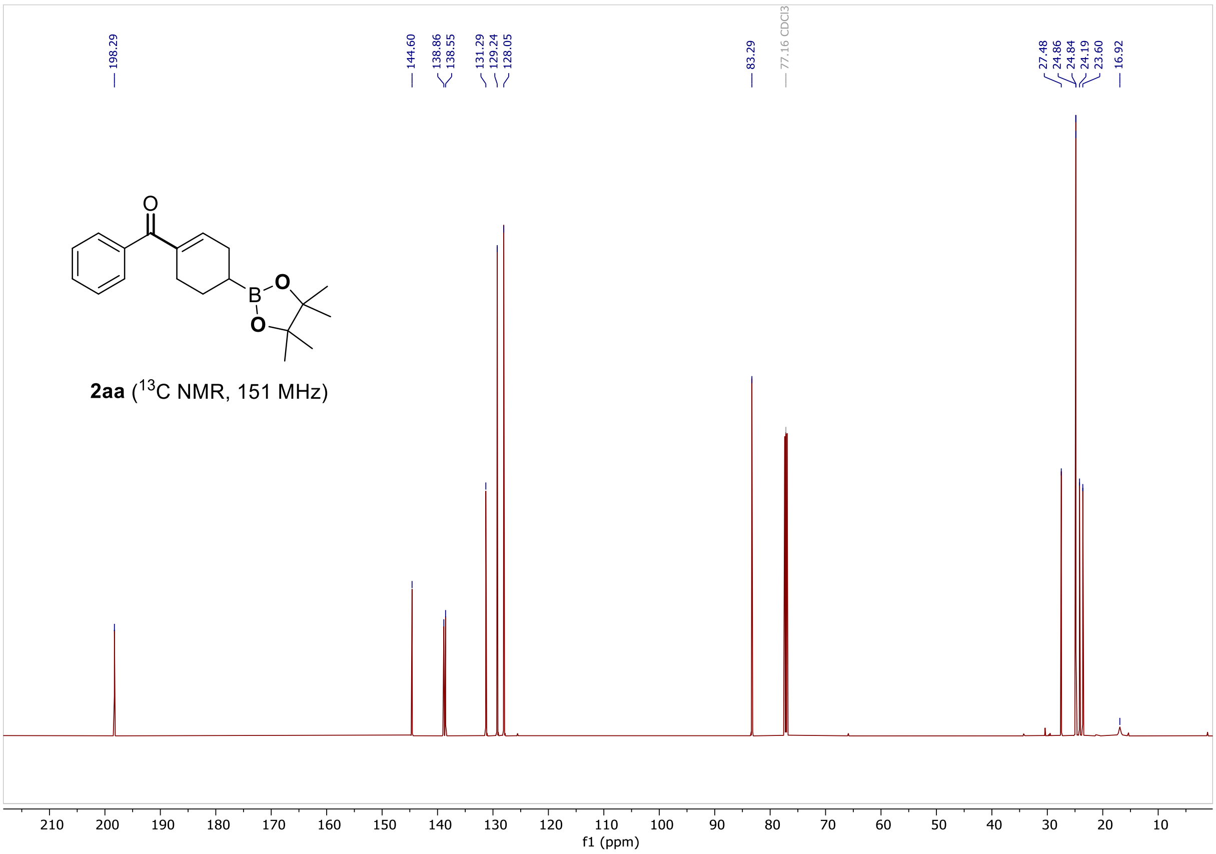
198.29
144.60
138.86
138.55
131.29
129.24
128.05
83.29
77.16 CDCl3
27.48
24.86
24.84
24.19
23.60
16.92
O
B
O
O
2aa (^{13}C NMR, 151 MHz)
210 200 190 180 170 160 150 140 130 120 110 100 90 80 70 60 50 40 30 20 10
f1 (ppm)

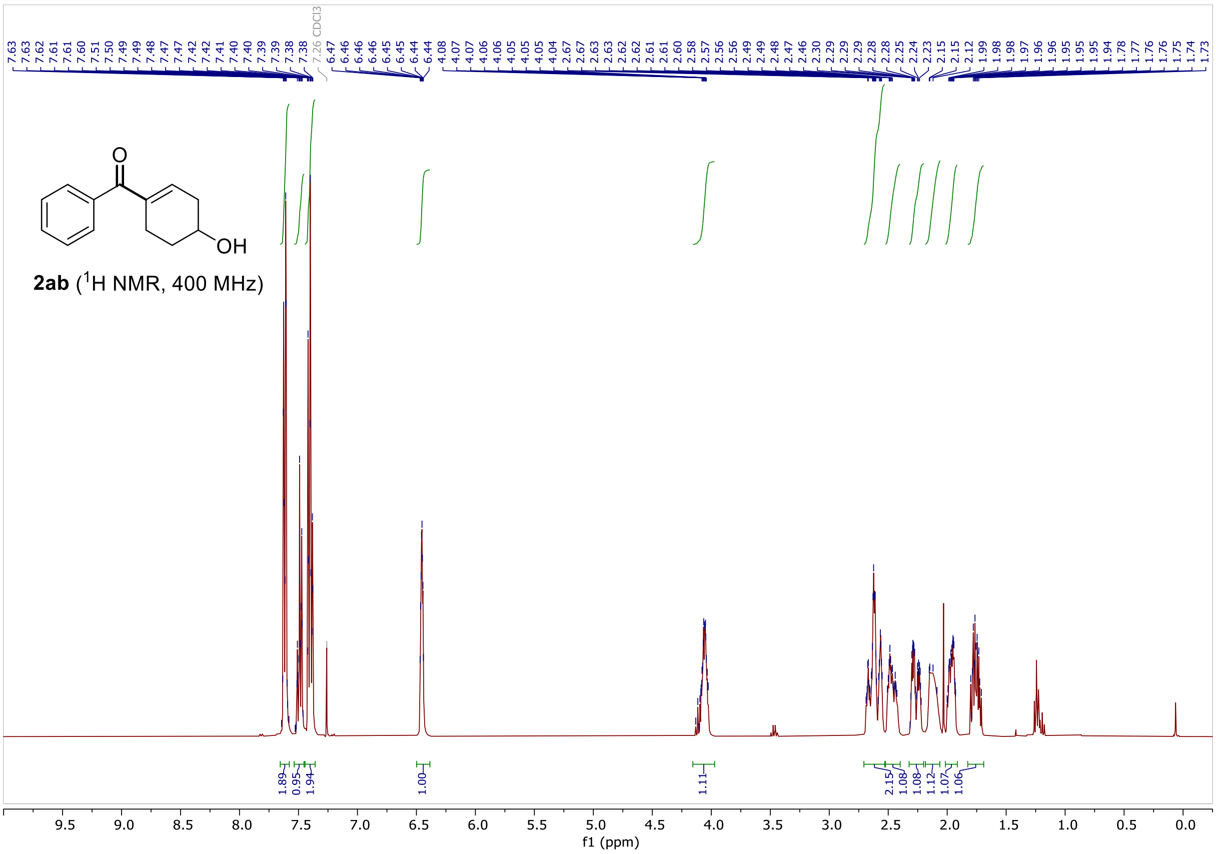

2ab (^{1}H NMR, 400 MHz)

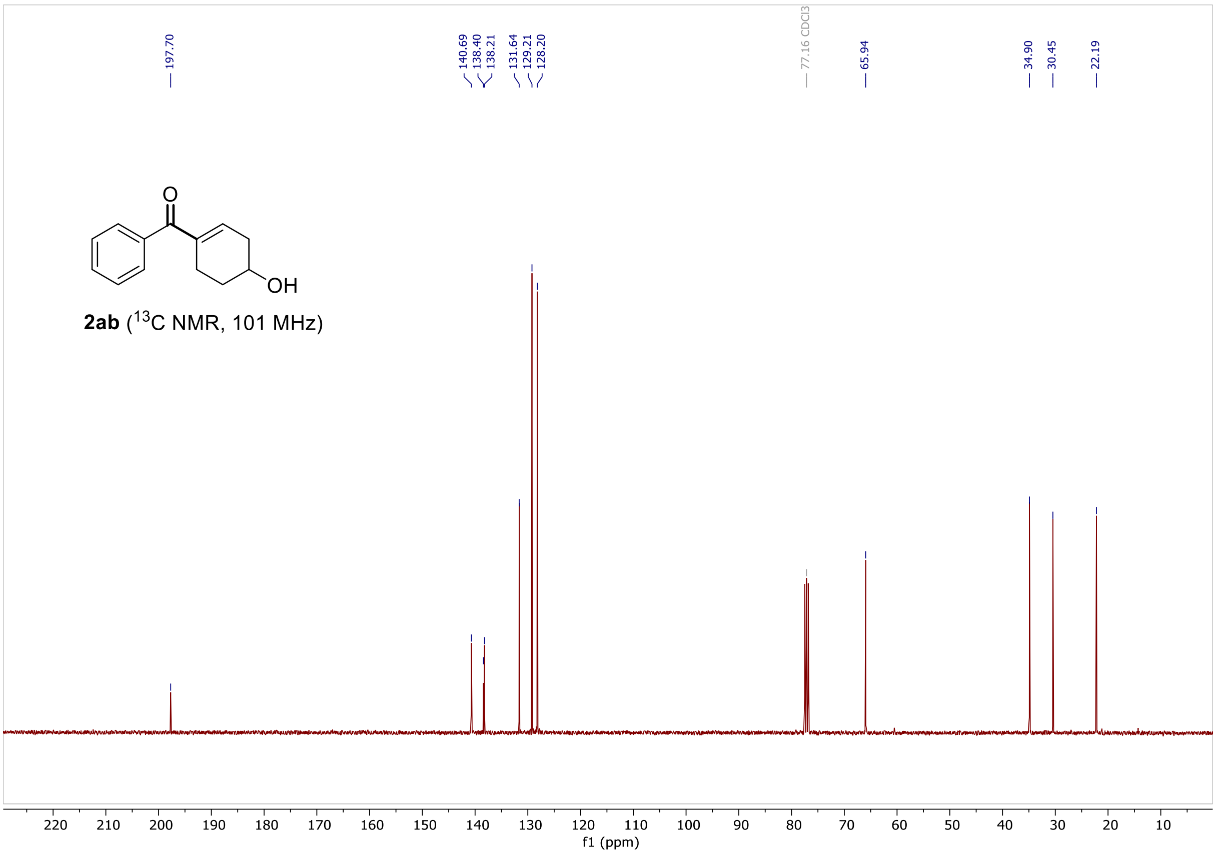

2ab (^{13}C NMR, 101 MHz)

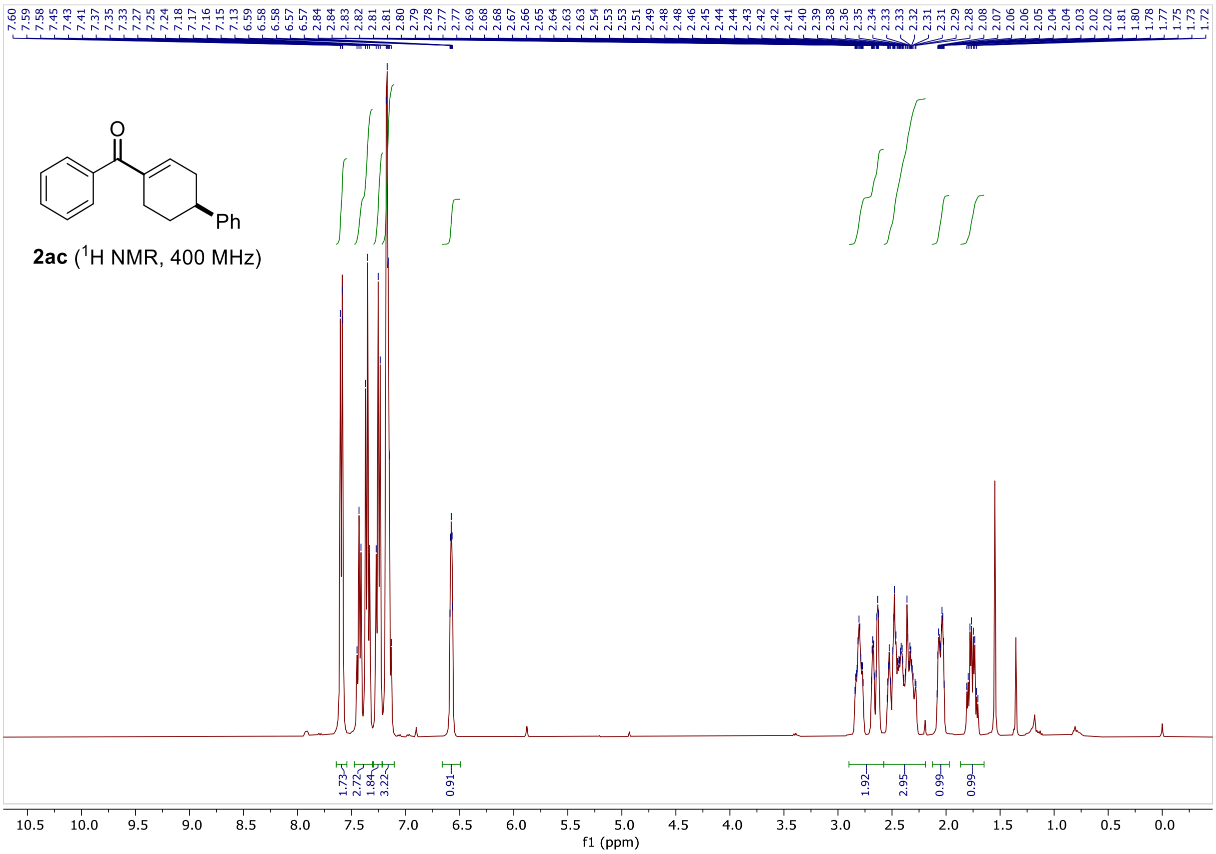
2ac (1H NMR, 400 MHz)
Ph
O
f1 (ppm)

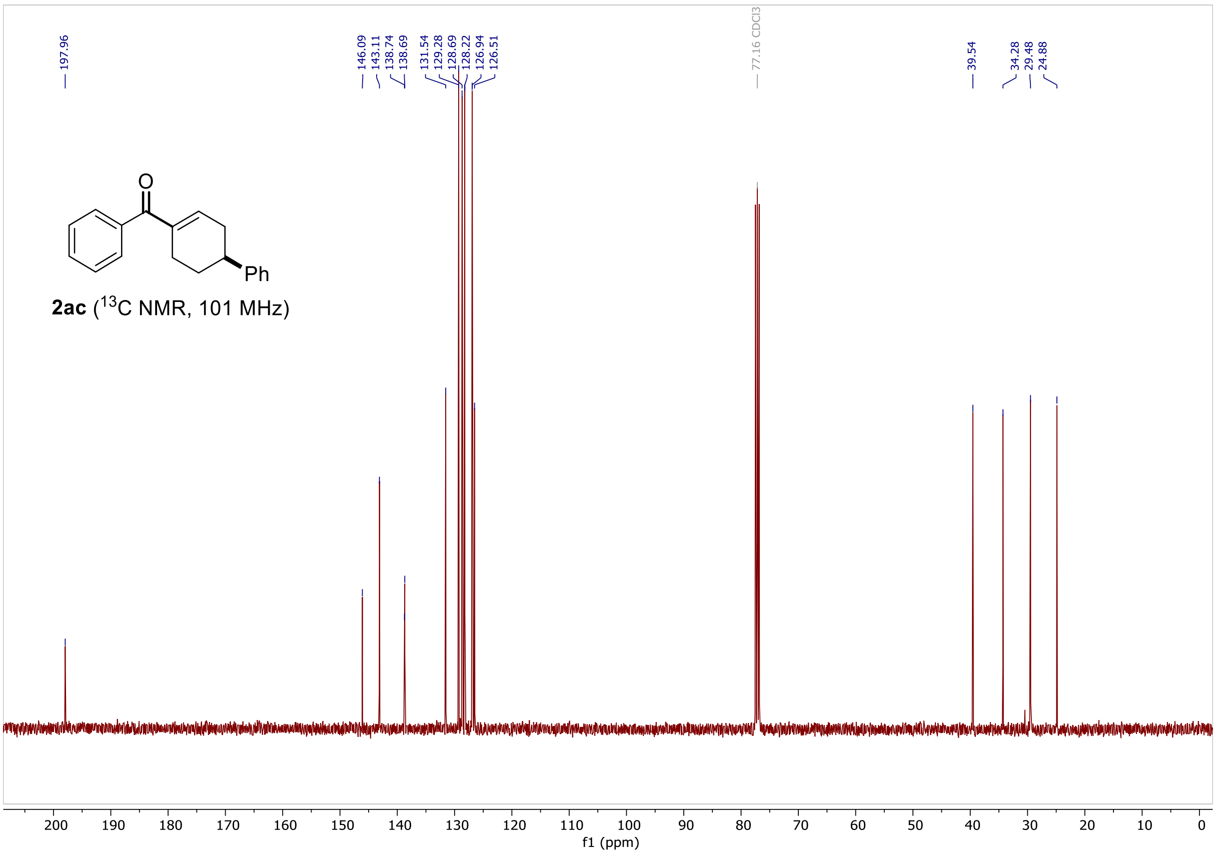
2ac (13C NMR, 101 MHz)
Ph
O
197.96
146.09
143.11
138.74
138.69
131.54
129.28
128.69
128.22
126.94
126.51
77.16 CDCl3
39.54
34.28
29.48
24.88
f1 (ppm)

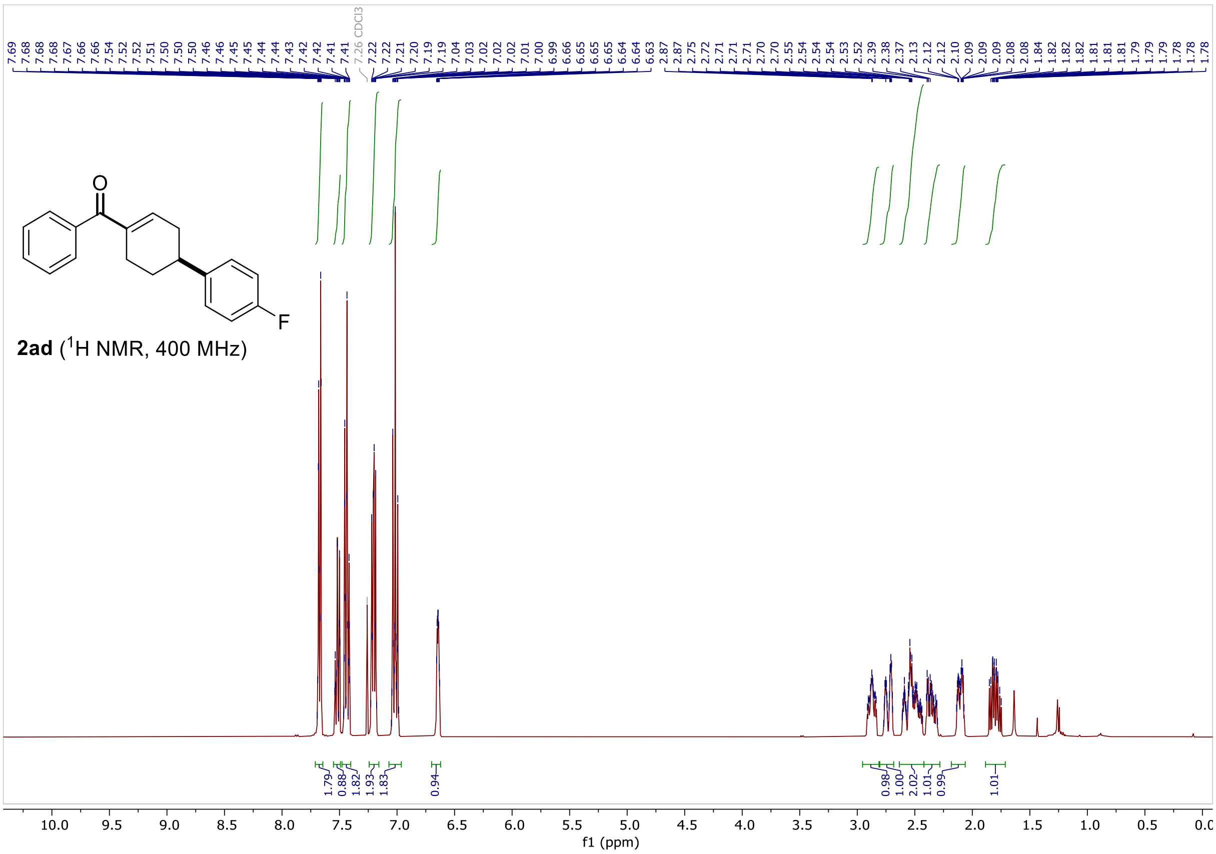
2ad (1H NMR, 400 MHz)
f1 (ppm)

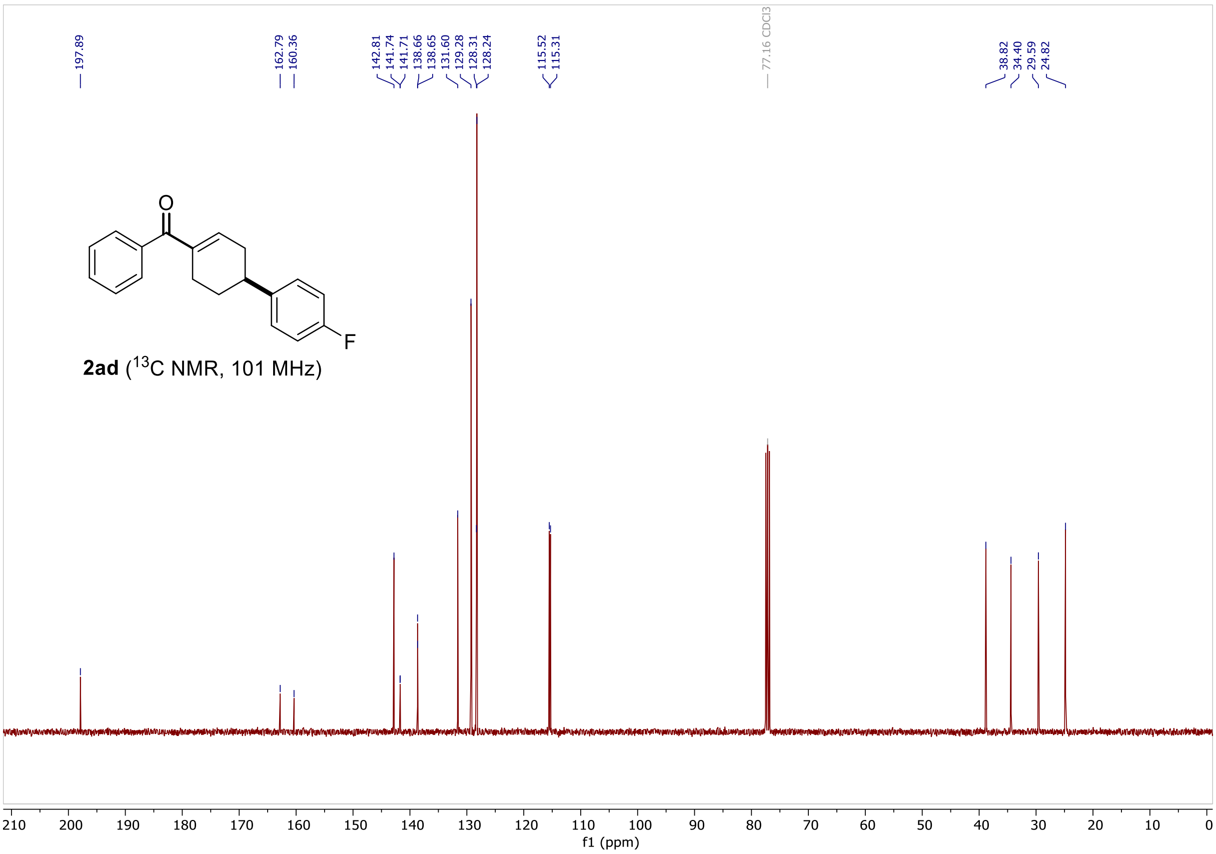
2ad (13C NMR, 101 MHz)
197.89
162.79
160.36
142.81
141.74
141.71
138.66
138.65
131.60
129.28
128.31
128.24
115.52
115.31
77.16 CDCl3
38.82
34.40
29.59
24.82
f1 (ppm)

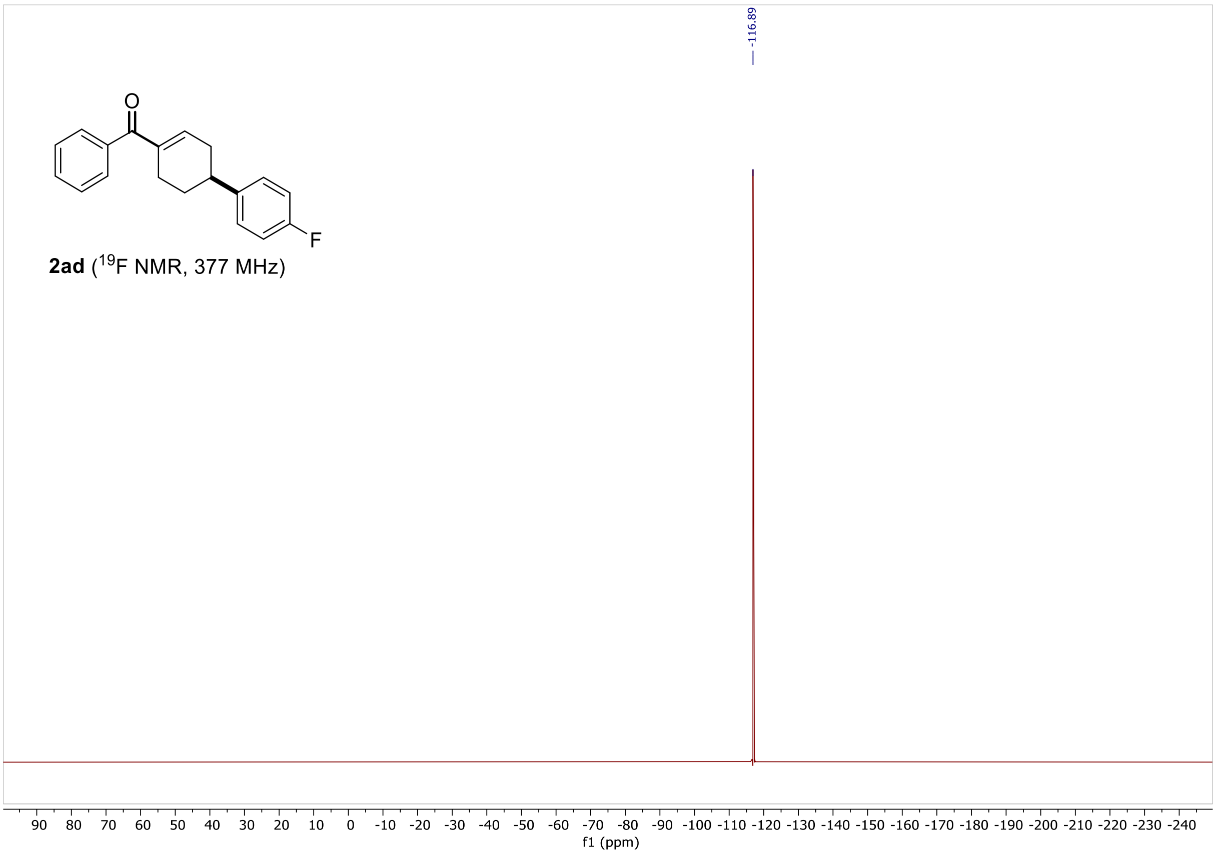
-116.89
2ad (19F NMR, 377 MHz)
90 80 70 60 50 40 30 20 10 0 -10 -20 -30 -40 -50 -60 -70 -80 -90 -100 -110 -120 -130 -140 -150 -160 -170 -180 -190 -200 -210 -220 -230 -240
f1 (ppm)

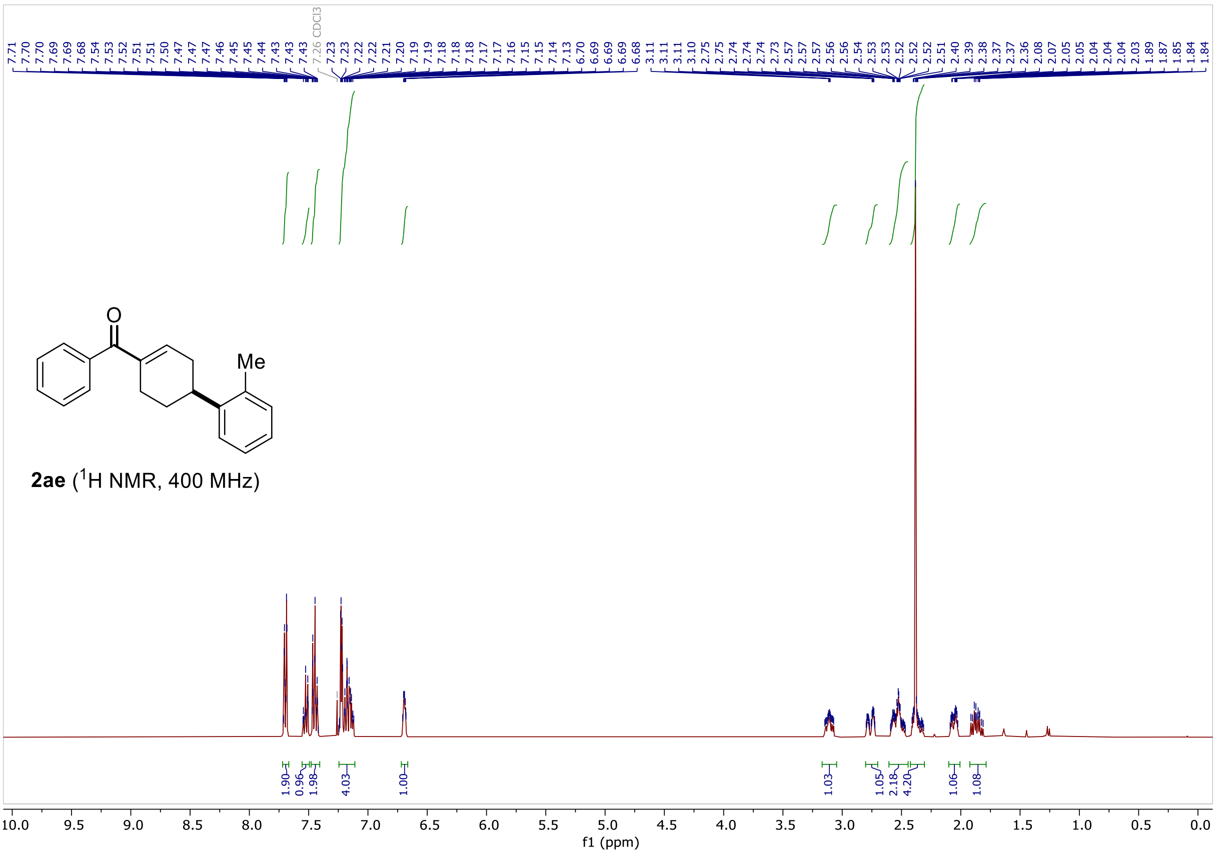
2ae (1H NMR, 400 MHz)
f1 (ppm)

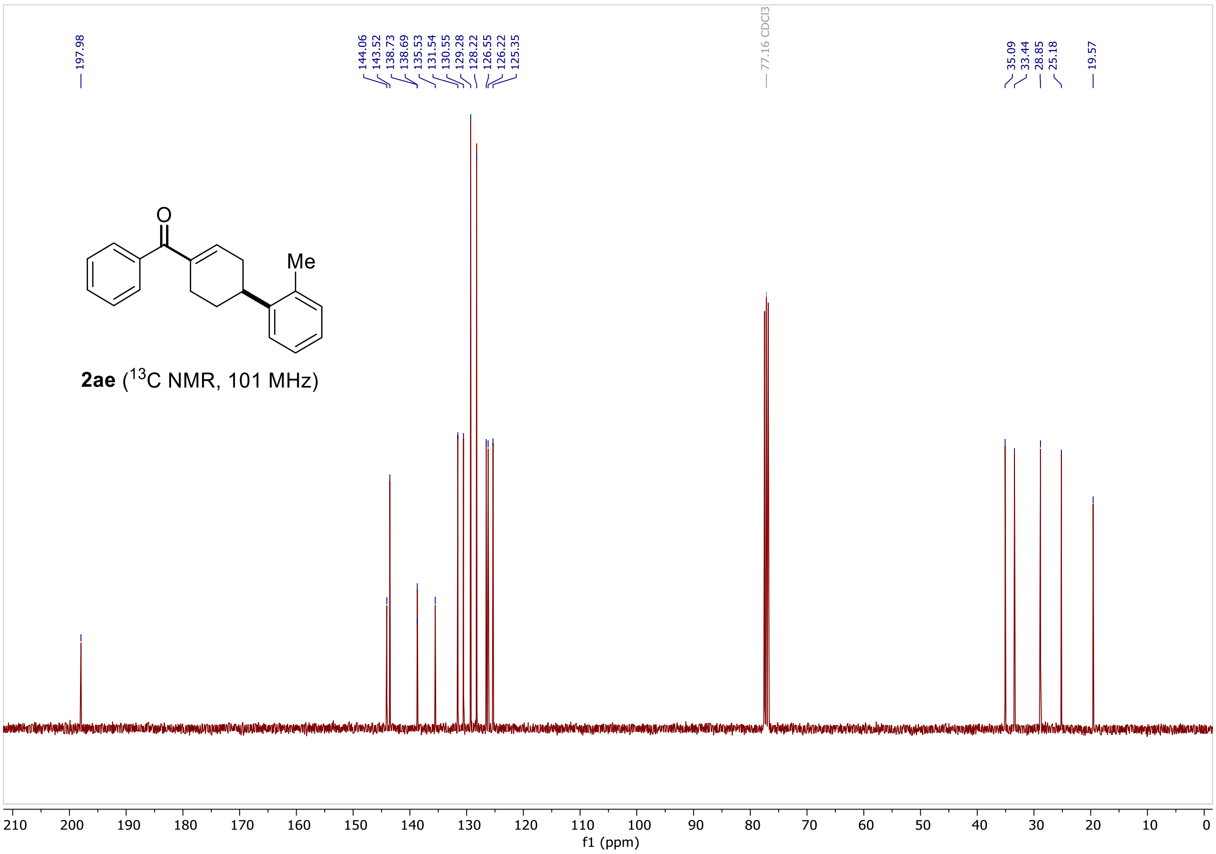
2ae (13C NMR, 101 MHz)
197.98
144.06
143.52
138.73
138.69
135.53
131.54
130.55
129.28
128.22
126.55
126.22
125.35
77.16 CDCl3
35.09
33.44
28.85
25.18
19.57
f1 (ppm)

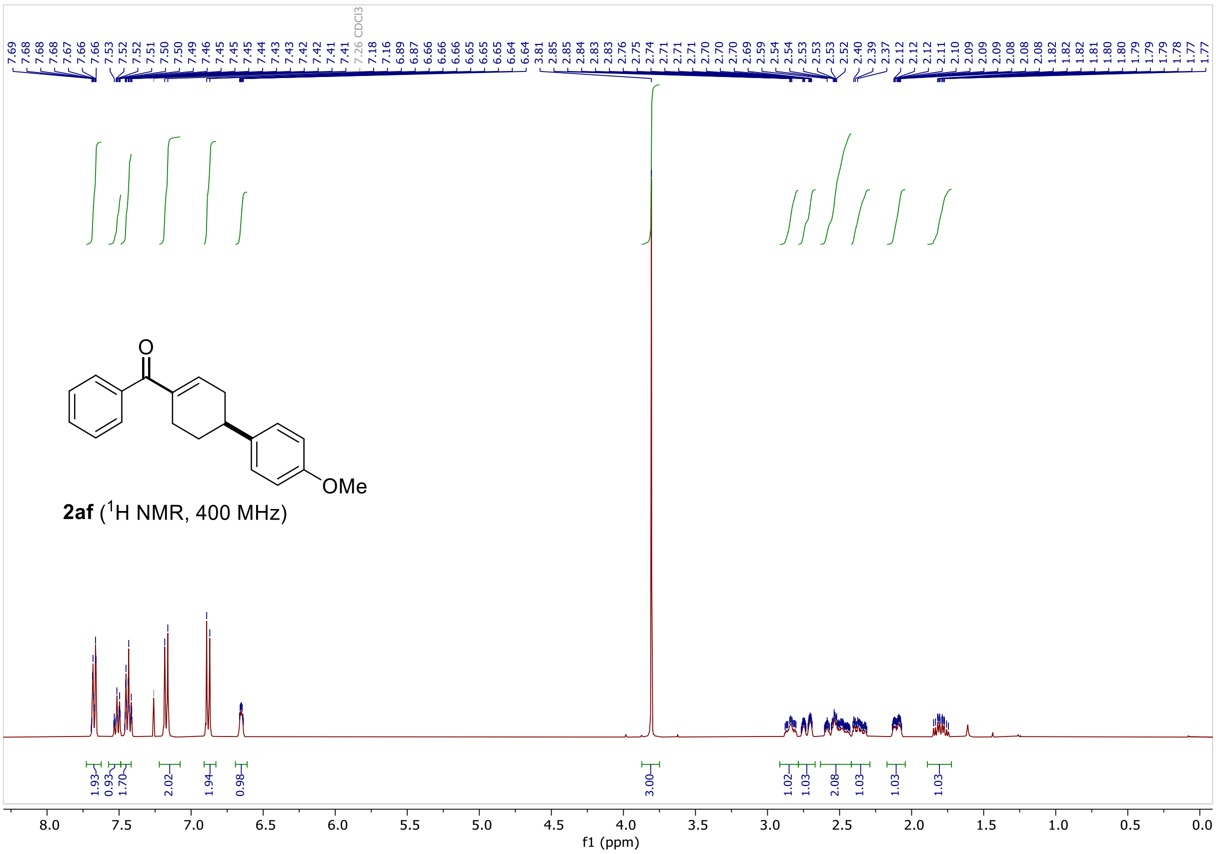
2af (1H NMR, 400 MHz)
OMe
f1 (ppm)

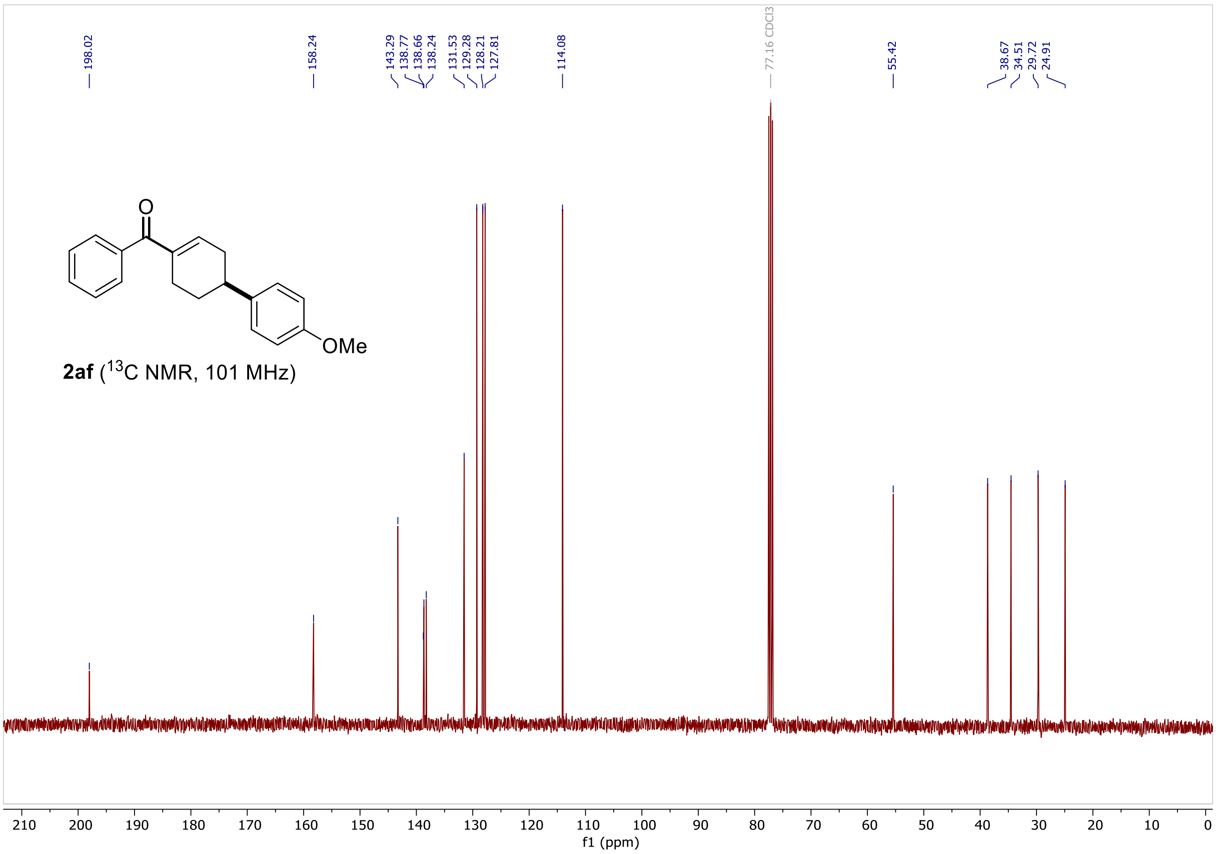
2af (13C NMR, 101 MHz)
OMe
198.02
158.24
143.29
138.77
138.66
138.24
131.53
129.28
128.21
127.81
114.08
77.16 CDCl3
55.42
38.67
34.51
29.72
24.91
f1 (ppm)

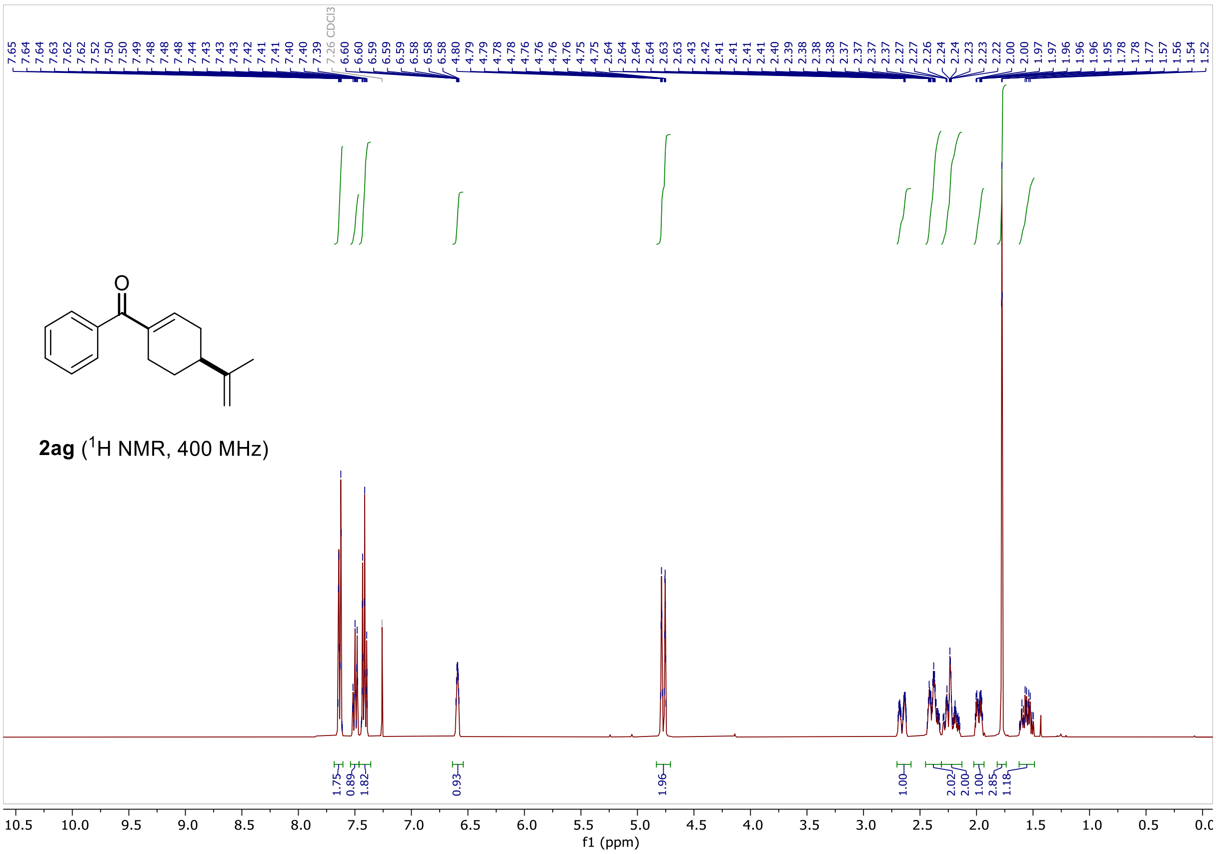
2ag (^{1}H NMR, 400 MHz)
f1 (ppm)

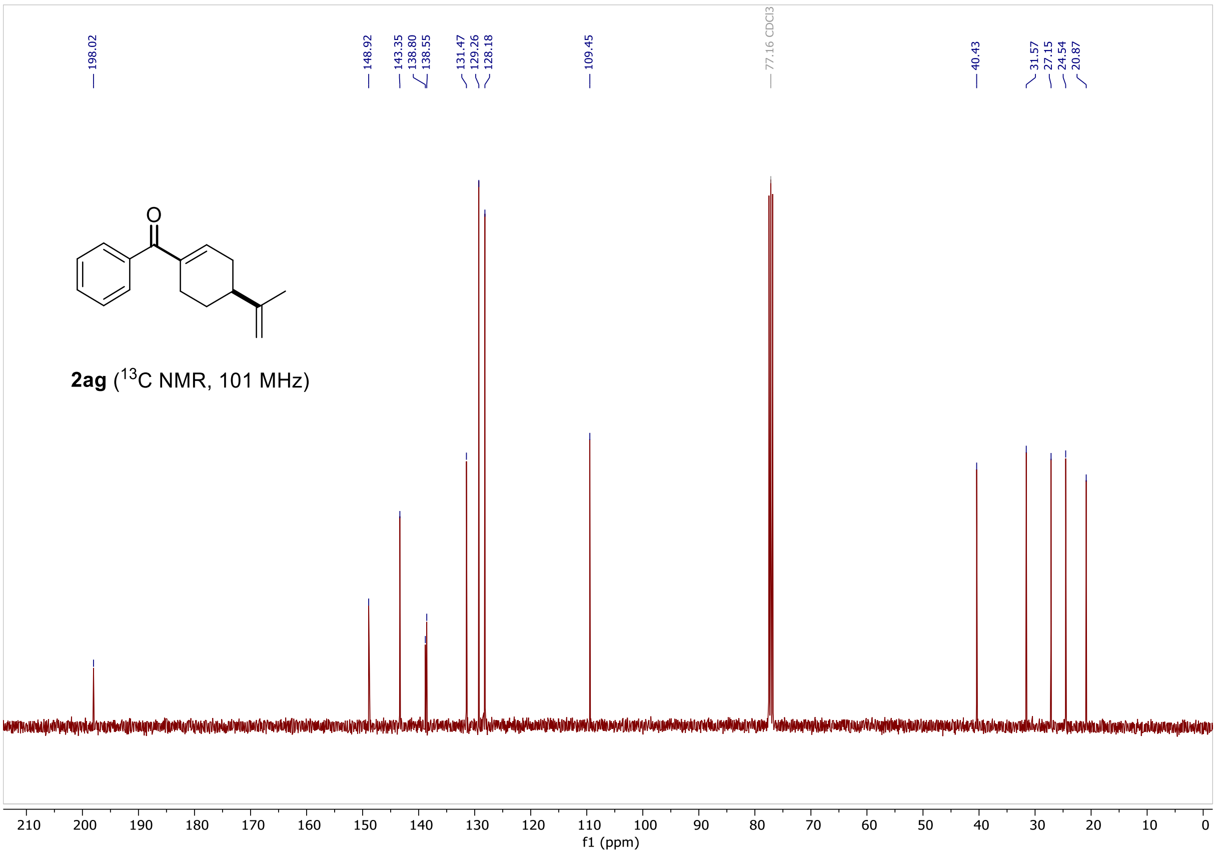
2ag (^{13}C NMR, 101 MHz)
198.02
148.92
143.35
138.80
138.55
131.47
129.26
128.18
109.45
77.16 CDCl3
40.43
31.57
27.15
24.54
20.87
f1 (ppm)

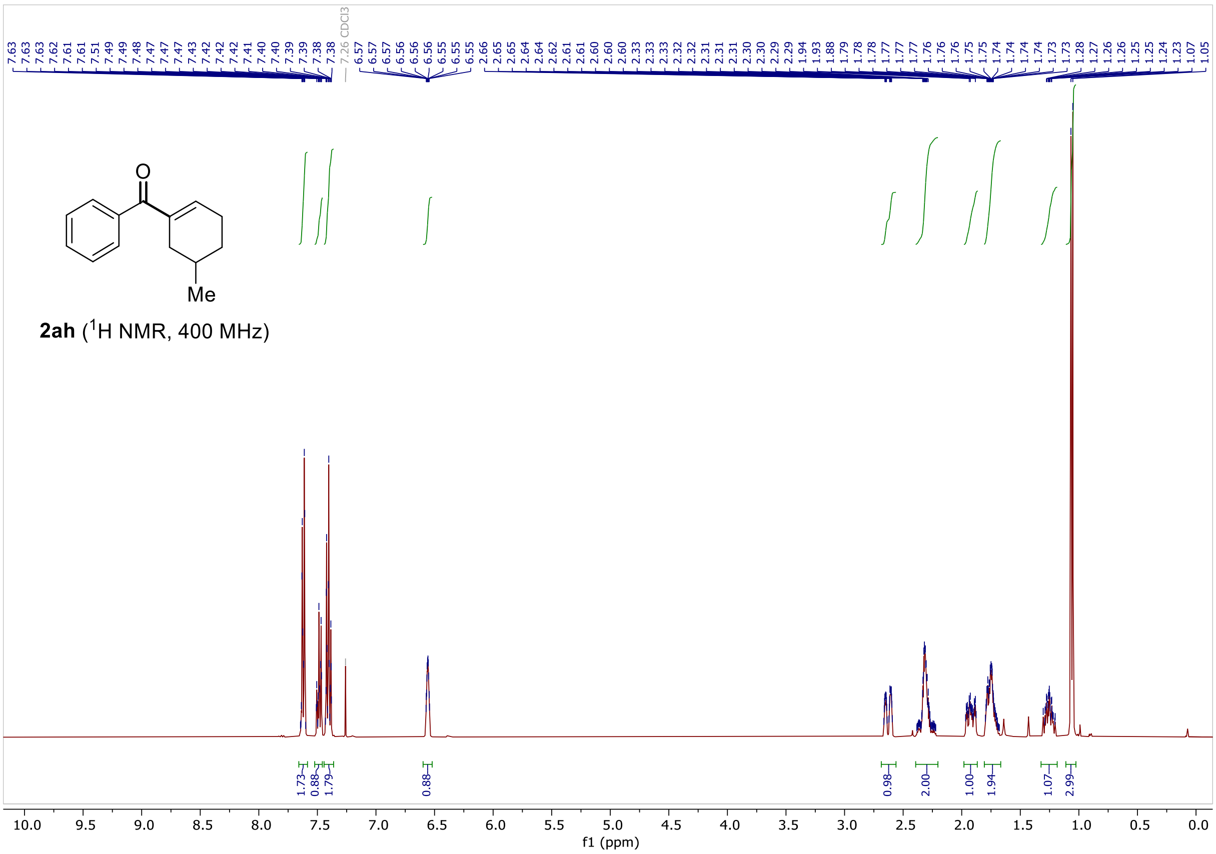
O
Me
2ah (¹H NMR, 400 MHz)
f1 (ppm)

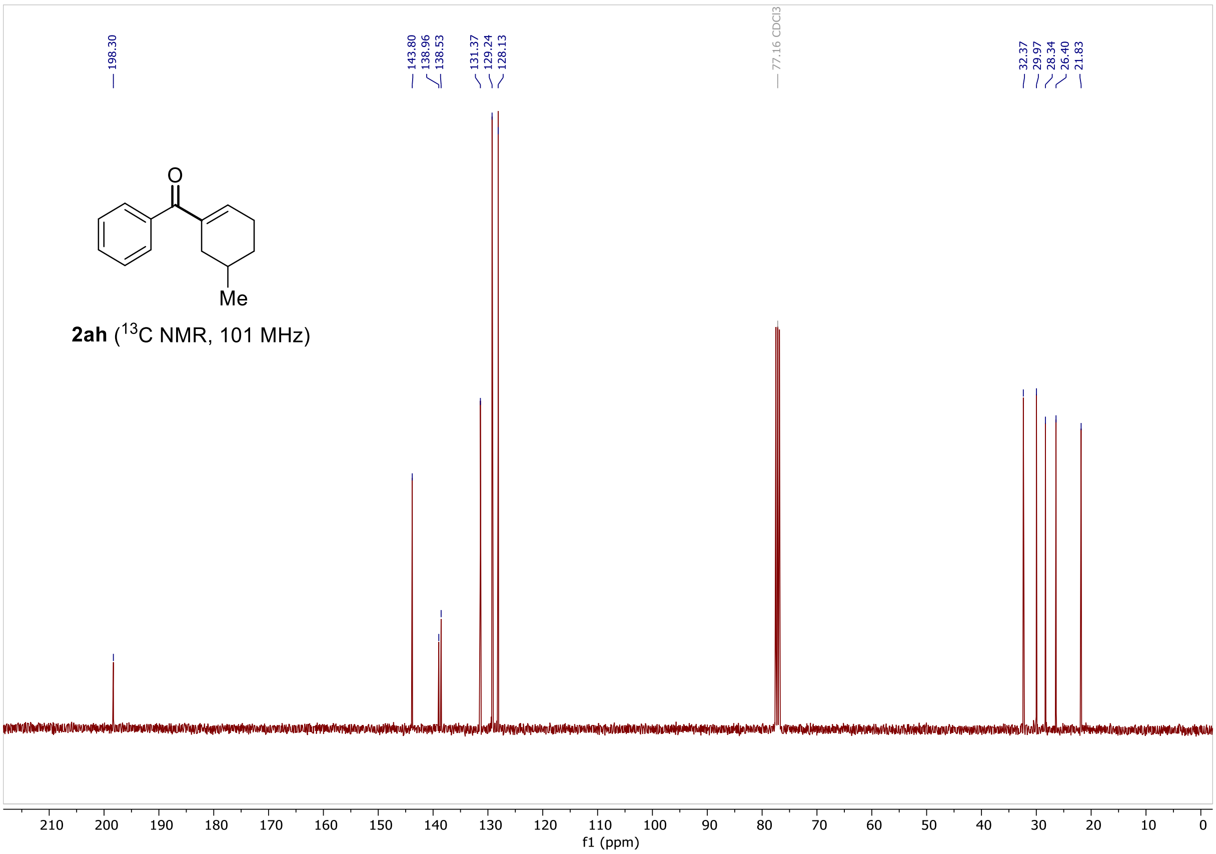
O
Me
2ah (¹³C NMR, 101 MHz)
198.30
143.80
138.96
138.53
131.37
129.24
128.13
77.16 CDCl3
32.37
29.97
28.34
26.40
21.83
f1 (ppm)

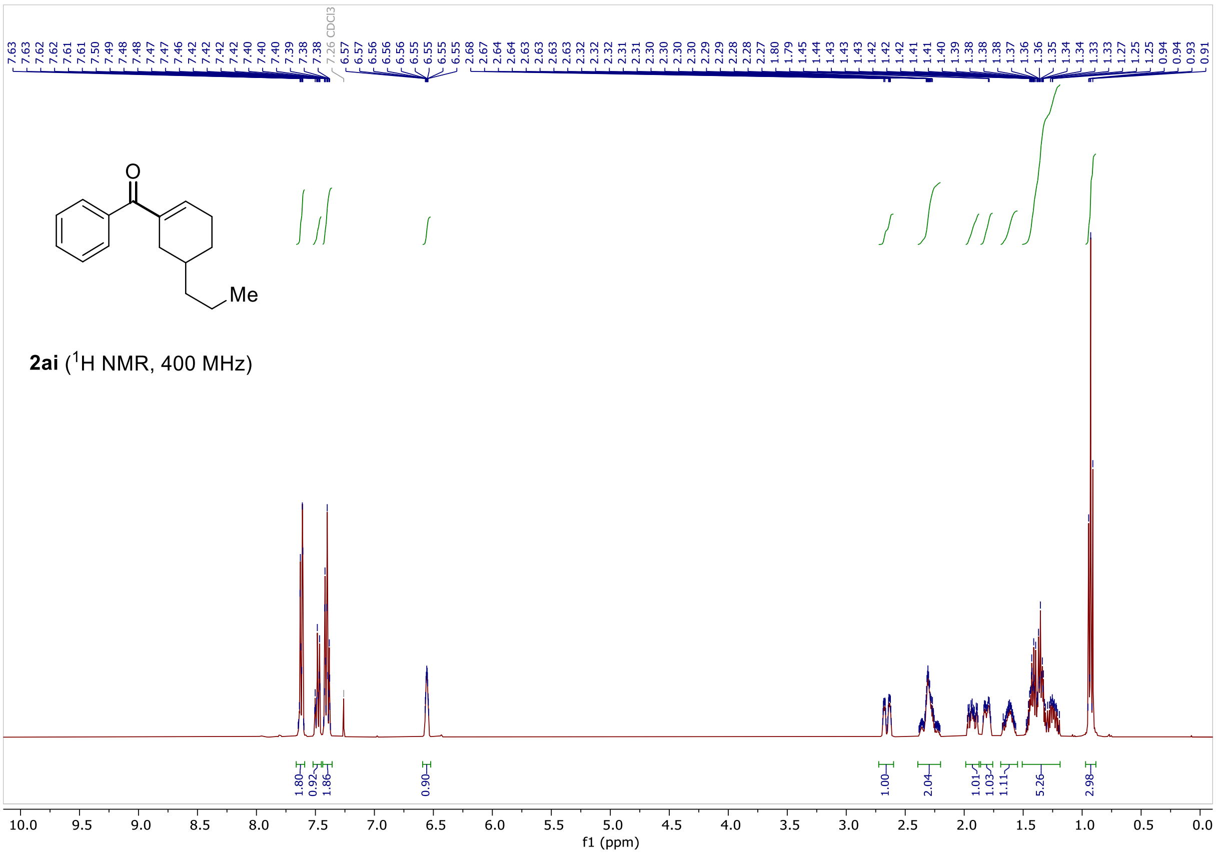

7.63
7.63
7.62
7.62
7.61
7.61
7.50
7.49
7.48
7.48
7.47
7.47
7.46
7.42
7.42
7.42
7.42
7.40
7.40
7.39
7.38
7.38
7.26 CDCl3
6.57
6.57
6.56
6.56
6.56
6.55
6.55
6.55
6.55
2.68
2.67
2.64
2.64
2.63
2.63
2.63
2.32
2.32
2.32
2.31
2.31
2.30
2.30
2.30
2.30
2.29
2.29
2.28
2.28
2.27
1.80
1.79
1.45
1.44
1.43
1.43
1.43
1.42
1.42
1.42
1.41
1.41
1.40
1.39
1.38
1.38
1.38
1.37
1.36
1.36
1.35
1.34
1.34
1.33
1.33
1.27
1.25
0.94
0.94
0.93
0.91
Me
2ai (^{1}H NMR, 400 MHz)
1.80
0.92
1.86
0.90
1.00
2.04
1.01
1.03
1.11
5.26
2.98
10.0
9.5
9.0
8.5
8.0
7.5
7.0
6.5
6.0
5.5
5.0
4.5
4.0
3.5
3.0
2.5
2.0
1.5
1.0
0.5
0.0
f1 (ppm)

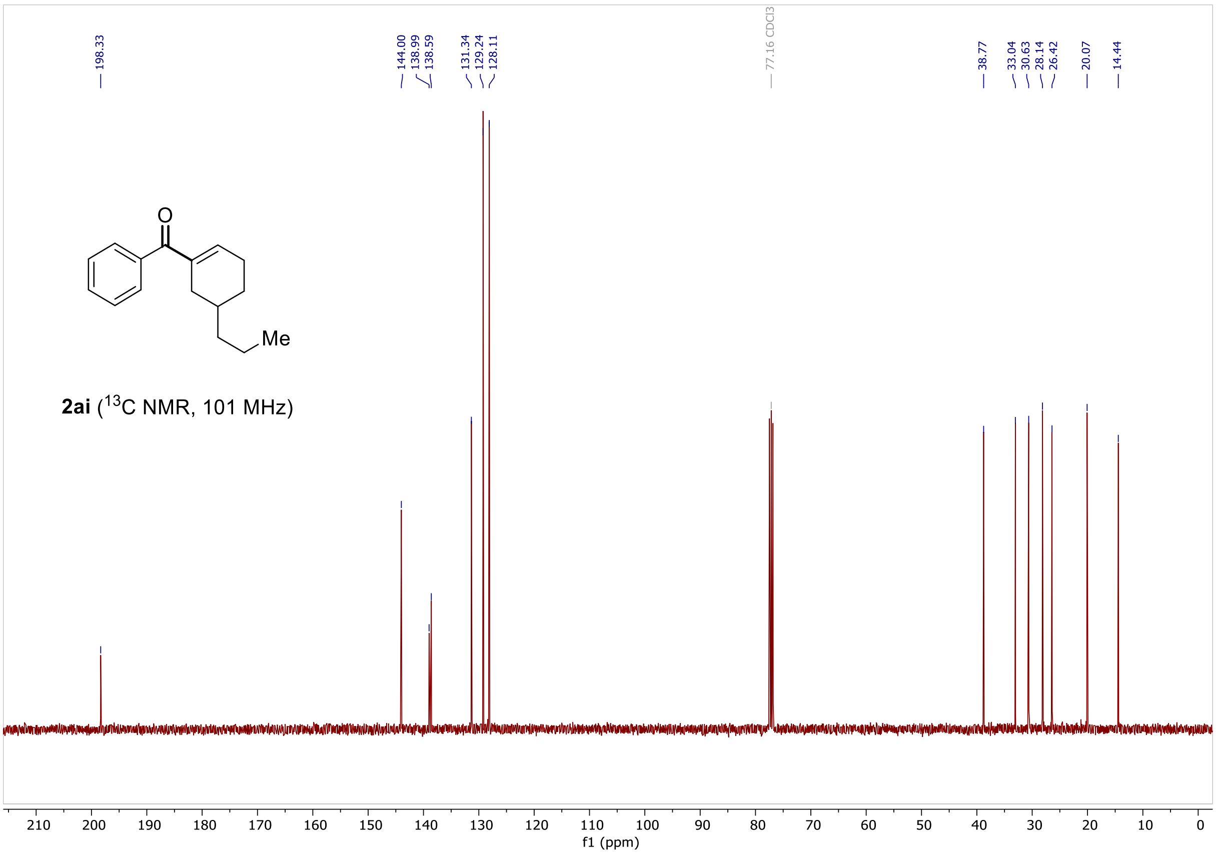

198.33
144.00
138.99
138.59
131.34
129.24
128.11
77.16 CDCl3
38.77
33.04
30.63
28.14
26.42
20.07
14.44
Me
2ai (^{13}C NMR, 101 MHz)
210
200
190
180
170
160
150
140
130
120
110
100
90
80
70
60
50
40
30
20
10
0
f1 (ppm)

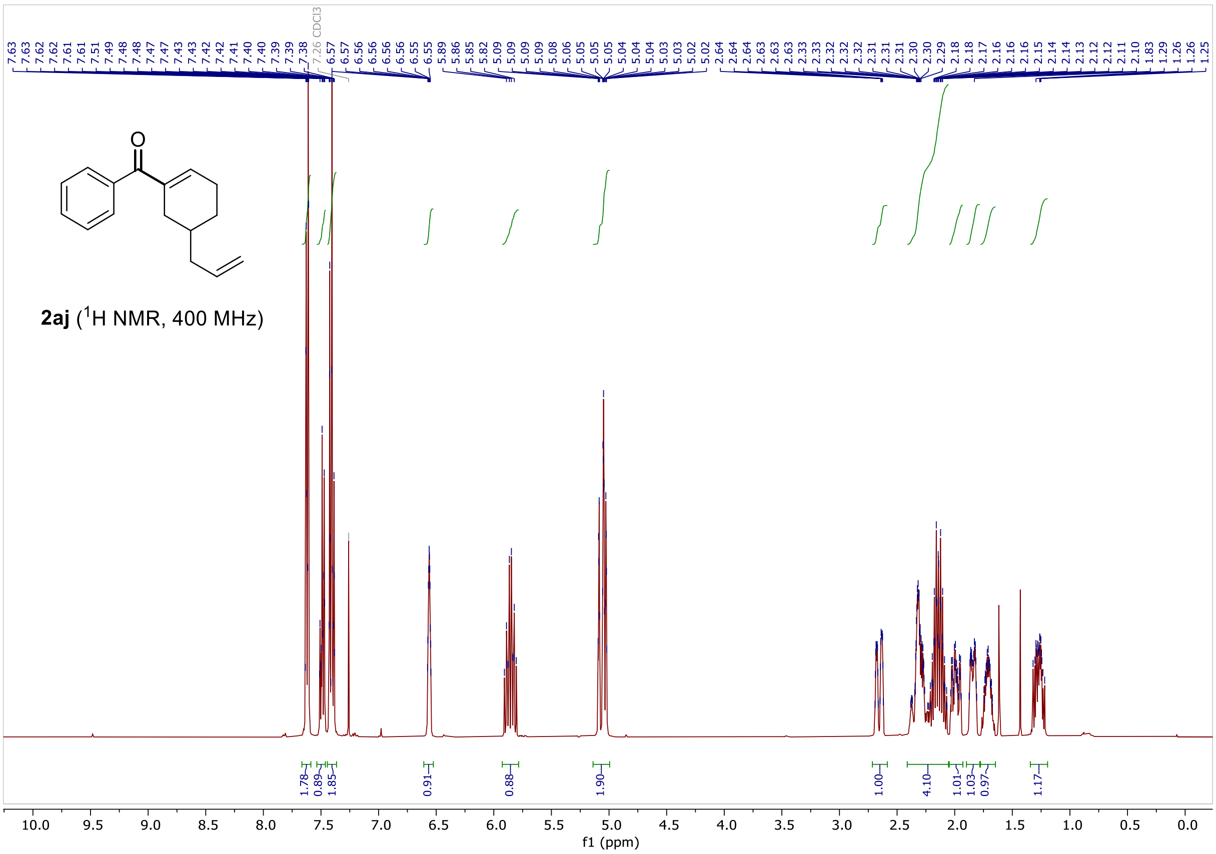

2aj (1H NMR, 400 MHz)
7.26 CDCl3
f1 (ppm)

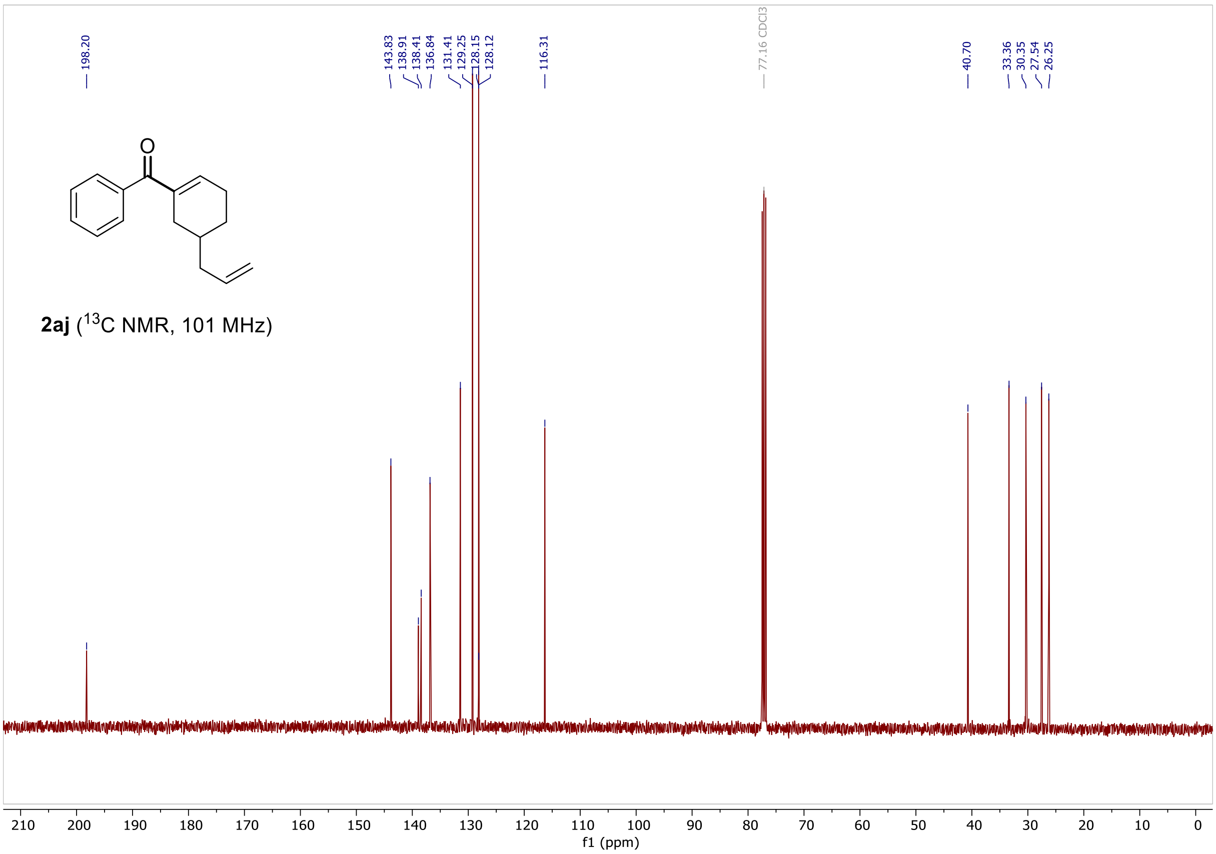

2aj (13C NMR, 101 MHz)
77.16 CDCl3
f1 (ppm)

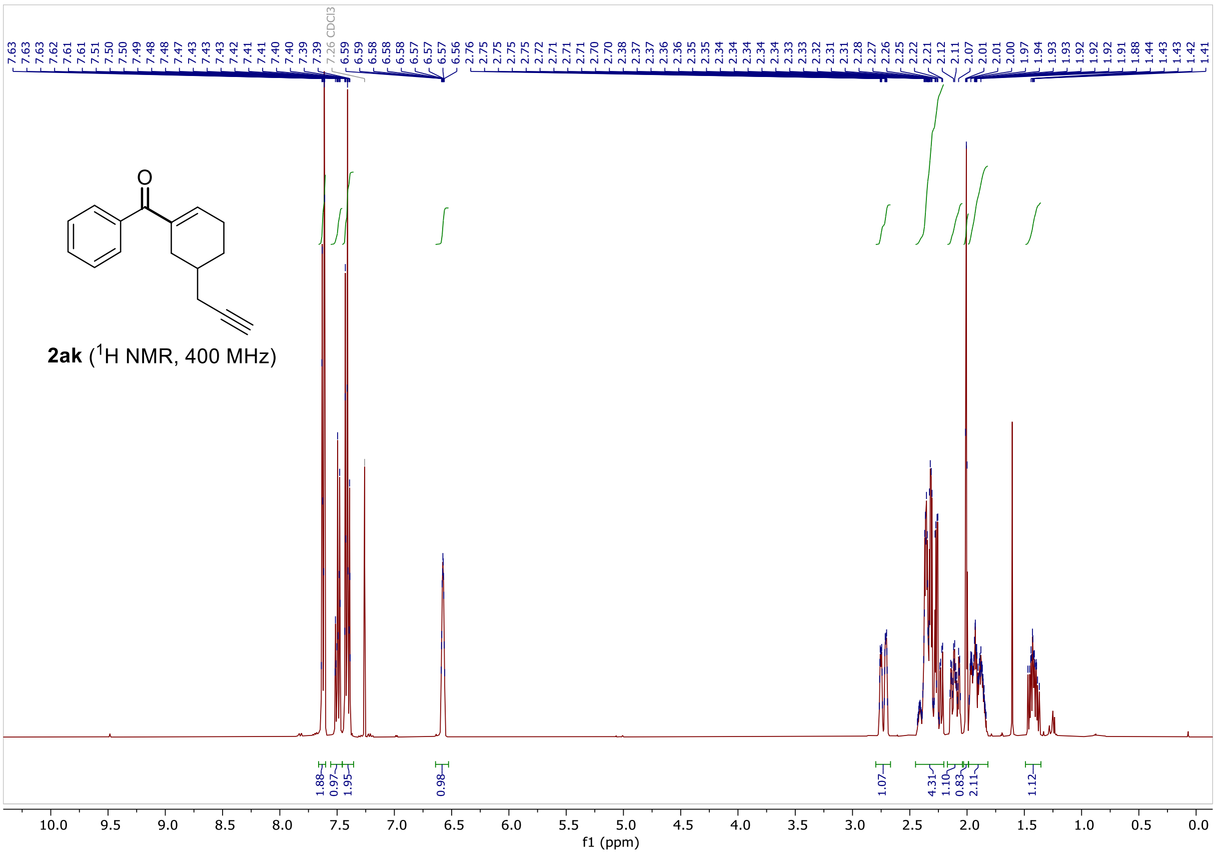
2ak (1H NMR, 400 MHz)
f1 (ppm)

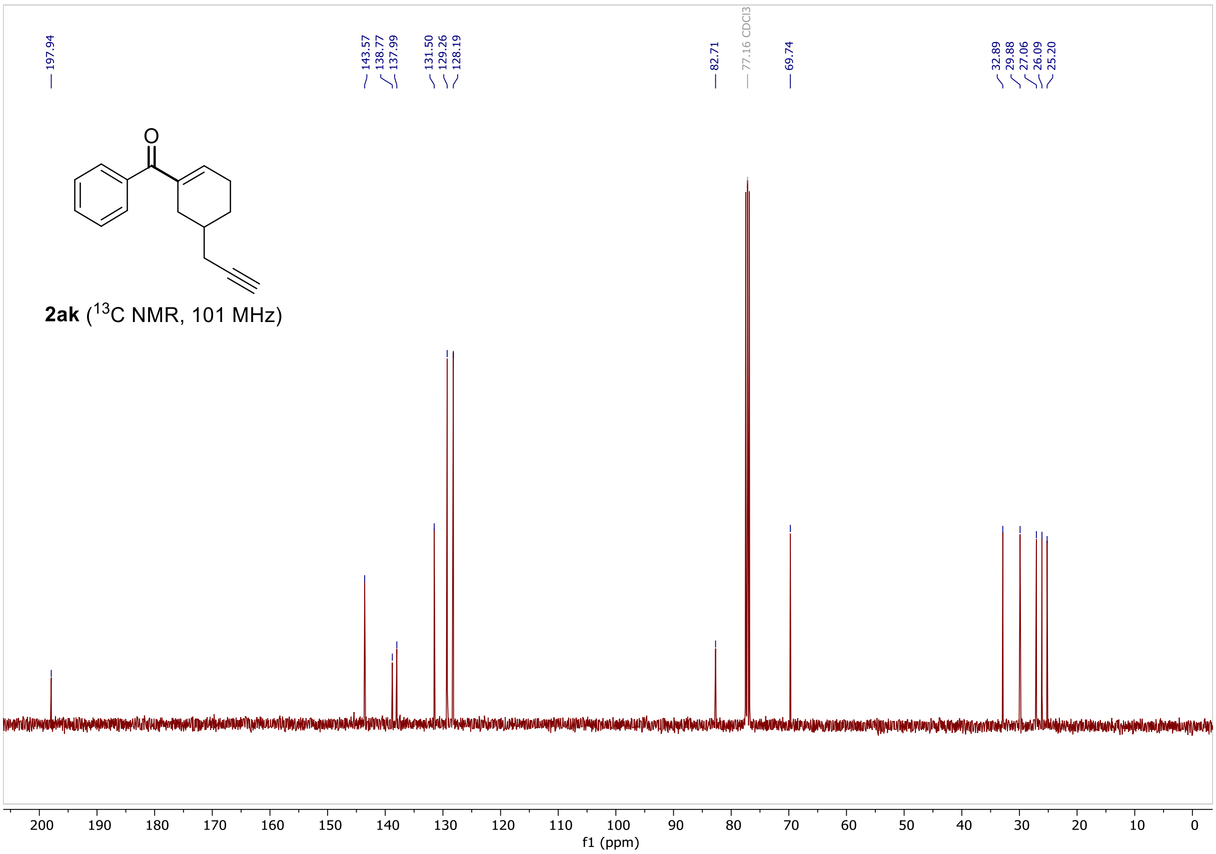
2ak (13C NMR, 101 MHz)
197.94
143.57
138.77
137.99
131.50
129.26
128.19
82.71
77.16 CDCl3
69.74
32.89
29.88
27.06
26.09
25.20
f1 (ppm)

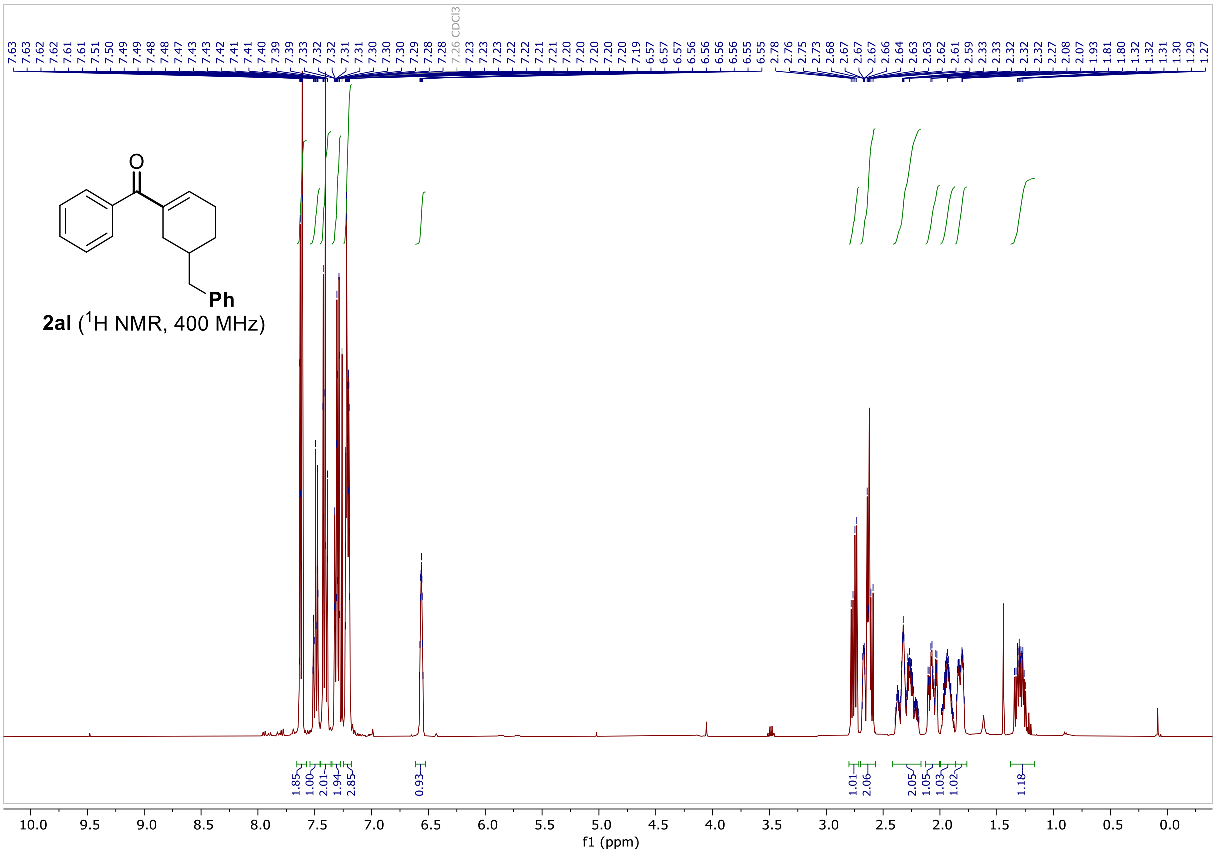

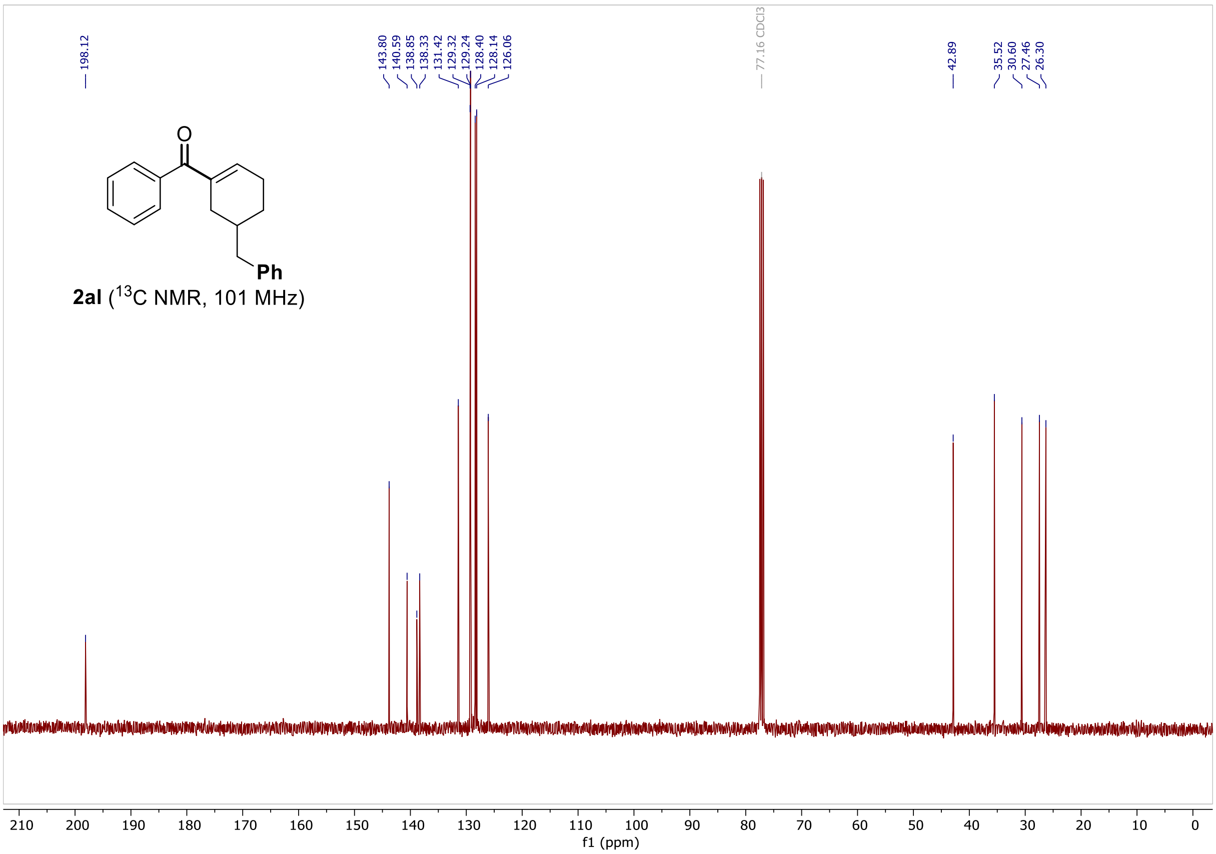

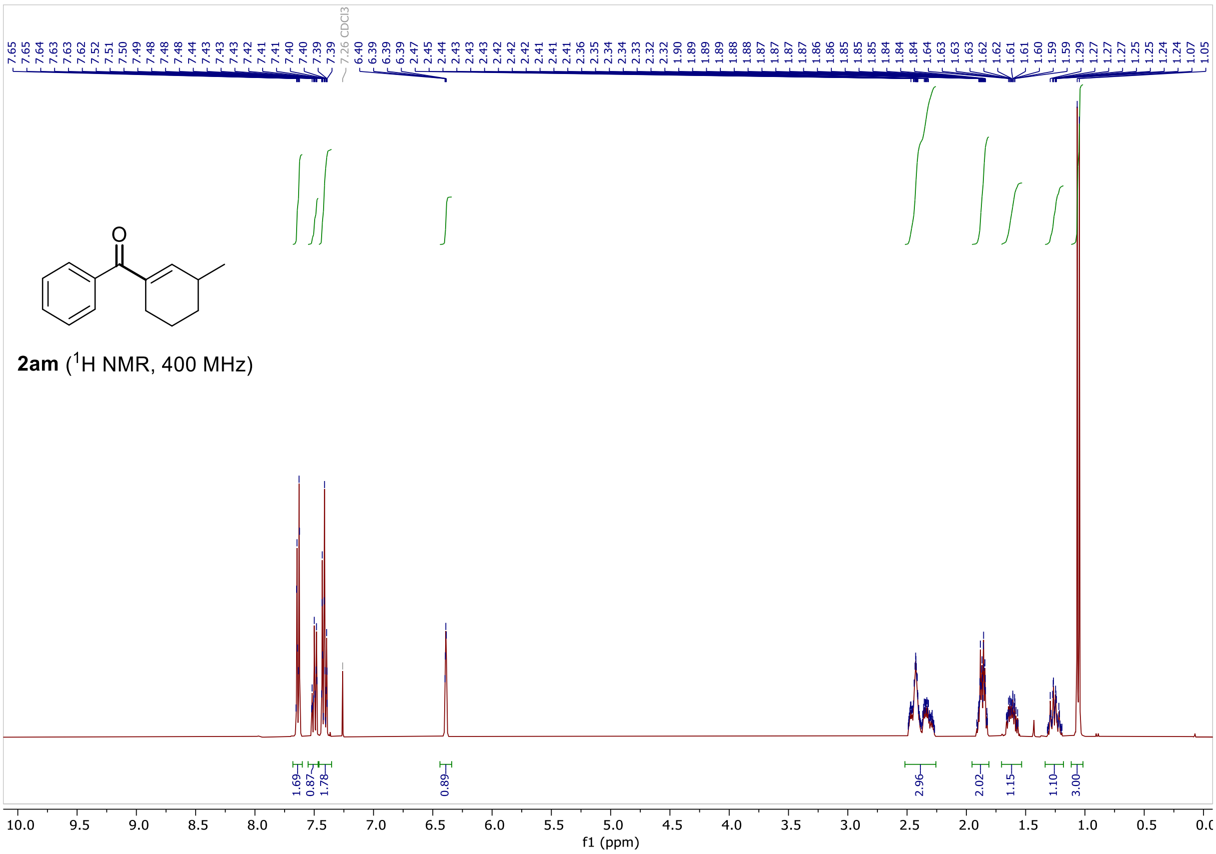
2am (1H NMR, 400 MHz)
7.26 CDCl3
1.69
0.87
1.78
0.89
2.96
2.02
1.15
1.10
3.00
f1 (ppm)

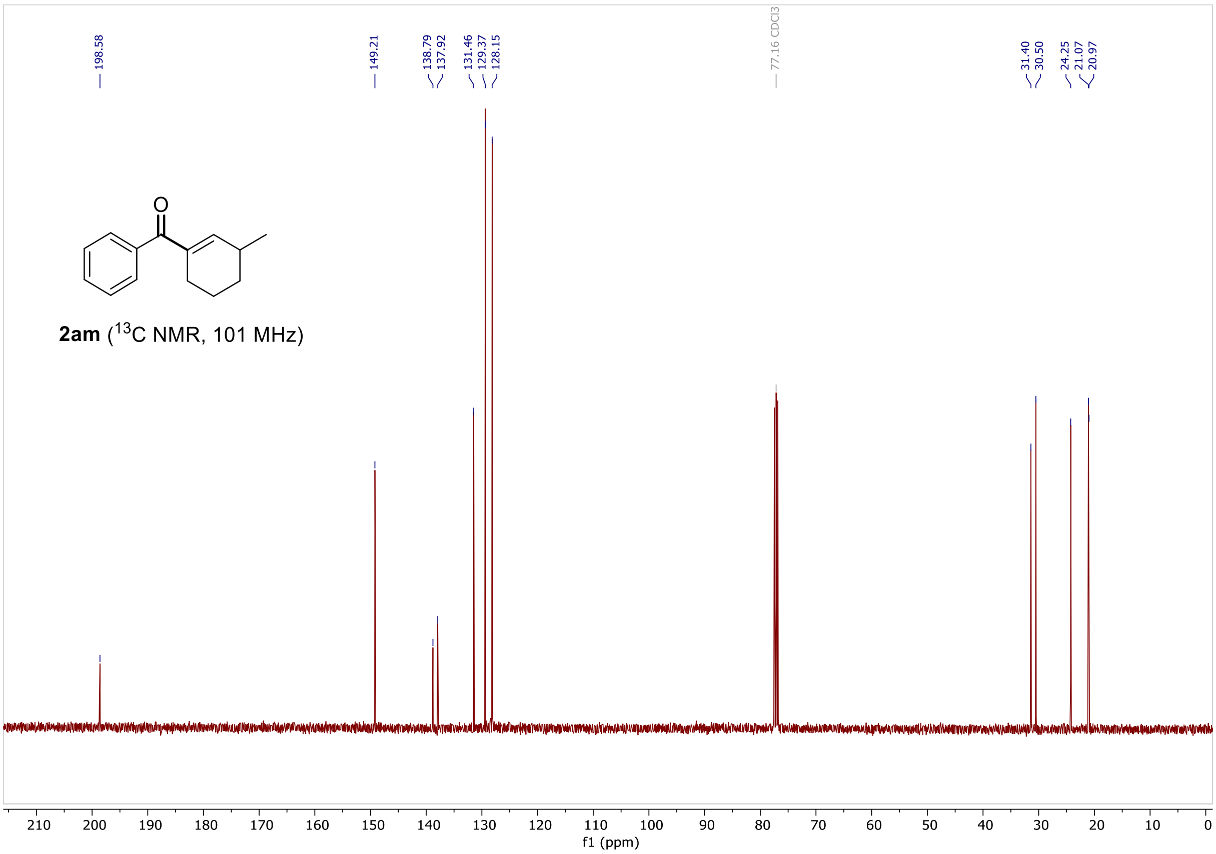
2am (13C NMR, 101 MHz)
198.58
149.21
138.79
137.92
131.46
129.37
128.15
77.16 CDCl3
31.40
30.50
24.25
21.07
20.97
f1 (ppm)

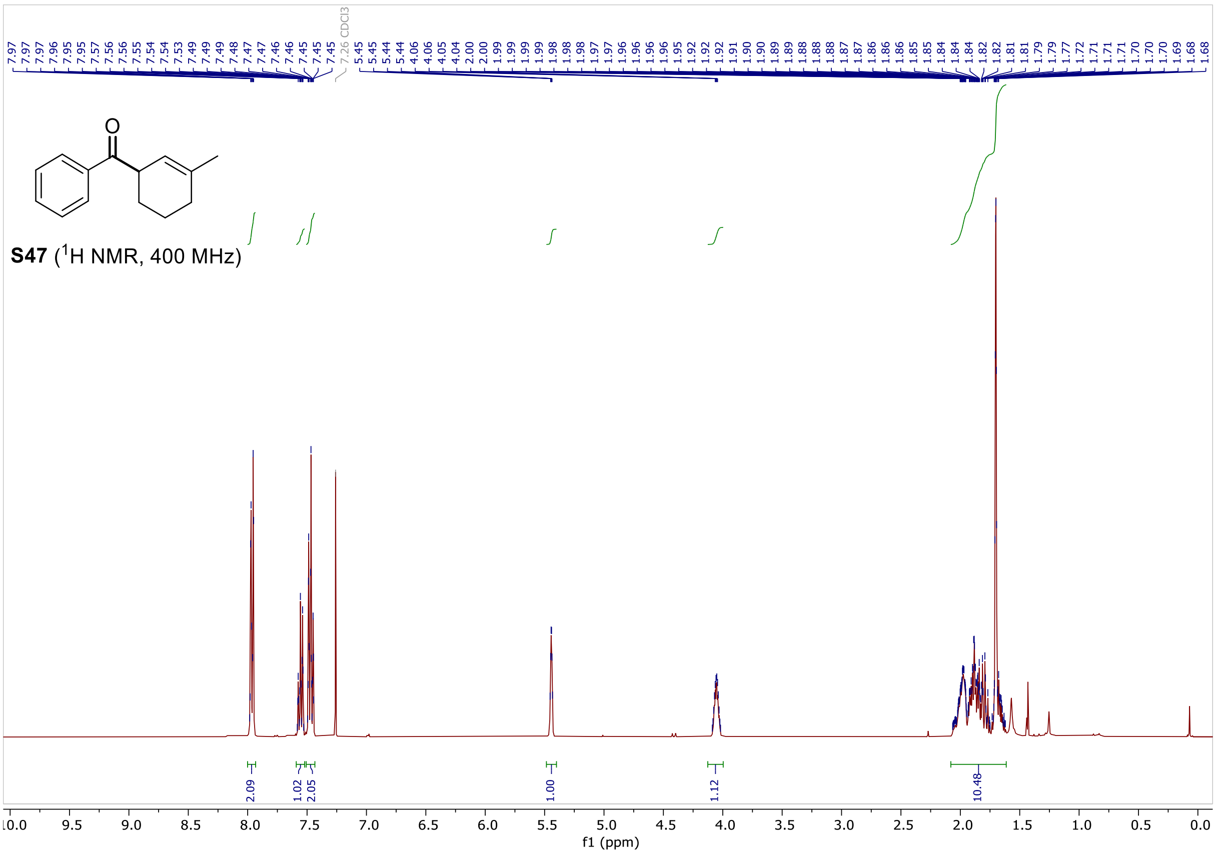
S47 (1H NMR, 400 MHz)
f1 (ppm)

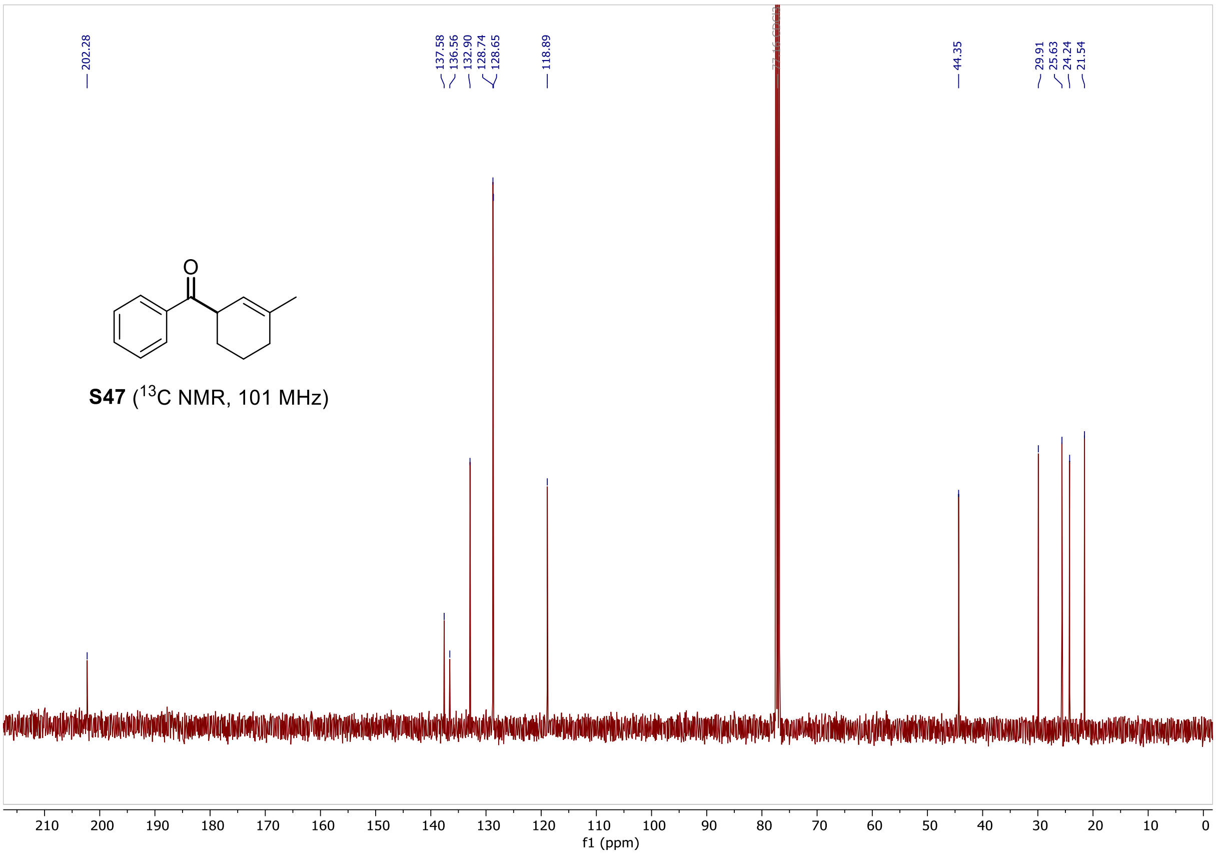
S47 (13C NMR, 101 MHz)
f1 (ppm)

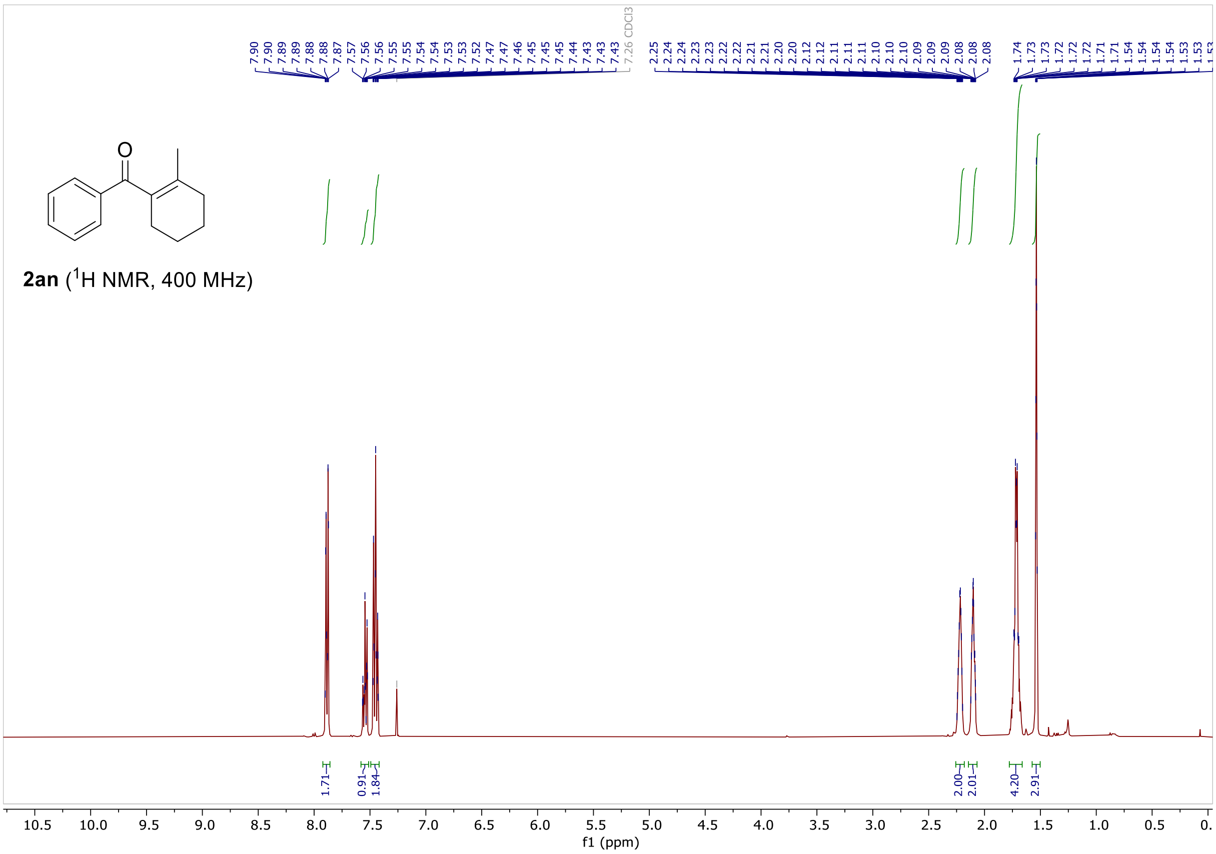
2an (1H NMR, 400 MHz)
f1 (ppm)

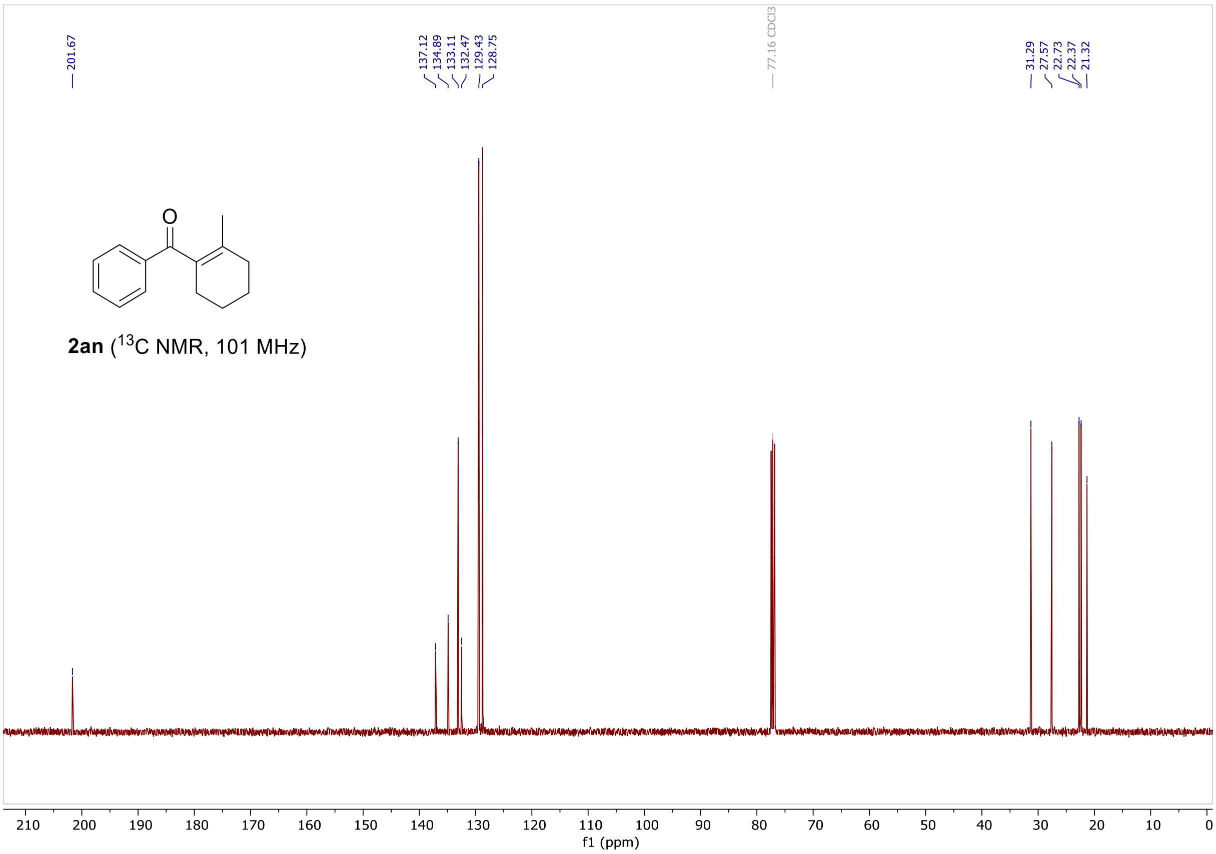
201.67
137.12
134.89
133.11
132.47
129.43
128.75
77.16 CDCl3
31.29
27.57
22.73
22.37
21.32
2an (13C NMR, 101 MHz)
f1 (ppm)

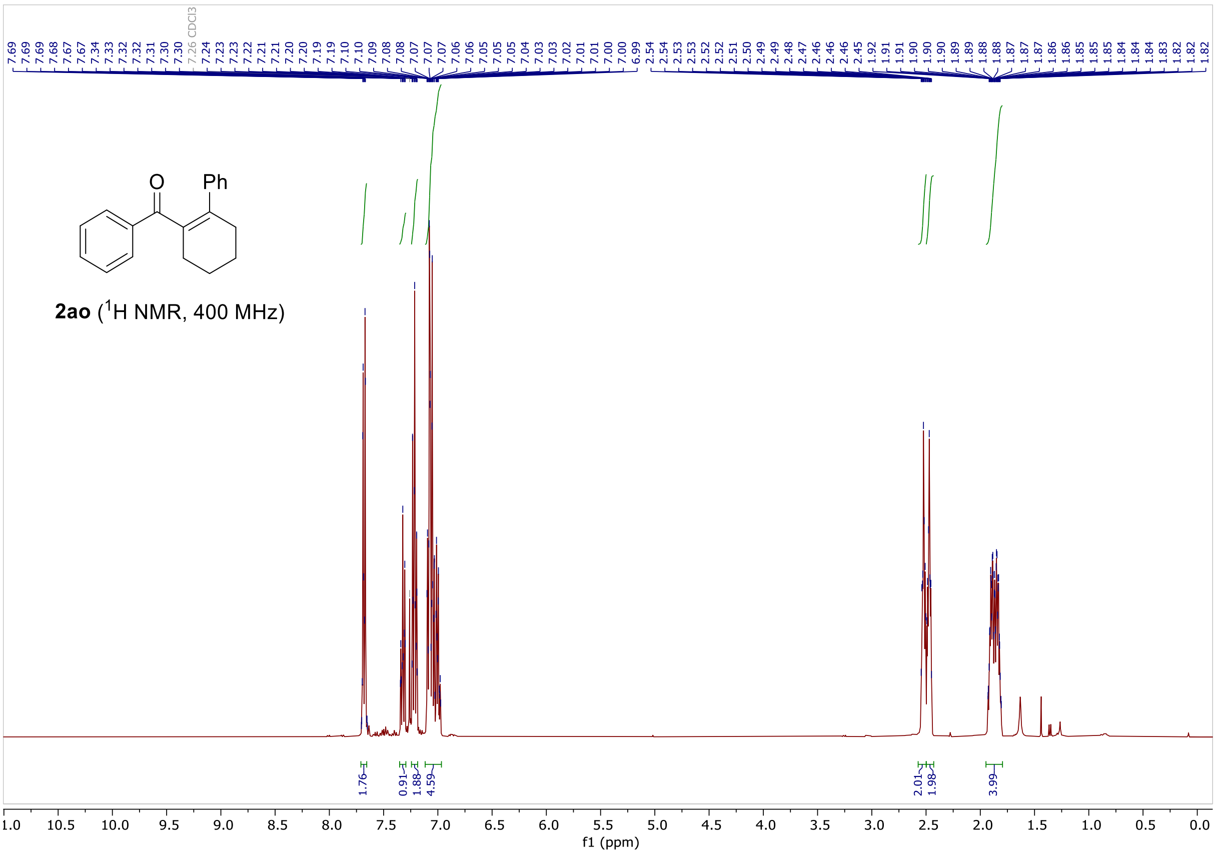
O
Ph
2ao (1H NMR, 400 MHz)
7.69 7.69 7.68 7.67 7.67 7.34 7.33 7.32 7.32 7.31 7.30 7.26 CDCl3 7.24 7.23 7.23 7.22 7.21 7.21 7.20 7.19 7.19 7.10 7.10 7.09 7.08 7.08 7.07 7.07 7.07 7.06 7.05 7.05 7.05 7.04 7.03 7.03 7.02 7.01 7.01 7.00 7.00 6.99
2.54 2.54 2.53 2.53 2.52 2.52 2.51 2.50 2.49 2.49 2.48 2.47 2.46 2.46 2.45 1.92 1.91 1.91 1.90 1.90 1.90 1.89 1.88 1.88 1.87 1.87 1.86 1.86 1.85 1.85 1.85 1.84 1.84 1.83 1.82 1.82 1.82
1.76 0.91 1.88 4.59 2.01 1.98 3.99
f1 (ppm)

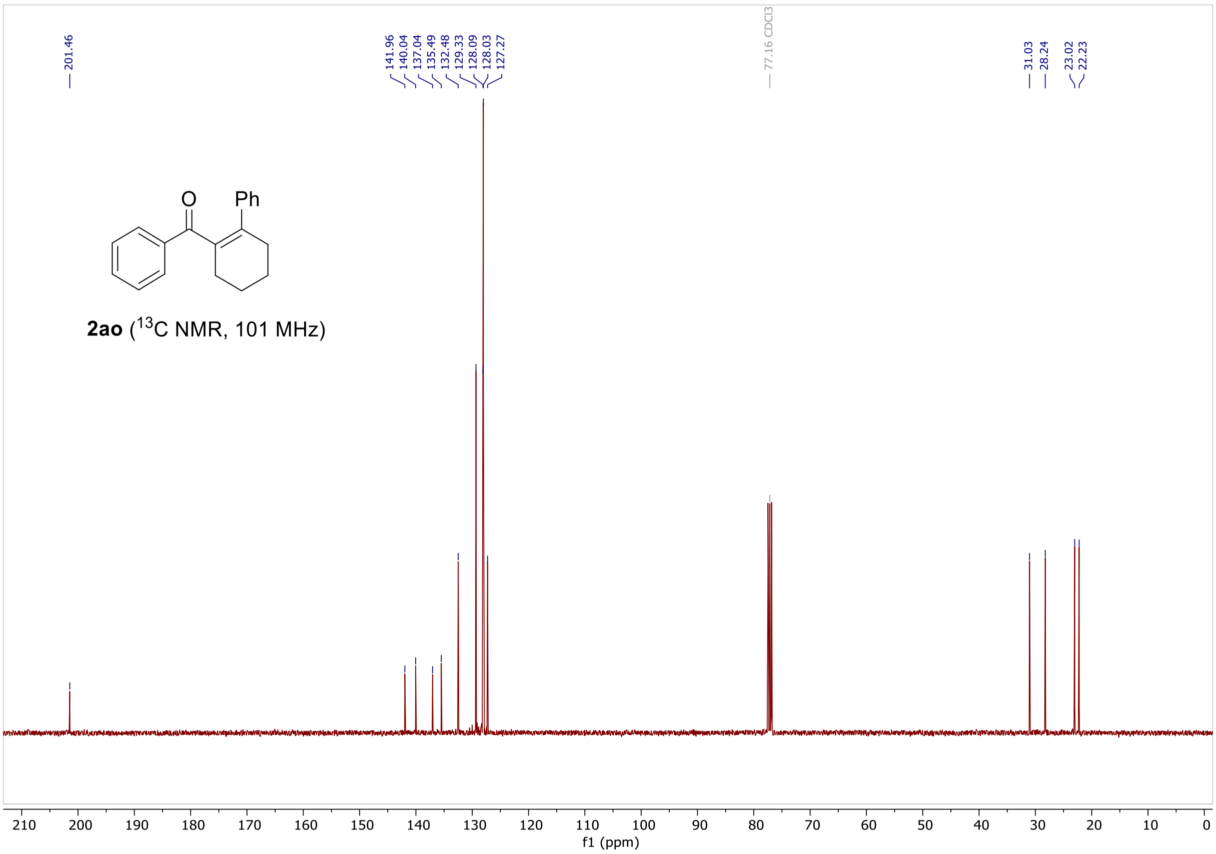
O
Ph
2ao (13C NMR, 101 MHz)
201.46
141.96 140.04 137.04 135.49 132.48 129.33 128.09 128.03 127.27
77.16 CDCl3
31.03 28.24 23.02 22.23
f1 (ppm)

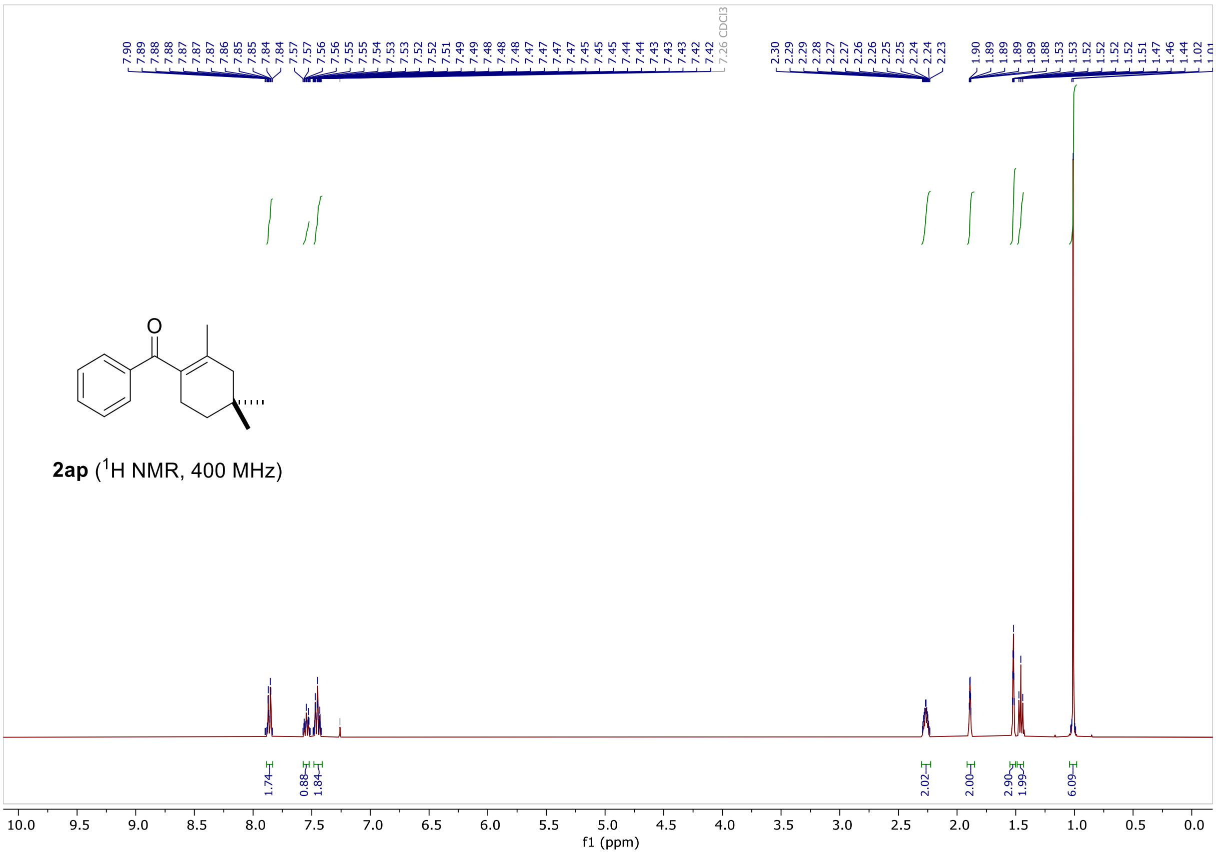
2ap (¹H NMR, 400 MHz)
f1 (ppm)

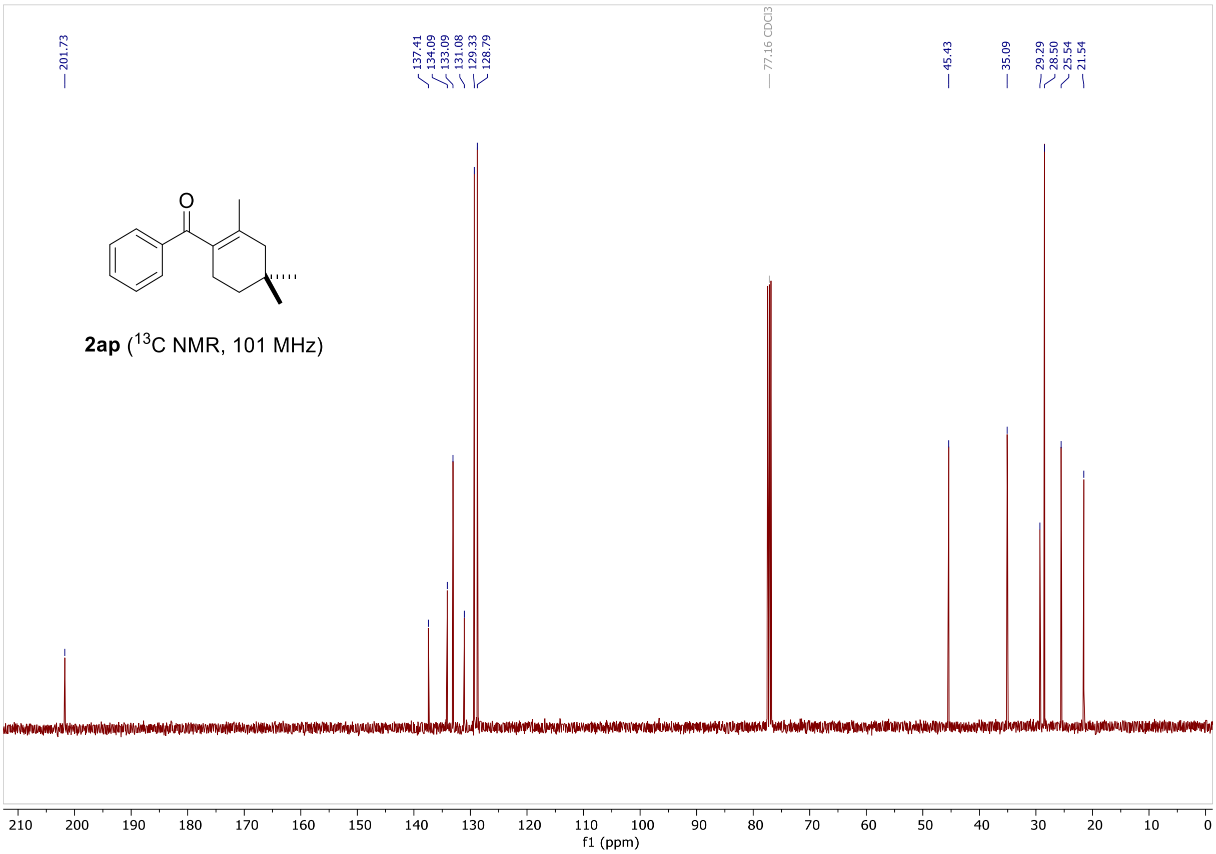
2ap (¹³C NMR, 101 MHz)
201.73
137.41
134.09
133.09
131.08
129.33
128.79
77.16 CDCl3
45.43
35.09
29.29
28.50
25.54
21.54
f1 (ppm)

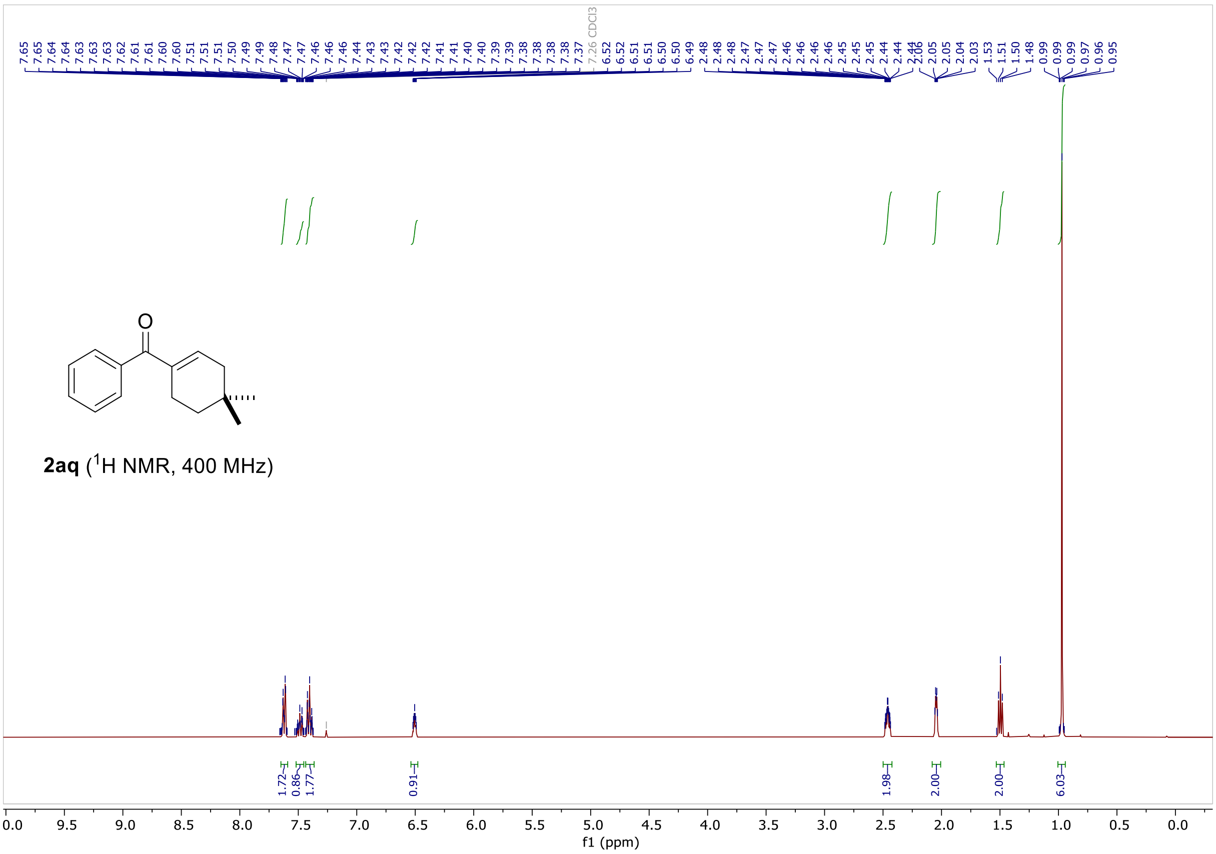
2aq (^{1}H NMR, 400 MHz)
7.26 CDCl3
1.72
0.86
1.77
0.91
1.98
2.00
2.00
6.03
f1 (ppm)

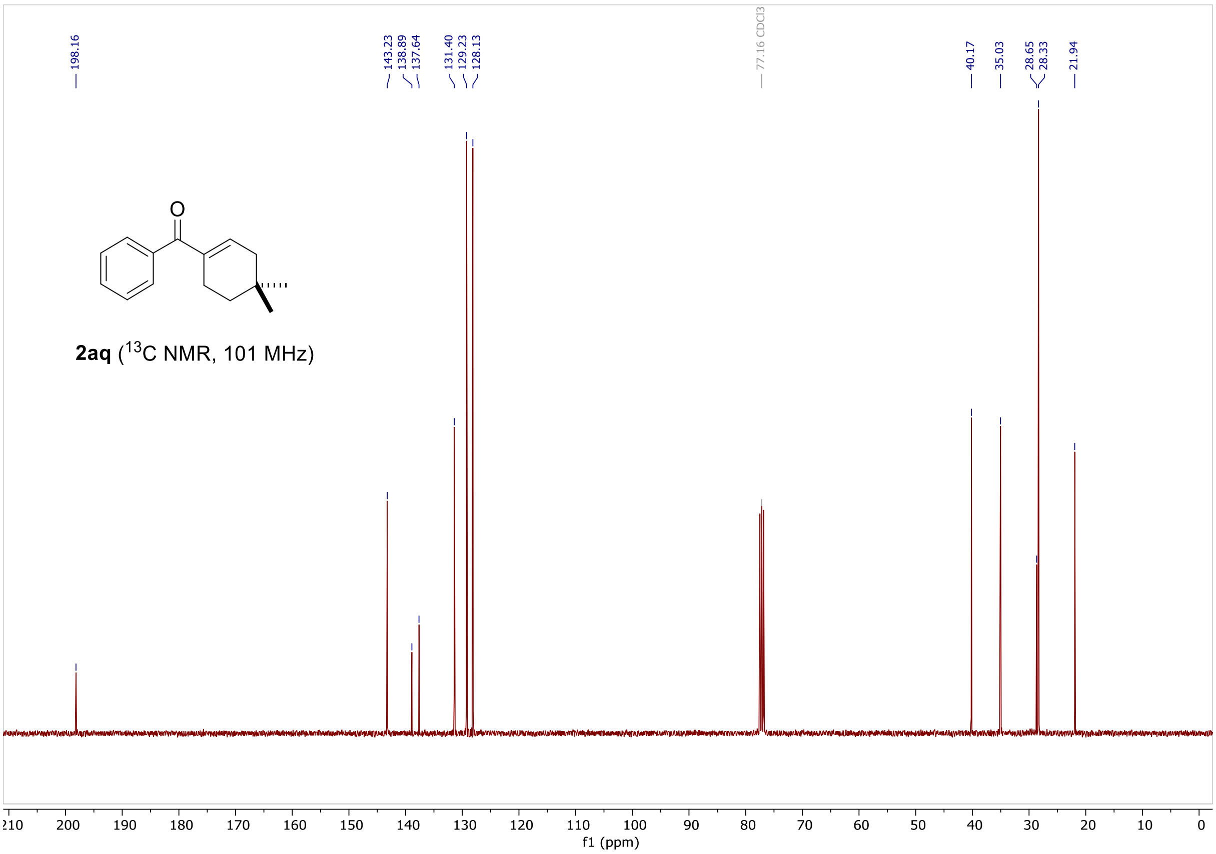
2aq (^{13}C NMR, 101 MHz)
198.16
143.23
138.89
137.64
131.40
129.23
128.13
77.16 CDCl3
40.17
35.03
28.65
28.33
21.94
f1 (ppm)

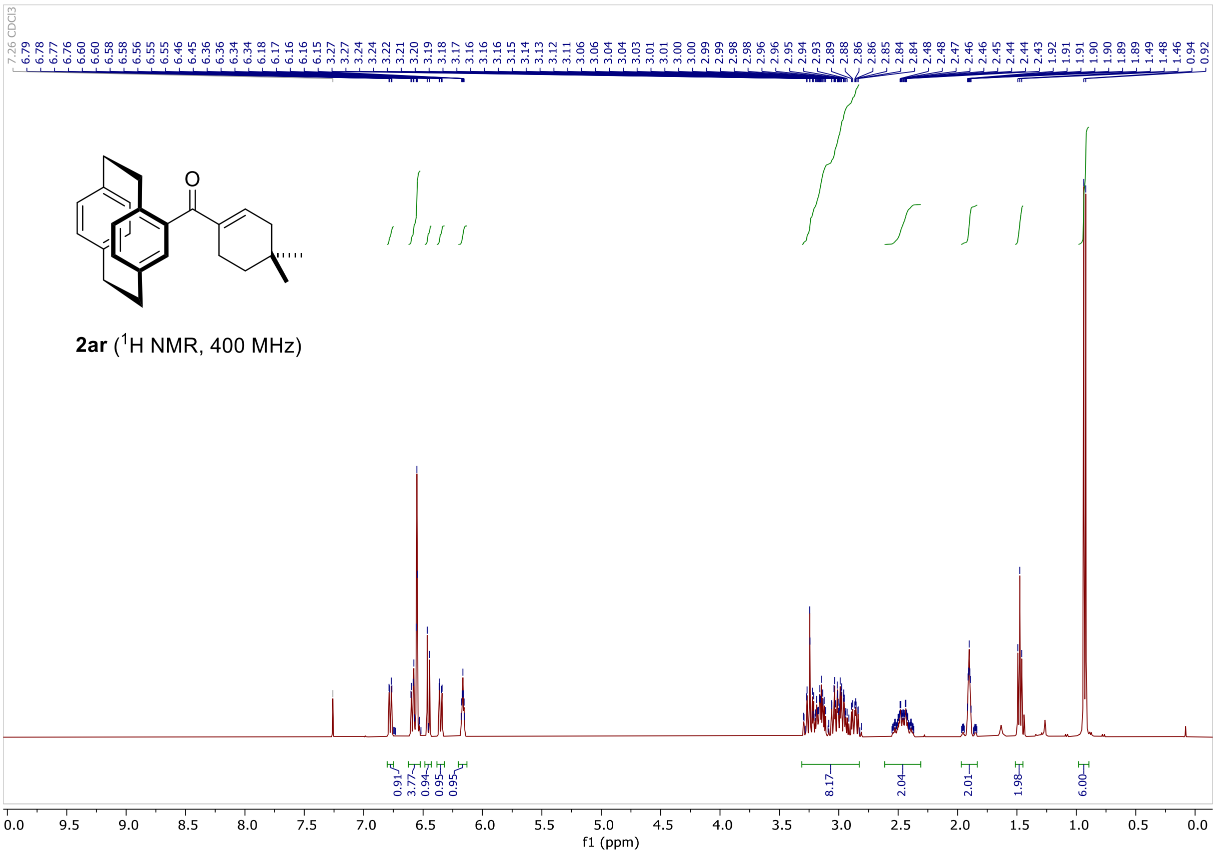
7.26 CDCl3
6.79 6.78 6.77 6.76 6.60 6.60 6.58 6.56 6.55 6.55 6.46 6.45 6.36 6.36 6.34 6.18 6.17 6.16 6.16 6.15 3.27 3.27 3.24 3.24 3.22 3.21 3.20 3.19 3.18 3.17 3.16 3.16 3.15 3.14 3.13 3.12 3.11 3.06 3.06 3.04 3.04 3.03 3.01 3.01 3.00 3.00 2.99 2.99 2.98 2.98 2.96 2.96 2.95 2.94 2.93 2.89 2.88 2.86 2.85 2.84 2.84 2.48 2.47 2.46 2.46 2.45 2.44 2.44 2.43 1.92 1.91 1.91 1.90 1.90 1.89 1.49 1.48 1.46 0.94 0.92
2ar (1H NMR, 400 MHz)
0.91 3.77 0.94 0.95 0.95 8.17 2.04 2.01 1.98 6.00
10.0 9.5 9.0 8.5 8.0 7.5 7.0 6.5 6.0 5.5 5.0 4.5 4.0 3.5 3.0 2.5 2.0 1.5 1.0 0.5 0.0
f1 (ppm)

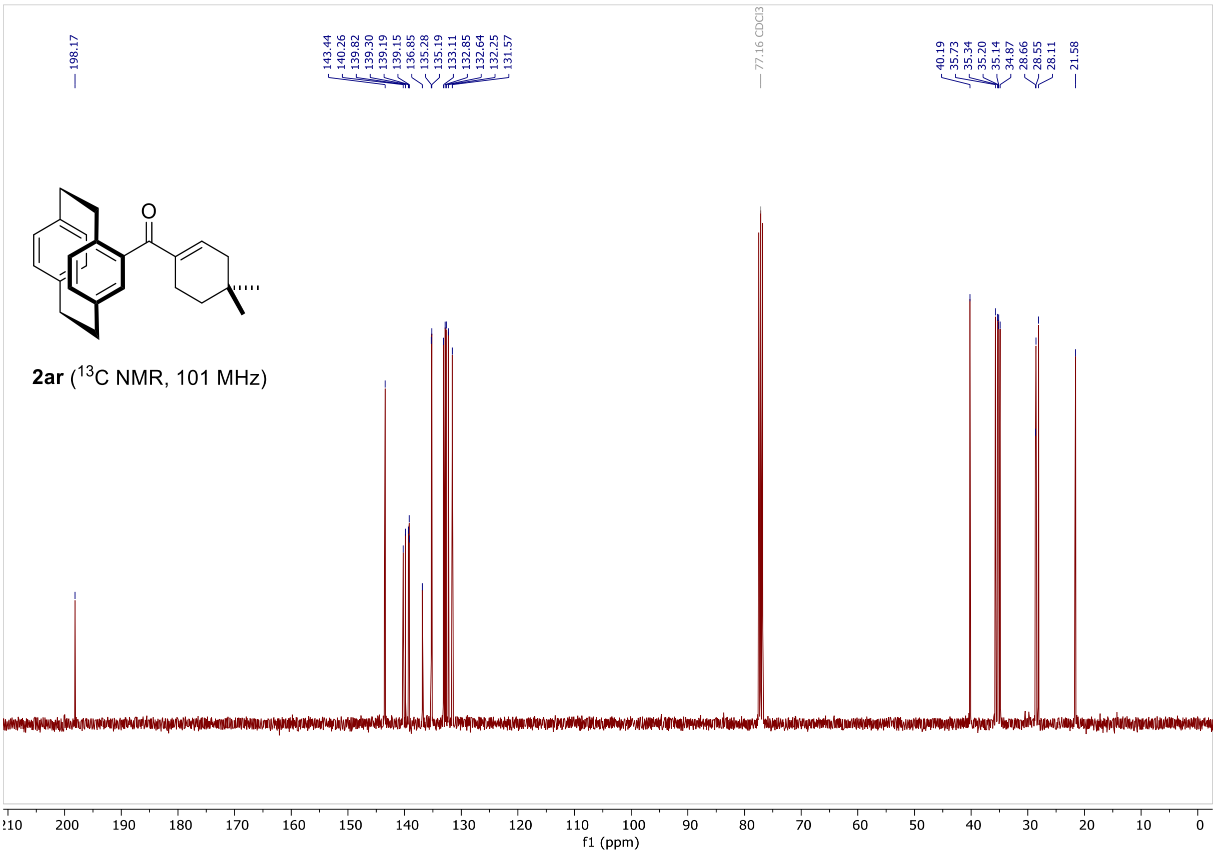
198.17
143.44 140.26 139.82 139.30 139.19 139.15 136.85 135.28 135.19 133.11 132.85 132.64 132.25 131.57
77.16 CDCl3
40.19 35.73 35.34 35.20 35.14 34.87 28.66 28.55 28.11 21.58
2ar (13C NMR, 101 MHz)
210 200 190 180 170 160 150 140 130 120 110 100 90 80 70 60 50 40 30 20 10 0
f1 (ppm)

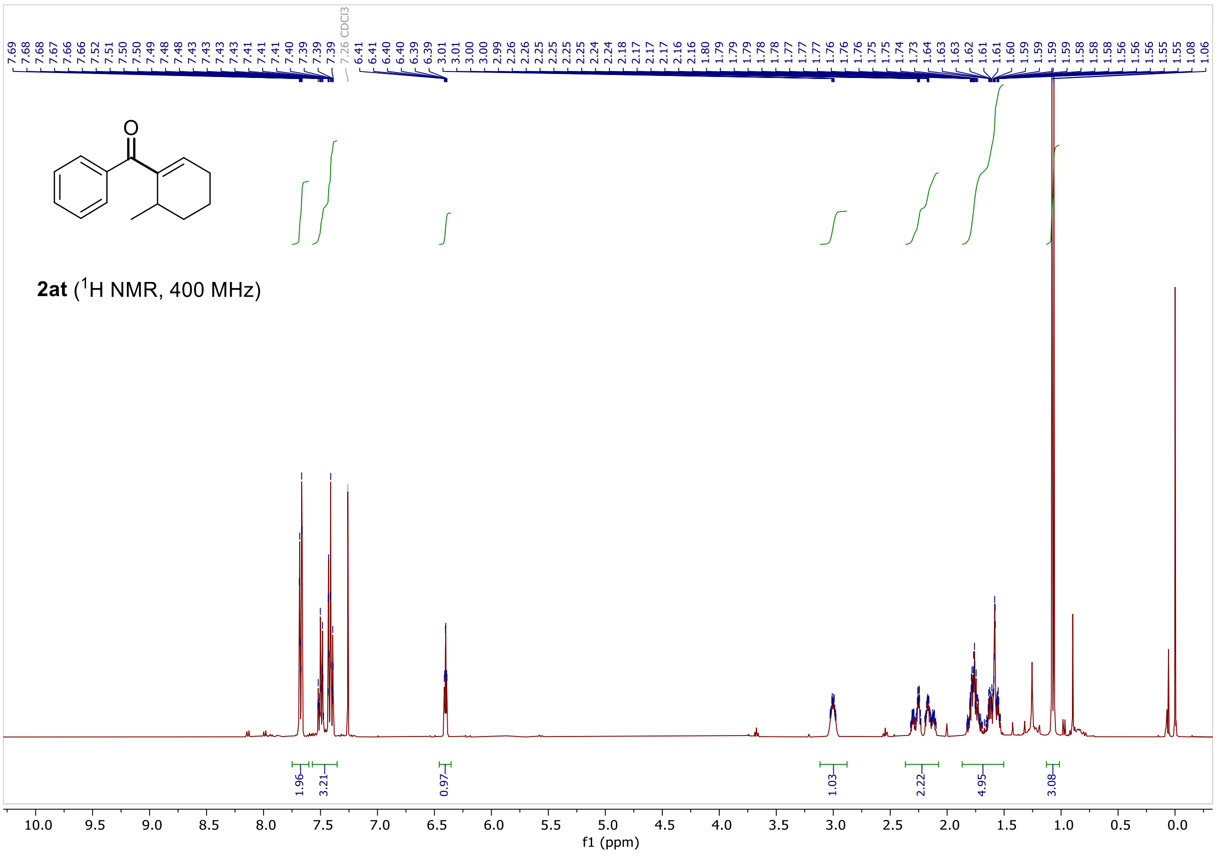

2at (^{1}H NMR, 400 MHz)

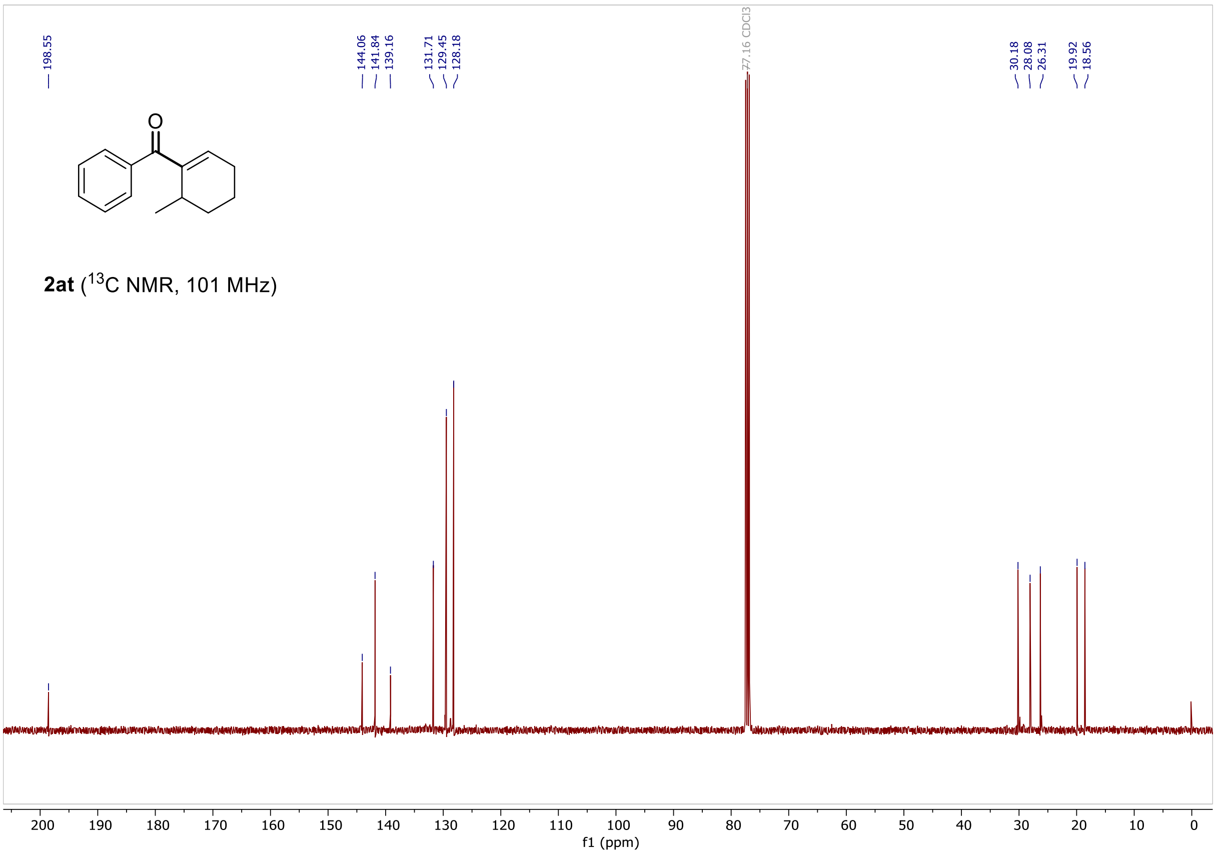

2at (^{13}C NMR, 101 MHz)

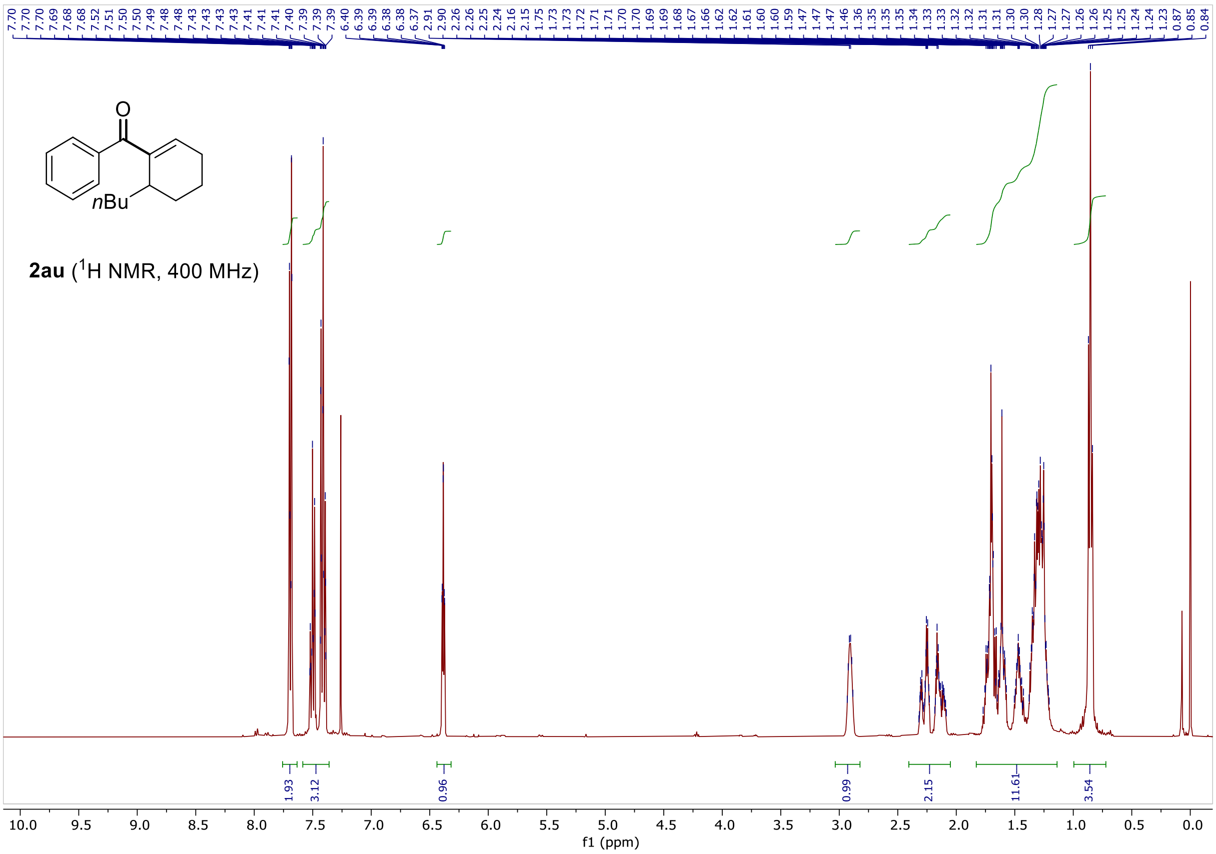
O
nBu
2au (1H NMR, 400 MHz)
7.70 7.70 7.70 7.69 7.68 7.68 7.52 7.51 7.50 7.50 7.49 7.48 7.48 7.43 7.43 7.43 7.41 7.41 7.40 7.39 7.39 7.39 6.40 6.39 6.39 6.38 6.38 6.37 2.91 2.90 2.26 2.26 2.25 2.24 2.16 2.15 1.75 1.73 1.73 1.72 1.71 1.71 1.70 1.70 1.69 1.69 1.68 1.67 1.66 1.62 1.62 1.61 1.60 1.60 1.59 1.47 1.47 1.47 1.46 1.36 1.35 1.35 1.35 1.34 1.33 1.33 1.32 1.32 1.31 1.31 1.30 1.30 1.28 1.27 1.27 1.26 1.25 1.25 1.25 1.24 1.23 0.87 0.85 0.84
1.93 3.12 0.96 0.99 2.15 11.61 3.54
f1 (ppm)

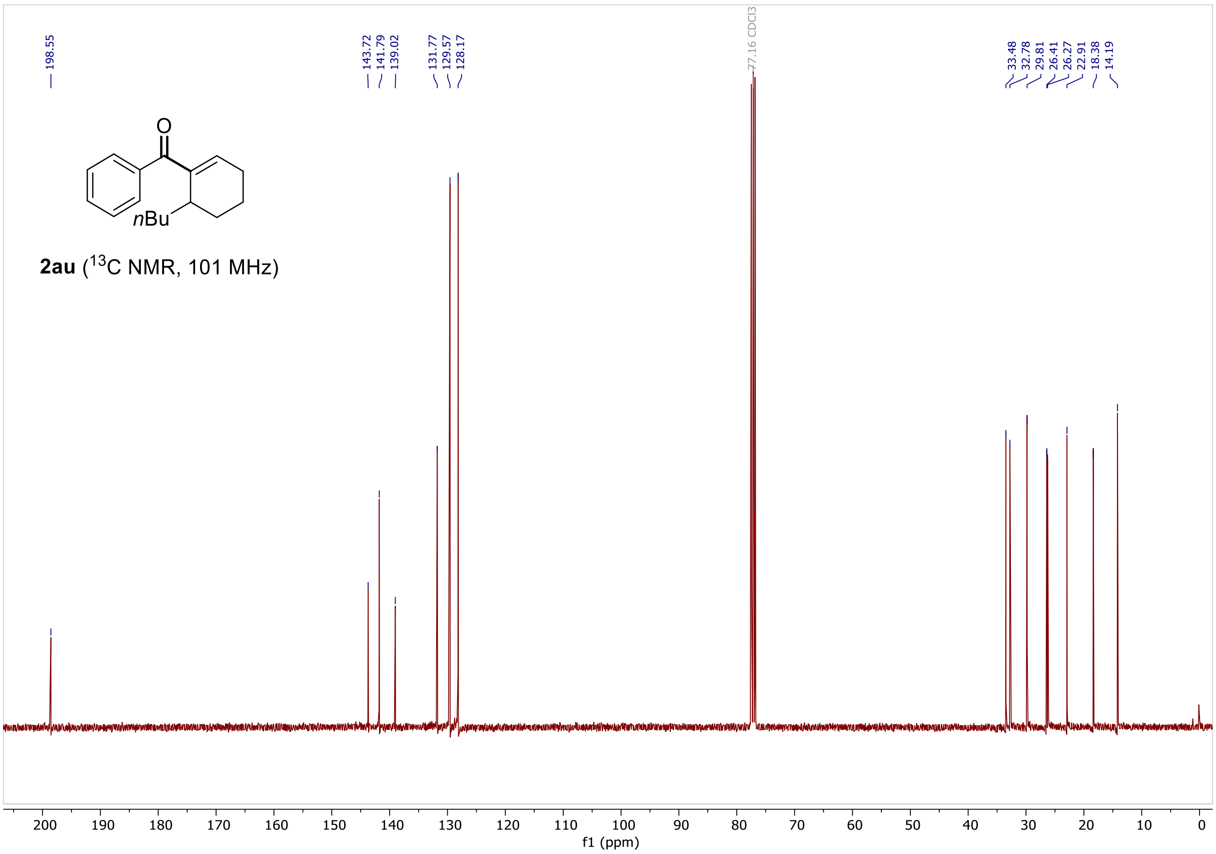
198.55
143.72 141.79 139.02
131.77 129.57 128.17
77.16 CDCl3
33.48 32.78 29.81 26.41 26.27 22.91 18.38 14.19
O
nBu
2au (13C NMR, 101 MHz)
f1 (ppm)

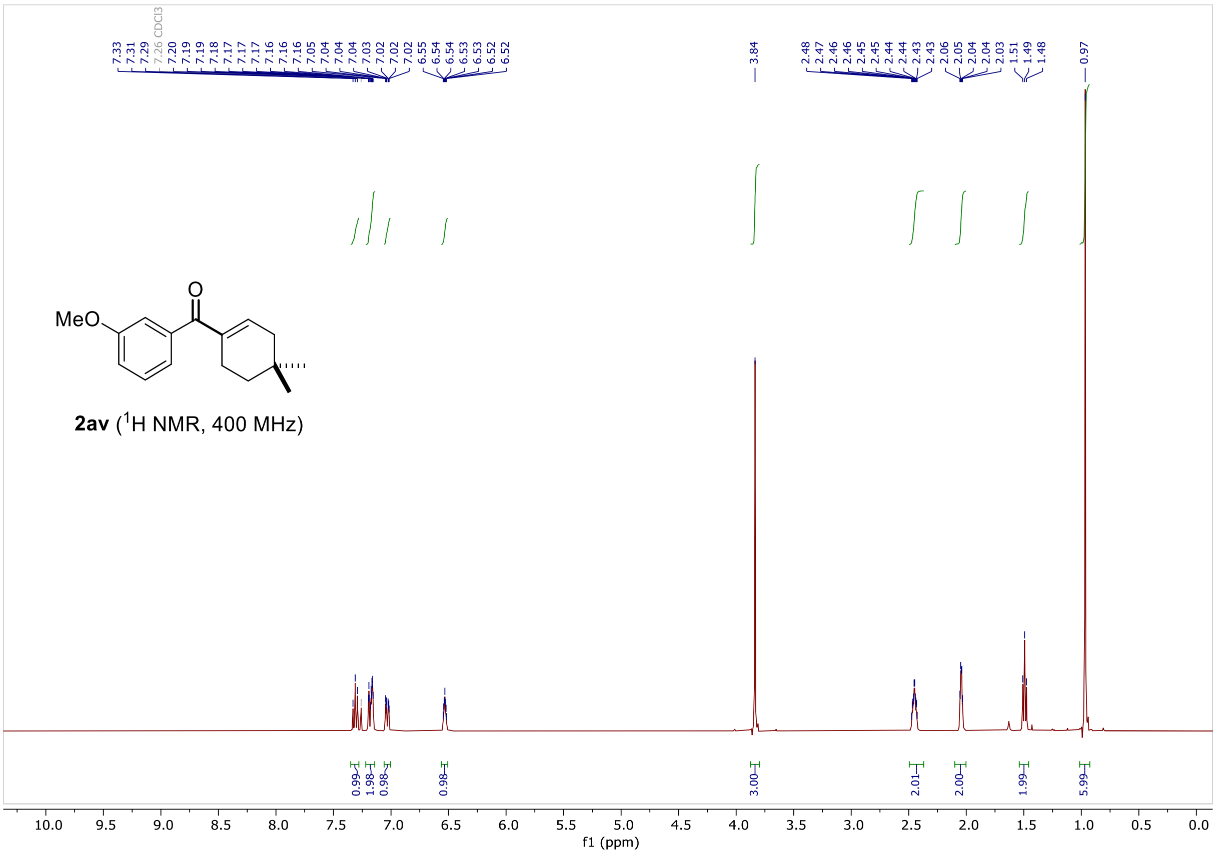

7.33 7.31 7.29 7.26 CDCl3 7.20 7.19 7.19 7.18 7.17 7.17 7.17 7.16 7.16 7.16 7.05 7.04 7.04 7.04 7.03 7.02 7.02 7.02 6.55 6.54 6.54 6.53 6.53 6.52 6.52 6.52
3.84
2.48 2.47 2.46 2.46 2.45 2.45 2.44 2.44 2.43 2.43 2.06 2.05 2.05 2.04 2.04 2.03 1.51 1.49 1.48
0.97
MeO
O
2av (¹H NMR, 400 MHz)
0.99 1.98 0.98 0.98 3.00 2.01 2.00 1.99 5.99
10.0 9.5 9.0 8.5 8.0 7.5 7.0 6.5 6.0 5.5 5.0 4.5 4.0 3.5 3.0 2.5 2.0 1.5 1.0 0.5 0.0
f1 (ppm)

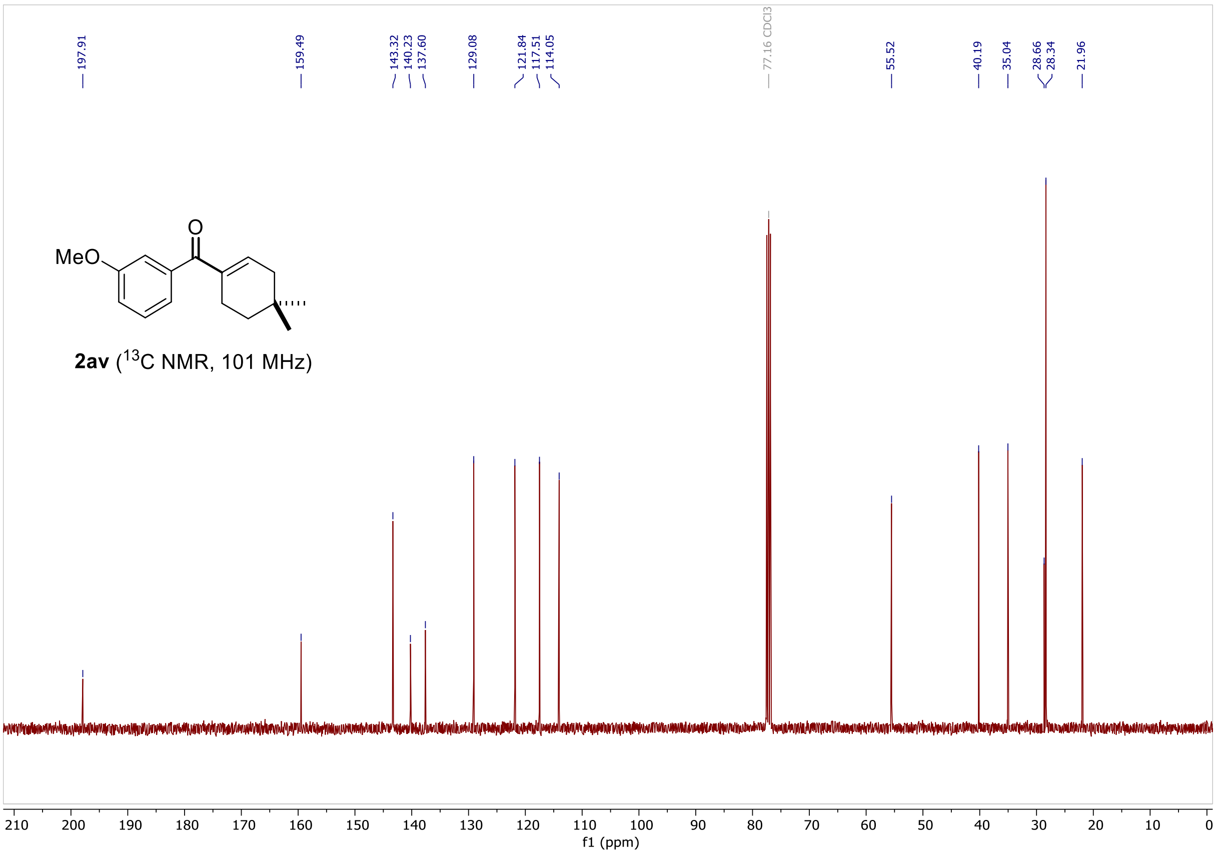

197.91
159.49
143.32 140.23 137.60
129.08
121.84 117.51 114.05
77.16 CDCl3
55.52
40.19
35.04
28.66 28.34
21.96
MeO
O
2av (¹³C NMR, 101 MHz)
210 200 190 180 170 160 150 140 130 120 110 100 90 80 70 60 50 40 30 20 10 0
f1 (ppm)

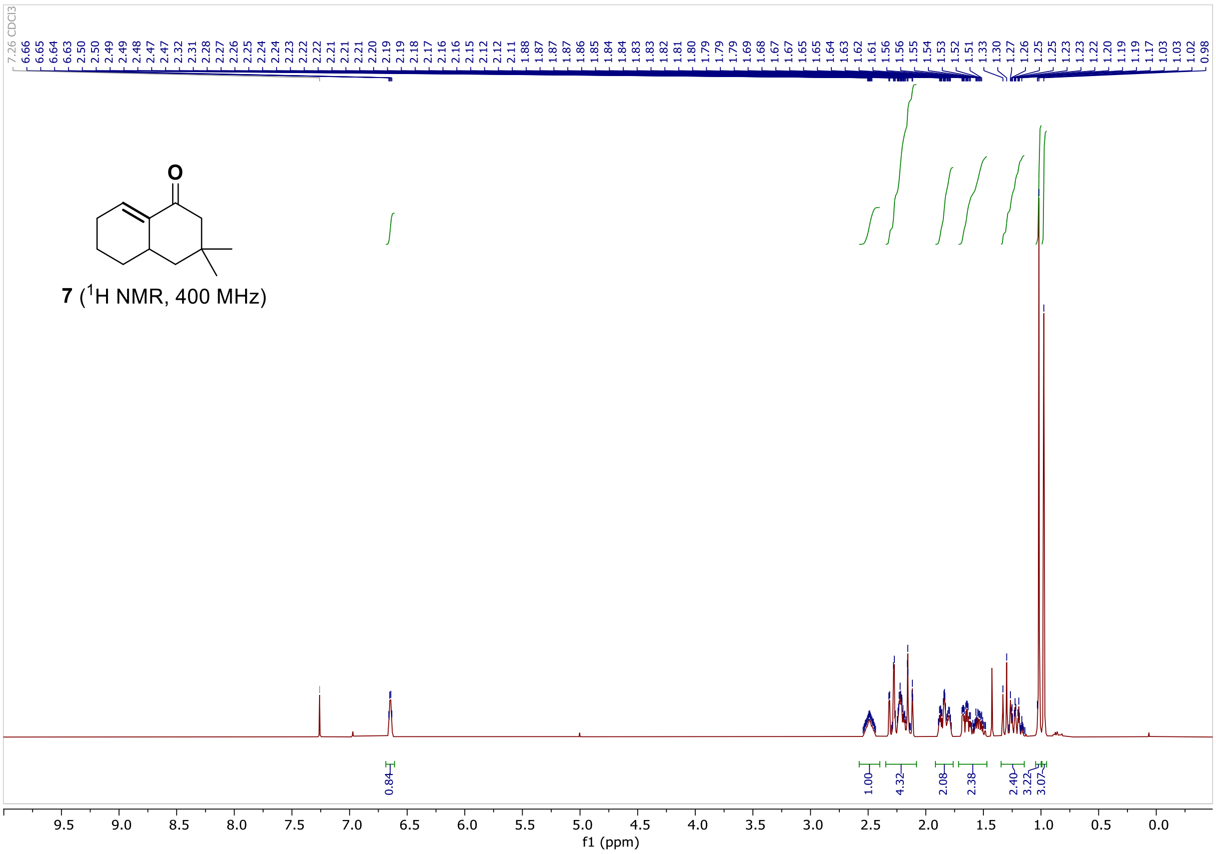

7 (^1H NMR, 400 MHz)

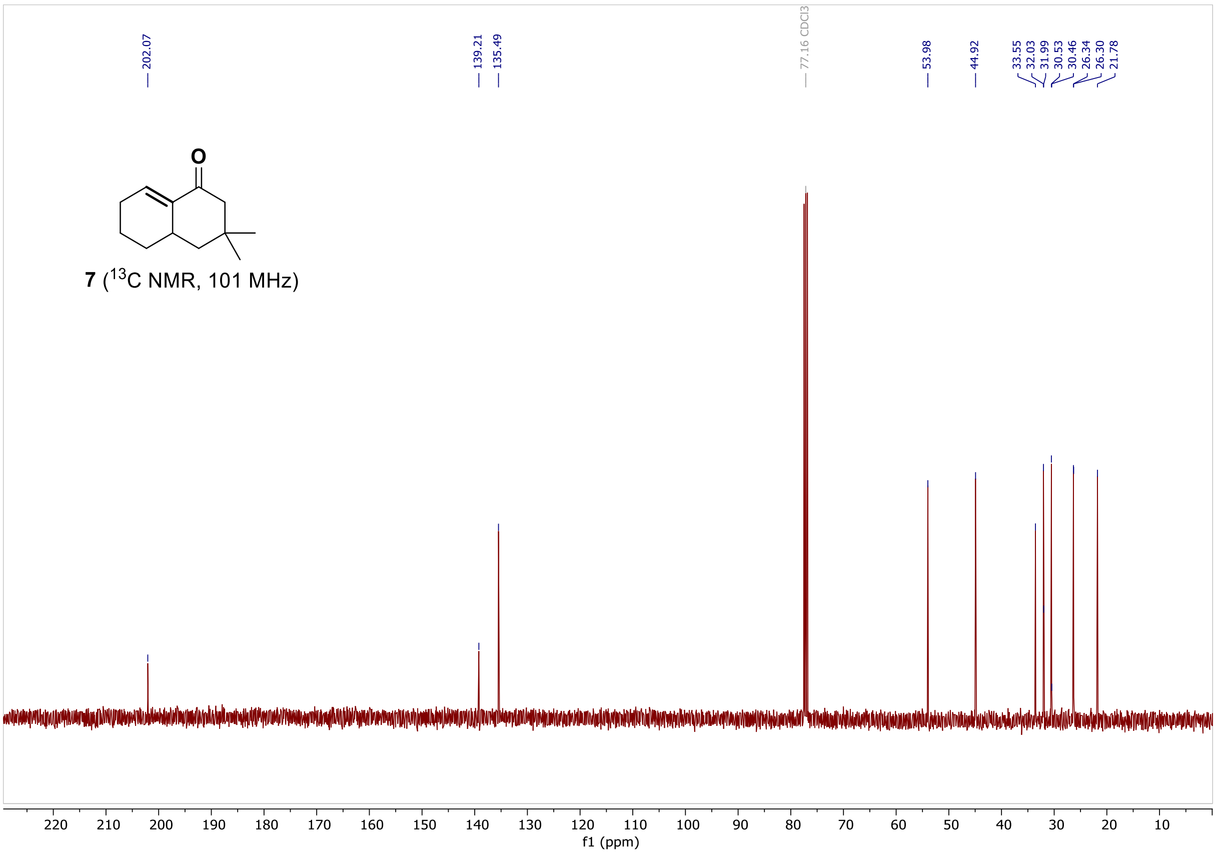

7 (^{13}C NMR, 101 MHz)

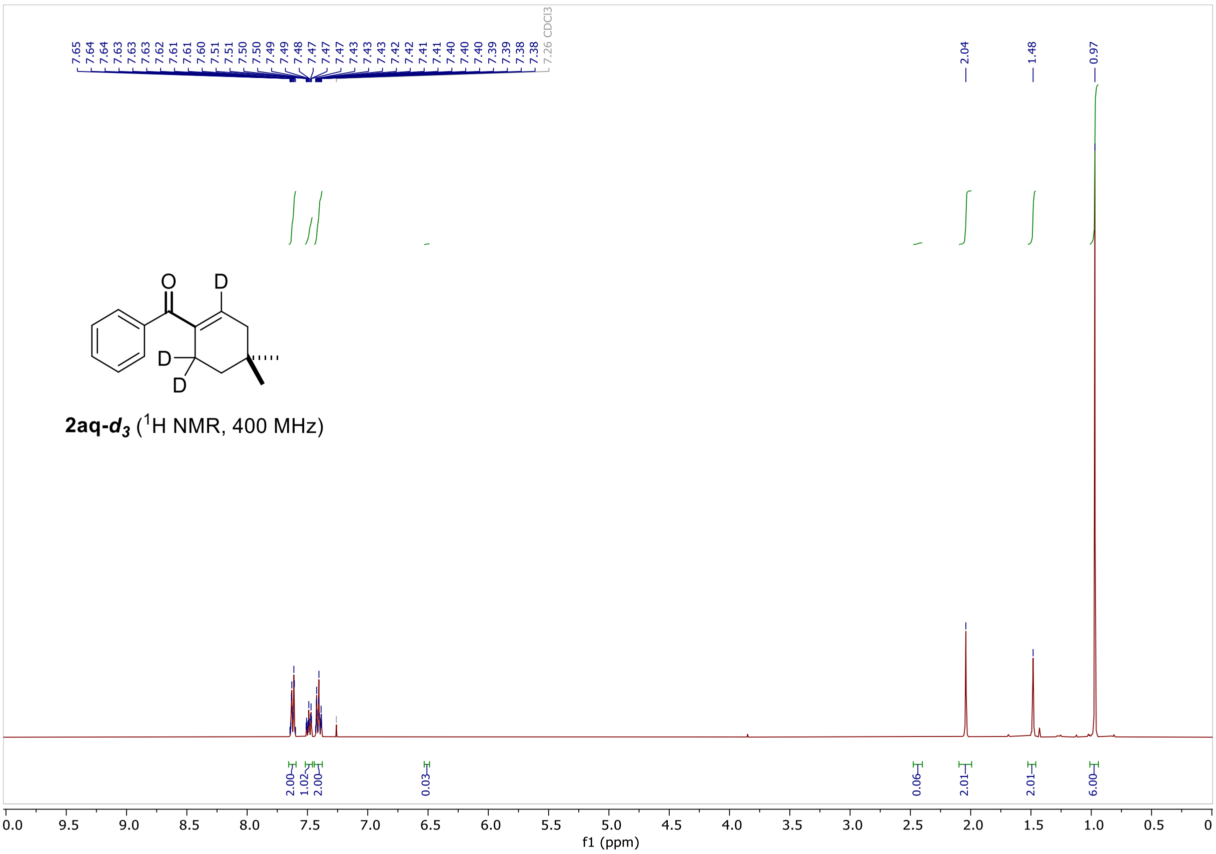

2aq-d_3 (^{1}H NMR, 400 MHz)
f1 (ppm)

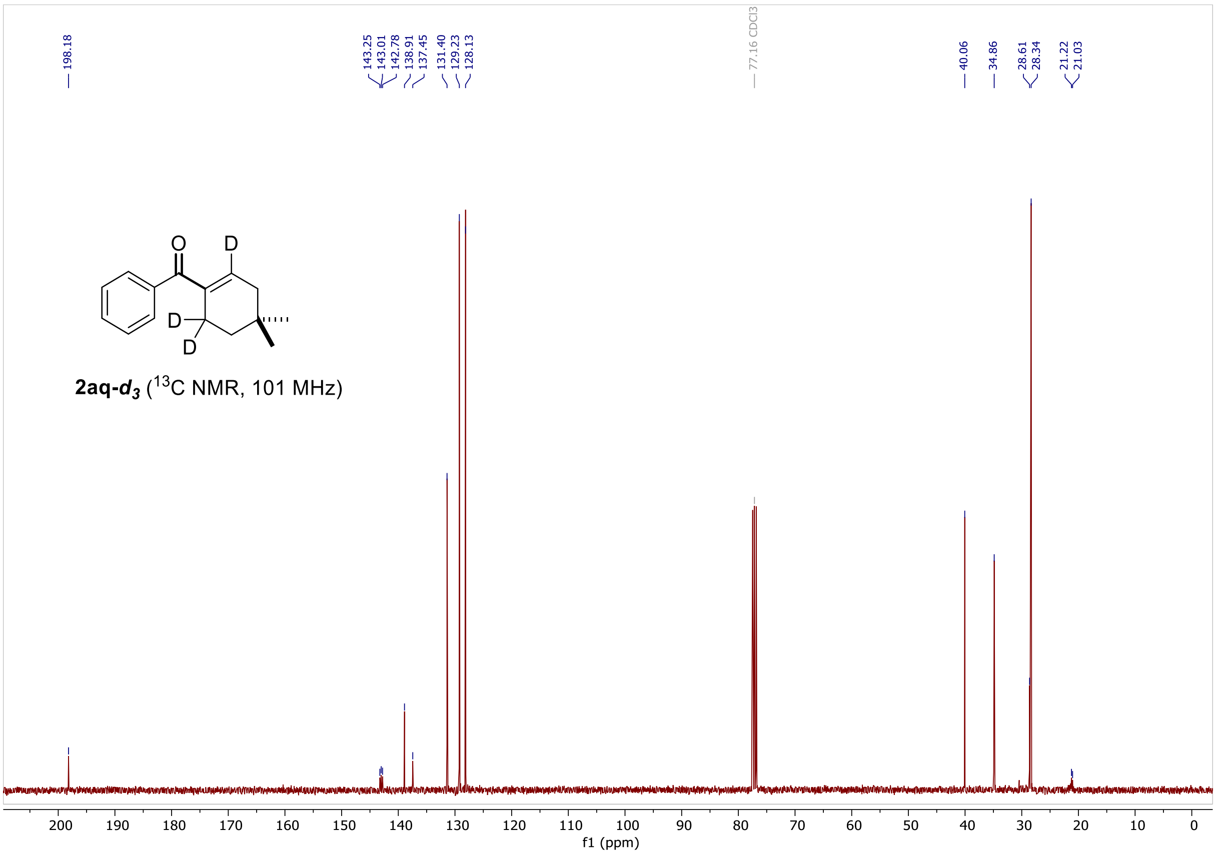

2aq-d_3 (^{13}C NMR, 101 MHz)
f1 (ppm)

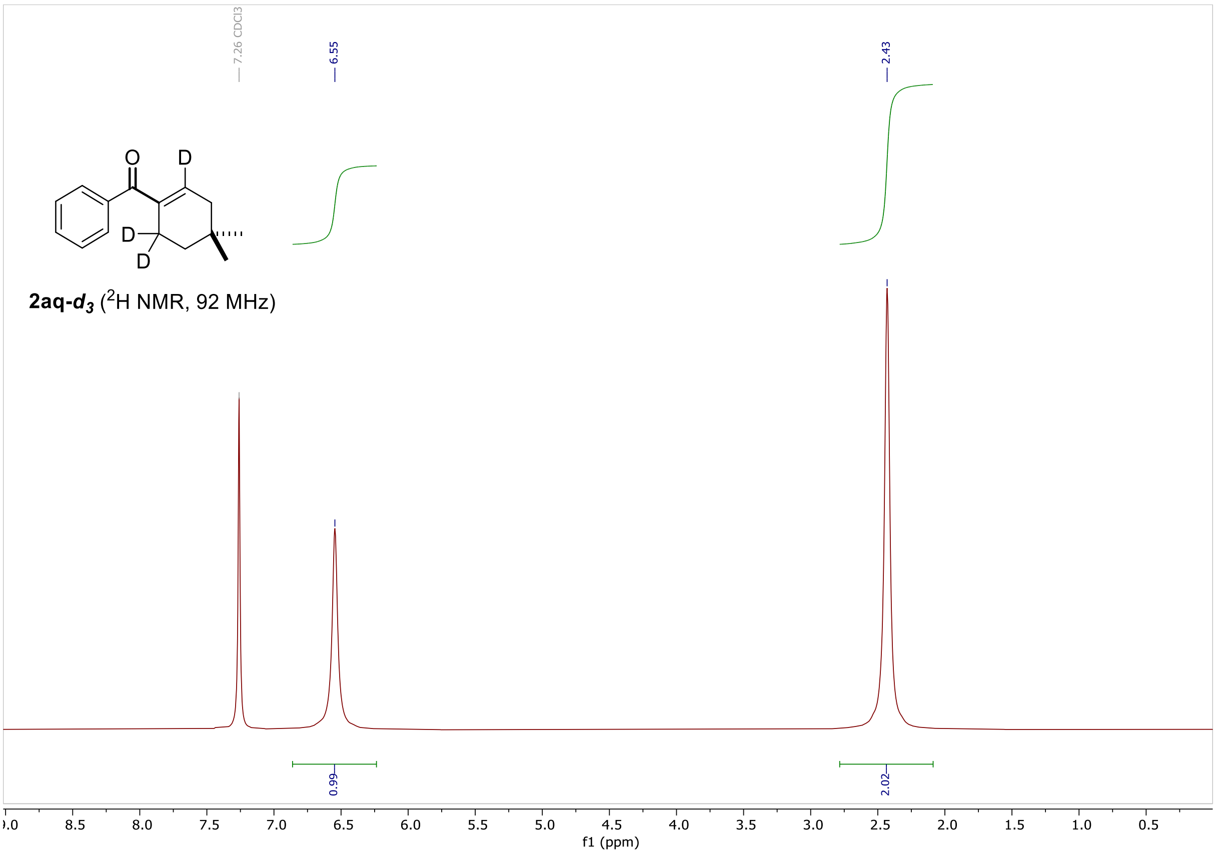

2aq-d_3 (^{2}H NMR, 92 MHz)
7.26 CDCl3
6.55
2.43
0.99
2.02
f1 (ppm)
